# Prenatal and childhood exposure to per-/polyfluoroalkyl substances (PFASs) and its associations with childhood overweight and/or obesity: a systematic review with meta-analyses

**DOI:** 10.1186/s12940-023-01006-6

**Published:** 2023-08-14

**Authors:** Gianfranco Frigerio, Chiara Matilde Ferrari, Silvia Fustinoni

**Affiliations:** 1 Environmental Cheminformatics, Luxembourg Centre for Systems Biomedicine (LCSB), University of Luxembourg, Campus Belval | House of Biomedicine II, 6 Avenue du Swing, L-4367 Belvaux, Luxembourg; 2 Department of Clinical Sciences and Community Health, University of Milan, Milano, Italy; 3 Occupational Health Unit, Fondazione IRCCS Ca’ Granda Ospedale Maggiore Policlinico, Milan, Italy

**Keywords:** Perfluoroalkyl substances, Fetal exposure, Early life exposure, Paediatric exposure, Childhood adiposity, Effect estimate conversion

## Abstract

**Background:**

Per-/polyfluoroalkyl substances (PFASs) are persistent organic pollutants and suspected endocrine disruptors.

**Objective:**

The aim of this work was to conduct a systematic review with meta-analysis to summarise the associations between prenatal or childhood exposure to PFASs and childhood overweight/obesity.

**Methods:**

The search was performed on the bibliographic databases PubMed and Embase with text strings containing terms related to prenatal, breastfeeding, childhood, overweight, obesity, and PFASs. Only papers describing a biomonitoring study in pregnant women or in children up to 18 years that assessed body mass index (BMI), waist circumference (WC), or fat mass in children were included. When the estimates of the association between a PFAS and an outcome were reported from at least 3 studies, a meta-analysis was conducted; moreover, to correctly compare the studies, we developed a method to convert the different effect estimates and made them comparable each other. Meta-analyses were performed also stratifying by sex and age, and sensitivity analyses were also performed.

**Results:**

In total, 484 and 779 articles were retrieved from PubMed and Embase, respectively, resulting in a total of 826 articles after merging duplicates. The papers included in this systematic review were 49: 26 evaluating prenatal exposure to PFASs, 17 childhood exposure, and 6 both. Considering a qualitative evaluation, results were conflicting, with positive, negative, and null associations. 30 papers were included in meta-analyses (19 prenatal, 7 children, and 4 both). Positive associations were evidenced between prenatal PFNA and BMI, between PFOA and BMI in children who were more than 3 years, and between prenatal PFNA and WC. Negative associations were found between prenatal PFOS and BMI in children who were 3 or less years, and between PFHxS and risk of overweight. Relatively more consistent negative associations were evidenced between childhood exposure to three PFASs (PFOA, PFOS, and PFNA) and BMI, in particular PFOS in boys. However, heterogeneity among studies was high.

**Conclusion:**

Even though heterogeneous across studies, the pooled evidence suggests possible associations, mostly positive, between prenatal exposure to some PFASs and childhood BMI/WC; and relatively stronger evidence for negative associations between childhood exposure to PFASs and childhood BMI.

**Supplementary Information:**

The online version contains supplementary material available at 10.1186/s12940-023-01006-6.

## Background

Childhood overweight and obesity are recognised worldwide issues. According to the World Health Organisation, 39 million children under 5 years were affected by overweight or obesity in 2020 and over 340 million aged 5–19 years were affected by overweight or obesity in 2016 [1]. The high prevalence of overweight and obesity is caused by a complex interaction between predisposing genetic factors and environmental factors [2, 3]. Unhealthy diet and physical inactivity are well-known causes leading to these problematic conditions [4]. However, other environmental factors that may contribute to the development of these conditions include exposure to endocrine-disrupting chemicals (EDCs) [5, 6]. Furthermore, the exposure to environmental pollutants may be critical, especially if it occurs in susceptible period of life, such as the prenatal period, or infancy [7], including lactation [8].

Per- and polyfluoroalkyl substances (PFASs) are a group of artificial compounds. Thanks to their surfactant, greaseproof, stain-proof, water repellent, and fire repellent properties, PFASs are used widely, including in food processing, medical articles, apparel, household products, electronics, and firefighting [9, 10]. PFAS pollution has been reported at global level since the beginning of this millennium, especially perfluorooctanoic acid (PFOA) and perfluorooctanesulfonic acid (PFOS) [11–13]. Despite the use of some PFASs have been restricted [14], these persistent compounds are still widespread in the environment and in living organisms [15–17]. PFASs are often classified as EDCs, i.e., they can potentially interfere with the endocrine system [18–20]. Some *in-vitro* studies showed that PFASs can disrupt some hormone functions [21, 22], and adipocyte differentiation [23]; moreover, effects on body weight and adipogenesis were observed in animals [24, 25], highlighting the importance of investigating these compounds in human epidemiological studies.

Several observational studies in humans were conducted to assess the effects of PFASs, and many studied their effect on prenatal growth: indeed, previous systematic reviews evidenced that a prenatal exposure to PFOA and PFOS is associated with a lower birth weight [26–28]. Several epidemiological studies also investigated the adverse effects of the exposure to PFASs on childhood overweight and obesity, and the evidence deriving from these studies were partially summarised in some narrative reviews [6, 29, 30]. Vrijheid et al., in their narrative review, classified the evidence of the effects of PFASs on childhood growth and obesity as “insufficient”, due to the low number of studies and the inconsistent findings [31]. Conversely, according to the narrative review conducted by Braun, a prenatal exposure to PFASs may be related to increased adiposity and risk of childhood overweight or obesity [18]. Szilagyi et al., in their narrative review, stated that in utero exposure to PFAS is associated with increased incidence of childhood obesity [32]. Rappazzo and co-workers carried out a systematic review of the epidemiologic literature evaluating the exposure to PFASs and different health outcomes, among which outcomes of overweight and obesity were described within the cardio-metabolic category [33]. Lee and co-workers wrote a systematic review summarising the evidence of early-life exposure to several PFASs and different outcomes in children, including adiposity, and concluded that, although the evidence was inconsistent, prenatal PFASs mostly have negative associations with BMI in the first 2 years of life and positive associations with adiposity in childhood and adolescence, the latter observation is true for PFOA in particular [34]. Ribeiro and co-workers conducted a systematic review with meta-analysis considering the exposure to different EDCs after the age of 2 years and their association with anthropometric measures of obesity or body fat, but only one study assessing PFASs was included [35]. Liu and co-workers published a systematic review with meta-analysed focused exclusively to early life exposure to PFOA, in which they pooled the evidence from 10 prospective cohort studies and found a positive association with an increased risk for childhood adiposity [36]. Finally, a recent comprehensive systematic review with meta-analysis conducted by Stratakis and co-workers summarised the evidence of prenatal exposure to persistent organic pollutants and childhood obesity considering different outcomes such as childhood BMI-z, waist circumference, and overweight risk; for PFOS and PFOA, they found no overall significant associations for most of the considered outcomes [37]. However, in those previous meta-analyses, effect estimates were extracted from the studies and compared irrespectively from their differences: indeed, comparisons between continuous data and categorical data were found, as well as comparisons between data obtained from different log-transformations. Considering the importance of assessing both prenatal and childhood exposure to PFASs and to include a higher number of PFASs, the aim of this work was to conduct a systematic review of the literature to critically summarise the existing evidence of the effect of a prenatal and childhood exposure to as many PFASs as possible on childhood overweight and obesity, with also an effort to develop a methodology to convert data from different studies to make effect estimates comparable each other before conducting the meta-analysis.

The PECO statement [38] for the present study is the following: (P) in children, what is the effect of (E) higher PFASs exposure during pregnancy or during childhood versus (C) lowest PFASs exposure during pregnancy or during childhood on (O) childhood overweight and obesity.

## Materials and methods

### Search strategy and inclusion criteria

This review and its protocol were registered on PROSPERO, the International prospective register of systematic reviews [39, 40].

The papers were searched in the PubMed and Embase bibliographic databases. Search terms included: prenatal, children, adolescents, breastfeeding, and synonyms; overweight, obesity, BMI, waist circumference, fat mass, and similar; perfluorates and related terms. The PFASs considered for the research terms were the most spread and the most interesting PFASs on our knowledge, including both legacy and emerging compounds, and have been described on our previous work [41]. The complete text strings are reported in supplementary material (Supplementary text), and they were elaborated thanks to the help of the library system staff of the University of Milan. Only articles published from 1^st^ January 2000 to 31^st^ December 2022 and in English language were considered. Articles retrieved were collected in electronic databases (Table S01 and Table S02). Duplicates of papers were identified using the DOI number and merged in a single line using the R software [42] with the “tidyverse” package [43], and then exported to an Excel database (Table S03). The script developed to carry out this merge is reported in the supplementary material (Supplementary text, R script, Sect. 1).

Two reviewers (G.F. and C.M.F.) independently read the titles and abstracts to select suitable papers for inclusion, while blinded to each other’s decisions. At the end of the process, discrepancies were discussed and evaluated with the final decision made by a third person acting as a supervisor (S.F.). To be eligible for inclusion, a human biomonitoring study quantifying one or more PFASs in biological samples (such as serum, plasma, urine, or breastmilk) of pregnant women or in children/adolescent up to 18 years old had to be conducted. Furthermore, the measured outcomes had to include a measure of overweight/obesity in children (up to 18 years old) such as BMI, waist circumference (WC), or fat mass/body fat percentage (BFP), which can be obtained with different approaches such as Dual-Energy X-ray Absorptiometry (DXA) (considered the gold standard) or skinfold thickness [44, 45]. The measurement of weight alone was not considered an outcome suitable for inclusion; likewise, birthweight and any other parameters measured right after delivery were not considered suitable for inclusion, as they are indicator of prenatal growth, which is not the purpose of this review. Non-original works (such as reviews), non-full articles (such as conference abstracts or letters to editor), and studies considering only non-pregnant adults were excluded.

Afterwards, papers judged suitable after reading their title and abstract were further inspected in their entirety. Finally, included papers were grouped in three categories: studies considering only prenatal exposure to PFASs, studies considering only exposure in children/adolescents, and studies considering both prenatal and children exposure.

### Data elaboration

Information from included papers were collected in the Excel database (Table S03). As for the paper selection, this elaboration was performed independently by G.F. and C.M.F. and discussed with S.F.. Information collected from the papers included: type of study, number of subjects, country or region, years of the first enrolment, measured PFASs and blood sampling period(s), outcome measured and period(s) of measurements, and if they were statistically positively or negatively associated with the considered outcomes.

Furthermore, each article was evaluated for the quality of its reporting: a list of 28 items was established, most of which following the STrengthening Reporting of Observational studies in Epidemiology-Molecular Epidemiology (STROBE-ME) [46], while others were created to specifically match the considered studies. The complete list of items is reported in the supplementary material (Table S03). For each item, either a 0 (not reported or not fulfilled), 0.5 (partially fulfilled) or 1 (fulfilled) was assigned. For each paper, the scores assigned to all the items were summed to evaluate the quality of reporting.

### Meta-analyses

#### Inclusion criteria

Aside from the extraction of the information reported in the previous section and the qualitative evaluation of studies, meta-analyses were performed among a subset of the included papers. Studies were eligible for inclusion in meta-analyses if they reported the estimated beta coefficient of the association between PFAS concentrations and the considered outcomes (BMI, WC, fat mass), along with the 95% confidence intervals (CI); or the association between PFAS concentrations and the risk of overweight/obesity, along with 95% CI. For each study, the estimates were considered separately if they were calculated considering the entire population (both sexes combined), only boys, or only girls. If the same cohort of subjects was considered in more than one paper, only one study was included in the meta-analyses. For each PFAS, outcome, and sex-category, a meta-analysis was performed only if at least three studies reported suitable estimates.

#### Data extraction

The slopes of the continuous associations were considered; when only slopes from categories (such as percentiles) were reported, we considered the highest estimate reported (worst-case scenario) [47]. For risk of overweight/obesity, both odds ratio (OR) and risk ratios (RR) were considered. If multiple models were reported, we considered the adjusted estimates from the model with the highest number of considered confounding factors. When the estimates were reported for outcomes measured at different time periods during infancy, the one related to the latest measurement was considered. For each study, estimates from subgroups (e.g., region of the study) were considered only if there were not overall estimates reported. If necessary, corresponding author of the paper was contacted.

#### Data conversion of the effect estimates

A specific methodology was developed to ensure that the beta coefficients and the confidence intervals included for meta-analysis were comparable each other. The conversions were performed to have, for all the included studies, beta estimates and coefficient intervals that represent the mean increase of z-scores of the outcome for each unit (ng/mL) increase in the PFAS.

Firstly, the standard error was calculated from the 95% CI as follow:

$$se\left(\beta \right)=\left(UL-LL\right)/3.92$$

Where $se\left(\beta \right)$ is the calculated standard error, $\mathrm{UL}$ is the upper limit, and $\mathrm{LL}$ is the lower limit of the 95% coefficient interval.

Then, four different steps of conversion were performed:

1. If the concentrations of PFASs were log-transformed before performing the linear models, the beta was changed according to the following formula elaborated by Rodríguez-Barranco and co-workers [48].

$${{\beta }_{f}= \mathrm{log}}_{b}\left(1+ \frac{1}{E\left[X\right]}\right)*{\beta }_{i}$$

While 95% CI was calculated as follows:

$${{95\mathrm{\%CI}}_{f}=\mathrm{log}}_{b}\left(1+ \frac{1}{E\left[X\right]}\right)*({\beta }_{i} \pm 1.96*se({\beta }_{i}))$$

Where ${\beta }_{f}$ and ${95\mathrm{\%CI}}_{f}$ are the converted beta and coefficient intervals, $b$ is the base of the logarithm used, $E\left[X\right]$ is the mean (if not available, the median) of the PFAS concentration, ${\beta }_{i}$ is the slope reported by the study and $se({\beta }_{i})$ is the standard error of the beta calculated as reported above.

2) If also the outcome variable were log-transformed and the effect estimates were reported as percent change; first, the beta was calculated from the variation percentage (var%) with the following formula:

$${\beta }_{i}= \mathrm{ln}((\mathrm{var\%}/100)+1)$$

and the standard error was calculated from the 95% CI of the var% as follow:

$$se({\beta }_{i})= \mathrm{ln}(((\mathrm{UL}-\mathrm{LL})/3.92/100)+1)$$

then, beta and 95% CI were calculated with the following equation elaborated by Rodríguez-Barranco et. al [48].

$${\beta }_{f}=\left({a}^{{\mathrm{log}}_{b}\left(1+ \frac{1}{E\left[X\right]}\right)*{\beta }_{i}}-1\right)*E[Y]$$

While 95% coefficients intervals were calculated as follows:

$${95\%\mathrm C\mathrm I}_f=\left(a^{\log_b\left(1+\frac1{E\left[X\right]}\right)\ast(\beta_i\pm1.96\ast se\left(\beta_i\right))}-1\right)\ast E\lbrack Y\rbrack$$

Where ${\beta }_{f}$ and ${95\mathrm{\%CI}}_{f}$ are the converted beta and coefficient intervals, $a$ is the base of the logarithm used for outcome data, $b$ is the base of the logarithm used for PFASs, $E\left[X\right]$ is the mean (or median, if mean was not reported) of the PFAS concentration, $E[Y]$ is the mean (or median, if mean was not reported) of the outcome measure, ${\beta }_{i}$ is the slope reported by the study and $se({\beta }_{i})$ is the standard error of the beta.

3) If the effect estimate was reported by change in interquartile range, the slope was divided by the interquartile range in order to have a feasible beta for unit increase. Analogously, if the effect estimate was reported by change of a specific tercile versus the reference tercile, it was divided by the difference of means of tercile. The standard error was also divided accordingly, and the coefficient intervals calculated again.
4) Finally, a further correction was carried out considering the type of data outcome used in each study. For BMI data, no change was made if the effect estimate was calculated from BMI z-score or BMI SDS, while if calculated from BMI expressed as kg/m^2^ the reported BMI mean (or median) was converted into BMI z-score using the Word Health Organization (WHO) references [49, 50], implementing the “anthro” R package for calculation [51], which uses the following formula [52]:

$$Z=\left[{\left(\frac{X}{M}\right)}^{L}-1\right]/LS$$

Where $Z$ is the calculated z-score, $X$ is the mean (or median) of the BMI (in kg/m^2^) reported by the study; while the value used for $L$, $M$, and $S$ were chosen from the WHO references [49, 50] considering the overall age of children in each study and the sex of subjects: if the effect estimate of a study reported separately girls or boys, only one z-score was calculated, while if the study included both males and females, two different z-scores were calculated, and then the mean of the two was considered. Then, the beta estimates and 95%CI were calculated with a proportion:

$$\begin{array}{c}{\beta }_{f}=(Z*{\beta }_{i})/M\\ se\left({\beta }_{f}\right)=|(Z*se\left({\beta }_{i}\right))/M|\\ {95\mathrm{\%CI}}_{f} = {\beta }_{f}\pm 1.96 * se({\beta }_{f})\end{array}$$

Where ${\beta }_{f}$, $se\left({\beta }_{f}\right)$, and ${95\mathrm{\%CI}}_{f}$ are the final beta, standard error, and coefficient intervals, respectively; $Z$ is the z-score calculated as reported above, ${\beta }_{i}$ and $se\left({\beta }_{i}\right)$ are the beta and standard error as converted so far, $M$ is the reported mean (or median) of the BMI (in kg/m^2^) Analogously, estimates obtained from waist circumference (in cm) were transformed using the tables reported by Sharma et al. [53]. For body fat, if expressed as body fat percentage, the mean (or median) was firstly converted as total fat index (kg/m^2^) by dividing the percentage per 100 and multiplying by mean (or median) BMI, then as fat mass index z-score using tables from Weber et al. [54].

All the above calculations were performed on the beta coefficients considering the associations between PFASs and BMI or WC. Instead, in order to properly make comparable the data related to the risk of overweight, the following procedure was applied to:

- If the data were reported as RR, they were converted as OR following a formula elaborated considering the one reported by [55], and explicating the OR:

$$OR= \frac{(RR-P0*RR)}{(1-P0*RR)}$$

Where OR is the odds ratio, RR is the risk ratio, P0 is the prevalence of overweight (expressed as proportion, so a number from 0 to 1) in the reference population (e.g: the first tercile or the first quartile) or in the total proportion (when data were reported as continuous). The same formula was applied to the lower and upper limit of the 95% CI.

b) Since OR is not a symmetrical measure, OR data were converted as standardized mean difference following a formula reported by [56]. In particular:

$$d=Log\left(OR\right)*\left(\frac{\sqrt{3}}{\pi }\right)$$

Where d is the standardized mean difference and Log(OR) is the logarithm (base 10) of the OR. The same formula was applied to the lower and upper limit of the 95% CI.

Finally, the obtained standardized mean differences were further converted using the same methodology reported above in steps 1) and 3) to suitably consider together PFAS concentrations calculated with different log-transformations and the different ranges of concentrations considered in each study.

All these elaborations were carried out by developing a specific R-script, which is reported in the supplementary material (Supplementary text, R script, Sect. 2).

#### Performing the meta-analyses

The generic inverse variance method was used, both fixed and random pooled estimates were calculated, heterogeneity was measured with the *I*^*2*^ statistics [57] and the between-study variance was calculated using the restricted maximum-likelihood estimator (REML) (τ^2^) [58]. To assess for the possible publication bias, a funnel plot was plotted [59] and an Egger’s test was carried out [60]. All these functions were conducted in the R environment, using the R package “meta” [61]. Forest plots and funnel plots were created using functions from the “meta” package. Moreover, in further separated meta-analyses, the summary estimates were also calculated by weighting the studies by sample size, instead of using the inverse variance method. The latter were carried out using the R package “rmeta” [62] (Supplementary text, R script, Sect. 3). Furthermore, sensitivity analyses were performed by repeating the meta-analyses described above by excluding one study at a time (Supplementary text, R script, Sect. 4).

Finally, all of the above (generic inverse variance method analyses, analyses weighting by sample size, and the sensitivity analyses removing one study at a time) were also performed separately considering studies which measured the outcomes in children who were 3 or less years from those measuring the outcomes in children who were more than 3 years (Supplementary text, R script, Sect. 5 and 6).

For visualisation, innovative superimposed forest plots were ideated and set up by developing an R script, implementing functions from the packages “tidyverse”, “grid” and “gridExtra” [43, 63], which is reported in the supplementary material (Supplementary text, R script, Sect. 7).

## Results

### Search results

**Fig. 1:**
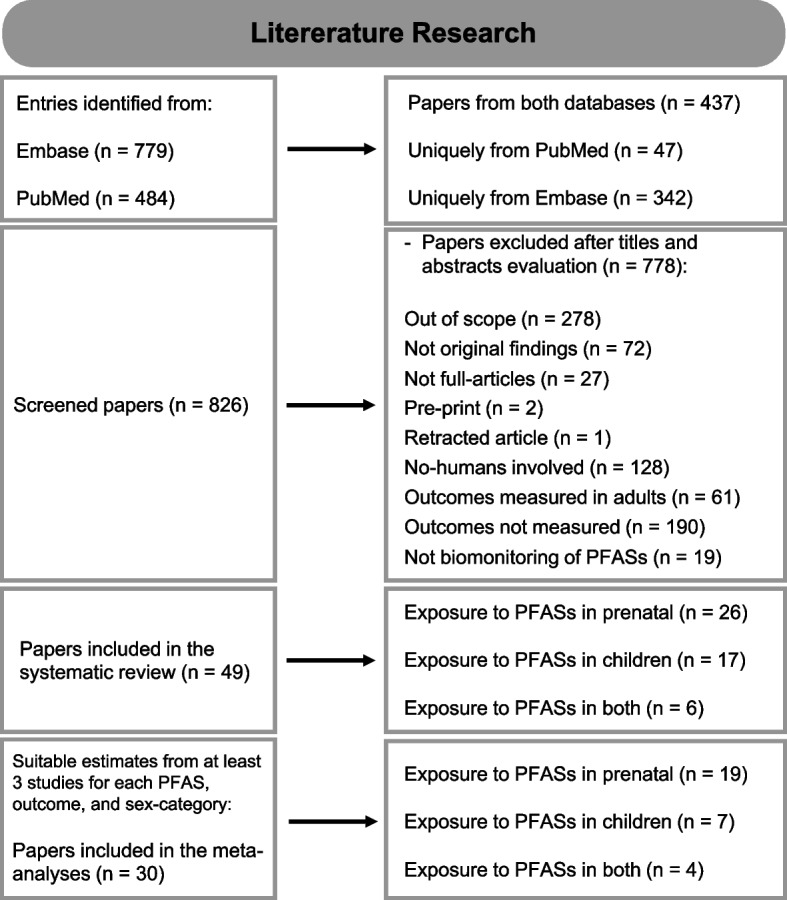
Summary of the literature search performed in this review

A total of 484 papers were retrieved from PubMed (Table S01) and 779 from Embase (Table S02), for a total of 1263 entries. Papers derived from both databases were 437, while 47 were uniquely retrieved from PubMed and 342 were unique from Embase; thus, after merging the duplicates, the papers considered for inclusion were 826. Titles and abstracts were evaluated and, in case, the papers were inspected in their full text. Several articles were not eligible for inclusion: 278 were out of scope and not relevant, 72 did not report original findings (such as reviews or project presentations), 27 were not full articles (such as letters or conference abstracts), 2 were pre-prints, and 1 was a retracted article. Furthermore, several studies did not meet the inclusion criteria for one or more reasons of the following: no humans involved (128); the outcomes were measured in human adults (61 of the remaining); while considering children or breastfeeding, the outcomes of interest were not measured (190 of the remaining); a biomonitoring study to measure exposure to PFASs was not performed (19). Hence, the total number of papers suitable for inclusion were 48. Later, an additional suitable paper was suggested by one of the external reviewers: it was not retrieved using the developed text string as it did not mention PFASs or related synonyms in title, abstract, or keywords, but suitable PFASs analyses were reported in the full text and in the supplementary material of the article [64]. Therefore, 49 papers were included in the systematic review, among which: 26 evaluated the prenatal exposure, 17 considered the exposure in children, and 6 both. A summary of the literature search results is given in Fig. 1, while the complete database is reported in the supplementary material (Table S03).

### Studies included in this review

A summary of the included papers is reported in Table 1 and 2. Overall, the enrolment periods ranged from 1986 to 2022. Several studies were conducted in Europe (24), among which most in Northern Europe (14) (Norway, Sweden, Denmark, Greenland, and Finland). Other studies were conducted in the United States (17), and a few in Asia (8). The number of subjects considered for the analyses varied greatly among studies, from only 54 to 9362 subjects. Among considered PFASs, PFOA and PFOS were monitored in almost all studies; PFNA, PFHxS, and PFDA were measured in 35, 33, and 21 studies, respectively; other measured PFASs were PFHpA, PFBS, and PFOSA measured in 12, 11, and 10 studies, respectively. For studies evaluating the prenatal exposure to PFASs, they were quantified in the blood of mothers at different periods during gestation, at birth, or a couple of weeks after birth; only one study evaluated the prenatal exposure in blood samples of children at birth. For studies assessing PFASs during childhood, they were quantified in children blood across various ages: from 1 to 18 years. BMI was measured in almost all studies, while WC and FT/BFP were measured in less than half of the studies. Outcomes were measured at different ages, from few weeks after birth to 18 years old.

**Table 1:** Summary of the studies included in this review considering a prenatal exposure to PFASs. For each study, information about the name of the study, country or region, year of the first enrolment, and number of subjects included in the statistical analyses were reported. Moreover, the information about PFASs (which ones monitored, when the samples were collected, and measured concentrations) is reported, as well as which outcomes were measured and when. The main associations found are also reported; in particular, each line represents a positive or a negative association between a compound and an outcome: an up arrow before the compound and an up arrow before the outcome indicates a positive association, while an up arrow before the compound and a downward arrow before the outcome indicates a negative association. Marginally significant associations are also reported (^m^). Finally, in the last column, it is reported if and which estimates were included in the meta-analyses

Reference	Name of the study	Country or region	Years of first enrolment	Number of subjects	Exposure assessment in mothers			Outcomes measured in children		Significant or marginally significant associations between exposure and outcomes	Data included in the meta-analyses
					**PFASs measured in blood**	**Sampling time**	**PFAS concentrations** **(ng/mL)**	**Type**	**Time**		
Papadopoulou et al., 2021 [65]	HELIX project	UK, France, Spain, Lithuania, Norway, Greece	from 1999 to 2010, across the 6 cohorts	1101	PFOA, PFNA, PFUnDA, PFHxS, PFOS	From mean (SD) week 14 (4) to 39 (1)	Median PFOA: 2.22 PFNA: 0.69 PFHxS: 0.53 PFOS: 6.15	WC	8 years (mean; range: 6 -12 years)	↑ PFAS mixture—↓ WC ↑ PFNA—↑ WC^m^	WC: PFOS, PFHxS, PFOA, PFNA (both sexes combined)
Braun et al., 2021 [66]	HOME study	Cincinnati, Ohio (U.S)	2003–2006	345	PFOA, PFOS, PFNA, PFHxS	16 weeks of gestation (*n* = 294, 85.2%), 26 weeks of gestation (*n* = 34, 9.9%), or within 24 h of delivery (*n* = 17, 4.2%)	Median PFOA: 5.5 PFOS: 13.8 PFNA: 0.9 PFHxS: 1.5	BMI	at 4 weeks and 1, 2, 3, 4, 5, 8, and 12 years	↑ PFOA—↓ BMI (infancy and early childhood) (non-monotonic) ↑ PFOA—↑ BMI (mid-childhood and adolescence) (non-monotonic) ↑ PFOS—↓ BMI (infancy, childhood, adolescence) ↑ PFHxS—↓ BMI (infancy, childhood, adolescence)	Data not used as, for the HOME study, data from Liu et al., 2020 were chosen for completeness
Gao, et al. 2022 [67]	Shanghai Birth Cohort	Shanghai, China	2013–2016	1350	PFOA, PFOS, PFNA, PFUA, PFDA, PFHxS, PFBS, PFDoA, PFHpA, PFOSA	From 9 and 16 weeks of gestation	Median PFOS: 9.68 PFOA: 11.66 PFNA: 1.77 PFDA: 1.82 PFUA: 1.48 PFHxS: 0.54 PFHpA: 0.06 PFDoA: 0.15 PFBS: 0.02 PFOSA: 0.00	WL WA	42 days, 6 months, 12 months, 24 months	↑ PFAS mixture—↓ WL high-rising and low rising group ↑ PFHxS—↓ WL high-rising group ↑ PFHpA—↓ WL low rising group ↑ PFOA—↑ WA low-rising group ↑ PFNA—↑ WA low-rising group ↑ PFDA—↑ WA low-rising group ↑ PFHxS—↑ WA low-rising group ↑ PFUA—↑ WA low-rising group ↑ PFAS mixture—↑ WA low-rising group	Not suitable data (WL not suitable)
Romano et al., 2022 [68]	New Hampshire Birth Cohort Study (NHBCS)	New England	2009–2018	418	PFHxS, n-PFOS, Sm-PFOS, n-PFOA, Sb-PFOA, PFNA, PFDA, PFUnDA, MeFOSAA,	~ 24–28 weeks’ gestation	Median PFOA: 1.44 PFOS: 4.00 MeFOSAA: < LOD PFDA: 0.20 PFHxS: 0.70 PFNA: 0.60 PFUnDA: < LOD	BMI	2 weeks, 1, 2, 4, 6, 9, and 12 months	↑ PFOA—↑ BMI at 12 months for girls and boys ^m^ ↑ PFNA—↑ BMI at 6 months for girls^m^ ↑ PFHxS—↑ BMI at 12 months for boys^m^ ↑ PFOS—↑ BMI at 6 months for girls	BMI: PFOA, PFOS, PFHxS, PFNA, PFDA, PFUA (only boys and only girls)
Zhang, Lei, et al., 2022 [69]	LWBC	Shandong, China	2010 – 2013	206	PFOS, PFOA, PFBS, PFHxS, PFDA, PFDoA, PFHpA, PFOSA, PFNA, PFUA	within 3 days before delivery	Median PFOA: 45.14 PFOS: 4.79 PFNA: 0.84 PFDA: 0.56 PFUA: 0.50 PFHxS: 0.33 PFDoA: 0.17 PFBS: 0.19 PFOSA: 0.13 PFHpA: 0.06	BMI WC FM/BFP WHtR	7 years	↑ PFHpA—↓ WC ↑ PFOSA—↓ FM/BFP ↑ PFHpA—↓ WC in boys ↑ PFHpA—↓ BMI in boys ↑ PFHpA—↓ FM in boys ↑ PFHpA—↓ WHtR in boys ↑ PFHpA—↑ BMI in girls ↑ PFOSA—↓ FM/BFP in boys ↑ PFOSA—↓ BMI in boys ↑ PFAS mixture—↓ FM/BFP ↑ PFAS mixture—↓ FM/BFP in boys ↑ PFAS mixture—↓ BMI in boys ↑ PFAS mixture—↓ WC in boys ↑ PFAS mixture—↓ WHtR in boys ↑ PFAS mixture—↑ FM in girls ↑ PFAS mixture—↑ BMI in girls ↑ PFAS mixture—↑ WC in girls ↑ PFAS mixture—↑ WHtR in girls	BMI: PFOA, PFOS, PFNA, PFHxS, PFDA, PFUA, PFDoA, PFBS (both sexes combined, only boys, and only girls) WC: PFOA, PFOS, PFNA, PFHxS, PFDA, PFUA (both sexes combined, only boys, and only girls)
Bloom et al., 2022 [70]	*Eunice Kennedy Shriver* NICHD Fetal Growth ECHO-FGS	U.S	2009–2013	803	PFHxS, PFOS, PFOSA, PFDS, PFHpA, PFOA, PFNA, PFDA, PFUnDA, PFDoDA	8–13 weeks gestation	Median PFHxS: 0.9 PFOS: 5.3 PFOA: 2.0 PFNA: 0.8 PFDA: 0.3 PFUnDA: 0.2	BMI WC FM/BFP	4–8 years	↑ PFUnDA—↑ WC, mother without obesity ↑ PFUnDA—↑ FM/BFP, mother without obesity ↑ PFOS—↑ BFP, mother without obesity ^m^ ↑ PFDA—↑ BMI, parous mother without obesity ↑ PFDA—↑ FM/BFP, parous mother without obesity ↑ PFOS—↓ BMI, mother with obesity ↑ PFDA—↓ BMI, mother with obesity ↑ PFOS—↓ FM/BFP, mother with obesity ↑ PFNA—↓ FM/BFP, mother with obesity ↑ PFDA—↓ FM/BFP, mother with obesity	BMI: PFHxS, PFOS, PFOA, PFNA, PFDA, PFUA (both sexes combined, only boys, and only girls) WC: PFHxS, PFOS, PFOA, PFNA, PFDA, PFUA (both sexes combined, only boys, and only girls) Overweight risk: PFHxS, PFOS, PFOA, PFNA (both sexes combined) Included only data from mothers without obesity
Martinsson et al., 2020 [71]	Southern Sweden Maternity Cohort	Malmö, Sweden	2003–2008	1048	PFOS, PFOA, PFHxS, PFNA	14 weeks of gestation	Median PFOS: 16.6 PFOA: 3.1 PFHxS: 0.7 PFNA: 0.4	BMI	4 years	No significant associations for the considered outcomes	Overweight risk: PFOS, PFOA, PFHxS, PFNA (both sexes combined)
Li, Liu et al., 2021 [72]	HOME Study	Cincinnati, Ohio (U.S)	2003–2006	221	PFOA, PFOS, PFNA, PFHxS	16 weeks of gestation (86% of women) 26-week of gestation (9.5%) or at delivery (4.5%)	Median PFOA: 5.3 PFOS: 12.9 PFNA: 0.9 PFHxS: 1.3	WC FM	12 years	↑ PFHxS—↑ WC ↑ PFOA—↑ WC^m^	Data not used as, for the HOME study, data from Liu et al., 2020 were chosen for completeness
Horikoshi et al., 2021 [73]	HBC study	Hamamatsu, Japan	2007 – 2012	597	PFOS and PFOA	at birth	Mean PFOS: 1.38 PFOA: 1.39	BMI	1, 4, 10, 18, 24, 32, 40, 50, and 66 months	↑ PFOS—↑ BMI increase while growing ^m^ ↑ PFOA—↑ BMI increase while growing	Not suitable data (increas of BMI SDS while growing, and not numerical data indicating needed estimates)
Lauritzen et al., 2018 [74]	NICHD-SGA	Norway and Sweden	1986–88	412 (254 Norway, 158 Sweden)	PFOA, PFOS	17–20 weeks of gestation	Median: PFOA: 1.64 (Norwegian), 2.33 (Swedish) PFOS: 9.62 (Norwegian), 16.3 (Swedish)	BMI FM/BFP	5 years	↑ PFOA—↑ BMI-for-age-and-sex z-score (only in Norway) ↑ PFOA—↑ FM Triceps skinfold z-score (only in Norway) ↑ PFOA—↑ OR Overweight (only in Norway) ↑ PFOS—↑ BMI BMI-for-age-and-sex z-score (all) ↑ PFOS—↑ FM Triceps skinfold z-score (all) ↑ PFOS—↑ OR Overweight (all)	BMI: PFOA, PFOS (both sexes combined) Overweight risk: PFOA, PFOS (both sexes combined)
Gyllenhammar et al., 2018 [75]	POPUP study	Uppsala County, Sweden	1996–2011	182–193	PFOA, PFNA, PFDA, PFUnDA, PFBS, PFHxS, and PFOS	3 weeks after delivery	Mean: PFOA 2.4 PFNA 0.46 PFDA 0.23 PFUnDA 0.19 PFBS 0.03 PFHxS 3.6 PFOS 14	BMI	at 3, 4, and 5 years	↑ PFOA—↑ BMI (3 and 4 years) ↑ PFNA—↑ BMI (3 and 4 years) ↑ PFHxS—↑ BMI (3 and 4 years) ↑ PFOS—↑ BMI (4 and 5 years)	BMI: PFOA, PFOS, PFNA, PFHxS (both sexes combined) (data asked to the author)
Zhang, Pan, et al., 2022 [76]	Shanghai Birth Cohort	Shanghai, China	2013–2016	2395	PFOA, PFOS, PFNA, PFDA, PFUA, PFDoA, PFHxS, PFHpA, PFBS, PFOSA	median gestational age of 15 weeks [interquartile range (IQR): 13–17 weeks]	Median PFOA: 11.62 PFOS: 9.38 PFNA: 1.68 PFDA: 1.72 PFUA: 1.40 PFHxS: 0.53 PFDoA: 0.17 PFBS: 0.03 PFHpA: 0.05	BMI WL	42 days (± 2 days), 6 months (mean ± SD: 6.4 ± 0.7 months), and 12 months	↑ PFBS—↓ WL ↑ PFBS—↓ BMI ↑ PFDoA—↑ WL ↑ PFDoA—↑ BMI	BMI: PFOA, PFOS, PFNA, PFDA, PFUA, PFHxS, PFDoA, PFBS (both sexes combined, only boys, and only girls)
Starling et al., 2019 [77]	Healthy Start Study	Colorado, (U.S)	2009–2014	415	PFOSA, N-EtFOSAA, N-MeFOSAA, PFHxS, PFOA, PFOS, PFNA	27 weeks of gestation, (median, range 20–34 weeks)	Median PFOA: 1.0 PFOS: 2.2 PFNA: 0.4 PFDA: 0.1 PFHxS: 0.7 MeFOSAA: 0.1	WL WA FM/BFP	5 months of age (average: 5.1 months, range: 2.8—9.4 months)	↑ PFOA—↑ FM/BFP (males) ↑ PFNA—↑ FM/BFP (males) ↑ PFOS—↓ FM/BFP ^m^ (females) ↑ PFOS—↓ WA (females)↑ PFOS—↓ WL (females) ↑ PFHxS—↓ WA (females) ↑ PFHxS—↑ rapid growth in WL ↑ N-MeFOSAA—↑ WA (both sexes combined and females) ↑ N-MeFOSAA—↑ rapid growth in WA ↑ N-MeFOSAA—↑ rapid growth in WL	Not suitable data (WL)
Hartman et al. 2017 [78]	ALSPAC	Avon, U.K	1991–1992	359 girls	PFOA, PFOS, PFHxS, PFNA	15 weeks of gestation (median; interquartile range: 10 and 28 weeks)	Median: PFOA: 3.7 PFOS: 19.7 PFHxS: 1.6 PFNA: 0.5	BMI WC FM/BFP	9 years	↑ PFOS—↓ BMI ↑ PFOS—↓ WC ↑ PFOA—↓ WC ↑ PFOA—↑ BFP (girls with mothers in the middle education group) ↑ PFOA—↓ BFP (girls with mothers in the highest education group) ↑ PFOS—↓ BFP(girls with mothers in the highest education group)	BMI: PFOA, PFOS, PFNA, PFHxS (only girls) WC: PFOA, PFOS, PFNA, PFHxS (only girls)
Sevelsted et al., 2022 [79]	COPSAC2010 birth cohort	Zealand, Denmark	2009–2011	675	PFOS and PFOA	pregnancy week 24 and 1 week postpartum	Median [IQR] PFOS: 6.24 [4.96–7.73] PFOA: 1.08 [0.78–1.47]	BMI BFP	6, 8, and 10 years	↑ PFOS—↓ BMI at 6 years in girls ↑ PFOS—↓ BFP at 6 years in girls ↑ PFOS—↑ BMI at 6 years in boys ^m^ ↑ PFOS—↑ BFP at 6 years in boys ^m^	BMI: PFOS, PFOA (both sexes combined)
Shoaff et al., 2018 [80]	HOME study	Cincinnati, Ohio (U.S)	2003–2006	334	PFOA, PFOS, PFNA, PHFxS	16 (86%) and 26 weeks gestation (9%), or within 48 h from delivery (5%)	Median: PFOA: 5.5 PFOS: 14 PFNA: 0.9 PFHxS: 1.5	BMI	4 weeks, 1 and 2 years old	↑ PFOA—↓ BMI ↑ PFOS—↓ BMI ↑ PFNA—↓ BMI ^m^ ↑ PFHxS—↓ BMI ^m^	Data not used as, for the HOME study, data from Liu et al., 2020 were chosen for completeness
Liu et al., 2020 [81]	HOME study	Cincinnati, Ohio (U.S)	2003–2006	212	PFOA, PFOS, PFNA, PFHxS	16 or 26 weeks of gestation or within 48 h from delivery	Median (during pregnancy): PFOA: 5.3 PFOS: 13.3 PFNA: 0.9 PFHxS: 1.3	BMI WC FM/BFP	12 years	↑ PFOA—↑ WtHr ↑ PFOA—↑ WC^m^ (non-linear) ↑ PFOA—↑ visceral fat area^m^ (non-linear) ↑ PFOA—↑ trunk fat percent^m^ ↑ PFOA—↑ android fat percent^m^ ↑ PFOA—↓ gynoid fat percent^m^ ↑ PFOA—↑ WC in girls ↑ PFOA—↑ WtHr in girls ↑ PFOA—↑ BFP in girls ↑ PFHxS—↑ WtHr ^m^ ↑ PFHxS—↑ WC ^m^ ↑ PFHxS—↑ visceral fat area ^m^ ↑ PFHxS—↑ trunk fat percent ^m^ ↑ PFHxS—↑ android fat percent ^m^ ↑ PFHxS—↓ gynoid fat percent ^m^ ↑ PFHxS—↑ WtHr in girls ↑ PFHxS—↑ android/gynoid fat percent ratio in girls ↑ PFHxS—↑ risk of overweight/obesity	BMI: PFOA, PFOS, PFNA, PFHxS (both sexes combined, only boys, and only girls) WC: PFOA, PFOS, PFNA, PFHxS (both sexes combined, only boys, and only girls)
Andersen et al., 2013 [82]	Danish National Birth Cohort	Denmark	1996–2002	811 (BMI) 804 (WC)	PFOA, PFOS	1^st^ and 2^nd^ trimesters of gestation, and at delivery	Median: PFOS: 33.8 PFOA: 5.25	BMI WC	7 years	↑ PFOA—↓ BMI ^m^ ↑ PFOA—↓ WC ^m^ (in boys) ↑ PFOS—↓ BMI^m^ ↑ PFOS—↓ WC^m^ (in boys)	BMI: PFOA, PFOS (only boys, and only girls) WC: PFOA, PFOS (only boys, and only girls)
Mora et al., 2017 [83]	Project Viva	Greater Boston, (U.S)	1999–2002	1645	PFOA, PFOS, PFHxS, PFNA	9.6 weeks of gestation (median)	Median: Children with early-childhood data PFOS: 24.8 PFOA: 5.6 PFHxS: 2.4 PFNA: 0.6 Median: Children with mid-childhood data PFOS: 24.7 PFOA: 5.6 PFHxS: 2.3 PFNA: 0.6	BMI WC FM/BFP	3.2 years (median; range: 2.9–6.1) for 1006 children (61%) 7.7 years (median; range: 6.6–10.9) for 876 children (53%)	↑ PFOS—↑ BMI (early childhood) ↑ PFHxS—↑ BFP (early childhood) ↑ PFOA—↑ WC (early childhood) ↑ PFOA—↑ WC (early childhood, boys) ↑ PFOS—↑ BMI (mid-childhood, girls) ↑ PFNA—↑ BMI (mid-childhood, girls) ↑ PFHxS—↑ BFP (mid-childhood, girls) ↑ PFNA—↑ BFP (mid-childhood) ↑ PFNA—↑ BFP (mid-childhood, girls)	BMI: PFOS, PFOA, PFHxS, PFNA (both sexes combined, only boys, and only girls) WC: PFOS, PFOA, PFHxS, PFNA (both sexes combined, only boys, and only girls) Overweight risk: PFOS, PFOA, PFHxS, PFNA (both sexes combined)
Braun et al., 2016 [84]	HOME Study	Cincinnati, Ohio (U.S)	2003–2006	204	PFOA, PFOS, PFNA, PFHxS	16 (*n* = 173, 87%) and 26 weeks of gestation (*n* = 19, 9%) and at delivery (*n* = 8, 4%)	Median: PFOA: 5.3 PFOS: 13 PFNA 0.9 PFHxS 1.4	BMI WC BFP	at 8 years and at 2, 3, 4, 5 years of age	↑ PFOA—↑ WC at 8 years (non-linear) ↑ PFOA—↑ BMI at 8 years (non-linear) ↑ PFOA—↑ BFP at 8 years (non-linear) ↑ PFOA—↑ BMI gains from 2 to 8 years	Overweight risk: PFOA, PFOS, PFNA, PFHxS (both sexes combined) Other data for the HOME study, taken from Liu et al., 2020 for completeness
Marks et al., 2021 [85]	ALSPAC	United Kingdom	1991 – 1992	301	PFOA, PFOS, PFHxS, PFNA, MeFOSAA, EtFOSAA, FOSA, PFDA	median of 15 (interquartile range (IQR): 10–28) weeks gestation	Median PFOA: 3.7 PFOS: 19.6 PFHxS: 1.6 PFNA: 0.49 FOSA: 0.20 MeFOSAA: 0.35 EtFOSAA: 0.70 PFDA: < LOD	BMI	2, 9, and 19 months	↑ overall endocrine disruptors—↓ BMI ^m^	Not suitable data (data were for weight-for-age z-score)
Jensen et al., 2020 [86]	OCC	Odense, Southern Denmark	2010–2012	602 (3 months) 530 (18 months)	PFOA, PFOS, PFHxS, PFNA, PFDA	before 16 weeks of gestation: median GA (IQR): 11.3 (9.9, 14.3) weeks	Median PFHxS: 0.30 PFOS: 8.04 PFOA: 1.62 PFNA: 0.66 PFDA: 0.26	BMI WC FM/BFP	3, and 18 months	↑ PFNA—↑ BMI (3 and 18 months) ↑ PFNA—↑ BMI (in girls, 3 and 18 months) ↑ PFNA—↑ BFP (3 months) ↑ PFDA—↑ BMI (3 and 18 months) ↑ PFDA—↑ BMI ^m^ (in girls, 3 and 18 months) ↑ PFDA—↑ BFP (3 months) ↑ PFOS—↑ BMI ^m^ (3 and 18 months)	BMI: PFOS, PFOA, PFHxS, PFNA, PFDA (both sexes combined, only boys, and only girls) WC: PFOS, PFOA, PFHxS, PFNA, PFDA (both sexes combined, only boys, and only girls)
Chen et al., 2019 [87]	Shanghai Prenatal Cohort	Shanghai, China	2012–2017	404	PFOA, PFOS, PFNA, PFDA, PFUnDA, PFHxS, PFOSA, PFDoA, PFBS, PFHpA	at birth	Median PFOS: 2.44 PFOA: 6.74 PFNA: 0.64 PFDA: 0.36 PFUA: 0.40 PFDoA: 0.09 PFHxS: 0.16 PFBS: 0.05	BMI WC WtHr FM/BFP	5 years	↑ PFBS—↑ WC (girls) ↑ PFBS—↑ WtHe (girls) ↑ PFBS—↑ FM (girls) ↑ PFBS—↑ BFP (girls) ↑PFDoA—↓ WC (girls) ↑PFDoA—↓ FM (girls) ↑PFDoA—↓ BFP (girls) ↑ PFNA—↑ BFP (boys)	BMI: PFOS, PFOA, PFHxS, PFNA, PFDA, PFUA, PFDoA, PFBS (both sexes combined, only boys, and only girls) WC: PFOS, PFOA, PFHxS, PFNA, PFDA, PFUA (both sexes combined, only boys, and only girls)
Chen et al., 2017 [88]	Taiwan Birth Panel Study	Taipei and New Taipei, Taiwan	2004–2005	429	PFOA, PFOS	At birth	Median PFOA: 2.6 PFOS: 7.6	BMI	at 4, 6, 12, 24, 60, 84 and 108 months	↑ PFOS—↓ BMI (for girls, time span from 6 to 12 months) ↑ PFOS—↓ BMI (for girls, time span from 12 to 24 months) ↑ PFOS—↑ BMI (for girls, time span from 60 to 108 months)	BMI: PFOS, PFOA (both sexes combined, only boys, and only girls)
Andersen et al., 2010 [89]	Danish National Birth Cohort	Denmark	1996–2002	1010	PFOS, PFOA	1^st^ and 2^nd^ trimesters of gestation, and at delivery	Median: PFOS 33.4 PFOA 5.21	BMI	5 months 12 months	↑ PFOS—↓ BMI ^m^ (5 months) ↑ PFOS—↓ BMI (12 months) ↑ PFOA—↓ BMI ^m^ (5 months) ↑ PFOA—↓ BMI ^m^ (12 months) ↑ PFOS—↓ BMI ^m^ (5 months, boys) ↑ PFOS—↓ BMI (12 months, boys) ↑ PFOA—↓ BMI (5 months, boys) ↑ PFOA—↓ BMI (12 months, boys)	BMI: PFOS, PFOA (both sexes combined) Data separated for sexes were not included as for those data of the Danish National Birth Cohort from Andersen et al., 2013 were considered instead
Karlsen et al., 2017 [90]	Not specified	Faroe Islands, Denmark	2007–2009	444 (at 18 months) 371 (at 5 years)	PFOS, PFOA, PFHxS, PFNA, PFDA	2 weeks after delivery	Median PFOS: 8.25 PFOA: 1.40 PFHxS: 0.20 PFNA: 0.66 PFDA: 0.26	BMI	at 18 months and 5 years	↑ PFOS—↑ BMI (18 months) ↑ PFOS—↑ RR overweight (18 months) ↑ PFOA—↑ overweight risk (5 years) ↑ PFOA—↑ BMI^m^ (18 months) ↑ PFHxS—↑ BMI ^m^ (18 months) ↑ PFDA—↓ overweight risk (5 years) (non-linear) ↑ PFNA—↓ overweight risk ^m^ (18 months and 5 years) (non-linear	BMI: PFOS, PFOA, PFHxS, PFNA, PFDA (both sexes combined) Risk overweight: PFOA, PFOS, PFHxS, PFNA (both sexes combined)
Yeung et al., 2019 [91]	Upstate KIDS Study	New York State (excluding New York City), (U.S)	2008–2010	2920 (1,954 singletons and 966 twins)	PFOA, PFOS	at birth in children (dried blood spot)	Mean PFOS 1.7 PFOA 1.1	BMI	4, 8, 12, 18, 24, 30, and 36 months	↑ PFOA—↓ BMI (in singletons) ↑ PFOS—↓ BMI (in singletons) ↑ PFOA—↓ BMI (in singleton girls) ↑ PFOS—↓ BMI (in singleton girls) ↑ PFOA—↓ BMI (in singleton boys, non-linearly) ↑ PFOA—↑ BMI (in twins, non-linearly)	BMI: PFOS, PFOA (both sexes combined, only boys, and only girls)
Manzano-Salgado et al., 2017 [92]	INMA birth cohort study	three Spanish regions: Gipuzkoa, Sabadell, and Valencia	2003–2008	1154 (6 months) 1230 (4 years) 1086 (7 years)	PFHxS, PFOS, PFOA, PFNA	1^st^ trimester of gestation	Geometric mean: PFHxS: 0.61 PFOS: 5.80 PFOA: 2.32 PFNA: 0.66	Weight gain from birth to 6 months BMI WC (only at 4 years for the Valencia and Sabadell subcohorts (*n* = 839, 68%))	6 months, 4, and 7 years	↑ PFOA—↑ weight gain to 6 months (boys) ↑ PFHxS—↓ weight gain to 6 months ^m^ ↑ PFHxS—↓ BMI ^m^ (overall and boys) (at 4 and 7 years) ↑ PFHxS—↓ WC ^m^ (overall and boys) (at 4 and 7 years) ↑ PFHxS—↑ BMI ^m^ (girls) (at 4 and 7 years) ↑ PFHxS—↑ WC ^m^ (girls) (at 4 and 7 years) ↑ PFOS—↑ BMI ^m^ (overall and boys) (at 4 and 7 years) ↑ PFOA—↑ BMI ^m^ (overall and boys) (at 4 and 7 years) ↑ PFNA—↑ BMI ^m^ (overall and boys) (at 4 and 7 years) ↑ PFOS—↑ WC ^m^ (at 7 years) ↑ PFNA—↑ BMI ^m^ (at 7 years) ↑ PFNA—↑ WC ^m^ (at 7 years)	BMI: PFOS, PFOA, PFHxS, PFNA (both sexes combined, only boys, and only girls) WC: PFOS, PFOA, PFHxS, PFNA (both sexes combined, only boys, and only girls) Risk overweight: PFOA, PFOS, PFHxS, PFNA (both sexes combined)
Alkhalawi et al., 2016 [93]	Duisburg Birth Cohort Study	North Rhine–Westphalia State (Germany)	2000–2002	156	PFOA, PFOS, PFHxS	32 weeks of gestation and at delivery	Geometric mean: PFOA: 2.43 PFOS: 9.04 PFHxS: 0.62	Ponderal index	4–5 weeks, 3–4 months, 6–7 months, and 12 months	↑ PFHxS—↑ Ponderal index (at 3–4 months)	Not suitable data (ponderal index not suitable)
Høyer et al., 2015 [94]	CLEAR and INUENDO studies	Greenland and Kharkiv (Ukraine)	2002–2004	1022	PFOA, PFOS	24 ± 10 weeks of gestation (mean ± SD)	Median PFOA: 1.3 PFOS: 10.8 Greenland: PFOA: 1.8 PFOS: 20.2 Ukraine: PFOA 1.0 PFOS 5.0	BMI WHtR	between 5 and 9 years old	↑ PFOA—↑ WtHe ^m^ ↑ PFOS—↑ WtHe ↑ PFOA—↑ overweight (girls, Greenland) ↑ PFOS—↑ WtHe (girls, ↑ PFOA—↑ BMI (Ukraine) ↑ PFOA—↑ WtHr (girls, Greenland)	BMI: PFOS, PFOA Greenland and Ukraine separated (both sexes combined) Overweight risk: PFOA, PFOS (both sexes combined)
Cai et al., 2023 [95]	FLEHS	Belgium	2002–2004 and 2008–2009	346	PFOA, PFOS	Right after birth	Median PFOA: 1.5 PFOS: 2.7	BMI	4–8 years	No significant associations for the considered outcomes﻿	Not suitable data (BMI change not suitable)
Vrijheid et al., 2020 [64]	HELIX	Europe	2013 – 2016	1301	PFOA, PFNA, PFUnDA, PFHxS, PFOS	During pregnancy	Inter Quartile Ranges PFOA: 2.0 PFNA: 0.66 PFUnDA: 0.18 PFHxS: 0.6 PFOS: 5.5	BMI WC FM/BFP	6–11 years	No significant associations for the considered outcomes﻿	BMI: PFOS, PFOA, PFHxS, PFNA, PFUA (both sexes combined) (mean concentrations of PFASs asked to the authors)

**Table 2:** Summary of the studies included in this review considering a childhood exposure to PFASs. For each study, information about the name of the study, country, year of the first enrolment, and number of subjects included in the statistical analyses were reported. Moreover, the information about PFASs (which ones monitored, when the samples were collected, and measured concentrations) is reported, as well as which outcomes were measured and when. The main associations found are also reported; in particular, each line represents a positive or a negative association between a compound and an outcome: an up arrow before the compound and an up arrow before the outcome indicates a positive association, while an up arrow before the compound and a downward arrow before the outcome indicates a negative association. Marginally significant associations are also reported (^m^). Finally, in the last column, it is reported if and which estimates were included in the meta-analyses

Reference	Name of study	Country and region	Years of first enrolment	Number of subjects	Exposure assessment in children			Outcomes measured in children		Significant or marginally significant associations between exposure and outcomes	Data included in the meta-analyses
					**PFASs measured in blood**	**Sampling time**	**PFASs concentrations** **(ng/mL)**	**Type**	**Time**		
Papadopoulou et al., 2021 [65]	HELIX project	UK, France, Spain, Lithuania, Norway, Greece	from 1999 to 2010, across the 6 cohorts	1101	PFOA, PFNA, PFUnDA, PFHxS, PFOS	From mean (SD) age 7 (0.5) to 11 (0.6) years	Median PFOA: 1.53 PFNA: 0.47 PFUnDA: 0.06 PFHxS: 0.34 PFOS: 1.93	WC	8 years (mean; range: 6 -12 years)	↑ PFAS mixture—↓ WC ↑ PFOA—↓ WC	Data not included as for the HELIX project, data from Vrijheid et al., 2020 considered a larger population
Geiger et al., 2021 [96]	NHANES	U.S	1999–2000, 2003–2012	2473	PFOA, PFOS	12–18 years	Mean PFOA: 3.79 PFOS: 15.66	BMI WC	12–18 years	↑ PFOA—↑ BMI ^m^ ↑ PFOS—↑ BMI (significant in the unadjusted model) ↑ PFOS—↑ WC (significant in the unadjusted model)	Overweight risk: PFOA, PFOS (both sexes combined)
Averina et al., 2021 [97]	Fit Futures study	Northern Norway	2010–2011	940	PFBS, PFPS,PFHxS, PFHpS, PFOS, PFNS, PFDS, PFDoDS, PFOSA, PFHxA, PFHpA, PFOA, PFNA, PFDA, PFUnDA, PFDoDA, PFTrDA, PFTeDA	Mean (SD) 16.3 (1.1) – 16.5 (1.4)	Mean (SD) PFBS: 0.005 (0.002) PFPS: 0.01 (0.007) PFHxS: 0.02 (0.01) PFHxA: 0.04 (0.04) PFHpS: 0.01 (0.01) PFHpS: 0.07 (0.06) PFOS: 0.04 (0.05) PFOA: 0.30 (0.13) PFNS: 0.02 (0.02) PFNA: 0.03 (0.01) PFDS: 0.02 (0.012) PFDA: 0.03 (0.01) PFUnDA: 0.03 (0.02) PFDoDS: 0.03 (0.035) PFDoDA: 0.02 (0.02) PFTrDA: 0.03 (0.030) PFTeDA: 0.08 (0.089) PFOSA: 0.01 (0.010)	BMI	Mean (SD) 16.3 (1.1) – 16.5 (1.4)	↑ PFHxS—↑ obesity ↑ PFHpS—↑ obesity	Not suitable data (not directly associating with BMI)
Canova et al., 2021 [98]	Veneto Region Health Surveillance Program	Italy	2017–2019	9362 (6669 adolescents and 2693 children)	PFOS, PFOA, PFHxS, PFNA, PFHpA, PFBS, PFHxA, PFBA, PFPeA, PFDeA, PFUnA, PFDoA	14–19 years (adolescents) and 8–11 (children)	Mean (SD) in adolescents: PFOA: 51.6 (47.2) PFOS: 4.1 (3.5) PFHxS: 3.6 (2.9) PFNA: 0.5 (0.3) Mean (SD) in children: PFOA: 26.2 (21.5) PFOS: 2.6 (2.5) PFHxS: 2.2 (1.5) PFNA: 0.4 (0.2)	BMI	14–19 years (adolescents) and 8–11 (children)	↑ PFOS—↓ BMI (adolescents and children) ↑ PFOA—↓ BMI (children) ↑ PFHxS—↓ BMI (children) ↑ PFNA—↓ BMI (children)	BMI: PFOS, PFOA, PFHxS (both sexes combined, only boys, and only girls) Included only data about adolescents (and not about children)
Janis et al., 2021 [99]	Project Viva	eastern Massachusetts, U.S	1999–2022	537	n-PFOA, total PFOA, n-PFOS, Sm-PFOS, total PFOS, PFDA, PFHxS, MeFOSAA, PFNA	6–10 years	Median (IQR) PFOA: 4.5 (3.0) PFOS: 6.4 (5.9) PFDA: 0.3 (0.3) PFHxS: 1.9 (2.4) MeFOSAA: 0.3 (0.5) PFNA: 1.5 (1.2)	BMI FM	6–10 years and 11–16 years	↑ PFOS—↓ BMI increase from mid-childhood to early adolescents ^m^ ↑ PFOS—↓ FM increase from mid-childhood to early adolescents ↑ PFHxS—↓ BMI increase from mid-childhood to early adolescents ^m^ ↑ PFHxS—↓ FM increase from mid-childhood to early adolescents ↑ PFDA—↑ FM increase from mid-childhood to early adolescents ^m^ ↑ PFNA—↑ FM increase from mid-childhood to early adolescents ^m^ ↑ PFDA—↑ FM increase from mid-childhood to early adolescents in boys ↑ PFAS mixture—↓ lean mass increase from mid-childhood to early adolescents	Not suitable data (data expressed as BMI change)
Fassler et al., 2019 [100]	BCERP	Cincinnati (U.S)	2004–2006	353 (only girls)	N-MeFOSAA, PFDA, PFNA, PFOA, PFOS, PFHxS	6–8 years	Median Me-PFOSA-AcOH: 0.8 PFDeA: 0.30 PFHxS: 5.20 PFNA: 1.40 PFOA: 7.30 PFOS: 13.60	BMI WtHe WtHi FM	6–8 years	↑ PFOA—↓ BMI ↑ N-MeFOSAA—↓ BMI ↑ PFDeA—↓ BMI ↑ PFOS—↓ BMI ↑ PFOA—↓ WtHe ↑ N-MeFOSAA—↓ WtHe ↑ PFDeA—↓ WtHe ↑ PFOS—↓ WtHe ↑ PFOA—↓ FM	Not suitable data (no confidence intervals)
Li, Liu, et al. 2021 [72]	HOME Study	Cincinnati, Ohio (U.S)	2003–2006	221	PFOA, PFOS, PFNA, PFHxS	3, 8, and 12 years	Median PFOA: from 1.3 to 5.4 PFOS: from 2.4 to 6.2 PFNA: from 0.3 to 1.3 PFHxS: from 0.7 to 1.9	WC FM	12 years	No significant associations for the considered outcomes﻿﻿	Data not used as, for the HOME study, data from Liu et al., 2020 were chosen for completeness
Thomsen et al., 2021 [101]	Copenhagen Mother–Child Cohort	Copenaghen, Denmark	2009	109	PFOA, PFOS, PFHxS, PFNA, and PFDA	Median (25^th^-75^th^ percentiles) 12.6 years (11.5–13.2)	Median (25^th^-75^th^ percentiles) PFOA: 2.79 (2.18–3.58) PFOS: 6.81 (5.85–9.47) PFHxS: 0.50 (0.40–0.65) PFNA: 0.92 (0.73–1.17) PFDA: 0.31 (0.25–0.37)	BFP	Median (25^th^-75^th^ percentiles) 12.6 years (11.5–13.2)	↑ PFOS—↓ BFP ^m^ ↑ PFDA—↓ BFP ^m^ ↑ PFNA—↓ BFP ^m^ ↑ PFOA—↑ BFP ^m^ ↑ PFHxS—↓ BFP^m^	Not suitable data (associations with DXA body fat, not enough from other studies to perform a meta-analysis)
Koponen et al., 2018 [102]	birth cohort study LUKAS2	Eastern Finland	2005–2015	54	PFHxA, PFHpA, PFOA, PFNA, PFDA, PFUnDA, PFDoDA, PFTrDA, PFTeDA, PFHxS, PFHpS, PFDS; N-MeFOSAA, N-EtFOSAA, 6:2 diPAP, 8:2 diPAP	1, 6 and 10.5 years	Median PFOS: 1.5—6.3 PFOA: 1.4—7.1 PFNA: 0.35—0.84 PFHxS: 0.20—0.49	BMI	1, 6, and 10.5 years	No significant associations for the considered outcomes﻿	Not suitable data (no suitable estimates reported)
Harris et al., 2017 [103]	Project Viva	Boston—urban and suburban Eastern Massachusetts (U.S)	2007–2010	653	PFOS, PFOA, PFHxS PFNA, N-EtFOSAA N-MeFOSAA, PFDA, PFOSA	7.7 years (median; range: 6.6 − 10.6)	Median PFOS: 6.2 PFOA: 4.4 PFHxS: 1.9 PFNA: 1.5 Et-PFOSA-AcOH:LOD Me-PFOSA-AcOH: 0.3 PFDeA: 0.3 FOSA: < LOD	BMI	7.7 years (median; range: 6.6 − 10.6)	↑ PFOA—↓ BMI ↑ PFDeA—↓BMI	Not suitable data (estimates as PFASs concentration change depending on the outcome, instead of the contrary)
Scinicariello et al., 2020 [104]	NHANES	U.S	2013–2014	600	PFOA, PFNA, PFHxS, PFOS	3–11 years	Geometric mean PFOA: 1.92 PFNA: 0.80 PFHxS: 0.85 PFOS: 3.90	BMI	3–11 years	↑ PFOA—↓ BMI ^m^ ↑ PFHxS—↓ BMI ^m^ ↑ PFOS—↓ BMI ^m^ ↑ PFOA—↓ BMI (boys) ↑ PFHxS—↓ BMI (boys)	BMI: PFOS, PFOA, PFHxS, PFNA (both sexes combined)
Sevelsted et al., 2022 [79]	COPSAC2010 birth cohort	Zealand, Denmark	2009–2011	533	PFOS and PFOA	6 and 18 months	Median [IQR]: PFOS: 5.29 [4.05–6.94] PFOA 2.33 [1.40–3.56]	BMI BFP	6, 8, and 10 years	No significant associations for the considered outcomes﻿	BMI: PFOS, PFOA (both sexes combined)
Liu et al., 2020 [81]	HOME Study	Cincinnati, Ohio (U.S)	2003–2006	212	PFOA, PFOS, PFNA, PFHxS	at birth and ages 3, 8 and 12 years	Median PFOA: 5.4 (3 y), 2.5 (8 y), 1.3 (12 y) PFOS: 6.2 (3 y), 3.6 (8 y), 2.4 (12 y) PFNA: 1.3 (3 y), 0.7 (8 y), 0.3 (12 y) PFHxS: 1.9 (3 y), 1.2 (8 y), 0.7 (12 y)	BMI WC FM/BFP	12 years	No significant associations for the considered outcomes﻿	BMI: PFOS, PFOA, PFHxS (both sexes combined, only boys, and only girls) PFNA (both sexes combined)
Timmermann et al., 2014 [105]	EYHS	Odense, Denmark	1997	499	PFOA, PFOS	8–10-year-old children (third-grade students)	Median PFOS: 41.5 PFOA: 9.3	BMI WC FM/BFP	8–10-year-old children (third-grade students)	↑ PFOS—↓ FM/BFP in girls^m^ ↑ PFOS—↓ FM/BFP in boys^m^	BMI: PFOS, PFOA (both sexes combined)
Li, Li et al., 2021 [106]	-	Shanghai, China	2019	189	PFHxA, PFHpA, PFOA, PFNA, PFDA, PFUnA, PFBS, PFHxS, PFOS, PFDS	8–12 years	Mean PFHxA: 21.06 PFHpA: 129.57 PFOA: 117.34 PFNA: 28.78 PFDA: < LOD PFUnA: < LOD PFBS: 21.57 PFHxS: 27.66 PFOS: 38.54 PFDS: < LOD ΣPFAAs: 394.83	BMI	8–12 years	↑ PFHpA—↓ children with overweight/obesity ↑ PFBS—↓ children with overweight/obesity ↑ PFOS—↓ children with overweight/obesity	Not suitable data (no estimates with BMI)
Ye et al.; 2018 [107]	NHANES	U.S	2013–2014	639	PFOSA, N-MeFOSAA, N-EtFOSAA, PFBS, PFHxS, PFHpA, PFNA, PFDA, PFUnDA, PFDoDA, n-PFOA, Sb-PFOA, n-PFOS, Sm-PFOS	3–11 years	Median ΣPFOS: 3.75 ΣPFOA: 1.94 PFHxS: 0.810 PFNA: 0.700 n-PFOS: 2.47 Sm-PFOS: 1.28 n-PFOA: 1.82 Sb-PFOA: < LOD PFBS: < LOD PFOSA: < LOD N-MeFOSAA: 0.110 N-EtFOSAA: < LOD PFHpA: < LOD PFDA: < LOD PFUnDA: < LOD PFDoDA: < LOD	BMI	3–11 years	↑ ΣPFOA—↓ BMI^m^ ↑ n-PFOA—↓ BMI^m^	Not suitable data (no beta estimates, only geometric mean)
Domazet et al., 2016 [108]	EYHS	Odense, Denmark	1997 and 2003	501	PFOA, PFOS	9 yeas 15 years	Medians PFOS: from 20.8 to 44.5 PFOA: from 3.4 to 9.7	BMI WC FM/BFP	9 yeas 15 years	↑ PFOS (at 9 y)—↑ BMI (at 15y) ↑ PFOS (at 9 y)—↑ WC (at 15 y) ↑ PFOS (at 9 y)—↑ FM/BFP (at 15 y)	Overweight risk: PFOS, PFOA (both sexes combined)
Karlsen et al., 2017 [90]	Not specified	Faroe Islands, Denmark	2007–2009	444	PFOS, PFOA, PFHxS, PFNA, PFDA	5 years	Median PFOS: 4.70 PFOA: 2.20 PFHxS: 0.33 PFNA: 1.13 PFDA: 0.34	BMI	5 years	↑ PFNA—↓ BMI ↑ PFDA—↓ BMI ↑ PFOA—↓ BMI ↑ PFOS—↓ BMI	BMI: PFOS, PFOA, PFHxS, PFNA (both sexes combined) Overweight risk: PFOS, PFOA (both sexes combined)
Kim et al., 2014 [109]	Not specified	Dae-gu City, South Korea	2012	120	PFBA, PFPeA, PFHxA, PFHpA, PFOA, PFNA, PFDA, PFUnDA, PFDoDA, PFTrDA, PFTeDA, PFBS, PFHxS, PFHpS, PFOS, PFDS	5—13 years	Mean PFBA: 0.346 PFPeA: 0.497 PFHxA: 0.353 PFHpA: 0.312 PFOA: 5.15 PFNA: 1.72 PFDA: 0.604 PFUnDA: 0.748 PFDoDA: - PFTrDA: 0.306 PFTeDA: - PFBS: 0.105 PFHxS: 1.13 PFHpS: 0.203 PFOS: 6.58 PFDS: -	BMI	5—13 years	↑ PFOA—↓ BMI ↑ PFHxS—↓ BMI ↑ total PFASs—↓ BMI	Not suitable data (no beta estimates, only Pearson's correlation)
Domazet et al., 2020 [110]	Danish sub-study of the EYHS	Odense, Denmark	1997	242	PFOS, PFOA, PFNA, PFDA, PFHxS	9 years	Median PFOS: 42.0—42.9 PFOA: 9.5 PFNA: 0.41—0.44 PFDA: 0.11 PFHxS 0.89—0.95	BMI FM/BFP	9 years	↑ PFNA—↓ BMI ↑ PFDA—↓ BMI ↑ PFNA—↓ FM ↑ PFDA—↓ FM ↑ PFHxS—↓ FM	Not suitable data (no beta estimates with BMI)
Schillemans et al., 2023 [111]	HBM4EU	Europe	2014–2021	1957	PFPeA, PFHxA, PFHpA, PFOA, PFNA, PFDA, PFUnDA, PFDoDA, PFBS, PFHxS, PFHpS, PFOS	12–18 years old	Median PFPeA: 0.10 PFHxA: 0.09 PFHpA: 0.05 PFOA: 0.99 PFNA: 0.32 PFDA: 0.14 PFUnDA: 0.08 PFHxS: 0.04 PFHpS: 0.03 PFOS: 2.1	BMI	12–18 years old	↑ PFPeA—↓ BMI ↑ PFHpA—↓ BMI ↑ PFOA—↓ BMI ↑ PFNA—↓ BMI ↑ PFOS—↓ BMI ↑ PFHxS—↑ BMI^m^ ↑ PFHpS—↑ BMI^m^	BMI: PFOS, PFOA, PFHxS (both sexes combined, only boys, and only girls), PFNA (both sexes combined)
Pinney et al., 2019 [112]	the female puberty cohort of the BCERP	San Francisco Bay Area and Cincinnati (U.S.)	2004–2007	704 (only girls)	PFOA	6–8 years at first enrolments	Median: 6.4	BMI WtHe WtHi	6–8 years at first enrolments Repeated measures up to 18 years	↑ PFOA—↓ BMI ↑ PFOA—↓ WtHe	BMI: PFOA (only girls)
Vrijheid et al., 2020 [64]	HELIX	Europe	2013 – 2016	1301	PFOA, PFNA, PFUnDA, PFHxS, PFOS	6–11 years	Inter Quartile Ranges PFOA: 0.78 PFNA: 0.43 PFUnDA: 0.08 PFHxS: 0.42 PFOS: 2.0	BMI WC FM/BFP	6–11 years	↑ PFNA—↓ BMI ↑ PFOA—↓ BMI ↑ PFUNDA—↓ BMI ↑ PFOA—↓ WC ↑ PFNA—↓ WC ↑ PFOS—↓ WC ↑ PFUNDA—↓ WC ↑ PFNA—↓ skinfold z-score/BFP ↑ PFOS—↓ skinfold z-score/BFP ↑ PFOA—↓ skinfold z-score/BFP	BMI: PFOS, PFOA, PFHxS, PFNA (both sexes combined) Overweight risk: PFOA (both sexes combined) (mean concentrations of PFASs asked to the authors)

The concentration of PFASs varied greatly in prenatal studies: for PFOA, mean or median concentrations ranged from 1 to 45.14 ng/mL; for PFOS, from 1.38 to 33.8; for PFHxS, from 0.33 to 3.6; for PFNA, from 0.4 to 1.77; for PFDA, from less than the limit of detection (LOD) to 1.82. Considering studies measuring PFASs in children, PFOA mean or median concentrations ranged from 0.3 to 117.34 ng/mL; PFOS from 0.04 to 41.5; PFHxS from 0.02 to 27.66; PFNA from 0.03 to 28.78; PFDA from less than the LOD to 0.604.

For prenatal studies, across all studies, most reported no significant associations between considered PFASs and outcomes, as well as some controversial results as both positive and negative associations were reported: for PFOA and BMI, 14 studies reported no associations, 4 a negative association, 8 a positive association, and 1 both a positive and a negative association; for PFOA and WC, 7 studies reported no associations, 2 a negative and 4 a positive; for PFOA and FM/BFP, 9 studies reported no associations, 4 a positive and 1 both; for PFOS and BMI 12 reported no associations, 6 a negative, 7 a positive, and 2 both; for PFOS and WC, 10 reported no associations, 2 a negative, and 1 a positive; for PFOS and FM/BFP, 9 no associations, 2 negative, 1 positive, and 2 both; for PFHxS and BMI, 11 no associations, 2 negative, 4 positive, and 1 both; for PFHxS and WC, 9 no associations, 2 positive, and 1 both; for PFHxS and FM/BFP, 10 no associations and 2 postive; for PFNA and BMI, 14 no associations, 2 negative, and 4 positive; for PFNA and WC, 10 no associations, 2 positive; for PFNA and FM/BFP, 8 no associations, 1 negative, and 3 positive; for PFDA and BMI, 6 no associations, 1 negative and 1 positive; for PFDA and WC, 4 no associations; for PFDA and FM/BFP, 2 no associations, 1 negative, and 1 positive.

For studies measuring PFASs in children, even though there were still several works reporting no significant associations, there were also several ones reporting a negative associations: in particular, for PFOA and BMI, 9 studies reported no associations, 10 a negative association, and 1 a positive association; for PFOA and WC, 5 studies reported no associations, and 2 a negative associations; for PFOA and FM/BFP, 7 studies reported no associations, 2 a negative; for PFOS and BMI 11 reported no associations, 7 a negative, and 1 a; for PFOS and WC, 11 reported no associations, 1 a negative, and 1 a positive; for PFOS and FM/BFP, 6 no associations, and 4 negative; for PFHxS and BMI, 9 no associations, 4 negative, 2 positive; for PFHxS and WC, just 4 no associations; for PFHxS and FM/BFP, 4 no associations and 3 negative; for PFNA and BMI, 10 no associations, and 5 negative; for PFNA and WC, 2 no associations, and 1 negative; for PFNA and FM/BFP, 4 no associations, and 3 negative; for PFDA and BMI, 7 no associations, and 3 negative; for PFDA and FM/BFP, 2 negative, and 1 positive.

Each study was evaluated for the quality of reporting, considering the guidelines of the STROBE-ME [46]. Most studies were judged of high quality: information needed were clearly reported, including selection criteria, possible follow-up, sample-size, collection of biological samples, methods and tools implemented for data collection and outcome measurements, demographic and clinical characteristics of subjects. Furthermore, most studies took into consideration several potential confounding factors in the statistical analysis. No particular bias was noted in the selection of study individuals. Even if it was not clearly stated in most studies, it is reasonable to assume that analytical measurements were blinded, in order to counter this possible bias. Considering the score of reporting, all the articles had score equal to or higher than 22/28, with a mean of 27.01 (Table S03), thus all of them were judged of high quality.

### Meta-analyses

After collecting the estimates from the studies included in this systematic review, considering the requirement of having suitable estimates from at least 3 studies, we calculated the pooled estimates for the overall association between prenatal concentrations of PFOA, PFOS, PFHxS, PFNA, PFDA, PFUA, PFDoA, PFBS and BMI; between prenatal concentrations of PFOA, PFOS, PFHxS, PFNA and risk of overweight; and between prenatal concentrations of PFOA, PFOS, PFHxS, PFNA, PFDA, PFUA and WC. Furthermore, suitable data were available for calculating pooled estimates of the association between childhood concentrations of PFOA, PFOS, PFHxS, PFNA and childhood BMI; and between childhood concentrations of PFOA, PFOS and risk of overweight. For all of the above, we were able to pool the estimates for both sexes combined, for boys exclusively, and for girls exclusively, with the exception of childhood PFNA and childhood BMI, and for all the data related to risk of overweight, for which only the effect of both sexes combined were calculated. When more studies describing the same cohort were reported, only one was included in the meta-analysis, in particular: for the HOME study, data from Liu et al. [81] were chosen for completeness of information, while associations with overweight risks were taken from Braun et al. [84]; for the Danish National Birth Cohort, the paper from Andersen et al. (2010) [89] was considered for the data of both sexes combined while the sex-specific data were taken from Andersen et al., (2013) [82]; data from the HELIX project considering childhood exposure, were taken from Vrijheid et al., 2020 [64] and not from Papadopoulou et al., 2021 [65] as the population was greater in the first. Moreover, authors of Gyllenhammar et al. and Vrijheid et al. were contacted to obtain suitable numeric data for inclusion [64, 75]. Overall, all the information about the data included in the meta-analyses from each study included in the systematic review is reported in the last column of Table 1 and Table 2.

Altogether, out of the 32 studies included in this systematic review evaluating prenatal exposure to PFASs, 23 could be included for the meta-analyses, in particular: data from 17, 12, and 13 studies were suitable for the meta-analysis evaluating the associations between prenatal PFOA and BMI for both sexes combined, boys, and girls, respectively; similarly, 17, 12, and 13 studies were included for PFOS; 11, 9, and 10 studies for PFHxS; 11, 9, and 10 studies for PFNA; 6, 6, and 6 studies for PFDA; 5, 5, and 5 studies for PFUA; 3, 3, and 3 studies for PFDoA; 3, 3, and 3 studies for PFBS. Considering the association between prenatal exposure to PFASs and risk of overweight, data from 8 studies were included for the meta-analysis for PFOA for both sexes combined, 8 studies for PFOS, 6 studies for PFHxS, 6 studies for PFNA. Considering the association between prenatal exposure to PFASs and childhood WC, data from 8, 8, and 9 were included for the meta-analyses for PFOA for both sexes combined, boys, and girls, respectively; 8, 8, and 9 for PFOS; 8, 7, and 8 for PFHxS; 8, 7, and 8 for PFNA; 4, 4, and 4 for PFDA; 3, 3, and 3 for PFUA (Table S04).

Out of the 23 studies included in this systematic review evaluating childhood exposure to PFASs, 11 were included in the meta-analyses, in particular: considering the association with childhood BMI, data from 8, 3, and 4 studies were included for the meta-analyses for PFOA for both sexes combined, boys, and girls, respectively; 8, 3, and 3 for PFOS; 6, 3, and 3 for PFHxS; and 5 for PFNA (the latter only both sexes combined, as not enough suitable data for the analyses stratified by sexes). Finally, considering the association between childhood exposure to PFASs and risk of overweight, data from 4 studies were suitable for PFOA and 3 for PFOS, in both cases only for both sexes combined (Table S04).

Overall, considering all the 30 studies included in the meta-analyses, the effect estimates were calculated with different log-transformations of PFASs: in particular, 8 were reported as unit (non log-transformed), 12 as natural logarithm, 5 as base-10 logarithm and 5 as base-2 logarithm. Moreover, one study also natural log-transformed the outcome variable and reported the result as percentage change in the outcome. Among all the 30 studies, 19 calculated the estimates out of continuous data, 6 reported mean changes for interquartile range increase, 2 reported differences between terciles, 1 reported differences between quartiles, 1 reported mean differences for each standard deviation increase, and 1 reported percentage change in the outcome. Among the 25 studies considering BMI, 20 calculated the beta estimate from BMI z-scores or BMI SDS, while 5 from BMI as kg/m^2^. Among the 10 studies considering WC, 5 calculated the beta estimate from WC z-scores or WC SDS, while 5 from WC as cm. Among the 10 studies considering the risk of overweight, 5 reported the OR, 4 the RR, and 1 reported a linear association with a categorical variable “overweight—yes” (so was not modified considering steps a) nor b) reported in the “2.3.3” section). Thus, the data conversions described in the section “2.3.3” were useful to properly include all these studies in the meta-analyses ensuring that the effects estimates were comparable each other (Table S05).

**Fig. 2:**
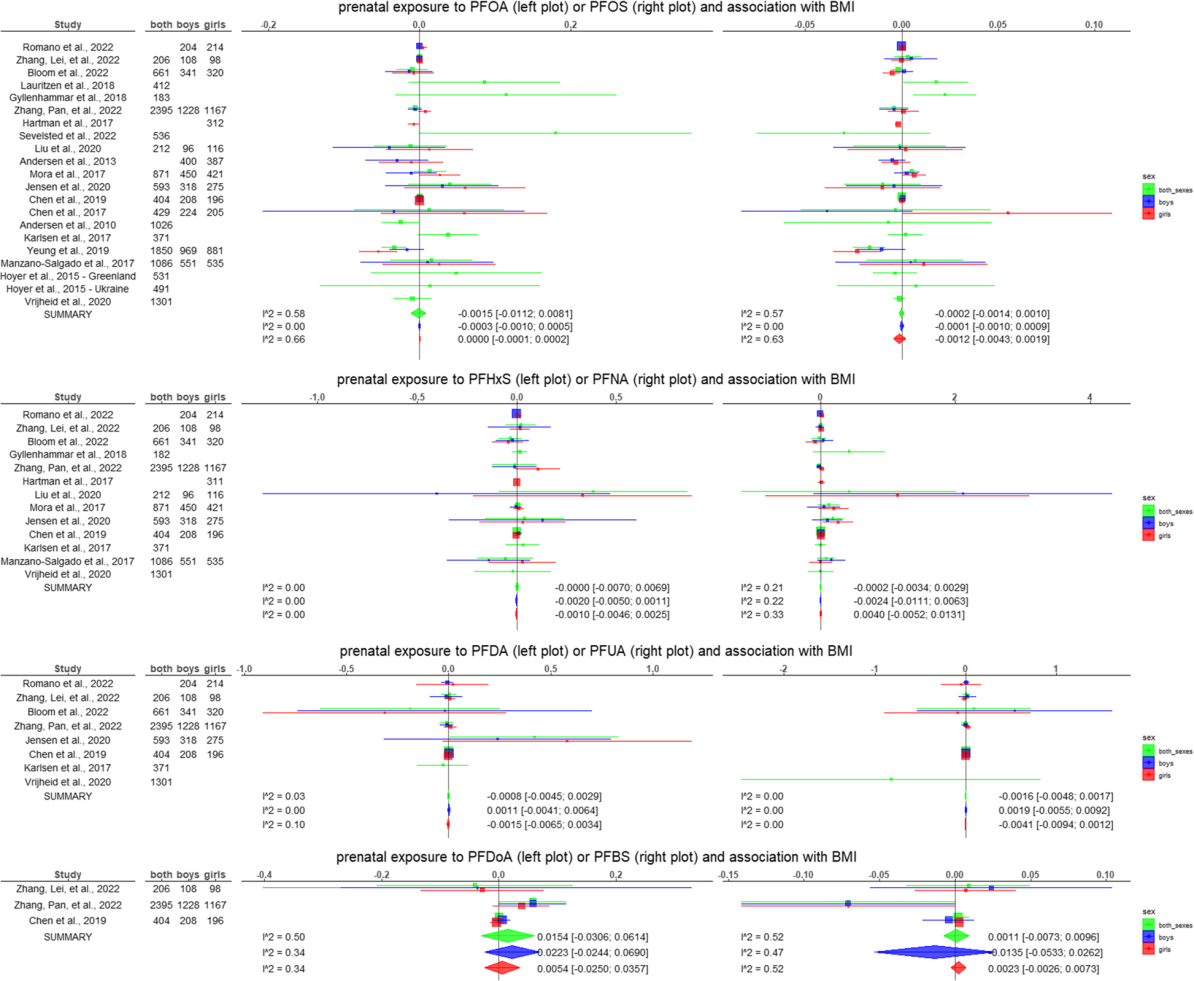

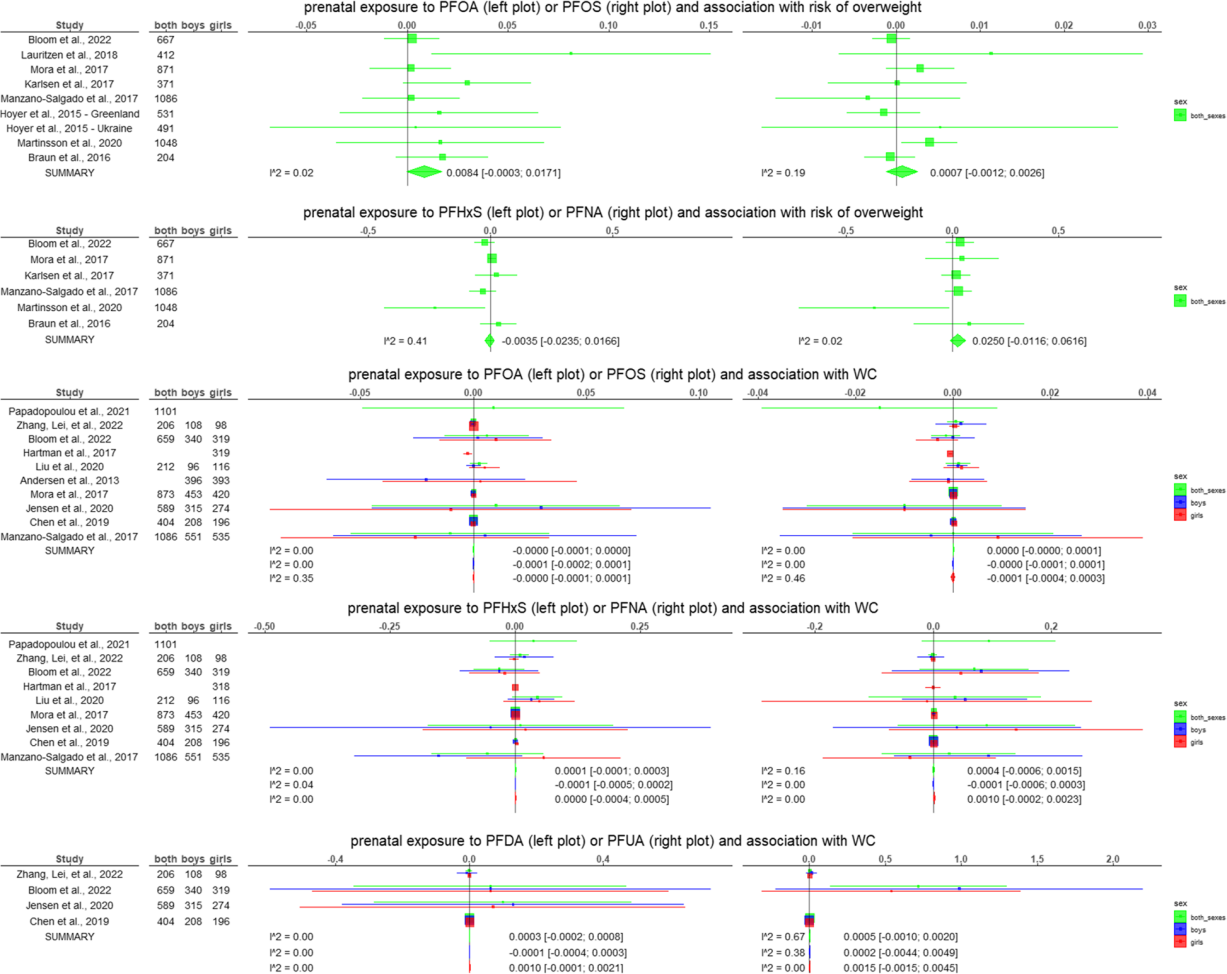

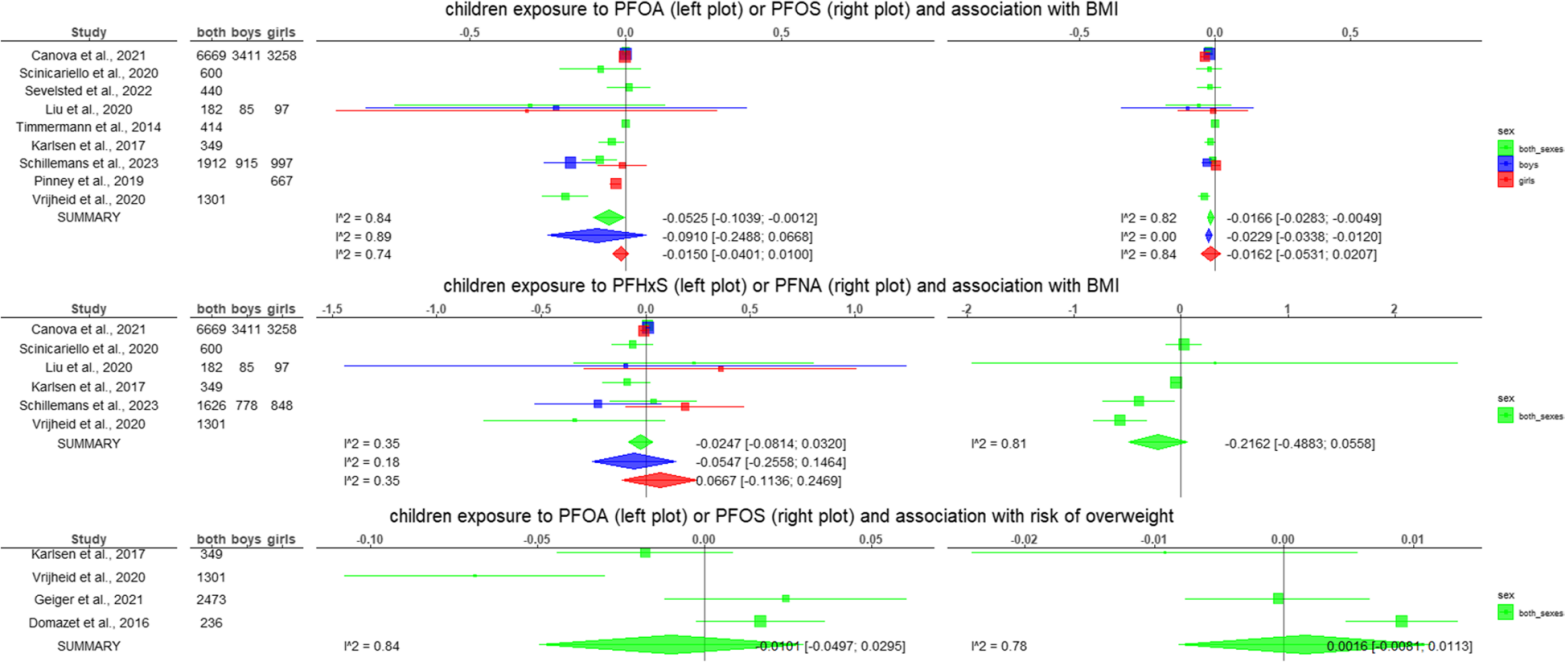
Superimposed forest plots of the meta-analyses performed with the inverse variance method considering separately both sexes, boys, and girls. The beta estimates represent the association between the increase of 1 z-score (of BMI or WC) per 1 ng/mL increase in the PFAS. For risk of overweight, the estimates represent transformed standardized mean differences (see section " 2.3.3 Data conversion of the effect estimates"). The squares are proportional to the weight assigned to each study in the random effects models, while horizontal lines represent the 95% confidence intervals reported. The diamonds at the bottom represent the pooled estimated of the random effects models (with the 95% confidence interval). The I^2^ is also reported as a measure of heterogeneity

The results of all the meta-analyses performed are summarised in Table 3 and graphical representations are given in Fig. 2. The forest plots and funnel plots for each meta-analysis are reported in the supplementary material (Supplementary Figures).

**Table 3:** Results of the meta-analyses performed on a subset of the studies included in the systematic review. For each combination of exposure and outcome, and for each sex category (boys and girls combined, only boys, and only girls) a meta-analysis was performed only if suitable information was reported from at least 3 studies. In this table, the pooled estimated of the random effects models (with the 95% confidence interval) from the generic invariance method, the I^2^ of the heterogeneity test, the *p*-value of the Egger’s test, and the pooled estimated of the random effects models (with the 95% confidence interval) by weighting per sample size are reported. The complete forest and funnel plots are reported in the supplementary figures

Conditions assessed	Studies included	Random effects model [95% CI] Generic invariance method	Heterogeneity (I^2^)	Egger’s test *p*-value	Random effects model [95% CI] Weighted per sample size
Prenatal PFOA and BMI					
Both sexes	[64, 69, 70, 74–76, 79, 81, 83, 86–92, 94]	-0.0015 [-0.0112; 0.0081]	0.58	0.743	0.0099 [-0.0031; 0.0228]
Boys	[68–70, 76, 81–83, 86–88, 91, 92]	-0.0003 [-0.0010; 0.0005]	0.00	0.027	-0.0081 [-0.0229; 0.0068]
Girls	[68–70, 76, 78, 81–83, 86–88, 91, 92]	0.0000 [-0.0001; 0.0002]	0.66	0.814	0.0015 [-0.0104; 0.0134]
Prenatal PFOS and BMI					
Both sexes	[64, 69, 70, 74–76, 79, 81, 83, 86–92, 94]	-0.0002 [-0.0014; 0.0010]	0.57	0.973	-0.0035 [-0.0096; 0.0025]
Boys	[68–70, 76, 81–83, 86–88, 91, 92]	-0.0001 [-0.0010; 0.0009]	0.00	0.299	-0.0044 [-0.0103; 0.0014]
Girls	[68–70, 76, 78, 81–83, 86–88, 91, 92]	-0.0012 [-0.0043; 0.0019]	0.63	0.588	-0.0009 [-0.0064; 0.0045]
Prenatal PFHxS and BMI					
Both sexes	[64, 69, 70, 75, 76, 81, 83, 86, 87, 90, 92]	0.0000 [-0.007; 0.0069]	0.00	0.389	-0.0008 [-0.0524; 0.0509]
Boys	[68–70, 76, 81, 83, 86, 87, 92]	-0.0020 [-0.0050; 0.0011]	0.00	0.601	-0.0268 [-0.0983; 0.0447]
Girls	[68–70, 76, 78, 81, 83, 86, 87, 92]	-0.0010 [-0.0046; 0.0025]	0.00	0.080	0.0486 [-0.0013; 0.0986]
Prenatal PFNA and BMI					
Both sexes	[64, 69, 70, 75, 76, 81, 83, 86, 87, 90, 92]	-0.0002 [-0.0034; 0.0029]	0.21	0.112	0.0481 [-0.013; 0.1091]
Boys	[68–70, 76, 81, 83, 86, 87, 92]	-0.0024 [-0.0111; 0.0063]	0.22	0.232	**0.0931 [0.0108; 0.1753]**
Girls	[68–70, 76, 78, 81, 83, 86, 87, 92]	0.0040 [-0.0052; 0.0131]	0.33	0.040	**0.0800 [0.0036; 0.1564]**
Prenatal PFDA and BMI					
Both sexes	[69, 70, 76, 86, 87, 90]	-0.0008 [-0.0045; 0.0029]	0.03	0.855	0.0201 [-0.0644; 0.1046]
Boys	[68–70, 76, 86, 87]	0.0011 [-0.0041; 0.0064]	0.00	0.753	0.0235 [-0.1035; 0.1504]
Girls	[68–70, 76, 86, 87]	-0.0015 [-0.0065; 0.0034]	0.1	0.362	0.0329 [-0.0815; 0.1473]
Prenatal PFUA and BMI					
Both sexes	[64, 69, 70, 76, 87]	-0.0016 [-0.0048; 0.0017]	0.00	0.618	-0.2062 [-0.6457; 0.2333]
Boys	[68–70, 76, 87]	0.0019 [-0.0055; 0.0092]	0.00	0.343	0.0860 [-0.0910; 0.2630]
Girls	[68–70, 76, 87]	-0.0041 [-0.0094; 0.0012]	0.00	0.994	-0.0082 [-0.1418; 0.1254]
Prenatal PFDoA and BMI					
Both sexes	[69, 76, 87]	0.0154 [-0.0306; 0.0614]	0.50	0.680	0.0433 [-0.0221; 0.1088]
Boys	[69, 76, 87]	0.0223 [-0.0244; 0.0690]	0.34	0.677	0.0442 [-0.0187; 0.1070]
Girls	[69, 76, 87]	0.0054 [-0.0250; 0.0357]	0.34	0.732	0.0284 [-0.0188; 0.0757]
Prenatal PFBS and BMI					
Both sexes	[69, 76, 87]	0.0011 [-0.0073; 0.0096]	0.52	0.576	-0.0555 [-0.1196; 0.0086]
Boys	[69, 76, 87]	-0.0135 [-0.0533; 0.0262]	0.47	0.757	-0.0552 [-0.1256; 0.0153]
Girls	[69, 76, 87]	0.0023 [-0.0026; 0.0073]	0.52	0.561	-0.0557 [-0.1178; 0.0065]
Prenatal PFOA and risk of overweight					
Both sexes	[70, 71, 74, 83, 84, 90, 92, 94]	0.0084 [-0.0003; 0.0171]	0.02	0.063	0.0142 [-0.0006; 0.0289]
Prenatal PFOS and risk of overweight					
Both sexes	[70, 71, 74, 83, 84, 90, 92, 94]	0.0007 [-0.0012; 0.0026]	0.19	0.504	0.0016 [-0.0018; 0.0050]
Prenatal PFHxS and risk of overweight					
Both sexes	[70, 71, 83, 84, 90, 92]	-0.0035 [-0.0235; 0.0166]	0.41	0.312	**-0.0627 [-0.1201; -0.0052]**
Prenatal PFNA and risk of overweight					
Both sexes	[70, 71, 83, 84, 90, 92]	0.0250 [-0.0116; 0.0616]	0.02	0.338	-0.0633 [-0.1611; 0.0344]
Prenatal PFOA and WC					
Both sexes	[65, 69, 70, 81, 83, 86, 87, 92]	0.0000 [-0.0001; 0.0000]	0.00	0.168	0.0017 [-0.0152; 0.0186]
Boys	[69, 70, 81–83, 86, 87, 92]	-0.0001 [-0.0002; 0.0001]	0.00	0.467	0.0019 [-0.0177; 0.0214]
Girls	[69, 70, 78, 81–83, 86, 87, 92]	0.0000 [-0.0001; 0.0001]	0.35	0.884	-0.0046 [-0.0207; 0.0114]
Prenatal PFOS and WC					
Both sexes	[65, 69, 70, 81, 83, 86, 87, 92]	0.0000 [0.0000; 0.0001]	0.00	0.405	-0.0045 [-0.0117; 0.0027]
Boys	[69, 70, 81–83, 86, 87, 92]	0.0000 [-0.0001; 0.0001]	0.00	0.987	-0.0024 [-0.0101; 0.0054]
Girls	[69, 70, 78, 81–83, 86, 87, 92]	-0.0001 [-0.0004; 0.0003]	0.46	0.500	0.0003 [-0.0063; 0.0070]
Prenatal PFHxS and WC					
Both sexes	[65, 69, 70, 81, 83, 86, 87, 92]	0.0001 [-0.0001; 0.0003]	0.00	0.589	-0.0050 [-0.0426; 0.0326]
Boys	[69, 70, 81, 83, 86, 87, 92]	-0.0001 [-0.0005; 0.0002]	0.04	0.661	-0.0516 [-0.1332; 0.0300]
Girls	[69, 70, 78, 81, 83, 86, 87, 92]	0.0000 [-0.0004; 0.0005]	0.00	0.620	0.0151 [-0.0300; 0.0601]
Prenatal PFNA and WC					
Both sexes	[65, 69, 70, 81, 83, 86, 87, 92]	0.0004 [-0.0006; 0.0015]	0.16	0.019	**0.0467 [0.0063; 0.0872]**
Boys	[69, 70, 81, 83, 86, 87, 92]	-0.0001 [-0.0006; 0.0003]	0.00	0.042	0.0465 [-0.0123; 0.1053]
Girls	[69, 70, 78, 81, 83, 86, 87, 92]	0.0010 [-0.0002; 0.0023]	0.00	0.592	0.0137 [-0.0353; 0.0628]
Prenatal PFDA and WC					
Both sexes	[69, 70, 86, 87]	0.0003 [-0.0002; 0.0008]	0.00	0.607	0.0538 [-0.1353; 0.2429]
Boys	[69, 70, 86, 87]	-0.0001 [-0.0004; 0.0003]	0.00	0.833	0.0632 [-0.2203; 0.3468]
Girls	[69, 70, 86, 87]	0.0010 [-0.0001; 0.0021]	0.00	0.728	0.0444 [-0.2168; 0.3056]
Prenatal PFUA and WC					
Both sexes	[69, 70, 87]	0.0005 [-0.0010; 0.0020]	0.67	0.345	**0.3729 [0.0703; 0.6755]**
Boys	[69, 70, 87]	0.0002 [-0.0044; 0.0049]	0.38	0.195	0.5132 [-0.1139; 1.1404]
Girls	[69, 70, 87]	0.0015 [-0.0015; 0.0045]	0.00	0.242	0.2805 [-0.1624; 0.7234]
Childhood PFOA and BMI					
Both sexes	[64, 79, 81, 90, 98, 104, 105, 111]	**-0.0525 [-0.1039; -0.0012]**	0.84	0.023	**-0.0445 [-0.0604; -0.0286]**
Boys	[81, 98, 111]	-0.0910 [-0.2488; 0.0668]	0.89	0.394	-0.0410 [-0.2285; 0.1464]
Girls	[81, 98, 111, 112]	-0.0150 [-0.0401; 0.0100]	0.74	0.256	-0.0134 [-0.0456; 0.0187]
Childhood PFOS and BMI					
Both sexes	[64, 79, 81, 90, 98, 104, 105, 111]	**-0.0166 [-0.0283; -0.0049]**	0.82	0.011	**-0.0226 [-0.0412; -0.0039]**
Boys	[81, 98, 111]	**-0.0229 [-0.0338; -0.0120]**	0.00	0.359	**-0.0235 [-0.0354; -0.0116]**
Girls	[81, 98, 111]	-0.0162 [-0.0531; 0.0207]	0.84	0.972	-0.0278 [-0.0719; 0.0164]
Childhood PFHxS and BMI					
Both sexes	[64, 81, 90, 98, 104, 111]	-0.0247 [-0.0814; 0.0320]	0.35	0.260	-0.0334 [-0.1157; 0.0489]
Boys	[81, 98, 111]	-0.0547 [-0.2558; 0.1464]	0.18	0.444	-0.0331 [-0.1620; 0.0957]
Girls	[81, 98, 111]	0.0667 [-0.1136; 0.2469]	0.35	0.079	0.0399 [-0.1249; 0.2047]
Childhood PFNA and BMI					
Both sexes	[64, 81, 90, 104, 111]	-0.2162 [-0.4883; 0.0558]	0.81	0.383	**-0.3279 [-0.6121; -0.0438]**
Childhood PFOA and risk of overweight					
Both sexes	[64, 90, 96, 108]	-0.0101 [-0.0497; 0.0295]	0.84	0.413	-0.0074 [-0.0554; 0.0407]
Childhood PFOS and risk of overweight					
Both sexes	[90, 96, 108]	0.0016 [-0.0081; 0.0113]	0.78	0.231	-0.0007 [-0.0137; 0.0123]

Overall, when considering prenatal exposure to PFASs and BMI, risk of overweight, and WC, the results from the meta-analyses performed with the inverse variance method were all non-significant, while with the analyses weighted per sample size, a positive association was observed for prenatal PFNA and BMI in boys (0.0931; 95% CI: 0.0108, 0.1753) and in girls (0.08; 95% CI: 0.0036, 0.1564), for prenatal PFNA and WC in both sexes combined (0.0467; 95% CI: 0.0063, 0.0872), and for prenatal PFUA and WC in both sexes combined (0.3729; 95% CI: 0.0703, 0.6755) (Table 3), even though the latter was not confirmed in any of the sensitivity analyses performed by removing one study per time (Table S06). Conversely, a negative association was found between PFHxS and the risk of overweight (-0.0627; 95% CI: -0.1201, -0.0052) (Table 3). Heterogeneity across studies was high for some meta-analyses, in particular for prenatal PFOA, PFOS and BMI in both sexes combined (I^2^ = 0.58, 0.57) and in girls (I^2^ = 0.66, 0.63) (Table 3). In the sensitivity analyses performed by removing one study per time, some more significant positive associations were noted in the meta-analyses weighted per sample sizes for prenatal PFOA and BMI (both sexes combined), and for prenatal PFOA and risk of overweight (both sexes combined), the latter also for analyses performed with the generic invariance method. In a single analysis removing a study, a negative association between prenatal PFOS and BMI was found in boys and, similarly, a positive association between prenatal PFNA and BMI in both sexes combined was found (Table S06).

Considering the childhood exposure to PFASs and measurements of BMI, significant negative associations were found both in the meta-analyses performed with the inverse variance method and in those weighted per sample size for childhood PFOA and BMI both sexes (-0.0525, 95% CI: -0.1039, -0.0012 and -0.0445; 95% CI: -0.0604, -0.0286), PFOS and BMI both sexes (-0.0166; 95% CI: -0.0283, -0.0049 and -0.0226; 95% CI: -0.0412, -0.0039) and only boys (-0.0229; 95% CI: -0.0338, -0.0120 and -0.0235; 95% CI:-0.0354, -0.0116); a significant associations was found for childhood PFNA and BMI both sexes in the analyses weighted per samples sizes (-0.3279; 95% CI: -0.6121, -0.0438) (Table 3). The heterogeneity, though, was high in most of those cases (I^2^ = 0.84 for childhood PFOA and BMI both sexes, 0.82 for PFOS and BMI both sexes, 0.81 for PFNA and BMI both sexes), and the Egger’s test *p*-value evaluating was below 0.05 for PFOA and PFOS (0.023 and 0.011, respectively) (Table 3). Sensitivity analyses confirmed the negative associations in particularly for PFOS and found other negative associations also in sex stratified analyses considering PFOS and PFOA. Interestingly, in an inverse variance method sensitivity analysis for childhood PFHxS and BMI in boys, excluding one study, a significant positive association was found (Table S06).

In the meta-analyses performed stratifying by age (Table 4), a significant positive association was found for prenatal PFOA and BMI measured in children of both sexes who were more than 3 years, but only in analyses weighted per sample size (0.0275; 95% CI: 0.0059, 0.0491), although the Egger’s test *p*-value was 0.047. Conversely, an inverse association between prenatal PFOS and BMI measured in children of both sexes who were 3 or less years was found only in the invariance method analyses (-0.0101; 95% CI: -0.0189, -0.0013). Significant positive associations were also found also, only with the analyses weighted per sample size, between prenatal PFNA and boys who were more than 3 years (0.1851; 95% CI: 0.028, 0.3421). Significant negative associations were also found in the analyses weighted per sample size between prenatal PFHxS and risk of overweight in children who were more than 3 years and positive associations were found between prenatal PFUA and WC; significant negative associations, confirmed both with inverse variance and weighted per samples size analyses were also found between childhood PFOA and BMI in children of both sexes who were more than 3 years, and between childhood PFOS and BMI in children who were more than 3 years in both sexes and in boys only; finally, a negative association, exclusively in analyses weighted per sample size, was found for childhood PFNA and BMI in children of both sexes who were more than 3 years: all of these results were the same of the non-age-stratified analyses as all the included studies considered children who were more than 3 years.

**Table 4:** Results of the meta-analyses performed on a subset of the studies included in the systematic review stratifying separately studies that measured the outcomes in children who were 3 or less years from studies that considered children who were more than 3 years. For each combination of exposure and outcome, and for each sex category (boys and girls combined, only boys, and only girls) a meta-analysis was performed only if suitable information was reported from at least 3 studies. In this table, the pooled estimated of the random effects models (with the 95% confidence interval) from the generic invariance method, the I^2^ of the heterogeneity test, the *p*-value of the Egger’s test, and the pooled estimated of the random effects models (with the 95% confidence interval) by weighting per sample size are reported. The complete forest and funnel plots are reported in the supplementary figures

Conditions assessed	Studies included	Random effects model [95% CI] Generic invariance method	Heterogeneity (I^2^)	Egger’s test *p*-value	Random effects model [95% CI] Weighted per sample size
Prenatal PFOA and BMI in both sexes 0–3 years	[76, 86, 89, 91]	-0.0140 [-0.0351; 0.0070]	0.77	0.910	-0.0133 [-0.0343; 0.0078]
4–18 years	[64, 69, 70, 74, 75, 79, 81, 83, 87, 88, 90, 92, 94]	0.0000 [-0.0008; 0.0009]	0.24	0.047	**0.0275 [0.0059; 0.0491]**
Prenatal PFOA and BMI in boys 0–3 years	[68, 76, 86, 91]	-0.0022 [-0.0070; 0.0026]	0.19	0.533	-0.0051 [-0.0182; 0.0079]
4–18 years	[69, 70, 81–83, 87, 88, 92]	0.0000 [-0.0009; 0.0008]	0.00	0.039	-0.0115 [-0.0398; 0.0168]
Prenatal PFOA and BMI in girls 0–3 years	[68, 76, 86, 91]	-0.0036 [-0.0413; 0.0341]	0.87	0.673	-0.0087 [-0.0313; 0.0139]
4–18 years	[69, 70, 78, 81–83, 87, 88, 92]	0.0000 [-0.0001; 0.0002]	0.23	0.672	0.0115 [-0.0084; 0.0313]
Prenatal PFOS and BMI in both sexes 0–3 years	[76, 86, 89, 91]	**-0.0101 [-0.0189; -0.0013]**	0.38	0.965	-0.0092 [-0.0210; 0.0026]
4–18 years	[64, 69, 70, 74, 75, 79, 81, 83, 87, 88, 90, 92, 94]	0.0002 [-0.0009; 0.0014]	0.42	0.251	0.0008 [-0.0056; 0.0072]
Prenatal PFOS and BMI in boys 0–3 years	[68, 76, 86, 91]	-0.0024 [-0.0071; 0.0024]	0.21	0.123	-0.0060 [-0.0128; 0.0007]
4–18 years	[69, 70, 81–83, 87, 88, 92]	0.0002 [-0.0029; 0.0033]	0.00	0.538	-0.0026 [-0.0128; 0.0077]
Prenatal PFOS and BMI in girls 0–3 years	[68, 76, 86, 91]	-0.0065 [-0.0186; 0.0056]	0.78	0.356	-0.0086 [-0.0198; 0.0026]
4–18 years	[69, 70, 78, 81–83, 87, 88, 92]	-0.0006 [-0.0033; 0.0021]	0.56	0.883	0.0065 [-0.0019; 0.0150]
Prenatal PFHxS and BMI in both sexes 4–18 years	[64, 69, 70, 75, 81, 83, 87, 90, 92]	0.0000 [-0.0071; 0.0071]	0.00	0.435	-0.0009 [-0.0600; 0.0582]
Prenatal PFHxS and BMI in boys 0–3 years	[68, 76, 86]	-0.0023 [-0.0054; 0.0009]	0.00	0.659	0.0159 [-0.0998; 0.1317]
4–18 years	[69, 70, 81, 83, 87, 92]	0.0040 [-0.0108; 0.0188]	0.00	0.051	-0.0694 [-0.1534; 0.0146]
Prenatal PFHxS and BMI in girls 0–3 years	[68, 76, 86]	0.0343 [-0.0360; 0.1046]	0.33	0.463	0.0806 [-0.0197; 0.181]
4–18 years	[69, 70, 78, 81, 83, 87, 92]	-0.0014 [-0.0050; 0.0021]	0.00	0.387	0.0221 [-0.0348; 0.0790]
Prenatal PFNA and BMI in both sexes 4–18 years	[64, 69, 70, 75, 81, 83, 87, 90, 92]	-0.0001 [-0.0033; 0.0030]	0.00	0.072	0.0656 [-0.0252; 0.1564]
Prenatal PFNA and BMI in boys 0–3 years	[68, 76, 86]	-0.0083 [-0.0183; 0.0016]	0.00	0.831	0.0009 [-0.0495; 0.0513]
4–18 years	[69, 70, 81, 83, 87, 92]	0.0022 [-0.0052; 0.0097]	0.17	0.120	**0.1851 [0.0280; 0.3421]**
Prenatal PFNA and BMI in girls 0–3 years	[68, 76, 86]	0.0216 [-0.0031; 0.0463]	0.52	0.119	0.0588 [-0.0023; 0.1198]
4–18 years	[69, 70, 78, 81, 83, 87, 92]	-0.0002 [-0.0027; 0.0023]	0.05	0.290	0.0976 [-0.0355; 0.2307]
Prenatal PFDA and BMI in both sexes 4–18 years	[69, 70, 87, 90]	-0.0007 [-0.003; 0.0015]	0.00	0.001	-0.0816 [-0.2605; 0.0973]
Prenatal PFDA and BMI in boys 0–3 years	[68, 76, 86]	-0.0069 [-0.0286; 0.0147]	0.00	0.196	0.0365 [-0.0673; 0.1402]
4–18 years	[69, 70, 87]	0.0016 [-0.0038; 0.0070]	0.00	0.410	-0.0111 [-0.3852; 0.3630]
Prenatal PFDA and BMI in girls 0–3 years	[68, 76, 86]	0.0111 [-0.0205; 0.0427]	0.41	0.407	0.1055 [-0.0437; 0.2546]
4–18 years	[69, 70, 87]	-0.0017 [-0.0077; 0.0043]	0.00	0.829	-0.1628 [-0.4729; 0.1474]
Prenatal PFUA and BMI in both sexes 4–18 years	[64, 69, 70, 87]	-0.0015 [-0.0048; 0.0017]	0.00	0.749	-0.3931 [-1.2412; 0.4549]
Prenatal PFUA and BMI in boys 4–18 years	[69, 70, 87]	0.0021 [-0.0055; 0.0098]	0.00	0.263	0.2827 [-0.2755; 0.8409]
Prenatal PFUA and BMI in girls 4–18 years	[69, 70, 87]	-0.0045 [-0.0098; 0.0008]	0.00	0.325	-0.0501 [-0.4704; 0.3701]
Prenatal PFOA and risk of overweight 4–18 years	[70, 71, 74, 83, 84, 90, 92, 94]	0.0084 [-0.0003; 0.0171]	0.02	0.063	0.0142 [-0.0006; 0.0289]
Prenatal PFOS and risk of overweight 4–18 years	[70, 71, 74, 83, 84, 90, 92, 94]	0.0007 [-0.0012; 0.0026]	0.19	0.504	0.0016 [-0.0018; 0.0050]
Prenatal PFHxS and risk of overweight 4–18 years	[70, 71, 83, 84, 90, 92]	-0.0035 [-0.0235; 0.0166]	0.41	0.312	**-0.0627 [-0.1201; -0.0052]**
Prenatal PFNA and risk of overweight 4–18 years	[70, 71, 83, 84, 90, 92]	0.025 [-0.0116; 0.0616]	0.02	0.338	-0.0633 [-0.1611; 0.0344]
Prenatal PFOA and WC in both sexes 4–18 years	[65, 69, 70, 81, 83, 87, 92]	0.0000 [-0.0001; 0.0000]	0.00	0.232	0.0007 [-0.0171; 0.0184]
Prenatal PFOA and WC in boys 4–18 years	[69, 70, 81–83, 87, 92]	-0.0001 [-0.0002; 0.0001]	0.00	0.199	-0.0023 [-0.0218; 0.0173]
Prenatal PFOA and WC in girls 4–18 years	[69, 70, 78, 81–83, 87, 92]	0.0000 [-0.0001; 0.0001]	0.43	0.939	-0.0040 [-0.0194; 0.0114]
Prenatal PFOS and WC in both sexes 4–18 years	[65, 69, 70, 81, 83, 87, 92]	0.0000 [0.0000; 0.0001]	0.00	0.675	-0.0038 [-0.0115; 0.0039]
Prenatal PFOS and WC in boys 4–18 years	[69, 70, 81–83, 87, 92]	0.0000 [-0.0001; 0.0001]	0.00	0.492	-0.0012 [-0.0093; 0.0068]
Prenatal PFOS and WC in girls 4–18 years	[69, 70, 78, 81–83, 87, 92]	-0.0001 [-0.0004; 0.0003]	0.51	0.631	0.0015 [-0.0053; 0.0083]
Prenatal PFHxS and WC in both sexes 4–18 years	[65, 69, 70, 81, 83, 87, 92]	0.0003 [-0.0014; 0.0020]	0.13	0.621	-0.0069 [-0.0420; 0.0281]
Prenatal PFHxS and WC in boys 4–18 years	[69, 70, 81, 83, 87, 92]	-0.0001 [-0.0041; 0.0039]	0.19	0.726	-0.0518 [-0.1069; 0.0032]
Prenatal PFHxS and WC in girls 4–18 years	[69, 70, 78, 81, 83, 87, 92]	0.0000 [-0.0004; 0.0005]	0.06	0.675	0.0144 [-0.0285; 0.0572]
Prenatal PFNA and WC in both sexes 4–18 years	[65, 69, 70, 81, 83, 87, 92]	0.0004 [-0.0006; 0.0015]	0.14	0.051	0.0411 [-0.0002; 0.0824]
Prenatal PFNA and WC in boys 4–18 years	[69, 70, 81, 83, 87, 92]	-0.0001 [-0.0006; 0.0003]	0.00	0.070	0.0477 [-0.0105; 0.1059]
Prenatal PFNA and WC in girls 4–18 years	[69, 70, 78, 81, 83, 87, 92]	0.0010 [-0.0002; 0.0023]	0.00	0.779	-0.0035 [-0.0509; 0.0438]
Prenatal PFDA and WC in both sexes 4–18 years	[69, 70, 87]	0.0003 [-0.0002; 0.0008]	0.00	0.956	0.0324 [-0.1791; 0.2438]
Prenatal PFDA and WC in boys 4–18 years	[69, 70, 87]	-0.0001 [-0.0004; 0.0003]	0.00	0.738	0.0312 [-0.3097; 0.3721]
Prenatal PFDA and WC in girls 4–18 years	[69, 70, 87]	0.0010 [-0.0001; 0.0021]	0.00	0.963	0.0329 [-0.2441; 0.3100]
Prenatal PFUA and WC in both sexes 4–18 years	[69, 70, 87]	0.0005 [-0.0010; 0.0020]	0.67	0.345	**0.3729 [0.0703; 0.6755]**
Prenatal PFUA and WC in boys 4–18 years	[69, 70, 87]	0.0002 [-0.0044; 0.0049]	0.38	0.195	0.5132 [-0.1139; 1.1404]
Prenatal PFUA and WC in girls 4–18 years	[69, 70, 87]	0.0015 [-0.0015; 0.0045]	0.00	0.242	0.2805 [-0.1624; 0.7234]
Childhood PFOA and BMI in both sexes 4–18 years	[64, 79, 81, 90, 98, 104, 105, 111]	**-0.0525 [-0.1039; -0.0012]**	0.84	0.023	**-0.0445 [-0.0604; -0.0286]**
Childhood PFOA and BMI in boys 4–18 years	[81, 98, 111]	-0.0910 [-0.2488; 0.0668]	0.89	0.394	-0.0410 [-0.2285; 0.1464]
Childhood PFOA and BMI in girls 4–18 years	[81, 98, 111, 112]	-0.0150 [-0.0401; 0.0100]	0.74	0.256	-0.0134 [-0.0456; 0.0187]
Childhood PFOS and BMI in both sexes 4–18 years	[64, 79, 81, 90, 98, 104, 105, 111]	**-0.0166 [-0.0283; -0.0049]**	0.82	0.011	**-0.0226 [-0.0412; -0.0039]**
Childhood PFOS and BMI in boys 4–18 years	[81, 98, 111]	**-0.0229 [-0.0338; -0.0120]**	0.00	0.359	**-0.0235 [-0.0354; -0.0116]**
Childhood PFOS and BMI in girls 4–18 years	[81, 98, 111]	-0.0162 [-0.0531; 0.0207]	0.84	0.972	-0.0278 [-0.0719; 0.0164]
Childhood PFHxS and BMI in both sexes 4–18 years	[64, 81, 90, 98, 104, 111]	-0.0247 [-0.0814; 0.0320]	0.35	0.260	-0.0334 [-0.1157; 0.0489]
Childhood PFHxS and BMI in boys 4–18 years	[81, 98, 111]	-0.0547 [-0.2558; 0.1464]	0.18	0.444	-0.0331 [-0.1620; 0.0957]
Childhood PFHxS and BMI in girls 4–18 years	[81, 98, 111]	0.0667 [-0.1136; 0.2469]	0.35	0.079	0.0399 [-0.1249; 0.2047]
Childhood PFNA and BMI in both sexes 4–18 years	[64, 81, 90, 104, 111]	-0.2162 [-0.4883; 0.0558]	0.81	0.383	**-0.3279 [-0.6121; -0.0438]**
Childhood PFOA and risk of overweight in both sexes 4–18 years	[64, 90, 96, 108]	-0.0101 [-0.0497; 0.0295]	0.84	0.413	-0.0074 [-0.0554; 0.0407]
Childhood PFOS and risk of overweight in both sexes 4–18 years	[90, 96, 108]	0.0016 [-0.0081; 0.0113]	0.78	0.231	-0.0007 [-0.0137; 0.0123]

The sensitivity analyses stratified by age performed by excluding one study per time mostly confirmed the significant associations found, with an additional negative significant association in a single inverse variance method analysis between prenatal PFOA and BMI in children of both sexes who were than 3 years (Table S07).

## Discussion

We conducted a systematic review aimed to summarise the available evidence about an early-life exposure to PFASs (including prenatal exposure, including exposure through breastfeeding, and exposure in children) and the association with childhood overweight and obesity. Forty-nine different papers were included, among which 26 assessing prenatal exposure to PFASs, 17 childhood exposure, and 6 both. Overall, most associations were conflicting and there was no clear pattern of unequivocal evidence, but a certain number of studies reported of a negative association between PAFSs measured during childhood and outcomes assessed during childhood. We developed a methodology to convert the effect estimates from the different studies to make them comparable to each other, and when data were available and suitable from at least three studies, considering separately both sexes combined, only boys, and only girls, we conducted meta-analyses to pool the estimates. In particular, meta-analyses were performed with 30 studies (19 evaluating prenatal exposure, 7 evaluating childhood exposure, and 4 both). Considering differences in the two methods performed for pooling the evidence (inverse variance and weighting per sample size) and in the sensitivity analyses (excluding one sample per time), no overall strong significant association was observed between prenatal exposure to PFASs and the outcomes. Otherwise, relatively more consistent significant negative associations were found between childhood concentrations of PFASs (PFOA and PFOS in particular) and BMI in children.

Concerning the PFASs measured in the included studies, PFOA and PFOS were always quantified and PFHxS and PFNA were measured in several studies. These are legacy compounds, whose presence is very persistent and ubiquitous in the environments despite having been restricted. Moreover, in the last years, other emerging PFASs have been introduced in the market; they include molecules with a shorter carbon chain such as PFBA and PFBS [10, 113, 114]. Eleven studies quantified PFBS, one of which found positive associations between prenatal levels of PFBS and adiposity in girls [87], one found negative association between prenatal concentrations and WL and BMI [76], and one a negative association between childhood levels and overweight/obesity in children [106]; and only two studies quantified PFBA [98, 109]. Other emerging PFASs, such as the fluorotelomers and per-/polyfluoroalkyl ether acids [114], were not quantified in any studies. Furthermore, most studies did not report information about the chain branching of PFASs; indeed, both linear and branched isomers can be found in the environment, with potentially different implication for human health [115], and they can be separately quantified, as performed in Ye et al. 2018, Romano et al. 2022, and Janis et al. 2021 [68, 99, 107]. Unfortunately, there were not enough suitable data for inclusion in meta-analyses for any of these non-legacy PFASs.

Concerning the analytical assay, PFASs were quantified using liquid chromatography coupled with tandem mass spectrometry, with isotopic dilution; this can be considered the gold standard approach to quantify PFASs in biological samples [116, 117]. Moreover, some studies also reported validation data, in particular the limits of determination or quantitation (LOD/LOQ). A further support to the validity of the analytical measures would be the participation in interlaboratory quality assessment studies, such as the German External Quality Assessment Scheme (G -EQUAS) [118], which would certify a better reproducibility of the data among laboratories. Moreover, PFASs were measured in both serum and plasma; this is not regarded as a source of bias as no difference in the quantitation of PFASs was reported [119]. According to the new definition by Organisation for Economic Co-operation and Development (OECD), more than 6 million molecules in PubChem can be defined as PFASs [120, 121], thus suggesting that, although targeted biomarker investigations remain the gold standard in terms of accuracy and precision, also non-targeted and suspect screening investigations are required to have a better picture of the exposure to this vast class of compounds [122–125].

The outcomes considered for this review were measures of overweight, obesity, or adiposity. Almost all studies measured BMI in children, and some of them also WC and/or FM/BFP. A possible source of bias is represented by how the anthropometric measures were collected: they were measured by experts in most studies (34), while in few studies they were only reported by questionnaires or in other indirect ways (6), or by a hybrid way of the two (2), while the information about the collection of anthropometric measures was not clear in a few other studies (4) (Table S03). Although the measurements of height and weight performed by experts can reduce the bias of measurements, previous investigations reported that there is a good correlation between measured and self-reported measures [126, 127]. Furthermore, the evaluation of FM/BFP (19) was performed with different approaches including skinfold thickness (7), bioelectrical impedance analysis (BIA) (5), dual-energy x-ray absorptiometry (DXA) (6), air displacement plethysmography (ADP) (1). While DXA can be considered the gold standard, other approaches such as skinfold thickness, are generally considered a good proxy for measuring adiposity [44]; however, some limitations in their use have been pointed out, especially in children and adolescents of particular population [128, 129]. As previously reported, BMI can be considered a suitable surrogate measure of adiposity in children, even though it has a strong correlation with adiposity measured with DXA at higher level [130].

Although the reporting of the studies was judged of good quality, some limitations in the study design can be pointed out. Studies evaluating the prenatal exposure to PFASs were longitudinal studies (i.e., PFASs were measured during pregnancy or at birth and the outcomes were measured later in the childhood), while mostly of those assessing the exposure to PFASs in children are cross-sectional studies. Results from longitudinal analyses are more likely to suggest a possible causal relationship than cross-sectional studies. Only 5 studies quantifying PFASs in children were conducted measuring outcomes some years later [72, 79, 81, 108, 112] (all of them reported repeated measures during childhood at different ages; also, among those five studies, three of them found no significant associations, one found positive associations between PFOS at 9 years and measures of adiposity at 15 years, and one reported negative association between PFOA at 6–8 years and BMI and waist to height ratio in girls, even though decreasing with age). Finally, while several confounders were taken into account in the considered studies, the concomitant exposure to other persistent organic pollutants may also play a role [37] but it was not considered in most studies.

The meta-analyses allowed to perform a quantitative evaluation of the association between PFASs exposure and the considered outcome for 30 of the studies included in the systematic review. In order to include in the systematic review as many studies as possible, while also ensuring the data compatibility, a specific methodology was developed to convert the effect estimates from different studies (section “ 2.3.3 Data conversion of the effect estimates”). Overall, this methodology for conversion of estimates can be applied to other similar future meta-analyses. The two previous meta-analyses that evaluated the association of prenatal exposure of PFOA [36], and of PFOS or PFOA [37] and outcome of overweight or obesity in children, considered the effect estimates from the different studies regardless of whether the data used to calculate the estimates were log-transformed, without performing any conversion. Likewise, estimates obtained from continuous associations or from differences of percentiles were combined as such. Furthermore, these other two meta-analyses considered only studies that expressed BMI as z-scores, thus missing the information from those studies that reported the data as kg/m^2^; while this was taken into consideration in the present work thanks to the conversions carried out; similarly in the meta-analyses of Stratakis and co-workers [37] only studies reporting WC as cm were included, not considering not calculating the WC z-scores, while in the present work the estimates were converted and meta-analyses were performed on WC z-scores. Moreover, considering risk, Stratakis and co-workers considered only studies with RR. Indeed, the method used for data conversion developed for the present meta-analysis allowed the inclusion of a greater number of studies, while suitably taking into account the different ways data were reported from the included works. A possible limitation of the present methodology applied to the studies included in this systematic review, is that the conversions carried out to estimate z-scores, instead of raw measurements, gave very low standard errors of the slopes, that led to some meta-analyses where a few studies had a predominant weight. Also, for this purpose, further meta-analyses by weighting per sample-size were carried out and sensitivity analyses were performed by repeating the meta-analyses excluding one study at a time.

Comparing the results of the present work with the two previously published meta-analyses, our results can be considered in agreement with the one performed by Liu and co-workers, as they found a positive association between early life exposure to PFOA and childhood BMI z-score [36]: while we found no overall significant associations, we did find a significant association in the meta-analyses weighted per sample size considering children of both sexes who were more than 3 years (Table 4); indeed, the studies included in the meta-analyses by Liu et al., considered only children who were 5 or more years [63, 75, 79, 108, 112, 131]. On the other hand, Liu et al. found a significant positive association between PFOA and risk of overweight, while we did not. A systematic review with meta-analysis conducted by Stratakis and co-workers investigated the association between prenatal exposure to persistent organic pollutants and childhood obesity [37]. Although some differences between that and our work, among which some additional studies included in the present work, the conversion of estimates that we carried out prior the meta-analyses, and our additional analyses stratified by sex, the overall conclusion about the absence of a clear overall association between prenatal exposure to PFOA and PFOS and childhood BMI was the same. Furthermore, both this review and the one conducted by Stratakis et al. considered waist circumference as an outcome and we both found no considerable significant associations with prenatal PFOA and PFOS. Other differences between our work and the one from Stratakis and co-workers include: other persistent organic pollutants considered by Stratakis, more PFASs assessed in our work (in particular, meta-analyses also for PFHxS, PFNA, PFDA, PFUA, PFBS), further meta-analyses evaluating childhood exposure to PFASs performed in our work, and the methodology for data conversion of estimates.

There are also some limitations in performing the meta-analyses, due to differences in the designs of the studies, in particular for the various times, or range of times, the PFASs were quantified during gestation, as well as the different ages, or range of ages, the PFASs were measured in children. Likewise, the outcomes were measured at several different ages, or range of ages, among the studies. The stratified analyses performed separately with studies that considered children who were 3 or less years from those considering children who were more than 3 years allowed to mitigate this limitation. Furthermore, considering the meta-analyses performed to pool the evidence about the risk of overweight, even though we mathematically made comparable RR with OR and data normalised with different logarithms or comparing different ranges of concentrations, criteria for defining overweight varied across studies. Moreover, for some of the studies, the measures of outcomes suitable for this review were the result of a secondary analysis. Finally, since only studies published in indexed journals were considered, there is the possibility that other unpublished studies were missed, in particular studies reporting no statistically significant associations [131]. We tried to assess this source of bias with the funnel plots and the Egger’s test (Supplementary Figures). Moreover, the low number of studies available for some associations does not allow a firm conclusion with the performed meta-analysis. As an additional limitation, we did not use a defined method to assess bias such as the one used within the Navigation Guide [27], but we implemented a score to evaluate the quality of each included study and all studies were considered suitable for inclusion. Finally, having considered exclusively papers in English as inclusion criteria is an additional limitation of the present review and a possible source bias [132].

To our knowledge, this is the first systematic review conducting meta-analyses evaluating the exposure to PFASs in children and their associations with childhood overweight and obesity, although this evidence comes mostly from cross sectional studies, thus limiting the causal relationship. The developed methodology for the conversion of effect estimates before performing the meta-analyses can be implemented in future meta-analyses in environmental epidemiology to ensure a better comparability of data among studies.

In conclusion, this systematic review with meta-analyses evidenced positive associations between prenatal concentrations of PFOA and BMI in children who were more than 3 years, and between prenatal PFNA and BMI (particularly in boys who were more than 3 years). Negative associations were found between prenatal PFOS and BMI in children who were 3 or less years, and between PFHxS and risk of overweight. Positive associations were also found between prenatal PFNA and WC, and between prenatal PFUA and WC (even though the latter not confirmed in sensitivity analyses). Nevertheless, these results were not consistent across meta-analyses performed with the generic invariance method or by pooling per sample size. The evidence pooled from studies measuring PFASs during childhood was more consistent as confirmed in both generic invariance and weighting per sample size analyses, and also in most sensitivity analyses: negative associations were found between PFOA and BMI, PFOS and BMI (in particular in boys), and between PFNA and BMI (the latter only weighting by sample size), even though heterogeneity among studies was high.

### Supplementary Information

**Additional file 1: Supplementary text.** The supplementary text contains the text strings used for the search in PubMed and Embase and all the R scripts implemented prepared for this work

**Additional file 2: Supplementary figures.** In the supplementary figures file, for each meta-analysis, three figures are reported: a forest plot pooling the estimates with the generic invariance method, a forest plot pooling the estimates weighting by sample size, and a funnel plot evaluating the bias for included studies

**Additional file 3: Supplementary Table S01.** Table of entries retrieved from PubMed

**Additional file 4: Supplementary Table S02.** Table of entries retrieved from Embase

**Additional file 5: Supplementary Table S03.** Complete database of the considered studies, including: information retrieved from PubMed and Embase; whether the study was included or not in the systematic review and the category of inclusion or rejection; information collected from included articles; and score of reporting assigned to included articles

**Additional file 6: Supplementary Table S04.** Data extracted from articles used for performing the meta-analyses

**Additional file 7: Supplementary Table S05.** Data extracted transformed using the conversion factor developed

**Additional file 8: Supplementary Table S06.** Results of the meta-analyses performed with the generic invariance method (“inv_var”) and weighting by sample size (“wgtd”). The I2 as a measure of heterogeneity is also reported. Both the original meta-analyses are reported together with the sensitivity meta-analyses repeated by excluding one sample per time

**Additional file 9: Supplementary Table S07.** Results of the meta-analyses stratified by age (1-3 years or 4-18 years). Meta-analyses performed with the generic invariance method (“inv_var”) and weighting by sample size (“wgtd”) are reported. The I2 as a measure of heterogeneity is also reported. Both the original meta-analyses are reported together with the sensitivity meta-analyses repeated by excluding one sample per time

## Data Availability

All the data are reported in the supplementary material of the present article, including all the R-scripts developed for this work.

## Abbreviations

6:2 diPAP: 6:2 Polyfluoroalkyl phosphoric acid diesters
8:2 diPAP: 8:2 Polyfluoroalkyl phosphoric acid diesters
95% CI: 95% Confidence interval
ALSPAC: Avon Longitudinal Study of Parents and Children
BCERP: Breast Cancer and Environment Research Program
BFP: Body fat percentage
BMI: Body mass idex
CI: Confidence interval
CLEAR: Climate change, environmental contaminants and reproductive health
COPSAC-2010: Copenhagen Prospective Studies of Asthma in Childhood 2010
DXA: Dual-Energy X-ray Absorptiometry
ECHOFGS: The Environmental Influences on Child Health Outcomes Study
EDCs: Endocrine-disrupting chemicals
EYHS: European Youth Heart Study
FLEHS: Flemish Environment and Health Studies
FM: Fat mass
HBC: Hamamatsu Birth Cohort for Mothers and Children
HBM4EU: The Human Biomonitoring for Europe initiative
HELIX: Human Early Life Exposome project
HOME: Health Outcomes and Measures of the Environment
INMA: INfancia y Medio Ambiente, Environment and Childhood
INUENDO: Biopersistent organochlorines in diet and human fertility
LL: Lower limit of the 95% confidence interval
LOD: Limit of detection
LOQ: Limit of quantitation
LWBC: Laizhou Wan (Bay) Birth Cohort
N-EtFOSAA: N-Ethylperfluorooctane sulfonamidoacetic acid
NHANES: National Health and Nutrition Examination Survey
NICHD: National Institute of Child Health and Human Development
NICHD-SGA: U.S. National Institute of Child Health and Human Development Scandinavian Successive Small-for-Gestational Age births study
N-MeFOSAA: N-Methylperfluorooctane sulfonamidoacetic acid
n-PFOA: Linear perfluorooctanoic acid
n-PFOS: Linear perfluorooctanesulfonic acid
NHBCS: New Hampshire Birth Cohort Study
OCC: Odense Child Cohort
PFASs: Per-/polyfluoroalkyl substances
OR: Odds ration
PFBA: Perfluorobutanoic acid
PFBS: Perfluorobutanesulfonic acid
PFDA: Perfluorodecanoid acid
PFDoDA/PFDoA: Perfluorododecanoic acid
PFDS: Perfluorodecane sulfonic acid
PFHpA: Perfluoroheptanoic acid
PFHpS: Perfluoroheptanesulfonic acid
PFHxA: Perfluorohexanoic acid
PFHxS: Perfluorohexanesulfonic acid
PFNA: Perfluorononanoic acid
PFOA: Perfluorooctanoic acid
PFOS: Perfluorooctanesulfonic acid
PFOSA: Perfluorooctanesulfonamide
PFPeA: Perfluoropentanoic acid
PFTeDA: Perfluorotetradecanoic acid
PFTrDA: Perfluorotridecanoic acid
PFUnDA/PFUA: Perfluoroundecanoic acid
POPUP: Persistent Organic Pollutants in Uppsala Primiparas
REML: Restricted maximum-likelihood estimator
RR: Risk ratio
Sb-PFOA: Sum of branched isomers of perfluorooctanoic acid
Sm-PFOS: Sum of perfluoromethylheptane sulfonate isomers
STROBE-ME: Strengthening Reporting of Observational studies in Epidemiology-Molecular Epidemiology
UL: Upper limit of the 95% confidence interval
WA: Weight-for-age
WC: Waist circumference
WHO: World Health Organization
WL: Weight-for-length
WtHe: Waist to height ratio
WtHi: Waist to hip ratio
