# Supplementary material for: Prenatal and childhood exposure to per-/polyfluoroalkyl substances (PFASs) and its associations with childhood overweight and/or obesity: a systematic review with meta-analyses
Source: Environ Health. 2023 Aug 14;22:56. doi: 10.1186/s12940-023-01006-6 (PMC10424367; doi:10.1186/s12940-023-01006-6)
Supplement: Supplementary file 1 — Additional file 1: Supplementary text. The supplementary text contains the text strings used for the search in PubMed and Embase and all the R scripts implemented prepared for this work [file 12940_2023_1006_MOESM1_ESM.docx]

Prenatal and childhood exposure to per-/polyfluoroalkyl substances (PFASs) and its associations with childhood overweight and/or obesity: a systematic review with meta-analyses

Gianfranco Frigerio, Chiara Matilde Ferrari, and Silvia Fustinoni

**Supplementary text**

**Text string used for the search in PubMed:**

(((("Pediatric Obesity"[Mesh]) OR ("Obesity"[Mesh] AND (allchild[Filter] OR "Breast Feeding"[Mesh])) OR ("Overweight"[Mesh] AND (allchild[Filter] OR "Breast Feeding"[Mesh])) OR ("Overnutrition"[Mesh] AND (allchild[Filter] OR "Breast Feeding"[Mesh]))) AND (("Fluorocarbons"[Mesh]) OR ("Hydrocarbons, Fluorinated "[MESH]) OR ("Fluorocarbon Polymers "[MESH]) OR ("perfluoropentanoic acid" [Supplementary Concept]) OR ("perfluorododecanoic acid" [Supplementary Concept]) OR ("perfluorohexanesulfonic acid" [Supplementary Concept]) OR ("2-((6-chloro-1,1,2,2,3,3,4,4,5,5,6,6-dodecafluorohexyl)oxy)-1,1,2,2-tetrafluoroethanesulfonic acid" [Supplementary Concept]))) OR ((newborn*[Title/Abstract] OR neonat*[Title/Abstract] OR infan*[Title/Abstract] OR toddler*[Title/Abstract] OR kid[Title/Abstract] OR kids[Title/Abstract] OR baby[Title/Abstract] OR babies[Title/Abstract] OR boy[Title/Abstract] OR boys[Title/Abstract] OR girl*[Title/Abstract] OR child*[Title/Abstract] OR adolescen*[Title/Abstract] OR teen*[Title/Abstract] OR pediatr*[Title/Abstract] OR paediatr*[Title/Abstract] OR prenat*[Title/Abstract] OR intrauter*[Title/Abstract] OR pregnant*[Title/Abstract] OR gestat*[Title/Abstract] OR gravid*[Title/Abstract] OR fetus[Title/Abstract] OR breastfeed*[Title/Abstract] OR nourish[Title/Abstract] OR nursing[Title/Abstract] OR suckling[Title/Abstract] OR lactate[Title/Abstract] OR milk[Title/Abstract]) AND ((Obes*[Title/Abstract]) OR (Overweight*[Title/Abstract]) OR (Overnutrition[Title/Abstract]) OR (BMI[Title/Abstract]) OR (“body mass index”[Title/Abstract]) OR (weight[Title/Abstract]) OR (height[Title/Abstract]) OR (“waist circumference”[Title/Abstract]) OR (WC[Title/Abstract]) OR (“fat mass”[Title/Abstract]) OR (FM[Title/Abstract]) OR (“body fat”[Title/Abstract]) OR (adipos*[Title/Abstract])) AND ((Fluorocarb*[Title/Abstract]) OR (polyfluoro*[Title/Abstract]) OR (perfluoro*[Title/Abstract]) OR (PFAS[Title/Abstract]) OR (PFBA[Title/Abstract]) OR (PFPeA[Title/Abstract]) OR (PFHxA[Title/Abstract]) OR (PFHpA[Title/Abstract]) OR (PFOA[Title/Abstract]) OR (PFNA[Title/Abstract]) OR (PFDA[Title/Abstract]) OR (PFUnDA[Title/Abstract]) OR (PFDoDA[Title/Abstract]) OR (PFTrDA[Title/Abstract]) OR (PFTeDA[Title/Abstract]) OR (PFOSA[Title/Abstract]) OR (MeFOSAA[Title/Abstract]) OR (Methylperfluoro*[Title/Abstract]) OR (EtFOSAA[Title/Abstract]) OR (Ethylperfluoro*[Title/Abstract]) OR (PFBS[Title/Abstract]) OR (PFPeS[Title/Abstract]) OR (PFHxS[Title/Abstract]) OR (PFHpS[Title/Abstract]) OR (PFOS[Title/Abstract]) OR (PFNS[Title/Abstract]) OR (PFDS[Title/Abstract]) OR (FTSA[Title/Abstract]) OR (GenX[Title/Abstract]) OR (HFPO[Title/Abstract]) OR ("2,3,3,3-Tetrafluoro-2-(heptafluoropropoxy)propan*"[Title/Abstract]) OR (C6O4[Title/Abstract]) OR (DONA[Title/Abstract]) OR (PFESA[Title/Abstract]) OR (DIOX[Title/Abstract]) OR (“F-53B”[Title/Abstract]) OR (PFECHS[Title/Abstract]) OR (PFEtCHxS[Title/Abstract]))))

**Filter: publication year from 01-01-2000 to 31-12-2022**

**Text string used for the search in Embase:**

(((('childhood obesity'/exp) OR ('obesity'/exp AND ([adolescent]/lim OR [child]/lim OR [embryo]/lim OR [fetus]/lim OR [infant]/lim OR [newborn]/lim OR [preschool]/lim OR [school]/lim OR [young adult]/lim OR 'breast feeding'/exp)) OR ('body weight disorder'/exp AND ([adolescent]/lim OR [child]/lim OR [embryo]/lim OR [fetus]/lim OR [infant]/lim OR [newborn]/lim OR [preschool]/lim OR [school]/lim OR [young adult]/lim OR 'breast feeding'/exp)) OR ('overnutrition'/exp AND ([adolescent]/lim OR [child]/lim OR [embryo]/lim OR [fetus]/lim OR [infant]/lim OR [newborn]/lim OR [preschool]/lim OR [school]/lim OR [young adult]/lim OR 'breast feeding'/exp))) AND ('fluorocarbon'/exp OR 'fluorinated hydrocarbon'/exp OR 'perfluoroalkanoic acid'/exp OR 'perfluoro compound'/exp OR 'organofluorine derivative'/exp OR 'perfluorobutanoic acid'/exp OR 'perfluoropentanoic acid'/exp OR 'perfluoroheptanoic acid'/exp OR 'perfluorodecanoic acid'/exp OR 'perfluorotridecanoic acid'/exp OR 'perfluorotetradecanoic acid'/exp OR 'perfluorooctanesulfonamide'/exp OR 'n ethylperfluorooctanesulfonamide'/exp OR 'perfluorobutanesulfonic acid'/exp OR 'perfluorohexanesulfonic acid'/exp OR 'perfluoroheptane sulfonic acid'/exp OR 'perfluorooctanesulfonic acid'/exp OR 'perfluorodecane sulfonic acid'/exp OR 'perfluorodecanesulfonic acid'/exp)) OR ((newborn*:ti,ab,kw OR neonat*:ti,ab,kw OR infan*:ti,ab,kw OR toddler*:ti,ab,kw OR kid:ti,ab,kw OR kids:ti,ab,kw OR baby:ti,ab,kw OR babies:ti,ab,kw OR boy:ti,ab,kw OR boys:ti,ab,kw OR girl*:ti,ab,kw OR child*:ti,ab,kw OR adolescen*:ti,ab,kw OR teen*:ti,ab,kw OR pediatr*:ti,ab,kw OR paediatr*:ti,ab,kw OR prenat*:ti,ab,kw OR intrauter*:ti,ab,kw OR pregnant*:ti,ab,kw OR gestat*:ti,ab,kw OR gravid*:ti,ab,kw OR fetus:ti,ab,kw OR breastfeed*:ti,ab,kw OR nourish:ti,ab,kw OR nursing:ti,ab,kw OR suckling:ti,ab,kw OR lactate:ti,ab,kw OR milk:ti,ab,kw) AND (Obes*:ti,ab,kw OR Overweight*:ti,ab,kw OR Overnutrition:ti,ab,kw OR BMI:ti,ab,kw OR “body mass index”:ti,ab,kw OR weight:ti,ab,kw OR height:ti,ab,kw OR “waist circumference”:ti,ab,kw OR WC:ti,ab,kw OR “fat mass”:ti,ab,kw OR FM:ti,ab,kw OR “body fat”:ti,ab,kw OR adipos*:ti,ab,kw) AND (Fluorocarb*:ti,ab,kw OR polyfluoro*:ti,ab,kw OR perfluoro*:ti,ab,kw OR PFAS:ti,ab,kw OR PFBA:ti,ab,kw OR PFPeA:ti,ab,kw OR PFHxA:ti,ab,kw OR PFHpA:ti,ab,kw OR PFOA:ti,ab,kw OR PFNA:ti,ab,kw OR PFDA:ti,ab,kw OR PFUnDA:ti,ab,kw OR PFDoDA:ti,ab,kw OR PFTrDA:ti,ab,kw OR PFTeDA:ti,ab,kw OR PFOSA:ti,ab,kw OR MeFOSAA:ti,ab,kw OR Methylperfluoro*:ti,ab,kw OR EtFOSAA:ti,ab,kw OR Ethylperfluoro*:ti,ab,kw OR PFBS:ti,ab,kw OR PFPeS:ti,ab,kw OR PFHxS:ti,ab,kw OR PFHpS:ti,ab,kw OR PFOS:ti,ab,kw OR PFNS:ti,ab,kw OR PFDS:ti,ab,kw OR FTSA:ti,ab,kw OR GenX:ti,ab,kw OR HFPO:ti,ab,kw OR "2,3,3,3-Tetrafluoro-2-heptafluoropropoxypropan*":ti,ab,kw OR C6O4:ti,ab,kw OR DONA:ti,ab,kw OR PFESA:ti,ab,kw OR DIOX:ti,ab,kw OR “F-53B”:ti,ab,kw OR PFECHS:ti,ab,kw OR PFEtCHxS:ti,ab,kw OR '375 22 4':rn OR '2706 90 3':rn OR '307 24 4':rn OR '375 85 9':rn OR '335 67 1':rn OR '375 95 1':rn OR '335 76 2':rn OR '2058 94 8':rn OR '307 55 1':rn OR '72629 94 8':rn OR '376 06 7':rn OR '754 91 6':rn OR '2355 31 9':rn OR '2991 50 6':rn OR '375 73 5':rn OR '2706 91 4':rn OR '355 46 4':rn OR '375 92 8':rn OR '1763 23 1':rn OR '68259 12 1':rn OR '335 77 3':rn OR '757124 72 4':rn OR '27619 97 2':rn OR '39108 34 4':rn OR '13252 13 6':rn OR '1190931 41 9':rn OR '919005 14 4':rn OR '763051 92 9':rn OR '756426 58 1':rn OR '646 83 3':rn)) AND (2000:py OR 2001:py OR 2002:py OR 2003:py OR 2004:py OR 2005:py OR 2006:py OR 2007:py OR 2008:py OR 2009:py OR 2010:py OR 2011:py OR 2012:py OR 2013:py OR 2014:py OR 2015:py OR 2016:py OR 2017:py OR 2018:py OR 2019:py OR 2020:py OR 2021:py OR 2022:py))

**R script:**

####################start of the script##############

library(tidyverse)

library(meta)

library(anthro)

library(rmeta)

library(grid)

library(gridExtra)

######### This script can be subdivided in 7 sections:

# Section 1: "Merging the databases of studies"

# Section 2: "Transforming effect estimates from studies"

# Section 3: "Performing the meta-analyses"

# Section 4: "sensitivity analysis"

# Section 5: "Meta-analyses stratified by age"

# Section 6: "Meta-analyses stratified by age - sensitivity analyses"

# Section 7: "Developing the superimposed forest plots"

####### Section 1: "Merging the databases of studies"

#### In this section, articles from PubMed and Embase were merged based on the DOI

### Loading the data obtained from the two databases:

PM_tot <- read_csv("Frigerio_PFASs_rev_TableS01_All_PubMed.csv") %>%

mutate(DOI = tolower(DOI))

Em_tot <- read_csv("Frigerio_PFASs_rev_TableS02_All_Embase.csv") %>%

mutate(DOI = tolower(DOI))

### creating a column with TRUE or FALSE to verify which studies are present in each other databases

PM <- PM_tot %>%

add_column(code = paste0("nPM", str_pad(1:length(PM_tot$Title), width = 3, side = "left", pad = "0")), .before = 1) %>%

mutate(pres_in_Em_tot_DOI = ifelse(is.na(DOI),NA,ifelse(DOI %in% Em_tot$DOI, TRUE, FALSE)))

Em <- Em_tot %>%

add_column(code = paste0("nEm", str_pad(1:length(Em_tot$Title), width = 3, side = "left", pad = "0")), .before = 1) %>%

mutate(pres_in_PM_tot_DOI = ifelse(is.na(DOI),NA,ifelse(DOI %in% PM_tot$DOI, TRUE, FALSE)))

## check if the entries to be merged are actually of the same number:

summarise(PM, pres_Em_DOI = sum(pres_in_Em_tot_DOI, na.rm=TRUE))

summarise(Em, pres_PM_DOI = sum(pres_in_PM_tot_DOI, na.rm=TRUE))

## the number is not consistent, checking where the problem is:

which(duplicated(filter(PM, pres_in_Em_tot_DOI == TRUE)$DOI))

which(duplicated(filter(Em, pres_in_PM_tot_DOI == TRUE)$DOI))

## So, it seems that there are 2 pairs of entries in the Embase database with the same DOI that don't allow a consistent merge

## Fixing the issue:

duplicates <- which(Em_tot$DOI %in% filter(Em, pres_in_PM_tot_DOI == TRUE)$DOI[duplicated(filter(Em, pres_in_PM_tot_DOI == TRUE)$DOI)])

Em_tot_edit <- Em_tot

Em_tot_edit$DOI[duplicates[2]] <- paste0(Em_tot$DOI[duplicates[2]], "_BIS")

Em_tot_edit$DOI[duplicates[3]] <- paste0(Em_tot$DOI[duplicates[3]], "_BIS")

## So, preparing the data frames again:

PM_edit <- PM_tot %>%

add_column(code = paste0("nPM", str_pad(1:length(PM_tot$Title), width = 3, side = "left", pad = "0")), .before = 1) %>%

mutate(pres_in_Em_tot_DOI = ifelse(is.na(DOI),NA,ifelse(DOI %in% Em_tot_edit$DOI, TRUE, FALSE)))

Em_edit <- Em_tot_edit %>%

add_column(code = paste0("nEm", str_pad(1:length(Em_tot$Title), width = 3, side = "left", pad = "0")), .before = 1) %>%

mutate(pres_in_PM_tot_DOI = ifelse(is.na(DOI),NA,ifelse(DOI %in% PM_tot$DOI, TRUE, FALSE)))

## now, check:

summarise(PM_edit, pres_Em_DOI = sum(pres_in_Em_tot_DOI, na.rm=TRUE))

summarise(Em_edit, pres_PM_DOI = sum(pres_in_PM_tot_DOI, na.rm=TRUE))

## now the number is consistent, we can merge:

colnames(PM_edit) <- paste0("PM_", colnames(PM_edit))

colnames(Em_edit) <- paste0("Em_", colnames(Em_edit))

PM_in_comm_with_Em <- filter(PM_edit, PM_pres_in_Em_tot_DOI == TRUE)

Em_in_comm_with_PM <- filter(Em_edit, Em_pres_in_PM_tot_DOI == TRUE)

PM_not_in_comm_with_Em <- filter(PM_edit, PM_pres_in_Em_tot_DOI == FALSE | is.na(PM_pres_in_Em_tot_DOI))

Em_not_in_comm_with_PM <- filter(Em_edit, Em_pres_in_PM_tot_DOI == FALSE | is.na(Em_pres_in_PM_tot_DOI))

PM_in_comm_with_Em <- arrange(PM_in_comm_with_Em, PM_DOI)

Em_in_comm_with_PM <- arrange(Em_in_comm_with_PM, Em_DOI)

DB_merged1 <- cbind(PM_in_comm_with_Em, Em_in_comm_with_PM)

DB_merged2_left <- PM_not_in_comm_with_Em

DB_merged2_right <- as_tibble(matrix(data = rep(NA, length(PM_not_in_comm_with_Em$PM_code)*length(colnames(Em_not_in_comm_with_PM))),

nrow = length(PM_not_in_comm_with_Em$PM_code),

ncol = length(colnames(Em_not_in_comm_with_PM)),

dimnames = list(NULL,

colnames(Em_not_in_comm_with_PM))))

DB_merged2 <- cbind(DB_merged2_left, DB_merged2_right)

DB_merged3_left <- as_tibble(matrix(data = rep(NA, length(Em_not_in_comm_with_PM$Em_code)*length(colnames(PM_not_in_comm_with_Em))),

nrow = length(Em_not_in_comm_with_PM$Em_code),

ncol = length(colnames(PM_not_in_comm_with_Em)),

dimnames = list(NULL,

colnames(PM_not_in_comm_with_Em))))

DB_merged3_right <- Em_not_in_comm_with_PM

DB_merged3 <- cbind(DB_merged3_left, DB_merged3_right)

DB_merged <- rbind(DB_merged1, DB_merged2, DB_merged3)

DB_merged <- DB_merged %>%

arrange(PM_code, Em_code) %>%

add_column(Code = paste0("n", str_pad(1:length(DB_merged$PM_code), width = 3, side = "left", pad = "0")), .before = 1) %>%

arrange(Code)

write_tsv(DB_merged, "Frigerio_PFASs_rev_PM_Em_studies_merged.txt")

### This data frame was then manually modified in Excel, used for the study evaluation and inclusion, for the collection of information from studies, and for the quality of reporting. The final Excel database is reported in the Supplementary Table S03

####### End of Section 1 #######

######## Section 2: "Transforming effect estimates from studies"

#### In this section, data obtained from studies were transformed to be comparable each other

data_extracted <- read_tsv("Frigerio_PFASs_rev_TableS04_estr_estim.txt")

## loading tables for z-scores

## BMI:

# from: https://www.who.int/toolkits/child-growth-standards/standards/body-mass-index-for-age-bmi-for-age

BMI_girls_0_13_w <- read_tsv("WHO_bmi_girls_0-to-13-weeks_zscores.txt") %>% ## table downloaded from https://cdn.who.int/media/docs/default-source/child-growth/child-growth-standards/indicators/body-mass-index-for-age/bmi_girls_0-to-13-weeks_zscores.xlsx?sfvrsn=7668918a_7

mutate(Day = Week*7)

BMI_girls_0_2_y <- read_tsv("WHO_bmi_girls_0-to-2-years_zscores.txt") %>% ## table downloaded from https://cdn.who.int/media/docs/default-source/child-growth/child-growth-standards/indicators/body-mass-index-for-age/bmi_girls_0-to-2-years_zscores.xlsx?sfvrsn=2be9859c_7

mutate(Day = Month*30)

BMI_girls_2_5_y <- read_tsv("WHO_bmi_girls_2-to-5-years_zscores.txt") %>% ## table downloaded from https://cdn.who.int/media/docs/default-source/child-growth/child-growth-standards/indicators/body-mass-index-for-age/bmi_girls_2-to-5-years_zscores.xlsx?sfvrsn=452aca36_7

mutate(Day = Month*30)

BMI_boys_0_13_w <- read_tsv("WHO_bmi_boys_0-to-13-weeks_zscores.txt") %>% ## table downloaded from https://cdn.who.int/media/docs/default-source/child-growth/child-growth-standards/indicators/body-mass-index-for-age/bmi_boys_0-to-13-weeks_zscores.xlsx?sfvrsn=8eff3d30_7

mutate(Day = Week*7)

BMI_boys_0_2_y <- read_tsv("WHO_bmi_boys_0-to-2-years_zcores.txt") %>% ## table downloaded from https://cdn.who.int/media/docs/default-source/child-growth/child-growth-standards/indicators/body-mass-index-for-age/bmi_boys_0-to-2-years_zcores.xlsx?sfvrsn=df725cc9_7

mutate(Day = Month*30)

BMI_boys_2_5_y <- read_tsv("WHO_bmi_boys_2-to-5-years_zscores.txt") %>% ## table downloaded from https://cdn.who.int/media/docs/default-source/child-growth/child-growth-standards/indicators/body-mass-index-for-age/bmi_boys_2-to-5-years_zscores.xlsx?sfvrsn=73010c9b_5

mutate(Day = Month*30)

# from https://www.who.int/tools/growth-reference-data-for-5to19-years/indicators/bmi-for-age

BMI_girls_5_19_y <- read_tsv("WHO_bmi_girls_5-to-19-years_zscores.txt") %>% ## table downloaded from https://cdn.who.int/media/docs/default-source/child-growth/growth-reference-5-19-years/bmi-for-age-(5-19-years)/bmi-girls-z-who-2007-exp.xlsx?sfvrsn=79222875_2

mutate(Day = Month*30)

BMI_boys_5_19_y <- read_tsv("WHO_bmi_boys_5-to-19-years_zscores.txt") %>% ## table downloaded from https://cdn.who.int/media/docs/default-source/child-growth/growth-reference-5-19-years/bmi-for-age-(5-19-years)/bmi-boys-z-who-2007-exp.xlsx?sfvrsn=a84bca93_2

mutate(Day = Month*30)

## WC:

# from Table S01 of Sharma, A., Metzger, D., Daymont, C. et al. LMS tables for waist-circumference and waist-height ratio Z-scores in children aged 5-19 y in NHANES III: association with cardio-metabolic risks. Pediatr Res 78, 723-729 (2015).

# https://doi.org/10.1038/pr.2015.160

WC_girls_5_19_y <- read_tsv("Sharma_et_al_WC_girls_5-to-19-years_zscores.txt") %>% ## table downloaded from https://static-content.springer.com/esm/art%3A10.1038%2Fpr.2015.160/MediaObjects/41390_2015_BFpr2015160_MOESM39_ESM.xls

mutate(Day = month*30)

WC_boys_5_19_y <- read_tsv("Sharma_et_al_WC_boys_5-to-19-years_zscores.txt") %>% ## table downloaded from https://static-content.springer.com/esm/art%3A10.1038%2Fpr.2015.160/MediaObjects/41390_2015_BFpr2015160_MOESM39_ESM.xls

mutate(Day = month*30)

## FMI:

# from Table 2 of Weber DR, Moore RH, Leonard MB, Zemel BS. Fat and lean BMI reference curves in children and adolescents and their utility in identifying excess adiposity compared with BMI and percentage body fat. Am J Clin Nutr. 2013 Jul;98(1):49-56.

# doi: 10.3945/ajcn.112.053611

FMI_girls_8_20_y <- read_tsv("Weber_et_al_FMI_girls_8-to-20-years_zscores.txt") %>%

mutate(M = `50th`, Age_to_separate = Age) %>%

separate(col = Age_to_separate, into = c("Year1", "Year2"), sep = "-") %>%

mutate(Year1 = as.numeric(Year1), Year2 = as.numeric(str_replace(Year2, " y", ""))) %>%

rowwise() %>%

mutate(Year_mean = mean(c(Year1, Year2)), Day = Year_mean*365)

FMI_boys_8_20_y <- read_tsv("Weber_et_al_FMI_boys_8-to-20-years_zscores.txt") %>%

mutate(M = `50th`, Age_to_separate = Age) %>%

separate(col = Age_to_separate, into = c("Year1", "Year2"), sep = "-") %>%

mutate(Year1 = as.numeric(Year1), Year2 = as.numeric(str_replace(Year2, " y", ""))) %>%

rowwise() %>%

mutate(Year_mean = mean(c(Year1, Year2)), Day = Year_mean*365)

data_extracted_transf <- data_extracted %>%

# first convert the RR in OR:

mutate(estim_v0 = estim, ci95_lower_v0 = ci95_lower, ci95_upper_v0 = ci95_upper) %>%

mutate(estim_va = ifelse(outcome_variables_type == "RR",

(estim_v0-overweight_prevalence*estim_v0)/(1-overweight_prevalence*estim_v0), estim_v0),

ci95_lower_va = ifelse(outcome_variables_type == "RR",

(ci95_lower_v0-overweight_prevalence*ci95_lower_v0)/(1-overweight_prevalence*ci95_lower_v0), ci95_lower_v0),

ci95_upper_va = ifelse(outcome_variables_type == "RR",

(ci95_upper_v0-overweight_prevalence*ci95_upper_v0)/(1-overweight_prevalence*ci95_upper_v0), ci95_upper_v0)) %>%

# converting OR in standardized mean difference:

mutate(estim_vb = ifelse(outcome_variables_type %in% c("RR", "OR"),

log10(estim_va)*(sqrt(3)/pi), estim_va),

ci95_lower_vb = ifelse(outcome_variables_type %in% c("RR", "OR"),

log10(ci95_lower_va)*(sqrt(3)/pi), ci95_lower_va),

ci95_upper_vb = ifelse(outcome_variables_type %in% c("RR", "OR"),

log10(ci95_upper_va)*(sqrt(3)/pi), ci95_upper_va)) %>%

## then:

mutate(se_vb = (ci95_upper_vb - ci95_lower_vb)/3.92) %>% #to calculate standard error

mutate(estim_v1 = estim_vb, ci95_lower_v1 = ci95_lower_vb, ci95_upper_v1 = ci95_upper_vb, se_v1 = se_vb) %>%

mutate(estim_v2 = ifelse(estimate_reported != "% change in the outcome",

ifelse(PFAS_variable_type == "unit", estim_v1,

ifelse(PFAS_variable_type == "ln", log((1+(1/Mean_or_Median_PFAS_ngmL)), base = exp(1))*estim_v1,

ifelse(PFAS_variable_type == "log10", log((1+(1/Mean_or_Median_PFAS_ngmL)), base = 10)*estim_v1,

ifelse(PFAS_variable_type == "log2", log((1+(1/Mean_or_Median_PFAS_ngmL)), base = 2)*estim_v1, estim_v1)))), estim_v1),

ci95_lower_v2 = ifelse(estimate_reported != "% change in the outcome",

ifelse(PFAS_variable_type == "unit", estim_v1-(1.96*se_v1),

ifelse(PFAS_variable_type == "ln", log((1+(1/Mean_or_Median_PFAS_ngmL)), base = exp(1))*(estim_v1-(1.96*se_v1)),

ifelse(PFAS_variable_type == "log10", log((1+(1/Mean_or_Median_PFAS_ngmL)), base = 10)*(estim_v1-(1.96*se_v1)),

ifelse(PFAS_variable_type == "log2", log((1+(1/Mean_or_Median_PFAS_ngmL)), base = 2)*(estim_v1-(1.96*se_v1)), ci95_lower_v1)))), ci95_lower_v1),

ci95_upper_v2 = ifelse(estimate_reported != "% change in the outcome",

ifelse(PFAS_variable_type == "unit", estim_v1+(1.96*se_v1),

ifelse(PFAS_variable_type == "ln", log((1+(1/Mean_or_Median_PFAS_ngmL)), base = exp(1))*(estim_v1+(1.96*se_v1)),

ifelse(PFAS_variable_type == "log10", log((1+(1/Mean_or_Median_PFAS_ngmL)), base = 10)*(estim_v1+(1.96*se_v1)),

ifelse(PFAS_variable_type == "log2", log((1+(1/Mean_or_Median_PFAS_ngmL)), base = 2)*(estim_v1+(1.96*se_v1)), ci95_upper_v1)))), ci95_upper_v1),

estim_v3 = ifelse(estimate_reported == "% change in the outcome", # considering that the base of the log-transformation for Y is always e

ifelse(PFAS_variable_type == "unit", (exp(1)^(log((estim_v2/100)+1))-1)*Mean_or_Median_outcome,

ifelse(PFAS_variable_type == "ln", (exp(1)^(log(1+(1/Mean_or_Median_PFAS_ngmL), base = exp(1))*log((estim_v2/100)+1))-1)*Mean_or_Median_outcome,

ifelse(PFAS_variable_type == "log10", (exp(1)^(log(1+(1/Mean_or_Median_PFAS_ngmL), base = 10)*log((estim_v2/100)+1))-1)*Mean_or_Median_outcome,

ifelse(PFAS_variable_type == "log2", (exp(1)^(log(1+(1/Mean_or_Median_PFAS_ngmL), base = 2)*log((estim_v2/100)+1))-1)*Mean_or_Median_outcome, estim_v2)))), estim_v2),

ci95_lower_v3 = ifelse(estimate_reported == "% change in the outcome", # considering that the base of the log-transformation for Y is always e

ifelse(PFAS_variable_type == "unit", (exp(1)^(log((estim_v2/100)+1)-(1.96*log(((ci95_upper_v1 - ci95_lower_v1)/3.92/100)+1)))-1)*Mean_or_Median_outcome,

ifelse(PFAS_variable_type == "ln", (exp(1)^(log(1+(1/Mean_or_Median_PFAS_ngmL), base = exp(1))*(log((estim_v2/100)+1)-(1.96*log(((ci95_upper_v1 - ci95_lower_v1)/3.92/100)+1))))-1)*Mean_or_Median_outcome,

ifelse(PFAS_variable_type == "log10", (exp(1)^(log(1+(1/Mean_or_Median_PFAS_ngmL), base = 10)*(log((estim_v2/100)+1)-(1.96*log(((ci95_upper_v1 - ci95_lower_v1)/3.92/100)+1))))-1)*Mean_or_Median_outcome,

ifelse(PFAS_variable_type == "log2", (exp(1)^(log(1+(1/Mean_or_Median_PFAS_ngmL), base = 2)*(log((estim_v2/100)+1)-(1.96*log(((ci95_upper_v1 - ci95_lower_v1)/3.92/100)+1))))-1)*Mean_or_Median_outcome, ci95_lower_v2)))), ci95_lower_v2),

ci95_upper_v3 = ifelse(estimate_reported == "% change in the outcome", # considering that the base of the log-transformation for Y is always e

ifelse(PFAS_variable_type == "unit", (exp(1)^(log((estim_v2/100)+1)+(1.96*log(((ci95_upper_v1 - ci95_lower_v1)/3.92/100)+1)))-1)*Mean_or_Median_outcome,

ifelse(PFAS_variable_type == "ln", (exp(1)^(log(1+(1/Mean_or_Median_PFAS_ngmL), base = exp(1))*(log((estim_v2/100)+1)+(1.96*log(((ci95_upper_v1 - ci95_lower_v1)/3.92/100)+1))))-1)*Mean_or_Median_outcome,

ifelse(PFAS_variable_type == "log10", (exp(1)^(log(1+(1/Mean_or_Median_PFAS_ngmL), base = 10)*(log((estim_v2/100)+1)+(1.96*log(((ci95_upper_v1 - ci95_lower_v1)/3.92/100)+1))))-1)*Mean_or_Median_outcome,

ifelse(PFAS_variable_type == "log2", (exp(1)^(log(1+(1/Mean_or_Median_PFAS_ngmL), base = 2)*(log((estim_v2/100)+1)+(1.96*log(((ci95_upper_v1 - ci95_lower_v1)/3.92/100)+1))))-1)*Mean_or_Median_outcome, ci95_upper_v2)))), ci95_upper_v2),

estim_v4 = ifelse(estimate_reported %in% c("T3vsT1", "T2vsT1", "Q4vsQ1", "Q3vsQ1", "Q2vsQ1", "change per interquartile range", "change for each SD increase"), estim_v3/range_ngmL, estim_v3),

se_v4 = ifelse(estimate_reported %in% c("T3vsT1", "T2vsT1", "Q4vsQ1", "Q3vsQ1", "Q2vsQ1", "change per interquartile range", "change for each SD increase"), ((ci95_upper_v3 - ci95_lower_v3)/3.92)/range_ngmL, (ci95_upper_v3 - ci95_lower_v3)/3.92),

ci95_lower_v4 = estim_v4-(1.96*se_v4),

ci95_upper_v4 = estim_v4+(1.96*se_v4),

Calc_BMI_zscore = NA,

Calc_WC_zscore = NA,

Calc_FMI_zscore = NA,

estim_v5 = NA,

se_v5 = NA,

ci95_lower_v5 = NA,

ci95_upper_v5 = NA)

for (i in 1:length(data_extracted_transf$META)) {

compute_zscore <- function(zscoretable, Y) {

vect <- zscoretable$Day

my_number <- data_extracted_transf$age_in_days[i]

ref_zscore_table <- which(abs(vect-my_number)==min(abs(vect-my_number)))

return(anthro_api_compute_zscore(y = Y,

m = zscoretable$M[ref_zscore_table],

l = zscoretable$L[ref_zscore_table],

s = zscoretable$S[ref_zscore_table]))

}

out_data <- data_extracted_transf$Mean_or_Median_outcome[i]

if (data_extracted_transf$outcome_variables_type[i] == "BMI (kg/m2)") {

if (data_extracted_transf$age_in_days[i] < 7*13) {

if (data_extracted_transf$sex[i] == "girls") {

data_extracted_transf$Calc_BMI_zscore[i] <- compute_zscore(BMI_girls_0_13_w, Y = out_data)

} else if (data_extracted_transf$sex[i] == "boys") {

data_extracted_transf$Calc_BMI_zscore[i] <- compute_zscore(BMI_boys_0_13_w, Y = out_data)

} else if (data_extracted_transf$sex[i] == "both_sexes") {

data_extracted_transf$Calc_BMI_zscore[i] <- mean(c(compute_zscore(BMI_girls_0_13_w, Y = out_data), compute_zscore(BMI_boys_0_13_w, Y = out_data)))

}

} else if (data_extracted_transf$age_in_days[i] < 365*2) {

if (data_extracted_transf$sex[i] == "girls") {

data_extracted_transf$Calc_BMI_zscore[i] <- compute_zscore(BMI_girls_0_2_y, Y = out_data)

} else if (data_extracted_transf$sex[i] == "boys") {

data_extracted_transf$Calc_BMI_zscore[i] <- compute_zscore(BMI_boys_0_2_y, Y = out_data)

} else if (data_extracted_transf$sex[i] == "both_sexes") {

data_extracted_transf$Calc_BMI_zscore[i] <- mean(c(compute_zscore(BMI_girls_0_2_y, Y = out_data), compute_zscore(BMI_boys_0_2_y, Y = out_data)))

}

} else if (data_extracted_transf$age_in_days[i] < 365*5) {

if (data_extracted_transf$sex[i] == "girls") {

data_extracted_transf$Calc_BMI_zscore[i] <- compute_zscore(BMI_girls_2_5_y, Y = out_data)

} else if (data_extracted_transf$sex[i] == "boys") {

data_extracted_transf$Calc_BMI_zscore[i] <- compute_zscore(BMI_boys_2_5_y, Y = out_data)

} else if (data_extracted_transf$sex[i] == "both_sexes") {

data_extracted_transf$Calc_BMI_zscore[i] <- mean(c(compute_zscore(BMI_girls_2_5_y, Y = out_data), compute_zscore(BMI_boys_2_5_y, Y = out_data)))

}

} else if (data_extracted_transf$age_in_days[i] < 365*19) {

if (data_extracted_transf$sex[i] == "girls") {

data_extracted_transf$Calc_BMI_zscore[i] <- compute_zscore(BMI_girls_5_19_y, Y = out_data)

} else if (data_extracted_transf$sex[i] == "boys") {

data_extracted_transf$Calc_BMI_zscore[i] <- compute_zscore(BMI_boys_5_19_y, Y = out_data)

} else if (data_extracted_transf$sex[i] == "both_sexes") {

data_extracted_transf$Calc_BMI_zscore[i] <- mean(c(compute_zscore(BMI_girls_5_19_y, Y = out_data), compute_zscore(BMI_boys_5_19_y, Y = out_data)))

}

}

data_extracted_transf$estim_v5[i] <- (data_extracted_transf$Calc_BMI_zscore[i]*data_extracted_transf$estim_v4[i])/data_extracted_transf$Mean_or_Median_outcome[i]

data_extracted_transf$se_v5[i] <- abs((data_extracted_transf$Calc_BMI_zscore[i]*data_extracted_transf$se_v4[i])/data_extracted_transf$Mean_or_Median_outcome[i])

data_extracted_transf$ci95_lower_v5[i] <- data_extracted_transf$estim_v5[i]-(1.96*data_extracted_transf$se_v5[i])

data_extracted_transf$ci95_upper_v5[i] <- data_extracted_transf$estim_v5[i]+(1.96*data_extracted_transf$se_v5[i])

} else if (data_extracted_transf$outcome_variables_type[i] == "WC (cm)") {

if (data_extracted_transf$sex[i] == "girls") {

data_extracted_transf$Calc_WC_zscore[i] <- compute_zscore(WC_girls_5_19_y, Y = out_data)

} else if (data_extracted_transf$sex[i] == "boys") {

data_extracted_transf$Calc_WC_zscore[i] <- compute_zscore(WC_boys_5_19_y, Y = out_data)

} else if (data_extracted_transf$sex[i] == "both_sexes") {

data_extracted_transf$Calc_WC_zscore[i] <- mean(c(compute_zscore(WC_girls_5_19_y, Y = out_data), compute_zscore(WC_boys_5_19_y, Y = out_data)))

}

data_extracted_transf$estim_v5[i] <- (data_extracted_transf$Calc_WC_zscore[i]*data_extracted_transf$estim_v4[i])/data_extracted_transf$Mean_or_Median_outcome[i]

data_extracted_transf$se_v5[i] <- abs((data_extracted_transf$Calc_WC_zscore[i]*data_extracted_transf$se_v4[i])/data_extracted_transf$Mean_or_Median_outcome[i])

data_extracted_transf$ci95_lower_v5[i] <- data_extracted_transf$estim_v5[i]-(1.96*data_extracted_transf$se_v5[i])

data_extracted_transf$ci95_upper_v5[i] <- data_extracted_transf$estim_v5[i]+(1.96*data_extracted_transf$se_v5[i])

} else if (data_extracted_transf$outcome_variables_type[i] == "DXA total fat mass index (kg/m2)") {

if (data_extracted_transf$sex[i] == "girls") {

data_extracted_transf$Calc_FMI_zscore[i] <- compute_zscore(FMI_girls_8_20_y, Y = out_data)

} else if (data_extracted_transf$sex[i] == "boys") {

data_extracted_transf$Calc_FMI_zscore[i] <- compute_zscore(FMI_boys_8_20_y, Y = out_data)

} else if (data_extracted_transf$sex[i] == "both_sexes") {

data_extracted_transf$Calc_FMI_zscore[i] <- mean(c(compute_zscore(FMI_girls_8_20_y, Y = out_data), compute_zscore(FMI_boys_8_20_y, Y = out_data)))

}

data_extracted_transf$estim_v5[i] <- (data_extracted_transf$Calc_FMI_zscore[i]*data_extracted_transf$estim_v4[i])/data_extracted_transf$Mean_or_Median_outcome[i]

data_extracted_transf$se_v5[i] <- abs((data_extracted_transf$Calc_FMI_zscore[i]*data_extracted_transf$se_v4[i])/data_extracted_transf$Mean_or_Median_outcome[i])

data_extracted_transf$ci95_lower_v5[i] <- data_extracted_transf$estim_v5[i]-(1.96*data_extracted_transf$se_v5[i])

data_extracted_transf$ci95_upper_v5[i] <- data_extracted_transf$estim_v5[i]+(1.96*data_extracted_transf$se_v5[i])

} else if (data_extracted_transf$outcome_variables_type[i] == "DXA-total body fat (%)") {

out_data <- (data_extracted_transf$Mean_or_Median_outcome[i]/100)*data_extracted_transf$furhter_bmi[i]

if (data_extracted_transf$sex[i] == "girls") {

data_extracted_transf$Calc_FMI_zscore[i] <- compute_zscore(FMI_girls_8_20_y, Y = out_data)

} else if (data_extracted_transf$sex[i] == "boys") {

data_extracted_transf$Calc_FMI_zscore[i] <- compute_zscore(FMI_boys_8_20_y, Y = out_data)

} else if (data_extracted_transf$sex[i] == "both_sexes") {

data_extracted_transf$Calc_FMI_zscore[i] <- mean(c(compute_zscore(FMI_girls_8_20_y, Y = out_data), compute_zscore(FMI_boys_8_20_y, Y = out_data)))

}

data_extracted_transf$estim_v5[i] <- (data_extracted_transf$Calc_FMI_zscore[i]*data_extracted_transf$estim_v4[i])/out_data

data_extracted_transf$se_v5[i] <- abs((data_extracted_transf$Calc_FMI_zscore[i]*data_extracted_transf$se_v4[i])/out_data)

data_extracted_transf$ci95_lower_v5[i] <- data_extracted_transf$estim_v5[i]-(1.96*data_extracted_transf$se_v5[i])

data_extracted_transf$ci95_upper_v5[i] <- data_extracted_transf$estim_v5[i]+(1.96*data_extracted_transf$se_v5[i])

} else {

data_extracted_transf$estim_v5[i] <- data_extracted_transf$estim_v4[i]

data_extracted_transf$se_v5[i] <- data_extracted_transf$se_v4[i]

data_extracted_transf$ci95_lower_v5[i] <- data_extracted_transf$ci95_lower_v4[i]

data_extracted_transf$ci95_upper_v5[i] <- data_extracted_transf$ci95_upper_v4[i]

}

}

data_extracted_transf <- mutate(data_extracted_transf, estim = estim_v5, ci95_lower = ci95_lower_v5, ci95_upper = ci95_upper_v5, se = se_v5)

write_tsv(data_extracted_transf, "Frigerio_PFASs_rev_TableS05_transf_estim.txt")

## This table is reported in supplementary material, Table S05

##### end of section 2 ######

######## Section 3: "Performing the meta-analyses"

#### In this section, a function was built to obtain the forest and funnel plots from each combination of exposure and outcome, besides storing the results of the meta-analysis in an object called "[...]_meta"

### creating the function to perform the meta-analyses (forest plots and funnel-plots) and to store the results in an object called "[...]_meta_inv_var" and "[...]_meta_subjects_weighted"

Performing_meta_analysis <- function(DF, num_to_add = "") {

meta_df <- metagen(data = DF,

TE = estim,

studlab = study,

random = TRUE,

fixed = TRUE,

method.tau = "REML",

n.e = n_subj,

lower = ci95_lower,

upper = ci95_upper

)

assign(paste0(num_to_add, deparse(substitute(DF)), "_iv"), meta_df, envir = globalenv())

meta_subj_wgtd <- meta.summaries(d = DF$estim,

se = DF$se,

method="random",

weights=DF$n_subj,

names = DF$study)

assign(paste0(num_to_add, deparse(substitute(DF)), "_sw"), meta_subj_wgtd, envir = globalenv())

forest.meta(meta_df,

fixed = TRUE,

random = TRUE,

leftcols = c("studlab", "age_measurament", "age_outcome", "n_subj"),

leftlabs = c("Study", "Sampling", "Outcome", "Subj"),

col.square = "dark blue",

col.inside = "dark blue",

col.diamond.fixed = "orange",

col.diamond.random = "red",

col.fixed = "orange",

col.random = "red",

smlab = str_replace_all(deparse(substitute(DF)), "_", " ")

)

dev.print(png, paste0("Frigerio_", num_to_add, deparse(substitute(DF)), "_Forest_iv.png"), width=1000, height=460)

tabletext <- cbind(c("Study", DF$study, NA, "Summary"),

c("Sampling", DF$age_measurament, NA, NA),

c("Outcome", DF$age_outcome, NA, NA),

c("Subjects", DF$n_subj, NA, NA),

c("Estimate [95% CI]", paste0(format(round(DF$estim, 3), nsmall = 3), " [", format(round(DF$ci95_lower, 3), nsmall = 3), "; ", format(round(DF$ci95_upper, 3), nsmall = 3), "]"), NA, paste0(format(round(meta_subj_wgtd[["summary"]], 3), nsmall = 3), " [", format(round(meta_subj_wgtd[["summary"]]-(meta_subj_wgtd[["se.summary"]]*1.96), 3), nsmall = 3), "; ", format(round(meta_subj_wgtd[["summary"]]+(meta_subj_wgtd[["se.summary"]]*1.96), 3), nsmall = 3), "]")))

forestplot(labeltext = tabletext,

mean = c(NA, DF$estim, NA, meta_subj_wgtd[["summary"]]),

lower = c(NA, DF$ci95_lower, NA, meta_subj_wgtd[["summary"]]-(meta_subj_wgtd[["se.summary"]]*1.96)),

upper = c(NA, DF$ci95_upper, NA, meta_subj_wgtd[["summary"]]+(meta_subj_wgtd[["se.summary"]]*1.96)),

align = NULL,

is.summary = c(TRUE, rep(FALSE, length(DF$estim)), FALSE, TRUE),

boxsize = c(NA, 4*(meta_subj_wgtd[["weights"]]/sum(meta_subj_wgtd[["weights"]])), NA, 1),

col = meta.colors(box="dark blue", summary="red"))

dev.print(png, paste0("Frigerio_", num_to_add, deparse(substitute(DF)), "_Forest_sw.png"), width=1000, height=460)

Bias_results <- metabias(meta_df, method.bias = "Egger", k.min = length(DF$study))

funnel.meta(meta_df,

col = "blue",

col.random = "red",

col.fixed = "blue"

)

mtext(text = str_replace_all(deparse(substitute(DF)), "_", " "),

side = 3,

line = 0,

adj = 0.8,

cex=1.3,

padj = 3,

font =2 )

mtext(text = paste0("Egger's test",

"\nt = ", round(Bias_results$statistic, digits = 3),

"\ndf = ", Bias_results$DF,

"\np-value = ", round(Bias_results$pval, digits = 3),

"\n\nEstimate\nbias = ", round(Bias_results$estimate["bias"], digits = 3),

"\nse bias = ", round(Bias_results$estimate["se.bias"], digits = 3),

"\nintercept = ", round(Bias_results$estimate["intercept"], digits = 3),

"\nse intercept = ", round(Bias_results$estimate["se.intercept"], digits = 3)),

side = 3,

line = 0,

adj = 0.8,

cex=1.3,

padj = 1.35)

dev.print(png, paste0("Frigerio_", num_to_add, deparse(substitute(DF)), "_Funnel.png"), width=1000, height=800)

}

### apply the function to obtain the results of the meta-analyses:

wanted_order <- c("PFOA_prenatal_BMI_both_sexes", "PFOA_prenatal_BMI_boys", "PFOA_prenatal_BMI_girls", "PFOS_prenatal_BMI_both_sexes", "PFOS_prenatal_BMI_boys", "PFOS_prenatal_BMI_girls", "PFHxS_prenatal_BMI_both_sexes", "PFHxS_prenatal_BMI_boys", "PFHxS_prenatal_BMI_girls", "PFNA_prenatal_BMI_both_sexes", "PFNA_prenatal_BMI_boys", "PFNA_prenatal_BMI_girls", "PFDA_prenatal_BMI_both_sexes", "PFDA_prenatal_BMI_boys", "PFDA_prenatal_BMI_girls", "PFUA_prenatal_BMI_both_sexes", "PFUA_prenatal_BMI_boys", "PFUA_prenatal_BMI_girls", "PFHpA_prenatal_BMI_both_sexes", "PFHpA_prenatal_BMI_boys", "PFHpA_prenatal_BMI_girls", "PFUnDA_prenatal_BMI_both_sexes", "PFUnDA_prenatal_BMI_boys", "PFUnDA_prenatal_BMI_girls", "MeFOSAA_prenatal_BMI_both_sexes", "MeFOSAA_prenatal_BMI_boys", "MeFOSAA_prenatal_BMI_girls", "PFDoA_prenatal_BMI_both_sexes", "PFDoA_prenatal_BMI_boys", "PFDoA_prenatal_BMI_girls", "PFOSA_prenatal_BMI_both_sexes", "PFOSA_prenatal_BMI_boys", "PFOSA_prenatal_BMI_girls", "PFBS_prenatal_BMI_both_sexes", "PFBS_prenatal_BMI_boys", "PFBS_prenatal_BMI_girls",

"PFOA_prenatal_risk_both_sexes", "PFOA_prenatal_risk_boys", "PFOA_prenatal_risk_girls", "PFOS_prenatal_risk_both_sexes", "PFOS_prenatal_risk_boys", "PFOS_prenatal_risk_girls", "PFHxS_prenatal_risk_both_sexes", "PFHxS_prenatal_risk_boys", "PFHxS_prenatal_risk_girls", "PFNA_prenatal_risk_both_sexes", "PFNA_prenatal_risk_boys", "PFNA_prenatal_risk_girls", "PFDA_prenatal_risk_both_sexes", "PFDA_prenatal_risk_boys", "PFDA_prenatal_risk_girls", "PFUA_prenatal_risk_both_sexes", "PFUA_prenatal_risk_boys", "PFUA_prenatal_risk_girls", "PFHpA_prenatal_risk_both_sexes", "PFHpA_prenatal_risk_boys", "PFHpA_prenatal_risk_girls", "PFUnDA_prenatal_risk_both_sexes", "PFUnDA_prenatal_risk_boys", "PFUnDA_prenatal_risk_girls", "MeFOSAA_prenatal_risk_both_sexes", "MeFOSAA_prenatal_risk_boys", "MeFOSAA_prenatal_risk_girls", "PFDoA_prenatal_risk_both_sexes", "PFDoA_prenatal_risk_boys", "PFDoA_prenatal_risk_girls", "PFOSA_prenatal_risk_both_sexes", "PFOSA_prenatal_risk_boys", "PFOSA_prenatal_risk_girls", "PFBS_prenatal_risk_both_sexes", "PFBS_prenatal_risk_boys", "PFBS_prenatal_risk_girls",

"PFOA_prenatal_WC_both_sexes", "PFOA_prenatal_WC_boys", "PFOA_prenatal_WC_girls", "PFOS_prenatal_WC_both_sexes", "PFOS_prenatal_WC_boys", "PFOS_prenatal_WC_girls", "PFHxS_prenatal_WC_both_sexes", "PFHxS_prenatal_WC_boys", "PFHxS_prenatal_WC_girls", "PFNA_prenatal_WC_both_sexes", "PFNA_prenatal_WC_boys", "PFNA_prenatal_WC_girls", "PFDA_prenatal_WC_both_sexes", "PFDA_prenatal_WC_boys", "PFDA_prenatal_WC_girls", "PFUA_prenatal_WC_both_sexes", "PFUA_prenatal_WC_boys", "PFUA_prenatal_WC_girls", "PFHpA_prenatal_WC_both_sexes", "PFHpA_prenatal_WC_boys", "PFHpA_prenatal_WC_girls", "PFUnDA_prenatal_WC_both_sexes", "PFUnDA_prenatal_WC_boys", "PFUnDA_prenatal_WC_girls", "MeFOSAA_prenatal_WC_both_sexes", "MeFOSAA_prenatal_WC_boys", "MeFOSAA_prenatal_WC_girls", "PFDoA_prenatal_WC_both_sexes", "PFDoA_prenatal_WC_boys", "PFDoA_prenatal_WC_girls", "PFOSA_prenatal_WC_both_sexes", "PFOSA_prenatal_WC_boys", "PFOSA_prenatal_WC_girls", "PFBS_prenatal_WC_both_sexes", "PFBS_prenatal_WC_boys", "PFBS_prenatal_WC_girls",

"PFOA_prenatal_DXA_both_sexes", "PFOA_prenatal_DXA_boys", "PFOA_prenatal_DXA_girls", "PFOS_prenatal_DXA_both_sexes", "PFOS_prenatal_DXA_boys", "PFOS_prenatal_DXA_girls", "PFHxS_prenatal_DXA_both_sexes", "PFHxS_prenatal_DXA_boys", "PFHxS_prenatal_DXA_girls", "PFNA_prenatal_DXA_both_sexes", "PFNA_prenatal_DXA_boys", "PFNA_prenatal_DXA_girls", "PFDA_prenatal_DXA_both_sexes", "PFDA_prenatal_DXA_boys", "PFDA_prenatal_DXA_girls", "PFUA_prenatal_DXA_both_sexes", "PFUA_prenatal_DXA_boys", "PFUA_prenatal_DXA_girls", "PFHpA_prenatal_DXA_both_sexes", "PFHpA_prenatal_DXA_boys", "PFHpA_prenatal_DXA_girls", "PFUnDA_prenatal_DXA_both_sexes", "PFUnDA_prenatal_DXA_boys", "PFUnDA_prenatal_DXA_girls", "MeFOSAA_prenatal_DXA_both_sexes", "MeFOSAA_prenatal_DXA_boys", "MeFOSAA_prenatal_DXA_girls", "PFDoA_prenatal_DXA_both_sexes", "PFDoA_prenatal_DXA_boys", "PFDoA_prenatal_DXA_girls", "PFOSA_prenatal_DXA_both_sexes", "PFOSA_prenatal_DXA_boys", "PFOSA_prenatal_DXA_girls", "PFBS_prenatal_DXA_both_sexes", "PFBS_prenatal_DXA_boys", "PFBS_prenatal_DXA_girls",

"PFOA_children_BMI_both_sexes", "PFOA_children_BMI_boys", "PFOA_children_BMI_girls", "PFOS_children_BMI_both_sexes", "PFOS_children_BMI_boys", "PFOS_children_BMI_girls", "PFHxS_children_BMI_both_sexes", "PFHxS_children_BMI_boys", "PFHxS_children_BMI_girls", "PFNA_children_BMI_both_sexes", "PFNA_children_BMI_boys", "PFNA_children_BMI_girls", "PFDA_children_BMI_both_sexes", "PFDA_children_BMI_boys", "PFDA_children_BMI_girls", "PFUA_children_BMI_both_sexes", "PFUA_children_BMI_boys", "PFUA_children_BMI_girls", "PFHpA_children_BMI_both_sexes", "PFHpA_children_BMI_boys", "PFHpA_children_BMI_girls", "PFUnDA_children_BMI_both_sexes", "PFUnDA_children_BMI_boys", "PFUnDA_children_BMI_girls", "MeFOSAA_children_BMI_both_sexes", "MeFOSAA_children_BMI_boys", "MeFOSAA_children_BMI_girls", "PFDoA_children_BMI_both_sexes", "PFDoA_children_BMI_boys", "PFDoA_children_BMI_girls", "PFOSA_children_BMI_both_sexes", "PFOSA_children_BMI_boys", "PFOSA_children_BMI_girls", "PFBS_children_BMI_both_sexes", "PFBS_children_BMI_boys", "PFBS_children_BMI_girls",

"PFOA_children_risk_both_sexes", "PFOA_children_risk_boys", "PFOA_children_risk_girls", "PFOS_children_risk_both_sexes", "PFOS_children_risk_boys", "PFOS_children_risk_girls", "PFHxS_children_risk_both_sexes", "PFHxS_children_risk_boys", "PFHxS_children_risk_girls", "PFNA_children_risk_both_sexes", "PFNA_children_risk_boys", "PFNA_children_risk_girls", "PFDA_children_risk_both_sexes", "PFDA_children_risk_boys", "PFDA_children_risk_girls", "PFUA_children_risk_both_sexes", "PFUA_children_risk_boys", "PFUA_children_risk_girls", "PFHpA_children_risk_both_sexes", "PFHpA_children_risk_boys", "PFHpA_children_risk_girls", "PFUnDA_children_risk_both_sexes", "PFUnDA_children_risk_boys", "PFUnDA_children_risk_girls", "MeFOSAA_children_risk_both_sexes", "MeFOSAA_children_risk_boys", "MeFOSAA_children_risk_girls", "PFDoA_children_risk_both_sexes", "PFDoA_children_risk_boys", "PFDoA_children_risk_girls", "PFOSA_children_risk_both_sexes", "PFOSA_children_risk_boys", "PFOSA_children_risk_girls", "PFBS_children_risk_both_sexes", "PFBS_children_risk_boys", "PFBS_children_risk_girls",

"PFOA_children_WC_both_sexes", "PFOA_children_WC_boys", "PFOA_children_WC_girls", "PFOS_children_WC_both_sexes", "PFOS_children_WC_boys", "PFOS_children_WC_girls", "PFHxS_children_WC_both_sexes", "PFHxS_children_WC_boys", "PFHxS_children_WC_girls", "PFNA_children_WC_both_sexes", "PFNA_children_WC_boys", "PFNA_children_WC_girls", "PFDA_children_WC_both_sexes", "PFDA_children_WC_boys", "PFDA_children_WC_girls", "PFUA_children_WC_both_sexes", "PFUA_children_WC_boys", "PFUA_children_WC_girls", "PFHpA_children_WC_both_sexes", "PFHpA_children_WC_boys", "PFHpA_children_WC_girls", "PFUnDA_children_WC_both_sexes", "PFUnDA_children_WC_boys", "PFUnDA_children_WC_girls", "MeFOSAA_children_WC_both_sexes", "MeFOSAA_children_WC_boys", "MeFOSAA_children_WC_girls", "PFDoA_children_WC_both_sexes", "PFDoA_children_WC_boys", "PFDoA_children_WC_girls", "PFOSA_children_WC_both_sexes", "PFOSA_children_WC_boys", "PFOSA_children_WC_girls", "PFBS_children_WC_both_sexes", "PFBS_children_WC_boys", "PFBS_children_WC_girls",

"PFOA_children_DXA_both_sexes", "PFOA_children_DXA_boys", "PFOA_children_DXA_girls", "PFOS_children_DXA_both_sexes", "PFOS_children_DXA_boys", "PFOS_children_DXA_girls", "PFHxS_children_DXA_both_sexes", "PFHxS_children_DXA_boys", "PFHxS_children_DXA_girls", "PFNA_children_DXA_both_sexes", "PFNA_children_DXA_boys", "PFNA_children_DXA_girls", "PFDA_children_DXA_both_sexes", "PFDA_children_DXA_boys", "PFDA_children_DXA_girls", "PFUA_children_DXA_both_sexes", "PFUA_children_DXA_boys", "PFUA_children_DXA_girls", "PFHpA_children_DXA_both_sexes", "PFHpA_children_DXA_boys", "PFHpA_children_DXA_girls", "PFUnDA_children_DXA_both_sexes", "PFUnDA_children_DXA_boys", "PFUnDA_children_DXA_girls", "MeFOSAA_children_DXA_both_sexes", "MeFOSAA_children_DXA_boys", "MeFOSAA_children_DXA_girls", "PFDoA_children_DXA_both_sexes", "PFDoA_children_DXA_boys", "PFDoA_children_DXA_girls", "PFOSA_children_DXA_both_sexes", "PFOSA_children_DXA_boys", "PFOSA_children_DXA_girls", "PFBS_children_DXA_both_sexes", "PFBS_children_DXA_boys", "PFBS_children_DXA_girls")

METAs <- wanted_order[wanted_order %in% unique(data_extracted_transf$META)]

for (a in METAs) {

number <- which(METAs == a)

number_with_zero <-ifelse(number<100 & number>=10, paste0("0", number), ifelse(number<10, paste0("00", number), paste0(number)))

assign(a, filter(data_extracted_transf, META == a))

eval(parse(text = paste0("Performing_meta_analysis(", a, ", num_to_add = 'r", number_with_zero, "')")))

}

### The Figures obtained are reported in the Supplementary material

####### End of Section 3 #######

##### Section 4: "sensitivity analysis" #######

## In this section, meta-analyses were repeated by excluding one study at a time.

Table_all_summary_estimates <- tibble(META = character(),

studies = character(),

study_removed = character(),

estim_inv_var = double(),

ci95_lower_inv_var = double(),

CI95_upper_inv_var = double(),

I2 = double(),

estim_subj_wgtd = double(),

CI95_lower_subj_wgtd = double(),

CI95_upper_subj_wgtd = double())

for (a in METAs) {

data_extracted_transf_fil <- filter(data_extracted_transf, META == a)

meta_inv_var <- metagen(data = data_extracted_transf_fil,

TE = estim,

studlab = study,

random = TRUE,

fixed = TRUE,

method.tau = "REML",

n.e = n_subj,

lower = ci95_lower,

upper = ci95_upper

)

meta_subj_wgtd <- meta.summaries(d = data_extracted_transf_fil$estim,

se = data_extracted_transf_fil$se,

method="random",

weights=data_extracted_transf_fil$n_subj,

names = data_extracted_transf_fil$study)

new_row <- tibble(META = a,

studies = paste(data_extracted_transf_fil$study, collapse = "; "),

study_removed = "NONE",

estim_inv_var = meta_inv_var[["TE.random"]],

CI95_lower_inv_var = meta_inv_var[["lower.random"]],

CI95_upper_inv_var = meta_inv_var[["upper.random"]],

I2 = meta_inv_var[["I2"]],

estim_subj_wgtd = meta_subj_wgtd[["summary"]],

CI95_lower_subj_wgtd = meta_subj_wgtd[["summary"]]-(meta_subj_wgtd[["se.summary"]]*1.96),

CI95_upper_subj_wgtd = meta_subj_wgtd[["summary"]]+(meta_subj_wgtd[["se.summary"]]*1.96))

Table_all_summary_estimates <- rbind(Table_all_summary_estimates, new_row)

studies_vector <- data_extracted_transf_fil$study

for (s in 1:length(studies_vector)) {

study_to_exclude <- studies_vector[s]

data_extracted_transf_fil_fil <- filter(data_extracted_transf_fil, study != study_to_exclude)

meta_inv_var <- metagen(data = data_extracted_transf_fil_fil,

TE = estim,

studlab = study,

random = TRUE,

fixed = TRUE,

method.tau = "REML",

n.e = n_subj,

lower = ci95_lower,

upper = ci95_upper

)

meta_subj_wgtd <- meta.summaries(d = data_extracted_transf_fil_fil$estim,

se = data_extracted_transf_fil_fil$se,

method="random",

weights=sqrt(data_extracted_transf_fil_fil$n_subj),

names = data_extracted_transf_fil_fil$study)

new_row <- tibble(META = a,

studies = paste(studies_vector[-s], collapse = "; "),

study_removed = study_to_exclude,

estim_inv_var = meta_inv_var[["TE.random"]],

CI95_lower_inv_var = meta_inv_var[["lower.random"]],

CI95_upper_inv_var = meta_inv_var[["upper.random"]],

I2 = meta_inv_var[["I2"]],

estim_subj_wgtd = meta_subj_wgtd[["summary"]],

CI95_lower_subj_wgtd = meta_subj_wgtd[["summary"]]-(meta_subj_wgtd[["se.summary"]]*1.96),

CI95_upper_subj_wgtd = meta_subj_wgtd[["summary"]]+(meta_subj_wgtd[["se.summary"]]*1.96))

Table_all_summary_estimates <- rbind(Table_all_summary_estimates, new_row)

}

}

write_tsv(Table_all_summary_estimates, "Table_all_summary_estimates.txt")

## This Table was further modified in Excel and reported as Table S06

####### End of Section 4 #######

##### Section 5: "Meta-analyses stratified by age" #######

## In this section, meta-analyses were performed by considering separately studies that evaluated the outcomes in children with 3 or less years old to those that evaluated the outcomes in children with more than 3 years

number_up_to_now <- number

for (a in METAs) {

number <- which(METAs == a)+number_up_to_now

number_with_zero <-ifelse(number<100 & number>=10, paste0("0", number), ifelse(number<10, paste0("00", number), paste0(number)))

assign(paste0(a, "_0_3y"), filter(data_extracted_transf, META == a, age_in_years <= 3))

df_0_3 <- filter(data_extracted_transf, META == a, age_in_years <= 3)

assign(paste0(a, "_4_18y"), filter(data_extracted_transf, META == a, age_in_years > 3))

df_4_18 <- filter(data_extracted_transf, META == a, age_in_years > 3)

if(length(df_0_3$META)>=3) {

eval(parse(text = paste0("Performing_meta_analysis(", a, "_0_3y, num_to_add = 'r", number_with_zero, "')")))

}

if(length(df_4_18$META)>=3) {

eval(parse(text = paste0("Performing_meta_analysis(", a, "_4_18y, num_to_add = 'r", number_with_zero, "')")))

}

}

### The Figures obtained are reported in the Supplementary material

####### End of Section 5 #######

##### Section 6: "Meta-analyses stratified by age - sensitivity analyses" #######

## In this section, the same meta-analyses of the previous section were performed by excluding one study at a time.

Table_all_summary_estimates_ages <- tibble(META = character(),

studies = character(),

study_removed = character(),

estim_inv_var = double(),

CI95_lower_inv_var = double(),

CI95_upper_inv_var = double(),

I2 = double(),

estim_subj_wgtd = double(),

CI95_lower_subj_wgtd = double(),

CI95_upper_subj_wgtd = double())

for (a in METAs) {

data_extracted_transf_fil_0_3 <- filter(data_extracted_transf, META == a, age_in_years <= 3)

if(length(data_extracted_transf_fil_0_3$META)>=3) {

meta_inv_var <- metagen(data = data_extracted_transf_fil_0_3,

TE = estim,

studlab = study,

random = TRUE,

fixed = TRUE,

method.tau = "REML",

n.e = n_subj,

lower = ci95_lower,

upper = ci95_upper

)

meta_subj_wgtd <- meta.summaries(d = data_extracted_transf_fil_0_3$estim,

se = data_extracted_transf_fil_0_3$se,

method="random",

weights=data_extracted_transf_fil_0_3$n_subj,

names = data_extracted_transf_fil_0_3$study)

new_row <- tibble(META = paste0(a, "_0_3y"),

studies = paste(data_extracted_transf_fil_0_3$study, collapse = "; "),

study_removed = "NONE",

estim_inv_var = meta_inv_var[["TE.random"]],

CI95_lower_inv_var = meta_inv_var[["lower.random"]],

CI95_upper_inv_var = meta_inv_var[["upper.random"]],

I2 = meta_inv_var[["I2"]],

estim_subj_wgtd = meta_subj_wgtd[["summary"]],

CI95_lower_subj_wgtd = meta_subj_wgtd[["summary"]]-(meta_subj_wgtd[["se.summary"]]*1.96),

CI95_upper_subj_wgtd = meta_subj_wgtd[["summary"]]+(meta_subj_wgtd[["se.summary"]]*1.96))

Table_all_summary_estimates_ages <- rbind(Table_all_summary_estimates_ages, new_row)

studies_vector <- data_extracted_transf_fil_0_3$study

for (s in 1:length(studies_vector)) {

study_to_exclude <- studies_vector[s]

data_extracted_transf_fil_fil <- filter(data_extracted_transf_fil_0_3, study != study_to_exclude)

meta_inv_var <- metagen(data = data_extracted_transf_fil_fil,

TE = estim,

studlab = study,

random = TRUE,

fixed = TRUE,

method.tau = "REML",

n.e = n_subj,

lower = ci95_lower,

upper = ci95_upper

)

meta_subj_wgtd <- meta.summaries(d = data_extracted_transf_fil_fil$estim,

se = data_extracted_transf_fil_fil$se,

method="random",

weights=sqrt(data_extracted_transf_fil_fil$n_subj),

names = data_extracted_transf_fil_fil$study)

new_row <- tibble(META = paste0(a, "_0_3y"),

studies = paste(studies_vector[-s], collapse = "; "),

study_removed = study_to_exclude,

estim_inv_var = meta_inv_var[["TE.random"]],

CI95_lower_inv_var = meta_inv_var[["lower.random"]],

CI95_upper_inv_var = meta_inv_var[["upper.random"]],

I2 = meta_inv_var[["I2"]],

estim_subj_wgtd = meta_subj_wgtd[["summary"]],

CI95_lower_subj_wgtd = meta_subj_wgtd[["summary"]]-(meta_subj_wgtd[["se.summary"]]*1.96),

CI95_upper_subj_wgtd = meta_subj_wgtd[["summary"]]+(meta_subj_wgtd[["se.summary"]]*1.96))

Table_all_summary_estimates_ages <- rbind(Table_all_summary_estimates_ages, new_row)

}

}

data_extracted_transf_fil_4_18 <- filter(data_extracted_transf, META == a, age_in_years > 3)

if(length(data_extracted_transf_fil_4_18$META)>=3) {

meta_inv_var <- metagen(data = data_extracted_transf_fil_4_18,

TE = estim,

studlab = study,

random = TRUE,

fixed = TRUE,

method.tau = "REML",

n.e = n_subj,

lower = ci95_lower,

upper = ci95_upper

)

meta_subj_wgtd <- meta.summaries(d = data_extracted_transf_fil_4_18$estim,

se = data_extracted_transf_fil_4_18$se,

method="random",

weights=data_extracted_transf_fil_4_18$n_subj,

names = data_extracted_transf_fil_4_18$study)

new_row <- tibble(META = paste0(a, "_4_18y"),

studies = paste(data_extracted_transf_fil_4_18$study, collapse = "; "),

study_removed = "NONE",

estim_inv_var = meta_inv_var[["TE.random"]],

CI95_lower_inv_var = meta_inv_var[["lower.random"]],

CI95_upper_inv_var = meta_inv_var[["upper.random"]],

I2 = meta_inv_var[["I2"]],

estim_subj_wgtd = meta_subj_wgtd[["summary"]],

CI95_lower_subj_wgtd = meta_subj_wgtd[["summary"]]-(meta_subj_wgtd[["se.summary"]]*1.96),

CI95_upper_subj_wgtd = meta_subj_wgtd[["summary"]]+(meta_subj_wgtd[["se.summary"]]*1.96))

Table_all_summary_estimates_ages <- rbind(Table_all_summary_estimates_ages, new_row)

studies_vector <- data_extracted_transf_fil_4_18$study

for (s in 1:length(studies_vector)) {

study_to_exclude <- studies_vector[s]

data_extracted_transf_fil_fil <- filter(data_extracted_transf_fil_4_18, study != study_to_exclude)

meta_inv_var <- metagen(data = data_extracted_transf_fil_fil,

TE = estim,

studlab = study,

random = TRUE,

fixed = TRUE,

method.tau = "REML",

n.e = n_subj,

lower = ci95_lower,

upper = ci95_upper

)

meta_subj_wgtd <- meta.summaries(d = data_extracted_transf_fil_fil$estim,

se = data_extracted_transf_fil_fil$se,

method="random",

weights=sqrt(data_extracted_transf_fil_fil$n_subj),

names = data_extracted_transf_fil_fil$study)

new_row <- tibble(META = paste0(a, "_4_18y"),

studies = paste(studies_vector[-s], collapse = "; "),

study_removed = study_to_exclude,

estim_inv_var = meta_inv_var[["TE.random"]],

CI95_lower_inv_var = meta_inv_var[["lower.random"]],

CI95_upper_inv_var = meta_inv_var[["upper.random"]],

I2 = meta_inv_var[["I2"]],

estim_subj_wgtd = meta_subj_wgtd[["summary"]],

CI95_lower_subj_wgtd = meta_subj_wgtd[["summary"]]-(meta_subj_wgtd[["se.summary"]]*1.96),

CI95_upper_subj_wgtd = meta_subj_wgtd[["summary"]]+(meta_subj_wgtd[["se.summary"]]*1.96))

Table_all_summary_estimates_ages <- rbind(Table_all_summary_estimates_ages, new_row)

}

}

}

write_tsv(Table_all_summary_estimates_ages, "Table_all_summary_estimates_ages.txt")

## This Table was further modified in Excel and reported as Table S07

####### End of Section 6 #######

######## Section 7: "Developing the superimposed forest plots"

#### In this section, a script was carefully developed to produce graphs of superimposed forest plots

## considering data from the meta-analyses with inverse variance, all ages combined, and considering stratification by sex.

### creating the database with the information from all meta-analyses with inverse variance:

all_objects <- ls()

iv_metas <- all_objects[grep("_iv", all_objects)]

iv_metas_not_age_stratif <- iv_metas[!grepl("_0_3y", iv_metas)]

iv_metas_not_age_stratif <- iv_metas_not_age_stratif[!grepl("_4_18y", iv_metas_not_age_stratif)]

list_metasiv <- lapply(iv_metas_not_age_stratif, get)

names(list_metasiv) <- iv_metas_not_age_stratif

all_meta_dataiv <- tibble(META = character(),

group = character(),

compound = character(),

outcome = character(),

sex = character(),

study = character(),

estim = double(),

lower = double(),

upper = double(),

w.random = double(),

w.random_perc = double(),

I2 <- double())

for (l in 1:length(list_metasiv)) {

META <- names(list_metasiv[l])

group <- ifelse(grepl("prenatal", META, fixed = TRUE), "prenatal",

ifelse(grepl("children", META, fixed = TRUE), "children", NA))

compound <- ifelse(grepl("PFOA", META, fixed = TRUE), "PFOA",

ifelse(grepl("PFOS", META, fixed = TRUE), "PFOS",

ifelse(grepl("PFNA", META, fixed = TRUE), "PFNA",

ifelse(grepl("PFHxS", META, fixed = TRUE), "PFHxS",

ifelse(grepl("PFDA", META, fixed = TRUE), "PFDA",

ifelse(grepl("PFUA", META, fixed = TRUE), "PFUA",

ifelse(grepl("PFDoA", META, fixed = TRUE), "PFDoA",

ifelse(grepl("PFBS", META, fixed = TRUE), "PFBS", NA))))))))

outcome = ifelse(grepl("BMI", META, fixed = TRUE), "BMI",

ifelse(grepl("risk", META, fixed = TRUE), "risk",

ifelse(grepl("WC", META, fixed = TRUE), "WC",

ifelse(grepl("DXA", META, fixed = TRUE), "DXA", NA))))

sex = ifelse(grepl("both_sexes", META, fixed = TRUE), "both_sexes",

ifelse(grepl("boys", META, fixed = TRUE), "boys",

ifelse(grepl("girls", META, fixed = TRUE), "girls", NA)))

new_rows <- tibble(META = rep(META, length(list_metasiv[[l]][["studlab"]])+1),

group = rep(group, length(list_metasiv[[l]][["studlab"]])+1),

compound = rep(compound, length(list_metasiv[[l]][["studlab"]])+1),

outcome = rep(outcome, length(list_metasiv[[l]][["studlab"]])+1),

sex = rep(sex, length(list_metasiv[[l]][["studlab"]])+1),

study = c(list_metasiv[[l]][["studlab"]], "SUMMARY"),

estim = c(list_metasiv[[l]][["TE"]], list_metasiv[[l]][["TE.random"]]),

lower = c(list_metasiv[[l]][["lower"]], list_metasiv[[l]][["lower.random"]]),

upper = c(list_metasiv[[l]][["upper"]], list_metasiv[[l]][["upper.random"]]),

w.random = c(list_metasiv[[l]][["w.random"]], NA),

w.random_perc = c(((list_metasiv[[l]][["w.random"]]/sum(list_metasiv[[l]][["w.random"]]))*100), NA),

I2 = c(rep(NA, length(list_metasiv[[l]][["studlab"]])), list_metasiv[[l]][["I2"]]))

all_meta_dataiv <- rbind(all_meta_dataiv, new_rows)

}

all_meta_dataiv <- mutate(all_meta_dataiv,

n_subj = NA,

age_measurament = NA,

age_outcome = NA,

cohort_name = NA,

n_both_sexes = NA,

n_boys = NA,

n_girls = NA)

for (m in 1:length(all_meta_dataiv$META)) {

for (e in 1:length(data_extracted_transf$META)) {

if(all_meta_dataiv$study[m] == data_extracted_transf$study[e] &

all_meta_dataiv$group[m] == data_extracted_transf$sampling_time[e] &

all_meta_dataiv$compound[m] == data_extracted_transf$compound[e] &

all_meta_dataiv$outcome[m] == data_extracted_transf$outcome[e] &

all_meta_dataiv$sex[m] == data_extracted_transf$sex[e]) {

all_meta_dataiv$n_subj[m] <- data_extracted_transf$n_subj[e]

all_meta_dataiv$age_measurament[m] <- data_extracted_transf$age_measurament[e]

all_meta_dataiv$age_outcome[m] <- data_extracted_transf$age_outcome[e]

all_meta_dataiv$cohort_name[m] <- data_extracted_transf$cohort_name[e]

both_sexes_index <- which(data_extracted_transf$sex == "both_sexes" &

all_meta_dataiv$study[m] == data_extracted_transf$study &

all_meta_dataiv$group[m] == data_extracted_transf$sampling_time &

all_meta_dataiv$compound[m] == data_extracted_transf$compound &

all_meta_dataiv$outcome[m] == data_extracted_transf$outcome)

boys_index <- which(data_extracted_transf$sex == "boys" &

all_meta_dataiv$study[m] == data_extracted_transf$study &

all_meta_dataiv$group[m] == data_extracted_transf$sampling_time &

all_meta_dataiv$compound[m] == data_extracted_transf$compound &

all_meta_dataiv$outcome[m] == data_extracted_transf$outcome)

girls_index <- which(data_extracted_transf$sex == "girls" &

all_meta_dataiv$study[m] == data_extracted_transf$study &

all_meta_dataiv$group[m] == data_extracted_transf$sampling_time &

all_meta_dataiv$compound[m] == data_extracted_transf$compound &

all_meta_dataiv$outcome[m] == data_extracted_transf$outcome)

if (length(both_sexes_index) == 0) {

all_meta_dataiv$n_both_sexes[m] <- NA

} else if (length(both_sexes_index) == 1) {

all_meta_dataiv$n_both_sexes[m] <- data_extracted_transf$n_subj[both_sexes_index]

} else if (length(both_sexes_index) > 1) {

stop("something wrong")

}

if (length(boys_index) == 0) {

all_meta_dataiv$n_boys[m] <- NA

} else if (length(boys_index) == 1) {

all_meta_dataiv$n_boys[m] <- data_extracted_transf$n_subj[boys_index]

} else if (length(boys_index) > 1) {

stop("something wrong")

}

if (length(girls_index) == 0) {

all_meta_dataiv$n_girls[m] <- NA

} else if (length(girls_index) == 1) {

all_meta_dataiv$n_girls[m] <- data_extracted_transf$n_subj[girls_index]

} else if (length(girls_index) > 1) {

stop("something wrong")

}

}

}

}

all_studies <- unique(data_extracted_transf$study)

wanted_order_studies <- c(all_studies, "SUMMARY")

## formula to build graph for each molecule:

generate_combined_forest <- function(all_meta_data, meta_num, molecules_to_consider, outcome_to_consider, group_to_consider) {

all_meta_dataiv_specif <- filter(all_meta_data, group == group_to_consider, compound == molecules_to_consider, outcome == outcome_to_consider)

specif_studies <- wanted_order_studies[wanted_order_studies %in% unique(all_meta_dataiv_specif$study)]

all_meta_dataiv_specif <- mutate(all_meta_dataiv_specif, num_pos = NA, num_pos_jit = NA)

for (p in 1:length(all_meta_dataiv_specif$META)) {

all_meta_dataiv_specif$num_pos[p] <- which(specif_studies == all_meta_dataiv_specif$study[p])

if(length(unique(all_meta_dataiv_specif$sex))==1) {

all_meta_dataiv_specif$num_pos_jit[p] <- all_meta_dataiv_specif$num_pos[p]

} else if (length(unique(all_meta_dataiv_specif$sex))==2) {

all_meta_dataiv_specif$num_pos_jit[p] <- all_meta_dataiv_specif$num_pos[p]

if (all_meta_dataiv_specif$sex[p] == unique(all_meta_dataiv_specif$sex)[1]) { all_meta_dataiv_specif$num_pos_jit[p] <- all_meta_dataiv_specif$num_pos_jit[p] - 0.1 }

if (all_meta_dataiv_specif$sex[p] == unique(all_meta_dataiv_specif$sex)[2]) { all_meta_dataiv_specif$num_pos_jit[p] <- all_meta_dataiv_specif$num_pos_jit[p] + 0.1 }

} else if (length(unique(all_meta_dataiv_specif$sex))==3) {

all_meta_dataiv_specif$num_pos_jit[p] <- all_meta_dataiv_specif$num_pos[p]

if (all_meta_dataiv_specif$sex[p] == unique(all_meta_dataiv_specif$sex)[1]) { all_meta_dataiv_specif$num_pos_jit[p] <- all_meta_dataiv_specif$num_pos_jit[p] - 0.15 }

if (all_meta_dataiv_specif$sex[p] == unique(all_meta_dataiv_specif$sex)[2]) { all_meta_dataiv_specif$num_pos_jit[p] <- all_meta_dataiv_specif$num_pos_jit[p] }

if (all_meta_dataiv_specif$sex[p] == unique(all_meta_dataiv_specif$sex)[3]) { all_meta_dataiv_specif$num_pos_jit[p] <- all_meta_dataiv_specif$num_pos_jit[p] + 0.15 }

} else if (length(unique(all_meta_dataiv_specif$sex))>3) {

stop("something wrong")

}

if (all_meta_dataiv_specif$study[p] == "SUMMARY" & all_meta_dataiv_specif$sex[p] == "both_sexes") {

all_meta_dataiv_specif$num_pos_jit[p] <- all_meta_dataiv_specif$num_pos[p]

} else if (all_meta_dataiv_specif$study[p] == "SUMMARY" & all_meta_dataiv_specif$sex[p] == "boys") {

all_meta_dataiv_specif$num_pos_jit[p] <- all_meta_dataiv_specif$num_pos[p] + 1

} else if (all_meta_dataiv_specif$study[p] == "SUMMARY" & all_meta_dataiv_specif$sex[p] == "girls") {

all_meta_dataiv_specif$num_pos_jit[p] <- all_meta_dataiv_specif$num_pos[p] + 2

}

}

all_meta_dataiv_specif$num_pos <- all_meta_dataiv_specif$num_pos*(-1)

all_meta_dataiv_specif$num_pos_jit <- all_meta_dataiv_specif$num_pos_jit*(-1)

## preparing the data to show the diamonds of the pooled estimates in the graph for prenatal exposure:

diamond_y <- c(-0.4, 0, 0.4, 0)

index_summary_both_sex <- which(all_meta_dataiv_specif$sex == "both_sexes" & all_meta_dataiv_specif$study == "SUMMARY")

index_summary_boys <- which(all_meta_dataiv_specif$sex == "boys" & all_meta_dataiv_specif$study == "SUMMARY")

index_summary_girls <- which(all_meta_dataiv_specif$sex == "girls" & all_meta_dataiv_specif$study == "SUMMARY")

if (length(index_summary_both_sex) != 1) {stop("Something wrong")}

if (length(index_summary_boys) == 0 | length(index_summary_girls) == 0) {

diamonds_specif <- tibble(

TE = c(all_meta_dataiv_specif$estim[index_summary_both_sex], all_meta_dataiv_specif$upper[index_summary_both_sex], all_meta_dataiv_specif$estim[index_summary_both_sex], all_meta_dataiv_specif$lower[index_summary_both_sex]),

sex = c(rep("both_sexes", 4)),

pos_diam = c(all_meta_dataiv_specif$num_pos_jit[index_summary_both_sex]-diamond_y[1],

all_meta_dataiv_specif$num_pos_jit[index_summary_both_sex]-diamond_y[2],

all_meta_dataiv_specif$num_pos_jit[index_summary_both_sex]-diamond_y[3],

all_meta_dataiv_specif$num_pos_jit[index_summary_both_sex]-diamond_y[4]

)

)

dat_text_specif <- tibble(sex = c("both_sexes"),

pos_x = rep(0.09*(max(all_meta_dataiv_specif$upper)-min(all_meta_dataiv_specif$lower)), 1),

pos_y = c(all_meta_dataiv_specif$num_pos_jit[index_summary_both_sex]),

text = c(paste0(formatC(all_meta_dataiv_specif$estim[index_summary_both_sex], format = "f", digits = 4), " [", formatC(all_meta_dataiv_specif$lower[index_summary_both_sex], format = "f", digits = 4), "; ", formatC(all_meta_dataiv_specif$upper[index_summary_both_sex], format = "f", digits = 4), "]")))

dat_textI2_specif <- tibble(sex = c("both_sexes"),

pos_x = rep(min(all_meta_dataiv_specif$lower), 1),

pos_y = c(all_meta_dataiv_specif$num_pos_jit[index_summary_both_sex]),

text = c(paste0("I^2", " = ", formatC(all_meta_dataiv_specif$I2[index_summary_both_sex], format = "f", digits = 2))

))

} else {

diamonds_specif <- tibble(

TE = c(all_meta_dataiv_specif$estim[index_summary_both_sex], all_meta_dataiv_specif$upper[index_summary_both_sex], all_meta_dataiv_specif$estim[index_summary_both_sex], all_meta_dataiv_specif$lower[index_summary_both_sex],

all_meta_dataiv_specif$estim[index_summary_boys], all_meta_dataiv_specif$upper[index_summary_boys], all_meta_dataiv_specif$estim[index_summary_boys], all_meta_dataiv_specif$lower[index_summary_boys],

all_meta_dataiv_specif$estim[index_summary_girls], all_meta_dataiv_specif$upper[index_summary_girls], all_meta_dataiv_specif$estim[index_summary_girls], all_meta_dataiv_specif$lower[index_summary_girls]),

sex = c(rep("both_sexes", 4), rep("boys", 4), rep("girls", 4)),

pos_diam = c(all_meta_dataiv_specif$num_pos_jit[index_summary_both_sex]-diamond_y[1],

all_meta_dataiv_specif$num_pos_jit[index_summary_both_sex]-diamond_y[2],

all_meta_dataiv_specif$num_pos_jit[index_summary_both_sex]-diamond_y[3],

all_meta_dataiv_specif$num_pos_jit[index_summary_both_sex]-diamond_y[4],

all_meta_dataiv_specif$num_pos_jit[index_summary_boys]-diamond_y[1],

all_meta_dataiv_specif$num_pos_jit[index_summary_boys]-diamond_y[2],

all_meta_dataiv_specif$num_pos_jit[index_summary_boys]-diamond_y[3],

all_meta_dataiv_specif$num_pos_jit[index_summary_boys]-diamond_y[4],

all_meta_dataiv_specif$num_pos_jit[index_summary_girls]-diamond_y[1],

all_meta_dataiv_specif$num_pos_jit[index_summary_girls]-diamond_y[2],

all_meta_dataiv_specif$num_pos_jit[index_summary_girls]-diamond_y[3],

all_meta_dataiv_specif$num_pos_jit[index_summary_girls]-diamond_y[4])

)

dat_text_specif <- tibble(sex = c("both_sexes", "boys", "girls"),

pos_x = rep(0.09*(max(all_meta_dataiv_specif$upper)-min(all_meta_dataiv_specif$lower)), 3),

pos_y = c(all_meta_dataiv_specif$num_pos_jit[index_summary_both_sex], all_meta_dataiv_specif$num_pos_jit[index_summary_boys], all_meta_dataiv_specif$num_pos_jit[index_summary_girls]),

text = c(paste0(formatC(all_meta_dataiv_specif$estim[index_summary_both_sex], format = "f", digits = 4), " [", formatC(all_meta_dataiv_specif$lower[index_summary_both_sex], format = "f", digits = 4), "; ", formatC(all_meta_dataiv_specif$upper[index_summary_both_sex], format = "f", digits = 4), "]"),

paste0(formatC(all_meta_dataiv_specif$estim[index_summary_boys], format = "f", digits = 4), " [", formatC(all_meta_dataiv_specif$lower[index_summary_boys], format = "f", digits = 4), "; ", formatC(all_meta_dataiv_specif$upper[index_summary_boys], format = "f", digits = 4), "]"),

paste0(formatC(all_meta_dataiv_specif$estim[index_summary_girls], format = "f", digits = 4), " [", formatC(all_meta_dataiv_specif$lower[index_summary_girls], format = "f", digits = 4), "; ", formatC(all_meta_dataiv_specif$upper[index_summary_girls], format = "f", digits = 4), "]")))

dat_textI2_specif <- tibble(sex = c("both_sexes", "boys", "girls"),

pos_x = rep(min(all_meta_dataiv_specif$lower), 3),

pos_y = c(all_meta_dataiv_specif$num_pos_jit[index_summary_both_sex], all_meta_dataiv_specif$num_pos_jit[index_summary_boys], all_meta_dataiv_specif$num_pos_jit[index_summary_girls]),

text = c(paste0("I^2", " = ", formatC(all_meta_dataiv_specif$I2[index_summary_both_sex], format = "f", digits = 2)),

paste0("I^2 = ", formatC(all_meta_dataiv_specif$I2[index_summary_boys], format = "f", digits = 2)),

paste0("I^2 = ", formatC(all_meta_dataiv_specif$I2[index_summary_girls], format = "f", digits = 2))))

}

# attaching together the plot with the Table of information

graph_forest <- ggplot(data = all_meta_dataiv_specif, aes(x = estim, y = num_pos_jit)) +

geom_point(aes(colour = sex, fill = sex, size = w.random_perc), shape = 22, alpha = 0.7) +

scale_size(guide = "none") +

geom_segment(aes(y=num_pos_jit, yend = num_pos_jit, x = lower, xend = upper, colour = sex)) +

scale_fill_manual(values = c("both_sexes" = "green", "boys" = "blue", "girls" = "red")) +

scale_colour_manual(values = c("both_sexes" = "green", "boys" = "blue", "girls" = "red")) +

scale_x_continuous(position = "top") +

geom_vline(xintercept = 0, alpha = 0.6) +

geom_polygon(data = diamonds_specif, aes(x = TE, y = pos_diam, colour = sex, fill = sex), alpha=0.7) +

geom_text(data = dat_text_specif, aes(x = pos_x, y = pos_y, label = text), hjust = "left", size = 5) +

geom_text(data = dat_textI2_specif, aes(x = pos_x, y = pos_y, label = text), hjust = "left", size = 5) +

theme_classic() +

theme(axis.title.x =element_blank(),

axis.text.x = element_text(size=13, face="bold"),

axis.line.y=element_blank(),

axis.title.y=element_blank(),

axis.text.y=element_blank(),

axis.ticks.y=element_blank())

if(abs(min(all_meta_dataiv_specif$lower))/abs(max(all_meta_dataiv_specif$upper))>2 & min(all_meta_dataiv_specif$lower)>0.9) {

graph_forest <- graph_forest + coord_cartesian(xlim = c(-0.3, 0.3))

} else if (abs(min(all_meta_dataiv_specif$lower))/abs(max(all_meta_dataiv_specif$upper))>2 & min(all_meta_dataiv_specif$lower)<0.9) {

graph_forest <- graph_forest + coord_cartesian(xlim = c(-0.9, 0.9))

}

Table_to_add1 <- select(all_meta_dataiv_specif, study, num_pos) %>%

group_by(study) %>%

slice(1) %>%

ungroup %>%

arrange(match(study, specif_studies))

Table_to_add1[nrow(Table_to_add1) + 1 , "num_pos"] <- -(nrow(Table_to_add1))-1

Table_to_add1[nrow(Table_to_add1) + 1 , "num_pos"] <- -(nrow(Table_to_add1))-1

Table_to_add1 <- gather(Table_to_add1, Stat, Value, study)

graph_table1 <- ggplot(data = Table_to_add1, aes(x = Stat, y = num_pos, label = Value)) +

geom_text(size = 5) +

scale_x_discrete(position = "top", labels = c("Study")) +

labs(y = NULL, x = NULL) +

theme_classic() +

theme(axis.title.x =element_blank(),

axis.text.x = element_text(size=14, face="bold"),

axis.line.y=element_blank(),

axis.title.y=element_blank(),

axis.text.y=element_blank(),

axis.ticks.y=element_blank())

Table_to_add2 <- select(all_meta_dataiv_specif, study, n_both_sexes, n_boys, n_girls, num_pos) %>%

group_by(study) %>%

slice(1) %>%

ungroup %>%

arrange(match(study, specif_studies))

Table_to_add2[nrow(Table_to_add2) + 1 , "num_pos"] <- -(nrow(Table_to_add2))-1

Table_to_add2[nrow(Table_to_add2) + 1 , "num_pos"] <- -(nrow(Table_to_add2))-1

Table_to_add2 <- select(Table_to_add2, -study) %>%

gather(Stat, Value, n_both_sexes, n_boys, n_girls)

graph_table2 <- ggplot(data = Table_to_add2, aes(x = Stat, y = num_pos, label = Value)) +

geom_text(size = 5) +

scale_x_discrete(position = "top", labels = c("both", "boys", "girls")) +

labs(y = NULL, x = NULL) +

theme_classic() +

theme(axis.title.x =element_blank(),

axis.text.x = element_text(size=14, face="bold"),

axis.line.y=element_blank(),

axis.title.y=element_blank(),

axis.text.y=element_blank(),

axis.ticks.y=element_blank())

grid.arrange(graph_table1, graph_table2, graph_forest ,ncol=3, widths = c(26/100,14/100, 60/100),

top = textGrob(paste0(group_to_consider, " exposure to ", molecules_to_consider, " and association with ", ifelse(outcome_to_consider=="risk", "risk of overweight", outcome_to_consider)), gp=gpar(fontsize=20, face="bold")))

dev.print(png, paste0("Frigerio_", meta_num, molecules_to_consider, group_to_consider, outcome_to_consider, ".png"), width=1000, height=abs(min(all_meta_dataiv_specif$num_pos_jit))*18+85)

}

## formula to create a graph combining two molecules:

generate_combined_forest_by2 <- function(all_meta_data, meta_num, molecules_to_consider, outcome_to_consider, group_to_consider) {

all_meta_dataiv_specif <- filter(all_meta_data, group == group_to_consider, compound %in% molecules_to_consider, outcome == outcome_to_consider)

specif_studies <- wanted_order_studies[wanted_order_studies %in% unique(all_meta_dataiv_specif$study)]

all_meta_dataiv_specif <- mutate(all_meta_dataiv_specif, num_pos = NA, num_pos_jit = NA)

for (p in 1:length(all_meta_dataiv_specif$META)) {

all_meta_dataiv_specif$num_pos[p] <- which(specif_studies == all_meta_dataiv_specif$study[p])

if(length(unique(all_meta_dataiv_specif$sex))==1) {

all_meta_dataiv_specif$num_pos_jit[p] <- all_meta_dataiv_specif$num_pos[p]

} else if (length(unique(all_meta_dataiv_specif$sex))==2) {

all_meta_dataiv_specif$num_pos_jit[p] <- all_meta_dataiv_specif$num_pos[p]

if (all_meta_dataiv_specif$sex[p] == unique(all_meta_dataiv_specif$sex)[1]) { all_meta_dataiv_specif$num_pos_jit[p] <- all_meta_dataiv_specif$num_pos_jit[p] - 0.1 }

if (all_meta_dataiv_specif$sex[p] == unique(all_meta_dataiv_specif$sex)[2]) { all_meta_dataiv_specif$num_pos_jit[p] <- all_meta_dataiv_specif$num_pos_jit[p] + 0.1 }

} else if (length(unique(all_meta_dataiv_specif$sex))==3) {

all_meta_dataiv_specif$num_pos_jit[p] <- all_meta_dataiv_specif$num_pos[p]

if (all_meta_dataiv_specif$sex[p] == unique(all_meta_dataiv_specif$sex)[1]) { all_meta_dataiv_specif$num_pos_jit[p] <- all_meta_dataiv_specif$num_pos_jit[p] - 0.15 }

if (all_meta_dataiv_specif$sex[p] == unique(all_meta_dataiv_specif$sex)[2]) { all_meta_dataiv_specif$num_pos_jit[p] <- all_meta_dataiv_specif$num_pos_jit[p] }

if (all_meta_dataiv_specif$sex[p] == unique(all_meta_dataiv_specif$sex)[3]) { all_meta_dataiv_specif$num_pos_jit[p] <- all_meta_dataiv_specif$num_pos_jit[p] + 0.15 }

} else if (length(unique(all_meta_dataiv_specif$sex))>3) {

stop("something wrong")

}

if (all_meta_dataiv_specif$study[p] == "SUMMARY" & all_meta_dataiv_specif$sex[p] == "both_sexes") {

all_meta_dataiv_specif$num_pos_jit[p] <- all_meta_dataiv_specif$num_pos[p]

} else if (all_meta_dataiv_specif$study[p] == "SUMMARY" & all_meta_dataiv_specif$sex[p] == "boys") {

all_meta_dataiv_specif$num_pos_jit[p] <- all_meta_dataiv_specif$num_pos[p] + 1

} else if (all_meta_dataiv_specif$study[p] == "SUMMARY" & all_meta_dataiv_specif$sex[p] == "girls") {

all_meta_dataiv_specif$num_pos_jit[p] <- all_meta_dataiv_specif$num_pos[p] + 2

}

}

all_meta_dataiv_specif$num_pos <- all_meta_dataiv_specif$num_pos*(-1)

all_meta_dataiv_specif$num_pos_jit <- all_meta_dataiv_specif$num_pos_jit*(-1)

Ylimit <- c(min(all_meta_dataiv_specif$num_pos_jit)-0.5, max(all_meta_dataiv_specif$num_pos_jit))

### for the first graph:

all_meta_dataiv_specif1 <- filter(all_meta_dataiv_specif, compound == molecules_to_consider[1])

diamond_y <- c(-0.4, 0, 0.4, 0)

index_summary_both_sex1 <- which(all_meta_dataiv_specif1$sex == "both_sexes" & all_meta_dataiv_specif1$study == "SUMMARY")

index_summary_boys1 <- which(all_meta_dataiv_specif1$sex == "boys" & all_meta_dataiv_specif1$study == "SUMMARY")

index_summary_girls1 <- which(all_meta_dataiv_specif1$sex == "girls" & all_meta_dataiv_specif1$study == "SUMMARY")

if (length(index_summary_both_sex1) != 1) {stop("Something wrong")}

if (length(index_summary_boys1) == 0 | length(index_summary_girls1) == 0) {

diamonds_specif1 <- tibble(

TE = c(all_meta_dataiv_specif1$estim[index_summary_both_sex1], all_meta_dataiv_specif1$upper[index_summary_both_sex1], all_meta_dataiv_specif1$estim[index_summary_both_sex1], all_meta_dataiv_specif1$lower[index_summary_both_sex1]),

sex = c(rep("both_sexes", 4)),

pos_diam = c(all_meta_dataiv_specif1$num_pos_jit[index_summary_both_sex1]-diamond_y[1],

all_meta_dataiv_specif1$num_pos_jit[index_summary_both_sex1]-diamond_y[2],

all_meta_dataiv_specif1$num_pos_jit[index_summary_both_sex1]-diamond_y[3],

all_meta_dataiv_specif1$num_pos_jit[index_summary_both_sex1]-diamond_y[4]

)

)

dat_text_specif1 <- tibble(sex = c("both_sexes"),

pos_x = rep(0.09*(max(all_meta_dataiv_specif1$upper)-min(all_meta_dataiv_specif1$lower)), 1),

pos_y = c(all_meta_dataiv_specif1$num_pos_jit[index_summary_both_sex1]),

text = c(paste0(formatC(all_meta_dataiv_specif1$estim[index_summary_both_sex1], format = "f", digits = 4), " [", formatC(all_meta_dataiv_specif1$lower[index_summary_both_sex1], format = "f", digits = 4), "; ", formatC(all_meta_dataiv_specif1$upper[index_summary_both_sex1], format = "f", digits = 4), "]")))

dat_textI2_specif1 <- tibble(sex = c("both_sexes"),

pos_x = rep(min(all_meta_dataiv_specif1$lower), 1),

pos_y = c(all_meta_dataiv_specif1$num_pos_jit[index_summary_both_sex1]),

text = c(paste0("I^2", " = ", formatC(all_meta_dataiv_specif1$I2[index_summary_both_sex1], format = "f", digits = 2))

))

} else {

diamonds_specif1 <- tibble(

TE = c(all_meta_dataiv_specif1$estim[index_summary_both_sex1], all_meta_dataiv_specif1$upper[index_summary_both_sex1], all_meta_dataiv_specif1$estim[index_summary_both_sex1], all_meta_dataiv_specif1$lower[index_summary_both_sex1],

all_meta_dataiv_specif1$estim[index_summary_boys1], all_meta_dataiv_specif1$upper[index_summary_boys1], all_meta_dataiv_specif1$estim[index_summary_boys1], all_meta_dataiv_specif1$lower[index_summary_boys1],

all_meta_dataiv_specif1$estim[index_summary_girls1], all_meta_dataiv_specif1$upper[index_summary_girls1], all_meta_dataiv_specif1$estim[index_summary_girls1], all_meta_dataiv_specif1$lower[index_summary_girls1]),

sex = c(rep("both_sexes", 4), rep("boys", 4), rep("girls", 4)),

pos_diam = c(all_meta_dataiv_specif1$num_pos_jit[index_summary_both_sex1]-diamond_y[1],

all_meta_dataiv_specif1$num_pos_jit[index_summary_both_sex1]-diamond_y[2],

all_meta_dataiv_specif1$num_pos_jit[index_summary_both_sex1]-diamond_y[3],

all_meta_dataiv_specif1$num_pos_jit[index_summary_both_sex1]-diamond_y[4],

all_meta_dataiv_specif1$num_pos_jit[index_summary_boys1]-diamond_y[1],

all_meta_dataiv_specif1$num_pos_jit[index_summary_boys1]-diamond_y[2],

all_meta_dataiv_specif1$num_pos_jit[index_summary_boys1]-diamond_y[3],

all_meta_dataiv_specif1$num_pos_jit[index_summary_boys1]-diamond_y[4],

all_meta_dataiv_specif1$num_pos_jit[index_summary_girls1]-diamond_y[1],

all_meta_dataiv_specif1$num_pos_jit[index_summary_girls1]-diamond_y[2],

all_meta_dataiv_specif1$num_pos_jit[index_summary_girls1]-diamond_y[3],

all_meta_dataiv_specif1$num_pos_jit[index_summary_girls1]-diamond_y[4])

)

dat_text_specif1 <- tibble(sex = c("both_sexes", "boys", "girls"),

pos_x = rep(0.09*(max(all_meta_dataiv_specif1$upper)-min(all_meta_dataiv_specif1$lower)), 3),

pos_y = c(all_meta_dataiv_specif1$num_pos_jit[index_summary_both_sex1], all_meta_dataiv_specif1$num_pos_jit[index_summary_boys1], all_meta_dataiv_specif1$num_pos_jit[index_summary_girls1]),

text = c(paste0(formatC(all_meta_dataiv_specif1$estim[index_summary_both_sex1], format = "f", digits = 4), " [", formatC(all_meta_dataiv_specif1$lower[index_summary_both_sex1], format = "f", digits = 4), "; ", formatC(all_meta_dataiv_specif1$upper[index_summary_both_sex1], format = "f", digits = 4), "]"),

paste0(formatC(all_meta_dataiv_specif1$estim[index_summary_boys1], format = "f", digits = 4), " [", formatC(all_meta_dataiv_specif1$lower[index_summary_boys1], format = "f", digits = 4), "; ", formatC(all_meta_dataiv_specif1$upper[index_summary_boys1], format = "f", digits = 4), "]"),

paste0(formatC(all_meta_dataiv_specif1$estim[index_summary_girls1], format = "f", digits = 4), " [", formatC(all_meta_dataiv_specif1$lower[index_summary_girls1], format = "f", digits = 4), "; ", formatC(all_meta_dataiv_specif1$upper[index_summary_girls1], format = "f", digits = 4), "]")))

dat_textI2_specif1 <- tibble(sex = c("both_sexes", "boys", "girls"),

pos_x = rep(min(all_meta_dataiv_specif1$lower), 3),

pos_y = c(all_meta_dataiv_specif1$num_pos_jit[index_summary_both_sex1], all_meta_dataiv_specif1$num_pos_jit[index_summary_boys1], all_meta_dataiv_specif1$num_pos_jit[index_summary_girls1]),

text = c(paste0("I^2", " = ", formatC(all_meta_dataiv_specif1$I2[index_summary_both_sex1], format = "f", digits = 2)),

paste0("I^2 = ", formatC(all_meta_dataiv_specif1$I2[index_summary_boys1], format = "f", digits = 2)),

paste0("I^2 = ", formatC(all_meta_dataiv_specif1$I2[index_summary_girls1], format = "f", digits = 2))))

}

graph_forest1 <- ggplot(data = all_meta_dataiv_specif1, aes(x = estim, y = num_pos_jit)) +

geom_point(aes(colour = sex, fill = sex, size = w.random_perc), shape = 22, alpha = 0.7) +

scale_size(guide = "none") +

geom_segment(aes(y=num_pos_jit, yend = num_pos_jit, x = lower, xend = upper, colour = sex)) +

scale_fill_manual(values = c("both_sexes" = "green", "boys" = "blue", "girls" = "red")) +

scale_colour_manual(values = c("both_sexes" = "green", "boys" = "blue", "girls" = "red")) +

scale_x_continuous(position = "top") +

geom_vline(xintercept = 0, alpha = 0.6) +

geom_polygon(data = diamonds_specif1, aes(x = TE, y = pos_diam, colour = sex, fill = sex), alpha=0.7) +

geom_text(data = dat_text_specif1, aes(x = pos_x, y = pos_y, label = text), hjust = "left", size = 5) +

geom_text(data = dat_textI2_specif1, aes(x = pos_x, y = pos_y, label = text), hjust = "left", size = 5) +

coord_cartesian(ylim = Ylimit) +

theme_classic() +

theme(axis.title.x =element_blank(),

axis.text.x = element_text(size=13, face="bold"),

axis.line.y=element_blank(),

axis.title.y=element_blank(),

axis.text.y=element_blank(),

axis.ticks.y=element_blank(),

legend.position = "none")

if(abs(min(all_meta_dataiv_specif1$lower))/abs(max(all_meta_dataiv_specif1$upper))>2 & min(all_meta_dataiv_specif1$lower)>0.9) {

graph_forest1 <- graph_forest1 + coord_cartesian(xlim = c(-0.3, 0.3))

} else if (abs(min(all_meta_dataiv_specif1$lower))/abs(max(all_meta_dataiv_specif1$upper))>2 & min(all_meta_dataiv_specif1$lower)<0.9) {

graph_forest1 <- graph_forest1 + coord_cartesian(xlim = c(-0.9, 0.9))

}

### for the second graph:

all_meta_dataiv_specif2 <- filter(all_meta_dataiv_specif, compound == molecules_to_consider[2])

diamond_y <- c(-0.4, 0, 0.4, 0)

index_summary_both_sex2 <- which(all_meta_dataiv_specif2$sex == "both_sexes" & all_meta_dataiv_specif2$study == "SUMMARY")

index_summary_boys2 <- which(all_meta_dataiv_specif2$sex == "boys" & all_meta_dataiv_specif2$study == "SUMMARY")

index_summary_girls2 <- which(all_meta_dataiv_specif2$sex == "girls" & all_meta_dataiv_specif2$study == "SUMMARY")

if (length(index_summary_both_sex2) != 1) {stop("Something wrong")}

if (length(index_summary_boys2) == 0 | length(index_summary_girls2) == 0) {

diamonds_specif2 <- tibble(

TE = c(all_meta_dataiv_specif2$estim[index_summary_both_sex2], all_meta_dataiv_specif2$upper[index_summary_both_sex2], all_meta_dataiv_specif2$estim[index_summary_both_sex2], all_meta_dataiv_specif2$lower[index_summary_both_sex2]),

sex = c(rep("both_sexes", 4)),

pos_diam = c(all_meta_dataiv_specif2$num_pos_jit[index_summary_both_sex2]-diamond_y[1],

all_meta_dataiv_specif2$num_pos_jit[index_summary_both_sex2]-diamond_y[2],

all_meta_dataiv_specif2$num_pos_jit[index_summary_both_sex2]-diamond_y[3],

all_meta_dataiv_specif2$num_pos_jit[index_summary_both_sex2]-diamond_y[4]

)

)

dat_text_specif2 <- tibble(sex = c("both_sexes"),

pos_x = rep(0.09*(max(all_meta_dataiv_specif2$upper)-min(all_meta_dataiv_specif2$lower)), 1),

pos_y = c(all_meta_dataiv_specif2$num_pos_jit[index_summary_both_sex2]),

text = c(paste0(formatC(all_meta_dataiv_specif2$estim[index_summary_both_sex2], format = "f", digits = 4), " [", formatC(all_meta_dataiv_specif2$lower[index_summary_both_sex2], format = "f", digits = 4), "; ", formatC(all_meta_dataiv_specif2$upper[index_summary_both_sex2], format = "f", digits = 4), "]")))

dat_textI2_specif2 <- tibble(sex = c("both_sexes"),

pos_x = rep(min(all_meta_dataiv_specif2$lower), 1),

pos_y = c(all_meta_dataiv_specif2$num_pos_jit[index_summary_both_sex2]),

text = c(paste0("I^2", " = ", formatC(all_meta_dataiv_specif2$I2[index_summary_both_sex2], format = "f", digits = 2))

))

} else {

diamonds_specif2 <- tibble(

TE = c(all_meta_dataiv_specif2$estim[index_summary_both_sex2], all_meta_dataiv_specif2$upper[index_summary_both_sex2], all_meta_dataiv_specif2$estim[index_summary_both_sex2], all_meta_dataiv_specif2$lower[index_summary_both_sex2],

all_meta_dataiv_specif2$estim[index_summary_boys2], all_meta_dataiv_specif2$upper[index_summary_boys2], all_meta_dataiv_specif2$estim[index_summary_boys2], all_meta_dataiv_specif2$lower[index_summary_boys2],

all_meta_dataiv_specif2$estim[index_summary_girls2], all_meta_dataiv_specif2$upper[index_summary_girls2], all_meta_dataiv_specif2$estim[index_summary_girls2], all_meta_dataiv_specif2$lower[index_summary_girls2]),

sex = c(rep("both_sexes", 4), rep("boys", 4), rep("girls", 4)),

pos_diam = c(all_meta_dataiv_specif2$num_pos_jit[index_summary_both_sex2]-diamond_y[1],

all_meta_dataiv_specif2$num_pos_jit[index_summary_both_sex2]-diamond_y[2],

all_meta_dataiv_specif2$num_pos_jit[index_summary_both_sex2]-diamond_y[3],

all_meta_dataiv_specif2$num_pos_jit[index_summary_both_sex2]-diamond_y[4],

all_meta_dataiv_specif2$num_pos_jit[index_summary_boys2]-diamond_y[1],

all_meta_dataiv_specif2$num_pos_jit[index_summary_boys2]-diamond_y[2],

all_meta_dataiv_specif2$num_pos_jit[index_summary_boys2]-diamond_y[3],

all_meta_dataiv_specif2$num_pos_jit[index_summary_boys2]-diamond_y[4],

all_meta_dataiv_specif2$num_pos_jit[index_summary_girls2]-diamond_y[1],

all_meta_dataiv_specif2$num_pos_jit[index_summary_girls2]-diamond_y[2],

all_meta_dataiv_specif2$num_pos_jit[index_summary_girls2]-diamond_y[3],

all_meta_dataiv_specif2$num_pos_jit[index_summary_girls2]-diamond_y[4])

)

dat_text_specif2 <- tibble(sex = c("both_sexes", "boys", "girls"),

pos_x = rep(0.09*(max(all_meta_dataiv_specif2$upper)-min(all_meta_dataiv_specif2$lower)), 3),

pos_y = c(all_meta_dataiv_specif2$num_pos_jit[index_summary_both_sex2], all_meta_dataiv_specif2$num_pos_jit[index_summary_boys2], all_meta_dataiv_specif2$num_pos_jit[index_summary_girls2]),

text = c(paste0(formatC(all_meta_dataiv_specif2$estim[index_summary_both_sex2], format = "f", digits = 4), " [", formatC(all_meta_dataiv_specif2$lower[index_summary_both_sex2], format = "f", digits = 4), "; ", formatC(all_meta_dataiv_specif2$upper[index_summary_both_sex2], format = "f", digits = 4), "]"),

paste0(formatC(all_meta_dataiv_specif2$estim[index_summary_boys2], format = "f", digits = 4), " [", formatC(all_meta_dataiv_specif2$lower[index_summary_boys2], format = "f", digits = 4), "; ", formatC(all_meta_dataiv_specif2$upper[index_summary_boys2], format = "f", digits = 4), "]"),

paste0(formatC(all_meta_dataiv_specif2$estim[index_summary_girls2], format = "f", digits = 4), " [", formatC(all_meta_dataiv_specif2$lower[index_summary_girls2], format = "f", digits = 4), "; ", formatC(all_meta_dataiv_specif2$upper[index_summary_girls2], format = "f", digits = 4), "]")))

dat_textI2_specif2 <- tibble(sex = c("both_sexes", "boys", "girls"),

pos_x = rep(min(all_meta_dataiv_specif2$lower), 3),

pos_y = c(all_meta_dataiv_specif2$num_pos_jit[index_summary_both_sex2], all_meta_dataiv_specif2$num_pos_jit[index_summary_boys2], all_meta_dataiv_specif2$num_pos_jit[index_summary_girls2]),

text = c(paste0("I^2", " = ", formatC(all_meta_dataiv_specif2$I2[index_summary_both_sex2], format = "f", digits = 2)),

paste0("I^2 = ", formatC(all_meta_dataiv_specif2$I2[index_summary_boys2], format = "f", digits = 2)),

paste0("I^2 = ", formatC(all_meta_dataiv_specif2$I2[index_summary_girls2], format = "f", digits = 2))))

}

graph_forest2 <- ggplot(data = all_meta_dataiv_specif2, aes(x = estim, y = num_pos_jit)) +

geom_point(aes(colour = sex, fill = sex, size = w.random_perc), shape = 22, alpha = 0.7) +

scale_size(guide = "none") +

geom_segment(aes(y=num_pos_jit, yend = num_pos_jit, x = lower, xend = upper, colour = sex)) +

scale_fill_manual(values = c("both_sexes" = "green", "boys" = "blue", "girls" = "red")) +

scale_colour_manual(values = c("both_sexes" = "green", "boys" = "blue", "girls" = "red")) +

scale_x_continuous(position = "top") +

geom_vline(xintercept = 0, alpha = 0.6) +

geom_polygon(data = diamonds_specif2, aes(x = TE, y = pos_diam, colour = sex, fill = sex), alpha=0.7) +

geom_text(data = dat_text_specif2, aes(x = pos_x, y = pos_y, label = text), hjust = "left", size = 5) +

geom_text(data = dat_textI2_specif2, aes(x = pos_x, y = pos_y, label = text), hjust = "left", size = 5) +

coord_cartesian(ylim = Ylimit) +

theme_classic() +

theme(axis.title.x =element_blank(),

axis.text.x = element_text(size=13, face="bold"),

axis.line.y=element_blank(),

axis.title.y=element_blank(),

axis.text.y=element_blank(),

axis.ticks.y=element_blank())

if(abs(min(all_meta_dataiv_specif2$lower))/abs(max(all_meta_dataiv_specif2$upper))>2 & min(all_meta_dataiv_specif2$lower)>0.9) {

graph_forest2 <- graph_forest2 + coord_cartesian(xlim = c(-0.3, 0.3))

} else if (abs(min(all_meta_dataiv_specif2$lower))/abs(max(all_meta_dataiv_specif2$upper))>2 & min(all_meta_dataiv_specif2$lower)<0.9) {

graph_forest2 <- graph_forest2 + coord_cartesian(xlim = c(-0.9, 0.9))

}

# attaching together the plot with the Table of information

Table_to_add1 <- select(all_meta_dataiv_specif, study, num_pos) %>%

group_by(study) %>%

slice(1) %>%

ungroup %>%

arrange(match(study, specif_studies))

Table_to_add1[nrow(Table_to_add1) + 1 , "num_pos"] <- -(nrow(Table_to_add1))-1

Table_to_add1[nrow(Table_to_add1) + 1 , "num_pos"] <- -(nrow(Table_to_add1))-1

Table_to_add1 <- gather(Table_to_add1, Stat, Value, study)

graph_table1 <- ggplot(data = Table_to_add1, aes(x = Stat, y = num_pos, label = Value)) +

geom_text(size = 5) +

scale_x_discrete(position = "top", labels = c("Study")) +

labs(y = NULL, x = NULL) +

coord_cartesian(ylim = Ylimit) +

theme_classic() +

theme(axis.title.x =element_blank(),

axis.text.x = element_text(size=14, face="bold"),

axis.line.y=element_blank(),

axis.title.y=element_blank(),

axis.text.y=element_blank(),

axis.ticks.y=element_blank())

Table_to_add2 <- select(all_meta_dataiv_specif, study, n_both_sexes, n_boys, n_girls, num_pos) %>%

group_by(study) %>%

slice(1) %>%

ungroup %>%

arrange(match(study, specif_studies))

Table_to_add2[nrow(Table_to_add2) + 1 , "num_pos"] <- -(nrow(Table_to_add2))-1

Table_to_add2[nrow(Table_to_add2) + 1 , "num_pos"] <- -(nrow(Table_to_add2))-1

Table_to_add2 <- select(Table_to_add2, -study) %>%

gather(Stat, Value, n_both_sexes, n_boys, n_girls)

graph_table2 <- ggplot(data = Table_to_add2, aes(x = Stat, y = num_pos, label = Value)) +

geom_text(size = 5) +

scale_x_discrete(position = "top", labels = c("both", "boys", "girls")) +

labs(y = NULL, x = NULL) +

coord_cartesian(ylim = Ylimit) +

theme_classic() +

theme(axis.title.x =element_blank(),

axis.text.x = element_text(size=14, face="bold"),

axis.line.y=element_blank(),

axis.title.y=element_blank(),

axis.text.y=element_blank(),

axis.ticks.y=element_blank())

grid.arrange(graph_table1, graph_table2, graph_forest1, graph_forest2, ncol=4, widths = c(12/100,8/100, 40/100, 40/100),

top = textGrob(paste0(group_to_consider, " exposure to ", molecules_to_consider[1], " (left plot) or ", molecules_to_consider[2], " (right plot) and association with ", ifelse(outcome_to_consider=="risk", "risk of overweight", outcome_to_consider)),gp=gpar(fontsize=20, face="bold")))

dev.print(png, paste0("Frigerio_", meta_num, molecules_to_consider[1], "_", molecules_to_consider[2], group_to_consider, outcome_to_consider, ".png"), width=1700, height=abs(min(all_meta_dataiv_specif$num_pos_jit))*18+85)

}

## Creating the plots:

generate_combined_forest_by2(all_meta_data = all_meta_dataiv, meta_num = "c01", molecules_to_consider = c("PFOA", "PFOS"), outcome_to_consider = "BMI", group_to_consider = "prenatal")

generate_combined_forest_by2(all_meta_data = all_meta_dataiv, meta_num = "c02", molecules_to_consider = c("PFHxS", "PFNA"), outcome_to_consider = "BMI", group_to_consider = "prenatal")

generate_combined_forest_by2(all_meta_data = all_meta_dataiv, meta_num = "c03", molecules_to_consider = c("PFDA", "PFUA"), outcome_to_consider = "BMI", group_to_consider = "prenatal")

generate_combined_forest_by2(all_meta_data = all_meta_dataiv, meta_num = "c04", molecules_to_consider = c("PFDoA", "PFBS"), outcome_to_consider = "BMI", group_to_consider = "prenatal")

generate_combined_forest_by2(all_meta_data = all_meta_dataiv, meta_num = "c05", molecules_to_consider = c("PFOA", "PFOS"), outcome_to_consider = "risk", group_to_consider = "prenatal")

generate_combined_forest_by2(all_meta_data = all_meta_dataiv, meta_num = "c06", molecules_to_consider = c("PFHxS", "PFNA"), outcome_to_consider = "risk", group_to_consider = "prenatal")

generate_combined_forest_by2(all_meta_data = all_meta_dataiv, meta_num = "c07", molecules_to_consider = c("PFOA", "PFOS"), outcome_to_consider = "WC", group_to_consider = "prenatal")

generate_combined_forest_by2(all_meta_data = all_meta_dataiv, meta_num = "c08", molecules_to_consider = c("PFHxS", "PFNA"), outcome_to_consider = "WC", group_to_consider = "prenatal")

generate_combined_forest_by2(all_meta_data = all_meta_dataiv, meta_num = "c09", molecules_to_consider = c("PFDA", "PFUA"), outcome_to_consider = "WC", group_to_consider = "prenatal")

generate_combined_forest_by2(all_meta_data = all_meta_dataiv, meta_num = "c10", molecules_to_consider = c("PFOA", "PFOS"), outcome_to_consider = "BMI", group_to_consider = "children")

generate_combined_forest_by2(all_meta_data = all_meta_dataiv, meta_num = "c11", molecules_to_consider = c("PFHxS", "PFNA"), outcome_to_consider = "BMI", group_to_consider = "children")

generate_combined_forest_by2(all_meta_data = all_meta_dataiv, meta_num = "c12", molecules_to_consider = c("PFOA", "PFOS"), outcome_to_consider = "risk", group_to_consider = "children")

## These plots were combined and saved as Figure 2

####### End of Section 7 #######

#################### End of the script ##################
