# Supplementary material for: Prenatal and childhood exposure to per-/polyfluoroalkyl substances (PFASs) and its associations with childhood overweight and/or obesity: a systematic review with meta-analyses
Source: Environ Health. 2023 Aug 14;22:56. doi: 10.1186/s12940-023-01006-6 (PMC10424367; doi:10.1186/s12940-023-01006-6)
Supplement: Supplementary file 2 — Additional file 2: Supplementary figures. In the supplementary figures file, for each meta-analysis, three figures are reported: a forest plot pooling the estimates with the generic invariance method, a forest plot pooling the estimates weighting by sample size, and a funnel plot evaluating the bias for included studies [file 12940_2023_1006_MOESM2_ESM.pdf]

Prenatal and childhood exposure to per-/polyfluoroalkyl substances (PFASs) and its associations with childhood overweight and/or obesity: a systematic review with meta-analyses

Gianfranco Frigerio, Chiara Matilde Ferrari, and Silvia Fustinoni

Supplementary figures

For each meta-analysis, three figures are reported:

- a forest plot pooling the estimates with the generic invariance method

- a forest plot pooling the estimates weighting by sample size

- a funnel plot evaluating the bias for included studies

Study	Sampling	Outcome	Subj	95%-CI	Weight (common)	Weight (random)
Zhang, Lei, et al., 2022	within 3 d before del	7 y	206	-0.00 [-0.00; 0.00]	30.1%	14.5%
Bloom et al., 2022	8-13 w gest	4-8 y	661	-0.01 [-0.03; 0.01]	0.0%	8.7%
Lauritzen et al., 2018	17-20 w gest	5 y	412	0.09 [-0.01; 0.19]	0.0%	0.9%
Gyllenhammar et al., 2018	3 w after del	5 y	183	0.11 [-0.03; 0.26]	0.0%	0.4%
Zhang, Pan, et al., 2022	15 w gest	3, 42, 182.6, and 365 d	2395	-0.01 [-0.01; 0.00]	0.1%	13.0%
Sevelsted et al., 2022	24 w gest - 1 w after del	10 y	536	0.18 [0.00; 0.36]	0.0%	0.3%
Liu et al., 2020	del	12 y	212	-0.01 [-0.06; 0.04]	0.0%	3.1%
Mora et al., 2017	9.6 w gest	7.7 y	871	0.01 [-0.01; 0.04]	0.0%	8.4%
Jensen et al., 2020	before 16 w gest	3 m, 18 m	593	0.04 [-0.01; 0.10]	0.0%	2.5%
Chen et al., 2019	del	5 y	404	0.00 [-0.00; 0.00]	69.7%	14.5%
Chen et al., 2017	del	60 to 108 m	429	0.01 [-0.09; 0.11]	0.0%	0.9%
Andersen et al., 2010	1st tr gest, 2nd tr gest, del	12 m	1026	-0.02 [-0.05; 0.00]	0.0%	7.4%
Karlsen et al., 2017	2 w after del	5 y	371	0.04 [-0.00; 0.08]	0.0%	4.2%
Yeung et al., 2019	del	4 to 36 m	1850	-0.03 [-0.05; -0.02]	0.0%	10.1%
Manzano-Salgado et al., 2017	1st tr gest	7 y	1086	0.02 [-0.04; 0.07]	0.0%	2.6%
Hoyer et al., 2015 - Greenland	24 ± 10 w gest	5-9 y	531	0.05 [-0.06; 0.16]	0.0%	0.7%
Hoyer et al., 2015 - Ukraine	24 ± 10 w gest	5-9 y	491	0.01 [-0.13; 0.16]	0.0%	0.4%
Vrijheid et al., 2020	during gest	6-11 years	1301	-0.01 [-0.03; 0.02]	0.0%	7.4%
**Common effect model**				**0.00 [-0.00; 0.00]**	**100.0%**	--
**Random effects model**				**-0.00 [-0.01; 0.01]**	--	**100.0%**

Heterogeneity: $I^2 = 58\%$, $\tau^2 = 0.0002$, $p < 0.01$

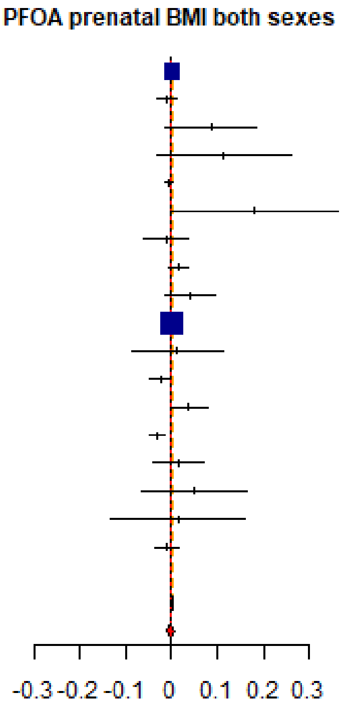

Study	Sampling	Outcome	Subjects	Estimate [95% CI]
Zhang, Lei, et al., 2022	within 3 d before del	7 y	206	0.000 [-0.001; 0.000]
Bloom et al., 2022	8-13 w gest	4-8 y	661	-0.010 [-0.031; 0.011]
Lauritzen et al., 2018	17-20 w gest	5 y	412	0.086 [-0.014; 0.186]
Gyllenhammar et al., 2018	3 w after del	5 y	183	0.114 [-0.032; 0.261]
Zhang, Pan, et al., 2022	15 w gest	3, 42, 182.6, and 365 d	2395	-0.006 [-0.014; 0.003]
Sevelsted et al., 2022	24 w gest - 1 w after del	10 y	536	0.180 [0.000; 0.360]
Liu et al., 2020	del	12 y	212	-0.012 [-0.061; 0.037]
Mora et al., 2017	9.6 w gest	7.7 y	871	0.014 [-0.008; 0.035]
Jensen et al., 2020	before 16 w gest	3 m, 18 m	593	0.040 [-0.015; 0.095]
Chen et al., 2019	del	5 y	404	0.000 [0.000; 0.000]
Chen et al., 2017	del	60 to 108 m	429	0.013 [-0.087; 0.112]
Andersen et al., 2010	1st tr gest, 2nd tr gest, del	12 m	1026	-0.025 [-0.050; 0.000]
Karlsen et al., 2017	2 w after del	5 y	371	0.037 [-0.002; 0.077]
Yeung et al., 2019	del	4 to 36 m	1850	-0.034 [-0.050; -0.017]
Manzano-Salgado et al., 2017	1st tr gest	7 y	1086	0.016 [-0.039; 0.070]
Hoyer et al., 2015 - Greenland	24 ± 10 w gest	5-9 y	531	0.049 [-0.064; 0.161]
Hoyer et al., 2015 - Ukraine	24 ± 10 w gest	5-9 y	491	0.014 [-0.132; 0.159]
Vrijheid et al., 2020	during gest	6-11 years	1301	-0.009 [-0.034; 0.015]
**Summary**				**0.010 [-0.003; 0.023]**

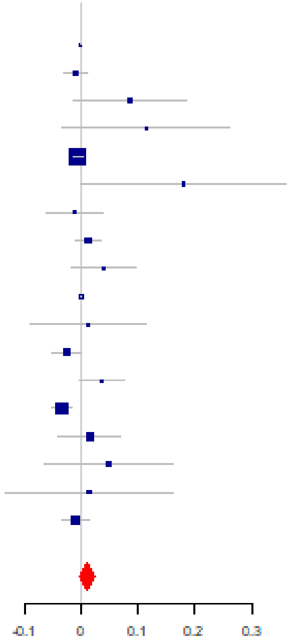

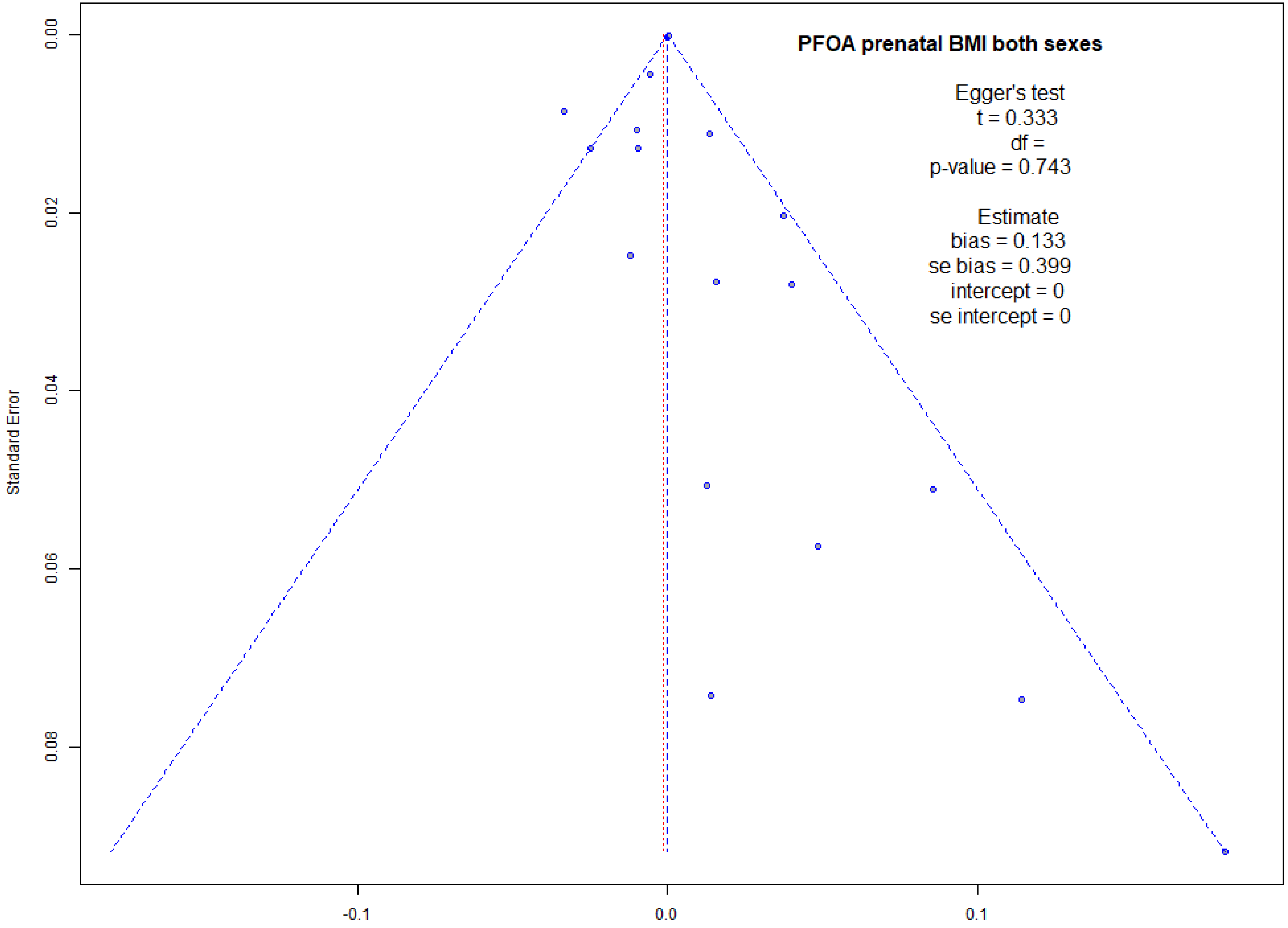
PFOA prenatal BMI both sexes
Egger's test
t = 0.333
df =
p-value = 0.743
Estimate
bias = 0.133
se bias = 0.399
intercept = 0
se intercept = 0
Standard Error
0.00
0.02
0.04
0.06
0.08
-0.1
0.0
0.1

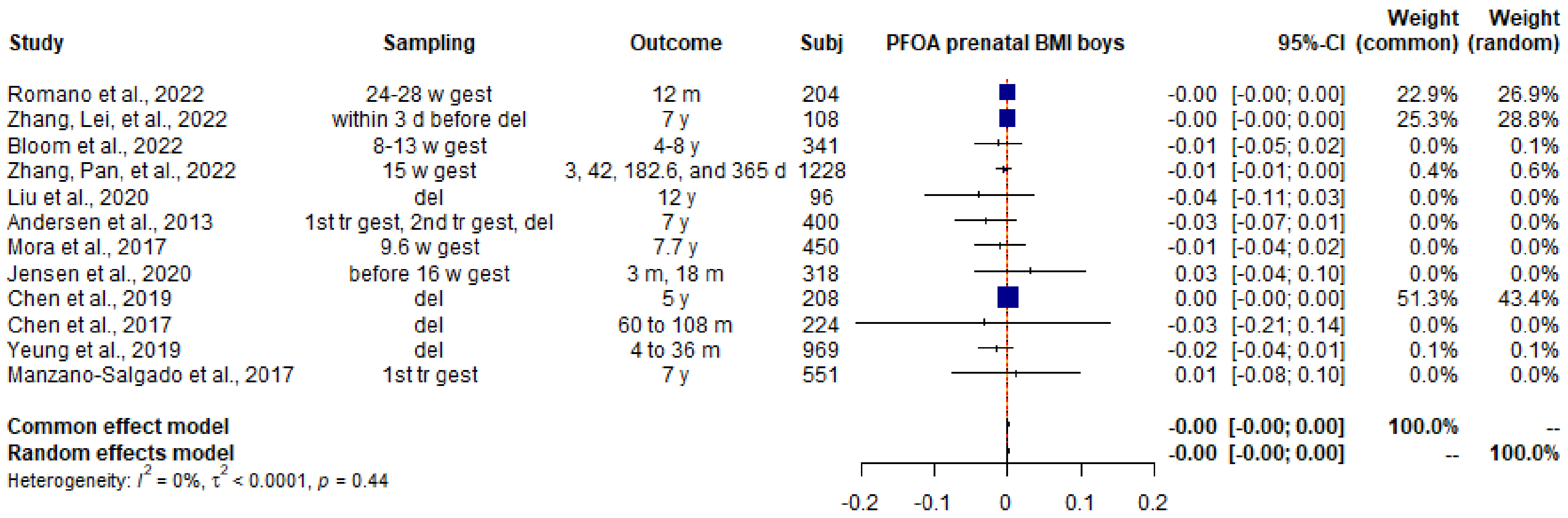
Study
Sampling
Outcome
Subj
PFOA prenatal BMI boys
95%-CI
Weight (common)
Weight (random)
Romano et al., 2022 24-28 w gest 12 m 204 -0.00 [-0.00; 0.00] 22.9% 26.9%
Zhang, Lei, et al., 2022 within 3 d before del 7 y 108 -0.00 [-0.00; 0.00] 25.3% 28.8%
Bloom et al., 2022 8-13 w gest 4-8 y 341 -0.01 [-0.05; 0.02] 0.0% 0.1%
Zhang, Pan, et al., 2022 15 w gest 3, 42, 182.6, and 365 d 1228 -0.01 [-0.01; 0.00] 0.4% 0.6%
Liu et al., 2020 del 12 y 96 -0.04 [-0.11; 0.03] 0.0% 0.0%
Andersen et al., 2013 1st tr gest, 2nd tr gest, del 7 y 400 -0.03 [-0.07; 0.01] 0.0% 0.0%
Mora et al., 2017 9.6 w gest 7.7 y 450 -0.01 [-0.04; 0.02] 0.0% 0.0%
Jensen et al., 2020 before 16 w gest 3 m, 18 m 318 0.03 [-0.04; 0.10] 0.0% 0.0%
Chen et al., 2019 del 5 y 208 0.00 [-0.00; 0.00] 51.3% 43.4%
Chen et al., 2017 del 60 to 108 m 224 -0.03 [-0.21; 0.14] 0.0% 0.0%
Yeung et al., 2019 del 4 to 36 m 969 -0.02 [-0.04; 0.01] 0.1% 0.1%
Manzano-Salgado et al., 2017 1st tr gest 7 y 551 0.01 [-0.08; 0.10] 0.0% 0.0%
Common effect model -0.00 [-0.00; 0.00] 100.0% --
Random effects model -0.00 [-0.00; 0.00] -- 100.0%
Heterogeneity: I^2 = 0%, τ^2 < 0.0001, p = 0.44
-0.2
-0.1
0
0.1
0.2

Study	Sampling	Outcome	Subjects	Estimate [95% CI]
Romano et al., 2022	24-28 w gest	12 m	204	-0.001 [-0.002; 0.001]
Zhang, Lei, et al., 2022	within 3 d before del	7 y	108	-0.001 [-0.002; 0.001]
Bloom et al., 2022	8-13 w gest	4-8 y	341	-0.014 [-0.046; 0.018]
Zhang, Pan, et al., 2022	15 w gest	3, 42, 182.6, and 365 d	1228	-0.006 [-0.015; 0.003]
Liu et al., 2020	del	12 y	96	-0.040 [-0.114; 0.034]
Andersen et al., 2013	1st tr gest, 2nd tr gest, del	7 y	400	-0.030 [-0.072; 0.011]
Mora et al., 2017	9.6 w gest	7.7 y	450	-0.011 [-0.045; 0.023]
Jensen et al., 2020	before 16 w gest	3 m, 18 m	318	0.030 [-0.045; 0.105]
Chen et al., 2019	del	5 y	208	0.000 [0.000; 0.001]
Chen et al., 2017	del	60 to 108 m	224	-0.034 [-0.207; 0.140]
Yeung et al., 2019	del	4 to 36 m	969	-0.017 [-0.040; 0.006]
Manzano-Salgado et al., 2017	1st tr gest	7 y	551	0.010 [-0.078; 0.098]
**Summary**				**-0.008 [-0.023; 0.007]**

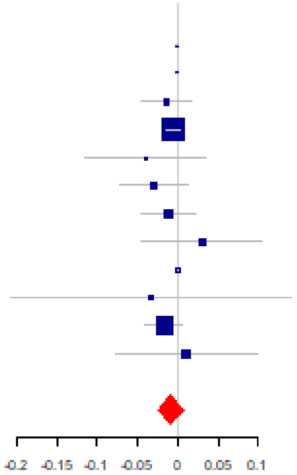

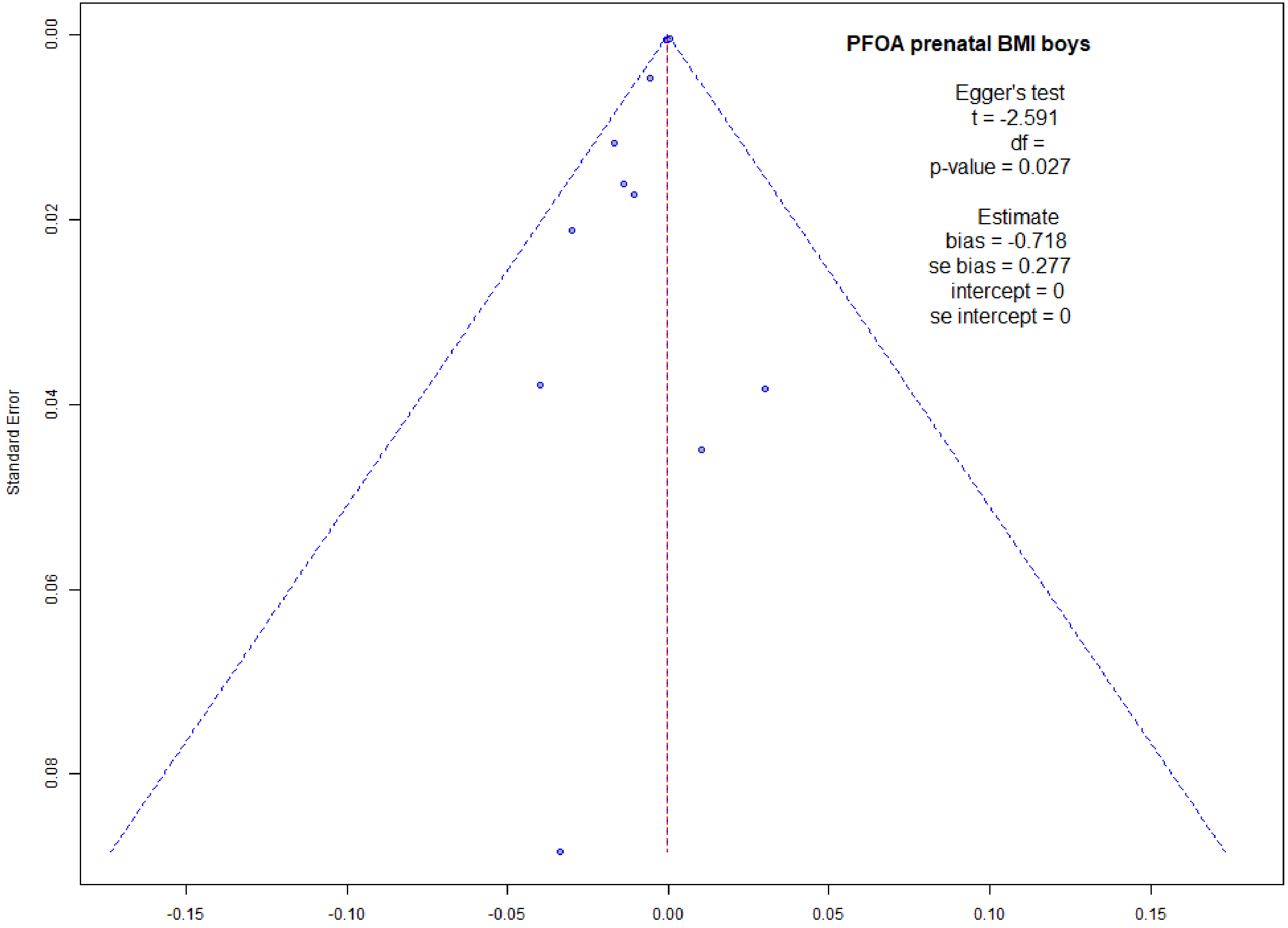
PFOA prenatal BMI boys
Egger's test
t = -2.591
df =
p-value = 0.027
Estimate
bias = -0.718
se bias = 0.277
intercept = 0
se intercept = 0
Standard Error
0.00
0.02
0.04
0.06
0.08
-0.15
-0.10
-0.05
0.00
0.05
0.10
0.15

Study	Sampling	Outcome	Subj		95%-CI	Weight (common)	Weight (random)
Romano et al., 2022	24-28 w gest	12 m	214	0.00	[-0.00; 0.01]	0.1%	0.1%
Zhang, Lei, et al., 2022	within 3 d before del	7 y	98	0.00	[-0.00; 0.00]	17.1%	17.1%
Bloom et al., 2022	8-13 w gest	4-8 y	320	-0.01	[-0.04; 0.02]	0.0%	0.0%
Zhang, Pan, et al., 2022	15 w gest	3, 42, 182.6, and 365 d	1167	0.01	[-0.00; 0.02]	0.0%	0.0%
Hartman et al., 2017	15 w gest	9 y	312	-0.01	[-0.02; 0.00]	0.0%	0.0%
Liu et al., 2020	del	12 y	116	0.01	[-0.04; 0.07]	0.0%	0.0%
Andersen et al., 2013	1st tr gest, 2nd tr gest, del	7 y	387	-0.01	[-0.05; 0.03]	0.0%	0.0%
Mora et al., 2017	9.6 w gest	7.7 y	421	0.03	[0.00; 0.05]	0.0%	0.0%
Jensen et al., 2020	before 16 w gest	3 m, 18 m	275	0.06	[-0.02; 0.14]	0.0%	0.0%
Chen et al., 2019	del	5 y	196	0.00	[-0.00; 0.00]	82.7%	82.7%
Chen et al., 2017	del	60 to 108 m	205	0.06	[-0.05; 0.17]	0.0%	0.0%
Yeung et al., 2019	del	4 to 36 m	881	-0.05	[-0.08; -0.03]	0.0%	0.0%
Manzano-Salgado et al., 2017	1st tr gest	7 y	535	0.03	[-0.05; 0.10]	0.0%	0.0%
**Common effect model**				**0.00**	**[-0.00; 0.00]**	**100.0%**	--
**Random effects model**				**0.00**	**[-0.00; 0.00]**	--	**100.0%**

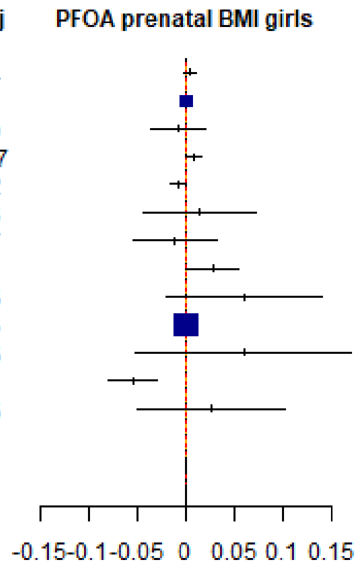

Heterogeneity: I^2 = 66%, τ^2 = 0, p < 0.01

Study	Sampling	Outcome	Subjects	Estimate [95% CI]
Romano et al., 2022	24-28 w gest	12 m	214	0.004 [-0.003; 0.010]
Zhang, Lei, et al., 2022	within 3 d before del	7 y	98	0.000 [0.000; 0.001]
Bloom et al., 2022	8-13 w gest	4-8 y	320	-0.008 [-0.036; 0.020]
Zhang, Pan, et al., 2022	15 w gest	3, 42, 182.6, and 365 d	1167	0.007 [-0.001; 0.016]
Hartman et al., 2017	15 w gest	9 y	312	-0.008 [-0.015; 0.000]
Liu et al., 2020	del	12 y	116	0.013 [-0.045; 0.071]
Andersen et al., 2013	1st tr gest, 2nd tr gest, del	7 y	387	-0.011 [-0.053; 0.032]
Mora et al., 2017	9.6 w gest	7.7 y	421	0.027 [0.000; 0.054]
Jensen et al., 2020	before 16 w gest	3 m, 18 m	275	0.060 [-0.020; 0.140]
Chen et al., 2019	del	5 y	196	0.000 [0.000; 0.000]
Chen et al., 2017	del	60 to 108 m	205	0.059 [-0.051; 0.169]
Yeung et al., 2019	del	4 to 36 m	881	-0.054 [-0.080; -0.029]
Manzano-Salgado et al., 2017	1st tr gest	7 y	535	0.026 [-0.049; 0.101]
**Summary**				**0.001 [-0.010; 0.013]**

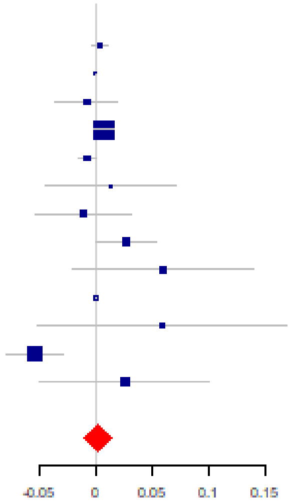

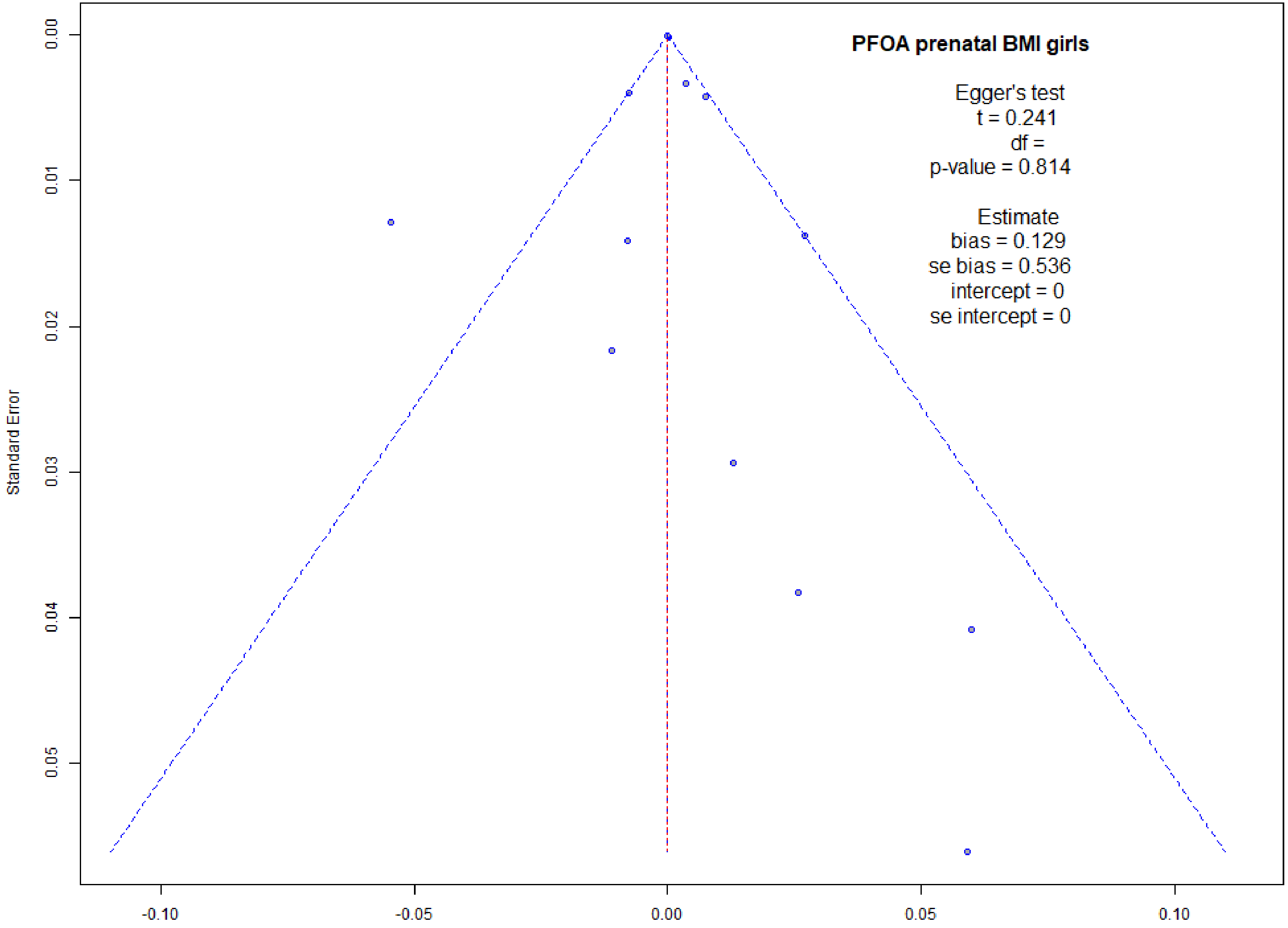
PFOA prenatal BMI girls
Egger's test
t = 0.241
df =
p-value = 0.814
Estimate
bias = 0.129
se bias = 0.536
intercept = 0
se intercept = 0
Standard Error
0.00
0.01
0.02
0.03
0.04
0.05
-0.10
-0.05
0.00
0.05
0.10

Study	Sampling	Outcome	Subj	95%-CI	Weight (common)	Weight (random)
Zhang, Lei, et al., 2022	within 3 d before del	7 y	206	0.00 [-0.00; 0.01]	1.2%	2.9%
Bloom et al., 2022	8-13 w gest	4-8 y	661	-0.00 [-0.01; 0.00]	5.7%	11.6%
Lauritzen et al., 2018	17-20 w gest	5 y	412	0.02 [0.00; 0.03]	0.2%	0.5%
Gyllenhammar et al., 2018	3 w after del	5 y	182	0.02 [0.01; 0.04]	0.2%	0.5%
Zhang, Pan, et al., 2022	15 w gest	3, 42, 182.6, and 365 d	2395	-0.00 [-0.01; 0.00]	1.0%	2.4%
Sevelsted et al., 2022	24 w gest - 1 w after del	10 y	536	-0.03 [-0.07; 0.01]	0.0%	0.1%
Liu et al., 2020	del	12 y	212	-0.00 [-0.03; 0.02]	0.1%	0.2%
Mora et al., 2017	9.6 w gest	7.7 y	871	0.01 [0.00; 0.01]	3.4%	7.4%
Jensen et al., 2020	before 16 w gest	3 m, 18 m	593	-0.01 [-0.03; 0.01]	0.2%	0.4%
Chen et al., 2019	del	5 y	404	-0.00 [-0.00; 0.00]	79.5%	56.2%
Chen et al., 2017	del	60 to 108 m	429	-0.00 [-0.05; 0.05]	0.0%	0.1%
Andersen et al., 2010	1st tr gest, 2nd tr gest, del	12 m	1026	-0.01 [-0.06; 0.05]	0.0%	0.0%
Karlsen et al., 2017	2 w after del	5 y	371	0.00 [-0.01; 0.01]	0.7%	1.7%
Yeung et al., 2019	del	4 to 36 m	1850	-0.02 [-0.03; -0.01]	0.9%	2.0%
Manzano-Salgado et al., 2017	1st tr gest	7 y	1086	0.01 [-0.02; 0.03]	0.1%	0.2%
Hoyer et al., 2015 - Greenland	24 ± 10 w gest	5-9 y	531	-0.00 [-0.01; 0.01]	0.5%	1.1%
Hoyer et al., 2015 - Ukraine	24 ± 10 w gest	5-9 y	491	0.01 [-0.03; 0.05]	0.0%	0.1%
Vrijheid et al., 2020	during gest	6-11 years	1301	-0.00 [-0.00; 0.00]	6.2%	12.6%
**Common effect model**				**-0.00 [-0.00; 0.00]**	**100.0%**	--
**Random effects model**				**-0.00 [-0.00; 0.00]**	--	**100.0%**

Heterogeneity: $I^2 = 57\%$, $\tau^2 < 0.0001$, $p < 0.01$

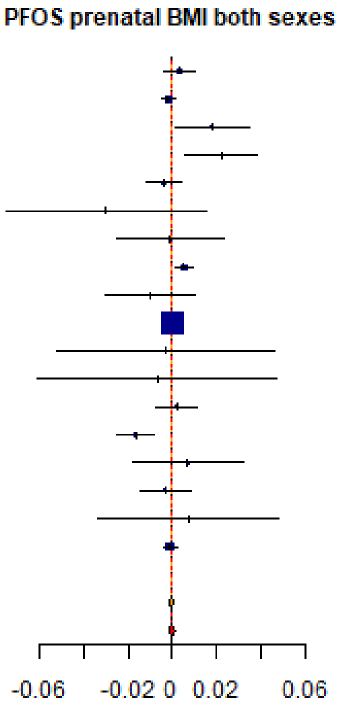

Study	Sampling	Outcome	Subjects	Estimate [95% CI]
Zhang, Lei, et al., 2022	within 3 d before del	7 y	206	0.003 [-0.004; 0.010]
Bloom et al., 2022	8-13 w gest	4-8 y	661	-0.002 [-0.005; 0.001]
Lauritzen et al., 2018	17-20 w gest	5 y	412	0.018 [0.001; 0.035]
Gyllenhammar et al., 2018	3 w after del	5 y	182	0.022 [0.006; 0.039]
Zhang, Pan, et al., 2022	15 w gest	3, 42, 182.6, and 365 d	2395	-0.004 [-0.012; 0.004]
Sevelsted et al., 2022	24 w gest - 1 w after del	10 y	536	-0.030 [-0.075; 0.015]
Liu et al., 2020	del	12 y	212	-0.001 [-0.025; 0.023]
Mora et al., 2017	9.6 w gest	7.7 y	871	0.005 [0.001; 0.009]
Jensen et al., 2020	before 16 w gest	3 m, 18 m	593	-0.010 [-0.030; 0.010]
Chen et al., 2019	del	5 y	404	0.000 [-0.001; 0.001]
Chen et al., 2017	del	60 to 108 m	429	-0.003 [-0.053; 0.046]
Andersen et al., 2010	1st tr gest, 2nd tr gest, del	12 m	1026	-0.007 [-0.061; 0.047]
Karlsen et al., 2017	2 w after del	5 y	371	0.002 [-0.007; 0.011]
Yeung et al., 2019	del	4 to 36 m	1850	-0.017 [-0.025; -0.008]
Manzano-Salgado et al., 2017	1st tr gest	7 y	1086	0.007 [-0.018; 0.032]
Hoyer et al., 2015 - Greenland	24 ± 10 w gest	5-9 y	531	-0.003 [-0.015; 0.008]
Hoyer et al., 2015 - Ukraine	24 ± 10 w gest	5-9 y	491	0.007 [-0.034; 0.048]
Vrijheid et al., 2020	during gest	6-11 years	1301	-0.001 [-0.004; 0.002]
**Summary**				**-0.004 [-0.010; 0.003]**

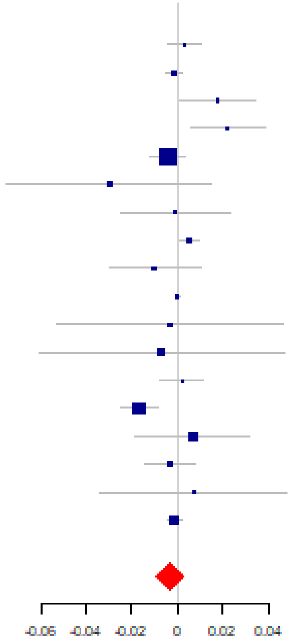

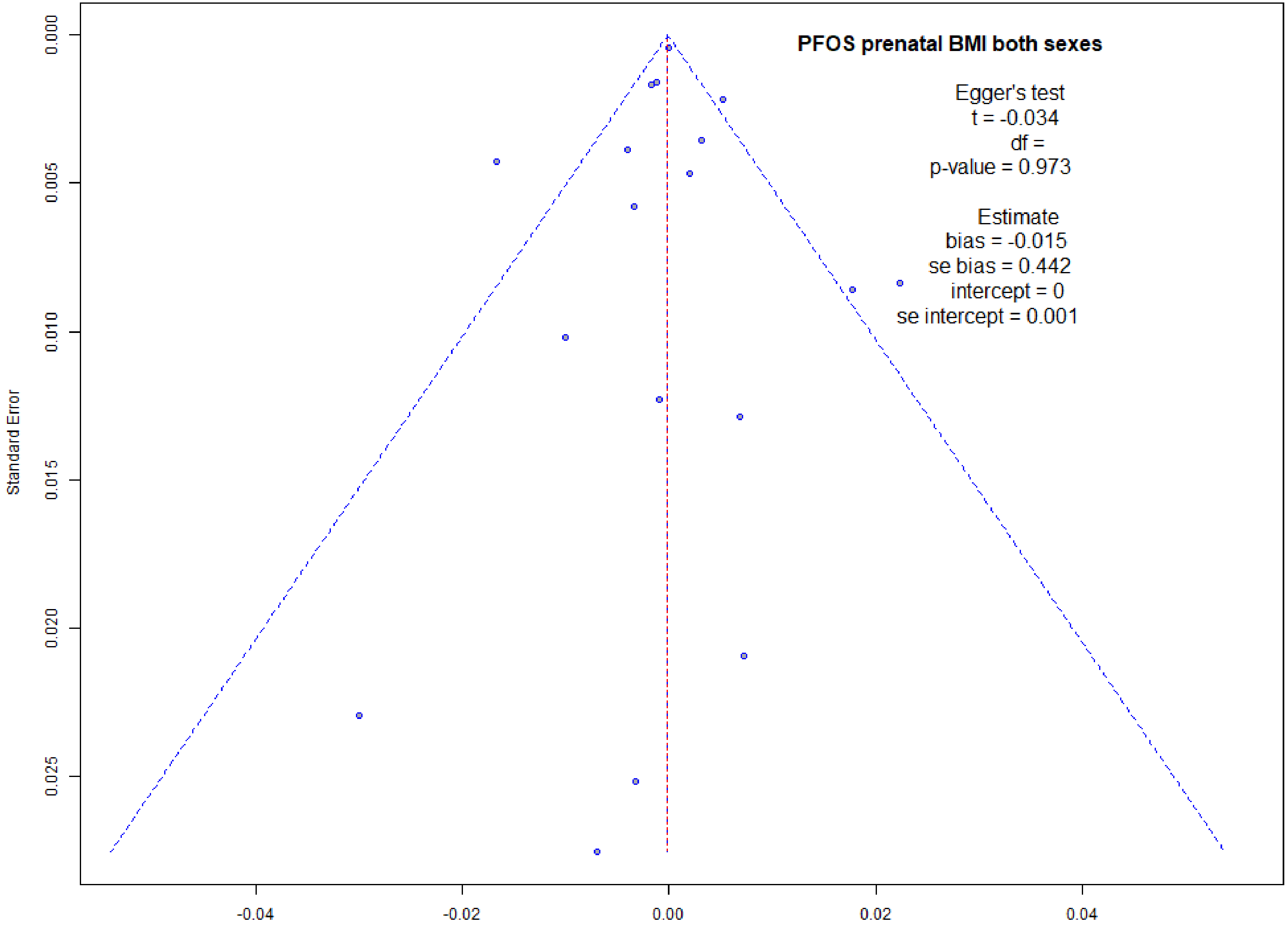
PFOS prenatal BMI both sexes
Egger's test
t = -0.034
df =
p-value = 0.973
Estimate
bias = -0.015
se bias = 0.442
intercept = 0
se intercept = 0.001
Standard Error
0.000
0.005
0.010
0.015
0.020
0.025
-0.04
-0.02
0.00
0.02
0.04

Study	Sampling	Outcome	Subj	PFOS prenatal BMI boys	95%-CI	Weight (common)	Weight (random)
Romano et al., 2022	24-28 w gest	12 m	204		-0.00 [-0.00; 0.00]	99.0%	68.9%
Zhang, Lei, et al., 2022	within 3 d before del	7 y	108		0.00 [-0.01; 0.02]	0.0%	0.5%
Bloom et al., 2022	8-13 w gest	4-8 y	341		0.00 [-0.00; 0.01]	0.1%	3.9%
Zhang, Pan, et al., 2022	15 w gest	3, 42, 182.6, and 365 d	1228		-0.00 [-0.01; 0.00]	0.0%	1.6%
Liu et al., 2020	del	12 y	96		-0.00 [-0.04; 0.03]	0.0%	0.1%
Andersen et al., 2013	1st tr gest, 2nd tr gest, del	7 y	400		-0.00 [-0.01; 0.00]	0.0%	1.9%
Mora et al., 2017	9.6 w gest	7.7 y	450		0.00 [-0.00; 0.01]	0.1%	2.1%
Jensen et al., 2020	before 16 w gest	3 m, 18 m	318		-0.00 [-0.03; 0.02]	0.0%	0.1%
Chen et al., 2019	del	5 y	208		0.00 [-0.00; 0.00]	0.7%	20.2%
Chen et al., 2017	del	60 to 108 m	224		-0.04 [-0.08; 0.01]	0.0%	0.0%
Yeung et al., 2019	del	4 to 36 m	969		-0.01 [-0.02; 0.00]	0.0%	0.6%
Manzano-Salgado et al., 2017	1st tr gest	7 y	551		0.00 [-0.03; 0.04]	0.0%	0.1%
**Common effect model**					**-0.00 [-0.00; 0.00]**	**100.0%**	**--**
**Random effects model**					**-0.00 [-0.00; 0.00]**	**--**	**100.0%**

Heterogeneity: $I^2 = 0\%$, $\tau^2 < 0.0001$, $p = 0.50$

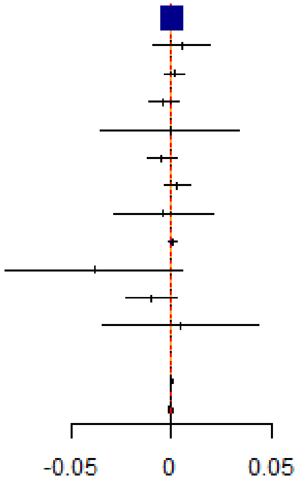

Study	Sampling	Outcome	Subjects	Estimate [95% CI]
Romano et al., 2022	24-28 w gest	12 m	204	0.000 [0.000; 0.000]
Zhang, Lei, et al., 2022	within 3 d before del	7 y	108	0.005 [-0.009; 0.019]
Bloom et al., 2022	8-13 w gest	4-8 y	341	0.001 [-0.004; 0.006]
Zhang, Pan, et al., 2022	15 w gest	3, 42, 182.6, and 365 d	1228	-0.004 [-0.012; 0.004]
Liu et al., 2020	del	12 y	96	-0.001 [-0.035; 0.033]
Andersen et al., 2013	1st tr gest, 2nd tr gest, del	7 y	400	-0.005 [-0.012; 0.002]
Mora et al., 2017	9.6 w gest	7.7 y	450	0.003 [-0.004; 0.009]
Jensen et al., 2020	before 16 w gest	3 m, 18 m	318	-0.004 [-0.029; 0.021]
Chen et al., 2019	del	5 y	208	0.000 [-0.001; 0.002]
Chen et al., 2017	del	60 to 108 m	224	-0.039 [-0.083; 0.006]
Yeung et al., 2019	del	4 to 36 m	969	-0.010 [-0.023; 0.002]
Manzano-Salgado et al., 2017	1st tr gest	7 y	551	0.005 [-0.034; 0.044]
**Summary**				**-0.004 [-0.010; 0.001]**

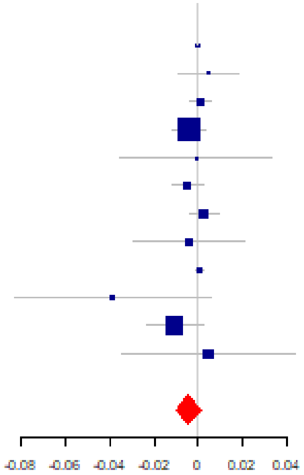

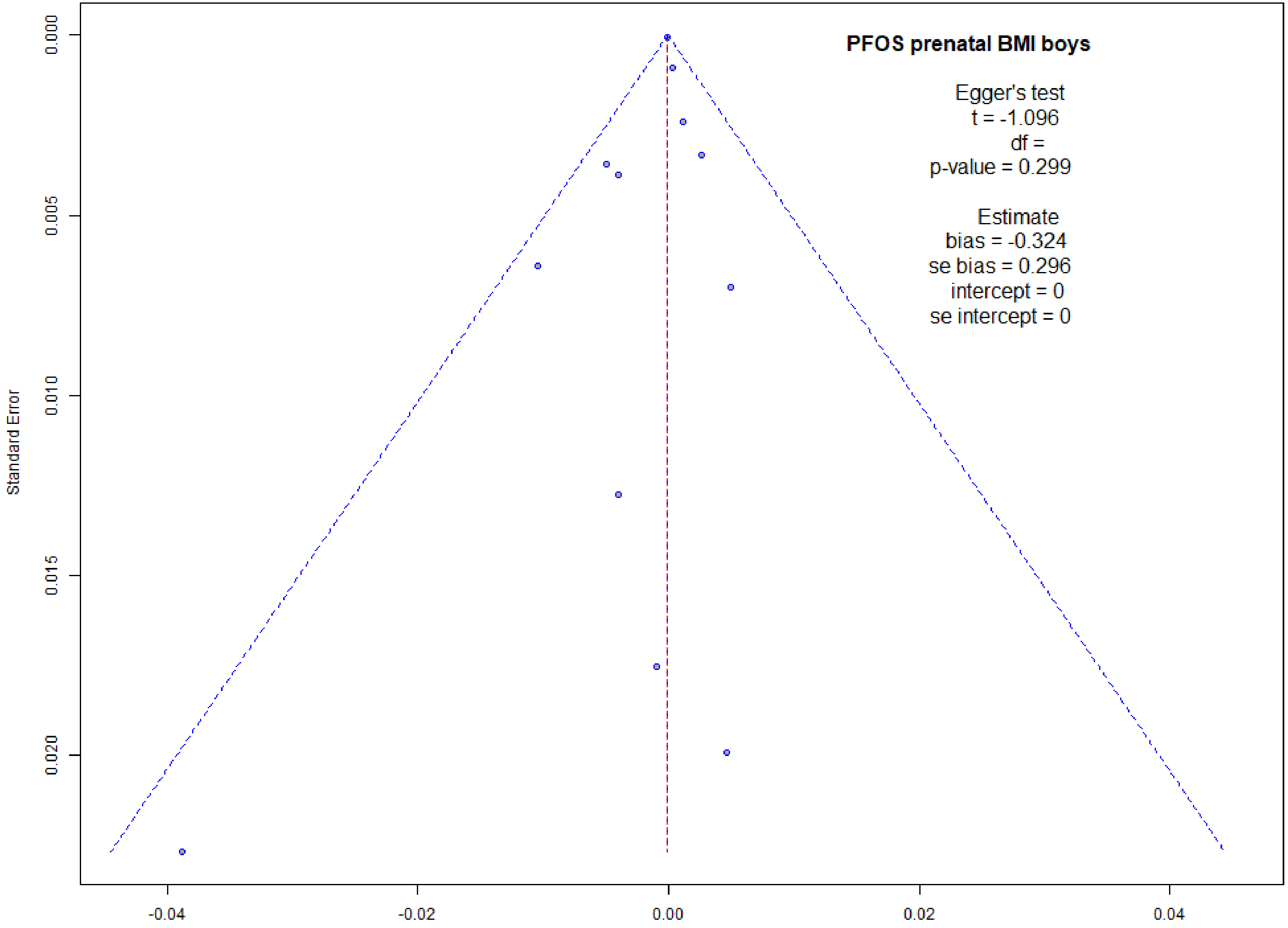
PFOS prenatal BMI boys
Egger's test
t = -1.096
df =
p-value = 0.299
Estimate
bias = -0.324
se bias = 0.296
intercept = 0
se intercept = 0
Standard Error
0.000
0.005
0.010
0.015
0.020
-0.04
-0.02
0.00
0.02
0.04

Study	Sampling	Outcome	Subj	PFOS prenatal BMI girls	95%-CI	Weight (common)	Weight (random)
Romano et al., 2022	24-28 w gest	12 m	214		0.00 [-0.00; 0.00]	16.5%	15.2%
Zhang, Lei, et al., 2022	within 3 d before del	7 y	98		-0.00 [-0.01; 0.00]	0.5%	10.8%
Bloom et al., 2022	8-13 w gest	4-8 y	320		-0.01 [-0.01; -0.00]	0.6%	11.5%
Zhang, Pan, et al., 2022	15 w gest	3, 42, 182.6, and 365 d	1167		0.00 [-0.01; 0.01]	0.2%	7.5%
Hartman et al., 2017	15 w gest	9 y	312		-0.00 [-0.00; -0.00]	4.7%	14.7%
Liu et al., 2020	del	12 y	116		0.00 [-0.03; 0.03]	0.0%	1.0%
Andersen et al., 2013	1st tr gest, 2nd tr gest, del	7 y	387		-0.00 [-0.01; 0.00]	0.2%	8.1%
Mora et al., 2017	9.6 w gest	7.7 y	421		0.01 [0.00; 0.01]	0.3%	9.5%
Jensen et al., 2020	before 16 w gest	3 m, 18 m	275		-0.01 [-0.04; 0.02]	0.0%	1.0%
Chen et al., 2019	del	5 y	196		-0.00 [-0.00; 0.00]	76.7%	15.3%
Chen et al., 2017	del	60 to 108 m	205		0.05 [0.00; 0.11]	0.0%	0.3%
Yeung et al., 2019	del	4 to 36 m	881		-0.02 [-0.04; -0.01]	0.1%	4.4%
Manzano-Salgado et al., 2017	1st tr gest	7 y	535		0.01 [-0.02; 0.04]	0.0%	0.8%
**Common effect model**					**-0.00 [-0.00; 0.00]**	**100.0%**	**--**
**Random effects model**					**-0.00 [-0.00; 0.00]**	**--**	**100.0%**

Heterogeneity: $I^2 = 63\%$, $\tau^2 < 0.0001$, $p < 0.01$

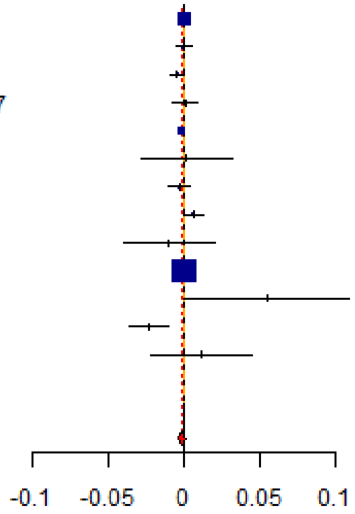

Study	Sampling	Outcome	Subjects	Estimate [95% CI]
Romano et al., 2022	24-28 w gest	12 m	214	0.000 [-0.001; 0.001]
Zhang, Lei, et al., 2022	within 3 d before del	7 y	98	0.000 [-0.005; 0.005]
Bloom et al., 2022	8-13 w gest	4-8 y	320	-0.005 [-0.010; 0.000]
Zhang, Pan, et al., 2022	15 w gest	3, 42, 182.6, and 365 d	1167	0.001 [-0.007; 0.009]
Hartman et al., 2017	15 w gest	9 y	312	-0.002 [-0.004; 0.000]
Liu et al., 2020	del	12 y	116	0.002 [-0.028; 0.032]
Andersen et al., 2013	1st tr gest, 2nd tr gest, del	7 y	387	-0.003 [-0.010; 0.004]
Mora et al., 2017	9.6 w gest	7.7 y	421	0.006 [0.000; 0.013]
Jensen et al., 2020	before 16 w gest	3 m, 18 m	275	-0.010 [-0.040; 0.020]
Chen et al., 2019	del	5 y	196	0.000 [0.000; 0.000]
Chen et al., 2017	del	60 to 108 m	205	0.055 [0.001; 0.109]
Yeung et al., 2019	del	4 to 36 m	881	-0.023 [-0.036; -0.010]
Manzano-Salgado et al., 2017	1st tr gest	7 y	535	0.011 [-0.022; 0.045]
**Summary**				**-0.001 [-0.006; 0.004]**

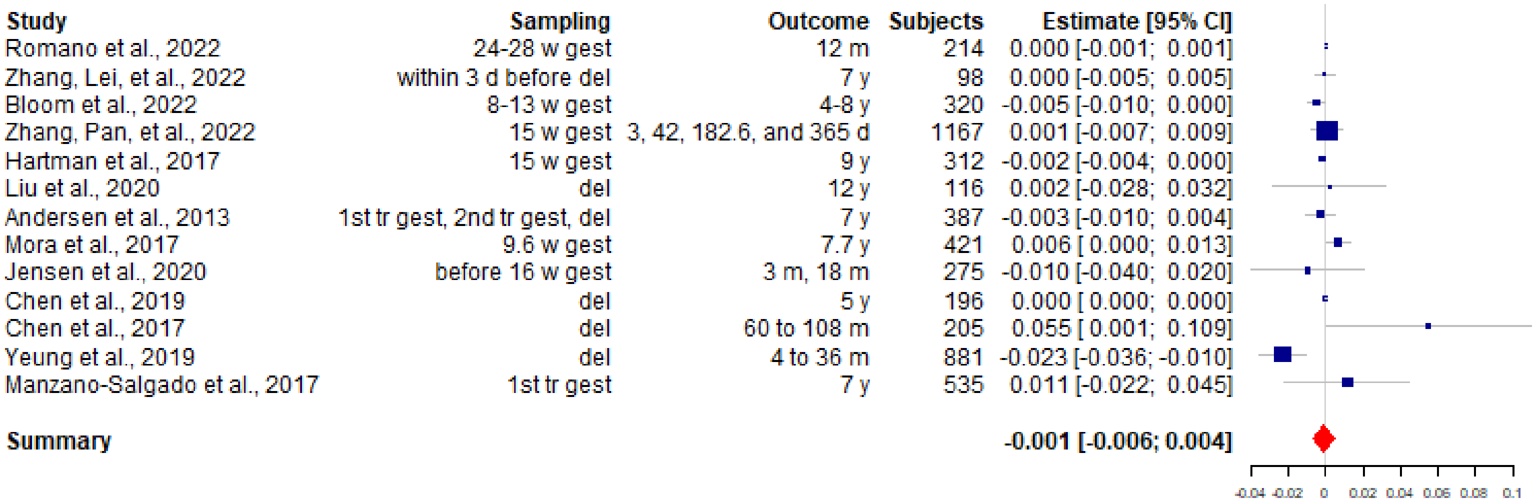

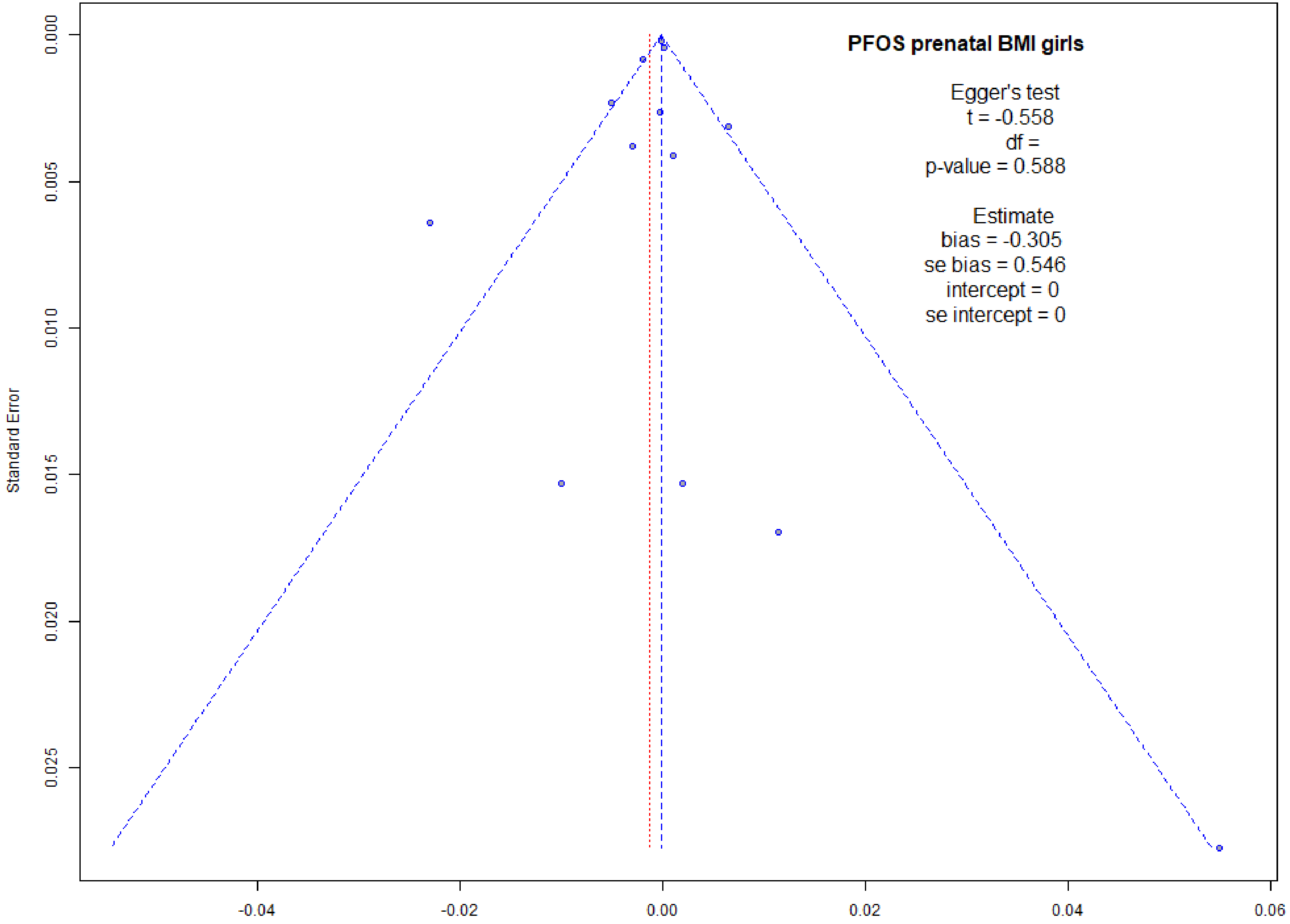
PFOS prenatal BMI girls
Egger's test
t = -0.558
df =
p-value = 0.588
Estimate
bias = -0.305
se bias = 0.546
intercept = 0
se intercept = 0
Standard Error
0.000
0.005
0.010
0.015
0.020
0.025
-0.04
-0.02
0.00
0.02
0.04
0.06

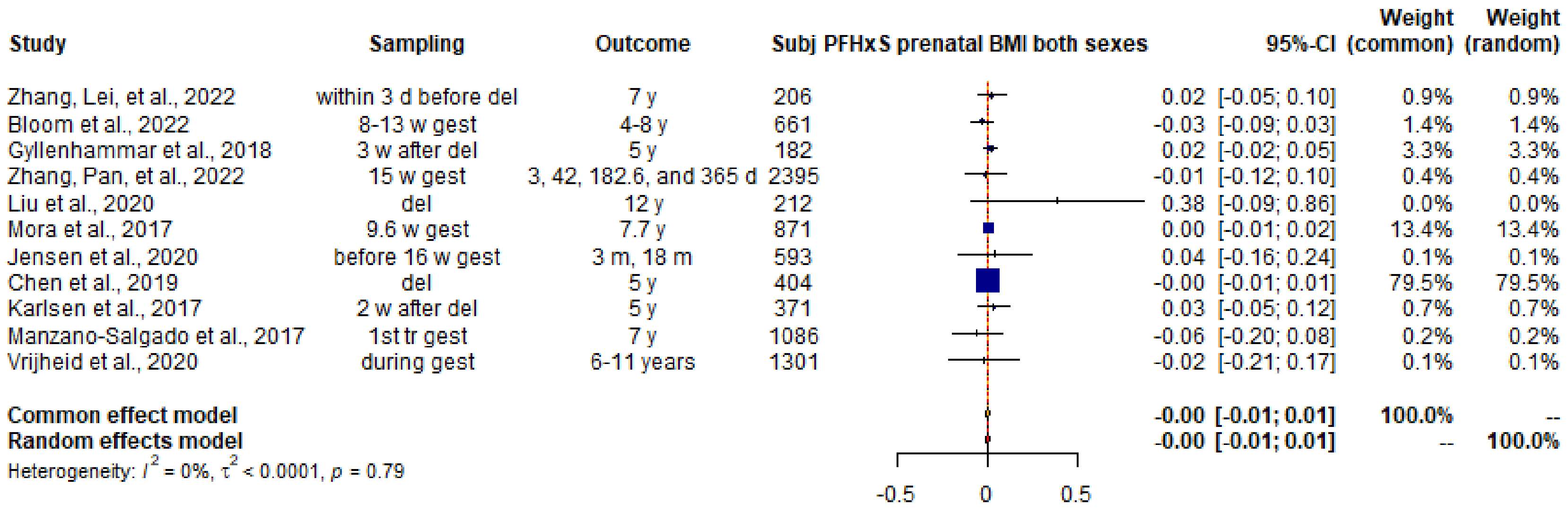
Study
Sampling
Outcome
Subj PFHxS prenatal BMI both sexes
95%-CI
Weight (common)
Weight (random)
Zhang, Lei, et al., 2022 within 3 d before del 7 y 206 0.02 [-0.05; 0.10] 0.9% 0.9%
Bloom et al., 2022 8-13 w gest 4-8 y 661 -0.03 [-0.09; 0.03] 1.4% 1.4%
Gyllenhammar et al., 2018 3 w after del 5 y 182 0.02 [-0.02; 0.05] 3.3% 3.3%
Zhang, Pan, et al., 2022 15 w gest 3, 42, 182.6, and 365 d 2395 -0.01 [-0.12; 0.10] 0.4% 0.4%
Liu et al., 2020 del 12 y 212 0.38 [-0.09; 0.86] 0.0% 0.0%
Mora et al., 2017 9.6 w gest 7.7 y 871 0.00 [-0.01; 0.02] 13.4% 13.4%
Jensen et al., 2020 before 16 w gest 3 m, 18 m 593 0.04 [-0.16; 0.24] 0.1% 0.1%
Chen et al., 2019 del 5 y 404 -0.00 [-0.01; 0.01] 79.5% 79.5%
Karlsen et al., 2017 2 w after del 5 y 371 0.03 [-0.05; 0.12] 0.7% 0.7%
Manzano-Salgado et al., 2017 1st tr gest 7 y 1086 -0.06 [-0.20; 0.08] 0.2% 0.2%
Vrijheid et al., 2020 during gest 6-11 years 1301 -0.02 [-0.21; 0.17] 0.1% 0.1%
Common effect model -0.00 [-0.01; 0.01] 100.0% --
Random effects model -0.00 [-0.01; 0.01] -- 100.0%
Heterogeneity: I^2 = 0%, τ^2 < 0.0001, p = 0.79
-0.5
0
0.5

Study	Sampling	Outcome	Subjects	Estimate [95% CI]
Zhang, Lei, et al., 2022	within 3 d before del	7 y	206	0.021 [-0.055; 0.096]
Bloom et al., 2022	8-13 w gest	4-8 y	661	-0.032 [-0.092; 0.027]
Gyllenhammar et al., 2018	3 w after del	5 y	182	0.015 [-0.023; 0.054]
Zhang, Pan, et al., 2022	15 w gest	3, 42, 182.6, and 365 d	2395	-0.011 [-0.122; 0.101]
Liu et al., 2020	del	12 y	212	0.384 [-0.091; 0.859]
Mora et al., 2017	9.6 w gest	7.7 y	871	0.005 [-0.014; 0.024]
Jensen et al., 2020	before 16 w gest	3 m, 18 m	593	0.040 [-0.160; 0.240]
Chen et al., 2019	del	5 y	404	-0.001 [-0.009; 0.006]
Karlsen et al., 2017	2 w after del	5 y	371	0.031 [-0.054; 0.117]
Manzano-Salgado et al., 2017	1st tr gest	7 y	1086	-0.056 [-0.196; 0.084]
Vrijheid et al., 2020	during gest	6-11 years	1301	-0.020 [-0.211; 0.170]
**Summary**				**-0.001 [-0.052; 0.051]**

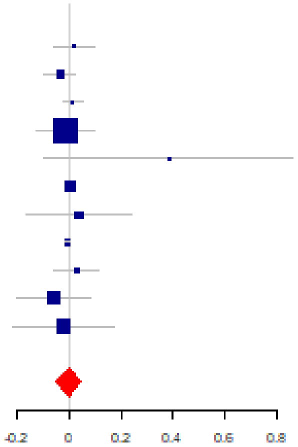

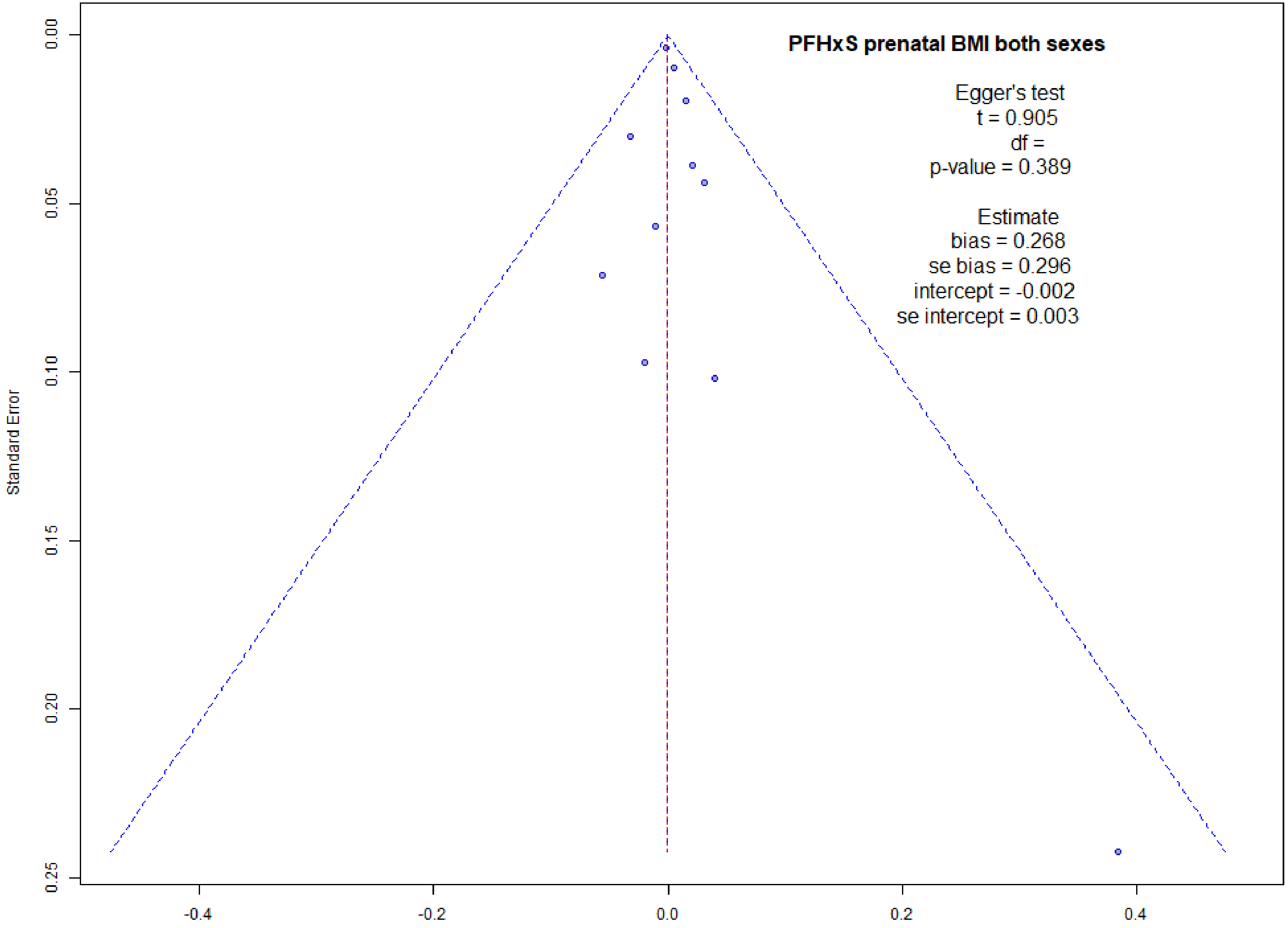
PFHxS prenatal BMI both sexes
Egger's test
t = 0.905
df =
p-value = 0.389
Estimate
bias = 0.268
se bias = 0.296
intercept = -0.002
se intercept = 0.003
Standard Error
0.00
0.05
0.10
0.15
0.20
0.25
-0.4
-0.2
0.0
0.2
0.4

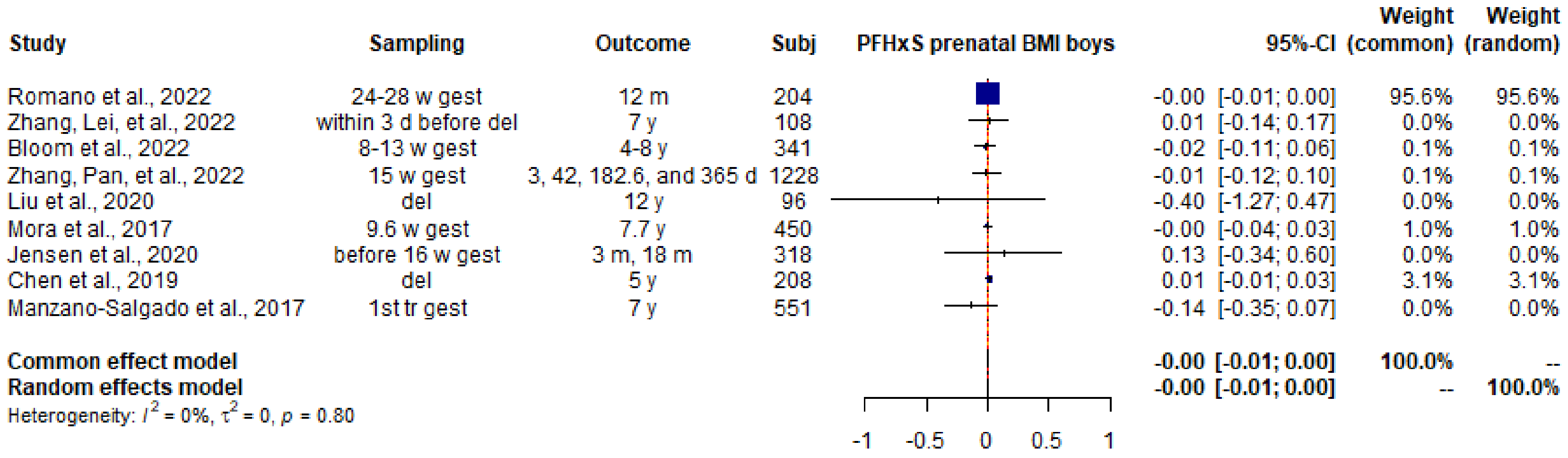
Study | Sampling | Outcome | Subj | PFHxS prenatal BMI boys | 95%-CI | Weight (common) | Weight (random)
Romano et al., 2022 | 24-28 w gest | 12 m | 204 | -0.00 [-0.01; 0.00] | 95.6% | 95.6%
Zhang, Lei, et al., 2022 | within 3 d before del | 7 y | 108 | 0.01 [-0.14; 0.17] | 0.0% | 0.0%
Bloom et al., 2022 | 8-13 w gest | 4-8 y | 341 | -0.02 [-0.11; 0.06] | 0.1% | 0.1%
Zhang, Pan, et al., 2022 | 15 w gest | 3, 42, 182.6, and 365 d | 1228 | -0.01 [-0.12; 0.10] | 0.1% | 0.1%
Liu et al., 2020 | del | 12 y | 96 | -0.40 [-1.27; 0.47] | 0.0% | 0.0%
Mora et al., 2017 | 9.6 w gest | 7.7 y | 450 | -0.00 [-0.04; 0.03] | 1.0% | 1.0%
Jensen et al., 2020 | before 16 w gest | 3 m, 18 m | 318 | 0.13 [-0.34; 0.60] | 0.0% | 0.0%
Chen et al., 2019 | del | 5 y | 208 | 0.01 [-0.01; 0.03] | 3.1% | 3.1%
Manzano-Salgado et al., 2017 | 1st tr gest | 7 y | 551 | -0.14 [-0.35; 0.07] | 0.0% | 0.0%
Common effect model | -0.00 [-0.01; 0.00] | 100.0% | --
Random effects model | -0.00 [-0.01; 0.00] | -- | 100.0%
Heterogeneity: I^2 = 0%, τ^2 = 0, p = 0.80
-1 -0.5 0 0.5 1

Study	Sampling	Outcome	Subjects	Estimate [95% CI]
Romano et al., 2022	24-28 w gest	12 m	204	-0.002 [-0.005; 0.001]
Zhang, Lei, et al., 2022	within 3 d before del	7 y	108	0.014 [-0.143; 0.172]
Bloom et al., 2022	8-13 w gest	4-8 y	341	-0.022 [-0.105; 0.062]
Zhang, Pan, et al., 2022	15 w gest	3, 42, 182.6, and 365 d	1228	-0.011 [-0.122; 0.101]
Liu et al., 2020	del	12 y	96	-0.401 [-1.274; 0.472]
Mora et al., 2017	9.6 w gest	7.7 y	450	-0.005 [-0.036; 0.026]
Jensen et al., 2020	before 16 w gest	3 m, 18 m	318	0.130 [-0.340; 0.600]
Chen et al., 2019	del	5 y	208	0.009 [-0.008; 0.026]
Manzano-Salgado et al., 2017	1st tr gest	7 y	551	-0.140 [-0.350; 0.070]
**Summary**				**-0.027 [-0.098; 0.045]**

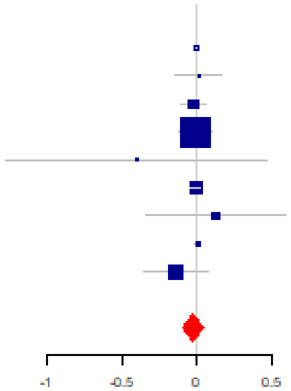

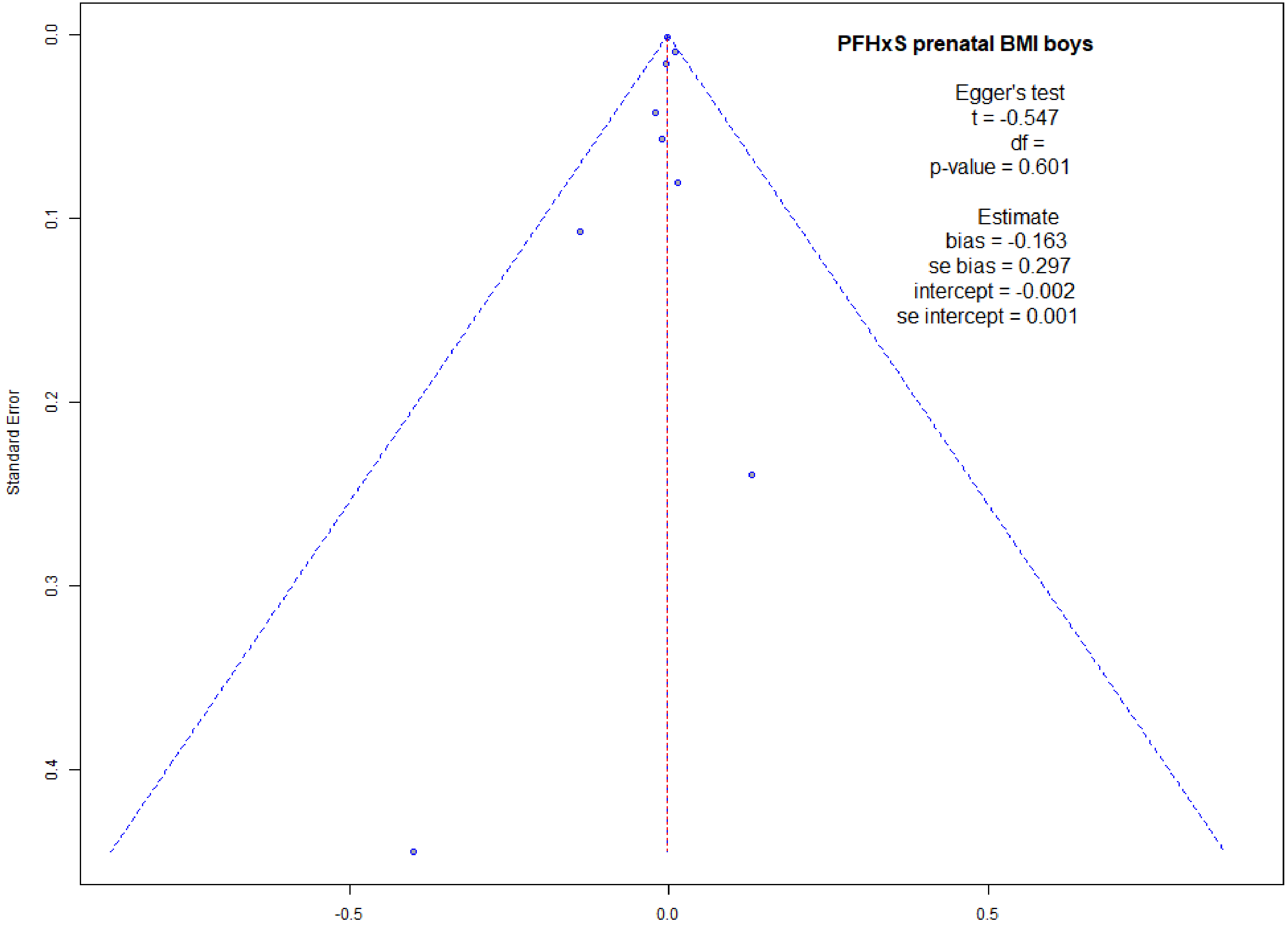
PFHxS prenatal BMI boys
Egger's test
t = -0.547
df =
p-value = 0.601
Estimate
bias = -0.163
se bias = 0.297
intercept = -0.002
se intercept = 0.001
Standard Error
0.0
0.1
0.2
0.3
0.4
-0.5
0.0
0.5

Study	Sampling	Outcome	Subj	95%-CI	Weight (common)	Weight (random)
Romano et al., 2022	24-28 w gest	12 m	214	0.01 [-0.01; 0.03]	1.2%	3.2%
Zhang, Lei, et al., 2022	within 3 d before del	7 y	98	0.02 [-0.03; 0.07]	0.2%	0.5%
Bloom et al., 2022	8-13 w gest	4-8 y	320	-0.04 [-0.12; 0.04]	0.1%	0.2%
Zhang, Pan, et al., 2022	15 w gest	3, 42, 182.6, and 365 d	1167	0.11 [-0.01; 0.22]	0.0%	0.1%
Hartman et al., 2017	15 w gest	9 y	311	-0.00 [-0.00; 0.00]	64.4%	52.5%
Liu et al., 2020	del	12 y	116	0.33 [-0.22; 0.88]	0.0%	0.0%
Mora et al., 2017	9.6 w gest	7.7 y	421	0.01 [-0.02; 0.04]	0.7%	1.8%
Jensen et al., 2020	before 16 w gest	3 m, 18 m	275	0.03 [-0.18; 0.24]	0.0%	0.0%
Chen et al., 2019	del	5 y	196	-0.00 [-0.01; 0.00]	33.5%	41.5%
Manzano-Salgado et al., 2017	1st tr gest	7 y	535	0.03 [-0.14; 0.20]	0.0%	0.0%
**Common effect model**				**-0.00 [-0.00; 0.00]**	**100.0%**	--
**Random effects model**				**-0.00 [-0.00; 0.00]**	--	**100.0%**

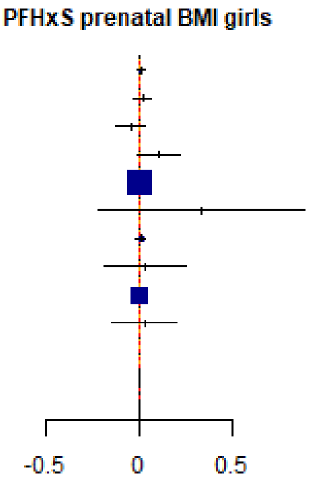

Heterogeneity: $I^2 = 0\%$, $\tau^2 < 0.0001$, $p = 0.46$

Study	Sampling	Outcome	Subjects	Estimate [95% CI]
Romano et al., 2022	24-28 w gest	12 m	214	0.007 [-0.012; 0.027]
Zhang, Lei, et al., 2022	within 3 d before del	7 y	98	0.016 [-0.034; 0.066]
Bloom et al., 2022	8-13 w gest	4-8 y	320	-0.043 [-0.121; 0.035]
Zhang, Pan, et al., 2022	15 w gest	3, 42, 182.6, and 365 d	1167	0.106 [-0.005; 0.217]
Hartman et al., 2017	15 w gest	9 y	311	-0.001 [-0.004; 0.002]
Liu et al., 2020	del	12 y	116	0.329 [-0.219; 0.878]
Mora et al., 2017	9.6 w gest	7.7 y	421	0.010 [-0.017; 0.036]
Jensen et al., 2020	before 16 w gest	3 m, 18 m	275	0.030 [-0.185; 0.245]
Chen et al., 2019	del	5 y	196	-0.003 [-0.006; 0.001]
Manzano-Salgado et al., 2017	1st tr gest	7 y	535	0.028 [-0.140; 0.196]
**Summary**				**0.049 [-0.001; 0.099]**

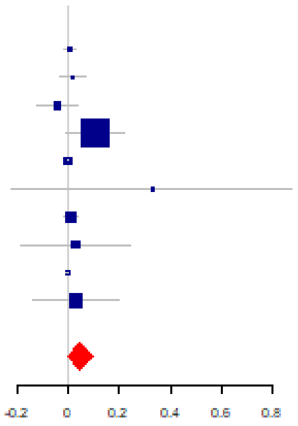

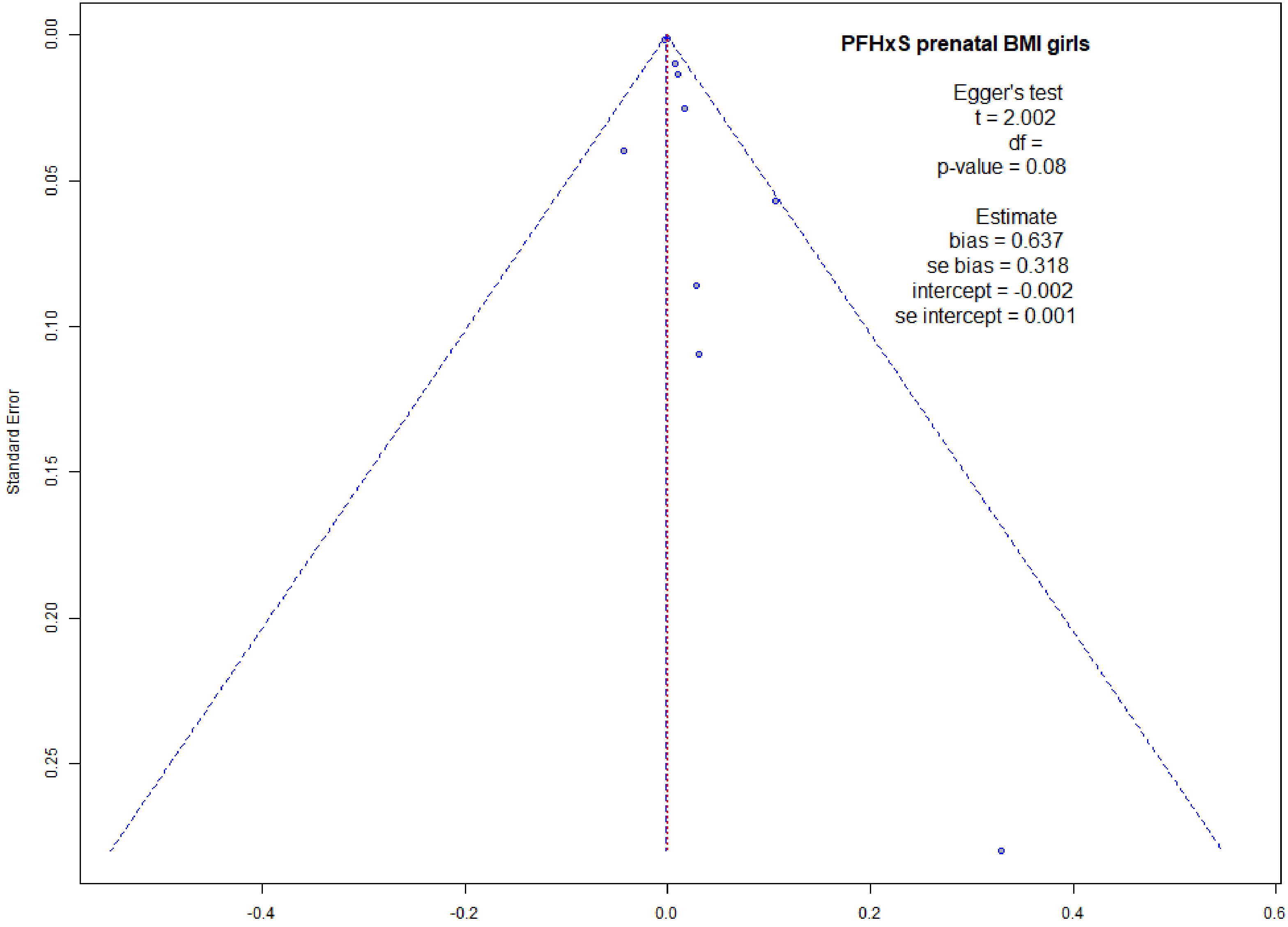
PFHxS prenatal BMI girls
Egger's test
t = 2.002
df =
p-value = 0.08
Estimate
bias = 0.637
se bias = 0.318
intercept = -0.002
se intercept = 0.001
Standard Error
0.00
0.05
0.10
0.15
0.20
0.25
-0.4
-0.2
0.0
0.2
0.4
0.6

Study	Sampling	Outcome	Subj	PFNA prenatal BMI both sexes	95%-CI	Weight (common)	Weight (random)
Zhang, Lei, et al., 2022	within 3 d before del	7 y	206	0.00	[-0.03; 0.03]	1.1%	1.1%
Bloom et al., 2022	8-13 w gest	4-8 y	661	-0.02	[-0.13; 0.09]	0.1%	0.1%
Gyllenhammar et al., 2018	3 w after del	5 y	183	0.42	[-0.12; 0.96]	0.0%	0.0%
Zhang, Pan, et al., 2022	15 w gest	3, 42, 182.6, and 365 d	2395	-0.02	[-0.06; 0.02]	0.6%	0.6%
Liu et al., 2020	del	12 y	212	0.42	[-1.17; 2.02]	0.0%	0.0%
Mora et al., 2017	9.6 w gest	7.7 y	871	0.12	[-0.04; 0.29]	0.0%	0.0%
Jensen et al., 2020	before 16 w gest	3 m, 18 m	593	0.18	[0.02; 0.34]	0.0%	0.0%
Chen et al., 2019	del	5 y	404	-0.00	[-0.00; 0.00]	97.8%	97.8%
Karlsen et al., 2017	2 w after del	5 y	371	0.00	[-0.08; 0.08]	0.1%	0.1%
Manzano-Salgado et al., 2017	1st tr gest	7 y	1086	0.08	[-0.05; 0.21]	0.1%	0.1%
Vrijheid et al., 2020	during gest	6-11 years	1301	0.00	[-0.19; 0.19]	0.0%	0.0%
**Common effect model**				**-0.00**	**[-0.00; 0.00]**	**100.0%**	--
**Random effects model**				**-0.00**	**[-0.00; 0.00]**	--	**100.0%**

Heterogeneity: $I^2 = 21\%$, $\tau^2 = 0$, $p = 0.24$

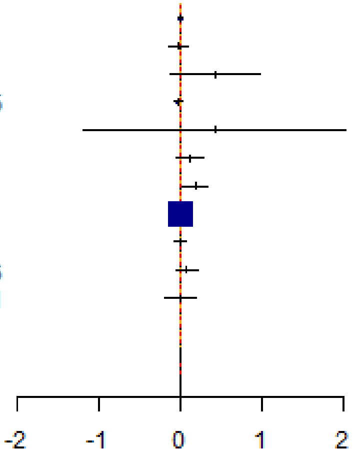

Study	Sampling	Outcome	Subjects	Estimate [95% CI]
Zhang, Lei, et al., 2022	within 3 d before del	7 y	206	0.001 [-0.029; 0.031]
Bloom et al., 2022	8-13 w gest	4-8 y	661	-0.023 [-0.133; 0.087]
Gyllenhammar et al., 2018	3 w after del	5 y	183	0.423 [-0.115; 0.962]
Zhang, Pan, et al., 2022	15 w gest	3, 42, 182.6, and 365 d	2395	-0.023 [-0.063; 0.016]
Liu et al., 2020	del	12 y	212	0.422 [-1.175; 2.018]
Mora et al., 2017	9.6 w gest	7.7 y	871	0.125 [-0.037; 0.288]
Jensen et al., 2020	before 16 w gest	3 m, 18 m	593	0.180 [0.020; 0.340]
Chen et al., 2019	del	5 y	404	0.000 [-0.003; 0.003]
Karlsen et al., 2017	2 w after del	5 y	371	0.000 [-0.082; 0.082]
Manzano-Salgado et al., 2017	1st tr gest	7 y	1086	0.080 [-0.053; 0.213]
Vrijheid et al., 2020	during gest	6-11 years	1301	0.000 [-0.188; 0.188]
**Summary**				**0.048 [-0.013; 0.109]**

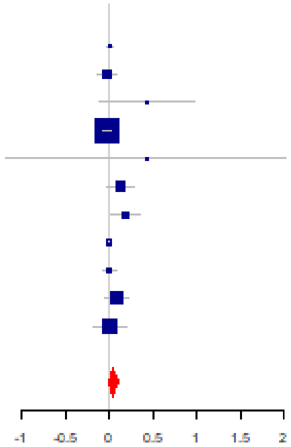

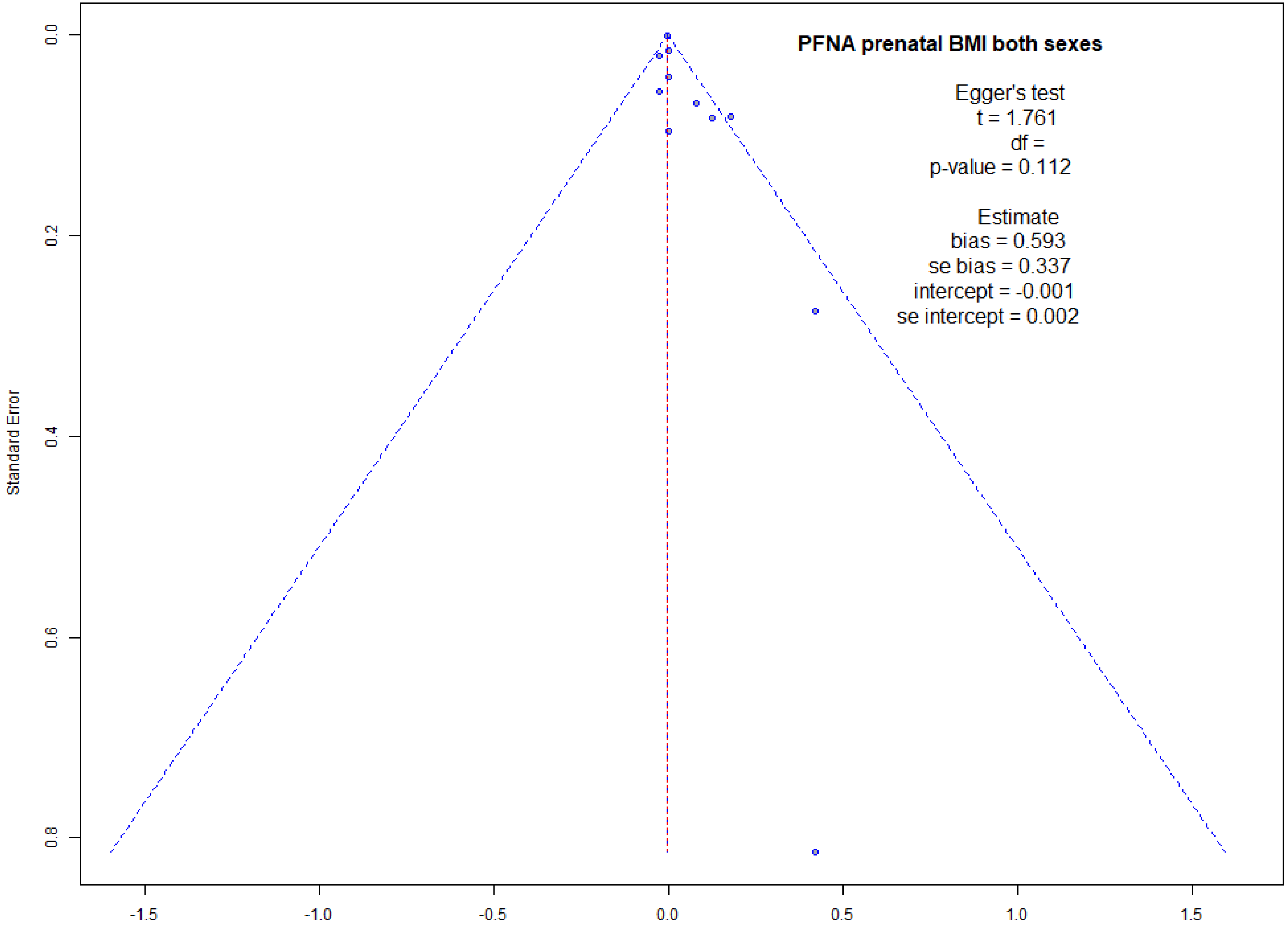
PFNA prenatal BMI both sexes
Egger's test
t = 1.761
df =
p-value = 0.112
Estimate
bias = 0.593
se bias = 0.337
intercept = -0.001
se intercept = 0.002
Standard Error
0.0
0.2
0.4
0.6
0.8
-1.5
-1.0
-0.5
0.0
0.5
1.0
1.5

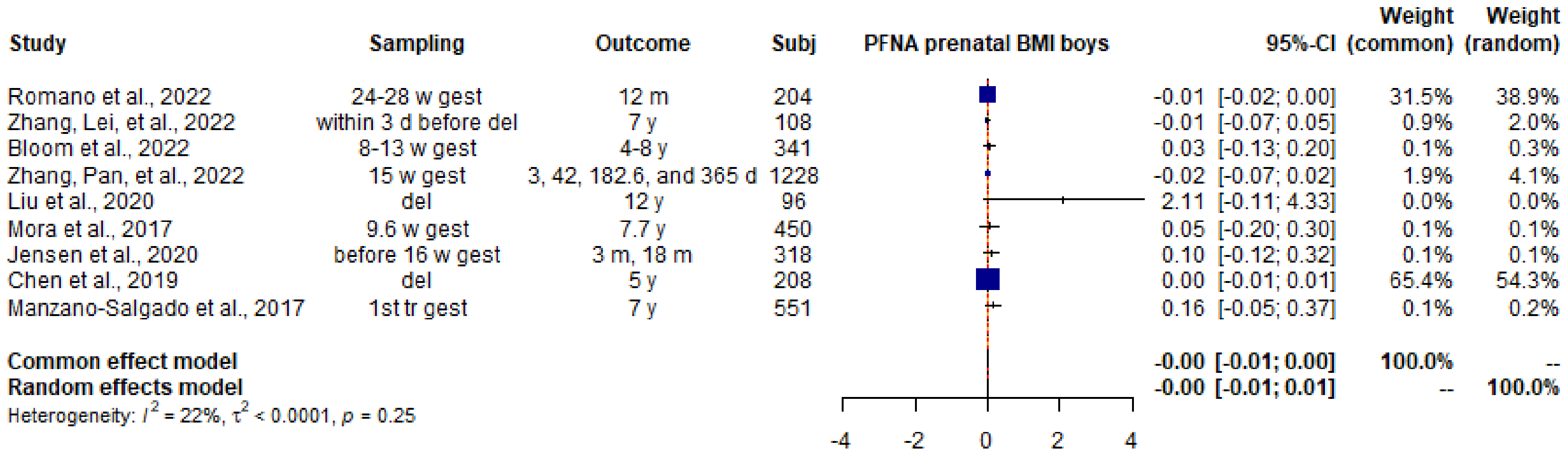
Study
Sampling
Outcome
Subj
PFNA prenatal BMI boys
95%-CI
Weight (common)
Weight (random)
Romano et al., 2022 24-28 w gest 12 m 204 -0.01 [-0.02; 0.00] 31.5% 38.9%
Zhang, Lei, et al., 2022 within 3 d before del 7 y 108 -0.01 [-0.07; 0.05] 0.9% 2.0%
Bloom et al., 2022 8-13 w gest 4-8 y 341 0.03 [-0.13; 0.20] 0.1% 0.3%
Zhang, Pan, et al., 2022 15 w gest 3, 42, 182.6, and 365 d 1228 -0.02 [-0.07; 0.02] 1.9% 4.1%
Liu et al., 2020 del 12 y 96 2.11 [-0.11; 4.33] 0.0% 0.0%
Mora et al., 2017 9.6 w gest 7.7 y 450 0.05 [-0.20; 0.30] 0.1% 0.1%
Jensen et al., 2020 before 16 w gest 3 m, 18 m 318 0.10 [-0.12; 0.32] 0.1% 0.1%
Chen et al., 2019 del 5 y 208 0.00 [-0.01; 0.01] 65.4% 54.3%
Manzano-Salgado et al., 2017 1st tr gest 7 y 551 0.16 [-0.05; 0.37] 0.1% 0.2%
Common effect model -0.00 [-0.01; 0.00] 100.0% --
Random effects model -0.00 [-0.01; 0.01] -- 100.0%
Heterogeneity: I^2 = 22%, τ^2 < 0.0001, p = 0.25
-4
-2
0
2
4

Study	Sampling	Outcome	Subjects	Estimate [95% CI]
Romano et al., 2022	24-28 w gest	12 m	204	-0.008 [-0.018; 0.003]
Zhang, Lei, et al., 2022	within 3 d before del	7 y	108	-0.010 [-0.070; 0.051]
Bloom et al., 2022	8-13 w gest	4-8 y	341	0.035 [-0.127; 0.196]
Zhang, Pan, et al., 2022	15 w gest	3, 42, 182.6, and 365 d	1228	-0.023 [-0.065; 0.019]
Liu et al., 2020	del	12 y	96	2.113 [-0.106; 4.332]
Mora et al., 2017	9.6 w gest	7.7 y	450	0.050 [-0.200; 0.300]
Jensen et al., 2020	before 16 w gest	3 m, 18 m	318	0.100 [-0.125; 0.325]
Chen et al., 2019	del	5 y	208	0.002 [-0.005; 0.009]
Manzano-Salgado et al., 2017	1st tr gest	7 y	551	0.160 [-0.053; 0.373]
**Summary**				**0.093 [0.011; 0.175]**

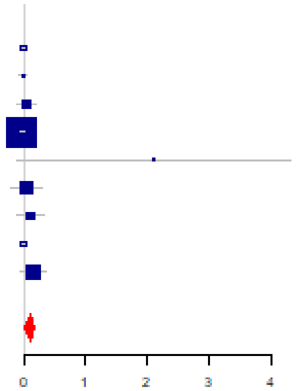

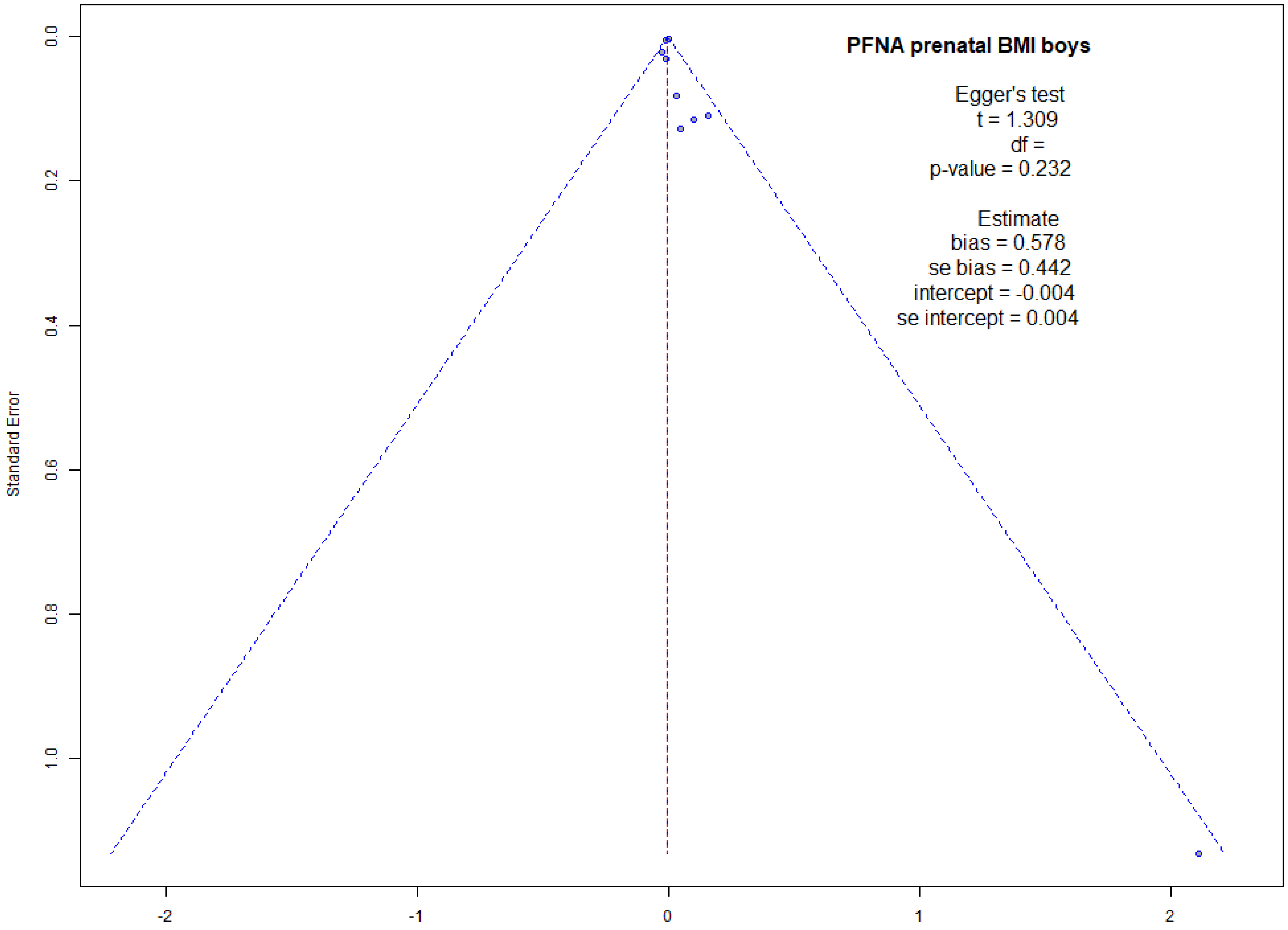
PFNA prenatal BMI boys
Egger's test
t = 1.309
df =
p-value = 0.232
Estimate
bias = 0.578
se bias = 0.442
intercept = -0.004
se intercept = 0.004
Standard Error
0.0
0.2
0.4
0.6
0.8
1.0
-2
-1
0
1
2

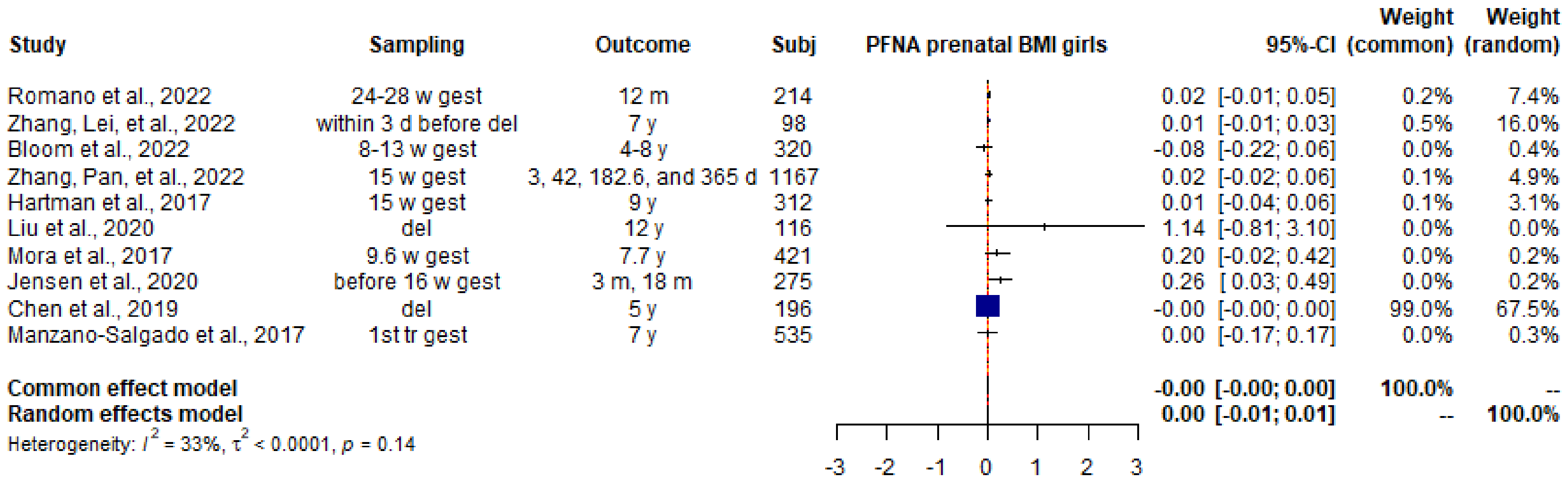
Study | Sampling | Outcome | Subj | PFNA prenatal BMI girls | 95%-CI | Weight (common) | Weight (random)
Romano et al., 2022 | 24-28 w gest | 12 m | 214 | 0.02 [-0.01; 0.05] | 0.2% | 7.4%
Zhang, Lei, et al., 2022 | within 3 d before del | 7 y | 98 | 0.01 [-0.01; 0.03] | 0.5% | 16.0%
Bloom et al., 2022 | 8-13 w gest | 4-8 y | 320 | -0.08 [-0.22; 0.06] | 0.0% | 0.4%
Zhang, Pan, et al., 2022 | 15 w gest | 3, 42, 182.6, and 365 d | 1167 | 0.02 [-0.02; 0.06] | 0.1% | 4.9%
Hartman et al., 2017 | 15 w gest | 9 y | 312 | 0.01 [-0.04; 0.06] | 0.1% | 3.1%
Liu et al., 2020 | del | 12 y | 116 | 1.14 [-0.81; 3.10] | 0.0% | 0.0%
Mora et al., 2017 | 9.6 w gest | 7.7 y | 421 | 0.20 [-0.02; 0.42] | 0.0% | 0.2%
Jensen et al., 2020 | before 16 w gest | 3 m, 18 m | 275 | 0.26 [0.03; 0.49] | 0.0% | 0.2%
Chen et al., 2019 | del | 5 y | 196 | -0.00 [-0.00; 0.00] | 99.0% | 67.5%
Manzano-Salgado et al., 2017 | 1st tr gest | 7 y | 535 | 0.00 [-0.17; 0.17] | 0.0% | 0.3%
Common effect model | -0.00 [-0.00; 0.00] | 100.0% | --
Random effects model | 0.00 [-0.01; 0.01] | -- | 100.0%
Heterogeneity: I² = 33%, τ² < 0.0001, p = 0.14
-3 -2 -1 0 1 2 3

Study	Sampling	Outcome	Subjects	Estimate [95% CI]
Romano et al., 2022	24-28 w gest	12 m	214	0.019 [-0.013; 0.051]
Zhang, Lei, et al., 2022	within 3 d before del	7 y	98	0.007 [-0.013; 0.027]
Bloom et al., 2022	8-13 w gest	4-8 y	320	-0.081 [-0.219; 0.058]
Zhang, Pan, et al., 2022	15 w gest	3, 42, 182.6, and 365 d	1167	0.019 [-0.021; 0.058]
Hartman et al., 2017	15 w gest	9 y	312	0.011 [-0.040; 0.061]
Liu et al., 2020	del	12 y	116	1.145 [-0.813; 3.103]
Mora et al., 2017	9.6 w gest	7.7 y	421	0.200 [-0.025; 0.425]
Jensen et al., 2020	before 16 w gest	3 m, 18 m	275	0.260 [0.030; 0.490]
Chen et al., 2019	del	5 y	196	0.000 [-0.002; 0.001]
Manzano-Salgado et al., 2017	1st tr gest	7 y	535	0.000 [-0.166; 0.166]
**Summary**				**0.080 [0.004; 0.156]**

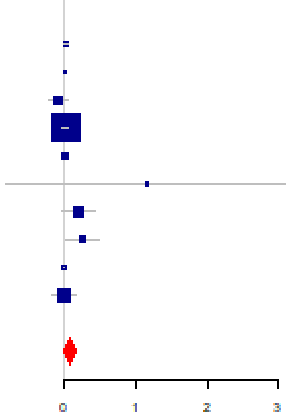

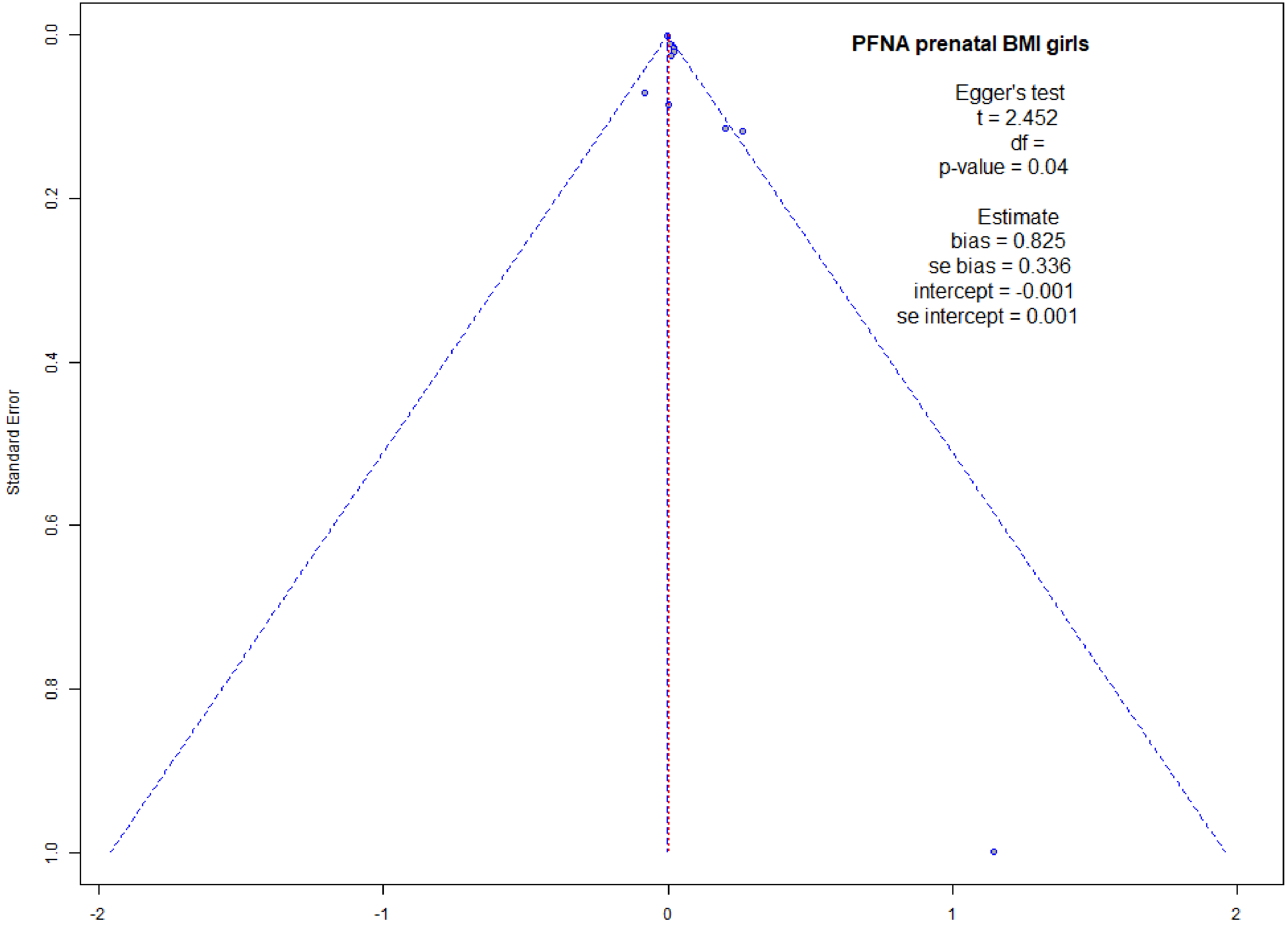
PFNA prenatal BMI girls
Egger's test
t = 2.452
df =
p-value = 0.04
Estimate
bias = 0.825
se bias = 0.336
intercept = -0.001
se intercept = 0.001
Standard Error
0.0
0.2
0.4
0.6
0.8
1.0
-2
-1
0
1
2

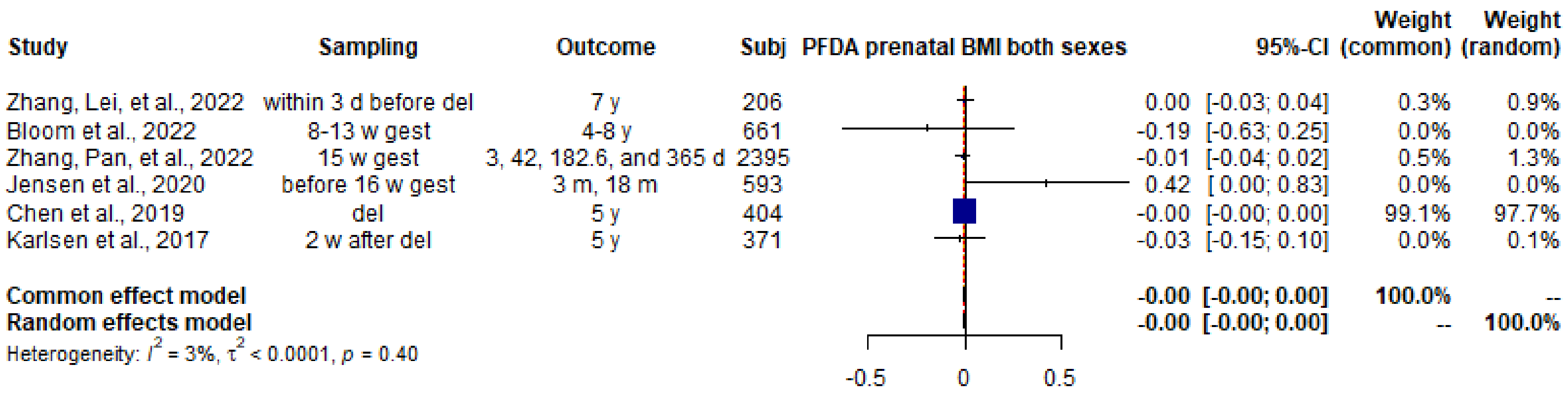
Study
Sampling
Outcome
Subj
PFDA prenatal BMI both sexes
95%-CI
Weight (common)
Weight (random)
Zhang, Lei, et al., 2022 within 3 d before del 7 y 206 0.00 [-0.03; 0.04] 0.3% 0.9%
Bloom et al., 2022 8-13 w gest 4-8 y 661 -0.19 [-0.63; 0.25] 0.0% 0.0%
Zhang, Pan, et al., 2022 15 w gest 3, 42, 182.6, and 365 d 2395 -0.01 [-0.04; 0.02] 0.5% 1.3%
Jensen et al., 2020 before 16 w gest 3 m, 18 m 593 0.42 [0.00; 0.83] 0.0% 0.0%
Chen et al., 2019 del 5 y 404 -0.00 [-0.00; 0.00] 99.1% 97.7%
Karlsen et al., 2017 2 w after del 5 y 371 -0.03 [-0.15; 0.10] 0.0% 0.1%
Common effect model -0.00 [-0.00; 0.00] 100.0% --
Random effects model -0.00 [-0.00; 0.00] -- 100.0%
Heterogeneity: I^2 = 3%, τ^2 < 0.0001, p = 0.40
-0.5
0
0.5

Study	Sampling	Outcome	Subjects	Estimate [95% CI]
Zhang, Lei, et al., 2022	within 3 d before del	7 y	206	0.004 [-0.035; 0.042]
Bloom et al., 2022	8-13 w gest	4-8 y	661	-0.188 [-0.626; 0.251]
Zhang, Pan, et al., 2022	15 w gest	3, 42, 182.6, and 365 d	2395	-0.009 [-0.041; 0.023]
Jensen et al., 2020	before 16 w gest	3 m, 18 m	593	0.420 [0.005; 0.835]
Chen et al., 2019	del	5 y	404	-0.001 [-0.003; 0.002]
Karlsen et al., 2017	2 w after del	5 y	371	-0.027 [-0.154; 0.099]
**Summary**				**0.020 [-0.064; 0.105]**

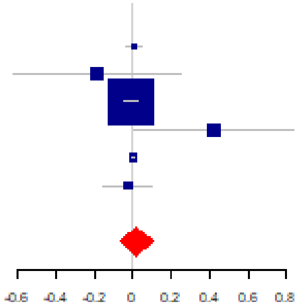

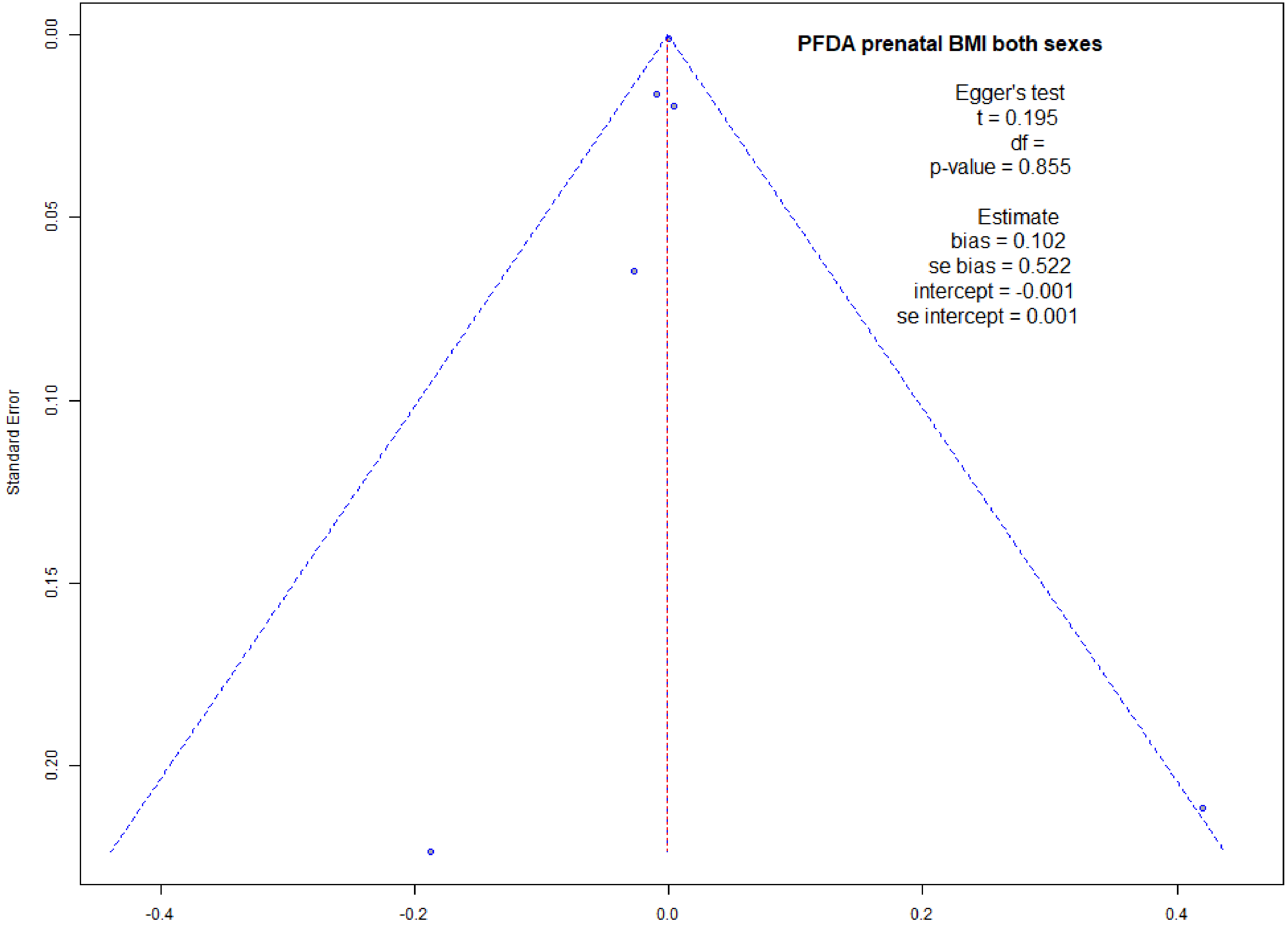
PFDA prenatal BMI both sexes
Egger's test
t = 0.195
df =
p-value = 0.855
Estimate
bias = 0.102
se bias = 0.522
intercept = -0.001
se intercept = 0.001
Standard Error
0.00
0.05
0.10
0.15
0.20
-0.4
-0.2
0.0
0.2
0.4

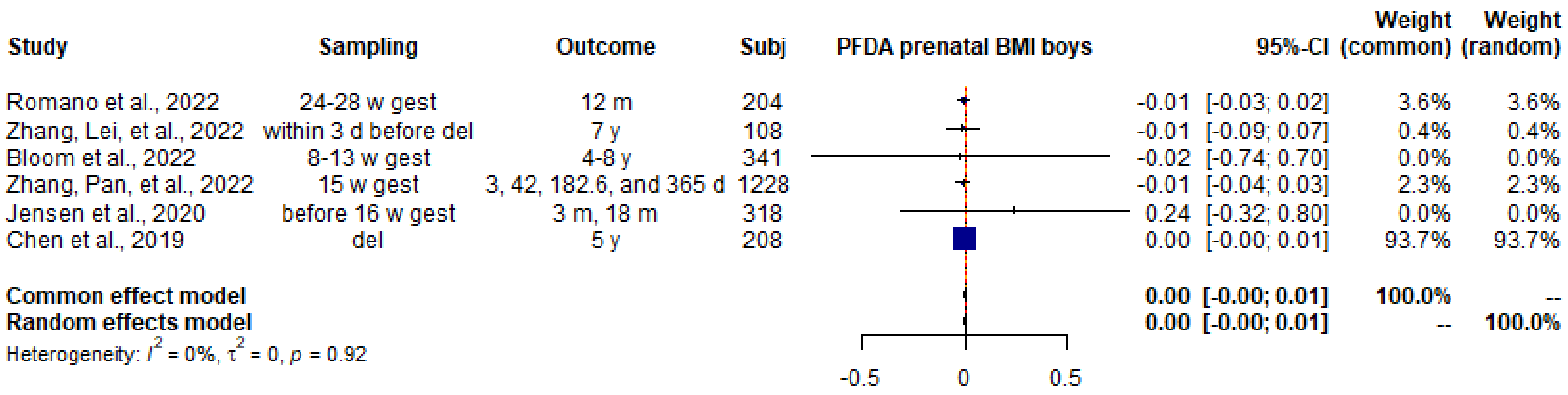
Study
Sampling
Outcome
Subj
PFDA prenatal BMI boys
95%-CI
Weight (common)
Weight (random)
Romano et al., 2022 24-28 w gest 12 m 204 -0.01 [-0.03; 0.02] 3.6% 3.6%
Zhang, Lei, et al., 2022 within 3 d before del 7 y 108 -0.01 [-0.09; 0.07] 0.4% 0.4%
Bloom et al., 2022 8-13 w gest 4-8 y 341 -0.02 [-0.74; 0.70] 0.0% 0.0%
Zhang, Pan, et al., 2022 15 w gest 3, 42, 182.6, and 365 d 1228 -0.01 [-0.04; 0.03] 2.3% 2.3%
Jensen et al., 2020 before 16 w gest 3 m, 18 m 318 0.24 [-0.32; 0.80] 0.0% 0.0%
Chen et al., 2019 del 5 y 208 0.00 [-0.00; 0.01] 93.7% 93.7%
Common effect model 0.00 [-0.00; 0.01] 100.0% --
Random effects model 0.00 [-0.00; 0.01] -- 100.0%
Heterogeneity: I^2 = 0%, τ^2 = 0, p = 0.92
-0.5
0
0.5

Study	Sampling	Outcome	Subjects	Estimate [95% CI]
Romano et al., 2022	24-28 w gest	12 m	204	-0.006 [-0.034; 0.022]
Zhang, Lei, et al., 2022	within 3 d before del	7 y	108	-0.011 [-0.091; 0.069]
Bloom et al., 2022	8-13 w gest	4-8 y	341	-0.019 [-0.739; 0.702]
Zhang, Pan, et al., 2022	15 w gest	3, 42, 182.6, and 365 d	1228	-0.009 [-0.044; 0.025]
Jensen et al., 2020	before 16 w gest	3 m, 18 m	318	0.240 [-0.315; 0.795]
Chen et al., 2019	del	5 y	208	0.002 [-0.004; 0.007]
**Summary**				**0.023 [-0.103; 0.150]**

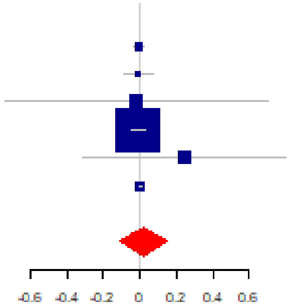

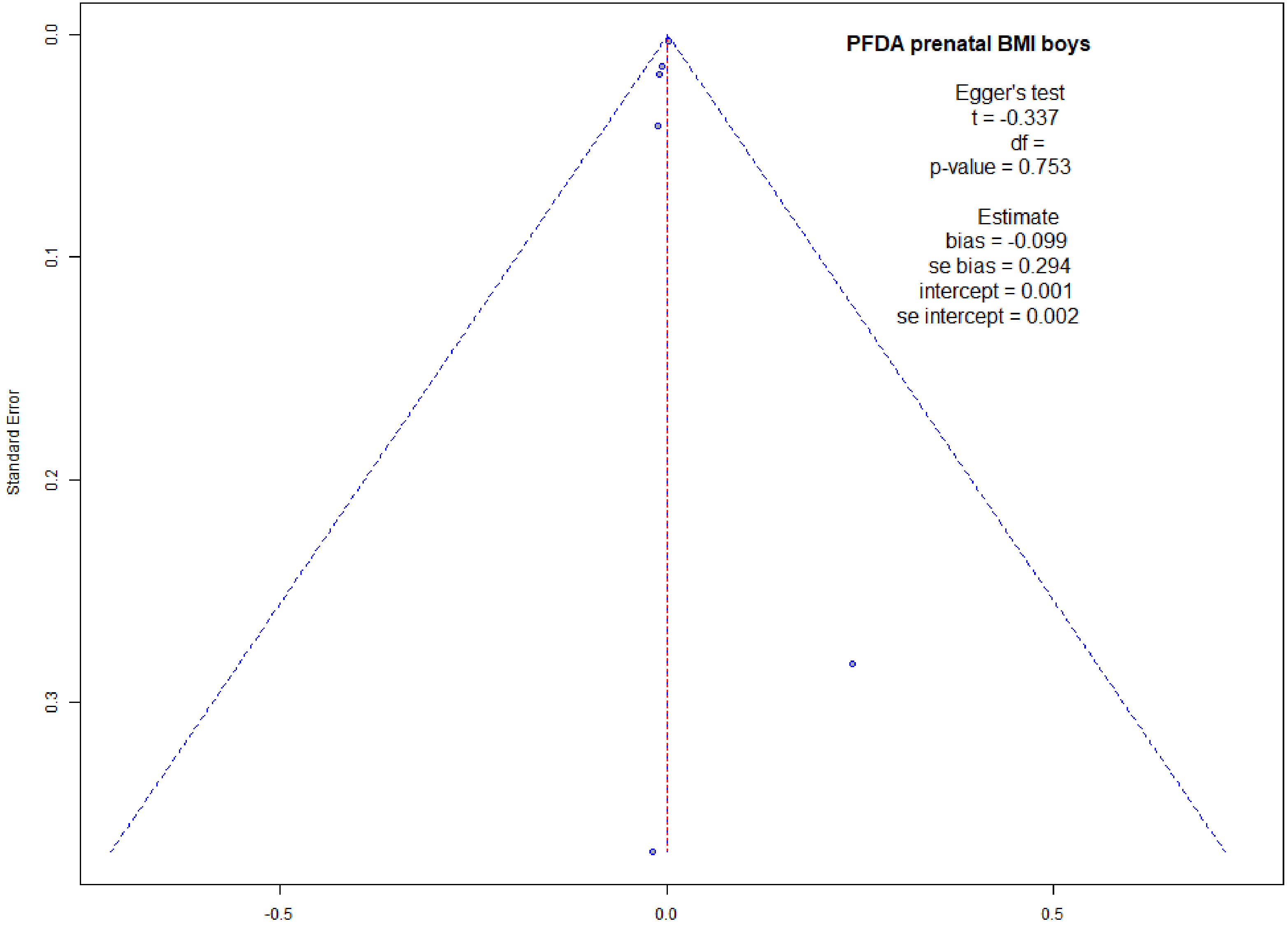
PFDA prenatal BMI boys
Egger's test
t = -0.337
df =
p-value = 0.753
Estimate
bias = -0.099
se bias = 0.294
intercept = 0.001
se intercept = 0.002
Standard Error
0.0
0.1
0.2
0.3
-0.5
0.0
0.5

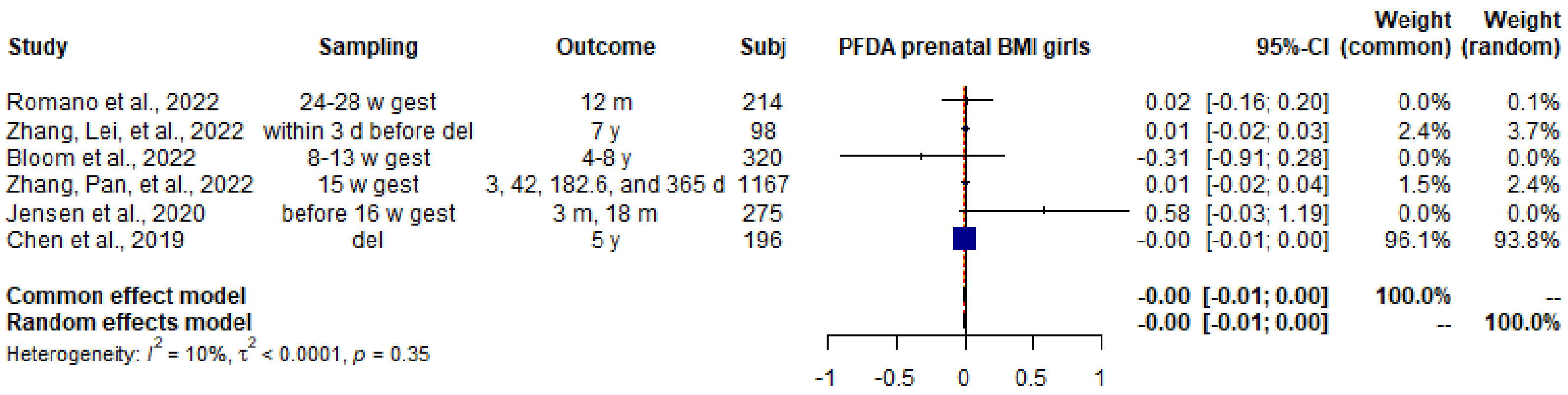
Study
Sampling
Outcome
Subj
PFDA prenatal BMI girls
95%-CI
Weight (common)
Weight (random)
Romano et al., 2022 | 24-28 w gest | 12 m | 214 | 0.02 [-0.16; 0.20] | 0.0% | 0.1%
Zhang, Lei, et al., 2022 | within 3 d before del | 7 y | 98 | 0.01 [-0.02; 0.03] | 2.4% | 3.7%
Bloom et al., 2022 | 8-13 w gest | 4-8 y | 320 | -0.31 [-0.91; 0.28] | 0.0% | 0.0%
Zhang, Pan, et al., 2022 | 15 w gest | 3, 42, 182.6, and 365 d | 1167 | 0.01 [-0.02; 0.04] | 1.5% | 2.4%
Jensen et al., 2020 | before 16 w gest | 3 m, 18 m | 275 | 0.58 [-0.03; 1.19] | 0.0% | 0.0%
Chen et al., 2019 | del | 5 y | 196 | -0.00 [-0.01; 0.00] | 96.1% | 93.8%
Common effect model | -0.00 [-0.01; 0.00] | 100.0% | --
Random effects model | -0.00 [-0.01; 0.00] | -- | 100.0%
Heterogeneity: I^2 = 10%, τ^2 < 0.0001, p = 0.35
-1
-0.5
0
0.5
1

Study	Sampling	Outcome	Subjects	Estimate [95% CI]
Romano et al., 2022	24-28 w gest	12 m	214	0.021 [-0.156; 0.197]
Zhang, Lei, et al., 2022	within 3 d before del	7 y	98	0.007 [-0.018; 0.033]
Bloom et al., 2022	8-13 w gest	4-8 y	320	-0.313 [-0.908; 0.282]
Zhang, Pan, et al., 2022	15 w gest	3, 42, 182.6, and 365 d	1167	0.009 [-0.023; 0.041]
Jensen et al., 2020	before 16 w gest	3 m, 18 m	275	0.580 [-0.030; 1.190]
Chen et al., 2019	del	5 y	196	-0.002 [-0.006; 0.002]
**Summary**				**0.033 [-0.081; 0.147]**

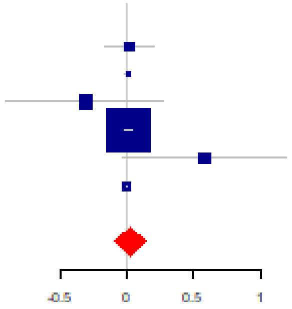

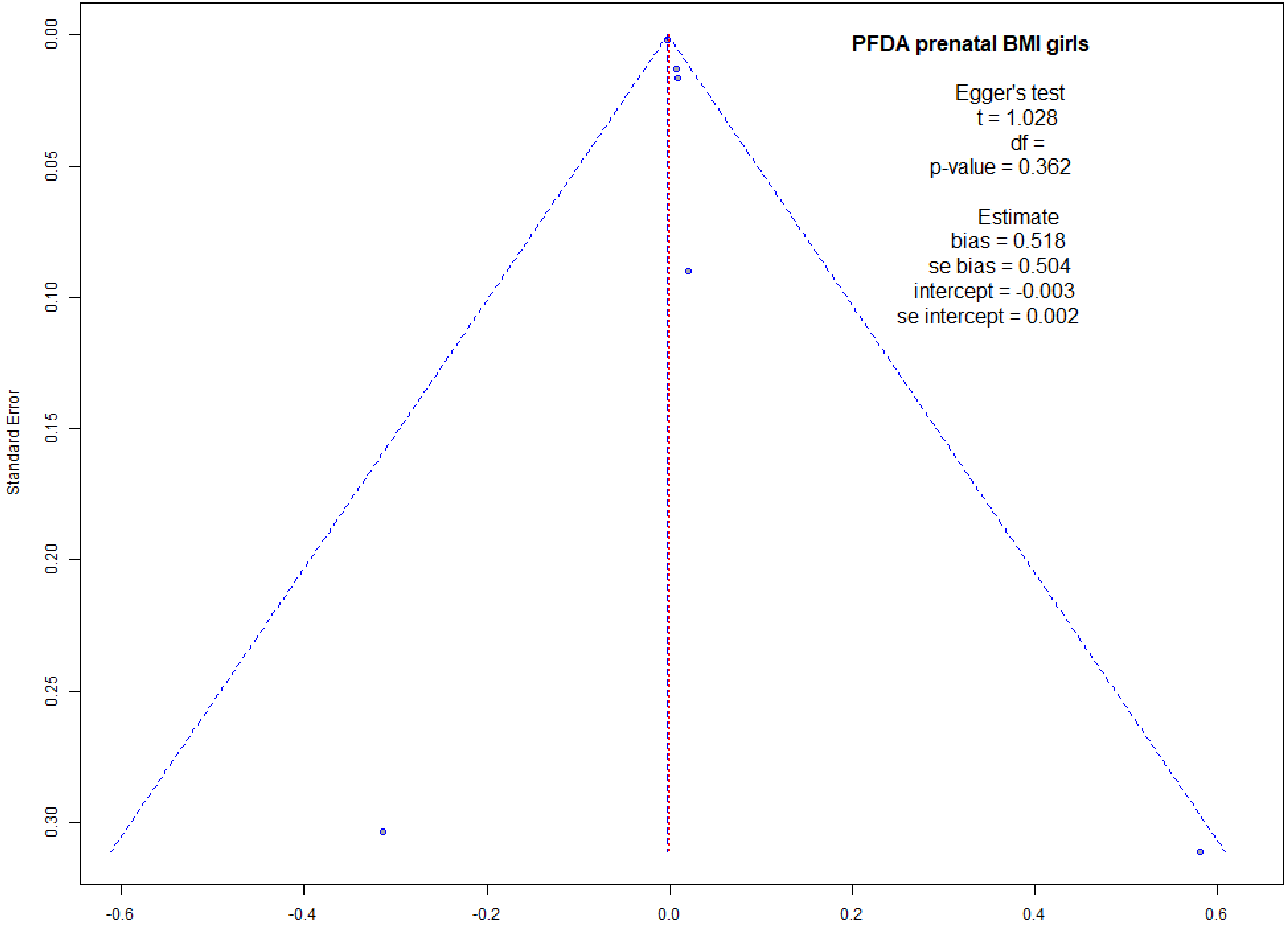
PFDA prenatal BMI girls
Egger's test
t = 1.028
df =
p-value = 0.362
Estimate
bias = 0.518
se bias = 0.504
intercept = -0.003
se intercept = 0.002
Standard Error
0.00
0.05
0.10
0.15
0.20
0.25
0.30
-0.6
-0.4
-0.2
0.0
0.2
0.4
0.6

Study	Sampling	Outcome	Subj	PFUA prenatal BMI both sexes	95%-CI	Weight (common)	Weight (random)
Zhang, Lei, et al., 2022	within 3 d before del	7 y	206	0.00	[-0.04; 0.05]	0.5%	0.5%
Bloom et al., 2022	8-13 w gest	4-8 y	661	0.09	[-0.54; 0.72]	0.0%	0.0%
Zhang, Pan, et al., 2022	15 w gest	3, 42, 182.6, and 365 d	2395	-0.01	[-0.04; 0.03]	0.7%	0.7%
Chen et al., 2019	del	5 y	404	-0.00	[-0.00; 0.00]	98.8%	98.8%
Vrijheid et al., 2020	during gest	6-11 years	1301	-0.82	[-2.47; 0.82]	0.0%	0.0%
**Common effect model**				**-0.00**	**[-0.00; 0.00]**	**100.0%**	--
**Random effects model**				**-0.00**	**[-0.00; 0.00]**	--	**100.0%**

Heterogeneity: $I^2 = 0\%$, $\tau^2 = 0$, $p = 0.89$

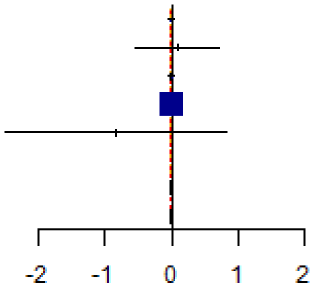

Study	Sampling	Outcome	Subjects	Estimate [95% CI]
Zhang, Lei, et al., 2022	within 3 d before del	7 y	206	0.005 [-0.041; 0.051]
Bloom et al., 2022	8-13 w gest	4-8 y	661	0.090 [-0.538; 0.717]
Zhang, Pan, et al., 2022	15 w gest	3, 42, 182.6, and 365 d	2395	-0.005 [-0.043; 0.032]
Chen et al., 2019	del	5 y	404	-0.002 [-0.005; 0.002]
Vrijheid et al., 2020	during gest	6-11 years	1301	-0.823 [-2.469; 0.823]
**Summary**				**-0.206 [-0.646; 0.233]**

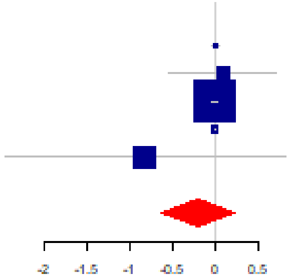

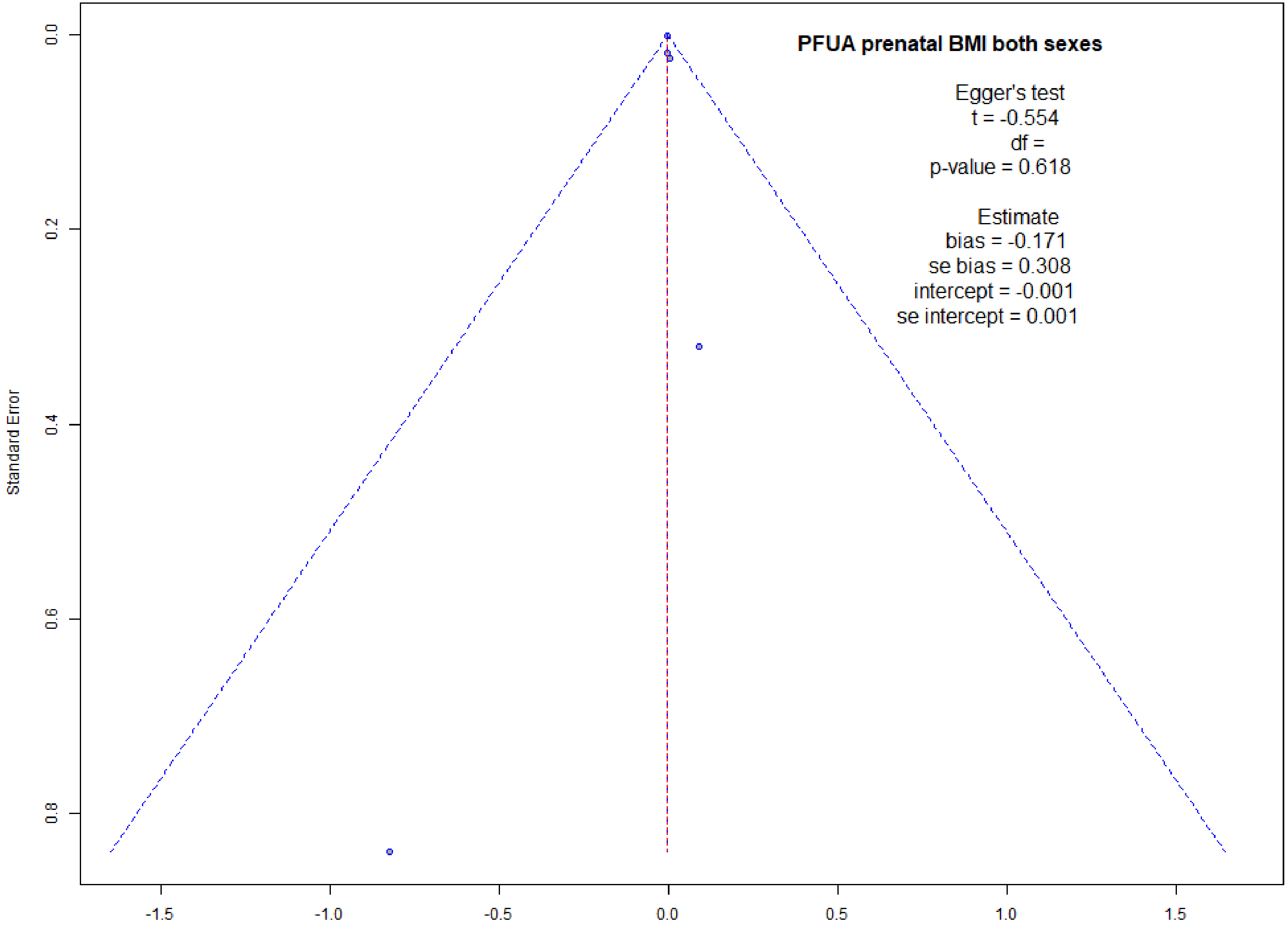
PFUA prenatal BMI both sexes
Egger's test
t = -0.554
df =
p-value = 0.618
Estimate
bias = -0.171
se bias = 0.308
intercept = -0.001
se intercept = 0.001
Standard Error
0.0
0.2
0.4
0.6
0.8
-1.5
-1.0
-0.5
0.0
0.5
1.0
1.5

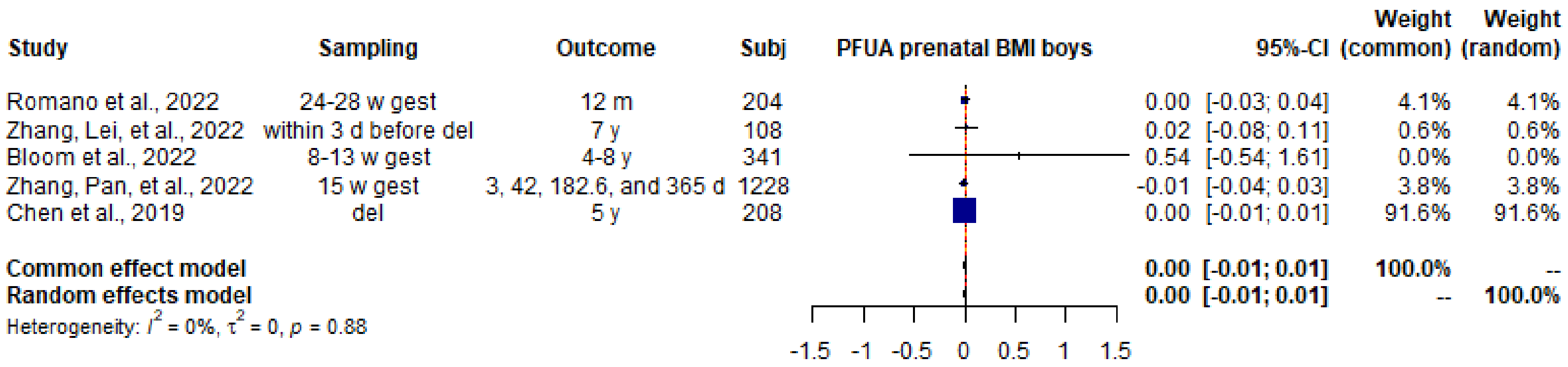
Study
Sampling
Outcome
Subj
PFUA prenatal BMI boys
95%-CI
Weight (common)
Weight (random)
Romano et al., 2022 | 24-28 w gest | 12 m | 204 | 0.00 [-0.03; 0.04] | 4.1% | 4.1%
Zhang, Lei, et al., 2022 | within 3 d before del | 7 y | 108 | 0.02 [-0.08; 0.11] | 0.6% | 0.6%
Bloom et al., 2022 | 8-13 w gest | 4-8 y | 341 | 0.54 [-0.54; 1.61] | 0.0% | 0.0%
Zhang, Pan, et al., 2022 | 15 w gest | 3, 42, 182.6, and 365 d | 1228 | -0.01 [-0.04; 0.03] | 3.8% | 3.8%
Chen et al., 2019 | del | 5 y | 208 | 0.00 [-0.01; 0.01] | 91.6% | 91.6%
Common effect model | 0.00 [-0.01; 0.01] | 100.0% | --
Random effects model | 0.00 [-0.01; 0.01] | -- | 100.0%
Heterogeneity: I^2 = 0%, τ^2 = 0, p = 0.88
-1.5 -1 -0.5 0 0.5 1 1.5

Study	Sampling	Outcome	Subjects	Estimate [95% CI]
Romano et al., 2022	24-28 w gest	12 m	204	0.003 [-0.033; 0.039]
Zhang, Lei, et al., 2022	within 3 d before del	7 y	108	0.019 [-0.077; 0.115]
Bloom et al., 2022	8-13 w gest	4-8 y	341	0.538 [-0.538; 1.613]
Zhang, Pan, et al., 2022	15 w gest	3, 42, 182.6, and 365 d	1228	-0.005 [-0.043; 0.032]
Chen et al., 2019	del	5 y	208	0.002 [-0.006; 0.010]
**Summary**				**0.086 [-0.091; 0.263]**

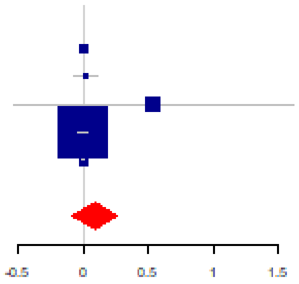

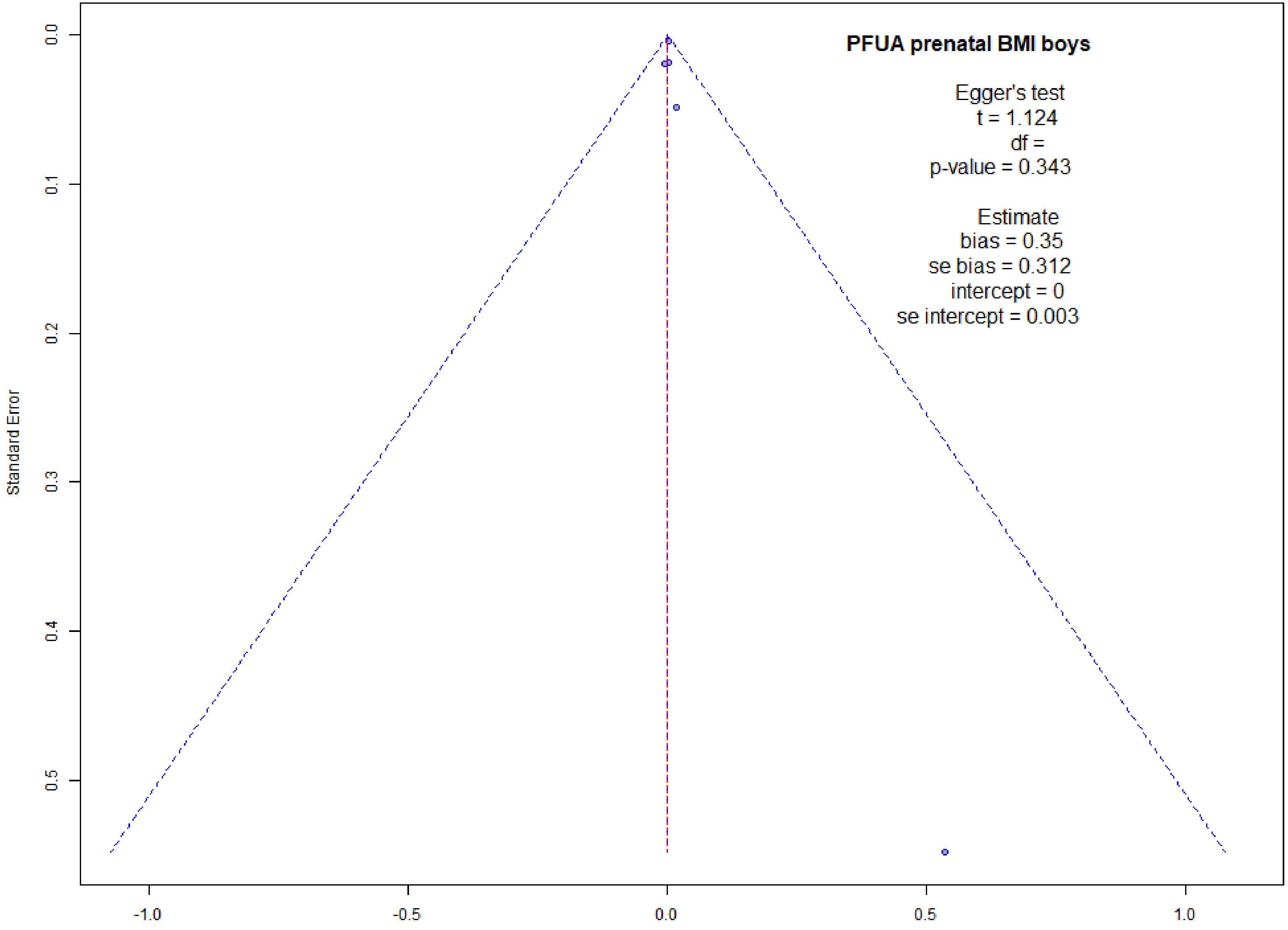
PFUA prenatal BMI boys
Egger's test
t = 1.124
df =
p-value = 0.343
Estimate
bias = 0.35
se bias = 0.312
intercept = 0
se intercept = 0.003
Standard Error
0.0
0.1
0.2
0.3
0.4
0.5
-1.0
-0.5
0.0
0.5
1.0

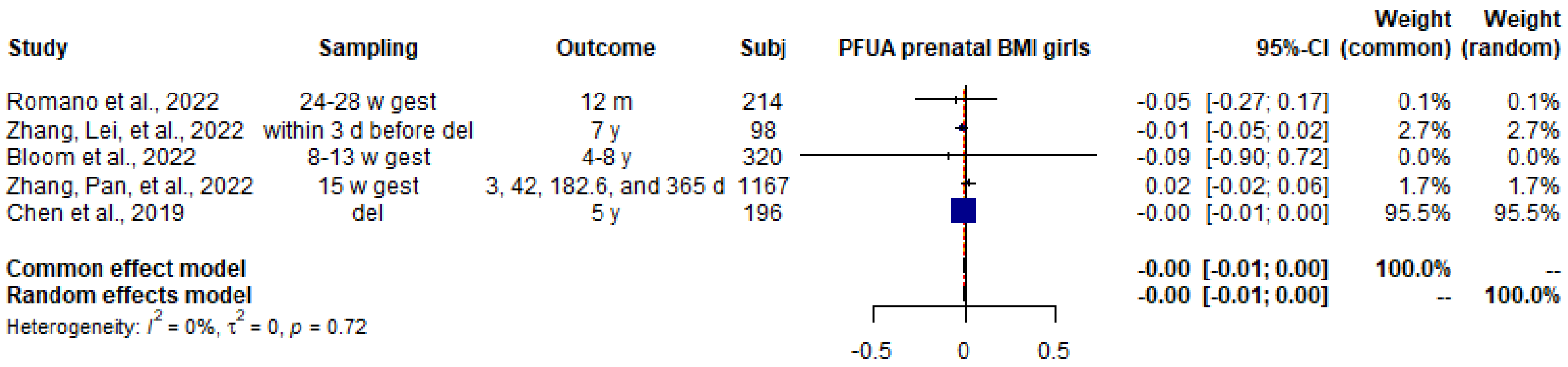
Study
Sampling
Outcome
Subj
PFUA prenatal BMI girls
95%-CI
Weight (common)
Weight (random)
Romano et al., 2022 | 24-28 w gest | 12 m | 214 | -0.05 | [-0.27; 0.17] | 0.1% | 0.1%
Zhang, Lei, et al., 2022 | within 3 d before del | 7 y | 98 | -0.01 | [-0.05; 0.02] | 2.7% | 2.7%
Bloom et al., 2022 | 8-13 w gest | 4-8 y | 320 | -0.09 | [-0.90; 0.72] | 0.0% | 0.0%
Zhang, Pan, et al., 2022 | 15 w gest | 3, 42, 182.6, and 365 d | 1167 | 0.02 | [-0.02; 0.06] | 1.7% | 1.7%
Chen et al., 2019 | del | 5 y | 196 | -0.00 | [-0.01; 0.00] | 95.5% | 95.5%
Common effect model | -0.00 | [-0.01; 0.00] | 100.0% | --
Random effects model | -0.00 | [-0.01; 0.00] | -- | 100.0%
Heterogeneity: $I^2 = 0\%$, $\tau^2 = 0$, $p = 0.72$
-0.5
0
0.5

Study	Sampling	Outcome	Subjects	Estimate [95% CI]
Romano et al., 2022	24-28 w gest	12 m	214	-0.050 [-0.269; 0.169]
Zhang, Lei, et al., 2022	within 3 d before del	7 y	98	-0.013 [-0.045; 0.019]
Bloom et al., 2022	8-13 w gest	4-8 y	320	-0.090 [-0.896; 0.717]
Zhang, Pan, et al., 2022	15 w gest	3, 42, 182.6, and 365 d	1167	0.022 [-0.019; 0.062]
Chen et al., 2019	del	5 y	196	-0.004 [-0.010; 0.001]
**Summary**				**-0.008 [-0.142; 0.125]**

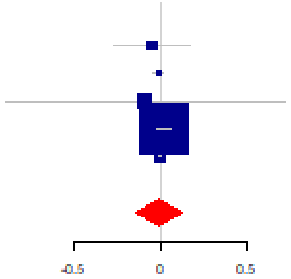

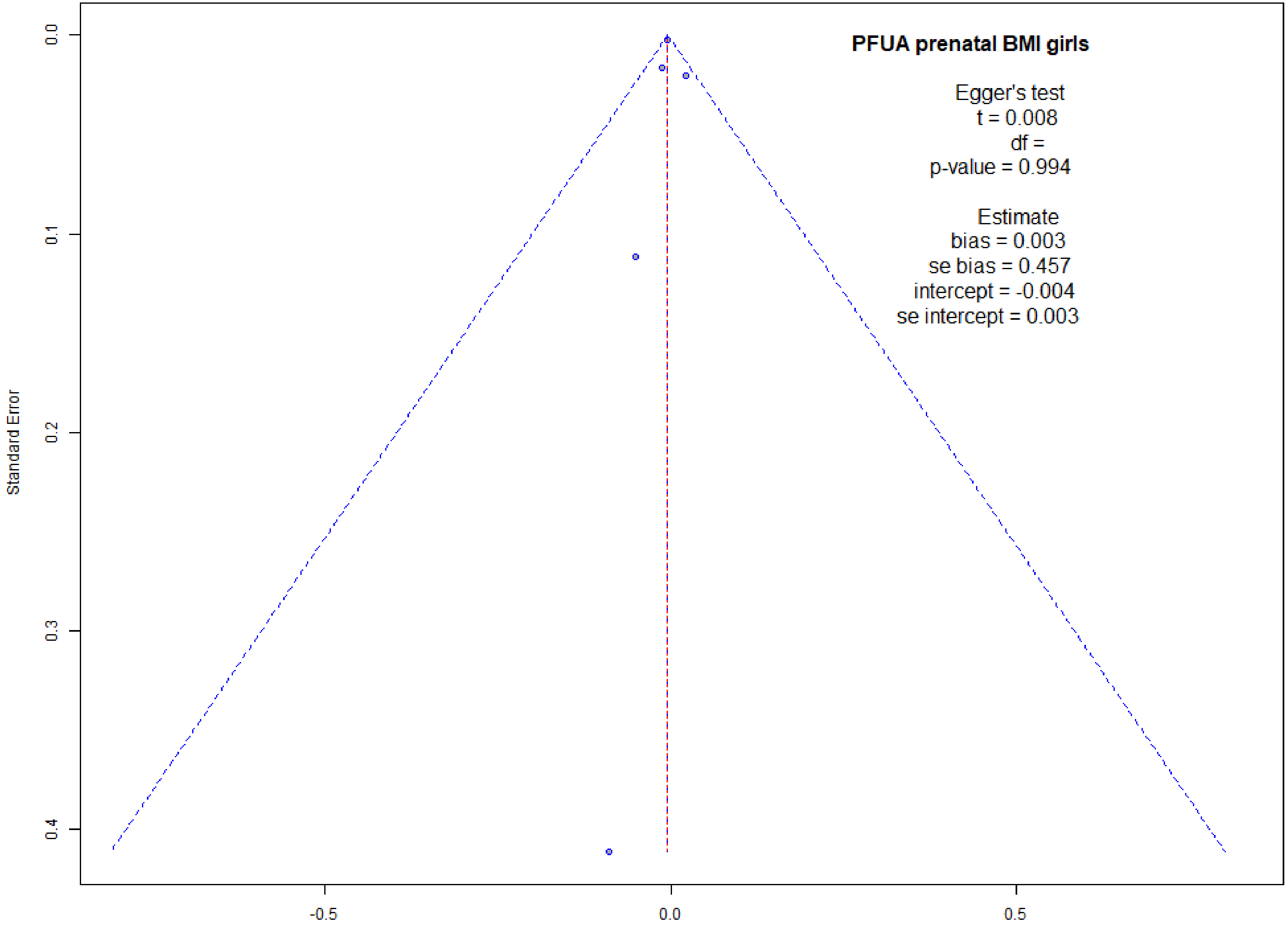
PFUA prenatal BMI girls
Egger's test
t = 0.008
df =
p-value = 0.994
Estimate
bias = 0.003
se bias = 0.457
intercept = -0.004
se intercept = 0.003
Standard Error
0.0
0.1
0.2
0.3
0.4
-0.5
0.0
0.5

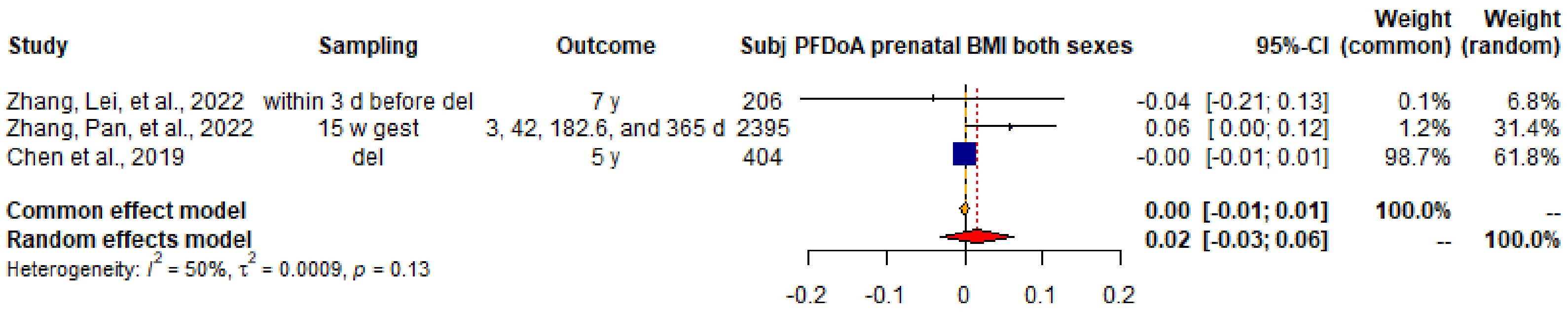
Study
Sampling
Outcome
Subj
PFDoA prenatal BMI both sexes
95%-CI
Weight (common)
Weight (random)
Zhang, Lei, et al., 2022
within 3 d before del
7 y
206
-0.04 [-0.21; 0.13]
0.1%
6.8%
Zhang, Pan, et al., 2022
15 w gest
3, 42, 182.6, and 365 d
2395
0.06 [0.00; 0.12]
1.2%
31.4%
Chen et al., 2019
del
5 y
404
-0.00 [-0.01; 0.01]
98.7%
61.8%
Common effect model
0.00 [-0.01; 0.01]
100.0%
--
Random effects model
0.02 [-0.03; 0.06]
--
100.0%
Heterogeneity: I^2 = 50%, τ^2 = 0.0009, p = 0.13
-0.2
-0.1
0
0.1
0.2

Study	Sampling	Outcome	Subjects	Estimate [95% CI]
Zhang, Lei, et al., 2022	within 3 d before del	7 y	206	-0.041 [-0.208; 0.127]
Zhang, Pan, et al., 2022	15 w gest	3, 42, 182.6, and 365 d	2395	0.058 [0.000; 0.116]
Chen et al., 2019	del	5 y	404	0.000 [-0.006; 0.006]
**Summary**				**0.043 [-0.022; 0.109]**

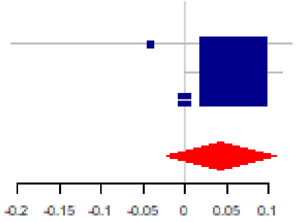

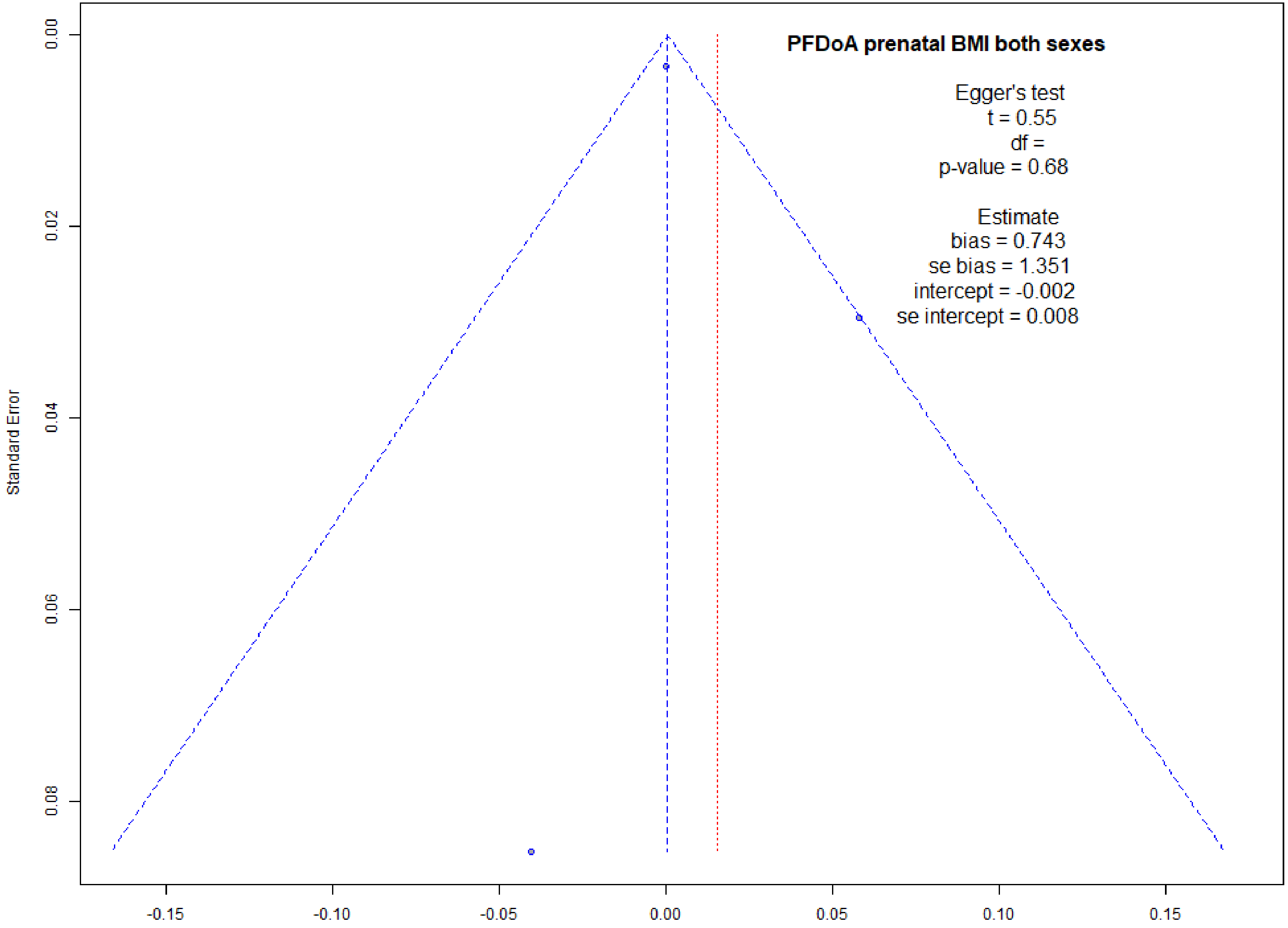
PFDoA prenatal BMI both sexes
Egger's test
t = 0.55
df =
p-value = 0.68
Estimate
bias = 0.743
se bias = 1.351
intercept = -0.002
se intercept = 0.008
Standard Error
0.00
0.02
0.04
0.06
0.08
-0.15
-0.10
-0.05
0.00
0.05
0.10
0.15

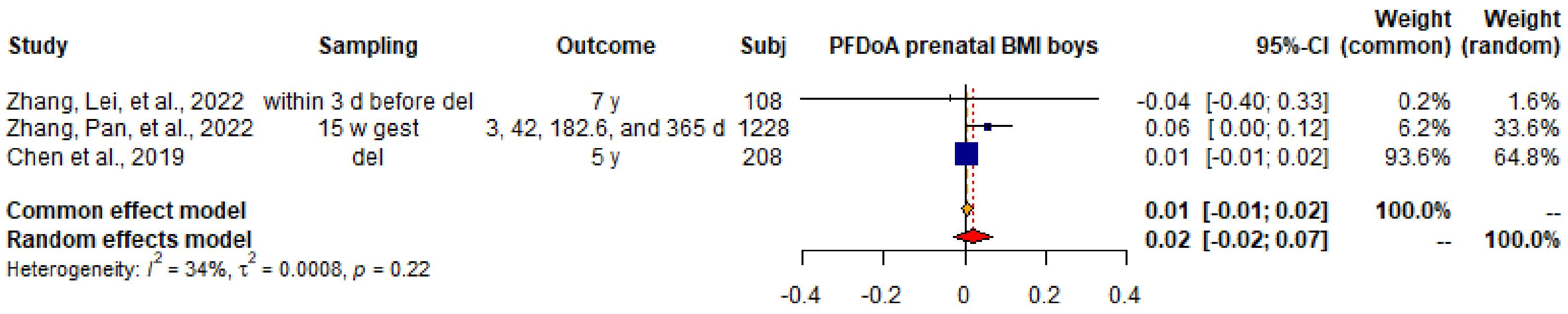
Study
Sampling
Outcome
Subj
PFDoA prenatal BMI boys
95%-CI
Weight (common)
Weight (random)
Zhang, Lei, et al., 2022 within 3 d before del 7 y 108 -0.04 [-0.40; 0.33] 0.2% 1.6%
Zhang, Pan, et al., 2022 15 w gest 3, 42, 182.6, and 365 d 1228 0.06 [0.00; 0.12] 6.2% 33.6%
Chen et al., 2019 del 5 y 208 0.01 [-0.01; 0.02] 93.6% 64.8%
Common effect model 0.01 [-0.01; 0.02] 100.0% --
Random effects model 0.02 [-0.02; 0.07] -- 100.0%
Heterogeneity: I^2 = 34%, τ^2 = 0.0008, p = 0.22
-0.4
-0.2
0
0.2
0.4

Study	Sampling	Outcome	Subjects	Estimate [95% CI]
Zhang, Lei, et al., 2022	within 3 d before del	7 y	108	-0.037 [-0.402; 0.328]
Zhang, Pan, et al., 2022	15 w gest	3, 42, 182.6, and 365 d	1228	0.058 [0.000; 0.116]
Chen et al., 2019	del	5 y	208	0.005 [-0.010; 0.020]
**Summary**				**0.044 [-0.019; 0.107]**

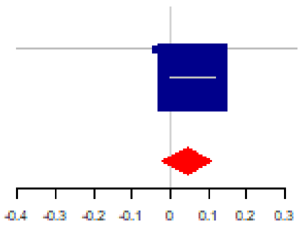

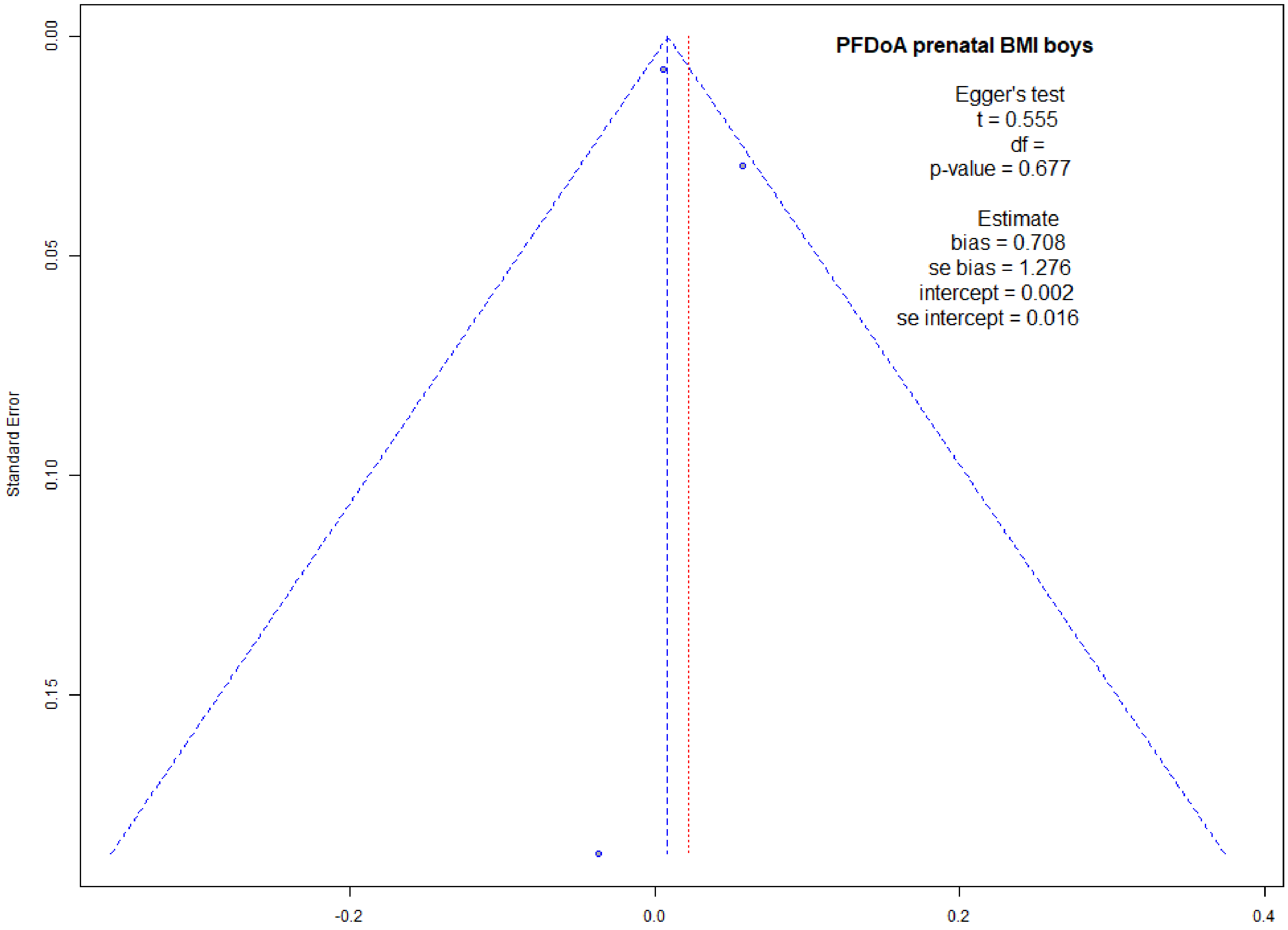
PFDoA prenatal BMI boys
Egger's test
t = 0.555
df =
p-value = 0.677
Estimate
bias = 0.708
se bias = 1.276
intercept = 0.002
se intercept = 0.016
Standard Error
0.00
0.05
0.10
0.15
-0.2
0.0
0.2
0.4

Study	Sampling	Outcome	Subj	PFDoA prenatal BMI girls	95%-CI	Weight (common)	Weight (random)
Zhang, Lei, et al., 2022	within 3 d before del	7 y	98		-0.03 [-0.13; 0.08]	1.1%	7.5%
Zhang, Pan, et al., 2022	15 w gest	3, 42, 182.6, and 365 d	1167		0.04 [-0.01; 0.09]	5.0%	25.7%
Chen et al., 2019	del	5 y	196		-0.00 [-0.01; 0.01]	93.9%	66.8%
**Common effect model**					**-0.00 [-0.01; 0.01]**	**100.0%**	--
**Random effects model**					**0.01 [-0.02; 0.04]**	--	**100.0%**

Heterogeneity: $I^2 = 34\%$, $\tau^2 = 0.0003$, $p = 0.22$

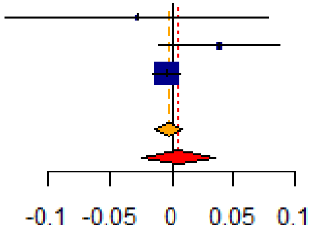

Study	Sampling	Outcome	Subjects	Estimate [95% CI]
Zhang, Lei, et al., 2022	within 3 d before del	7 y	98	-0.028 [-0.133; 0.077]
Zhang, Pan, et al., 2022	15 w gest	3, 42, 182.6, and 365 d	1167	0.039 [-0.010; 0.087]
Chen et al., 2019	del	5 y	196	-0.004 [-0.015; 0.008]
**Summary**				**0.028 [-0.019; 0.076]**

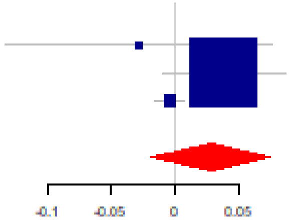

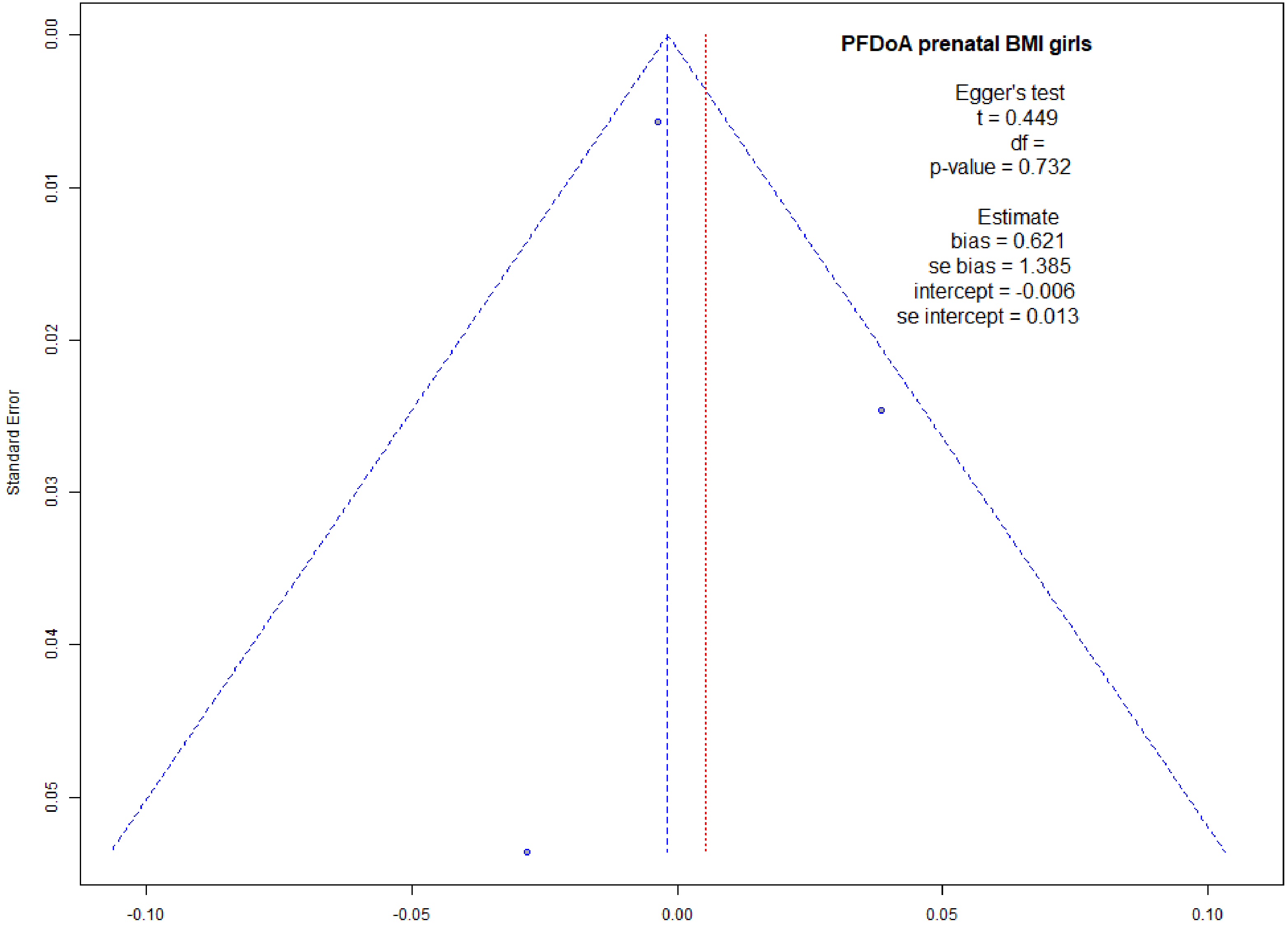
PFDoA prenatal BMI girls
Egger's test
t = 0.449
df =
p-value = 0.732
Estimate
bias = 0.621
se bias = 1.385
intercept = -0.006
se intercept = 0.013
Standard Error
0.00
0.01
0.02
0.03
0.04
0.05
-0.10
-0.05
0.00
0.05
0.10

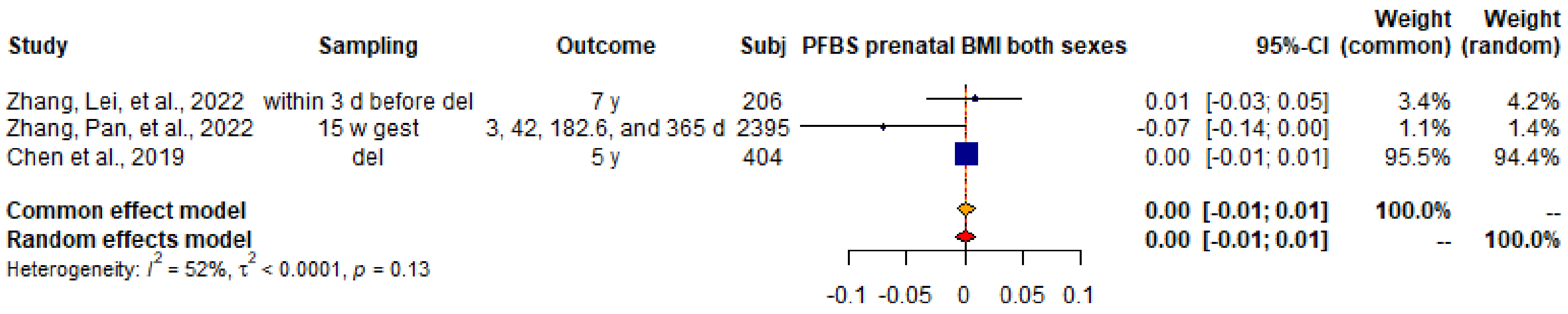
Study
Sampling
Outcome
Subj
PFBS prenatal BMI both sexes
95%-CI
Weight (common)
Weight (random)
Zhang, Lei, et al., 2022 within 3 d before del 7 y 206 0.01 [-0.03; 0.05] 3.4% 4.2%
Zhang, Pan, et al., 2022 15 w gest 3, 42, 182.6, and 365 d 2395 -0.07 [-0.14; 0.00] 1.1% 1.4%
Chen et al., 2019 del 5 y 404 0.00 [-0.01; 0.01] 95.5% 94.4%
Common effect model 0.00 [-0.01; 0.01] 100.0% --
Random effects model 0.00 [-0.01; 0.01] -- 100.0%
Heterogeneity: I2 = 52%, τ2 < 0.0001, p = 0.13
-0.1 -0.05 0 0.05 0.1

Study	Sampling	Outcome	Subjects	Estimate [95% CI]
Zhang, Lei, et al., 2022	within 3 d before del	7 y	206	0.009 [-0.032; 0.050]
Zhang, Pan, et al., 2022	15 w gest	3, 42, 182.6, and 365 d	2395	-0.071 [-0.141; 0.000]
Chen et al., 2019	del	5 y	404	0.002 [-0.006; 0.010]
**Summary**				**-0.056 [-0.120; 0.009]**

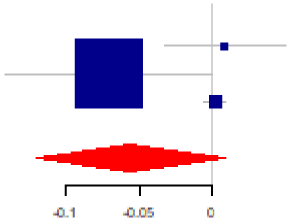

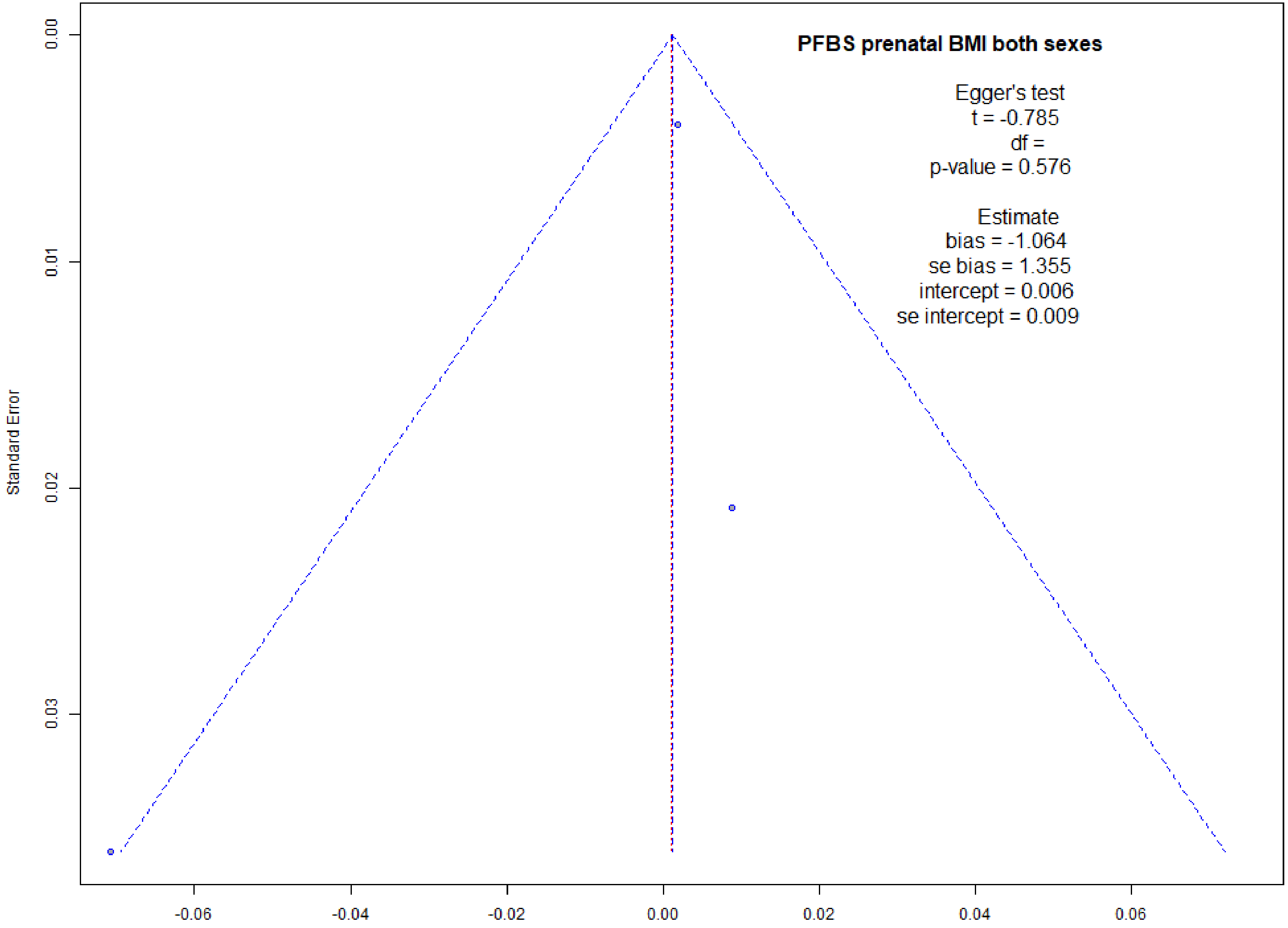
PFBS prenatal BMI both sexes
Egger's test
t = -0.785
df =
p-value = 0.576
Estimate
bias = -1.064
se bias = 1.355
intercept = 0.006
se intercept = 0.009
Standard Error
0.00
0.01
0.02
0.03
-0.06
-0.04
-0.02
0.00
0.02
0.04
0.06

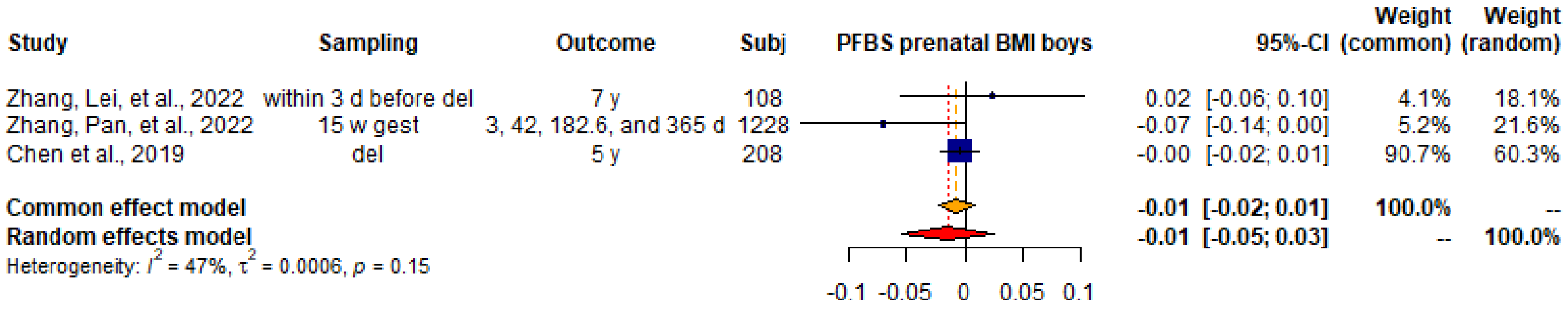
Study
Sampling
Outcome
Subj
PFBS prenatal BMI boys
95%-CI
Weight (common)
Weight (random)
Zhang, Lei, et al., 2022
within 3 d before del
7 y
108
0.02 [-0.06; 0.10]
4.1%
18.1%
Zhang, Pan, et al., 2022
15 w gest
3, 42, 182.6, and 365 d
1228
-0.07 [-0.14; 0.00]
5.2%
21.6%
Chen et al., 2019
del
5 y
208
-0.00 [-0.02; 0.01]
90.7%
60.3%
Common effect model
-0.01 [-0.02; 0.01]
100.0%
--
Random effects model
-0.01 [-0.05; 0.03]
--
100.0%
Heterogeneity: I^2 = 47%, τ^2 = 0.0006, p = 0.15
-0.1
-0.05
0
0.05
0.1

Study	Sampling	Outcome	Subjects	Estimate [95% CI]
Zhang, Lei, et al., 2022	within 3 d before del	7 y	108	0.024 [-0.056; 0.104]
Zhang, Pan, et al., 2022	15 w gest	3, 42, 182.6, and 365 d	1228	-0.071 [-0.141; 0.000]
Chen et al., 2019	del	5 y	208	-0.004 [-0.021; 0.013]
**Summary**				**-0.055 [-0.126; 0.015]**

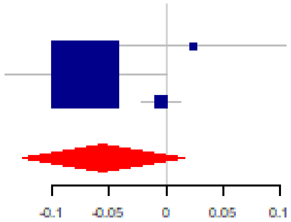

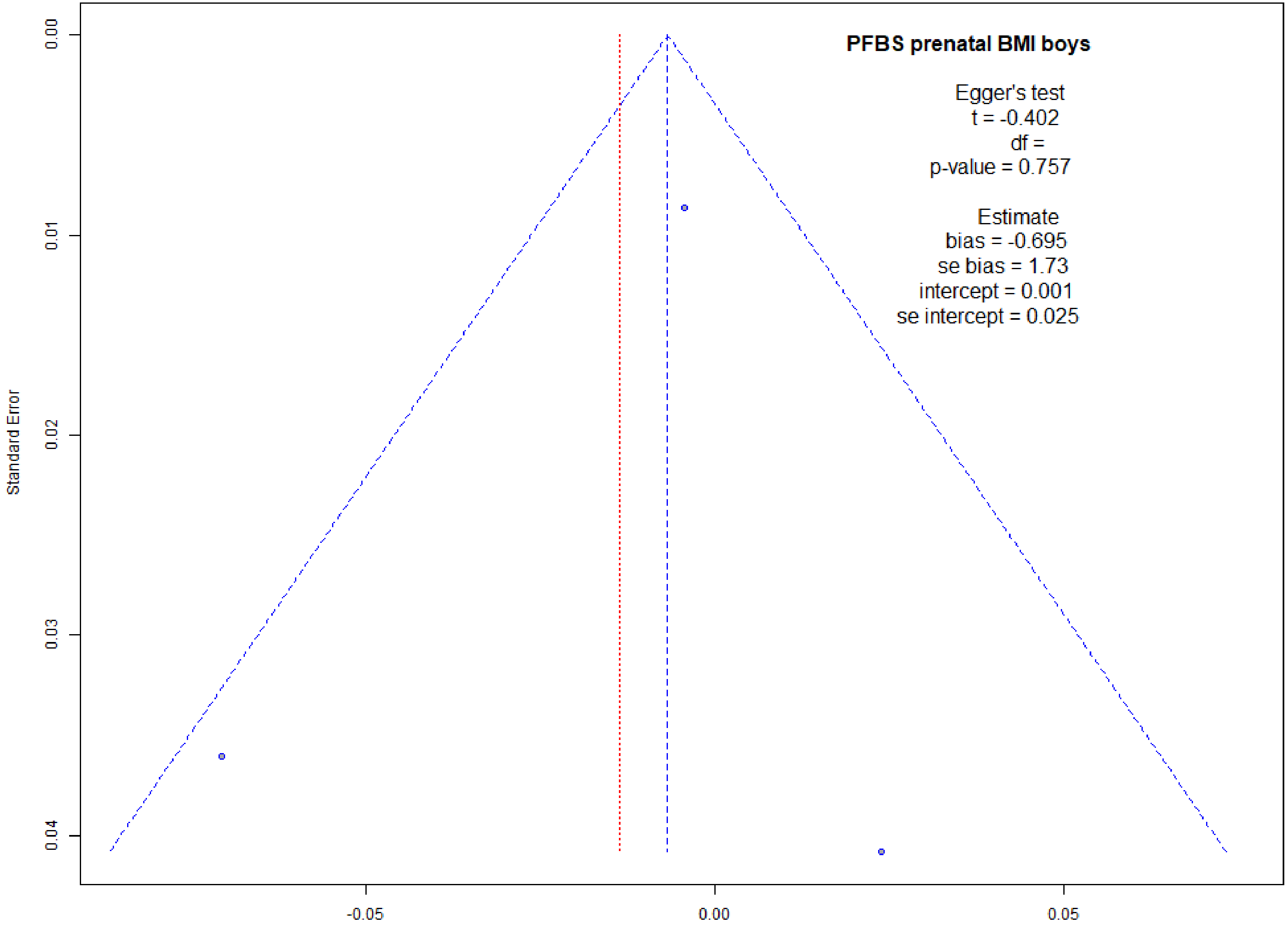
PFBS prenatal BMI boys
Egger's test
t = -0.402
df =
p-value = 0.757
Estimate
bias = -0.695
se bias = 1.73
intercept = 0.001
se intercept = 0.025
Standard Error
0.00
0.01
0.02
0.03
0.04
-0.05
0.00
0.05

Gianfranco Frigerio, Chiara Matilde Ferrari, and Silvia Fustinoni

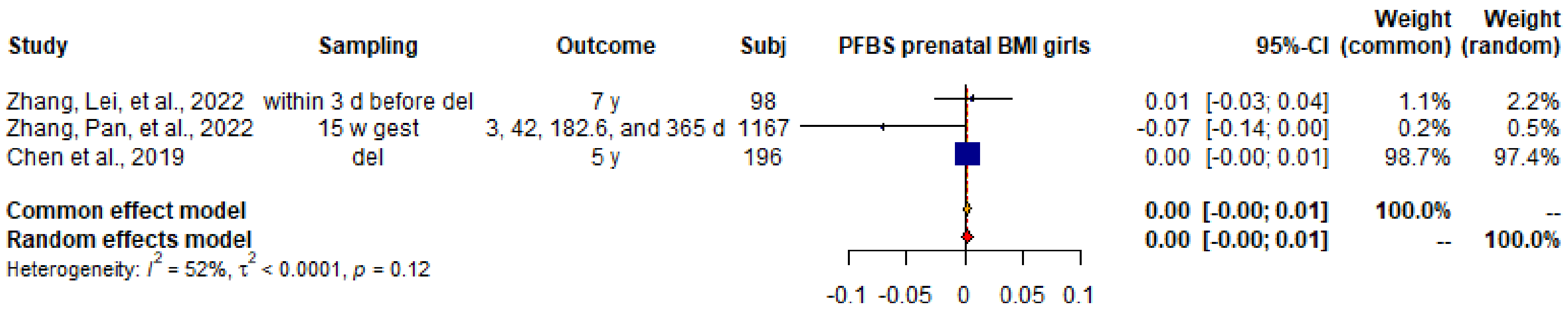

Study	Sampling	Outcome	Subjects	Estimate [95% CI]
Zhang, Lei, et al., 2022	within 3 d before del	7 y	98	0.007 [-0.026; 0.040]
Zhang, Pan, et al., 2022	15 w gest	3, 42, 182.6, and 365 d	1167	-0.071 [-0.141; 0.000]
Chen et al., 2019	del	5 y	196	0.003 [-0.001; 0.006]
**Summary**				**-0.056 [-0.118; 0.006]**

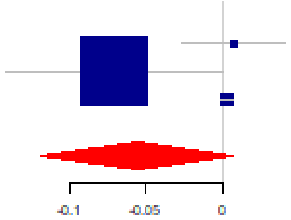

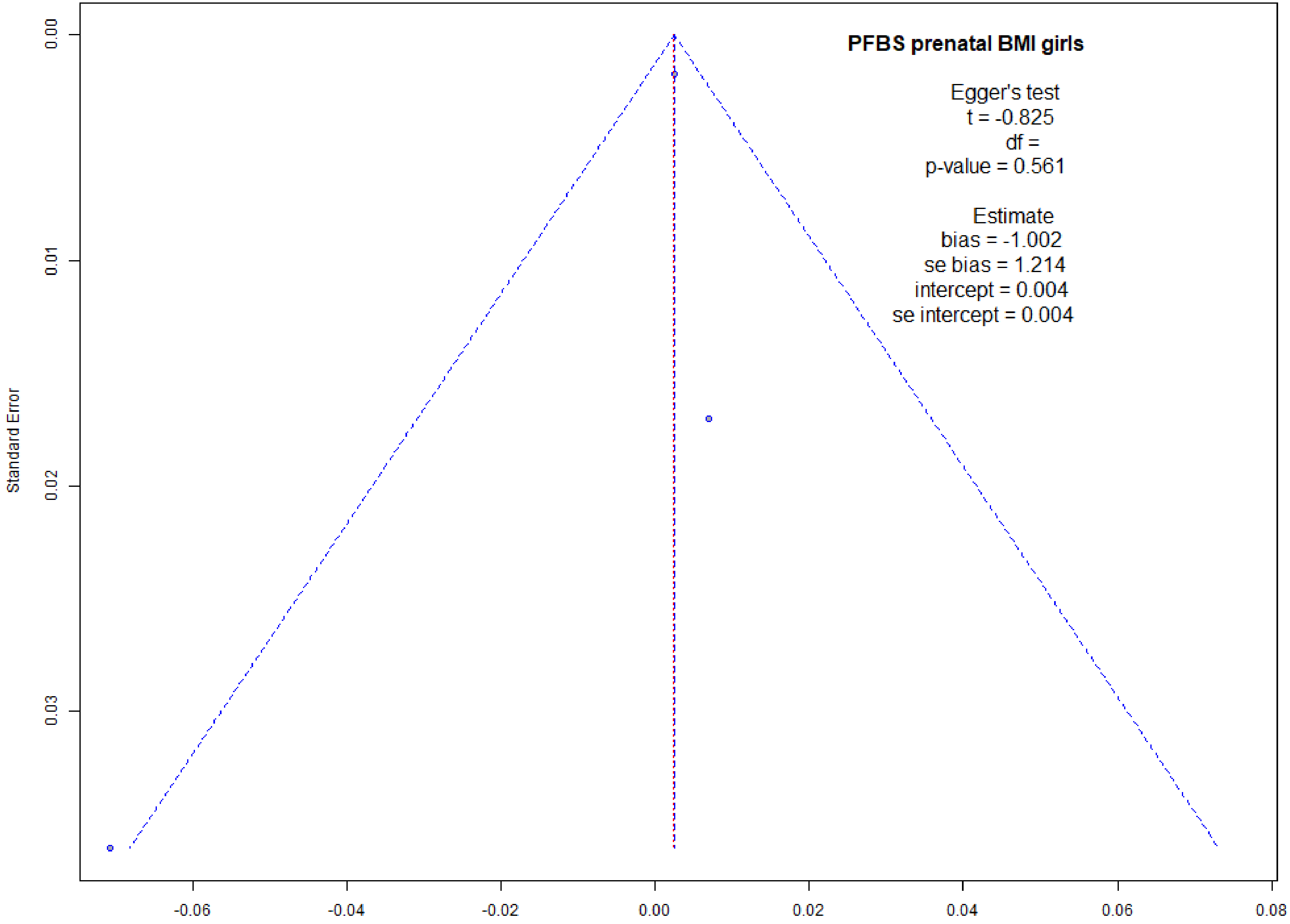
PFBS prenatal BMI girls
Egger's test
t = -0.825
df =
p-value = 0.561
Estimate
bias = -1.002
se bias = 1.214
intercept = 0.004
se intercept = 0.004
Standard Error
0.00
0.01
0.02
0.03
-0.06
-0.04
-0.02
0.00
0.02
0.04
0.06
0.08

Study	Sampling	Outcome	Subj	PFOA prenatal risk both sexes	95%-CI	Weight (common)	Weight (random)
Bloom et al., 2022	8-13 w gest	4-8 y	667	0.00	[-0.01; 0.02]	38.7%	38.0%
Martinsson et al., 2020	14 w of gest	4 y	1048	0.02	[-0.04; 0.07]	2.8%	2.8%
Lauritzen et al., 2018	17-20 w gest	5 y	412	0.08	[0.01; 0.15]	1.5%	1.6%
Mora et al., 2017	9.6 w gest	7.7 y	871	0.00	[-0.02; 0.02]	18.3%	18.4%
Braun et al., 2016	16 and 26 w gest and del	8 y	204	0.02	[-0.01; 0.04]	14.2%	14.3%
Karlsen et al., 2017	2 w after del	5 y	371	0.03	[-0.00; 0.06]	7.3%	7.4%
Manzano-Salgado et al., 2017	1st tr gest	7 y	1086	0.00	[-0.02; 0.03]	12.7%	12.8%
Hoyer et al., 2015 - Greenland	24 ± 10 w gest	5-9 y	531	0.02	[-0.03; 0.07]	3.1%	3.1%
Hoyer et al., 2015 - Ukraine	24 ± 10 w gest	5-9 y	491	0.00	[-0.07; 0.08]	1.4%	1.5%
**Common effect model**				**0.01**	**[-0.00; 0.02]**	**100.0%**	--
**Random effects model**				**0.01**	**[-0.00; 0.02]**	--	**100.0%**

Heterogeneity: $I^2 = 2\%$, $\tau^2 < 0.0001$, $p = 0.41$

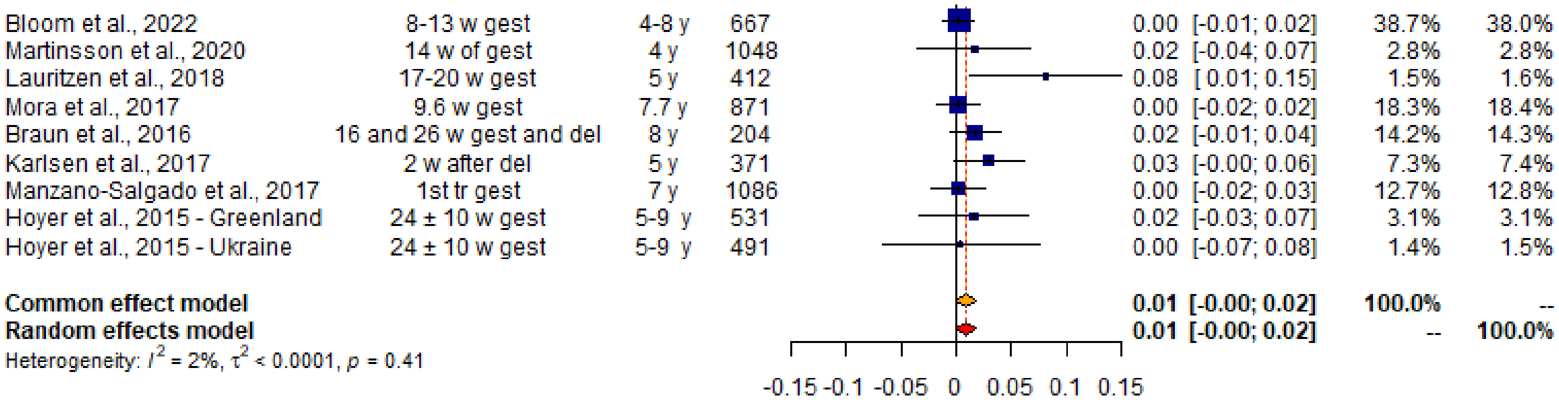

Study	Sampling	Outcome	Subjects	Estimate [95% CI]
Bloom et al., 2022	8-13 w gest	4-8 y	667	0.002 [-0.012; 0.016]
Martinsson et al., 2020	14 w of gest	4 y	1048	0.016 [-0.035; 0.068]
Lauritzen et al., 2018	17-20 w gest	5 y	412	0.081 [0.012; 0.151]
Mora et al., 2017	9.6 w gest	7.7 y	871	0.002 [-0.018; 0.022]
Braun et al., 2016	16 and 26 w gest and del	8 y	204	0.017 [-0.006; 0.040]
Karlsen et al., 2017	2 w after del	5 y	371	0.030 [-0.002; 0.062]
Manzano-Salgado et al., 2017	1st tr gest	7 y	1086	0.002 [-0.022; 0.026]
Hoyer et al., 2015 - Greenland	24 ± 10 w gest	5-9 y	531	0.016 [-0.034; 0.065]
Hoyer et al., 2015 - Ukraine	24 ± 10 w gest	5-9 y	491	0.004 [-0.068; 0.076]
**Summary**				**0.014 [-0.001; 0.029]**

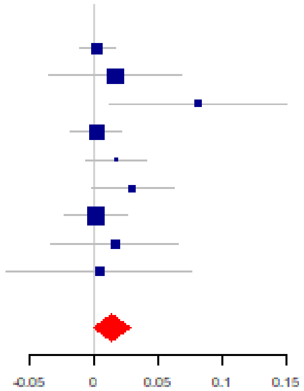

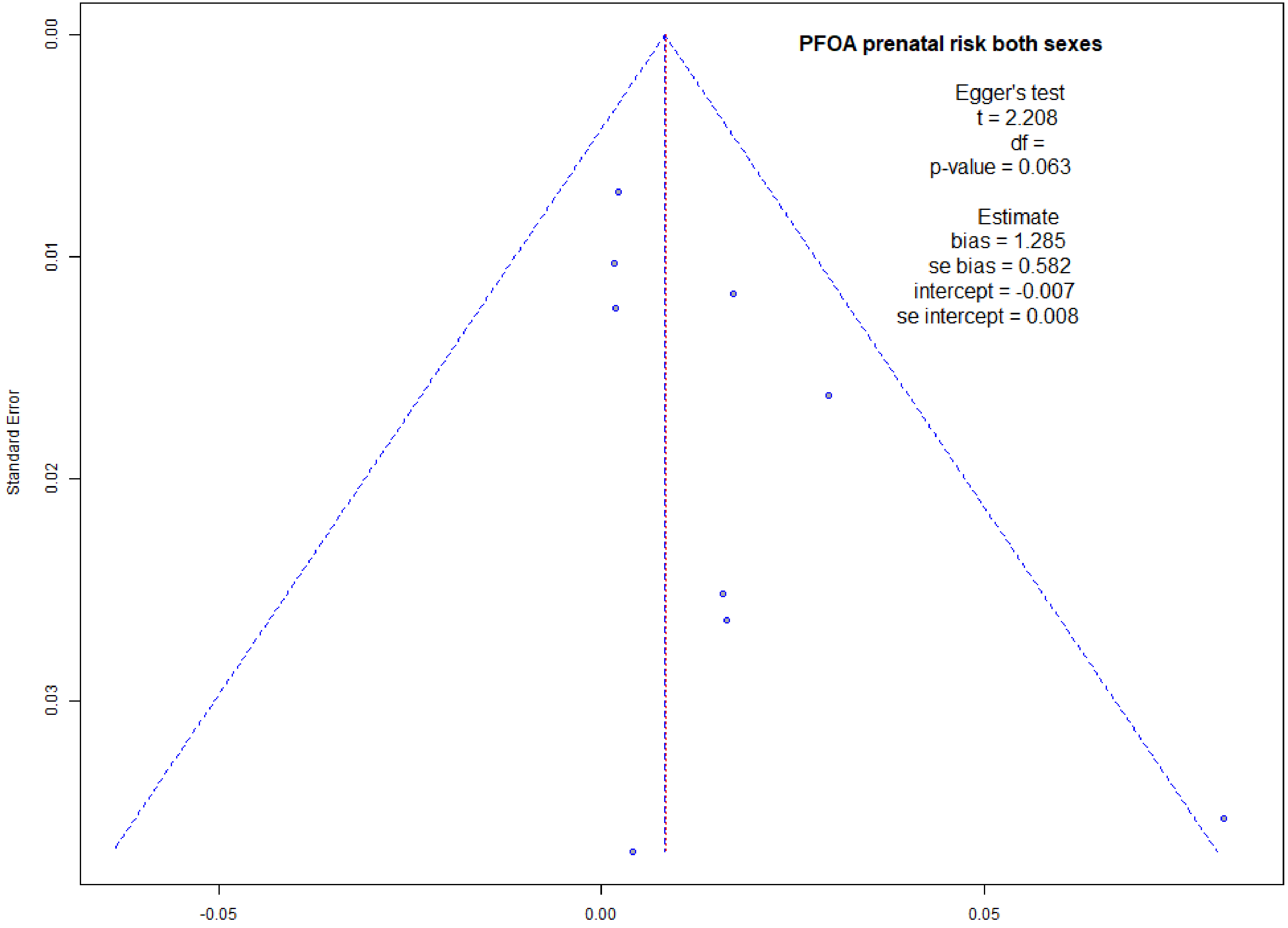
PFOA prenatal risk both sexes
Egger's test
t = 2.208
df =
p-value = 0.063
Estimate
bias = 1.285
se bias = 0.582
intercept = -0.007
se intercept = 0.008
Standard Error
0.00
0.01
0.02
0.03
-0.05
0.00
0.05

Study	Sampling	Outcome	Subj	95%-CI	Weight (common)	Weight (random)
Bloom et al., 2022	8-13 w gest	4-8 y	667	-0.00 [-0.00; 0.00]	36.3%	26.1%
Martinsson et al., 2020	14 w of gest	4 y	1048	0.00 [0.00; 0.01]	17.1%	18.2%
Lauritzen et al., 2018	17-20 w gest	5 y	412	0.01 [-0.01; 0.03]	0.6%	1.0%
Mora et al., 2017	9.6 w gest	7.7 y	871	0.00 [-0.00; 0.01]	11.2%	14.0%
Braun et al., 2016	16 and 26 w gest and del	8 y	204	-0.00 [-0.00; 0.00]	20.3%	20.0%
Karlsen et al., 2017	2 w after del	5 y	371	0.00 [-0.01; 0.01]	2.7%	4.5%
Manzano-Salgado et al., 2017	1st tr gest	7 y	1086	-0.00 [-0.01; 0.01]	1.5%	2.7%
Hoyer et al., 2015 - Greenland	24 ± 10 w gest	5-9 y	531	-0.00 [-0.01; 0.00]	9.8%	12.8%
Hoyer et al., 2015 - Ukraine	24 ± 10 w gest	5-9 y	491	0.01 [-0.02; 0.03]	0.4%	0.8%
**Common effect model**				**0.00 [-0.00; 0.00]**	**100.0%**	**--**
**Random effects model**				**0.00 [-0.00; 0.00]**	**--**	**100.0%**

Heterogeneity: $I^2 = 19\%$, $\tau^2 < 0.0001$, $p = 0.27$

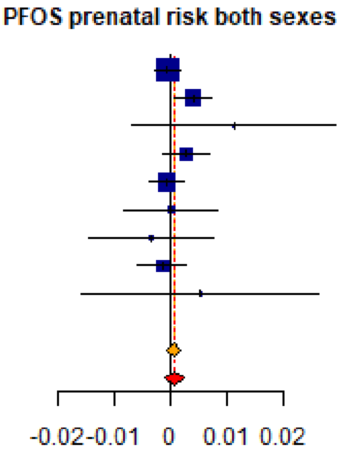

Study	Sampling	Outcome	Subjects	Estimate [95% CI]
Bloom et al., 2022	8-13 w gest	4-8 y	667	-0.001 [-0.003; 0.002]
Martinsson et al., 2020	14 w of gest	4 y	1048	0.004 [0.001; 0.007]
Lauritzen et al., 2018	17-20 w gest	5 y	412	0.011 [-0.007; 0.029]
Mora et al., 2017	9.6 w gest	7.7 y	871	0.003 [-0.001; 0.007]
Braun et al., 2016	16 and 26 w gest and del	8 y	204	-0.001 [-0.004; 0.002]
Karlsen et al., 2017	2 w after del	5 y	371	0.000 [-0.008; 0.008]
Manzano-Salgado et al., 2017	1st tr gest	7 y	1086	-0.003 [-0.014; 0.008]
Hoyer et al., 2015 - Greenland	24 ± 10 w gest	5-9 y	531	-0.002 [-0.006; 0.003]
Hoyer et al., 2015 - Ukraine	24 ± 10 w gest	5-9 y	491	0.005 [-0.016; 0.026]
**Summary**				**0.002 [-0.002; 0.005]**

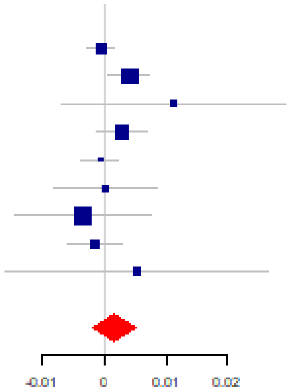

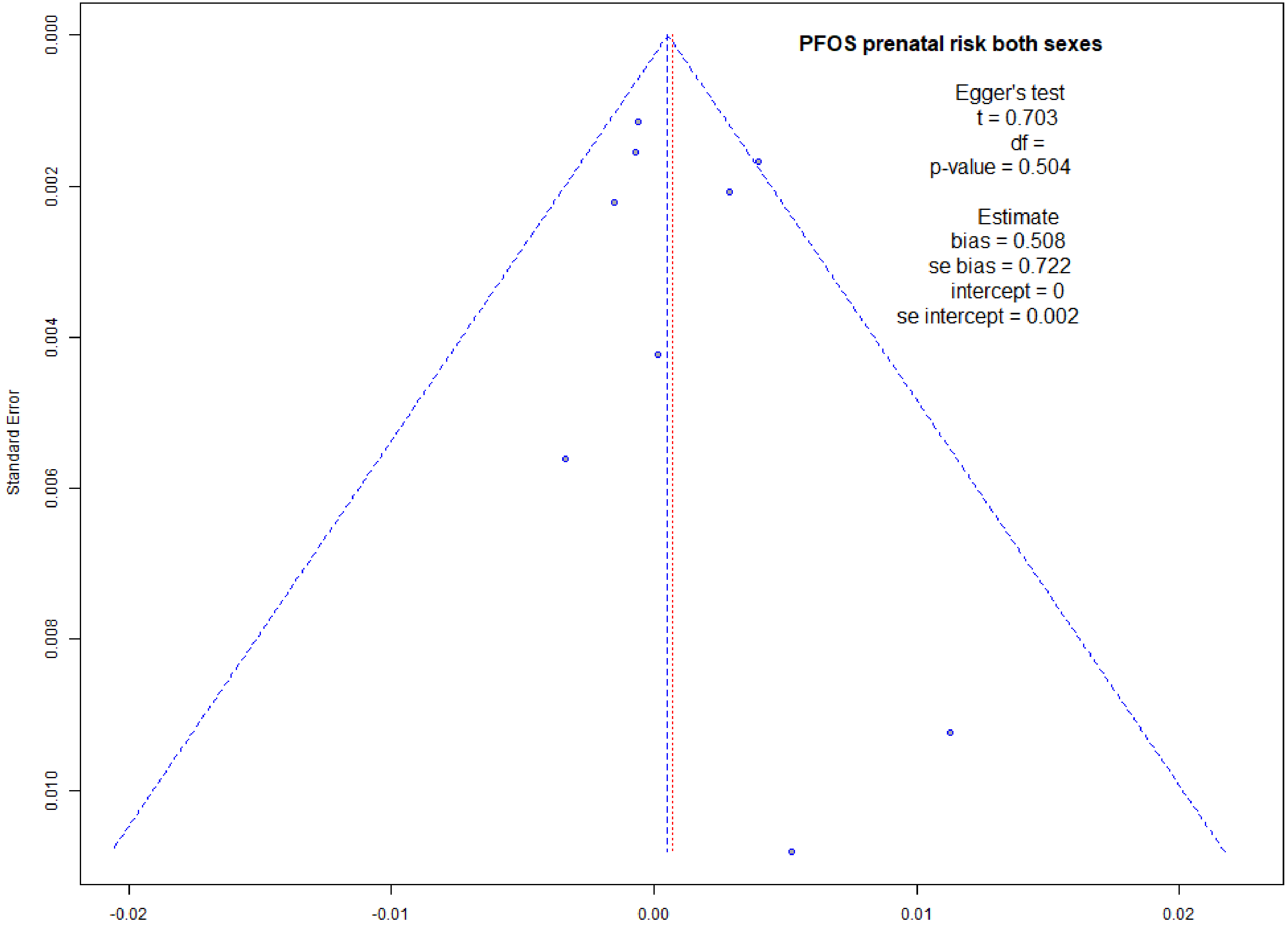
PFOS prenatal risk both sexes
Egger's test
t = 0.703
df =
p-value = 0.504
Estimate
bias = 0.508
se bias = 0.722
intercept = 0
se intercept = 0.002
Standard Error
0.000
0.002
0.004
0.006
0.008
0.010
-0.02
-0.01
0.00
0.01
0.02

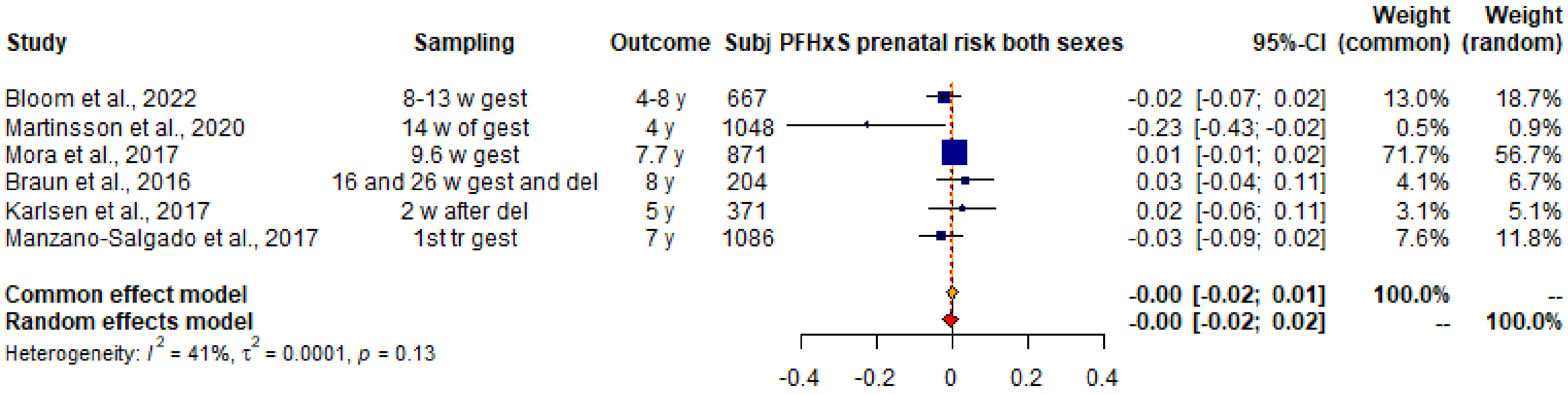
Study
Sampling
Outcome
Subj
PFHxS prenatal risk both sexes
95%-CI
Weight (common)
Weight (random)
Bloom et al., 2022 | 8-13 w gest | 4-8 y | 667 | -0.02 [-0.07; 0.02] | 13.0% | 18.7%
Martinsson et al., 2020 | 14 w of gest | 4 y | 1048 | -0.23 [-0.43; -0.02] | 0.5% | 0.9%
Mora et al., 2017 | 9.6 w gest | 7.7 y | 871 | 0.01 [-0.01; 0.02] | 71.7% | 56.7%
Braun et al., 2016 | 16 and 26 w gest and del | 8 y | 204 | 0.03 [-0.04; 0.11] | 4.1% | 6.7%
Karlsen et al., 2017 | 2 w after del | 5 y | 371 | 0.02 [-0.06; 0.11] | 3.1% | 5.1%
Manzano-Salgado et al., 2017 | 1st tr gest | 7 y | 1086 | -0.03 [-0.09; 0.02] | 7.6% | 11.8%
Common effect model | -0.00 [-0.02; 0.01] | 100.0% | --
Random effects model | -0.00 [-0.02; 0.02] | -- | 100.0%
Heterogeneity: I^2 = 41%, τ^2 = 0.0001, p = 0.13
-0.4
-0.2
0
0.2
0.4

Study	Sampling	Outcome	Subjects	Estimate [95% CI]
Bloom et al., 2022	8-13 w gest	4-8 y	667	-0.024 [-0.066; 0.018]
Martinsson et al., 2020	14 w of gest	4 y	1048	-0.227 [-0.434; -0.019]
Mora et al., 2017	9.6 w gest	7.7 y	871	0.006 [-0.012; 0.024]
Braun et al., 2016	16 and 26 w gest and del	8 y	204	0.033 [-0.041; 0.108]
Karlsen et al., 2017	2 w after del	5 y	371	0.024 [-0.062; 0.111]
Manzano-Salgado et al., 2017	1st tr gest	7 y	1086	-0.031 [-0.086; 0.024]
**Summary**				**-0.063 [-0.120; -0.005]**

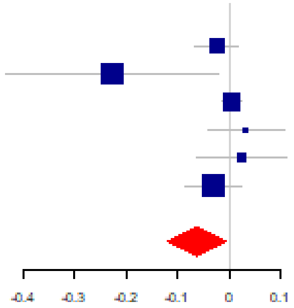

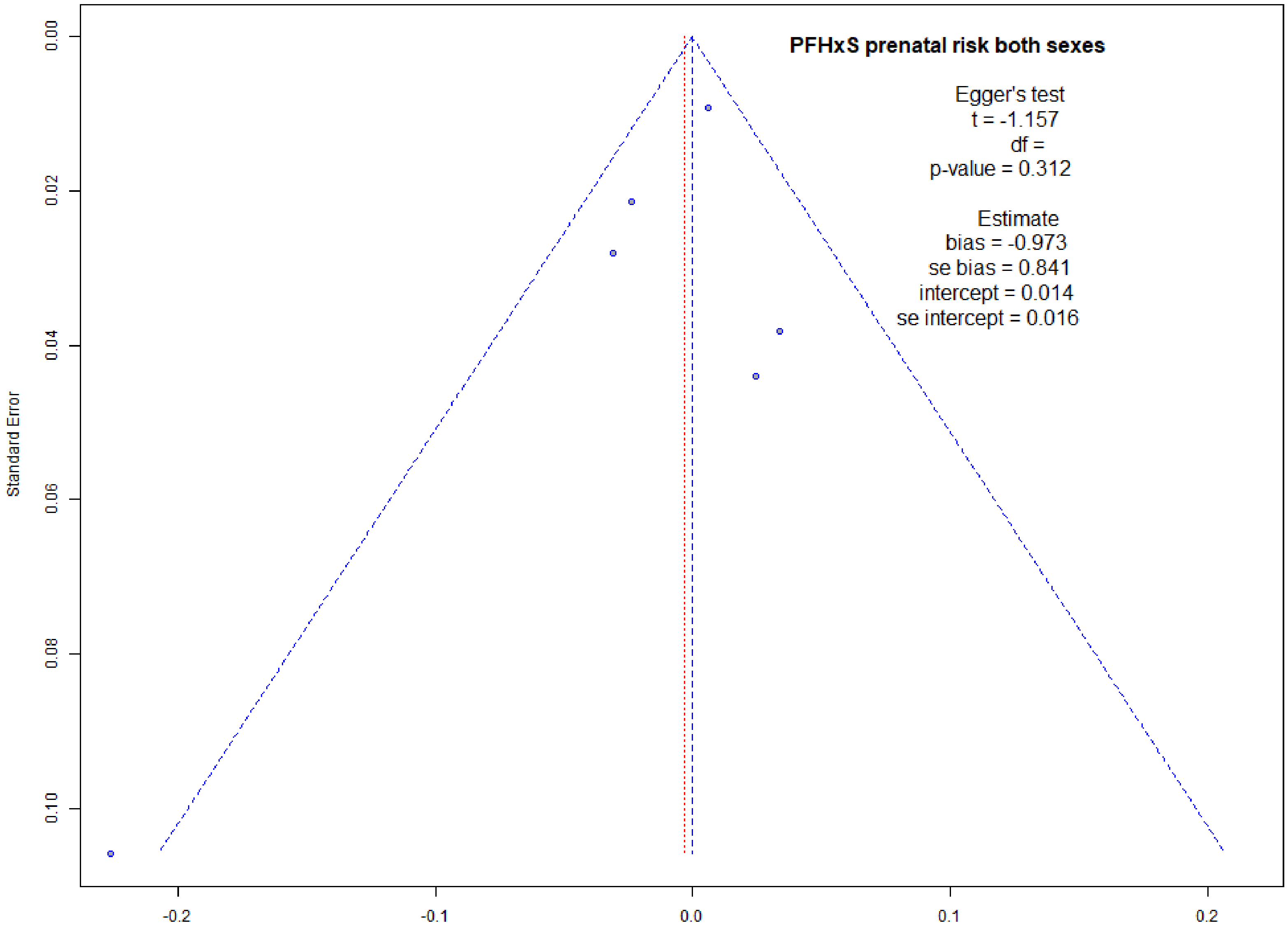
PFHxS prenatal risk both sexes
Egger's test
t = -1.157
df =
p-value = 0.312
Estimate
bias = -0.973
se bias = 0.841
intercept = 0.014
se intercept = 0.016
Standard Error
0.00
0.02
0.04
0.06
0.08
0.10
-0.2
-0.1
0.0
0.1
0.2

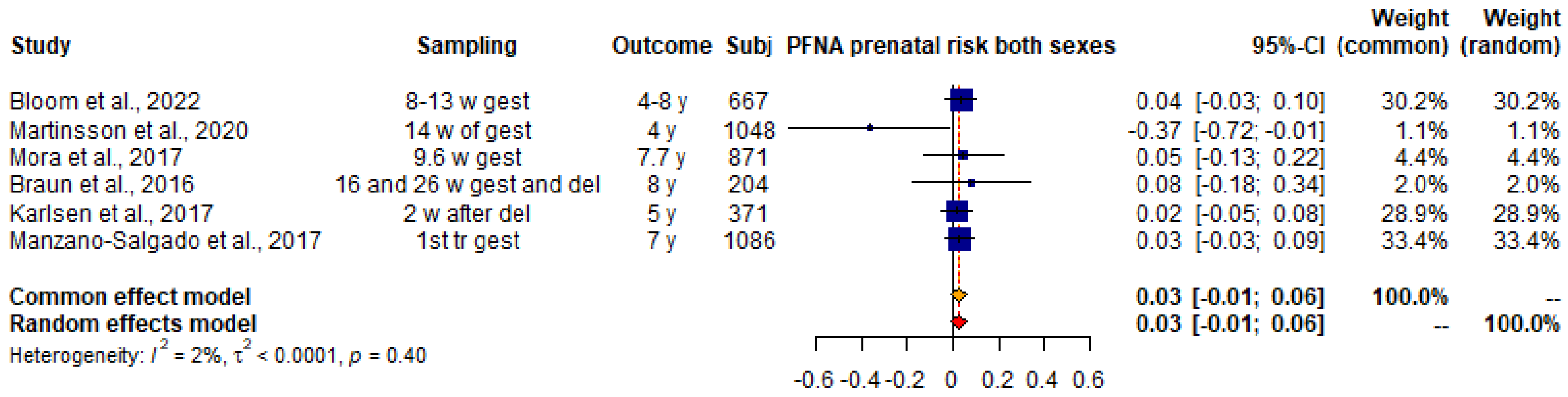
Study
Sampling
Outcome
Subj
PFNA prenatal risk both sexes
95%-CI
Weight (common)
Weight (random)
Bloom et al., 2022 8-13 w gest 4-8 y 667 0.04 [-0.03; 0.10] 30.2% 30.2%
Martinsson et al., 2020 14 w of gest 4 y 1048 -0.37 [-0.72; -0.01] 1.1% 1.1%
Mora et al., 2017 9.6 w gest 7.7 y 871 0.05 [-0.13; 0.22] 4.4% 4.4%
Braun et al., 2016 16 and 26 w gest and del 8 y 204 0.08 [-0.18; 0.34] 2.0% 2.0%
Karlsen et al., 2017 2 w after del 5 y 371 0.02 [-0.05; 0.08] 28.9% 28.9%
Manzano-Salgado et al., 2017 1st tr gest 7 y 1086 0.03 [-0.03; 0.09] 33.4% 33.4%
Common effect model 0.03 [-0.01; 0.06] 100.0% --
Random effects model 0.03 [-0.01; 0.06] -- 100.0%
Heterogeneity: I^2 = 2%, τ^2 < 0.0001, p = 0.40
-0.6 -0.4 -0.2 0 0.2 0.4 0.6

Study	Sampling	Outcome	Subjects	Estimate [95% CI]
Bloom et al., 2022	8-13 w gest	4-8 y	667	0.036 [-0.030; 0.103]
Martinsson et al., 2020	14 w of gest	4 y	1048	-0.368 [-0.724; -0.013]
Mora et al., 2017	9.6 w gest	7.7 y	871	0.045 [-0.128; 0.219]
Braun et al., 2016	16 and 26 w gest and del	8 y	204	0.080 [-0.181; 0.341]
Karlsen et al., 2017	2 w after del	5 y	371	0.017 [-0.051; 0.085]
Manzano-Salgado et al., 2017	1st tr gest	7 y	1086	0.028 [-0.035; 0.092]
**Summary**				**-0.063 [-0.161; 0.034]**

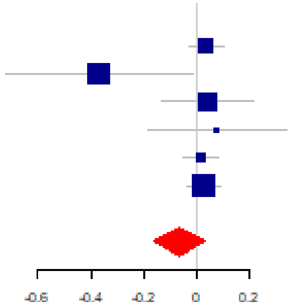

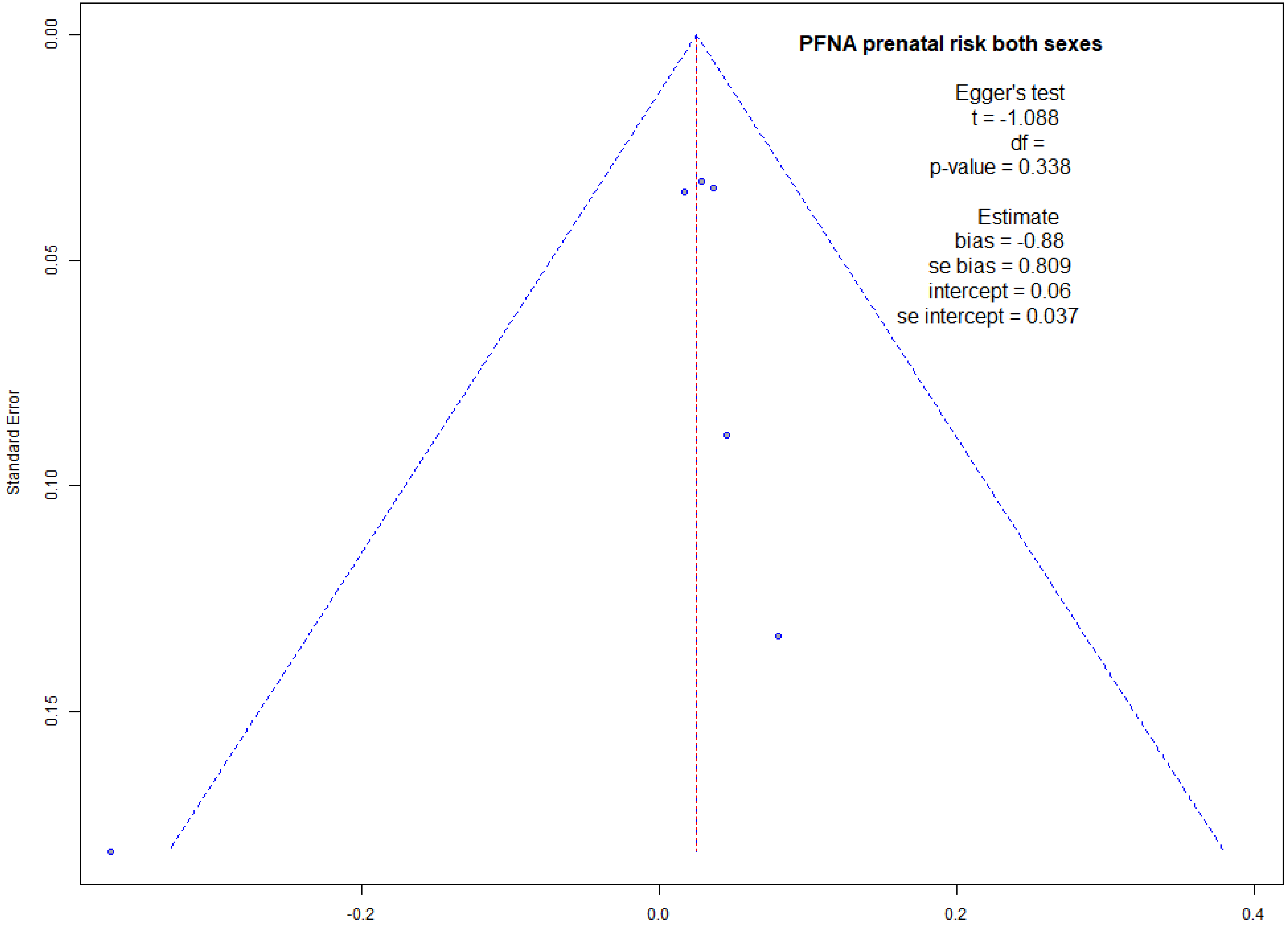
PFNA prenatal risk both sexes
Egger's test
t = -1.088
df =
p-value = 0.338
Estimate
bias = -0.88
se bias = 0.809
intercept = 0.06
se intercept = 0.037
Standard Error
0.00
0.05
0.10
0.15
-0.2
0.0
0.2
0.4

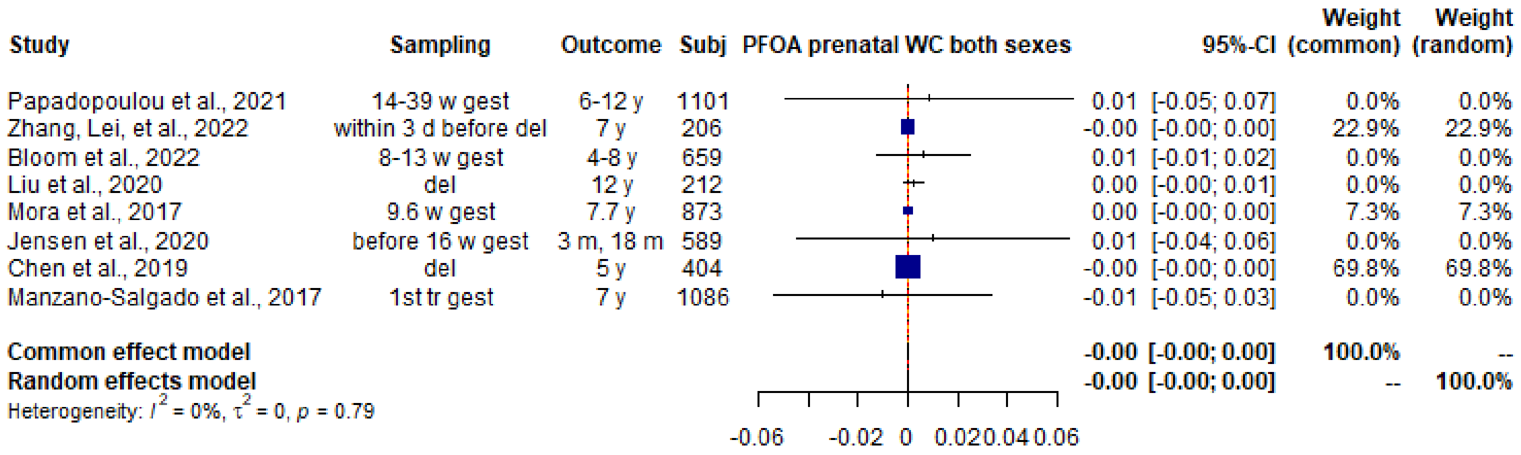
Study
Sampling
Outcome
Subj
PFOA prenatal WC both sexes
95%-CI
Weight (common)
Weight (random)
Papadopoulou et al., 2021 | 14-39 w gest | 6-12 y | 1101 | 0.01 [-0.05; 0.07] | 0.0% | 0.0%
Zhang, Lei, et al., 2022 | within 3 d before del | 7 y | 206 | -0.00 [-0.00; 0.00] | 22.9% | 22.9%
Bloom et al., 2022 | 8-13 w gest | 4-8 y | 659 | 0.01 [-0.01; 0.02] | 0.0% | 0.0%
Liu et al., 2020 | del | 12 y | 212 | 0.00 [-0.00; 0.01] | 0.0% | 0.0%
Mora et al., 2017 | 9.6 w gest | 7.7 y | 873 | 0.00 [-0.00; 0.00] | 7.3% | 7.3%
Jensen et al., 2020 | before 16 w gest | 3 m, 18 m | 589 | 0.01 [-0.04; 0.06] | 0.0% | 0.0%
Chen et al., 2019 | del | 5 y | 404 | -0.00 [-0.00; 0.00] | 69.8% | 69.8%
Manzano-Salgado et al., 2017 | 1st tr gest | 7 y | 1086 | -0.01 [-0.05; 0.03] | 0.0% | 0.0%
Common effect model | -0.00 [-0.00; 0.00] | 100.0% | --
Random effects model | -0.00 [-0.00; 0.00] | -- | 100.0%
Heterogeneity: I^2 = 0%, τ^2 = 0, p = 0.79
-0.06 -0.02 0 0.02 0.04 0.06

Study	Sampling	Outcome	Subjects	Estimate [95% CI]
Papadopoulou et al., 2021	14-39 w gest	6-12 y	1101	0.009 [-0.049; 0.067]
Zhang, Lei, et al., 2022	within 3 d before del	7 y	206	0.000 [0.000; 0.000]
Bloom et al., 2022	8-13 w gest	4-8 y	659	0.006 [-0.013; 0.025]
Liu et al., 2020	del	12 y	212	0.003 [-0.001; 0.006]
Mora et al., 2017	9.6 w gest	7.7 y	873	0.000 [0.000; 0.000]
Jensen et al., 2020	before 16 w gest	3 m, 18 m	589	0.010 [-0.045; 0.065]
Chen et al., 2019	del	5 y	404	0.000 [0.000; 0.000]
Manzano-Salgado et al., 2017	1st tr gest	7 y	1086	-0.010 [-0.054; 0.034]
**Summary**				**0.002 [-0.015; 0.019]**

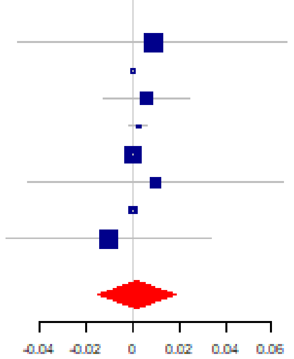

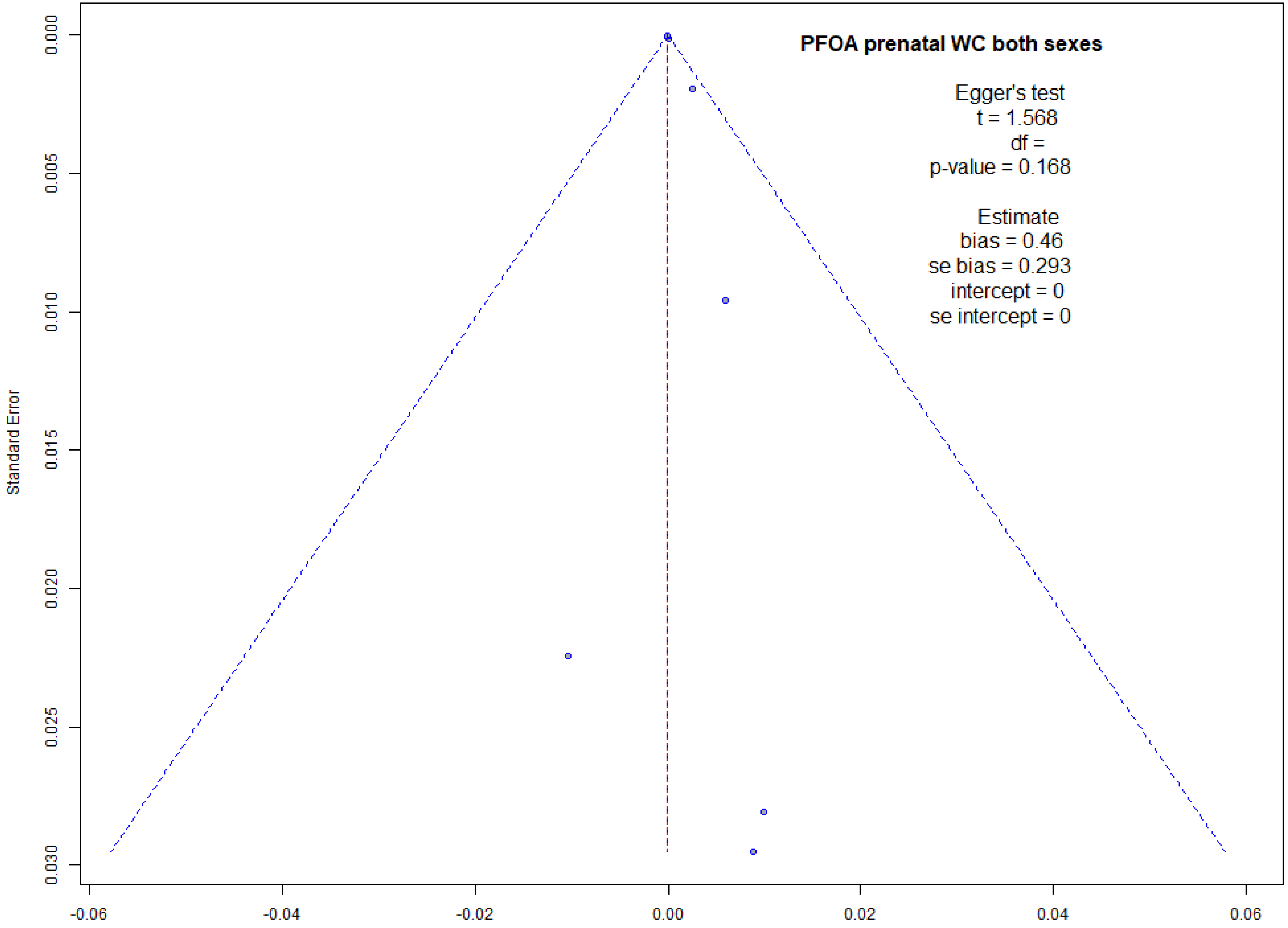
PFOA prenatal WC both sexes
Egger's test
t = 1.568
df =
p-value = 0.168
Estimate
bias = 0.46
se bias = 0.293
intercept = 0
se intercept = 0
Standard Error
0.000
0.005
0.010
0.015
0.020
0.025
0.030
-0.06
-0.04
-0.02
0.00
0.02
0.04
0.06

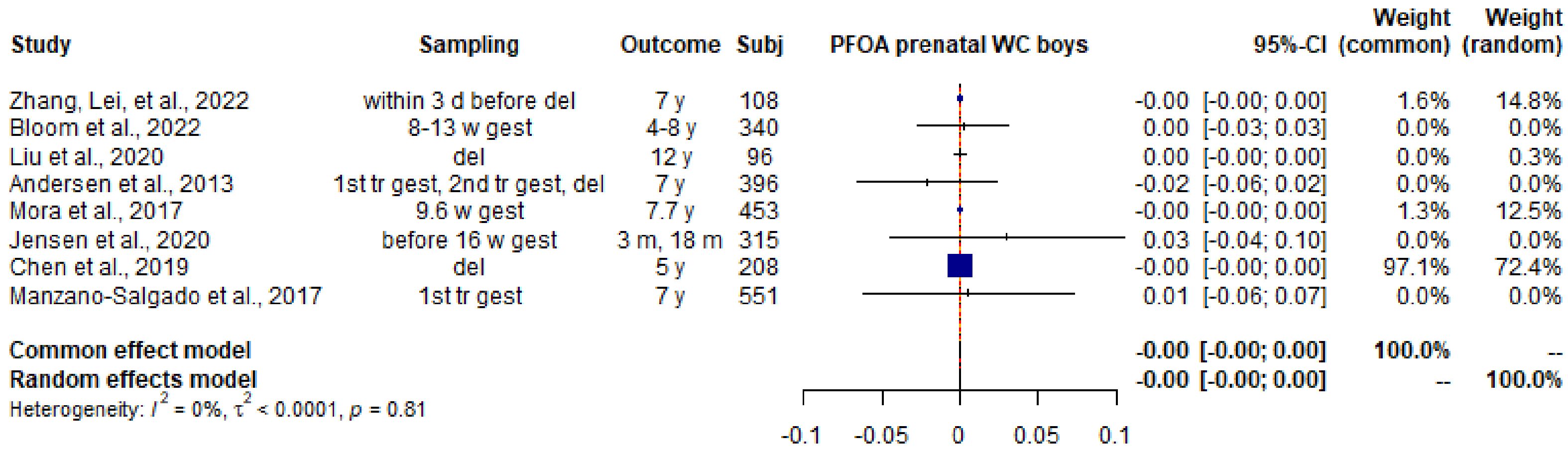
Study
Sampling
Outcome
Subj
PFOA prenatal WC boys
95%-CI
Weight (common)
Weight (random)
Zhang, Lei, et al., 2022 | within 3 d before del | 7 y | 108 | -0.00 [-0.00; 0.00] | 1.6% | 14.8%
Bloom et al., 2022 | 8-13 w gest | 4-8 y | 340 | 0.00 [-0.03; 0.03] | 0.0% | 0.0%
Liu et al., 2020 | del | 12 y | 96 | 0.00 [-0.00; 0.00] | 0.0% | 0.3%
Andersen et al., 2013 | 1st tr gest, 2nd tr gest, del | 7 y | 396 | -0.02 [-0.06; 0.02] | 0.0% | 0.0%
Mora et al., 2017 | 9.6 w gest | 7.7 y | 453 | -0.00 [-0.00; 0.00] | 1.3% | 12.5%
Jensen et al., 2020 | before 16 w gest | 3 m, 18 m | 315 | 0.03 [-0.04; 0.10] | 0.0% | 0.0%
Chen et al., 2019 | del | 5 y | 208 | -0.00 [-0.00; 0.00] | 97.1% | 72.4%
Manzano-Salgado et al., 2017 | 1st tr gest | 7 y | 551 | 0.01 [-0.06; 0.07] | 0.0% | 0.0%
Common effect model | -0.00 [-0.00; 0.00] | 100.0% | --
Random effects model | -0.00 [-0.00; 0.00] | -- | 100.0%
Heterogeneity: I^2 = 0%, τ^2 < 0.0001, p = 0.81
-0.1
-0.05
0
0.05
0.1

Study	Sampling	Outcome	Subjects	Estimate [95% CI]
Zhang, Lei, et al., 2022	within 3 d before del	7 y	108	0.000 [-0.001; 0.000]
Bloom et al., 2022	8-13 w gest	4-8 y	340	0.002 [-0.027; 0.031]
Liu et al., 2020	del	12 y	96	0.000 [-0.003; 0.003]
Andersen et al., 2013	1st tr gest, 2nd tr gest, del	7 y	396	-0.021 [-0.065; 0.023]
Mora et al., 2017	9.6 w gest	7.7 y	453	0.000 [-0.001; 0.000]
Jensen et al., 2020	before 16 w gest	3 m, 18 m	315	0.030 [-0.045; 0.105]
Chen et al., 2019	del	5 y	208	0.000 [0.000; 0.000]
Manzano-Salgado et al., 2017	1st tr gest	7 y	551	0.005 [-0.062; 0.072]
**Summary**				**0.002 [-0.018; 0.021]**

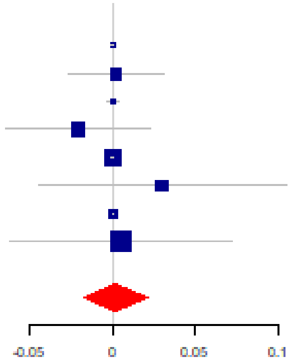

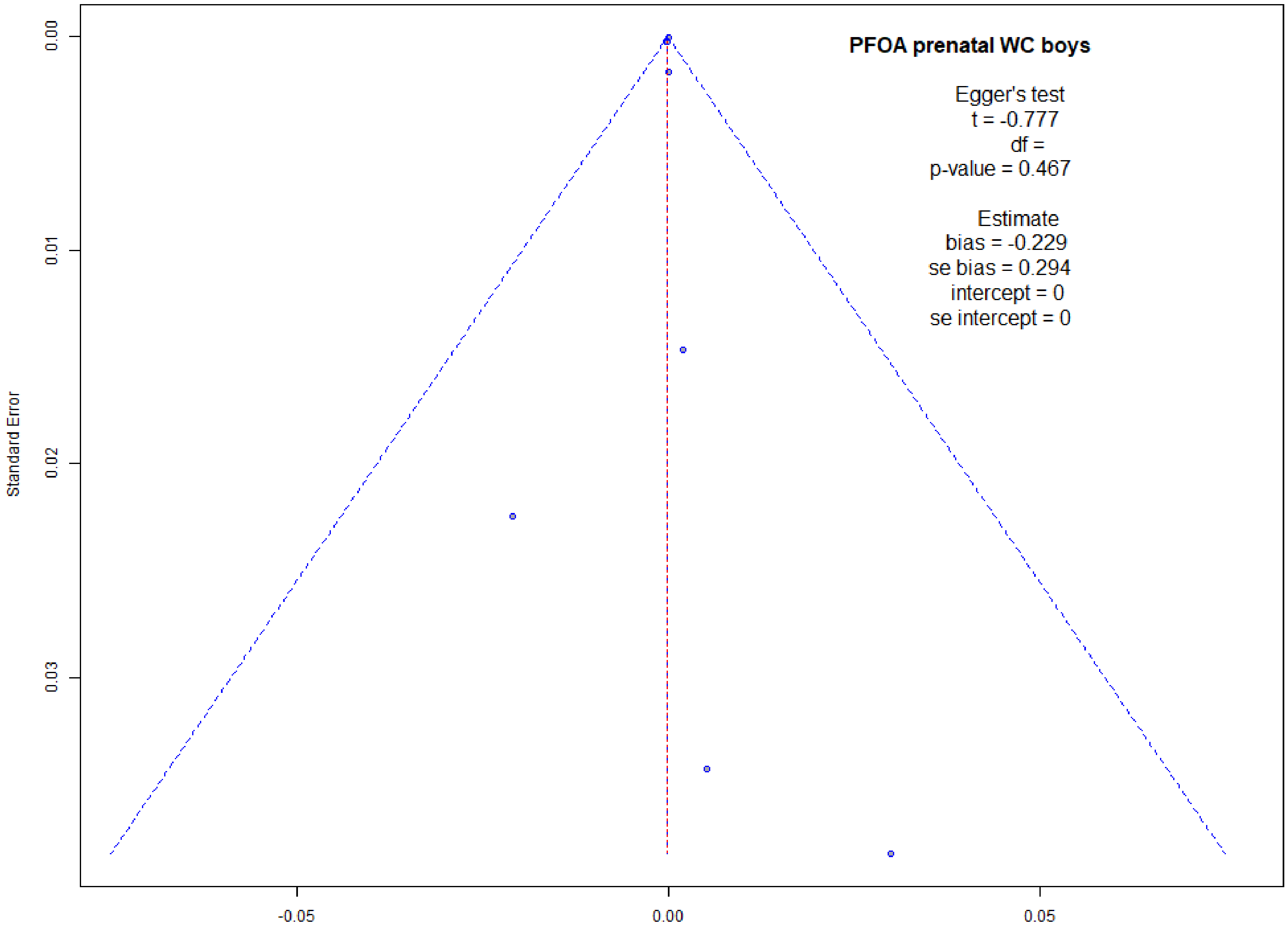
PFOA prenatal WC boys
Egger's test
t = -0.777
df =
p-value = 0.467
Estimate
bias = -0.229
se bias = 0.294
intercept = 0
se intercept = 0
Standard Error
0.00
0.01
0.02
0.03
-0.05
0.00
0.05

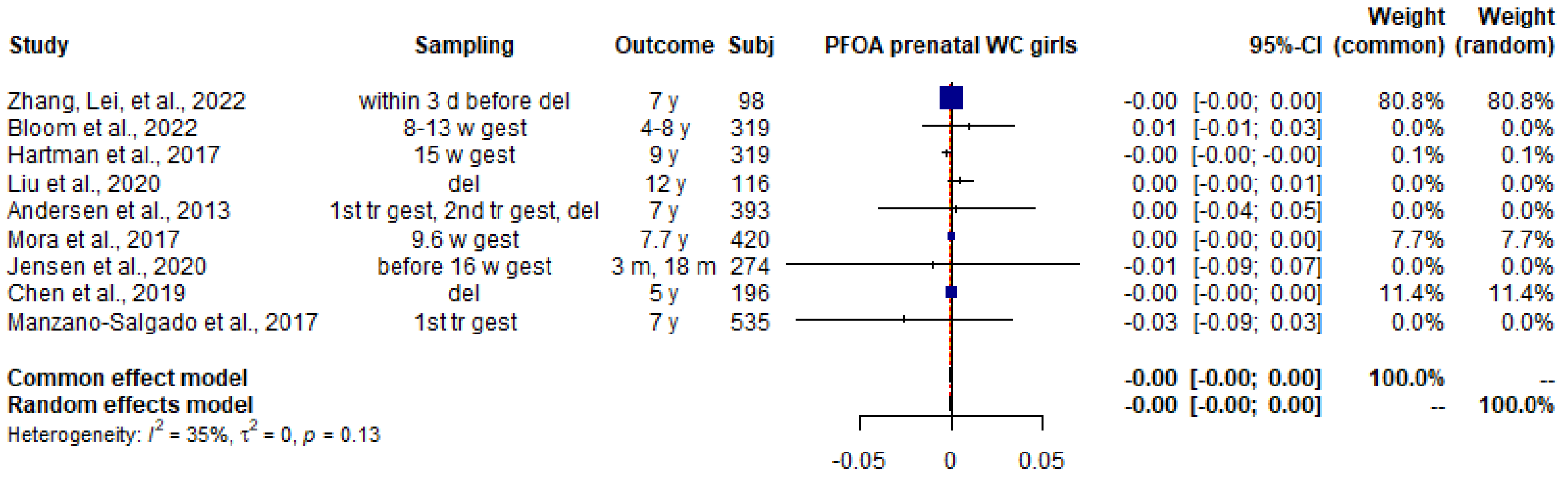
Study
Sampling
Outcome
Subj
PFOA prenatal WC girls
95%-CI
Weight (common)
Weight (random)
Zhang, Lei, et al., 2022 | within 3 d before del | 7 y | 98 | -0.00 [-0.00; 0.00] | 80.8% | 80.8%
Bloom et al., 2022 | 8-13 w gest | 4-8 y | 319 | 0.01 [-0.01; 0.03] | 0.0% | 0.0%
Hartman et al., 2017 | 15 w gest | 9 y | 319 | -0.00 [-0.00; -0.00] | 0.1% | 0.1%
Liu et al., 2020 | del | 12 y | 116 | 0.00 [-0.00; 0.01] | 0.0% | 0.0%
Andersen et al., 2013 | 1st tr gest, 2nd tr gest, del | 7 y | 393 | 0.00 [-0.04; 0.05] | 0.0% | 0.0%
Mora et al., 2017 | 9.6 w gest | 7.7 y | 420 | 0.00 [-0.00; 0.00] | 7.7% | 7.7%
Jensen et al., 2020 | before 16 w gest | 3 m, 18 m | 274 | -0.01 [-0.09; 0.07] | 0.0% | 0.0%
Chen et al., 2019 | del | 5 y | 196 | -0.00 [-0.00; 0.00] | 11.4% | 11.4%
Manzano-Salgado et al., 2017 | 1st tr gest | 7 y | 535 | -0.03 [-0.09; 0.03] | 0.0% | 0.0%
Common effect model | -0.00 [-0.00; 0.00] | 100.0% | --
Random effects model | -0.00 [-0.00; 0.00] | -- | 100.0%
Heterogeneity: I^2 = 35%, τ^2 = 0, p = 0.13
-0.05
0
0.05

Study	Sampling	Outcome	Subjects	Estimate [95% CI]
Zhang, Lei, et al., 2022	within 3 d before del	7 y	98	0.000 [0.000; 0.000]
Bloom et al., 2022	8-13 w gest	4-8 y	319	0.010 [-0.015; 0.035]
Hartman et al., 2017	15 w gest	9 y	319	-0.003 [-0.005; -0.001]
Liu et al., 2020	del	12 y	116	0.005 [-0.002; 0.012]
Andersen et al., 2013	1st tr gest, 2nd tr gest, del	7 y	393	0.003 [-0.040; 0.046]
Mora et al., 2017	9.6 w gest	7.7 y	420	0.000 [0.000; 0.000]
Jensen et al., 2020	before 16 w gest	3 m, 18 m	274	-0.010 [-0.090; 0.070]
Chen et al., 2019	del	5 y	196	0.000 [0.000; 0.000]
Manzano-Salgado et al., 2017	1st tr gest	7 y	535	-0.026 [-0.085; 0.034]
**Summary**				**-0.005 [-0.021; 0.011]**

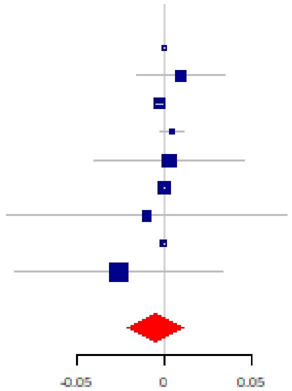

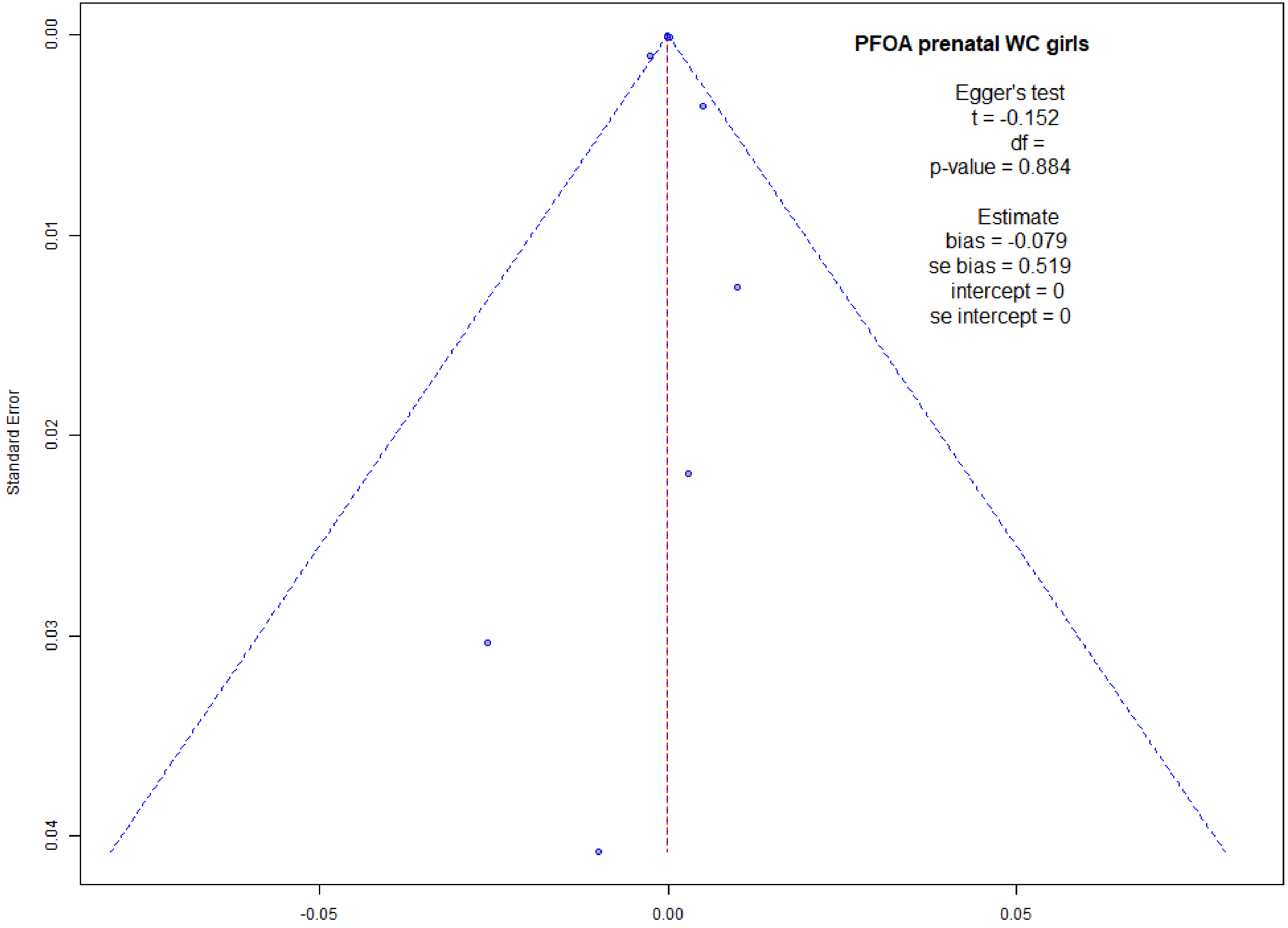
PFOA prenatal WC girls
Egger's test
t = -0.152
df =
p-value = 0.884
Estimate
bias = -0.079
se bias = 0.519
intercept = 0
se intercept = 0
Standard Error
0.00
0.01
0.02
0.03
0.04
-0.05
0.00
0.05

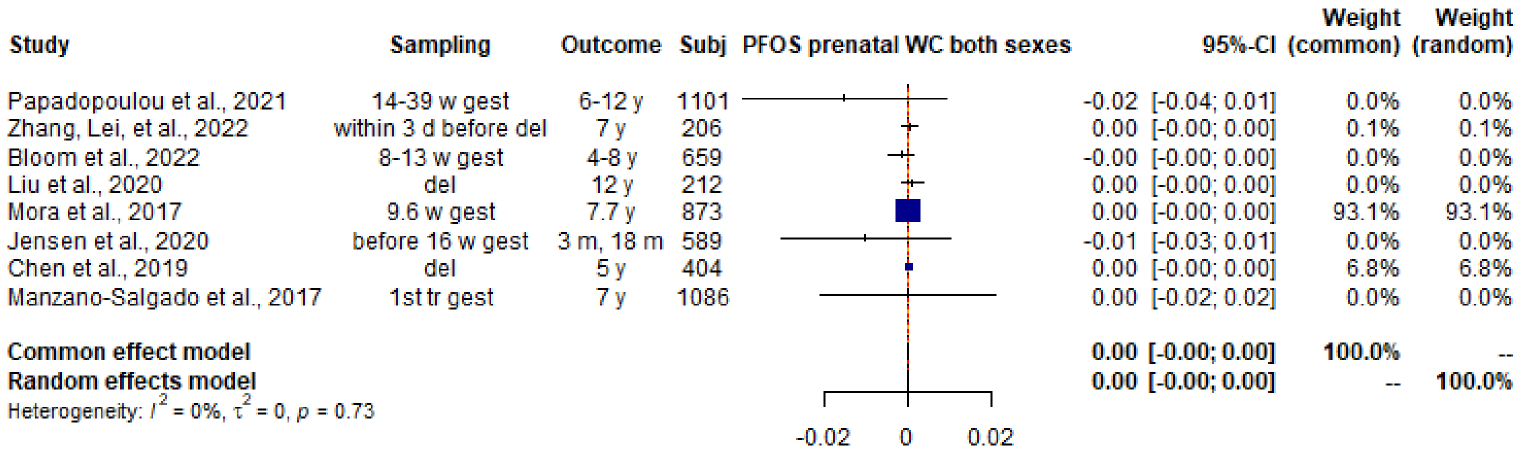
Study
Sampling
Outcome
Subj
PFOS prenatal WC both sexes
95%-CI
Weight (common)
Weight (random)
Papadopoulou et al., 2021 | 14-39 w gest | 6-12 y | 1101 | -0.02 [-0.04; 0.01] | 0.0% | 0.0%
Zhang, Lei, et al., 2022 | within 3 d before del | 7 y | 206 | 0.00 [-0.00; 0.00] | 0.1% | 0.1%
Bloom et al., 2022 | 8-13 w gest | 4-8 y | 659 | -0.00 [-0.00; 0.00] | 0.0% | 0.0%
Liu et al., 2020 | del | 12 y | 212 | 0.00 [-0.00; 0.00] | 0.0% | 0.0%
Mora et al., 2017 | 9.6 w gest | 7.7 y | 873 | 0.00 [-0.00; 0.00] | 93.1% | 93.1%
Jensen et al., 2020 | before 16 w gest | 3 m, 18 m | 589 | -0.01 [-0.03; 0.01] | 0.0% | 0.0%
Chen et al., 2019 | del | 5 y | 404 | 0.00 [-0.00; 0.00] | 6.8% | 6.8%
Manzano-Salgado et al., 2017 | 1st tr gest | 7 y | 1086 | 0.00 [-0.02; 0.02] | 0.0% | 0.0%
Common effect model | 0.00 [-0.00; 0.00] | 100.0% | --
Random effects model | 0.00 [-0.00; 0.00] | -- | 100.0%
Heterogeneity: I^2 = 0%, τ^2 = 0, p = 0.73
-0.02
0
0.02

Study	Sampling	Outcome	Subjects	Estimate [95% CI]
Papadopoulou et al., 2021	14-39 w gest	6-12 y	1101	-0.015 [-0.039; 0.009]
Zhang, Lei, et al., 2022	within 3 d before del	7 y	206	0.001 [-0.001; 0.002]
Bloom et al., 2022	8-13 w gest	4-8 y	659	-0.001 [-0.004; 0.001]
Liu et al., 2020	del	12 y	212	0.001 [-0.001; 0.004]
Mora et al., 2017	9.6 w gest	7.7 y	873	0.000 [0.000; 0.000]
Jensen et al., 2020	before 16 w gest	3 m, 18 m	589	-0.010 [-0.030; 0.010]
Chen et al., 2019	del	5 y	404	0.000 [0.000; 0.000]
Manzano-Salgado et al., 2017	1st tr gest	7 y	1086	0.000 [-0.021; 0.021]
**Summary**				**-0.005 [-0.012; 0.003]**

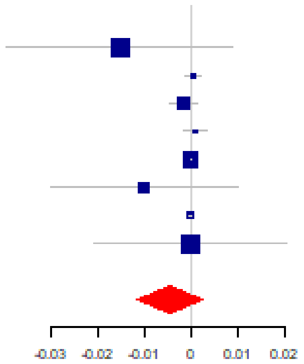

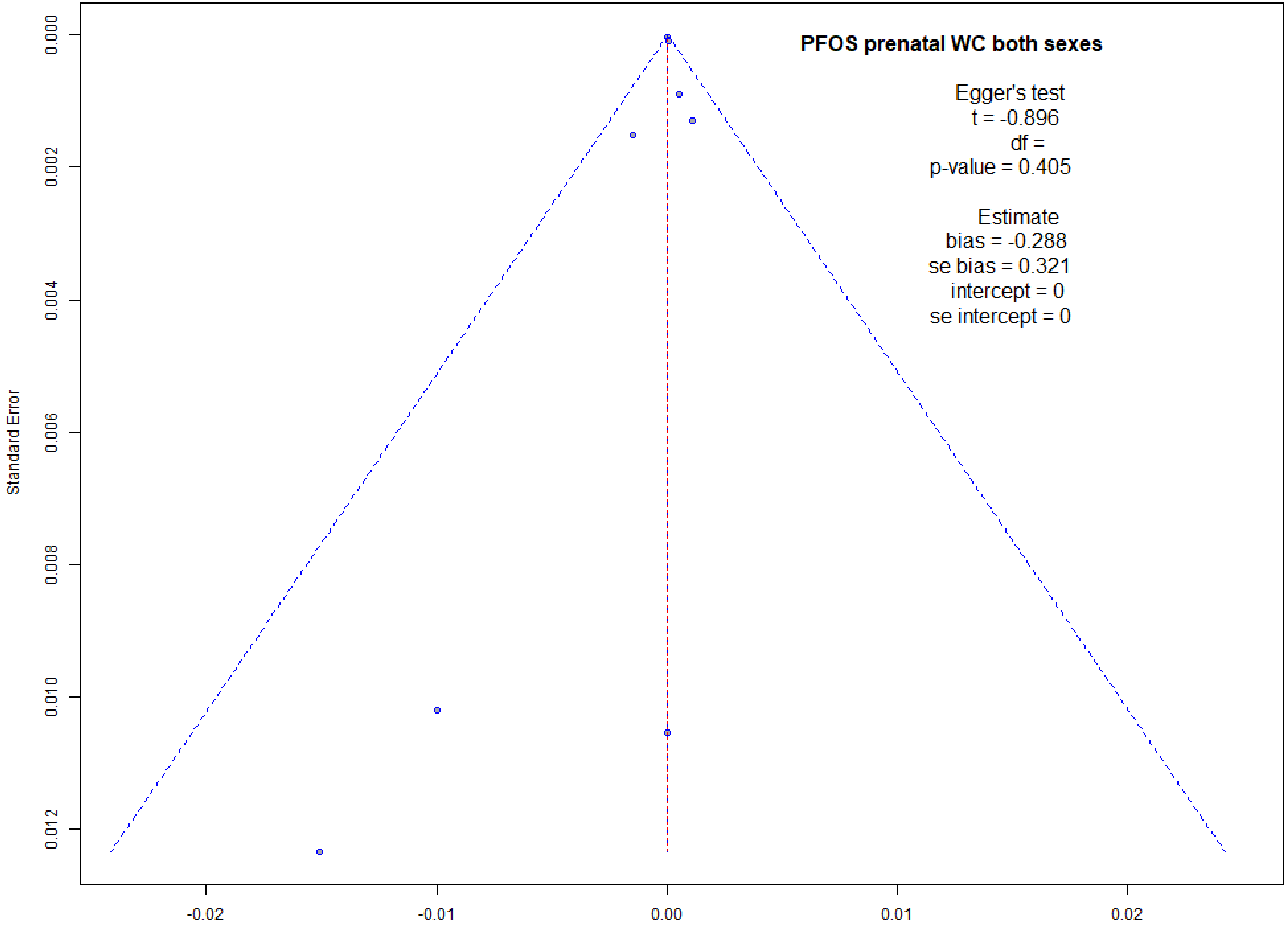
PFOS prenatal WC both sexes
Egger's test
t = -0.896
df =
p-value = 0.405
Estimate
bias = -0.288
se bias = 0.321
intercept = 0
se intercept = 0
Standard Error
0.000
0.002
0.004
0.006
0.008
0.010
0.012
-0.02
-0.01
0.00
0.01
0.02

Study	Sampling	Outcome	Subj	PFOS prenatal WC boys	95%-CI	Weight (common)	Weight (random)
Zhang, Lei, et al., 2022	within 3 d before del	7 y	108		0.00 [-0.00; 0.01]	0.0%	0.0%
Bloom et al., 2022	8-13 w gest	4-8 y	340		-0.00 [-0.00; 0.00]	0.0%	0.0%
Liu et al., 2020	del	12 y	96		0.00 [-0.00; 0.00]	0.1%	0.1%
Andersen et al., 2013	1st tr gest, 2nd tr gest, del	7 y	396		-0.00 [-0.01; 0.01]	0.0%	0.0%
Mora et al., 2017	9.6 w gest	7.7 y	453		-0.00 [-0.00; 0.00]	68.2%	68.2%
Jensen et al., 2020	before 16 w gest	3 m, 18 m	315		-0.01 [-0.04; 0.02]	0.0%	0.0%
Chen et al., 2019	del	5 y	208		-0.00 [-0.00; 0.00]	31.6%	31.6%
Manzano-Salgado et al., 2017	1st tr gest	7 y	551		-0.00 [-0.04; 0.03]	0.0%	0.0%
**Common effect model**					**-0.00 [-0.00; 0.00]**	**100.0%**	**--**
**Random effects model**					**-0.00 [-0.00; 0.00]**	**--**	**100.0%**

Heterogeneity: $I^2 = 0\%$, $\tau^2 = 0$, $p = 0.96$

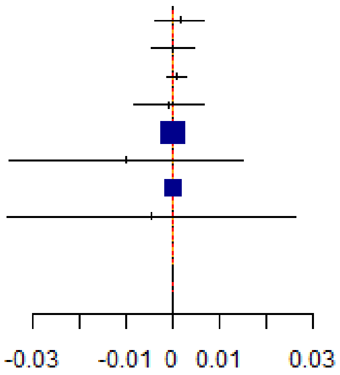

Study	Sampling	Outcome	Subjects	Estimate [95% CI]
Zhang, Lei, et al., 2022	within 3 d before del	7 y	108	0.002 [-0.004; 0.007]
Bloom et al., 2022	8-13 w gest	4-8 y	340	0.000 [-0.004; 0.004]
Liu et al., 2020	del	12 y	96	0.001 [-0.001; 0.003]
Andersen et al., 2013	1st tr gest, 2nd tr gest, del	7 y	396	-0.001 [-0.009; 0.006]
Mora et al., 2017	9.6 w gest	7.7 y	453	0.000 [0.000; 0.000]
Jensen et al., 2020	before 16 w gest	3 m, 18 m	315	-0.010 [-0.035; 0.015]
Chen et al., 2019	del	5 y	208	0.000 [0.000; 0.000]
Manzano-Salgado et al., 2017	1st tr gest	7 y	551	-0.005 [-0.036; 0.026]
**Summary**				**-0.002 [-0.010; 0.005]**

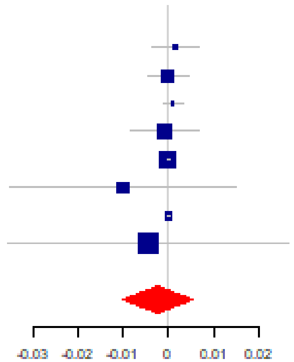

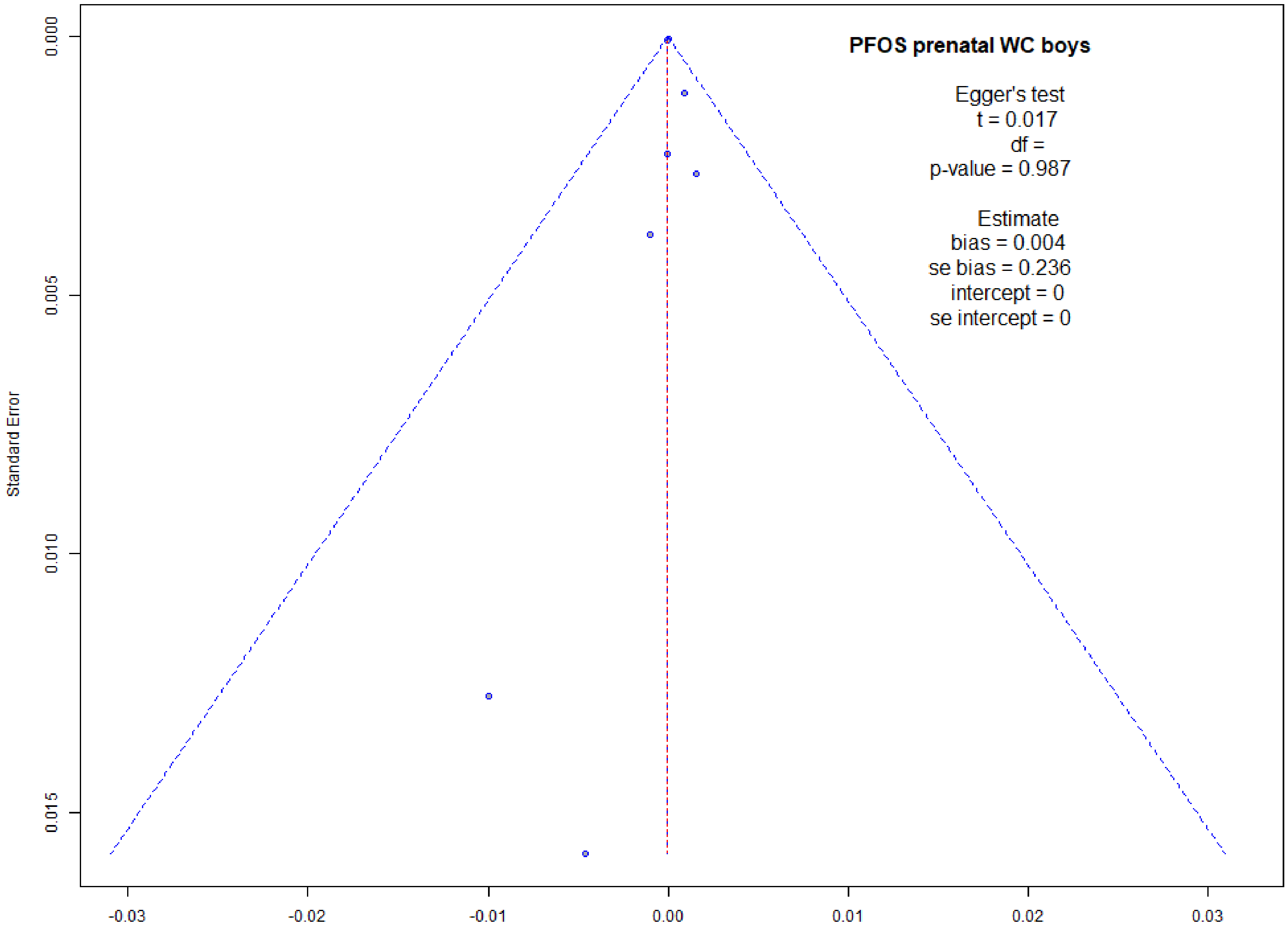
PFOS prenatal WC boys
Egger's test
t = 0.017
df =
p-value = 0.987
Estimate
bias = 0.004
se bias = 0.236
intercept = 0
se intercept = 0
Standard Error
0.000
0.005
0.010
0.015
-0.03
-0.02
-0.01
0.00
0.01
0.02
0.03

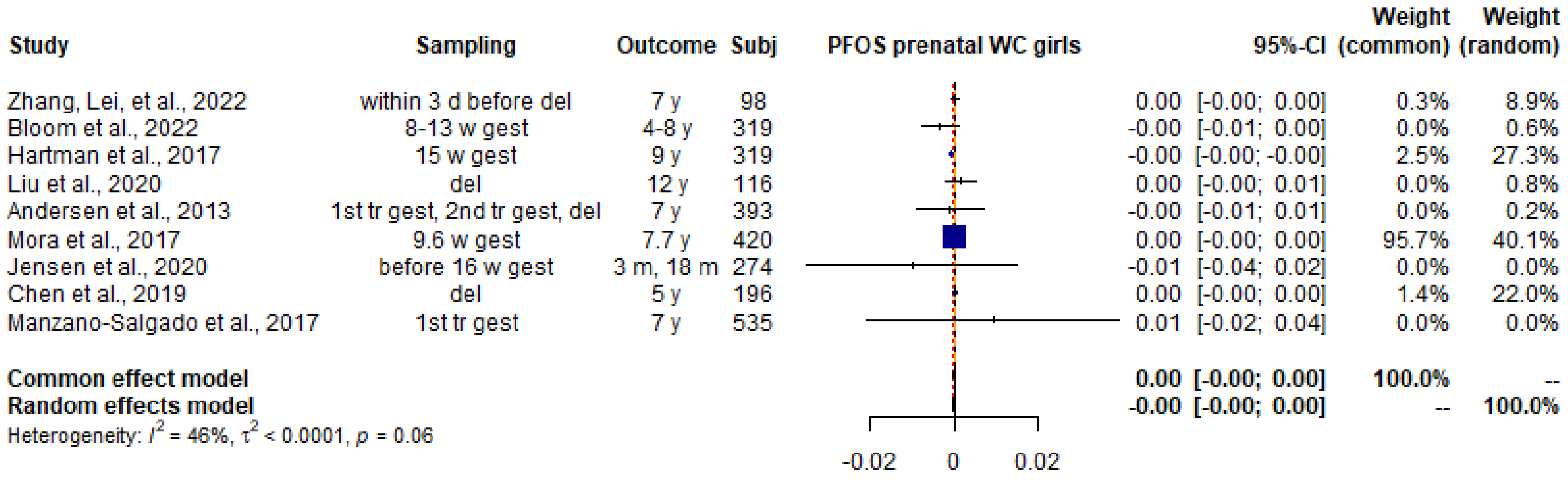
Study
Sampling
Outcome
Subj
PFOS prenatal WC girls
95%-CI
Weight (common)
Weight (random)
Zhang, Lei, et al., 2022 | within 3 d before del | 7 y | 98 | 0.00 [-0.00; 0.00] | 0.3% | 8.9%
Bloom et al., 2022 | 8-13 w gest | 4-8 y | 319 | -0.00 [-0.01; 0.00] | 0.0% | 0.6%
Hartman et al., 2017 | 15 w gest | 9 y | 319 | -0.00 [-0.00; -0.00] | 2.5% | 27.3%
Liu et al., 2020 | del | 12 y | 116 | 0.00 [-0.00; 0.01] | 0.0% | 0.8%
Andersen et al., 2013 | 1st tr gest, 2nd tr gest, del | 7 y | 393 | -0.00 [-0.01; 0.01] | 0.0% | 0.2%
Mora et al., 2017 | 9.6 w gest | 7.7 y | 420 | 0.00 [-0.00; 0.00] | 95.7% | 40.1%
Jensen et al., 2020 | before 16 w gest | 3 m, 18 m | 274 | -0.01 [-0.04; 0.02] | 0.0% | 0.0%
Chen et al., 2019 | del | 5 y | 196 | 0.00 [-0.00; 0.00] | 1.4% | 22.0%
Manzano-Salgado et al., 2017 | 1st tr gest | 7 y | 535 | 0.01 [-0.02; 0.04] | 0.0% | 0.0%
Common effect model | 0.00 [-0.00; 0.00] | 100.0% | --
Random effects model | -0.00 [-0.00; 0.00] | -- | 100.0%
Heterogeneity: $I^2 = 46\%$, $\tau^2 < 0.0001$, $p = 0.06$
-0.02
0
0.02

Study	Sampling	Outcome	Subjects	Estimate [95% CI]
Zhang, Lei, et al., 2022	within 3 d before del	7 y	98	0.000 [-0.001; 0.001]
Bloom et al., 2022	8-13 w gest	4-8 y	319	-0.003 [-0.008; 0.001]
Hartman et al., 2017	15 w gest	9 y	319	-0.001 [-0.001; 0.000]
Liu et al., 2020	del	12 y	116	0.002 [-0.002; 0.005]
Andersen et al., 2013	1st tr gest, 2nd tr gest, del	7 y	393	-0.001 [-0.009; 0.007]
Mora et al., 2017	9.6 w gest	7.7 y	420	0.000 [0.000; 0.000]
Jensen et al., 2020	before 16 w gest	3 m, 18 m	274	-0.010 [-0.035; 0.015]
Chen et al., 2019	del	5 y	196	0.000 [0.000; 0.001]
Manzano-Salgado et al., 2017	1st tr gest	7 y	535	0.009 [-0.021; 0.039]
**Summary**				**0.000 [-0.006; 0.007]**

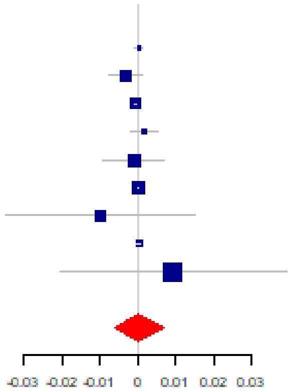

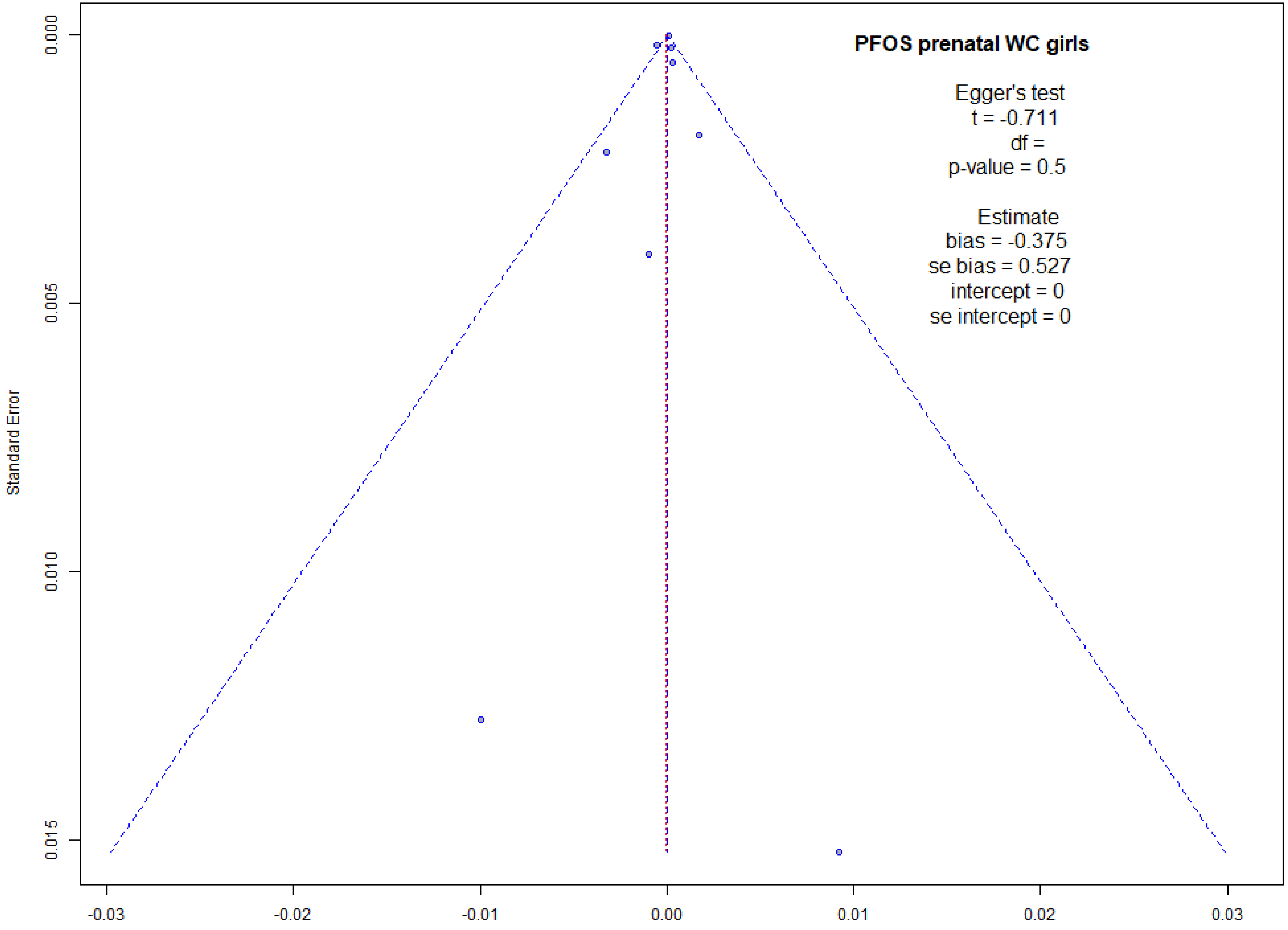
PFOS prenatal WC girls
Egger's test
t = -0.711
df =
p-value = 0.5
Estimate
bias = -0.375
se bias = 0.527
intercept = 0
se intercept = 0
Standard Error
0.000
0.005
0.010
0.015
-0.03
-0.02
-0.01
0.00
0.01
0.02
0.03

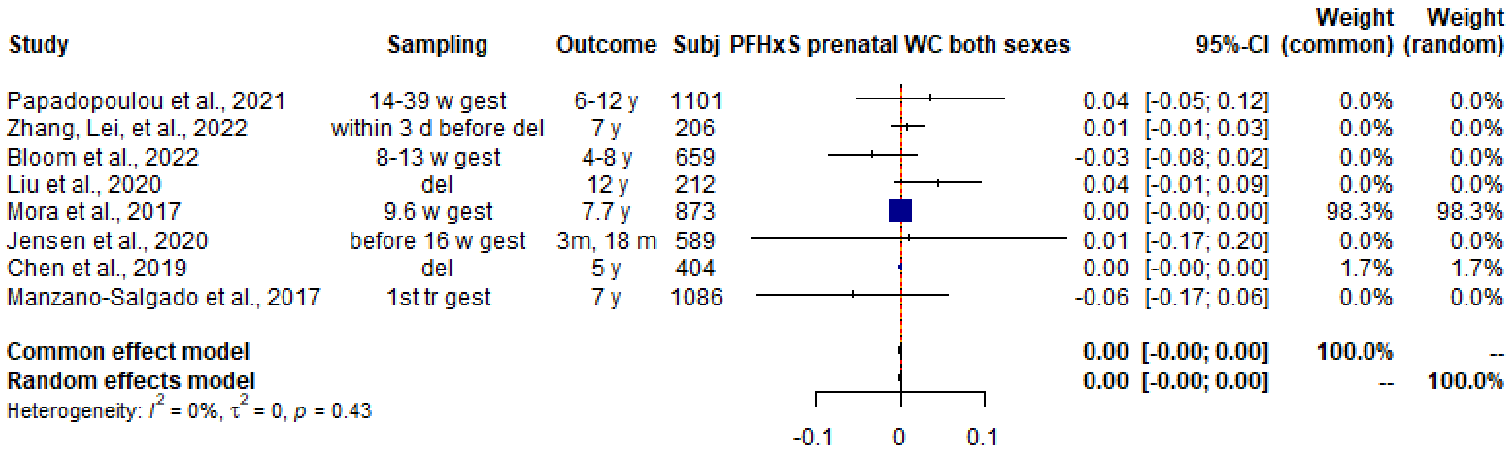
Study
Sampling
Outcome
Subj
PFHxS prenatal WC both sexes
95%-CI
Weight (common)
Weight (random)
Papadopoulou et al., 2021 | 14-39 w gest | 6-12 y | 1101 | 0.04 [-0.05; 0.12] | 0.0% | 0.0%
Zhang, Lei, et al., 2022 | within 3 d before del | 7 y | 206 | 0.01 [-0.01; 0.03] | 0.0% | 0.0%
Bloom et al., 2022 | 8-13 w gest | 4-8 y | 659 | -0.03 [-0.08; 0.02] | 0.0% | 0.0%
Liu et al., 2020 | del | 12 y | 212 | 0.04 [-0.01; 0.09] | 0.0% | 0.0%
Mora et al., 2017 | 9.6 w gest | 7.7 y | 873 | 0.00 [-0.00; 0.00] | 98.3% | 98.3%
Jensen et al., 2020 | before 16 w gest | 3m, 18 m | 589 | 0.01 [-0.17; 0.20] | 0.0% | 0.0%
Chen et al., 2019 | del | 5 y | 404 | 0.00 [-0.00; 0.00] | 1.7% | 1.7%
Manzano-Salgado et al., 2017 | 1st tr gest | 7 y | 1086 | -0.06 [-0.17; 0.06] | 0.0% | 0.0%
Common effect model | 0.00 [-0.00; 0.00] | 100.0% | --
Random effects model | 0.00 [-0.00; 0.00] | -- | 100.0%
Heterogeneity: I^2 = 0%, τ^2 = 0, p = 0.43
-0.1
0
0.1

Study	Sampling	Outcome	Subjects	Estimate [95% CI]
Papadopoulou et al., 2021	14-39 w gest	6-12 y	1101	0.036 [-0.052; 0.123]
Zhang, Lei, et al., 2022	within 3 d before del	7 y	206	0.009 [-0.010; 0.028]
Bloom et al., 2022	8-13 w gest	4-8 y	659	-0.032 [-0.084; 0.019]
Liu et al., 2020	del	12 y	212	0.044 [-0.007; 0.094]
Mora et al., 2017	9.6 w gest	7.7 y	873	0.000 [0.000; 0.000]
Jensen et al., 2020	before 16 w gest	3m, 18 m	589	0.010 [-0.175; 0.195]
Chen et al., 2019	del	5 y	404	0.000 [-0.001; 0.002]
Manzano-Salgado et al., 2017	1st tr gest	7 y	1086	-0.056 [-0.168; 0.056]
**Summary**				**-0.005 [-0.043; 0.033]**

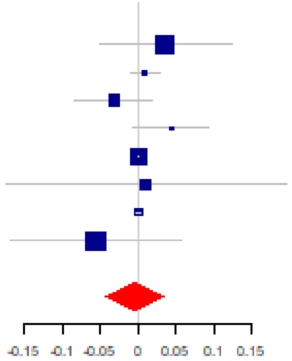

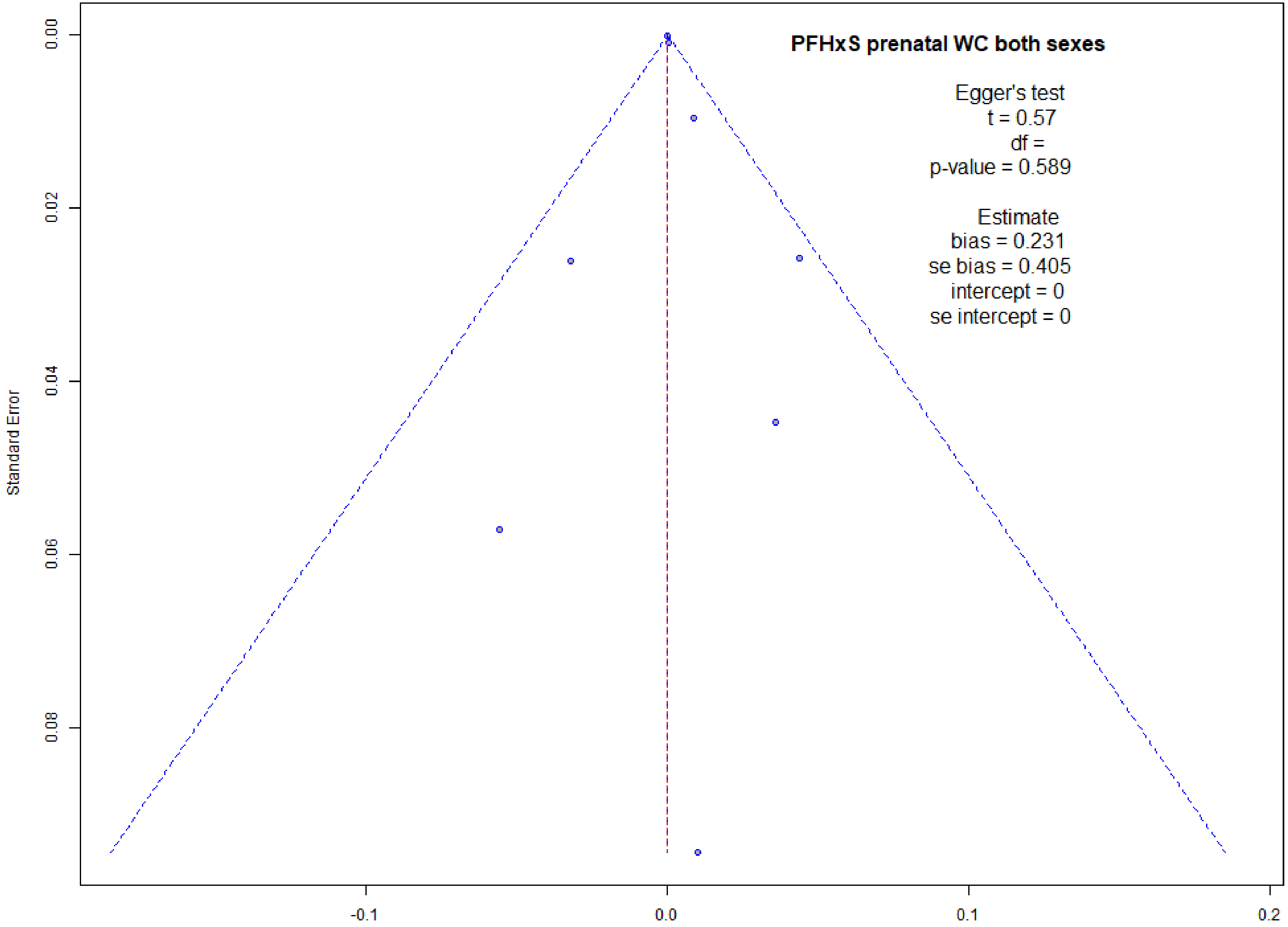
PFHxS prenatal WC both sexes
Egger's test
t = 0.57
df =
p-value = 0.589
Estimate
bias = 0.231
se bias = 0.405
intercept = 0
se intercept = 0
Standard Error
0.00
0.02
0.04
0.06
0.08
-0.1
0.0
0.1
0.2

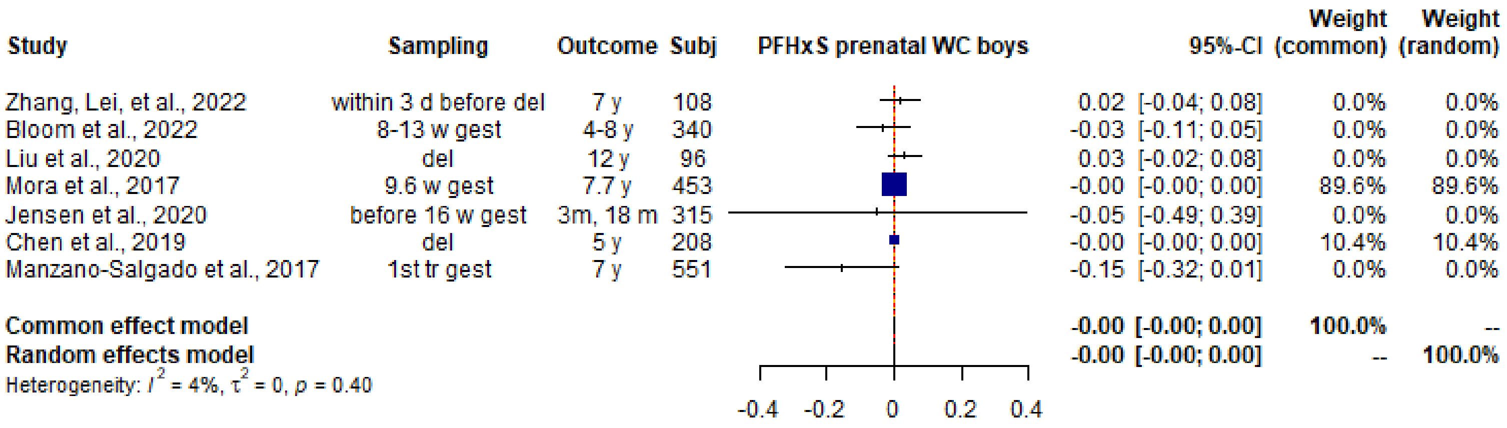
Study
Sampling
Outcome
Subj
PFHxS prenatal WC boys
95%-CI
Weight (common)
Weight (random)
Zhang, Lei, et al., 2022 within 3 d before del 7 y 108 0.02 [-0.04; 0.08] 0.0% 0.0%
Bloom et al., 2022 8-13 w gest 4-8 y 340 -0.03 [-0.11; 0.05] 0.0% 0.0%
Liu et al., 2020 del 12 y 96 0.03 [-0.02; 0.08] 0.0% 0.0%
Mora et al., 2017 9.6 w gest 7.7 y 453 -0.00 [-0.00; 0.00] 89.6% 89.6%
Jensen et al., 2020 before 16 w gest 3m, 18 m 315 -0.05 [-0.49; 0.39] 0.0% 0.0%
Chen et al., 2019 del 5 y 208 -0.00 [-0.00; 0.00] 10.4% 10.4%
Manzano-Salgado et al., 2017 1st tr gest 7 y 551 -0.15 [-0.32; 0.01] 0.0% 0.0%
Common effect model -0.00 [-0.00; 0.00] 100.0% --
Random effects model -0.00 [-0.00; 0.00] -- 100.0%
Heterogeneity: I^2 = 4%, τ^2 = 0, p = 0.40
-0.4
-0.2
0
0.2
0.4

Study	Sampling	Outcome	Subjects	Estimate [95% CI]
Zhang, Lei, et al., 2022	within 3 d before del	7 y	108	0.018 [-0.041; 0.077]
Bloom et al., 2022	8-13 w gest	4-8 y	340	-0.032 [-0.110; 0.046]
Liu et al., 2020	del	12 y	96	0.031 [-0.015; 0.078]
Mora et al., 2017	9.6 w gest	7.7 y	453	0.000 [-0.001; 0.000]
Jensen et al., 2020	before 16 w gest	3m, 18 m	315	-0.050 [-0.490; 0.390]
Chen et al., 2019	del	5 y	208	0.000 [-0.002; 0.001]
Manzano-Salgado et al., 2017	1st tr gest	7 y	551	-0.154 [-0.322; 0.014]
**Summary**				**-0.052 [-0.133; 0.030]**

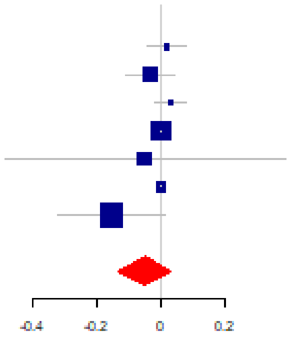

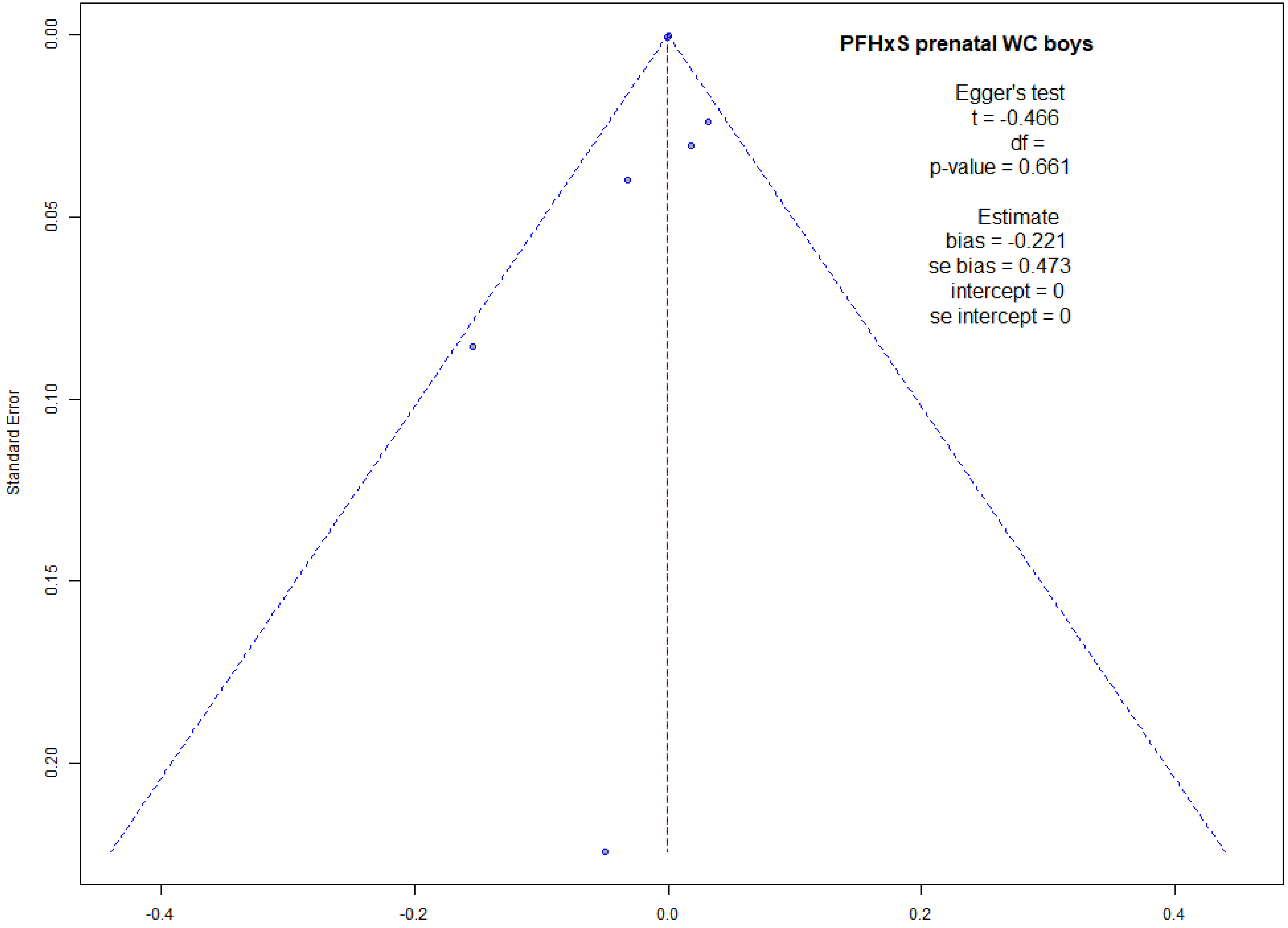
PFHxS prenatal WC boys
Egger's test
t = -0.466
df =
p-value = 0.661
Estimate
bias = -0.221
se bias = 0.473
intercept = 0
se intercept = 0
Standard Error
0.00
0.05
0.10
0.15
0.20
-0.4
-0.2
0.0
0.2
0.4

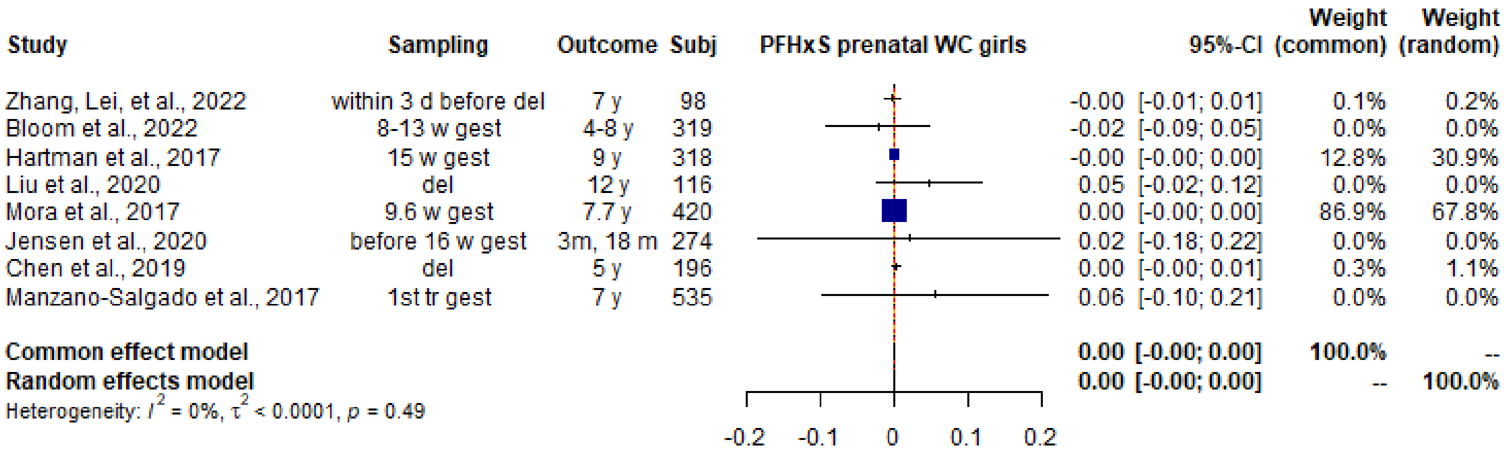
Study | Sampling | Outcome | Subj | PFHxS prenatal WC girls | 95%-CI | Weight (common) | Weight (random)
Zhang, Lei, et al., 2022 | within 3 d before del | 7 y | 98 | -0.00 [-0.01; 0.01] | 0.1% | 0.2%
Bloom et al., 2022 | 8-13 w gest | 4-8 y | 319 | -0.02 [-0.09; 0.05] | 0.0% | 0.0%
Hartman et al., 2017 | 15 w gest | 9 y | 318 | -0.00 [-0.00; 0.00] | 12.8% | 30.9%
Liu et al., 2020 | del | 12 y | 116 | 0.05 [-0.02; 0.12] | 0.0% | 0.0%
Mora et al., 2017 | 9.6 w gest | 7.7 y | 420 | 0.00 [-0.00; 0.00] | 86.9% | 67.8%
Jensen et al., 2020 | before 16 w gest | 3m, 18 m | 274 | 0.02 [-0.18; 0.22] | 0.0% | 0.0%
Chen et al., 2019 | del | 5 y | 196 | 0.00 [-0.00; 0.01] | 0.3% | 1.1%
Manzano-Salgado et al., 2017 | 1st tr gest | 7 y | 535 | 0.06 [-0.10; 0.21] | 0.0% | 0.0%
Common effect model | 0.00 [-0.00; 0.00] | 100.0% | --
Random effects model | 0.00 [-0.00; 0.00] | -- | 100.0%
Heterogeneity: I^2 = 0%, τ^2 < 0.0001, p = 0.49
-0.2 -0.1 0 0.1 0.2

Study	Sampling	Outcome	Subjects	Estimate [95% CI]
Zhang, Lei, et al., 2022	within 3 d before del	7 y	98	-0.003 [-0.012; 0.007]
Bloom et al., 2022	8-13 w gest	4-8 y	319	-0.022 [-0.092; 0.049]
Hartman et al., 2017	15 w gest	9 y	318	0.000 [-0.001; 0.000]
Liu et al., 2020	del	12 y	116	0.047 [-0.024; 0.118]
Mora et al., 2017	9.6 w gest	7.7 y	420	0.000 [0.000; 0.000]
Jensen et al., 2020	before 16 w gest	3m, 18 m	274	0.020 [-0.185; 0.225]
Chen et al., 2019	del	5 y	196	0.003 [-0.002; 0.007]
Manzano-Salgado et al., 2017	1st tr gest	7 y	535	0.056 [-0.098; 0.210]
**Summary**				**0.015 [-0.030; 0.060]**

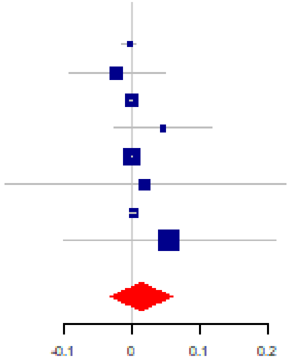

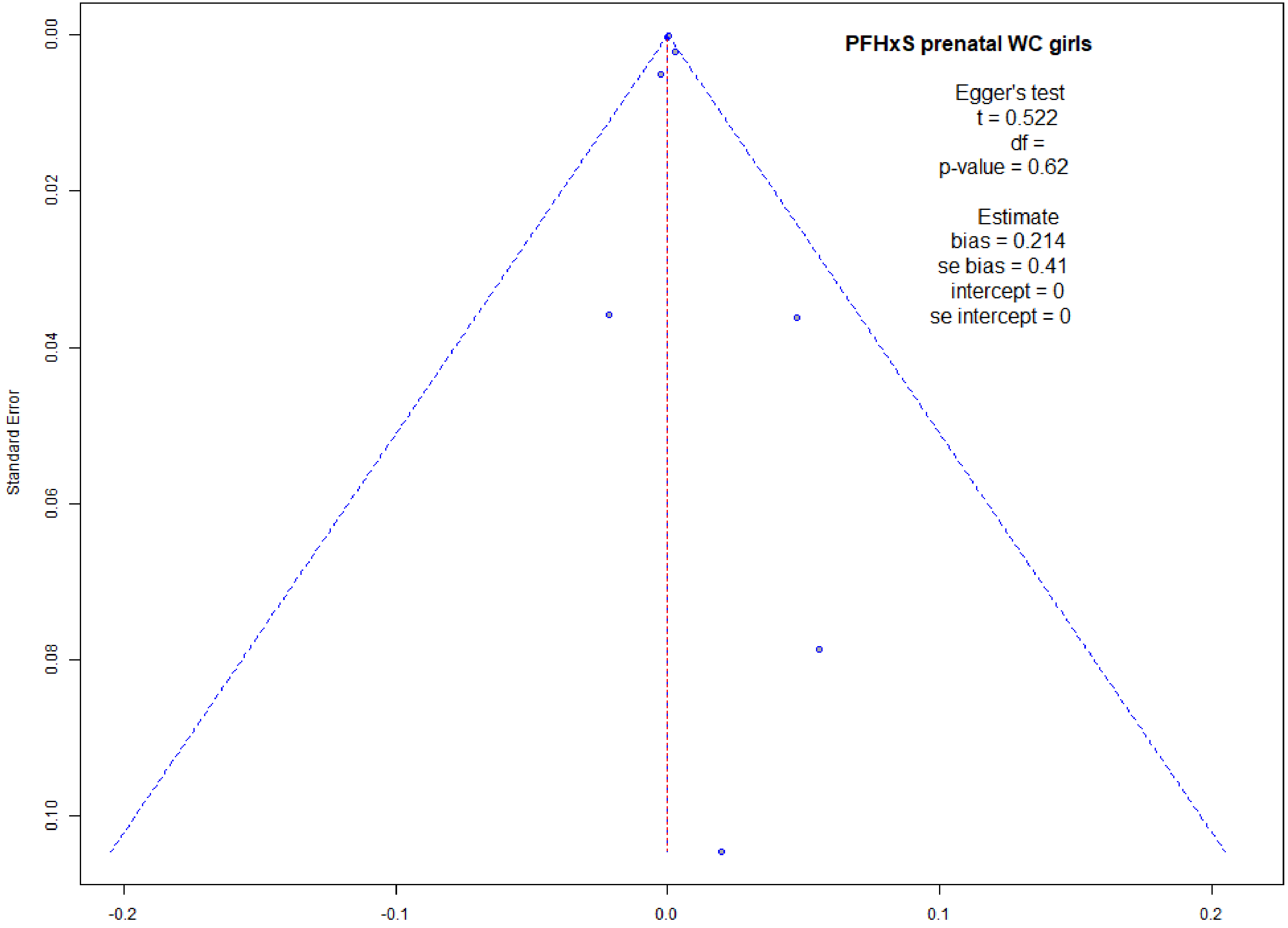
PFHxS prenatal WC girls
Egger's test
t = 0.522
df =
p-value = 0.62
Estimate
bias = 0.214
se bias = 0.41
intercept = 0
se intercept = 0
Standard Error
0.00
0.02
0.04
0.06
0.08
0.10
-0.2
-0.1
0.0
0.1
0.2

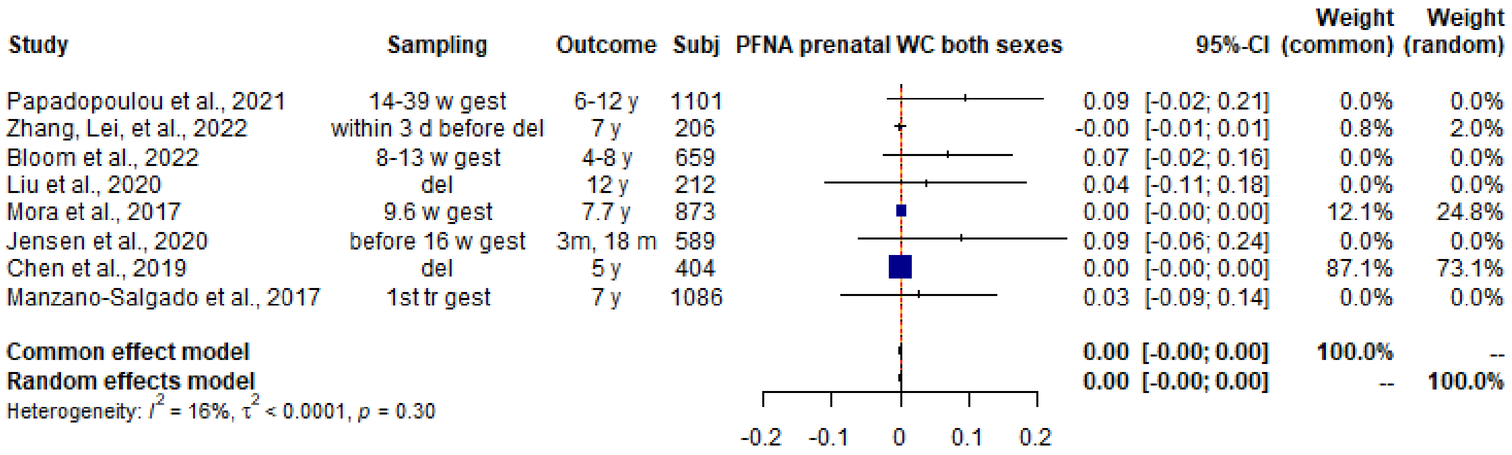
Study
Sampling
Outcome
Subj
PFNA prenatal WC both sexes
95%-CI
Weight (common)
Weight (random)
Papadopoulou et al., 2021 | 14-39 w gest | 6-12 y | 1101 | 0.09 [-0.02; 0.21] | 0.0% | 0.0%
Zhang, Lei, et al., 2022 | within 3 d before del | 7 y | 206 | -0.00 [-0.01; 0.01] | 0.8% | 2.0%
Bloom et al., 2022 | 8-13 w gest | 4-8 y | 659 | 0.07 [-0.02; 0.16] | 0.0% | 0.0%
Liu et al., 2020 | del | 12 y | 212 | 0.04 [-0.11; 0.18] | 0.0% | 0.0%
Mora et al., 2017 | 9.6 w gest | 7.7 y | 873 | 0.00 [-0.00; 0.00] | 12.1% | 24.8%
Jensen et al., 2020 | before 16 w gest | 3m, 18 m | 589 | 0.09 [-0.06; 0.24] | 0.0% | 0.0%
Chen et al., 2019 | del | 5 y | 404 | 0.00 [-0.00; 0.00] | 87.1% | 73.1%
Manzano-Salgado et al., 2017 | 1st tr gest | 7 y | 1086 | 0.03 [-0.09; 0.14] | 0.0% | 0.0%
Common effect model | 0.00 [-0.00; 0.00] | 100.0% | --
Random effects model | 0.00 [-0.00; 0.00] | -- | 100.0%
Heterogeneity: I^2 = 16%, τ^2 < 0.0001, p = 0.30
-0.2
-0.1
0
0.1
0.2

Study	Sampling	Outcome	Subjects	Estimate [95% CI]
Papadopoulou et al., 2021	14-39 w gest	6-12 y	1101	0.094 [-0.019; 0.207]
Zhang, Lei, et al., 2022	within 3 d before del	7 y	206	-0.001 [-0.008; 0.006]
Bloom et al., 2022	8-13 w gest	4-8 y	659	0.069 [-0.023; 0.162]
Liu et al., 2020	del	12 y	212	0.037 [-0.109; 0.183]
Mora et al., 2017	9.6 w gest	7.7 y	873	0.001 [0.000; 0.003]
Jensen et al., 2020	before 16 w gest	3m, 18 m	589	0.090 [-0.060; 0.240]
Chen et al., 2019	del	5 y	404	0.000 [-0.001; 0.001]
Manzano-Salgado et al., 2017	1st tr gest	7 y	1086	0.027 [-0.086; 0.140]
**Summary**				**0.047 [0.006; 0.087]**

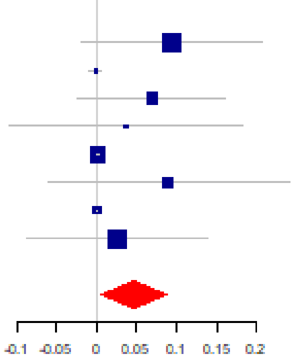

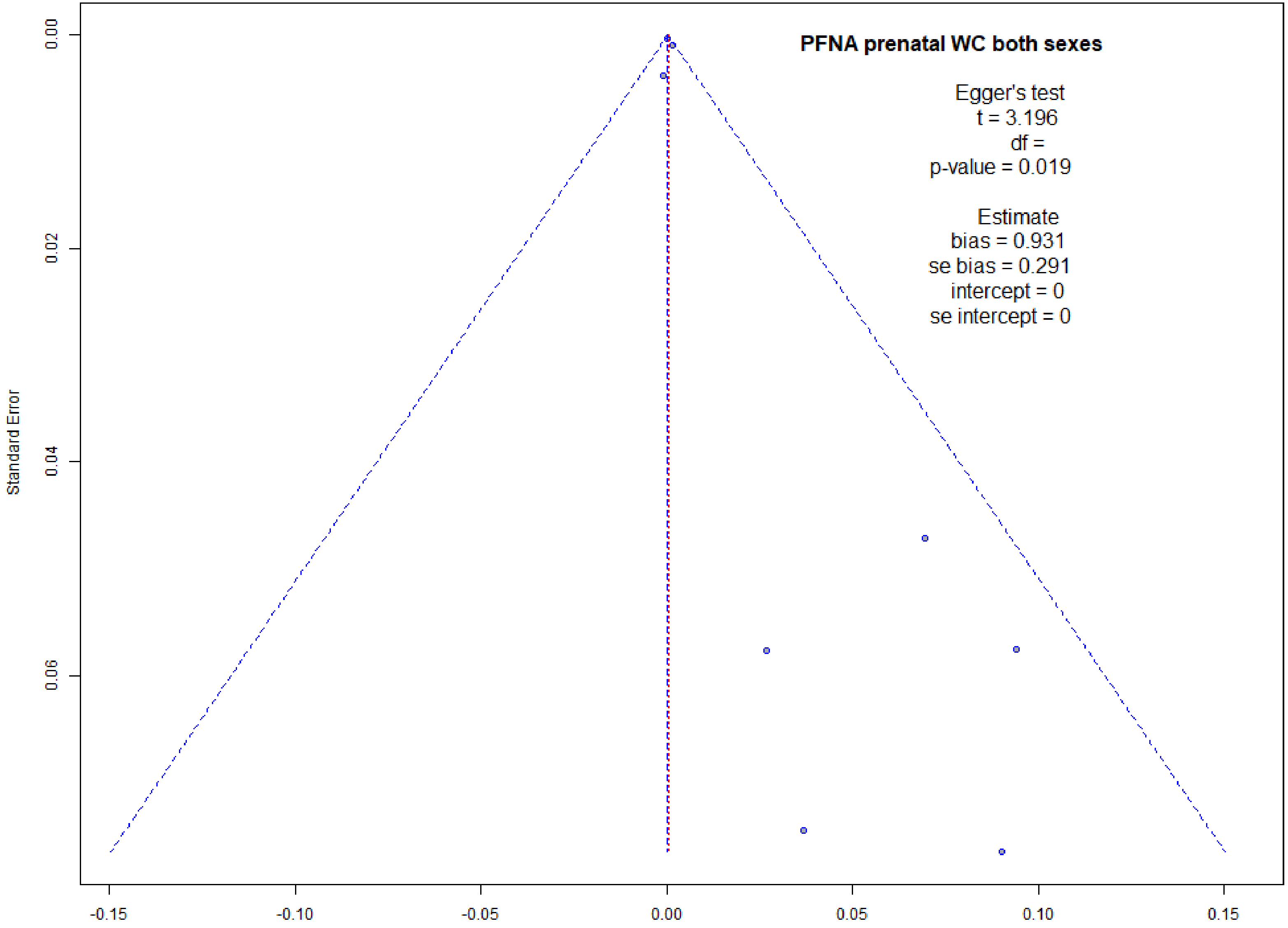
PFNA prenatal WC both sexes
Egger's test
t = 3.196
df =
p-value = 0.019
Estimate
bias = 0.931
se bias = 0.291
intercept = 0
se intercept = 0
Standard Error
0.00
0.02
0.04
0.06
-0.15
-0.10
-0.05
0.00
0.05
0.10
0.15

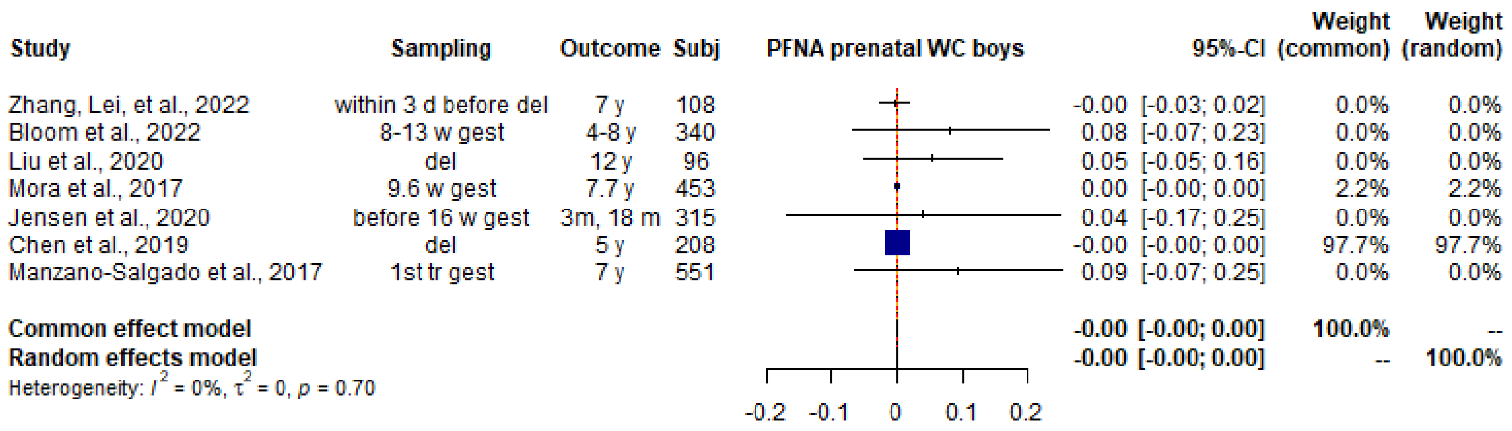
Study
Sampling
Outcome
Subj
PFNA prenatal WC boys
95%-CI
Weight (common)
Weight (random)
Zhang, Lei, et al., 2022 | within 3 d before del | 7 y | 108 | -0.00 [-0.03; 0.02] | 0.0% | 0.0%
Bloom et al., 2022 | 8-13 w gest | 4-8 y | 340 | 0.08 [-0.07; 0.23] | 0.0% | 0.0%
Liu et al., 2020 | del | 12 y | 96 | 0.05 [-0.05; 0.16] | 0.0% | 0.0%
Mora et al., 2017 | 9.6 w gest | 7.7 y | 453 | 0.00 [-0.00; 0.00] | 2.2% | 2.2%
Jensen et al., 2020 | before 16 w gest | 3m, 18 m | 315 | 0.04 [-0.17; 0.25] | 0.0% | 0.0%
Chen et al., 2019 | del | 5 y | 208 | -0.00 [-0.00; 0.00] | 97.7% | 97.7%
Manzano-Salgado et al., 2017 | 1st tr gest | 7 y | 551 | 0.09 [-0.07; 0.25] | 0.0% | 0.0%
Common effect model | -0.00 [-0.00; 0.00] | 100.0% | --
Random effects model | -0.00 [-0.00; 0.00] | -- | 100.0%
Heterogeneity: I² = 0%, τ² = 0, p = 0.70
-0.2
-0.1
0
0.1
0.2

Study	Sampling	Outcome	Subjects	Estimate [95% CI]
Zhang, Lei, et al., 2022	within 3 d before del	7 y	108	-0.004 [-0.027; 0.019]
Bloom et al., 2022	8-13 w gest	4-8 y	340	0.081 [-0.069; 0.231]
Liu et al., 2020	del	12 y	96	0.054 [-0.052; 0.159]
Mora et al., 2017	9.6 w gest	7.7 y	453	0.001 [-0.003; 0.004]
Jensen et al., 2020	before 16 w gest	3m, 18 m	315	0.040 [-0.170; 0.250]
Chen et al., 2019	del	5 y	208	0.000 [-0.001; 0.000]
Manzano-Salgado et al., 2017	1st tr gest	7 y	551	0.093 [-0.067; 0.253]
**Summary**				**0.047 [-0.012; 0.105]**

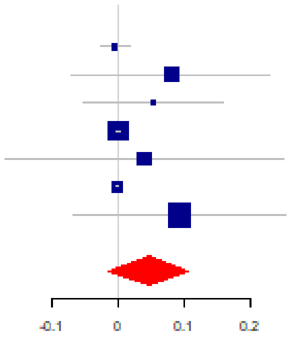

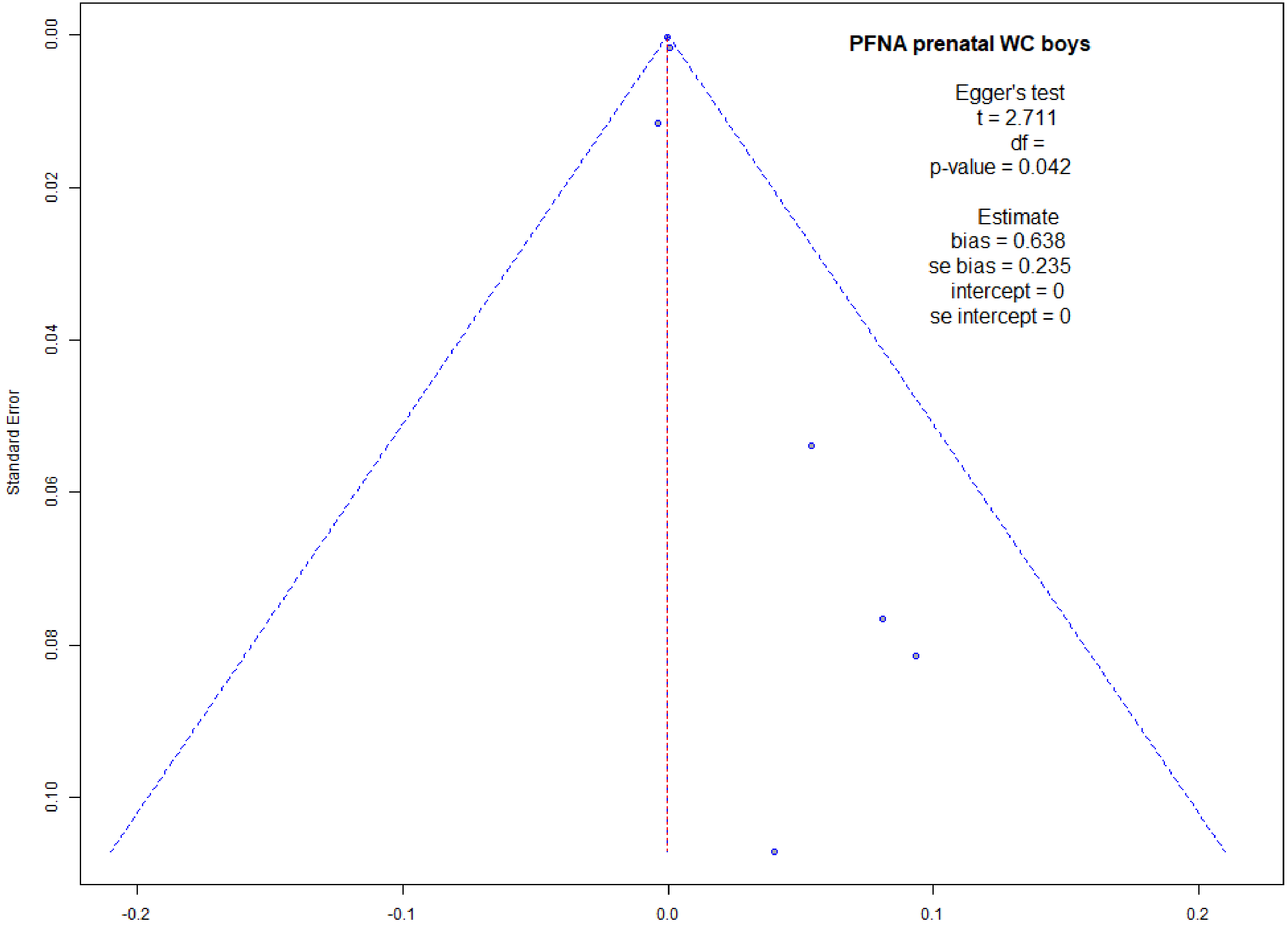
PFNA prenatal WC boys
Egger's test
t = 2.711
df =
p-value = 0.042
Estimate
bias = 0.638
se bias = 0.235
intercept = 0
se intercept = 0
Standard Error
0.00
0.02
0.04
0.06
0.08
0.10
-0.2
-0.1
0.0
0.1
0.2

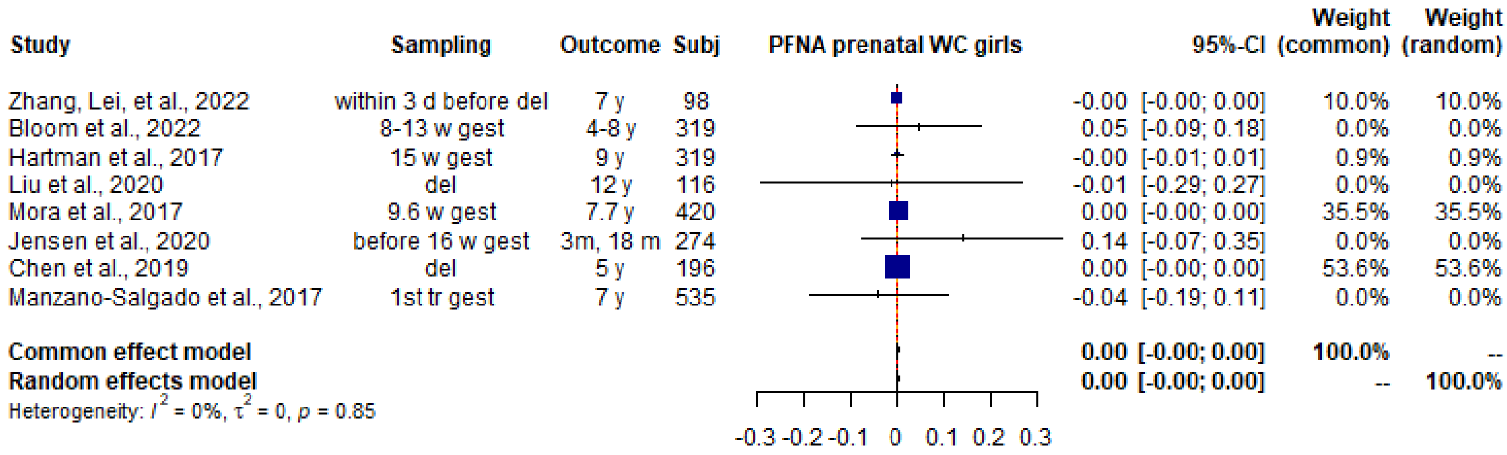
Study
Sampling
Outcome
Subj
PFNA prenatal WC girls
95%-CI
Weight (common)
Weight (random)
Zhang, Lei, et al., 2022 within 3 d before del 7 y 98 -0.00 [-0.00; 0.00] 10.0% 10.0%
Bloom et al., 2022 8-13 w gest 4-8 y 319 0.05 [-0.09; 0.18] 0.0% 0.0%
Hartman et al., 2017 15 w gest 9 y 319 -0.00 [-0.01; 0.01] 0.9% 0.9%
Liu et al., 2020 del 12 y 116 -0.01 [-0.29; 0.27] 0.0% 0.0%
Mora et al., 2017 9.6 w gest 7.7 y 420 0.00 [-0.00; 0.00] 35.5% 35.5%
Jensen et al., 2020 before 16 w gest 3m, 18 m 274 0.14 [-0.07; 0.35] 0.0% 0.0%
Chen et al., 2019 del 5 y 196 0.00 [-0.00; 0.00] 53.6% 53.6%
Manzano-Salgado et al., 2017 1st tr gest 7 y 535 -0.04 [-0.19; 0.11] 0.0% 0.0%
Common effect model 0.00 [-0.00; 0.00] 100.0% --
Random effects model 0.00 [-0.00; 0.00] -- 100.0%
Heterogeneity: I^2 = 0%, τ^2 = 0, p = 0.85
-0.3 -0.2 -0.1 0 0.1 0.2 0.3

Study	Sampling	Outcome	Subjects	Estimate [95% CI]
Zhang, Lei, et al., 2022	within 3 d before del	7 y	98	0.000 [-0.004; 0.004]
Bloom et al., 2022	8-13 w gest	4-8 y	319	0.046 [-0.087; 0.179]
Hartman et al., 2017	15 w gest	9 y	319	-0.001 [-0.014; 0.013]
Liu et al., 2020	del	12 y	116	-0.010 [-0.290; 0.269]
Mora et al., 2017	9.6 w gest	7.7 y	420	0.002 [0.000; 0.004]
Jensen et al., 2020	before 16 w gest	3m, 18 m	274	0.140 [-0.075; 0.355]
Chen et al., 2019	del	5 y	196	0.001 [-0.001; 0.002]
Manzano-Salgado et al., 2017	1st tr gest	7 y	535	-0.040 [-0.186; 0.106]
**Summary**				**0.014 [-0.035; 0.063]**

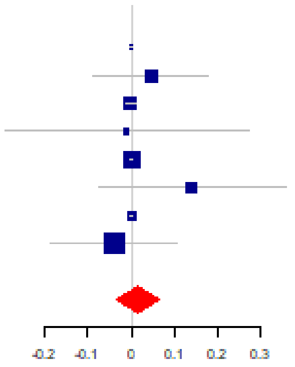

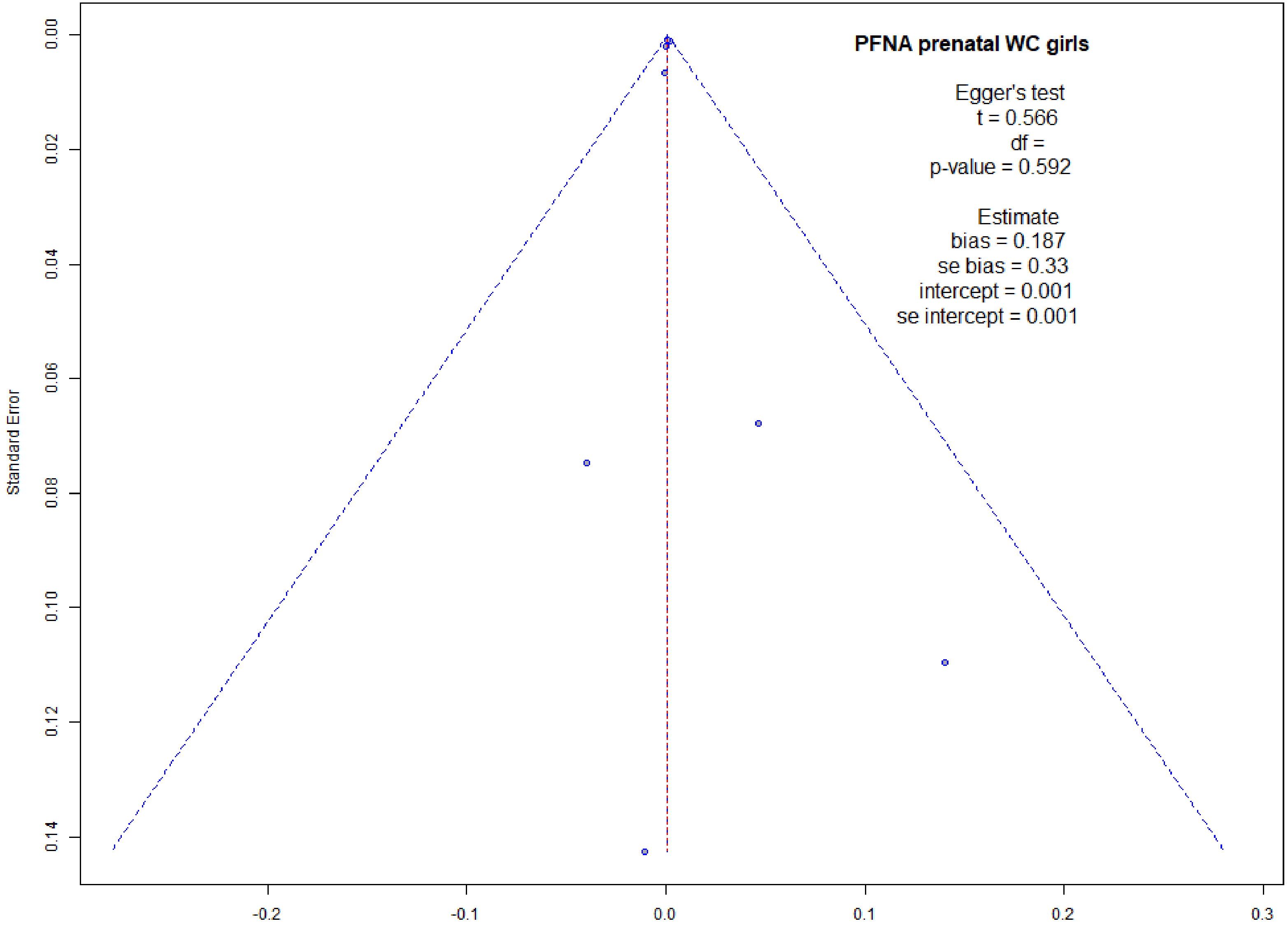
PFNA prenatal WC girls
Egger's test
t = 0.566
df =
p-value = 0.592
Estimate
bias = 0.187
se bias = 0.33
intercept = 0.001
se intercept = 0.001
Standard Error
0.00
0.02
0.04
0.06
0.08
0.10
0.12
0.14
-0.2
-0.1
0.0
0.1
0.2
0.3

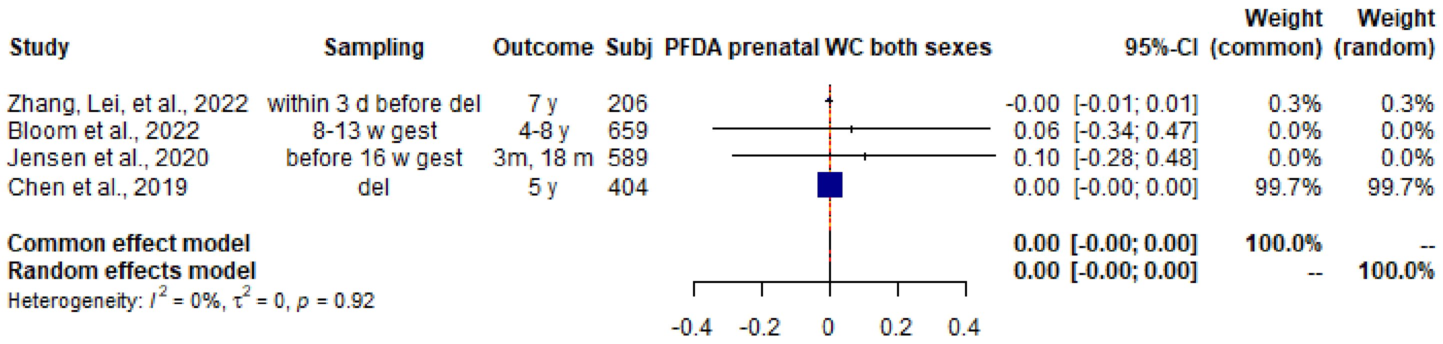
Study
Sampling
Outcome
Subj
PFDA prenatal WC both sexes
95%-CI
Weight (common)
Weight (random)
Zhang, Lei, et al., 2022 within 3 d before del 7 y 206 -0.00 [-0.01; 0.01] 0.3% 0.3%
Bloom et al., 2022 8-13 w gest 4-8 y 659 0.06 [-0.34; 0.47] 0.0% 0.0%
Jensen et al., 2020 before 16 w gest 3m, 18 m 589 0.10 [-0.28; 0.48] 0.0% 0.0%
Chen et al., 2019 del 5 y 404 0.00 [-0.00; 0.00] 99.7% 99.7%
Common effect model 0.00 [-0.00; 0.00] 100.0% --
Random effects model 0.00 [-0.00; 0.00] -- 100.0%
Heterogeneity: I² = 0%, τ² = 0, p = 0.92
-0.4
-0.2
0
0.2
0.4

Study	Sampling	Outcome	Subjects	Estimate [95% CI]
Zhang, Lei, et al., 2022	within 3 d before del	7 y	206	-0.001 [-0.011; 0.008]
Bloom et al., 2022	8-13 w gest	4-8 y	659	0.063 [-0.345; 0.470]
Jensen et al., 2020	before 16 w gest	3m, 18 m	589	0.100 [-0.285; 0.485]
Chen et al., 2019	del	5 y	404	0.000 [0.000; 0.001]
**Summary**				**0.054 [-0.135; 0.243]**

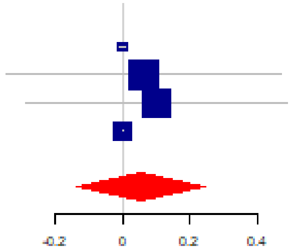

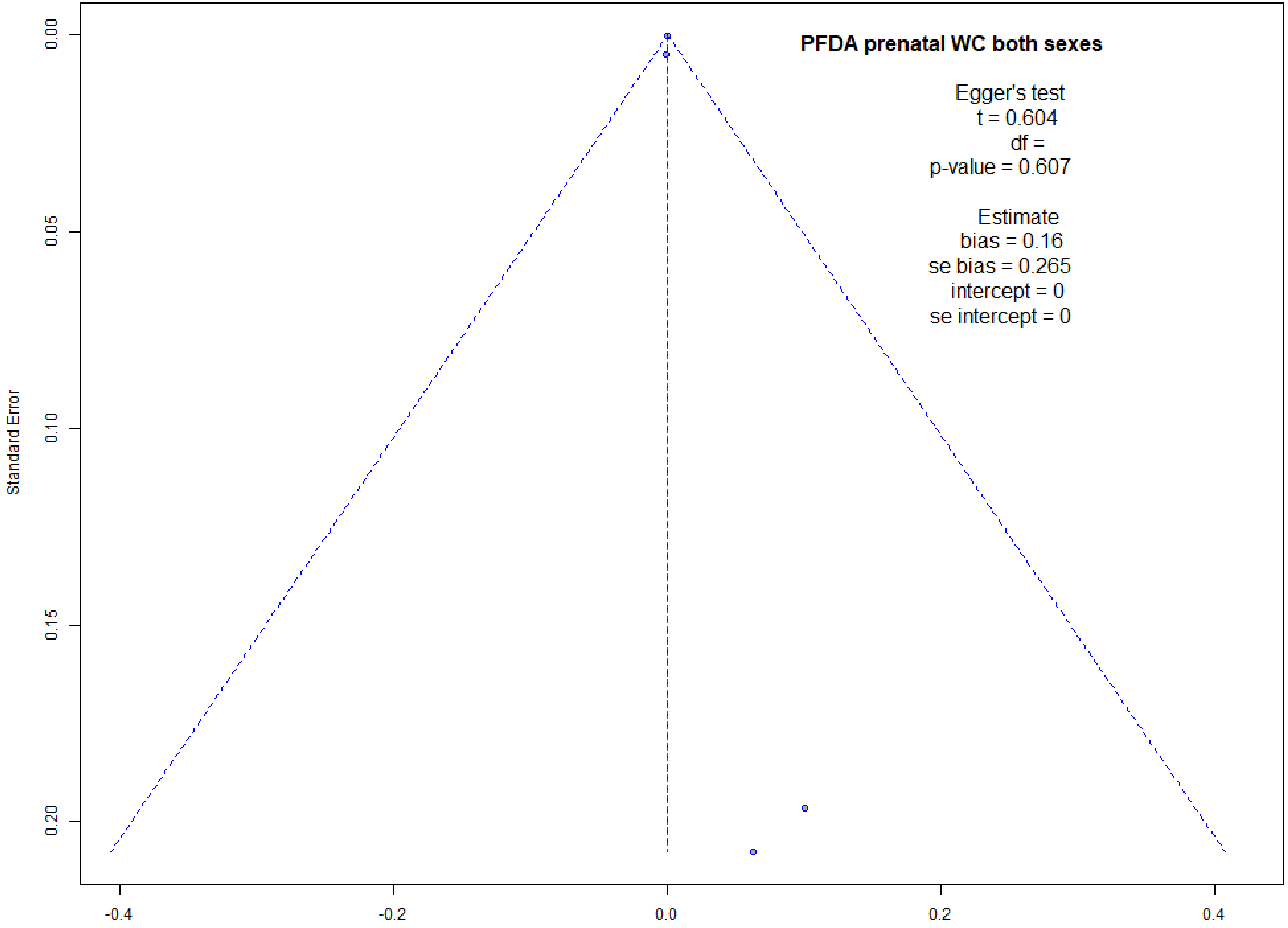
PFDA prenatal WC both sexes
Egger's test
t = 0.604
df =
p-value = 0.607
Estimate
bias = 0.16
se bias = 0.265
intercept = 0
se intercept = 0
Standard Error
0.00
0.05
0.10
0.15
0.20
-0.4
-0.2
0.0
0.2
0.4

Study	Sampling	Outcome	Subj	PFDA prenatal WC boys	95%-CI	Weight (common)	Weight (random)
Zhang, Lei, et al., 2022	within 3 d before del	7 y	108	-0.01	[-0.04; 0.02]	0.0%	0.0%
Bloom et al., 2022	8-13 w gest	4-8 y	340	0.06	[-0.60; 0.72]	0.0%	0.0%
Jensen et al., 2020	before 16 w gest	3m, 18 m	315	0.13	[-0.38; 0.64]	0.0%	0.0%
Chen et al., 2019	del	5 y	208	-0.00	[-0.00; 0.00]	100.0%	100.0%
**Common effect model**				**-0.00**	**[-0.00; 0.00]**	**100.0%**	--
**Random effects model**				**-0.00**	**[-0.00; 0.00]**	--	**100.0%**

Heterogeneity: $I^2 = 0\%$, $\tau^2 = 0$, $p = 0.91$

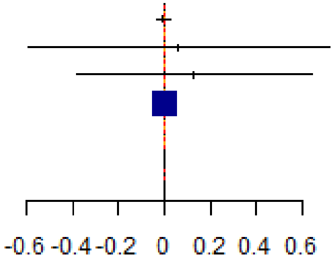

Study	Sampling	Outcome	Subjects	Estimate [95% CI]
Zhang, Lei, et al., 2022	within 3 d before del	7 y	108	-0.008 [-0.038; 0.023]
Bloom et al., 2022	8-13 w gest	4-8 y	340	0.063 [-0.595; 0.720]
Jensen et al., 2020	before 16 w gest	3m, 18 m	315	0.130 [-0.380; 0.640]
Chen et al., 2019	del	5 y	208	0.000 [0.000; 0.000]
**Summary**				**0.063 [-0.220; 0.347]**

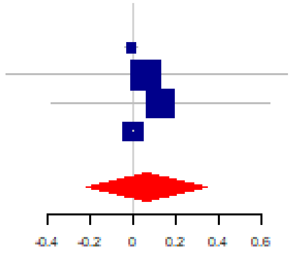

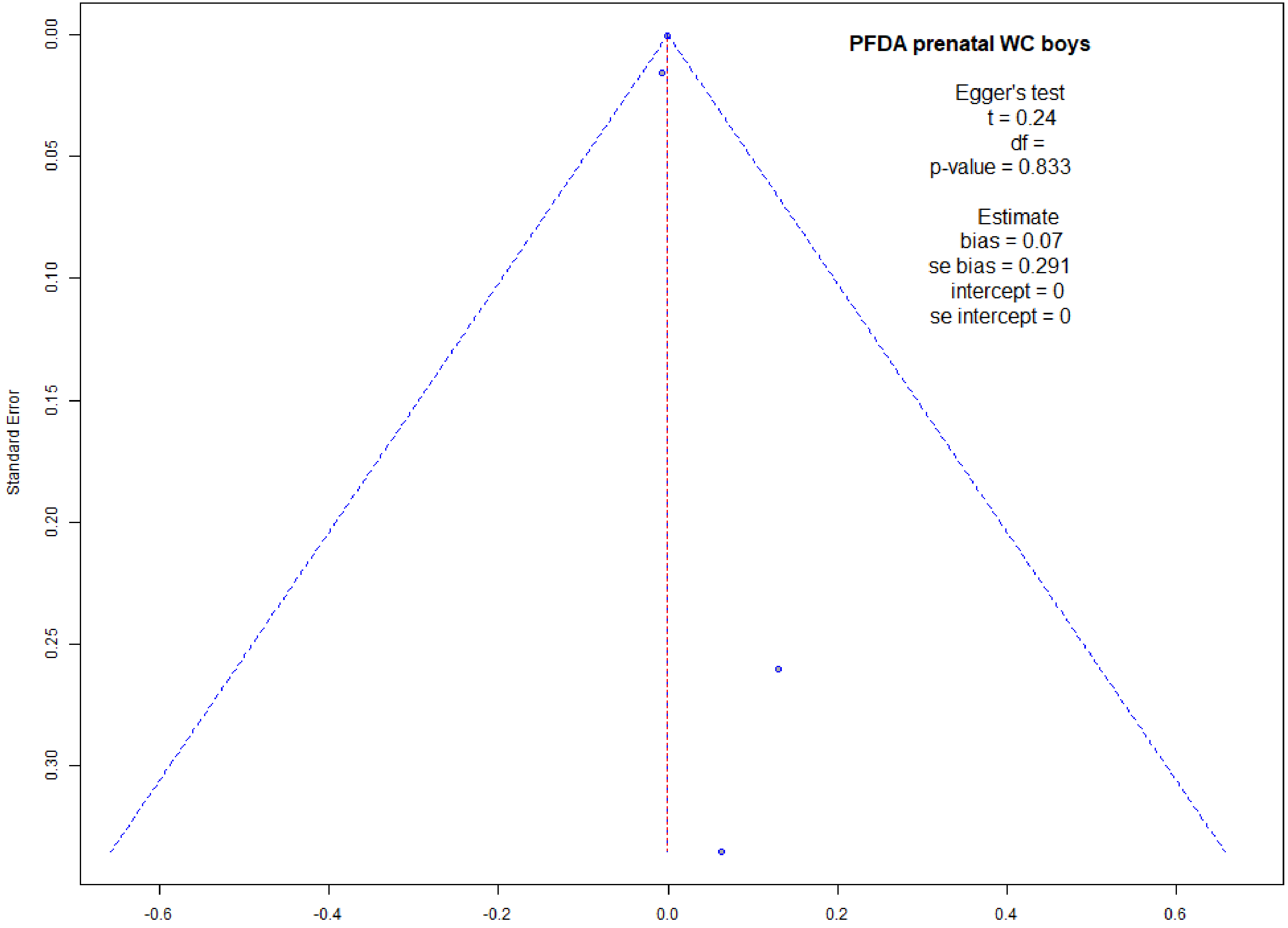
PFDA prenatal WC boys
Egger's test
t = 0.24
df =
p-value = 0.833
Estimate
bias = 0.07
se bias = 0.291
intercept = 0
se intercept = 0
Standard Error
0.00
0.05
0.10
0.15
0.20
0.25
0.30
-0.6
-0.4
-0.2
0.0
0.2
0.4
0.6

Prenatal and childhood exposure to per-/polyfluoroalkyl substances (PFASs) and its associations with childhood overweight and/or obesity: a systematic review with meta-analyses

Gianfranco Frigerio, Chiara Matilde Ferrari, and Silvia Fustinoni

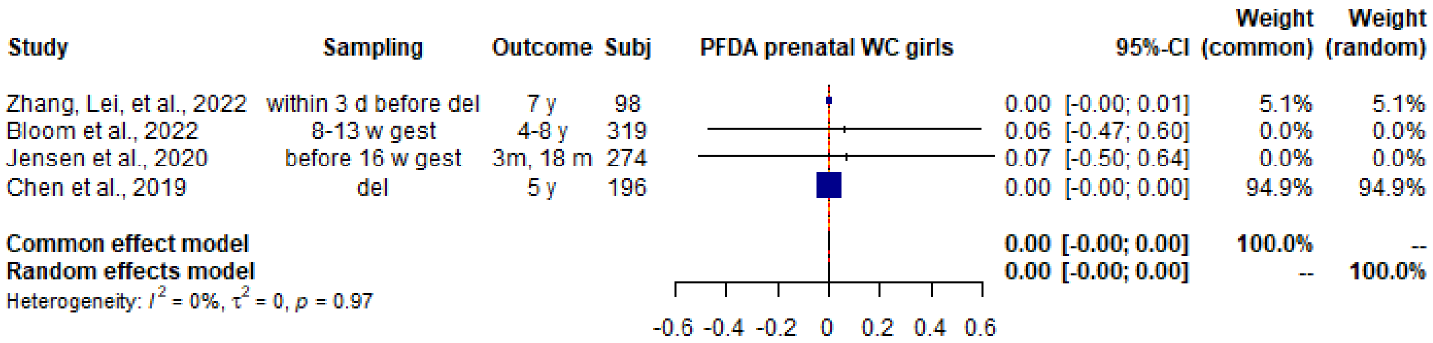

Study	Sampling	Outcome	Subjects	Estimate [95% CI]
Zhang, Lei, et al., 2022	within 3 d before del	7 y	98	0.000 [-0.005; 0.005]
Bloom et al., 2022	8-13 w gest	4-8 y	319	0.063 [-0.470; 0.595]
Jensen et al., 2020	before 16 w gest	3m, 18 m	274	0.070 [-0.505; 0.645]
Chen et al., 2019	del	5 y	196	0.001 [0.000; 0.002]
**Summary**				**0.044 [-0.217; 0.306]**

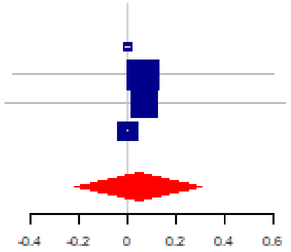

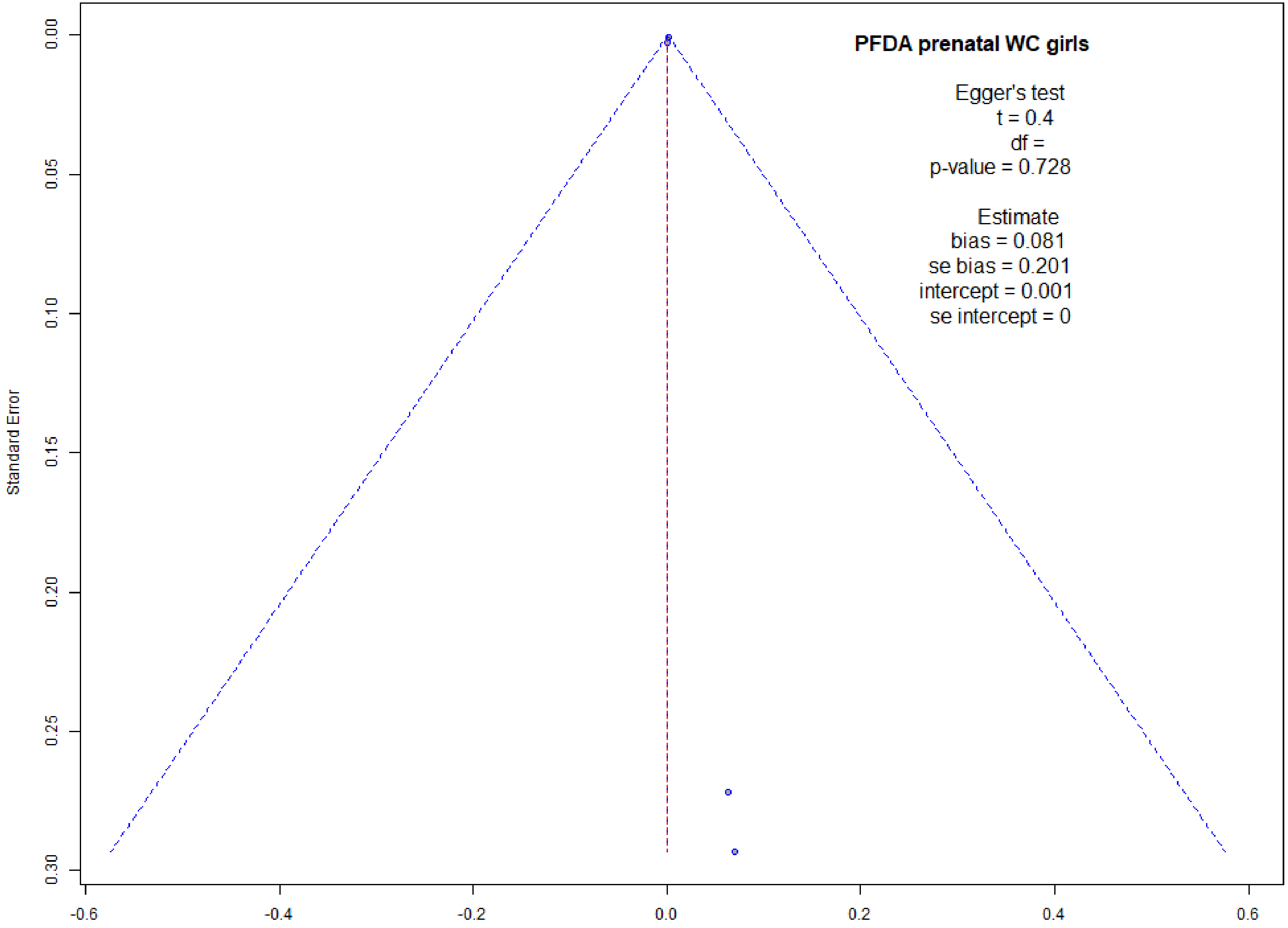
PFDA prenatal WC girls
Egger's test
t = 0.4
df =
p-value = 0.728
Estimate
bias = 0.081
se bias = 0.201
intercept = 0.001
se intercept = 0
Standard Error
0.00
0.05
0.10
0.15
0.20
0.25
0.30
-0.6
-0.4
-0.2
0.0
0.2
0.4
0.6

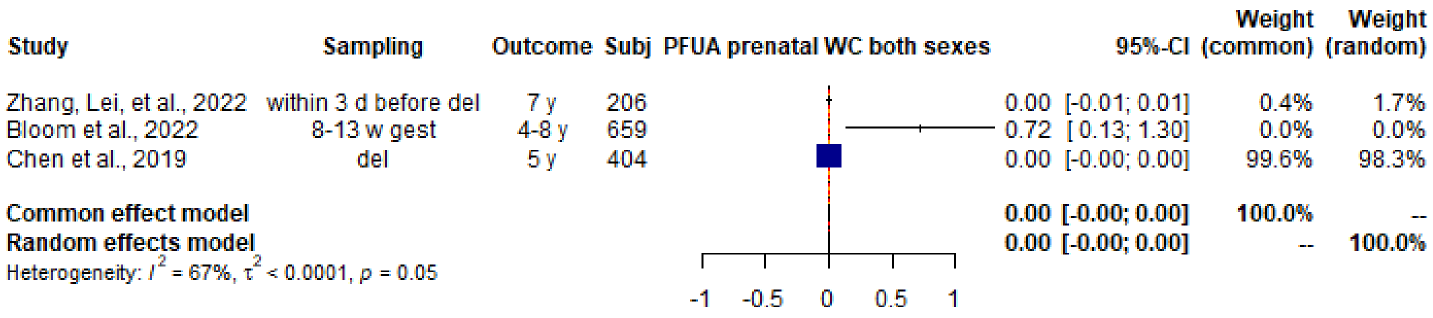
Study | Sampling | Outcome | Subj | PFUA prenatal WC both sexes | 95%-CI | Weight (common) | Weight (random)
Zhang, Lei, et al., 2022 | within 3 d before del | 7 y | 206 | 0.00 [-0.01; 0.01] | 0.4% | 1.7%
Bloom et al., 2022 | 8-13 w gest | 4-8 y | 659 | 0.72 [0.13; 1.30] | 0.0% | 0.0%
Chen et al., 2019 | del | 5 y | 404 | 0.00 [-0.00; 0.00] | 99.6% | 98.3%
Common effect model | 0.00 [-0.00; 0.00] | 100.0% | --
Random effects model | 0.00 [-0.00; 0.00] | -- | 100.0%
Heterogeneity: I^2 = 67%, τ^2 < 0.0001, p = 0.05
-1 -0.5 0 0.5 1

Study	Sampling	Outcome	Subjects	Estimate [95% CI]
Zhang, Lei, et al., 2022	within 3 d before del	7 y	206	0.003 [-0.008; 0.015]
Bloom et al., 2022	8-13 w gest	4-8 y	659	0.717 [0.134; 1.299]
Chen et al., 2019	del	5 y	404	0.000 [0.000; 0.001]
**Summary**				**0.373 [0.070; 0.675]**

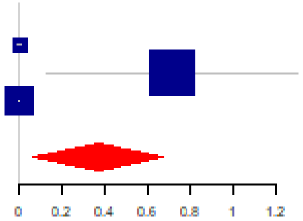

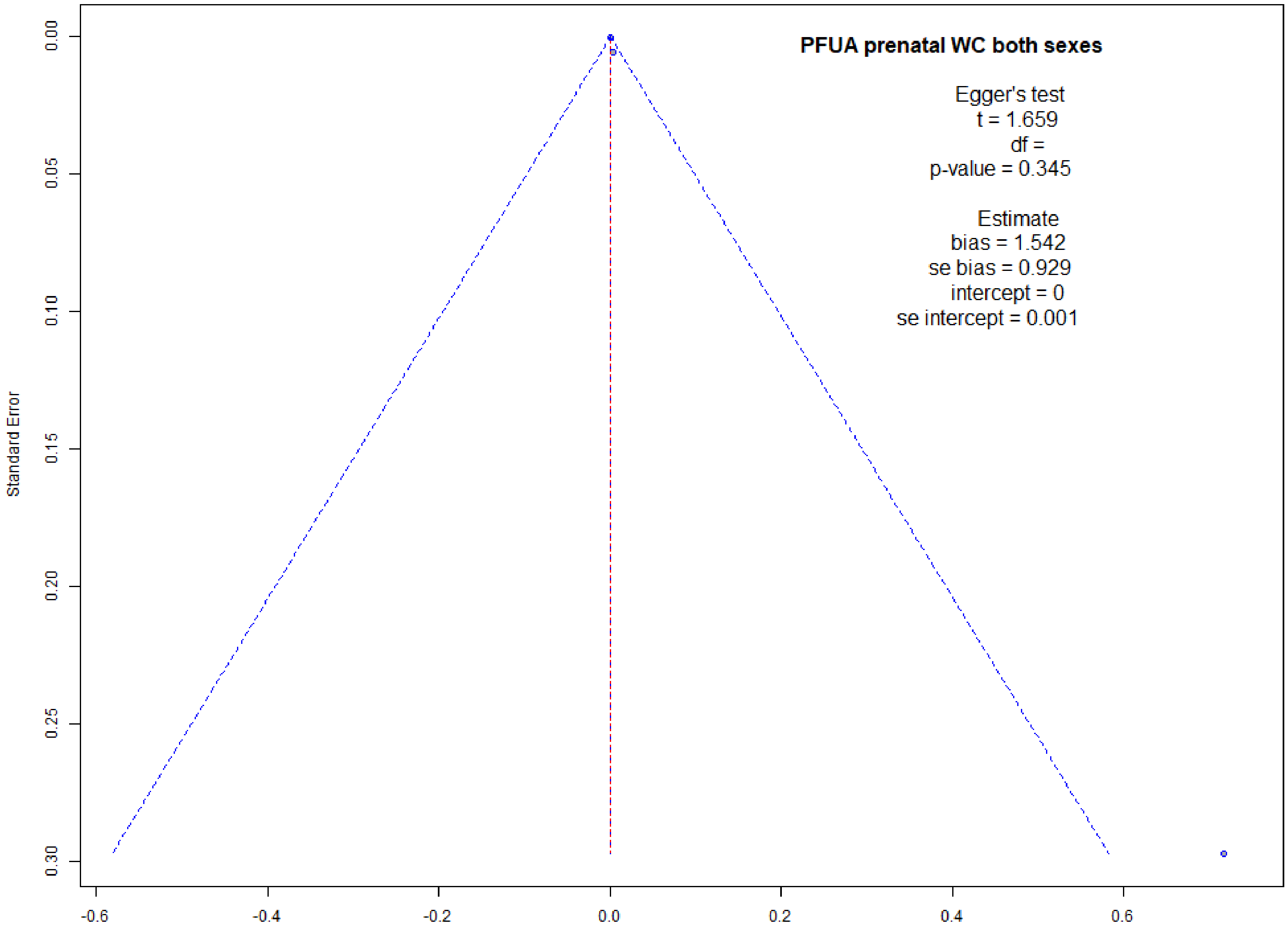
PFUA prenatal WC both sexes
Egger's test
t = 1.659
df =
p-value = 0.345
Estimate
bias = 1.542
se bias = 0.929
intercept = 0
se intercept = 0.001
Standard Error
0.00
0.05
0.10
0.15
0.20
0.25
0.30
-0.6
-0.4
-0.2
0.0
0.2
0.4
0.6

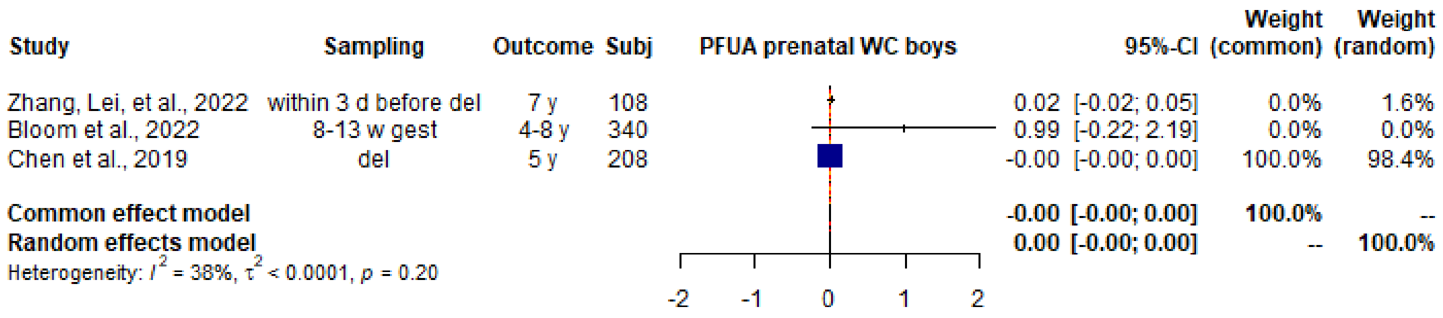
Study
Sampling
Outcome
Subj
PFUA prenatal WC boys
95%-CI
Weight (common)
Weight (random)
Zhang, Lei, et al., 2022 within 3 d before del 7 y 108 0.02 [-0.02; 0.05] 0.0% 1.6%
Bloom et al., 2022 8-13 w gest 4-8 y 340 0.99 [-0.22; 2.19] 0.0% 0.0%
Chen et al., 2019 del 5 y 208 -0.00 [-0.00; 0.00] 100.0% 98.4%
Common effect model -0.00 [-0.00; 0.00] 100.0% --
Random effects model 0.00 [-0.00; 0.00] -- 100.0%
Heterogeneity: I^2 = 38%, τ^2 < 0.0001, p = 0.20
-2
-1
0
1
2

Study	Sampling	Outcome	Subjects	Estimate [95% CI]
Zhang, Lei, et al., 2022	within 3 d before del	7 y	108	0.015 [-0.021; 0.051]
Bloom et al., 2022	8-13 w gest	4-8 y	340	0.985 [-0.224; 2.195]
Chen et al., 2019	del	5 y	208	0.000 [-0.001; 0.000]
**Summary**				**0.513 [-0.114; 1.140]**

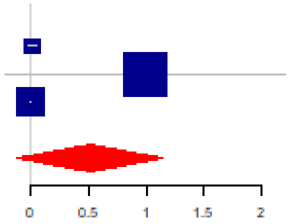

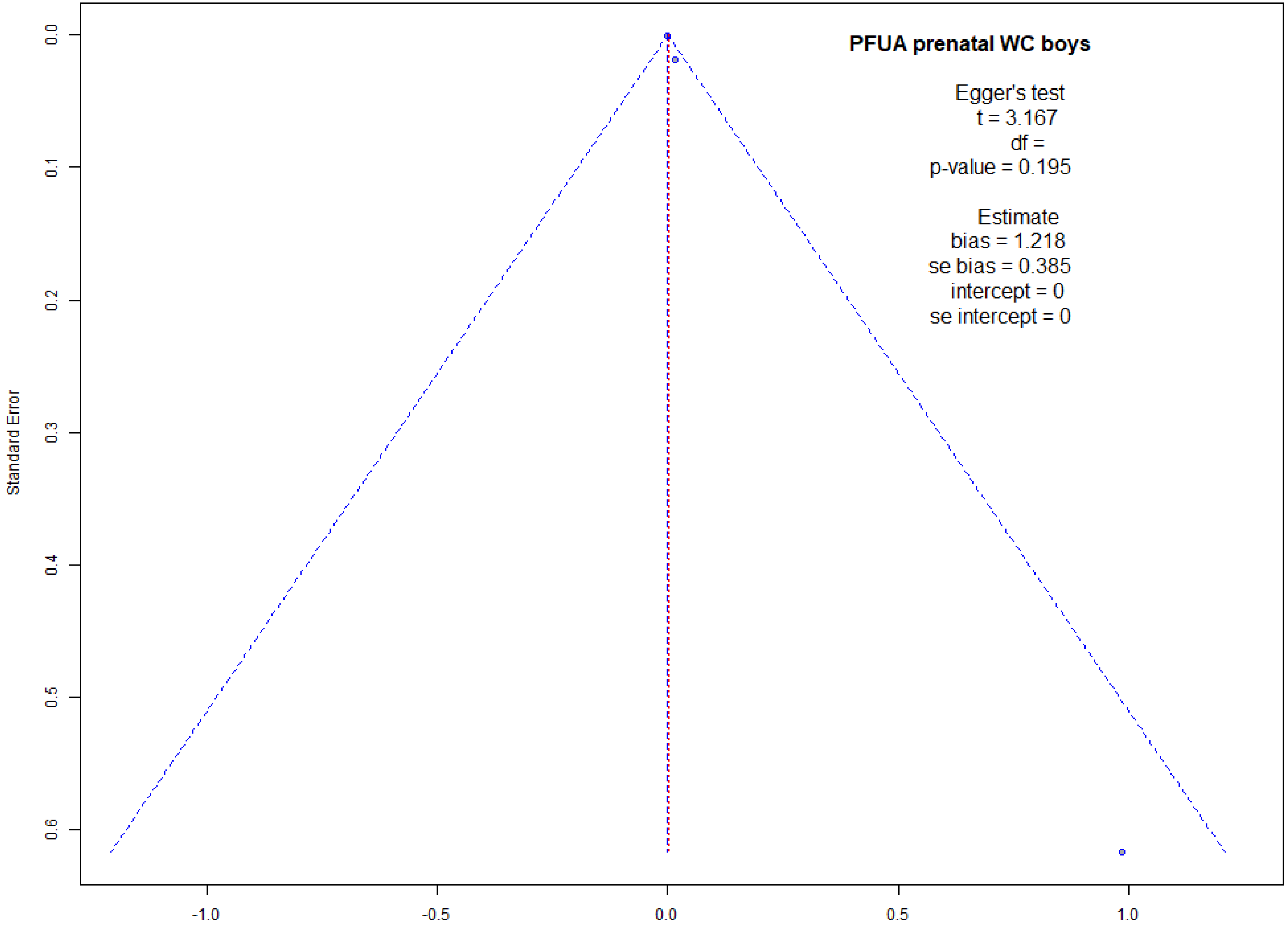
PFUA prenatal WC boys
Egger's test
t = 3.167
df =
p-value = 0.195
Estimate
bias = 1.218
se bias = 0.385
intercept = 0
se intercept = 0
Standard Error
0.0
0.1
0.2
0.3
0.4
0.5
0.6
-1.0
-0.5
0.0
0.5
1.0

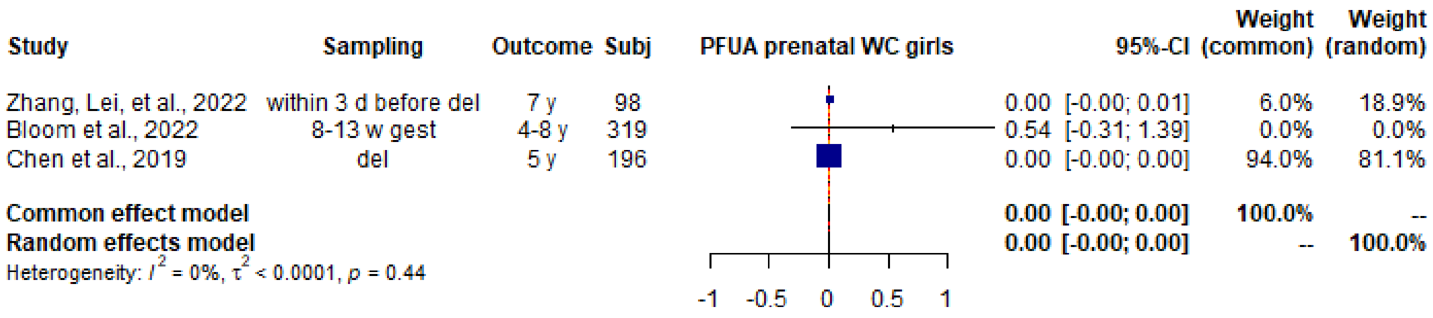
Study
Sampling
Outcome
Subj
PFUA prenatal WC girls
95%-CI
Weight (common)
Weight (random)
Zhang, Lei, et al., 2022 within 3 d before del 7 y 98 0.00 [-0.00; 0.01] 6.0% 18.9%
Bloom et al., 2022 8-13 w gest 4-8 y 319 0.54 [-0.31; 1.39] 0.0% 0.0%
Chen et al., 2019 del 5 y 196 0.00 [-0.00; 0.00] 94.0% 81.1%
Common effect model 0.00 [-0.00; 0.00] 100.0% --
Random effects model 0.00 [-0.00; 0.00] -- 100.0%
Heterogeneity: I^2 = 0%, τ^2 < 0.0001, p = 0.44
-1
-0.5
0
0.5
1

Study	Sampling	Outcome	Subjects	Estimate [95% CI]
Zhang, Lei, et al., 2022	within 3 d before del	7 y	98	0.002 [-0.004; 0.009]
Bloom et al., 2022	8-13 w gest	4-8 y	319	0.538 [-0.314; 1.389]
Chen et al., 2019	del	5 y	196	0.001 [0.000; 0.003]
**Summary**				**0.281 [-0.162; 0.723]**

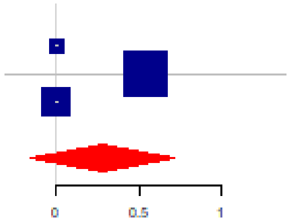

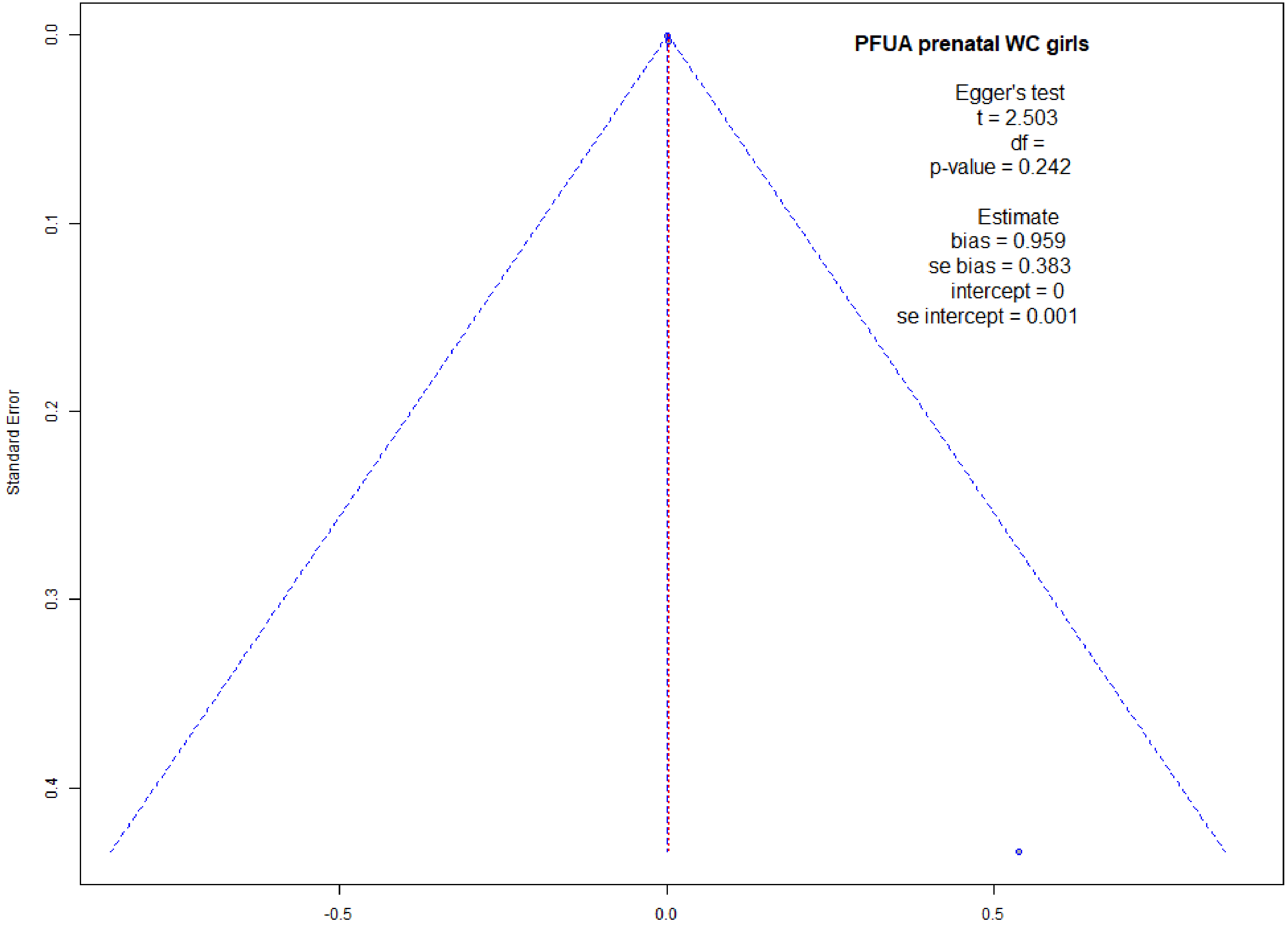
PFUA prenatal WC girls
Egger's test
t = 2.503
df =
p-value = 0.242
Estimate
bias = 0.959
se bias = 0.383
intercept = 0
se intercept = 0.001
Standard Error
0.0
0.1
0.2
0.3
0.4
-0.5
0.0
0.5

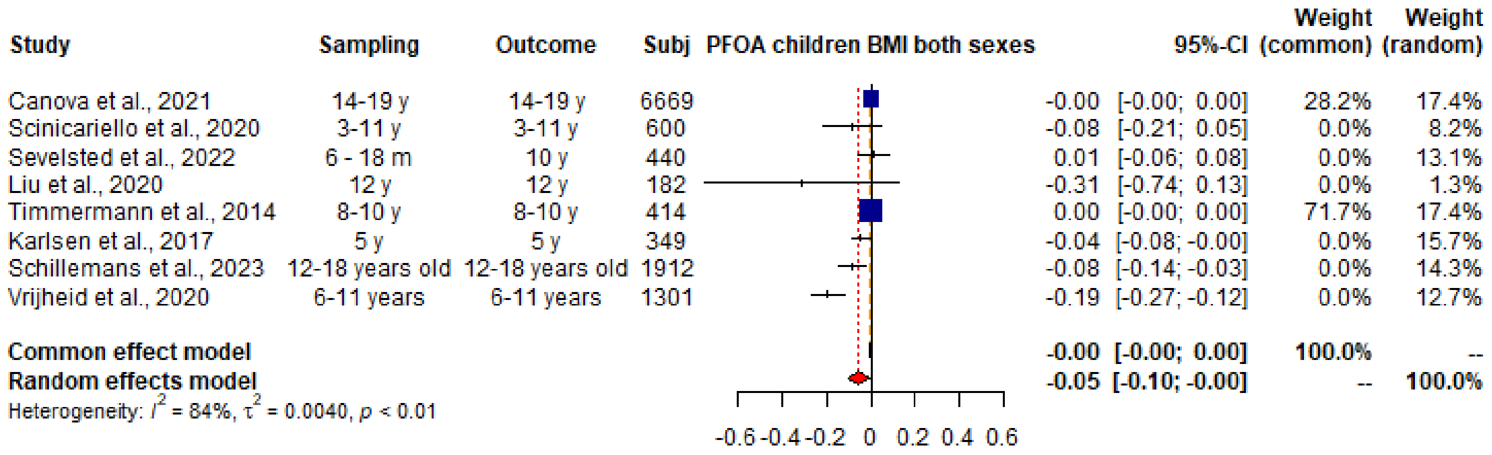
Study
Sampling
Outcome
Subj
PFOA children BMI both sexes
95%-CI
Weight (common)
Weight (random)
Canova et al., 2021 | 14-19 y | 14-19 y | 6669 | -0.00 [-0.00; 0.00] | 28.2% | 17.4%
Scinicariello et al., 2020 | 3-11 y | 3-11 y | 600 | -0.08 [-0.21; 0.05] | 0.0% | 8.2%
Sevelsted et al., 2022 | 6 - 18 m | 10 y | 440 | 0.01 [-0.06; 0.08] | 0.0% | 13.1%
Liu et al., 2020 | 12 y | 12 y | 182 | -0.31 [-0.74; 0.13] | 0.0% | 1.3%
Timmermann et al., 2014 | 8-10 y | 8-10 y | 414 | 0.00 [-0.00; 0.00] | 71.7% | 17.4%
Karlsen et al., 2017 | 5 y | 5 y | 349 | -0.04 [-0.08; -0.00] | 0.0% | 15.7%
Schillemans et al., 2023 | 12-18 years old | 12-18 years old | 1912 | -0.08 [-0.14; -0.03] | 0.0% | 14.3%
Vrijheid et al., 2020 | 6-11 years | 6-11 years | 1301 | -0.19 [-0.27; -0.12] | 0.0% | 12.7%
Common effect model | -0.00 [-0.00; 0.00] | 100.0% | --
Random effects model | -0.05 [-0.10; -0.00] | -- | 100.0%
Heterogeneity: I^2 = 84%, τ^2 = 0.0040, p < 0.01
-0.6 -0.4 -0.2 0 0.2 0.4 0.6

Study	Sampling	Outcome	Subjects	Estimate [95% CI]
Canova et al., 2021	14-19 y	14-19 y	6669	-0.001 [-0.001; 0.000]
Scinicariello et al., 2020	3-11 y	3-11 y	600	-0.080 [-0.210; 0.050]
Sevelsted et al., 2022	6 - 18 m	10 y	440	0.010 [-0.060; 0.080]
Liu et al., 2020	12 y	12 y	182	-0.306 [-0.741; 0.129]
Timmermann et al., 2014	8-10 y	8-10 y	414	0.000 [0.000; 0.001]
Karlsen et al., 2017	5 y	5 y	349	-0.044 [-0.085; -0.003]
Schillemans et al., 2023	12-18 years old	12-18 years old	1912	-0.083 [-0.141; -0.026]
Vrijheid et al., 2020	6-11 years	6-11 years	1301	-0.193 [-0.267; -0.118]
**Summary**				**-0.045 [-0.060; -0.029]**

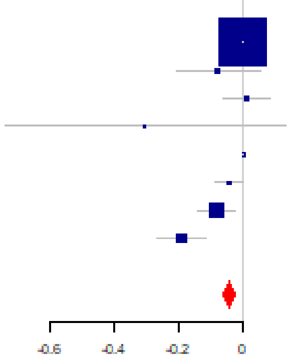

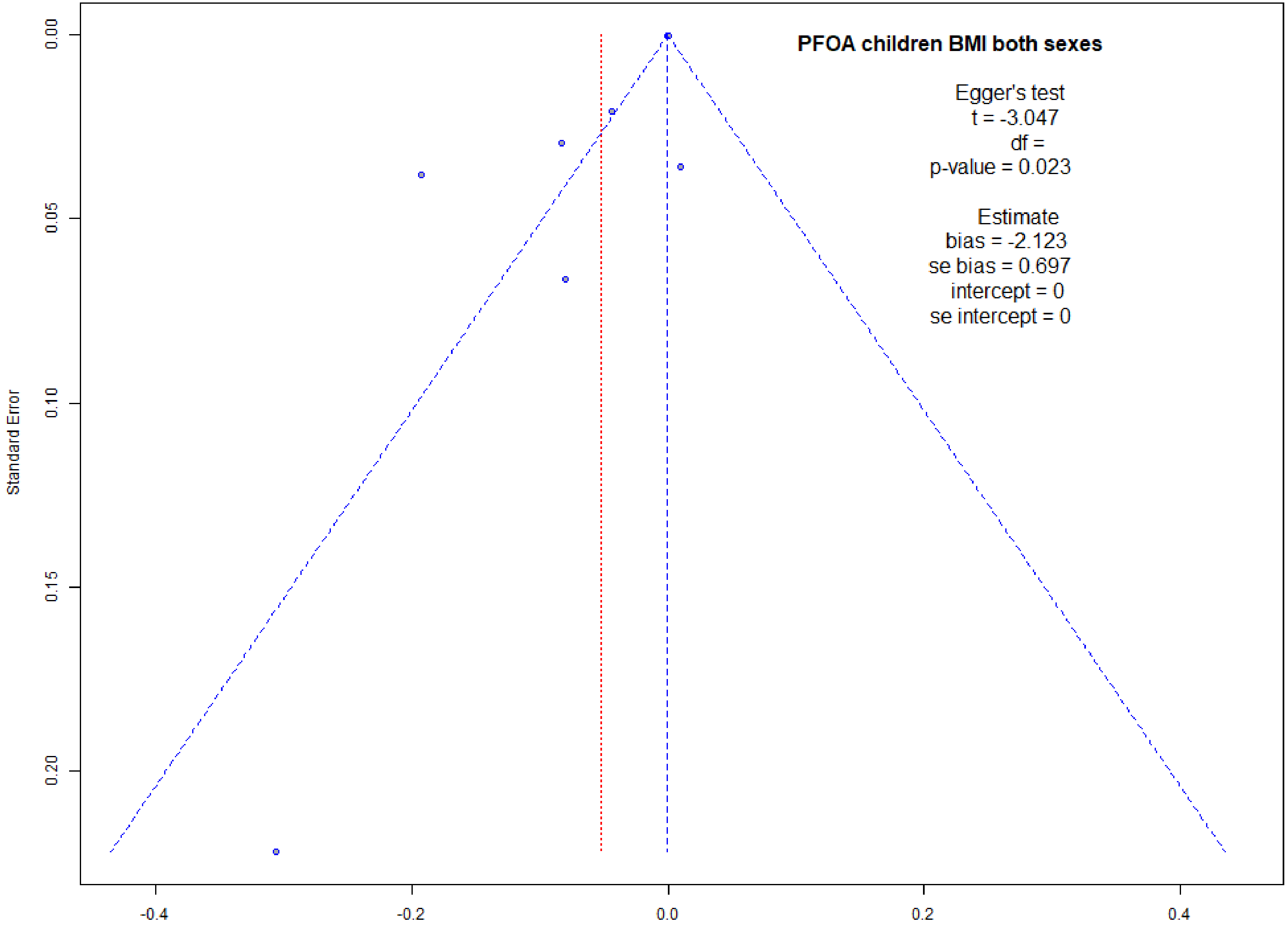
PFOA children BMI both sexes
Egger's test
t = -3.047
df =
p-value = 0.023
Estimate
bias = -2.123
se bias = 0.697
intercept = 0
se intercept = 0
Standard Error
0.00
0.05
0.10
0.15
0.20
-0.4
-0.2
0.0
0.2
0.4

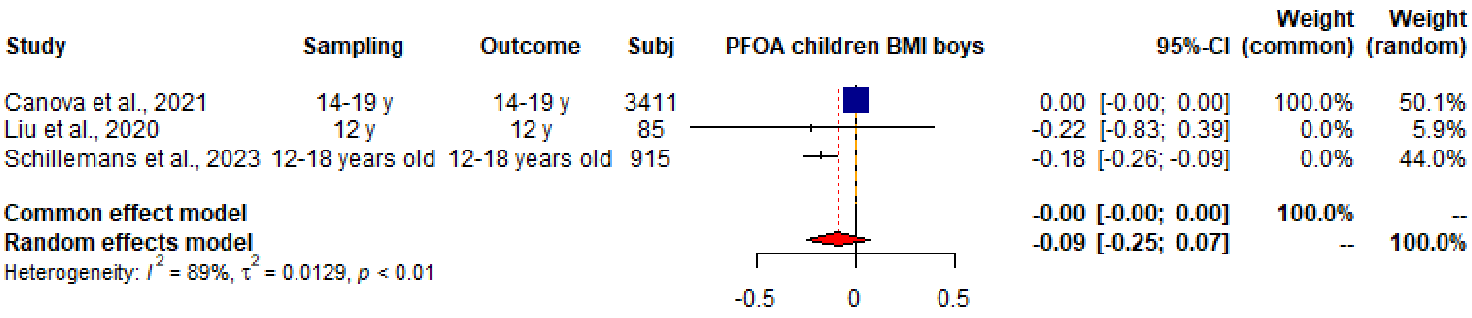
Study | Sampling | Outcome | Subj | PFOA children BMI boys | 95%-CI | Weight (common) | Weight (random)
Canova et al., 2021 | 14-19 y | 14-19 y | 3411 | 0.00 [-0.00; 0.00] | 100.0% | 50.1%
Liu et al., 2020 | 12 y | 12 y | 85 | -0.22 [-0.83; 0.39] | 0.0% | 5.9%
Schillemans et al., 2023 | 12-18 years old | 12-18 years old | 915 | -0.18 [-0.26; -0.09] | 0.0% | 44.0%
Common effect model | -0.00 [-0.00; 0.00] | 100.0% | --
Random effects model | -0.09 [-0.25; 0.07] | -- | 100.0%
Heterogeneity: I^2 = 89%, τ^2 = 0.0129, p < 0.01
-0.5
0
0.5

Study	Sampling	Outcome	Subjects	Estimate [95% CI]
Canova et al., 2021	14-19 y	14-19 y	3411	0.000 [-0.001; 0.001]
Liu et al., 2020	12 y	12 y	85	-0.222 [-0.833; 0.389]
Schillemans et al., 2023	12-18 years old	12-18 years old	915	-0.177 [-0.261; -0.094]
**Summary**				**-0.041 [-0.228; 0.146]**

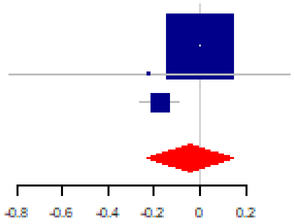

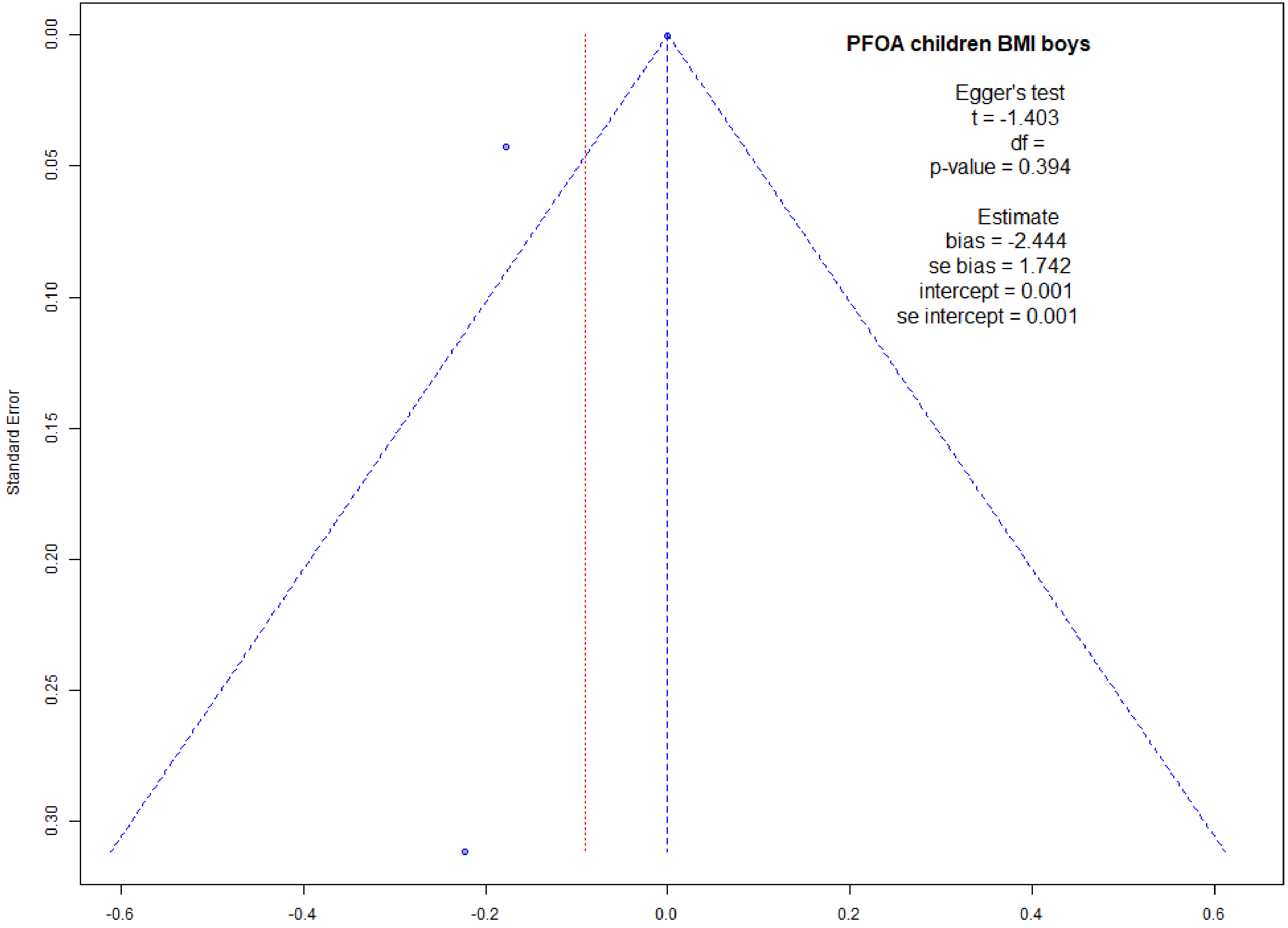
PFOA children BMI boys
Egger's test
t = -1.403
df =
p-value = 0.394
Estimate
bias = -2.444
se bias = 1.742
intercept = 0.001
se intercept = 0.001
Standard Error
0.00
0.05
0.10
0.15
0.20
0.25
0.30
-0.6
-0.4
-0.2
0.0
0.2
0.4
0.6

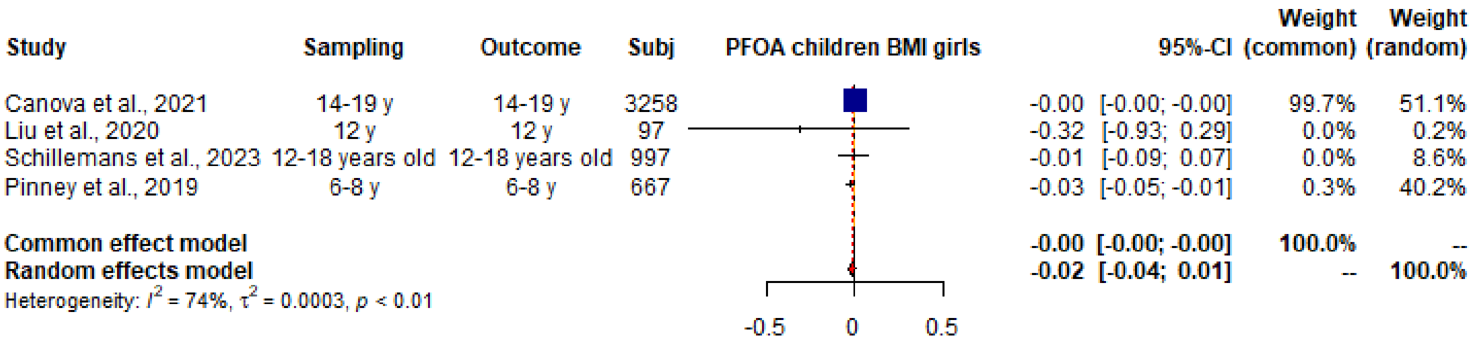
Study
Sampling
Outcome
Subj
PFOA children BMI girls
95%-CI
Weight (common)
Weight (random)
Canova et al., 2021 14-19 y 14-19 y 3258 -0.00 [-0.00; -0.00] 99.7% 51.1%
Liu et al., 2020 12 y 12 y 97 -0.32 [-0.93; 0.29] 0.0% 0.2%
Schillemans et al., 2023 12-18 years old 12-18 years old 997 -0.01 [-0.09; 0.07] 0.0% 8.6%
Pinney et al., 2019 6-8 y 6-8 y 667 -0.03 [-0.05; -0.01] 0.3% 40.2%
Common effect model -0.00 [-0.00; -0.00] 100.0% --
Random effects model -0.02 [-0.04; 0.01] -- 100.0%
Heterogeneity: I^2 = 74%, τ^2 = 0.0003, p < 0.01
-0.5
0
0.5

Study	Sampling	Outcome	Subjects	Estimate [95% CI]
Canova et al., 2021	14-19 y	14-19 y	3258	-0.002 [-0.003; -0.001]
Liu et al., 2020	12 y	12 y	97	-0.316 [-0.926; 0.295]
Schillemans et al., 2023	12-18 years old	12-18 years old	997	-0.010 [-0.089; 0.068]
Pinney et al., 2019	6-8 y	6-8 y	667	-0.032 [-0.050; -0.013]
**Summary**				**-0.013 [-0.046; 0.019]**

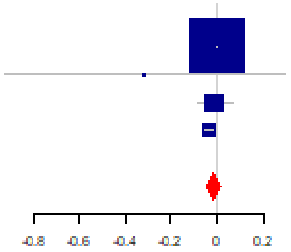

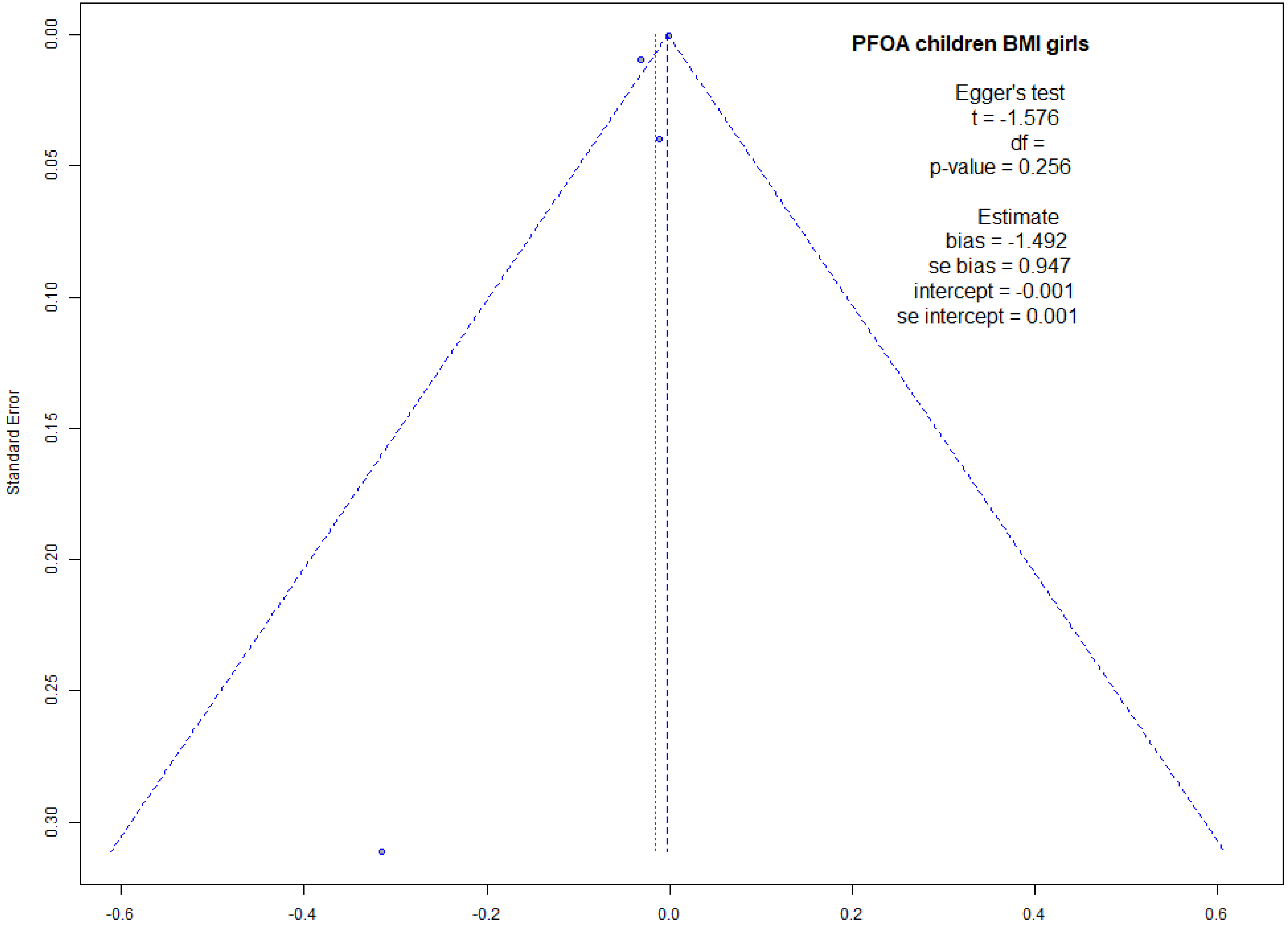
PFOA children BMI girls
Egger's test
t = -1.576
df =
p-value = 0.256
Estimate
bias = -1.492
se bias = 0.947
intercept = -0.001
se intercept = 0.001
Standard Error
0.00
0.05
0.10
0.15
0.20
0.25
0.30
-0.6
-0.4
-0.2
0.0
0.2
0.4
0.6

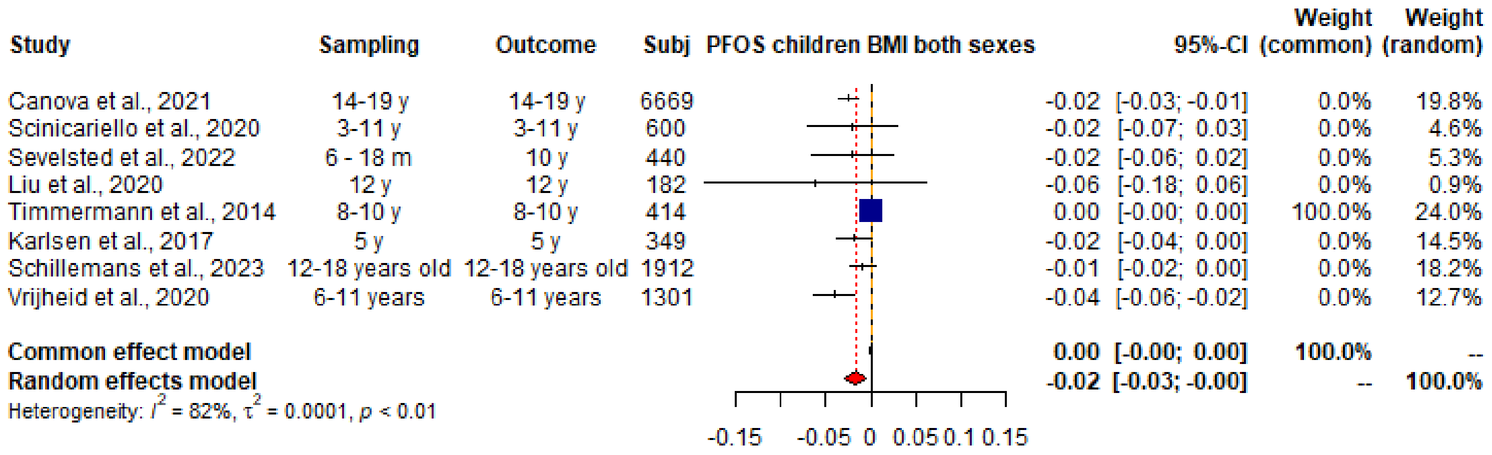
Study Sampling Outcome Subj PFOS children BMI both sexes 95%-CI Weight (common) Weight (random)
Canova et al., 2021 14-19 y 14-19 y 6669 -0.02 [-0.03; -0.01] 0.0% 19.8%
Scinicariello et al., 2020 3-11 y 3-11 y 600 -0.02 [-0.07; 0.03] 0.0% 4.6%
Sevelsted et al., 2022 6 - 18 m 10 y 440 -0.02 [-0.06; 0.02] 0.0% 5.3%
Liu et al., 2020 12 y 12 y 182 -0.06 [-0.18; 0.06] 0.0% 0.9%
Timmermann et al., 2014 8-10 y 8-10 y 414 0.00 [-0.00; 0.00] 100.0% 24.0%
Karlsen et al., 2017 5 y 5 y 349 -0.02 [-0.04; 0.00] 0.0% 14.5%
Schillemans et al., 2023 12-18 years old 12-18 years old 1912 -0.01 [-0.02; 0.00] 0.0% 18.2%
Vrijheid et al., 2020 6-11 years 6-11 years 1301 -0.04 [-0.06; -0.02] 0.0% 12.7%
Common effect model 0.00 [-0.00; 0.00] 100.0% --
Random effects model -0.02 [-0.03; -0.00] -- 100.0%
Heterogeneity: I^2 = 82%, τ^2 = 0.0001, p < 0.01
-0.15
-0.05
0
0.05
0.1
0.15

Study	Sampling	Outcome	Subjects	Estimate [95% CI]
Canova et al., 2021	14-19 y	14-19 y	6669	-0.024 [-0.035; -0.013]
Scinicariello et al., 2020	3-11 y	3-11 y	600	-0.021 [-0.070; 0.029]
Sevelsted et al., 2022	6 - 18 m	10 y	440	-0.020 [-0.065; 0.025]
Liu et al., 2020	12 y	12 y	182	-0.061 [-0.183; 0.061]
Timmermann et al., 2014	8-10 y	8-10 y	414	0.000 [0.000; 0.000]
Karlsen et al., 2017	5 y	5 y	349	-0.018 [-0.037; 0.002]
Schillemans et al., 2023	12-18 years old	12-18 years old	1912	-0.009 [-0.022; 0.004]
Vrijheid et al., 2020	6-11 years	6-11 years	1301	-0.040 [-0.063; -0.018]
**Summary**				**-0.023 [-0.041; -0.004]**

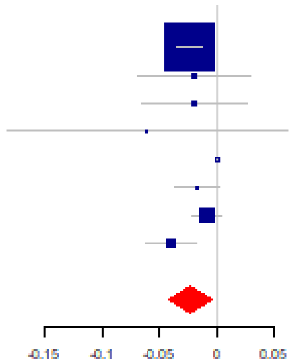

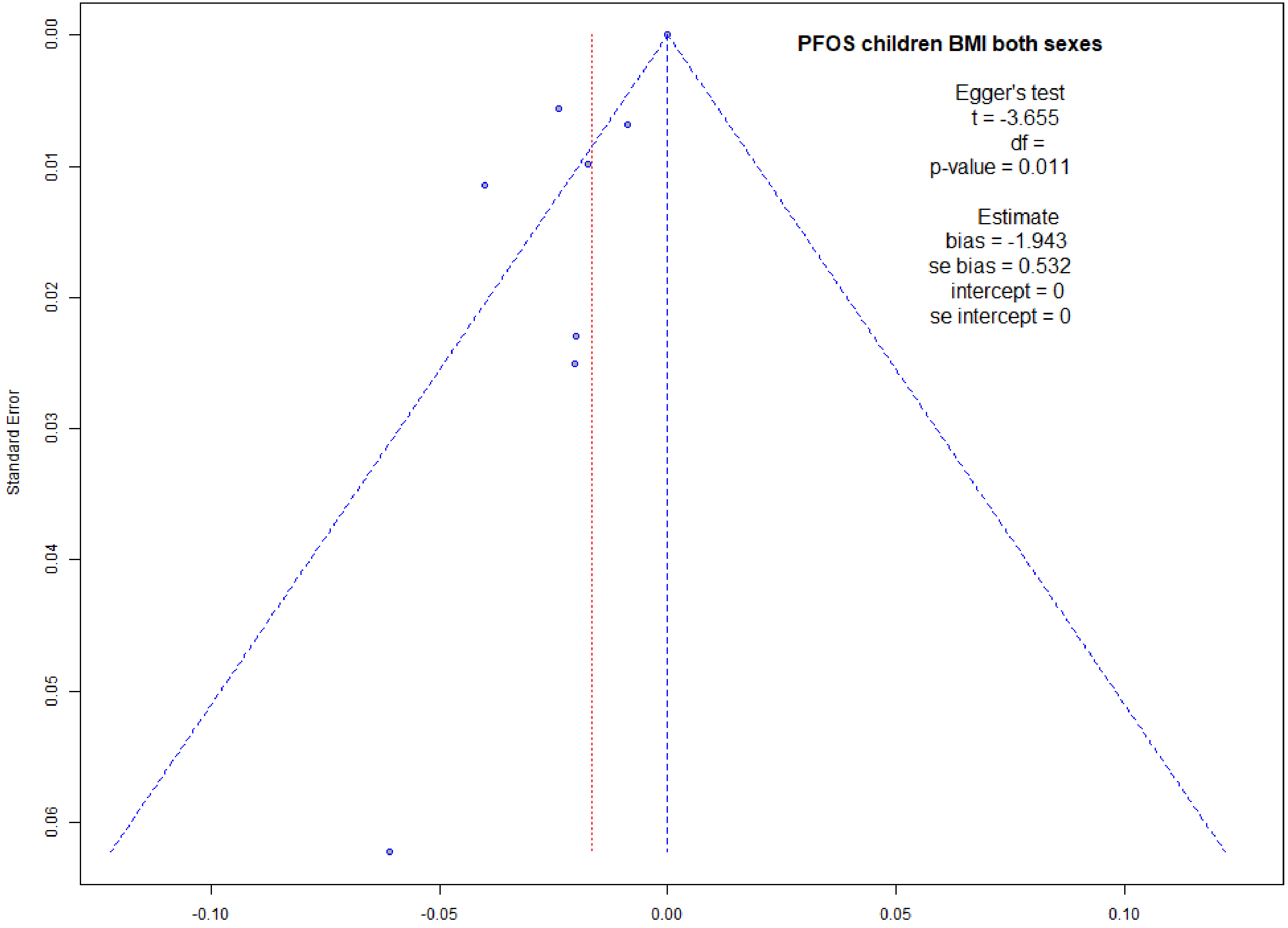
PFOS children BMI both sexes
Egger's test
t = -3.655
df =
p-value = 0.011
Estimate
bias = -1.943
se bias = 0.532
intercept = 0
se intercept = 0
Standard Error
0.00
0.01
0.02
0.03
0.04
0.05
0.06
-0.10
-0.05
0.00
0.05
0.10

Study	Sampling	Outcome	Subj	PFOS children BMI boys	95%-CI	Weight (common)	Weight (random)
Canova et al., 2021	14-19 y	14-19 y	3411	-0.02	[-0.03; -0.01]	70.3%	70.3%
Liu et al., 2020	12 y	12 y	85	-0.10	[-0.35; 0.15]	0.2%	0.2%
Schillemans et al., 2023	12-18 years old	12-18 years old	915	-0.03	[-0.05; -0.01]	29.5%	29.5%
**Common effect model**				**-0.02**	**[-0.03; -0.01]**	**100.0%**	**--**
**Random effects model**				**-0.02**	**[-0.03; -0.01]**	**--**	**100.0%**

Heterogeneity: $I^2 = 0\%$, $\tau^2 = 0$, $p = 0.63$

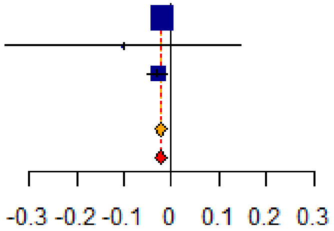

Study	Sampling	Outcome	Subjects	Estimate [95% CI]
Canova et al., 2021	14-19 y	14-19 y	3411	-0.020 [-0.033; -0.007]
Liu et al., 2020	12 y	12 y	85	-0.101 [-0.348; 0.146]
Schillemans et al., 2023	12-18 years old	12-18 years old	915	-0.029 [-0.049; -0.009]
**Summary**				**-0.024 [-0.035; -0.012]**

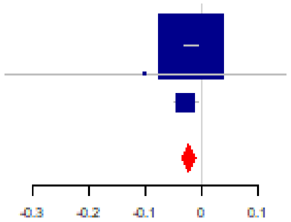

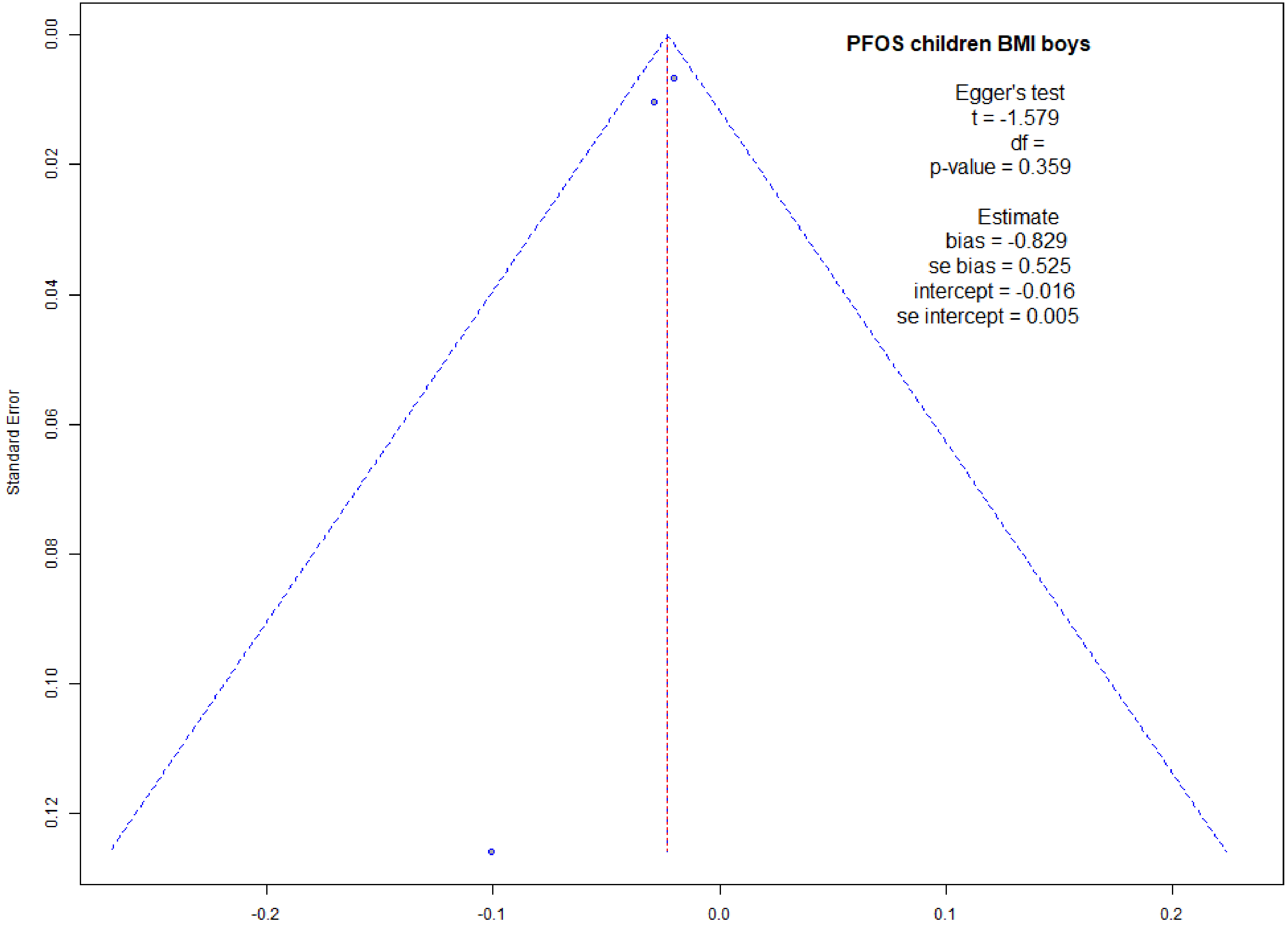
PFOS children BMI boys
Egger's test
t = -1.579
df =
p-value = 0.359
Estimate
bias = -0.829
se bias = 0.525
intercept = -0.016
se intercept = 0.005
Standard Error
0.00
0.02
0.04
0.06
0.08
0.10
0.12
-0.2
-0.1
0.0
0.1
0.2

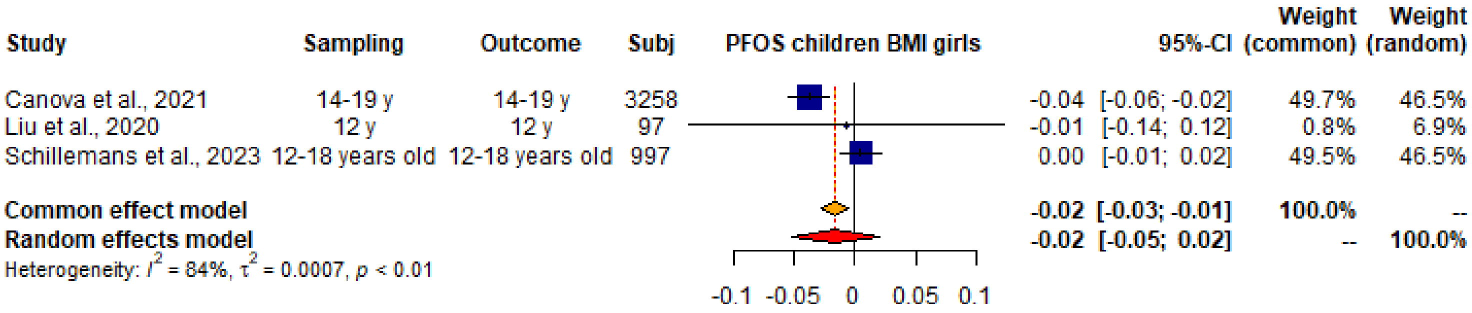
Study
Sampling
Outcome
Subj
PFOS children BMI girls
95%-CI
Weight (common)
Weight (random)
Canova et al., 2021 14-19 y 14-19 y 3258 -0.04 [-0.06; -0.02] 49.7% 46.5%
Liu et al., 2020 12 y 12 y 97 -0.01 [-0.14; 0.12] 0.8% 6.9%
Schillemans et al., 2023 12-18 years old 12-18 years old 997 0.00 [-0.01; 0.02] 49.5% 46.5%
Common effect model -0.02 [-0.03; -0.01] 100.0% --
Random effects model -0.02 [-0.05; 0.02] -- 100.0%
Heterogeneity: I^2 = 84%, τ^2 = 0.0007, p < 0.01
-0.1
-0.05
0
0.05
0.1

Study	Sampling	Outcome	Subjects	Estimate [95% CI]
Canova et al., 2021	14-19 y	14-19 y	3258	-0.038 [-0.055; -0.022]
Liu et al., 2020	12 y	12 y	97	-0.007 [-0.137; 0.123]
Schillemans et al., 2023	12-18 years old	12-18 years old	997	0.004 [-0.012; 0.021]
**Summary**				**-0.028 [-0.072; 0.016]**

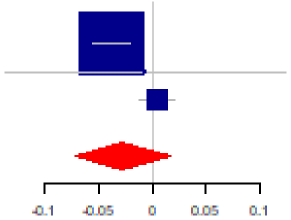

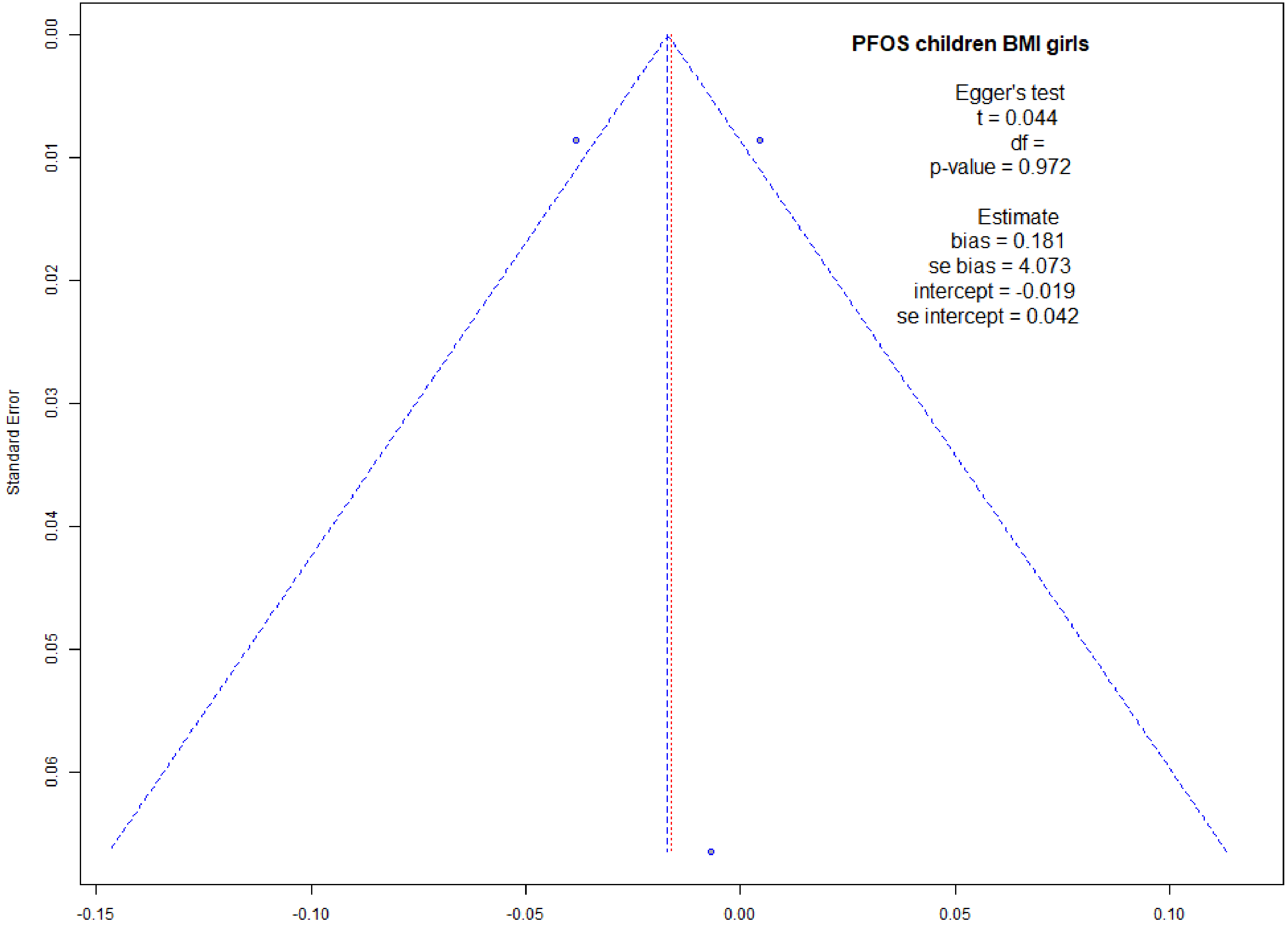
PFOS children BMI girls
Egger's test
t = 0.044
df =
p-value = 0.972
Estimate
bias = 0.181
se bias = 4.073
intercept = -0.019
se intercept = 0.042
Standard Error
0.00
0.01
0.02
0.03
0.04
0.05
0.06
-0.15
-0.10
-0.05
0.00
0.05
0.10

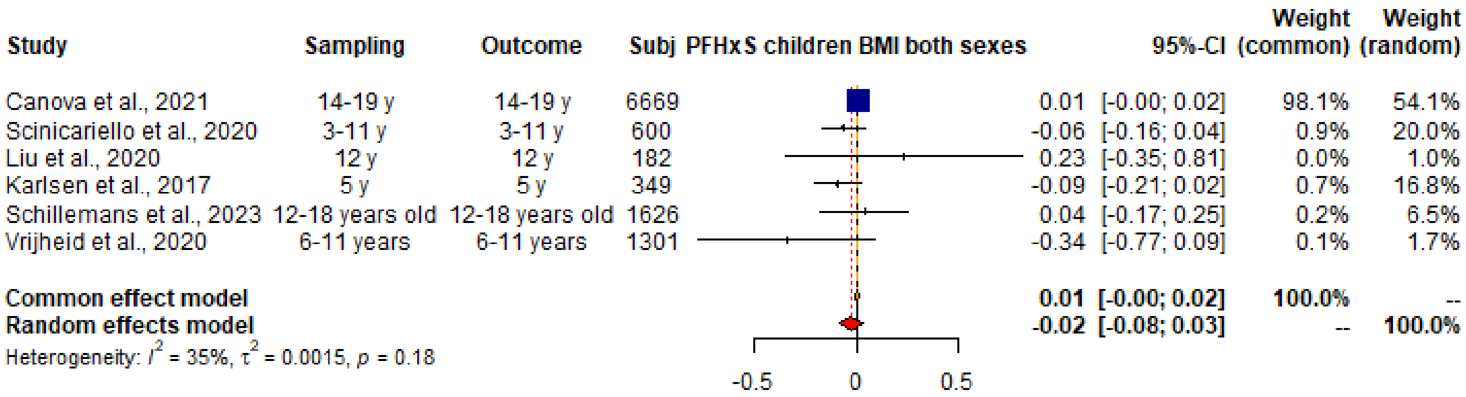
Study
Sampling
Outcome
Subj
PFHxS children BMI both sexes
95%-CI
Weight (common)
Weight (random)
Canova et al., 2021 14-19 y 14-19 y 6669 0.01 [-0.00; 0.02] 98.1% 54.1%
Scinicariello et al., 2020 3-11 y 3-11 y 600 -0.06 [-0.16; 0.04] 0.9% 20.0%
Liu et al., 2020 12 y 12 y 182 0.23 [-0.35; 0.81] 0.0% 1.0%
Karlsen et al., 2017 5 y 5 y 349 -0.09 [-0.21; 0.02] 0.7% 16.8%
Schillemans et al., 2023 12-18 years old 12-18 years old 1626 0.04 [-0.17; 0.25] 0.2% 6.5%
Vrijheid et al., 2020 6-11 years 6-11 years 1301 -0.34 [-0.77; 0.09] 0.1% 1.7%
Common effect model 0.01 [-0.00; 0.02] 100.0% --
Random effects model -0.02 [-0.08; 0.03] -- 100.0%
Heterogeneity: I^2 = 35%, τ^2 = 0.0015, p = 0.18
-0.5
0
0.5

Study	Sampling	Outcome	Subjects	Estimate [95% CI]
Canova et al., 2021	14-19 y	14-19 y	6669	0.007 [-0.002; 0.017]
Scinicariello et al., 2020	3-11 y	3-11 y	600	-0.062 [-0.163; 0.039]
Liu et al., 2020	12 y	12 y	182	0.230 [-0.346; 0.806]
Karlsen et al., 2017	5 y	5 y	349	-0.091 [-0.206; 0.024]
Schillemans et al., 2023	12-18 years old	12-18 years old	1626	0.038 [-0.171; 0.247]
Vrijheid et al., 2020	6-11 years	6-11 years	1301	-0.340 [-0.774; 0.094]
**Summary**				**-0.033 [-0.116; 0.049]**

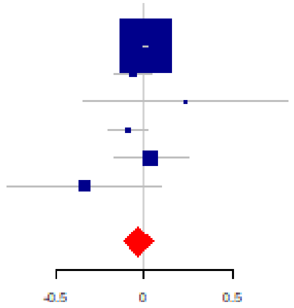

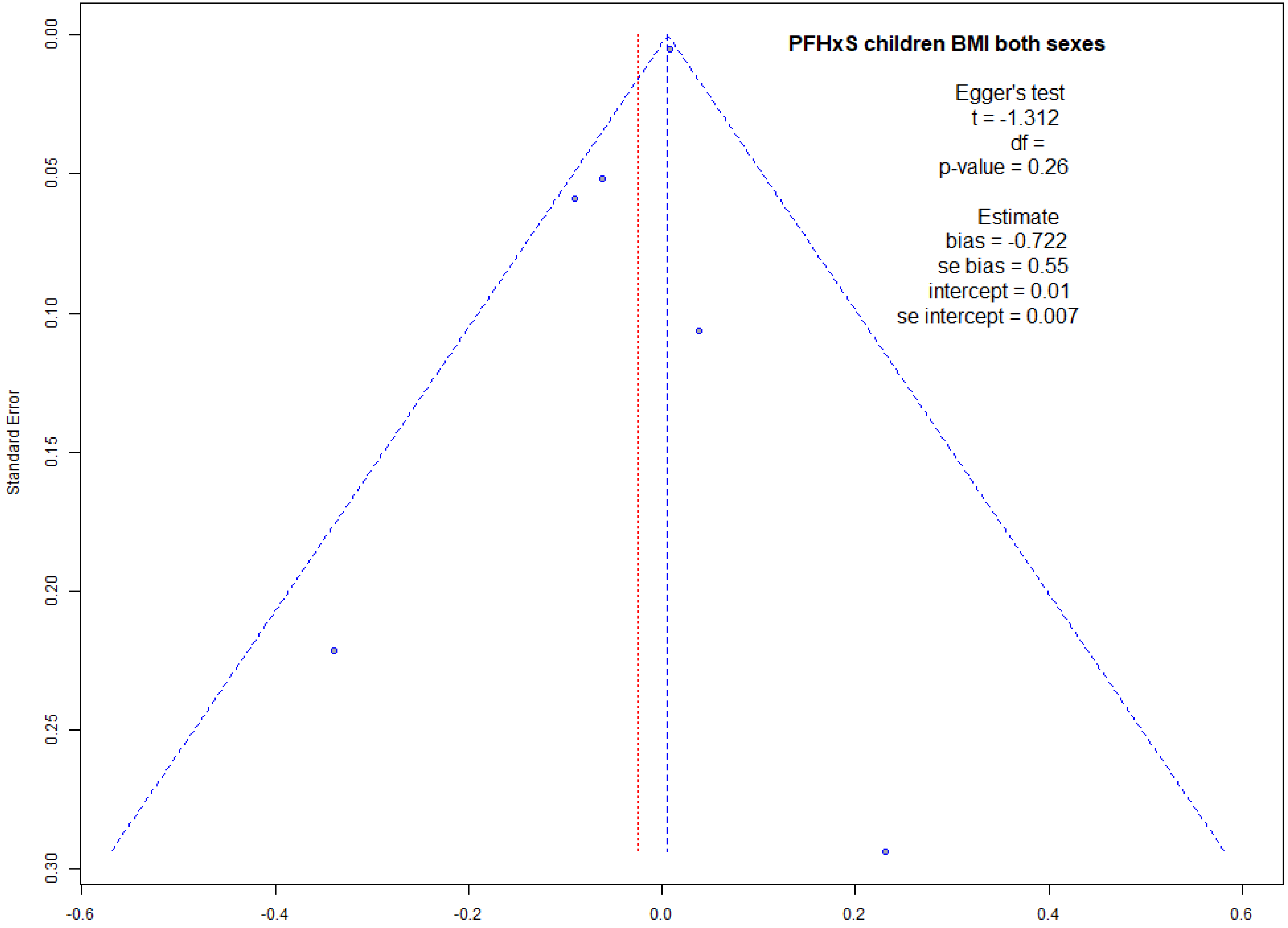
PFHxS children BMI both sexes
Egger's test
t = -1.312
df =
p-value = 0.26
Estimate
bias = -0.722
se bias = 0.55
intercept = 0.01
se intercept = 0.007
Standard Error
0.00
0.05
0.10
0.15
0.20
0.25
0.30
-0.6
-0.4
-0.2
0.0
0.2
0.4
0.6

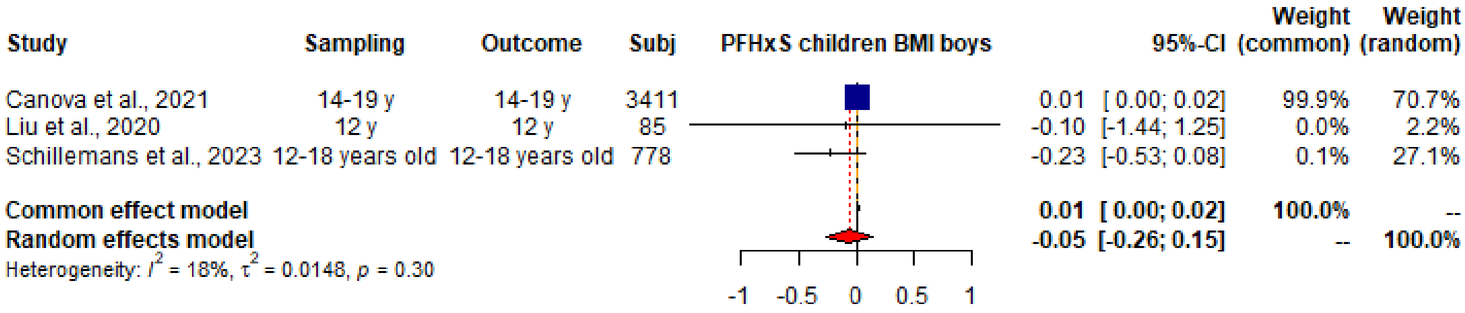
Study
Sampling
Outcome
Subj
PFHxS children BMI boys
95%-CI
Weight (common)
Weight (random)
Canova et al., 2021 | 14-19 y | 14-19 y | 3411 | 0.01 | [0.00; 0.02] | 99.9% | 70.7%
Liu et al., 2020 | 12 y | 12 y | 85 | -0.10 | [-1.44; 1.25] | 0.0% | 2.2%
Schillemans et al., 2023 | 12-18 years old | 12-18 years old | 778 | -0.23 | [-0.53; 0.08] | 0.1% | 27.1%
Common effect model | 0.01 | [0.00; 0.02] | 100.0% | --
Random effects model | -0.05 | [-0.26; 0.15] | -- | 100.0%
Heterogeneity: I^2 = 18%, τ^2 = 0.0148, p = 0.30
-1
-0.5
0
0.5
1

Study	Sampling	Outcome	Subjects	Estimate [95% CI]
Canova et al., 2021	14-19 y	14-19 y	3411	0.013 [0.002; 0.023]
Liu et al., 2020	12 y	12 y	85	-0.096 [-1.440; 1.248]
Schillemans et al., 2023	12-18 years old	12-18 years old	778	-0.228 [-0.531; 0.076]
**Summary**				**-0.033 [-0.162; 0.096]**

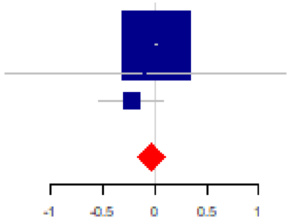

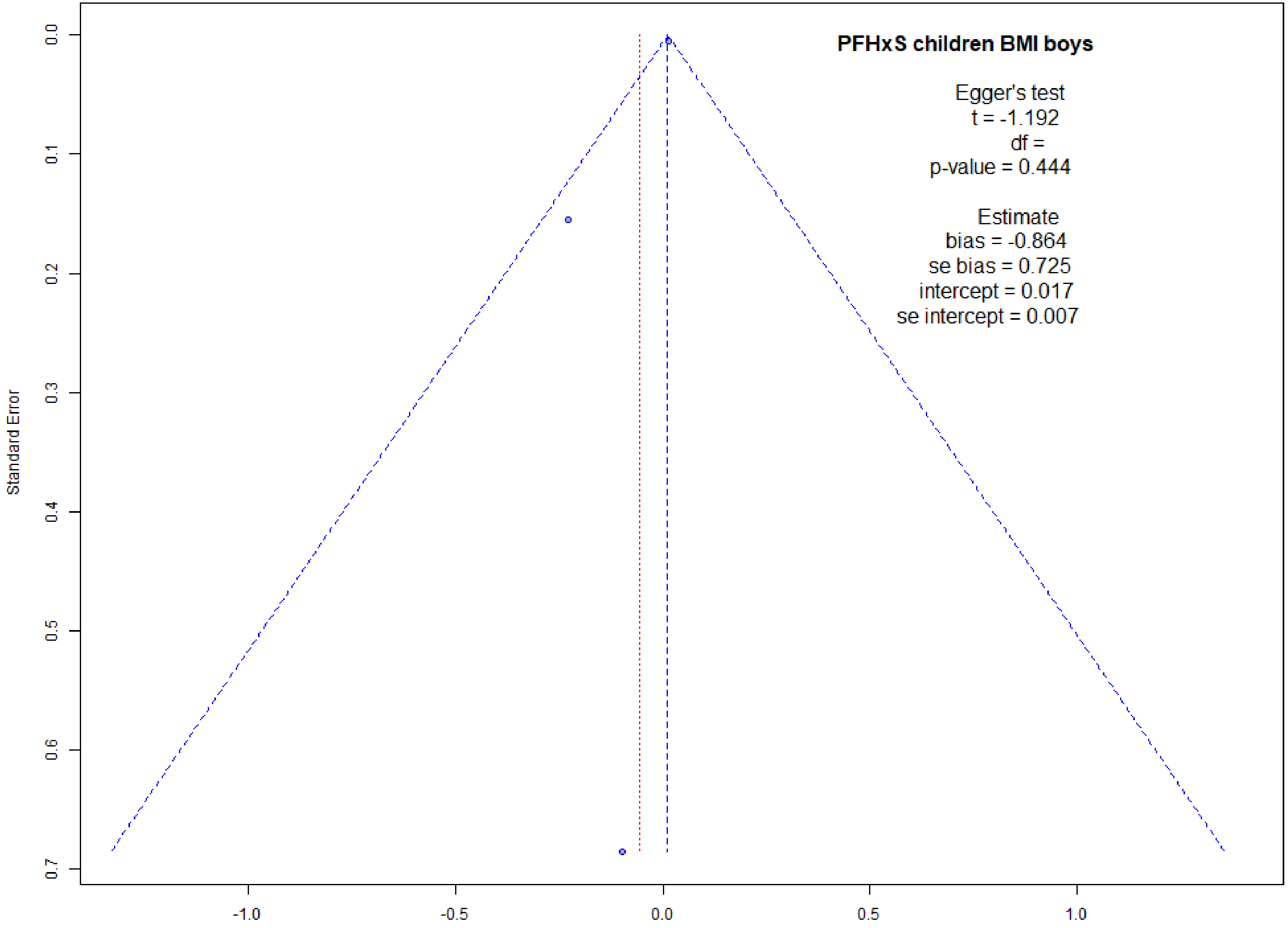
PFHxS children BMI boys
Egger's test
t = -1.192
df =
p-value = 0.444
Estimate
bias = -0.864
se bias = 0.725
intercept = 0.017
se intercept = 0.007
Standard Error
0.0
0.1
0.2
0.3
0.4
0.5
0.6
0.7
-1.0
-0.5
0.0
0.5
1.0

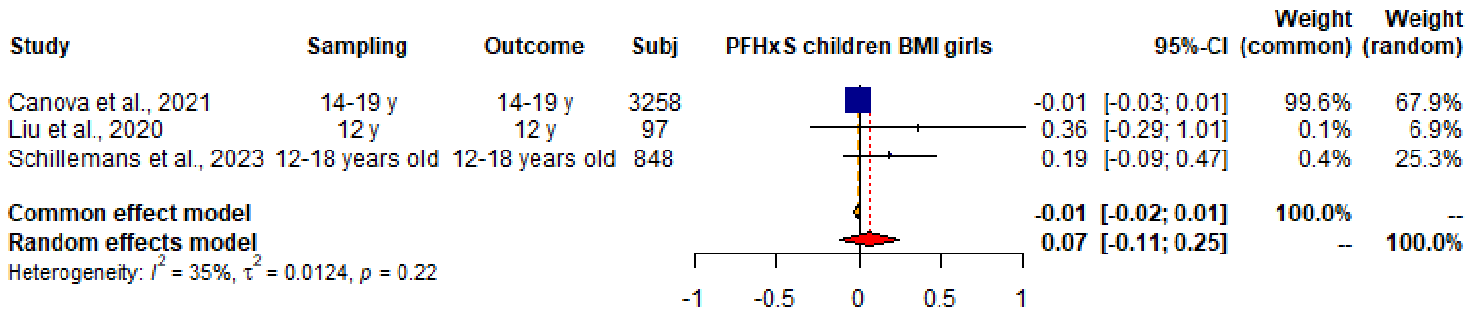
Study
Sampling
Outcome
Subj
PFHxS children BMI girls
95%-CI
Weight (common)
Weight (random)
Canova et al., 2021
14-19 y
14-19 y
3258
-0.01 [-0.03; 0.01]
99.6%
67.9%
Liu et al., 2020
12 y
12 y
97
0.36 [-0.29; 1.01]
0.1%
6.9%
Schillemans et al., 2023
12-18 years old
12-18 years old
848
0.19 [-0.09; 0.47]
0.4%
25.3%
Common effect model
-0.01 [-0.02; 0.01]
100.0%
--
Random effects model
0.07 [-0.11; 0.25]
--
100.0%
Heterogeneity: I^2 = 35%, τ^2 = 0.0124, p = 0.22
-1
-0.5
0
0.5
1

Study	Sampling	Outcome	Subjects	Estimate [95% CI]
Canova et al., 2021	14-19 y	14-19 y	3258	-0.009 [-0.026; 0.009]
Liu et al., 2020	12 y	12 y	97	0.358 [-0.294; 1.011]
Schillemans et al., 2023	12-18 years old	12-18 years old	848	0.190 [-0.095; 0.475]
**Summary**				**0.040 [-0.125; 0.205]**

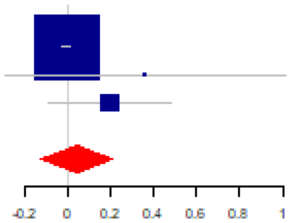

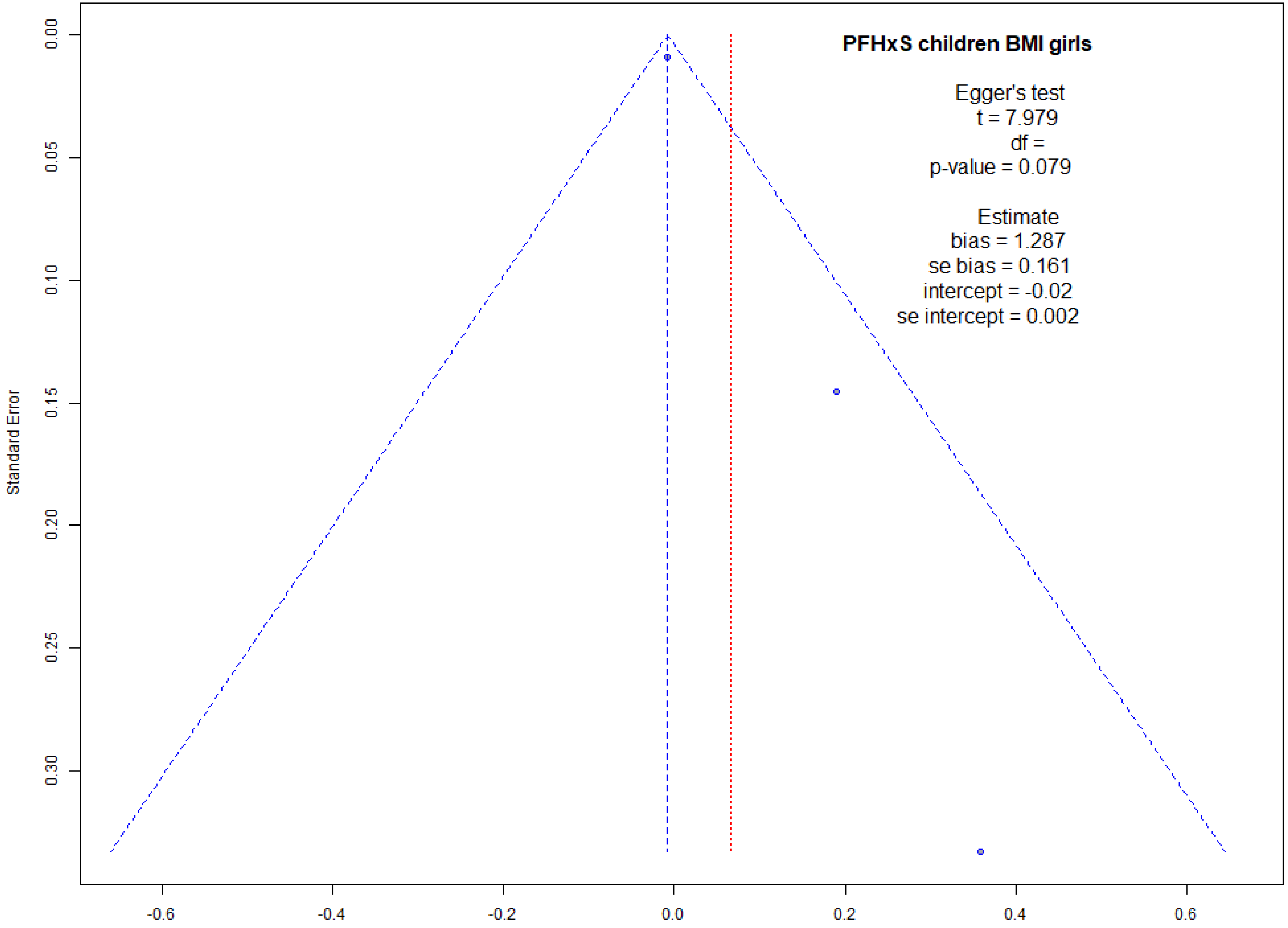
PFHxS children BMI girls
Egger's test
t = 7.979
df =
p-value = 0.079
Estimate
bias = 1.287
se bias = 0.161
intercept = -0.02
se intercept = 0.002
Standard Error
0.00
0.05
0.10
0.15
0.20
0.25
0.30
-0.6
-0.4
-0.2
0.0
0.2
0.4
0.6

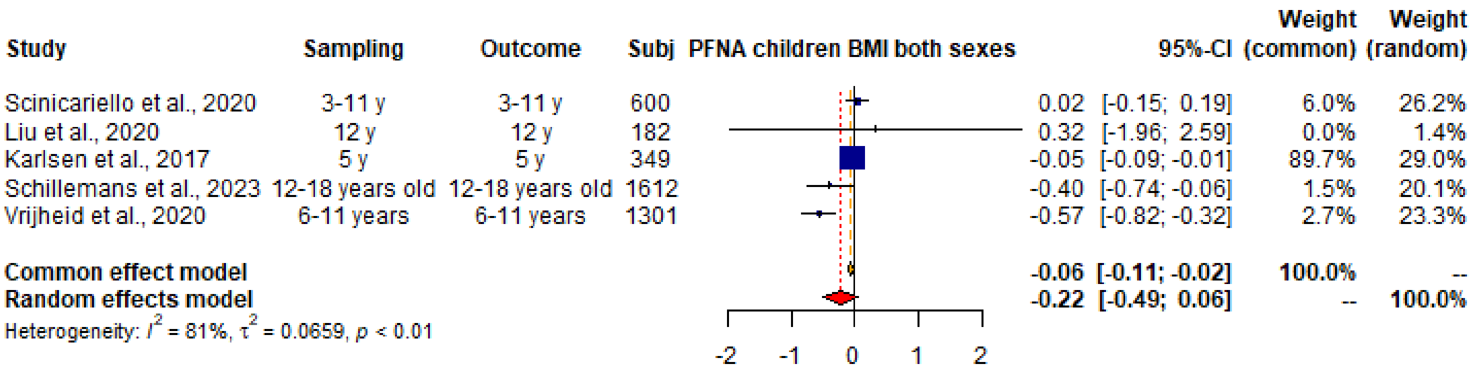
Study
Sampling
Outcome
Subj
PFNA children BMI both sexes
95%-CI
Weight (common)
Weight (random)
Scinicariello et al., 2020 3-11 y 3-11 y 600 0.02 [-0.15; 0.19] 6.0% 26.2%
Liu et al., 2020 12 y 12 y 182 0.32 [-1.96; 2.59] 0.0% 1.4%
Karlsen et al., 2017 5 y 5 y 349 -0.05 [-0.09; -0.01] 89.7% 29.0%
Schillemans et al., 2023 12-18 years old 12-18 years old 1612 -0.40 [-0.74; -0.06] 1.5% 20.1%
Vrijheid et al., 2020 6-11 years 6-11 years 1301 -0.57 [-0.82; -0.32] 2.7% 23.3%
Common effect model -0.06 [-0.11; -0.02] 100.0% --
Random effects model -0.22 [-0.49; 0.06] -- 100.0%
Heterogeneity: I^2 = 81%, τ^2 = 0.0659, p < 0.01
-2
-1
0
1
2

Study	Sampling	Outcome	Subjects	Estimate [95% CI]
Scinicariello et al., 2020	3-11 y	3-11 y	600	0.024 [-0.146; 0.195]
Liu et al., 2020	12 y	12 y	182	0.317 [-1.957; 2.591]
Karlsen et al., 2017	5 y	5 y	349	-0.050 [-0.094; -0.006]
Schillemans et al., 2023	12-18 years old	12-18 years old	1612	-0.397 [-0.737; -0.057]
Vrijheid et al., 2020	6-11 years	6-11 years	1301	-0.570 [-0.823; -0.317]
**Summary**				**-0.328 [-0.612; -0.044]**

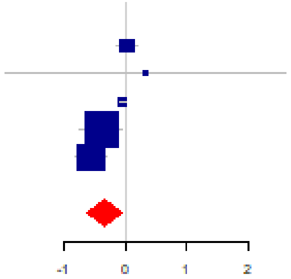

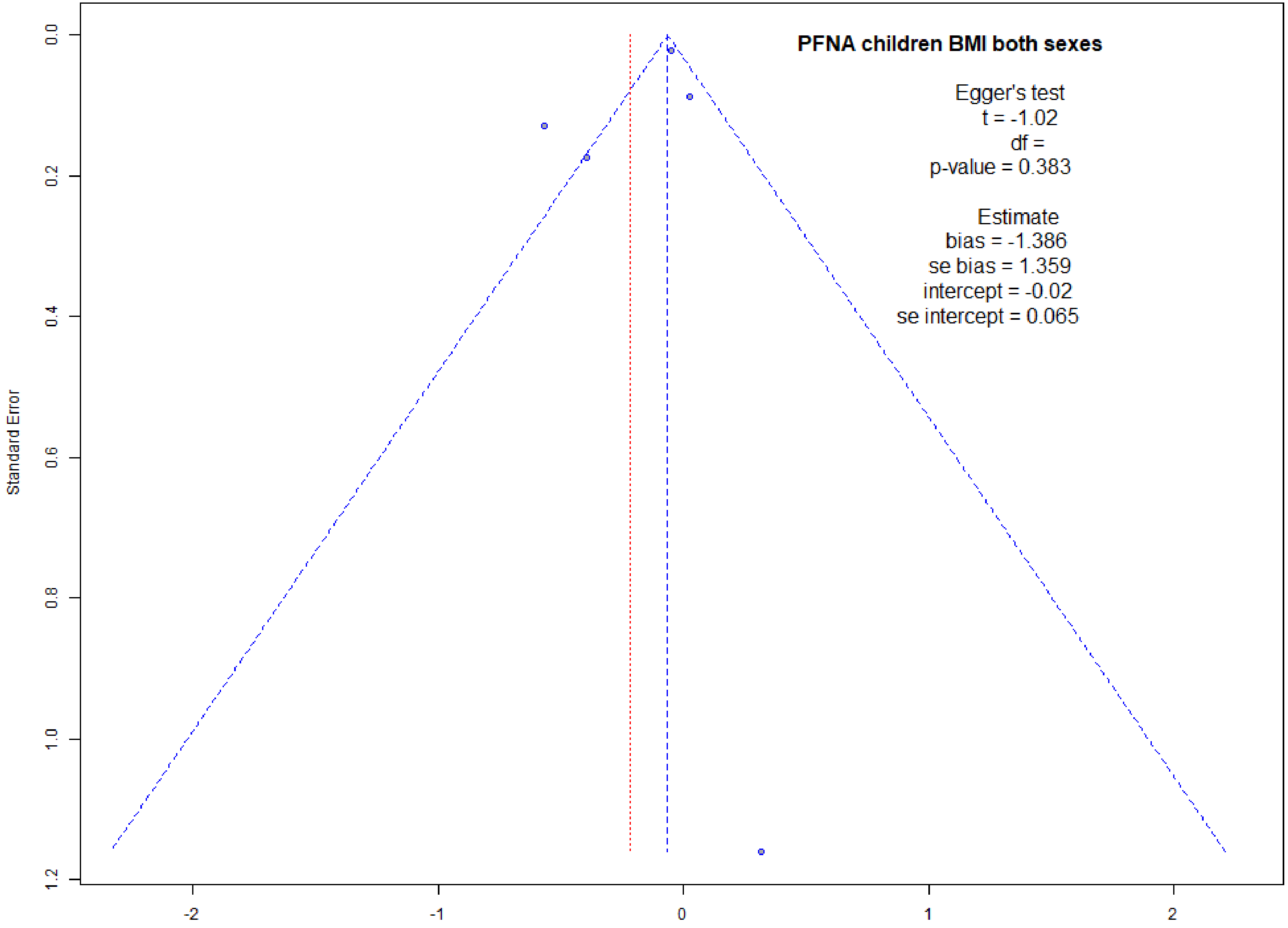
PFNA children BMI both sexes
Egger's test
t = -1.02
df =
p-value = 0.383
Estimate
bias = -1.386
se bias = 1.359
intercept = -0.02
se intercept = 0.065
Standard Error
0.0
0.2
0.4
0.6
0.8
1.0
1.2
-2
-1
0
1
2

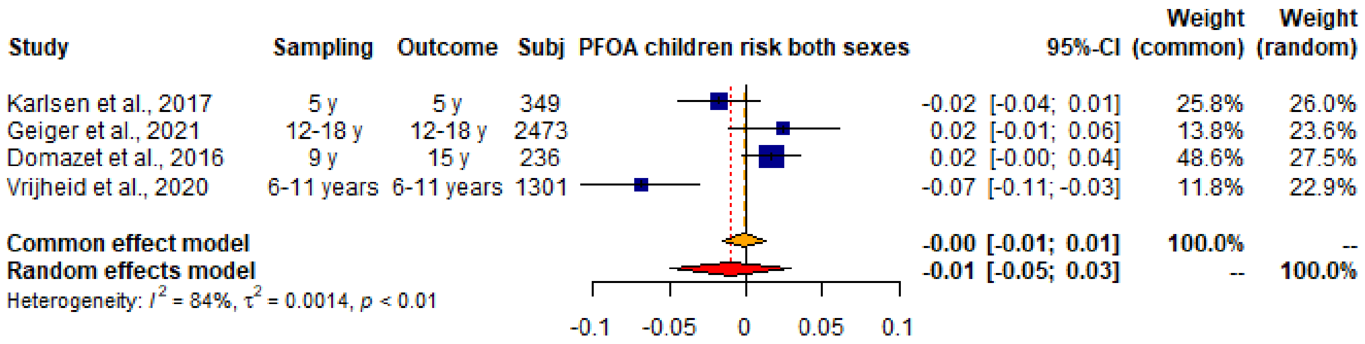
Study
Sampling
Outcome
Subj
PFOA children risk both sexes
95%-CI
Weight (common)
Weight (random)
Karlsen et al., 2017 5 y 5 y 349 -0.02 [-0.04; 0.01] 25.8% 26.0%
Geiger et al., 2021 12-18 y 12-18 y 2473 0.02 [-0.01; 0.06] 13.8% 23.6%
Domazet et al., 2016 9 y 15 y 236 0.02 [-0.00; 0.04] 48.6% 27.5%
Vrijheid et al., 2020 6-11 years 6-11 years 1301 -0.07 [-0.11; -0.03] 11.8% 22.9%
Common effect model -0.00 [-0.01; 0.01] 100.0% --
Random effects model -0.01 [-0.05; 0.03] -- 100.0%
Heterogeneity: I^2 = 84%, τ^2 = 0.0014, p < 0.01
-0.1
-0.05
0
0.05
0.1

Study	Sampling	Outcome	Subjects	Estimate [95% CI]
Karlsen et al., 2017	5 y	5 y	349	-0.018 [-0.044; 0.009]
Geiger et al., 2021	12-18 y	12-18 y	2473	0.024 [-0.012; 0.060]
Domazet et al., 2016	9 y	15 y	236	0.017 [-0.003; 0.036]
Vrijheid et al., 2020	6-11 years	6-11 years	1301	-0.069 [-0.108; -0.030]
**Summary**				**-0.007 [-0.055; 0.041]**

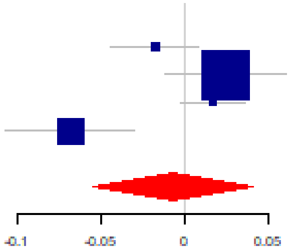

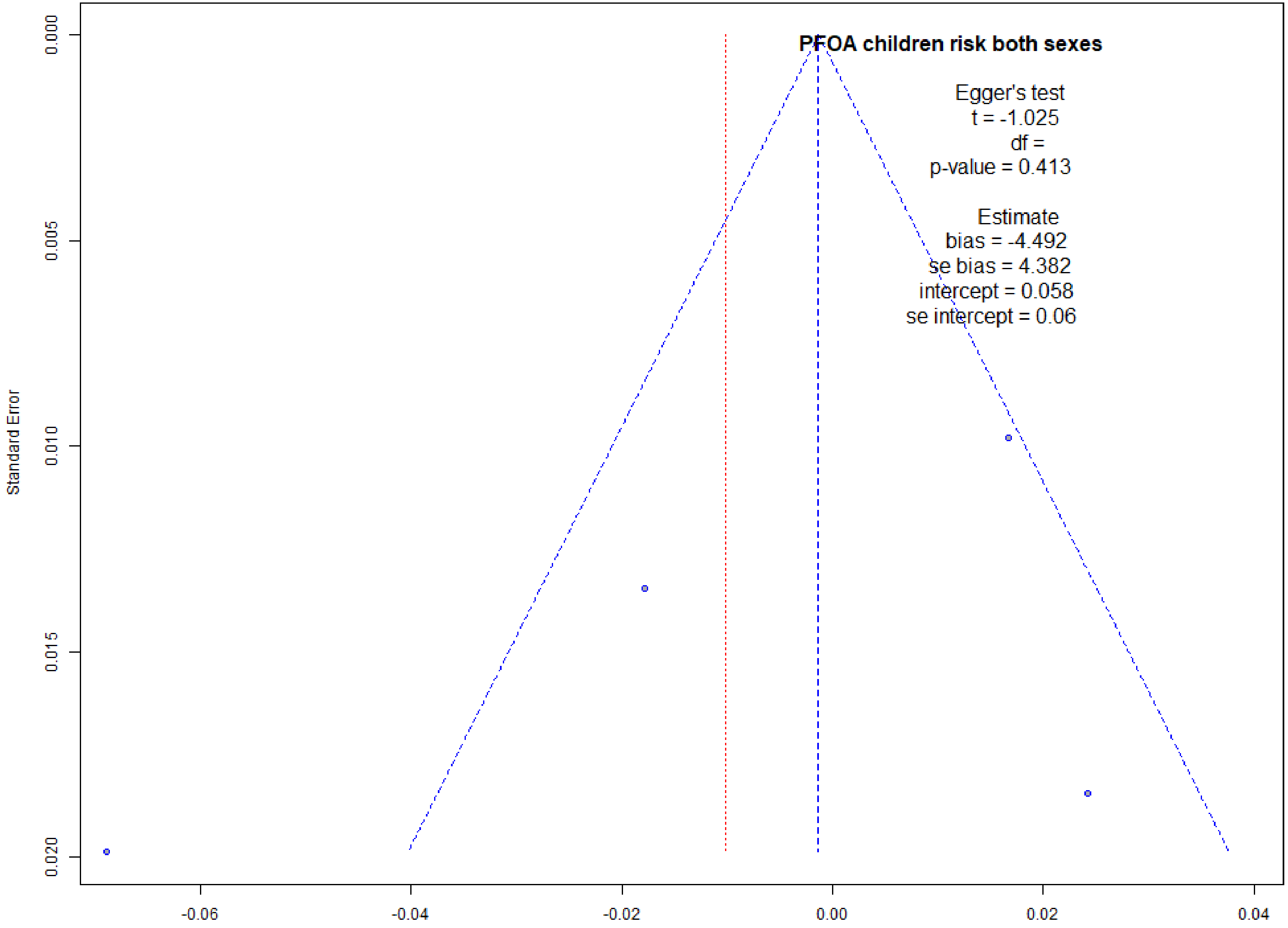
PFOA children risk both sexes
Egger's test
t = -1.025
df =
p-value = 0.413
Estimate
bias = -4.492
se bias = 4.382
intercept = 0.058
se intercept = 0.06
Standard Error
0.000
0.005
0.010
0.015
0.020
-0.06
-0.04
-0.02
0.00
0.02
0.04

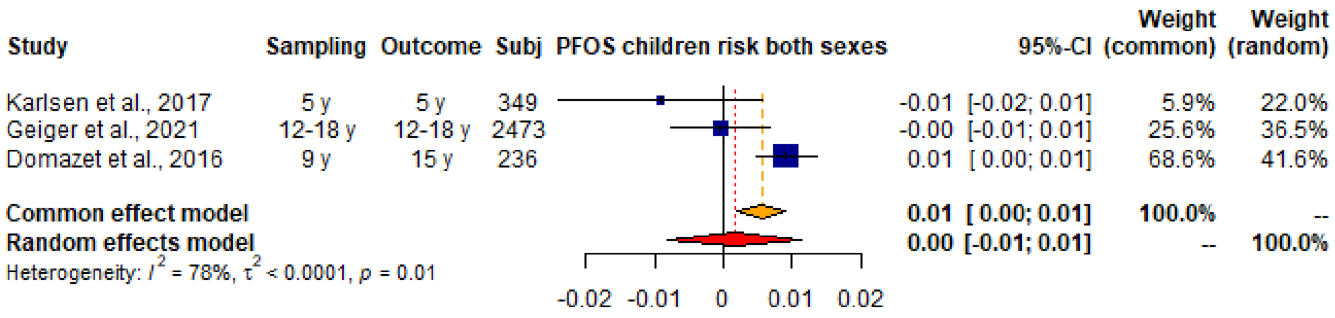
Study
Sampling
Outcome
Subj
PFOS children risk both sexes
95%-CI
Weight (common)
Weight (random)
Karlsen et al., 2017
5 y
5 y
349
-0.01 [-0.02; 0.01]
5.9%
22.0%
Geiger et al., 2021
12-18 y
12-18 y
2473
-0.00 [-0.01; 0.01]
25.6%
36.5%
Domazet et al., 2016
9 y
15 y
236
0.01 [0.00; 0.01]
68.6%
41.6%
Common effect model
0.01 [0.00; 0.01]
100.0%
--
Random effects model
0.00 [-0.01; 0.01]
--
100.0%
Heterogeneity: I^2 = 78%, τ^2 < 0.0001, p = 0.01
-0.02
-0.01
0
0.01
0.02

Study	Sampling	Outcome	Subjects	Estimate [95% CI]
Karlsen et al., 2017	5 y	5 y	349	-0.009 [-0.024; 0.006]
Geiger et al., 2021	12-18 y	12-18 y	2473	0.000 [-0.008; 0.007]
Domazet et al., 2016	9 y	15 y	236	0.009 [0.005; 0.013]
**Summary**				**-0.001 [-0.014; 0.012]**

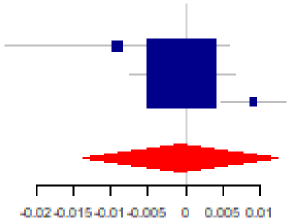

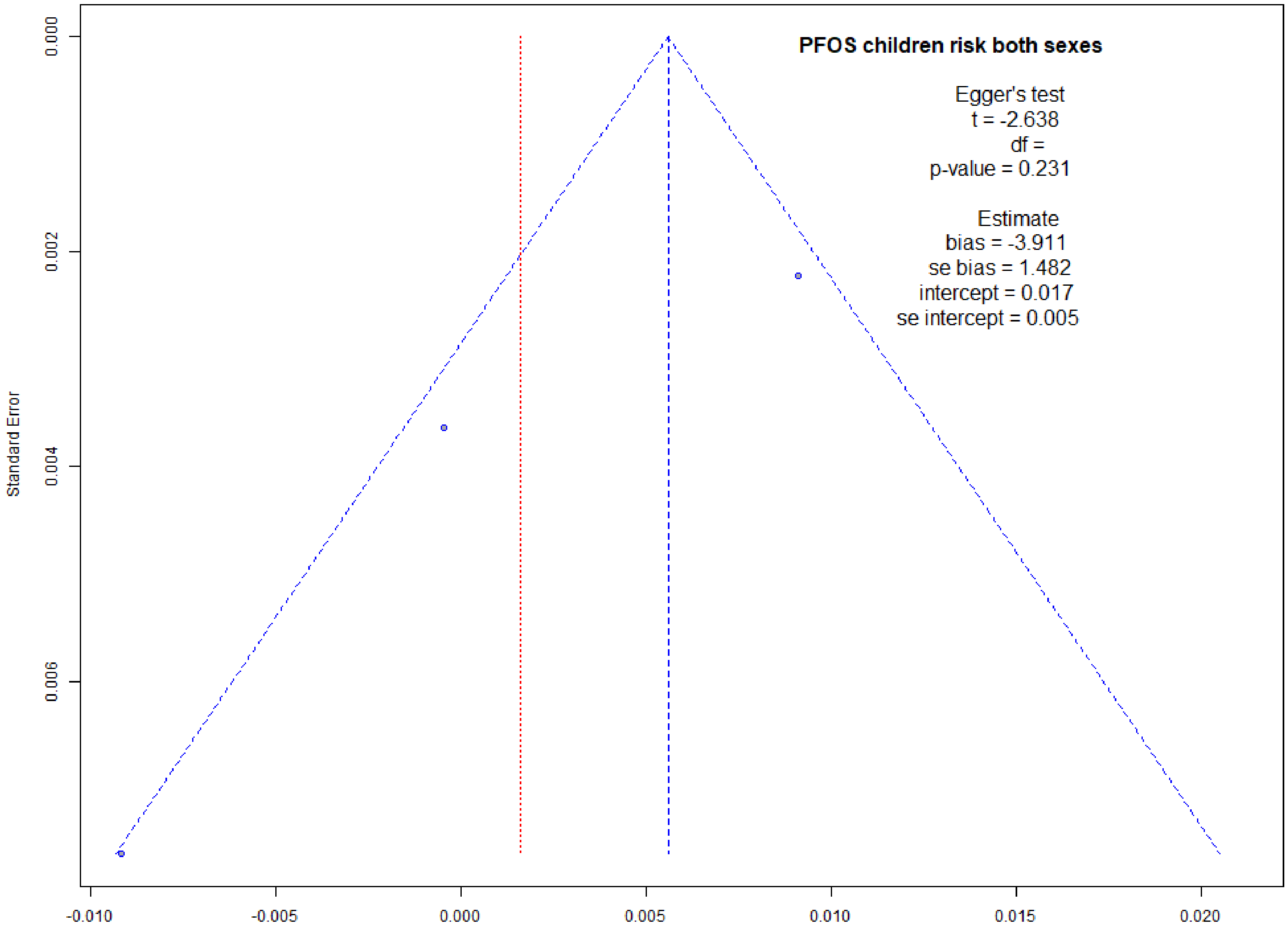
PFOS children risk both sexes
Egger's test
t = -2.638
df =
p-value = 0.231
Estimate
bias = -3.911
se bias = 1.482
intercept = 0.017
se intercept = 0.005
Standard Error
0.000
0.002
0.004
0.006
-0.010
-0.005
0.000
0.005
0.010
0.015
0.020

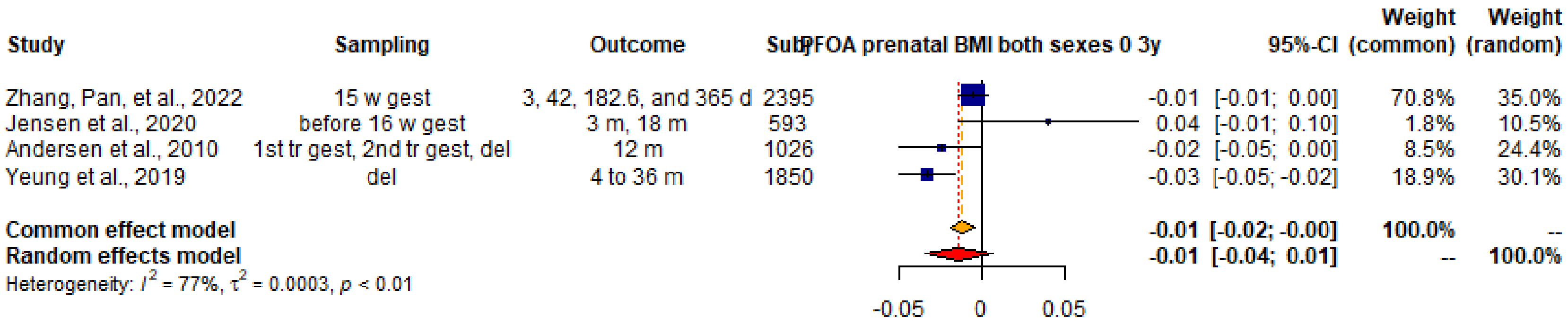
Study
Sampling
Outcome
Sub
PFOA prenatal BMI both sexes 0 3y
95%-CI
Weight (common)
Weight (random)
Zhang, Pan, et al., 2022 | 15 w gest | 3, 42, 182.6, and 365 d | 2395 | -0.01 [-0.01; 0.00] | 70.8% | 35.0%
Jensen et al., 2020 | before 16 w gest | 3 m, 18 m | 593 | 0.04 [-0.01; 0.10] | 1.8% | 10.5%
Andersen et al., 2010 | 1st tr gest, 2nd tr gest, del | 12 m | 1026 | -0.02 [-0.05; 0.00] | 8.5% | 24.4%
Yeung et al., 2019 | del | 4 to 36 m | 1850 | -0.03 [-0.05; -0.02] | 18.9% | 30.1%
Common effect model | -0.01 [-0.02; -0.00] | 100.0% | --
Random effects model | -0.01 [-0.04; 0.01] | -- | 100.0%
Heterogeneity: I^2 = 77%, τ^2 = 0.0003, p < 0.01
-0.05
0
0.05

Study	Sampling	Outcome	Subjects	Estimate [95% CI]
Zhang, Pan, et al., 2022	15 w gest	3, 42, 182.6, and 365 d	2395	-0.006 [-0.014; 0.003]
Jensen et al., 2020	before 16 w gest	3 m, 18 m	593	0.040 [-0.015; 0.095]
Andersen et al., 2010	1st tr gest, 2nd tr gest, del	12 m	1026	-0.025 [-0.050; 0.000]
Yeung et al., 2019	del	4 to 36 m	1850	-0.034 [-0.050; -0.017]
**Summary**				**-0.013 [-0.034; 0.008]**

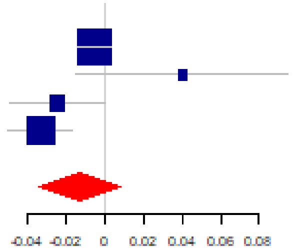

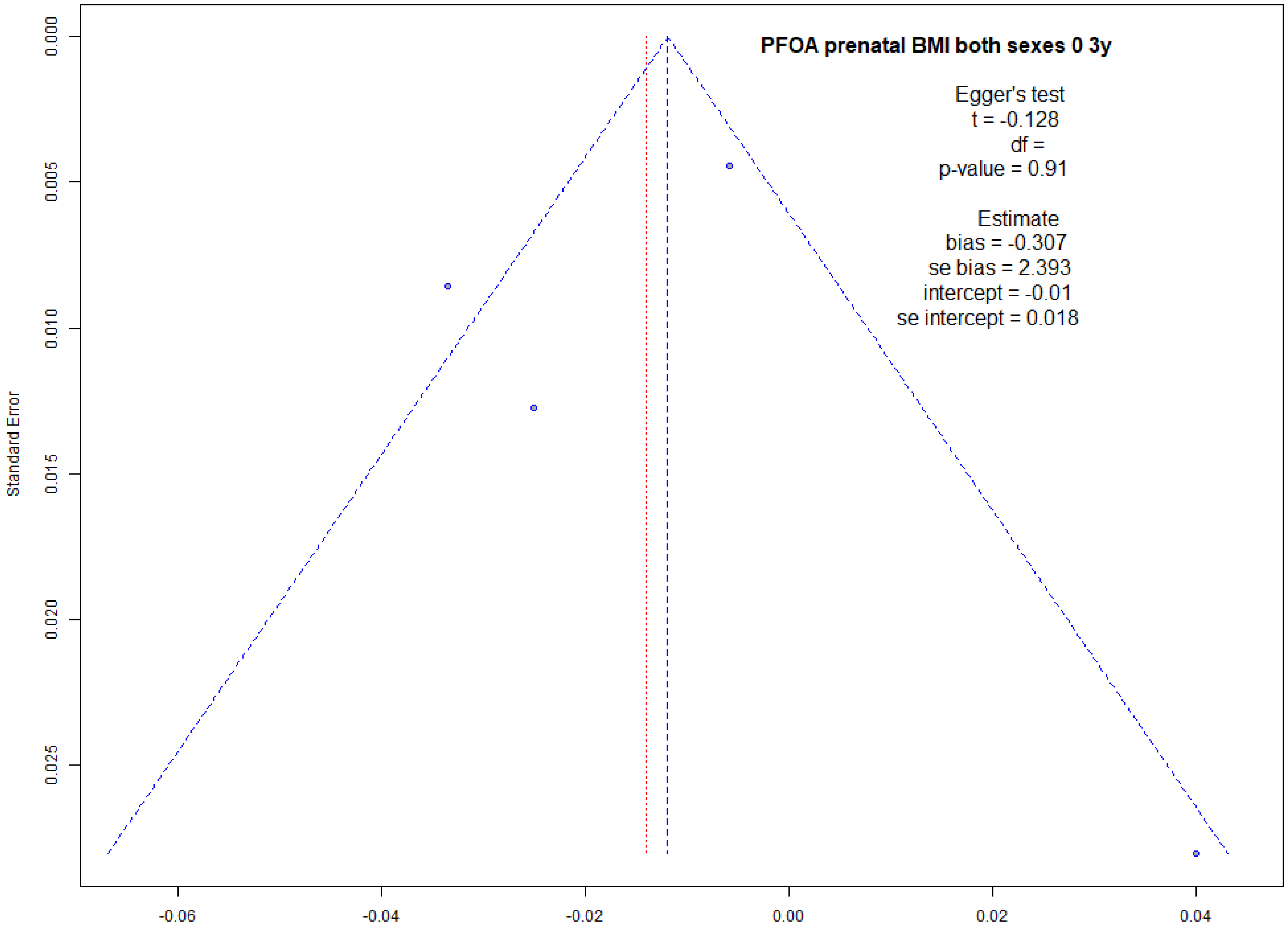
PFOA prenatal BMI both sexes 0 3y
Egger's test
t = -0.128
df =
p-value = 0.91
Estimate
bias = -0.307
se bias = 2.393
intercept = -0.01
se intercept = 0.018
Standard Error
0.000
0.005
0.010
0.015
0.020
0.025
-0.06
-0.04
-0.02
0.00
0.02
0.04

PFOA prenatal BMI both sexes 4 18y

Study	Sampling	Outcome	Subj		95%-CI	Weight (common)	Weight (random)
Zhang, Lei, et al., 2022	within 3 d before del	7 y	206	-0.00	[-0.00; 0.00]	30.2%	46.9%
Bloom et al., 2022	8-13 w gest	4-8 y	661	-0.01	[-0.03; 0.01]	0.0%	0.2%
Lauritzen et al., 2018	17-20 w gest	5 y	412	0.09	[-0.01; 0.19]	0.0%	0.0%
Gyllenhammar et al., 2018	3 w after del	5 y	183	0.11	[-0.03; 0.26]	0.0%	0.0%
Sevelsted et al., 2022	24 w gest - 1 w after del	10 y	536	0.18	[0.00; 0.36]	0.0%	0.0%
Liu et al., 2020	del	12 y	212	-0.01	[-0.06; 0.04]	0.0%	0.0%
Mora et al., 2017	9.6 w gest	7.7 y	871	0.01	[-0.01; 0.04]	0.0%	0.2%
Chen et al., 2019	del	5 y	404	0.00	[-0.00; 0.00]	69.8%	52.6%
Chen et al., 2017	del	60 to 108 m	429	0.01	[-0.09; 0.11]	0.0%	0.0%
Karlsen et al., 2017	2 w after del	5 y	371	0.04	[-0.00; 0.08]	0.0%	0.0%
Manzano-Salgado et al., 2017	1st tr gest	7 y	1086	0.02	[-0.04; 0.07]	0.0%	0.0%
Hoyer et al., 2015 - Greenland	24 ± 10 w gest	5-9 y	531	0.05	[-0.06; 0.16]	0.0%	0.0%
Hoyer et al., 2015 - Ukraine	24 ± 10 w gest	5-9 y	491	0.01	[-0.13; 0.16]	0.0%	0.0%
Vrijheid et al., 2020	during gest	6-11 years	1301	-0.01	[-0.03; 0.02]	0.0%	0.1%
**Common effect model**				**0.00**	**[-0.00; 0.00]**	**100.0%**	--
**Random effects model**				**0.00**	**[-0.00; 0.00]**	--	**100.0%**

Heterogeneity: $I^2 = 24\%$, $\tau^2 < 0.0001$, $p = 0.19$

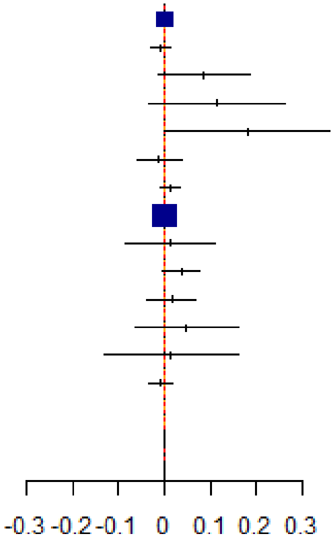

Study	Sampling	Outcome	Subjects	Estimate [95% CI]
Zhang, Lei, et al., 2022	within 3 d before del	7 y	206	0.000 [-0.001; 0.000]
Bloom et al., 2022	8-13 w gest	4-8 y	661	-0.010 [-0.031; 0.011]
Lauritzen et al., 2018	17-20 w gest	5 y	412	0.086 [-0.014; 0.186]
Gyllenhammar et al., 2018	3 w after del	5 y	183	0.114 [-0.032; 0.261]
Sevelsted et al., 2022	24 w gest - 1 w after del	10 y	536	0.180 [0.000; 0.360]
Liu et al., 2020	del	12 y	212	-0.012 [-0.061; 0.037]
Mora et al., 2017	9.6 w gest	7.7 y	871	0.014 [-0.008; 0.035]
Chen et al., 2019	del	5 y	404	0.000 [0.000; 0.000]
Chen et al., 2017	del	60 to 108 m	429	0.013 [-0.087; 0.112]
Karlsen et al., 2017	2 w after del	5 y	371	0.037 [-0.002; 0.077]
Manzano-Salgado et al., 2017	1st tr gest	7 y	1086	0.016 [-0.039; 0.070]
Hoyer et al., 2015 - Greenland	24 ± 10 w gest	5-9 y	531	0.049 [-0.064; 0.161]
Hoyer et al., 2015 - Ukraine	24 ± 10 w gest	5-9 y	491	0.014 [-0.132; 0.159]
Vrijheid et al., 2020	during gest	6-11 years	1301	-0.009 [-0.034; 0.015]
**Summary**				**0.028 [0.006; 0.049]**

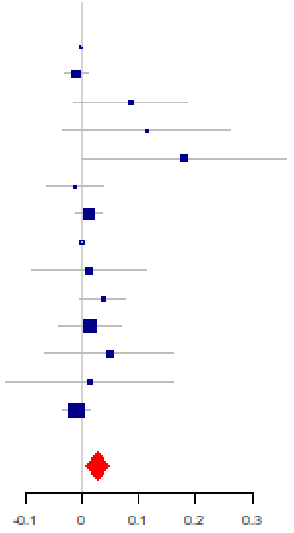

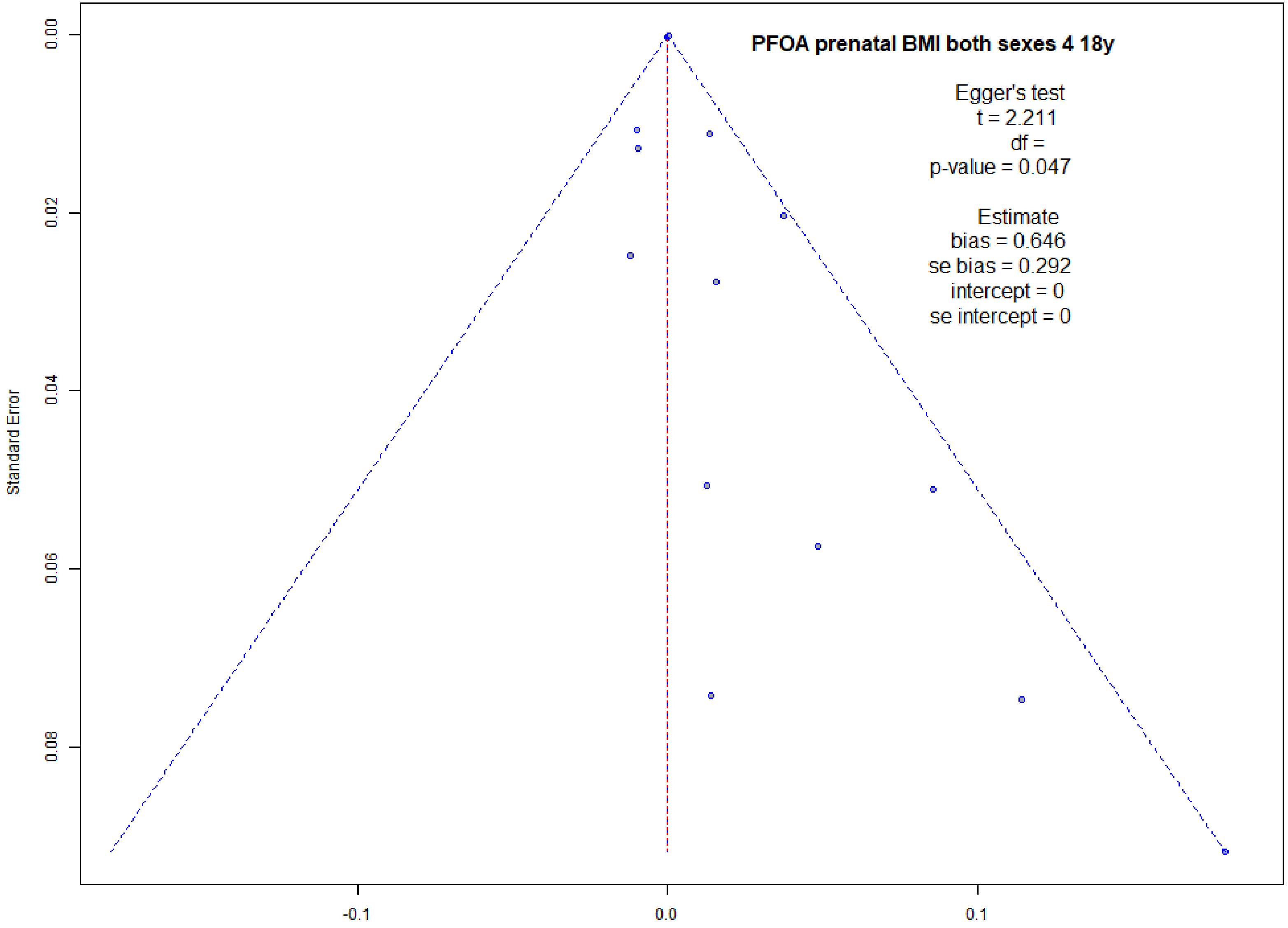
PFOA prenatal BMI both sexes 4 18y
Egger's test
t = 2.211
df =
p-value = 0.047
Estimate
bias = 0.646
se bias = 0.292
intercept = 0
se intercept = 0
Standard Error
0.00
0.02
0.04
0.06
0.08
-0.1
0.0
0.1

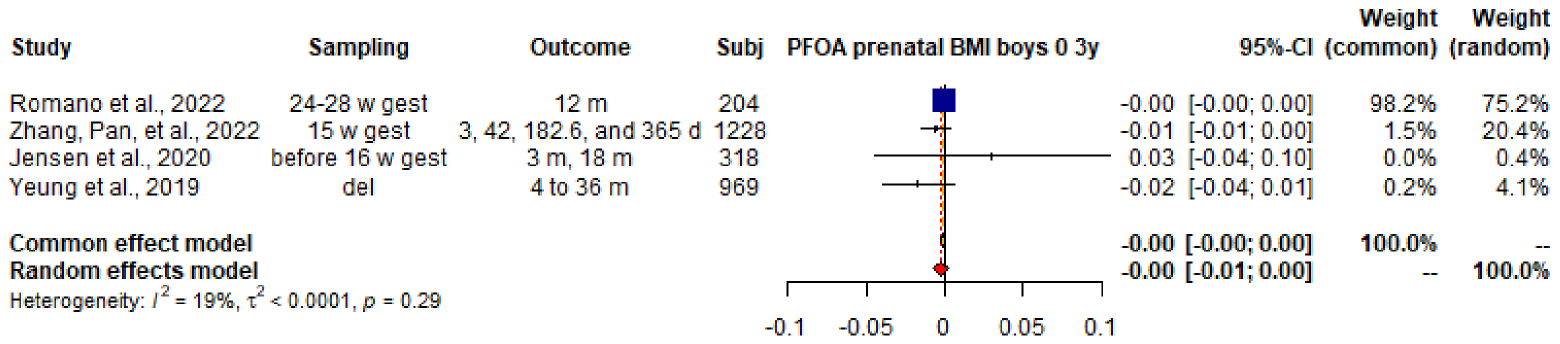
Study
Sampling
Outcome
Subj
PFOA prenatal BMI boys 0 3y
95%-CI
Weight (common)
Weight (random)
Romano et al., 2022 24-28 w gest 12 m 204 -0.00 [-0.00; 0.00] 98.2% 75.2%
Zhang, Pan, et al., 2022 15 w gest 3, 42, 182.6, and 365 d 1228 -0.01 [-0.01; 0.00] 1.5% 20.4%
Jensen et al., 2020 before 16 w gest 3 m, 18 m 318 0.03 [-0.04; 0.10] 0.0% 0.4%
Yeung et al., 2019 del 4 to 36 m 969 -0.02 [-0.04; 0.01] 0.2% 4.1%
Common effect model -0.00 [-0.00; 0.00] 100.0% --
Random effects model -0.00 [-0.01; 0.00] -- 100.0%
Heterogeneity: I^2 = 19%, τ^2 < 0.0001, p = 0.29
-0.1
-0.05
0
0.05
0.1

Study	Sampling	Outcome	Subjects	Estimate [95% CI]
Romano et al., 2022	24-28 w gest	12 m	204	-0.001 [-0.002; 0.001]
Zhang, Pan, et al., 2022	15 w gest	3, 42, 182.6, and 365 d	1228	-0.006 [-0.015; 0.003]
Jensen et al., 2020	before 16 w gest	3 m, 18 m	318	0.030 [-0.045; 0.105]
Yeung et al., 2019	del	4 to 36 m	969	-0.017 [-0.040; 0.006]
**Summary**				**-0.005 [-0.018; 0.008]**

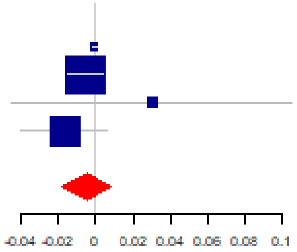

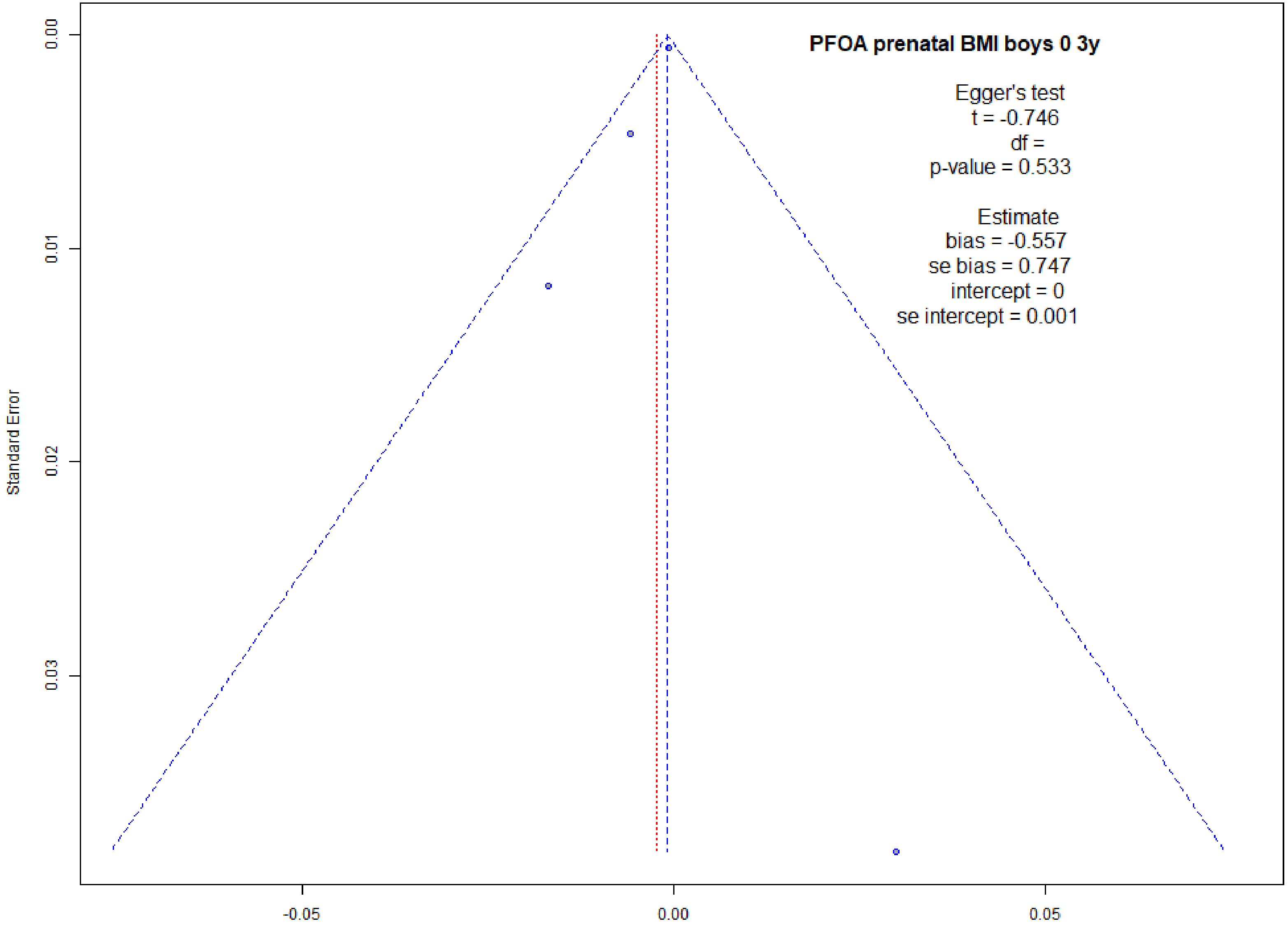
PFOA prenatal BMI boys 0 3y
Egger's test
t = -0.746
df =
p-value = 0.533
Estimate
bias = -0.557
se bias = 0.747
intercept = 0
se intercept = 0.001
Standard Error
0.00
0.01
0.02
0.03
-0.05
0.00
0.05

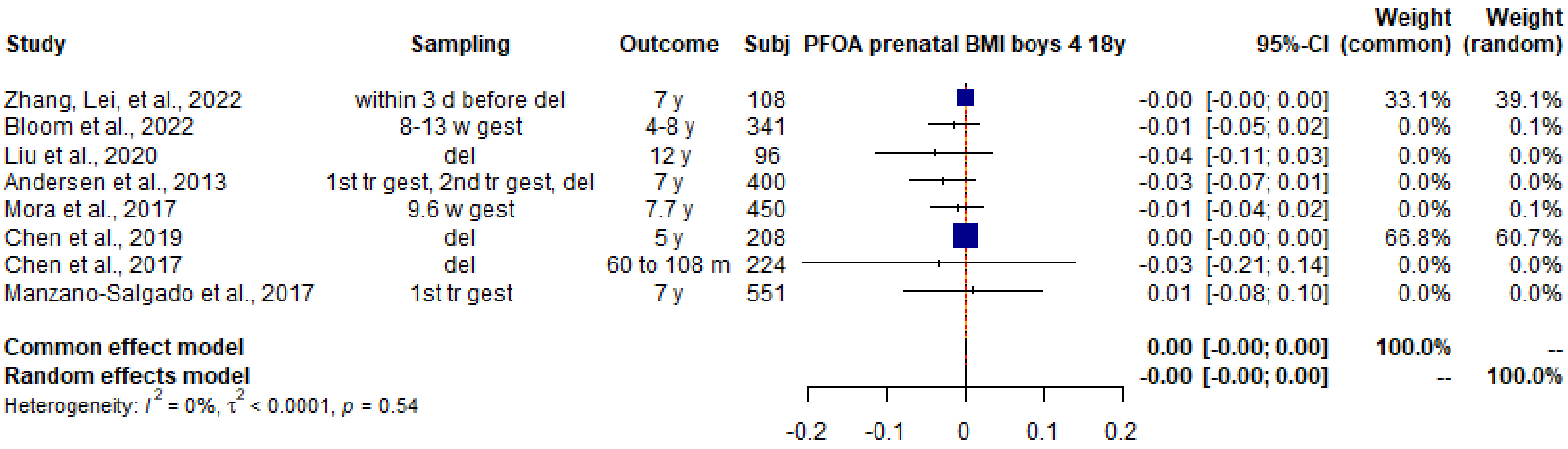
Study
Sampling
Outcome
Subj
PFOA prenatal BMI boys 4 18y
95%-CI
Weight (common)
Weight (random)
Zhang, Lei, et al., 2022 | within 3 d before del | 7 y | 108 | -0.00 [-0.00; 0.00] | 33.1% | 39.1%
Bloom et al., 2022 | 8-13 w gest | 4-8 y | 341 | -0.01 [-0.05; 0.02] | 0.0% | 0.1%
Liu et al., 2020 | del | 12 y | 96 | -0.04 [-0.11; 0.03] | 0.0% | 0.0%
Andersen et al., 2013 | 1st tr gest, 2nd tr gest, del | 7 y | 400 | -0.03 [-0.07; 0.01] | 0.0% | 0.0%
Mora et al., 2017 | 9.6 w gest | 7.7 y | 450 | -0.01 [-0.04; 0.02] | 0.0% | 0.1%
Chen et al., 2019 | del | 5 y | 208 | 0.00 [-0.00; 0.00] | 66.8% | 60.7%
Chen et al., 2017 | del | 60 to 108 m | 224 | -0.03 [-0.21; 0.14] | 0.0% | 0.0%
Manzano-Salgado et al., 2017 | 1st tr gest | 7 y | 551 | 0.01 [-0.08; 0.10] | 0.0% | 0.0%
Common effect model | 0.00 [-0.00; 0.00] | 100.0% | --
Random effects model | -0.00 [-0.00; 0.00] | -- | 100.0%
Heterogeneity: $I^2 = 0\%$, $\tau^2 < 0.0001$, $p = 0.54$
-0.2 -0.1 0 0.1 0.2

Study	Sampling	Outcome	Subjects	Estimate [95% CI]
Zhang, Lei, et al., 2022	within 3 d before del	7 y	108	-0.001 [-0.002; 0.001]
Bloom et al., 2022	8-13 w gest	4-8 y	341	-0.014 [-0.046; 0.018]
Liu et al., 2020	del	12 y	96	-0.040 [-0.114; 0.034]
Andersen et al., 2013	1st tr gest, 2nd tr gest, del	7 y	400	-0.030 [-0.072; 0.011]
Mora et al., 2017	9.6 w gest	7.7 y	450	-0.011 [-0.045; 0.023]
Chen et al., 2019	del	5 y	208	0.000 [0.000; 0.001]
Chen et al., 2017	del	60 to 108 m	224	-0.034 [-0.207; 0.140]
Manzano-Salgado et al., 2017	1st tr gest	7 y	551	0.010 [-0.078; 0.098]
**Summary**				**-0.011 [-0.040; 0.017]**

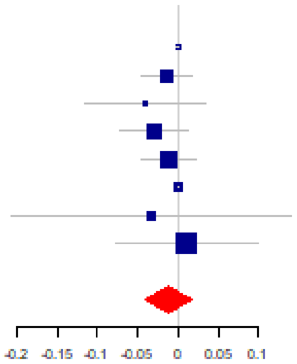

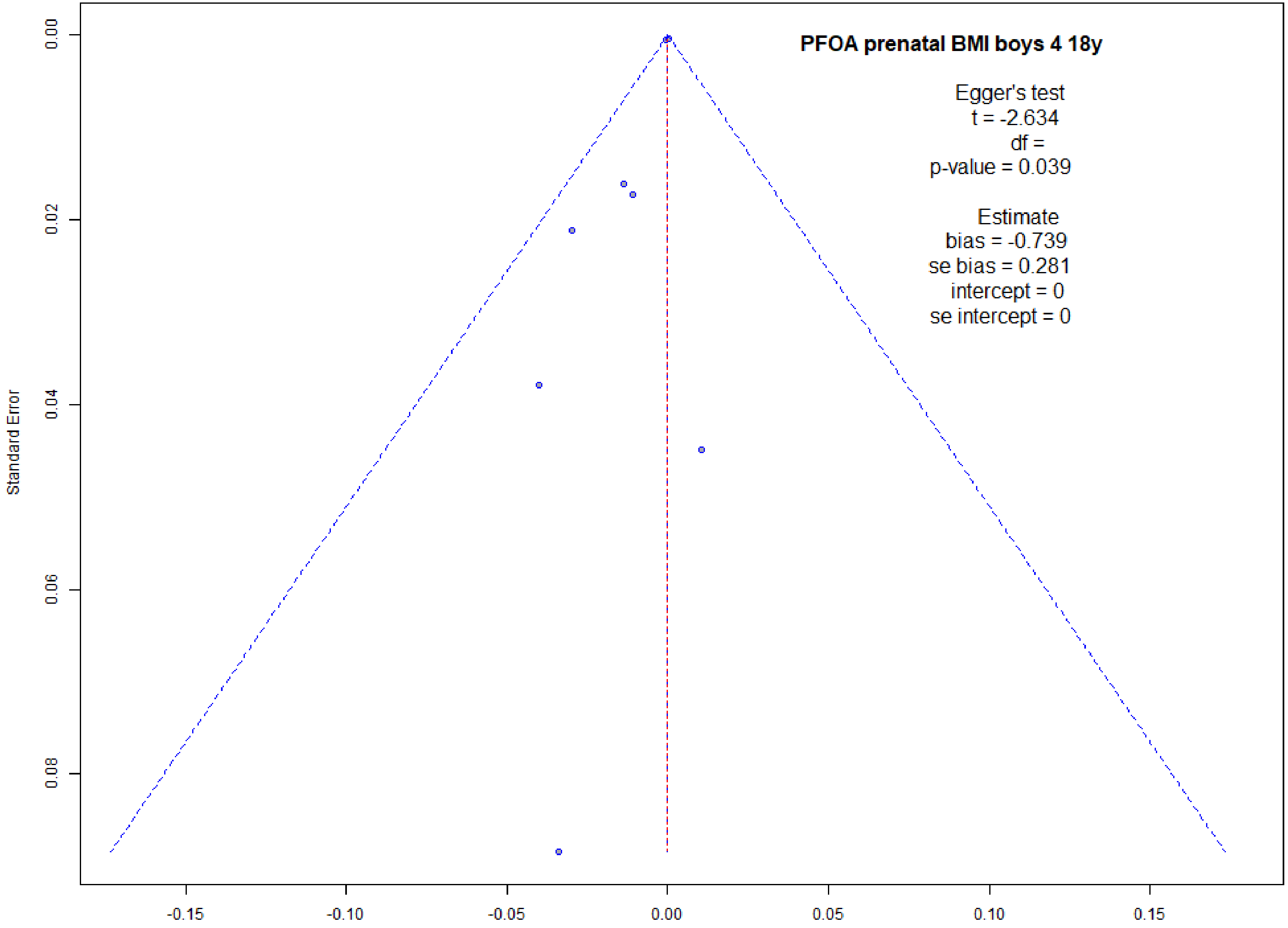
PFOA prenatal BMI boys 4 18y
Egger's test
t = -2.634
df =
p-value = 0.039
Estimate
bias = -0.739
se bias = 0.281
intercept = 0
se intercept = 0
Standard Error
0.00
0.02
0.04
0.06
0.08
-0.15
-0.10
-0.05
0.00
0.05
0.10
0.15

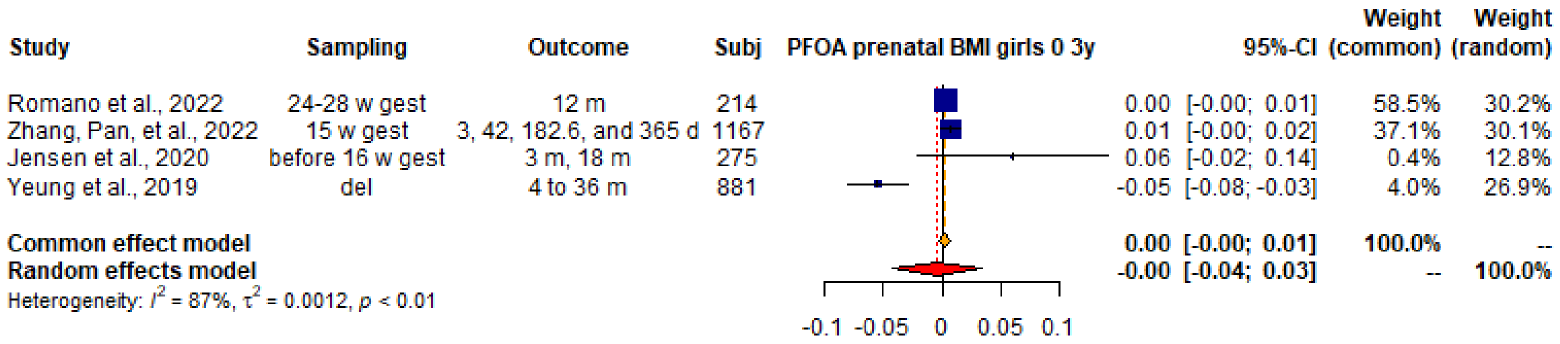
Study
Sampling
Outcome
Subj
PFOA prenatal BMI girls 0 3y
95%-CI
Weight (common)
Weight (random)
Romano et al., 2022 | 24-28 w gest | 12 m | 214 | 0.00 [-0.00; 0.01] | 58.5% | 30.2%
Zhang, Pan, et al., 2022 | 15 w gest | 3, 42, 182.6, and 365 d | 1167 | 0.01 [-0.00; 0.02] | 37.1% | 30.1%
Jensen et al., 2020 | before 16 w gest | 3 m, 18 m | 275 | 0.06 [-0.02; 0.14] | 0.4% | 12.8%
Yeung et al., 2019 | del | 4 to 36 m | 881 | -0.05 [-0.08; -0.03] | 4.0% | 26.9%
Common effect model | 0.00 [-0.00; 0.01] | 100.0% | --
Random effects model | -0.00 [-0.04; 0.03] | -- | 100.0%
Heterogeneity: I^2 = 87%, τ^2 = 0.0012, p < 0.01
-0.1
-0.05
0
0.05
0.1

Study	Sampling	Outcome	Subjects	Estimate [95% CI]
Romano et al., 2022	24-28 w gest	12 m	214	0.004 [-0.003; 0.010]
Zhang, Pan, et al., 2022	15 w gest	3, 42, 182.6, and 365 d	1167	0.007 [-0.001; 0.016]
Jensen et al., 2020	before 16 w gest	3 m, 18 m	275	0.060 [-0.020; 0.140]
Yeung et al., 2019	del	4 to 36 m	881	-0.054 [-0.080; -0.029]
**Summary**				**-0.009 [-0.031; 0.014]**

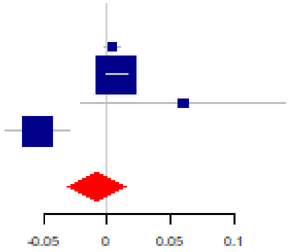

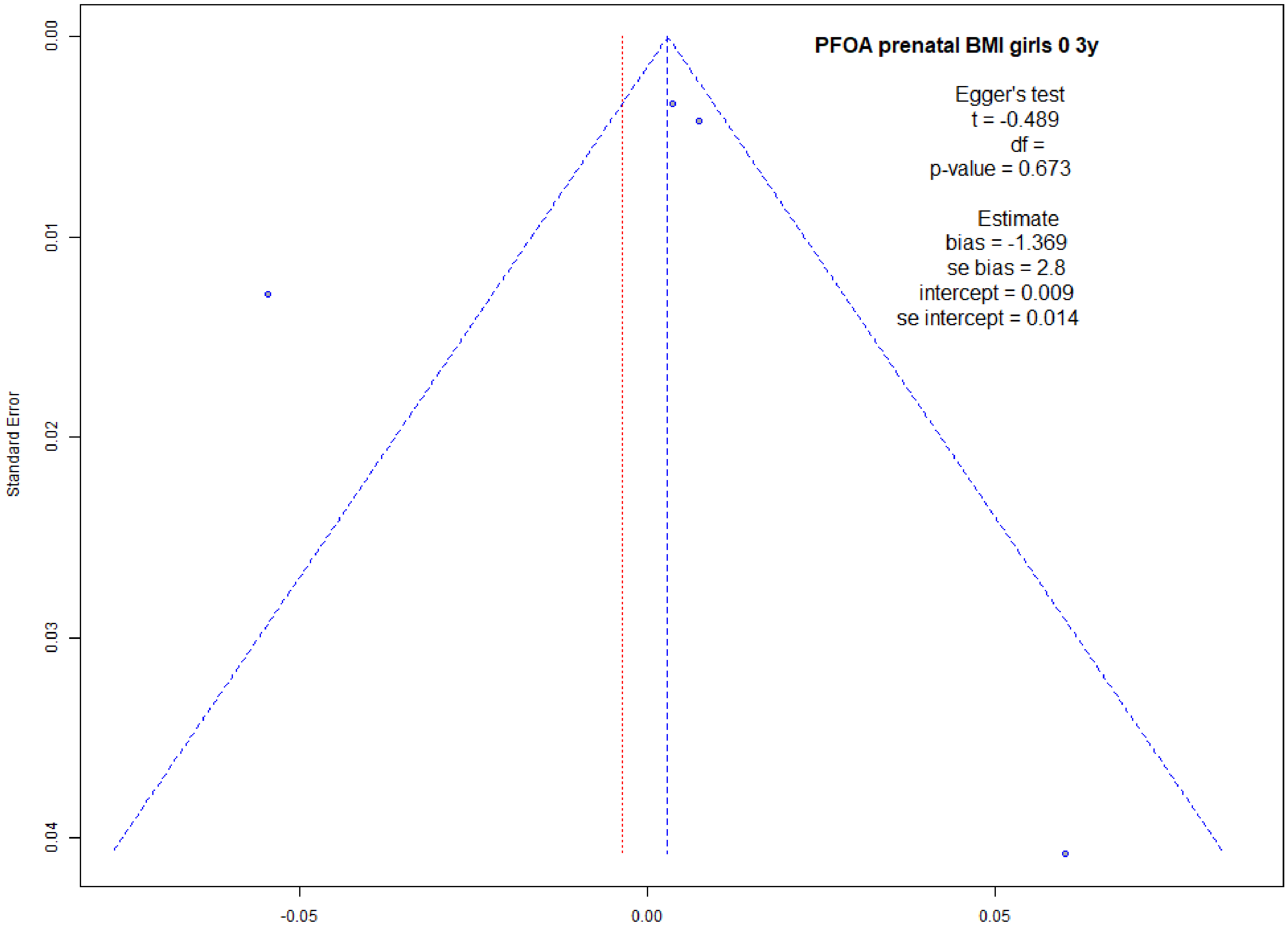
PFOA prenatal BMI girls 0 3y
Egger's test
t = -0.489
df =
p-value = 0.673
Estimate
bias = -1.369
se bias = 2.8
intercept = 0.009
se intercept = 0.014
Standard Error
0.00
0.01
0.02
0.03
0.04
-0.05
0.00
0.05

Study	Sampling	Outcome	Subj	PFOA prenatal BMI girls 4 18y	95%-CI	Weight (common)	Weight (random)
Zhang, Lei, et al., 2022	within 3 d before del	7 y	98		0.00 [-0.00; 0.00]	17.1%	17.1%
Bloom et al., 2022	8-13 w gest	4-8 y	320		-0.01 [-0.04; 0.02]	0.0%	0.0%
Hartman et al., 2017	15 w gest	9 y	312		-0.01 [-0.02; 0.00]	0.0%	0.0%
Liu et al., 2020	del	12 y	116		0.01 [-0.04; 0.07]	0.0%	0.0%
Andersen et al., 2013	1st tr gest, 2nd tr gest, del	7 y	387		-0.01 [-0.05; 0.03]	0.0%	0.0%
Mora et al., 2017	9.6 w gest	7.7 y	421		0.03 [0.00; 0.05]	0.0%	0.0%
Chen et al., 2019	del	5 y	196		0.00 [-0.00; 0.00]	82.8%	82.8%
Chen et al., 2017	del	60 to 108 m	205		0.06 [-0.05; 0.17]	0.0%	0.0%
Manzano-Salgado et al., 2017	1st tr gest	7 y	535		0.03 [-0.05; 0.10]	0.0%	0.0%
**Common effect model**					**0.00 [-0.00; 0.00]**	**100.0%**	--
**Random effects model**					**0.00 [-0.00; 0.00]**	--	**100.0%**

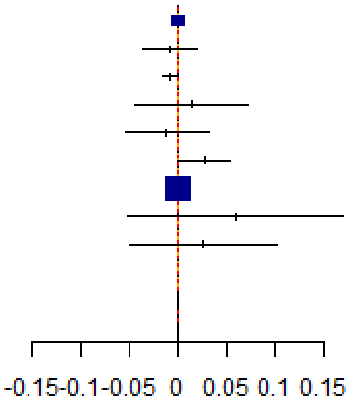

Heterogeneity: $I^2 = 23\%$, $\tau^2 = 0$, $p = 0.24$

Study	Sampling	Outcome	Subjects	Estimate [95% CI]
Zhang, Lei, et al., 2022	within 3 d before del	7 y	98	0.000 [0.000; 0.001]
Bloom et al., 2022	8-13 w gest	4-8 y	320	-0.008 [-0.036; 0.020]
Hartman et al., 2017	15 w gest	9 y	312	-0.008 [-0.015; 0.000]
Liu et al., 2020	del	12 y	116	0.013 [-0.045; 0.071]
Andersen et al., 2013	1st tr gest, 2nd tr gest, del	7 y	387	-0.011 [-0.053; 0.032]
Mora et al., 2017	9.6 w gest	7.7 y	421	0.027 [0.000; 0.054]
Chen et al., 2019	del	5 y	196	0.000 [0.000; 0.000]
Chen et al., 2017	del	60 to 108 m	205	0.059 [-0.051; 0.169]
Manzano-Salgado et al., 2017	1st tr gest	7 y	535	0.026 [-0.049; 0.101]
**Summary**				**0.011 [-0.008; 0.031]**

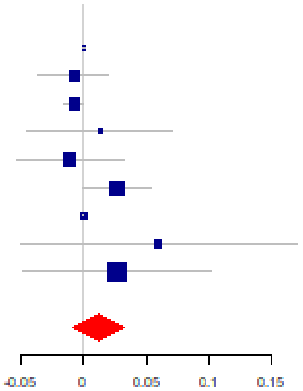

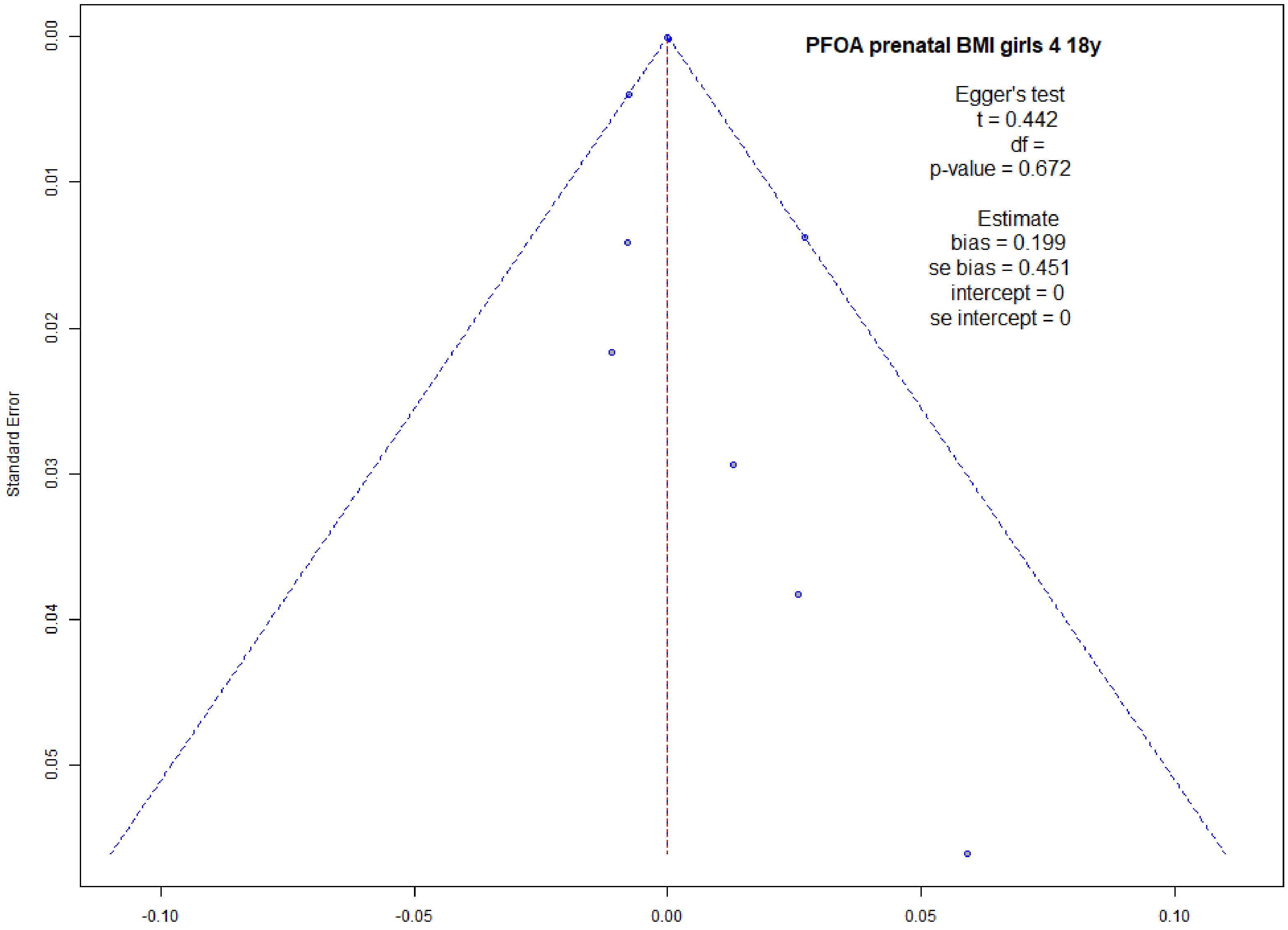
PFOA prenatal BMI girls 4 18y
Egger's test
t = 0.442
df =
p-value = 0.672
Estimate
bias = 0.199
se bias = 0.451
intercept = 0
se intercept = 0
Standard Error
0.00
0.01
0.02
0.03
0.04
0.05
-0.10
-0.05
0.00
0.05
0.10

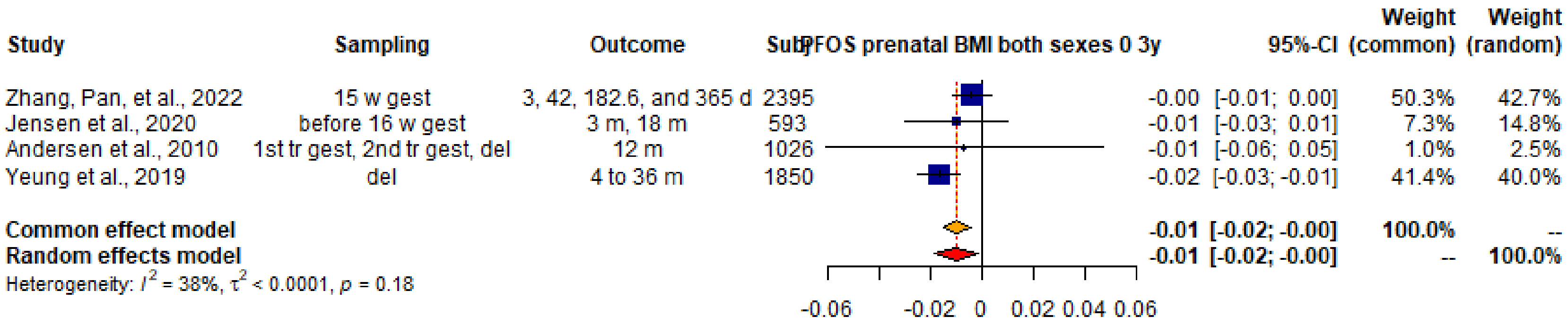
Study
Sampling
Outcome
Sub
PFOS prenatal BMI both sexes 0 3y
95%-CI
Weight (common)
Weight (random)
Zhang, Pan, et al., 2022 | 15 w gest | 3, 42, 182.6, and 365 d | 2395 | -0.00 [-0.01; 0.00] | 50.3% | 42.7%
Jensen et al., 2020 | before 16 w gest | 3 m, 18 m | 593 | -0.01 [-0.03; 0.01] | 7.3% | 14.8%
Andersen et al., 2010 | 1st tr gest, 2nd tr gest, del | 12 m | 1026 | -0.01 [-0.06; 0.05] | 1.0% | 2.5%
Yeung et al., 2019 | del | 4 to 36 m | 1850 | -0.02 [-0.03; -0.01] | 41.4% | 40.0%
Common effect model | -0.01 [-0.02; -0.00] | 100.0% | --
Random effects model | -0.01 [-0.02; -0.00] | -- | 100.0%
Heterogeneity: I^2 = 38%, τ^2 < 0.0001, p = 0.18
-0.06
-0.02
0
0.02
0.04
0.06

Study	Sampling	Outcome	Subjects	Estimate [95% CI]
Zhang, Pan, et al., 2022	15 w gest	3, 42, 182.6, and 365 d	2395	-0.004 [-0.012; 0.004]
Jensen et al., 2020	before 16 w gest	3 m, 18 m	593	-0.010 [-0.030; 0.010]
Andersen et al., 2010	1st tr gest, 2nd tr gest, del	12 m	1026	-0.007 [-0.061; 0.047]
Yeung et al., 2019	del	4 to 36 m	1850	-0.017 [-0.025; -0.008]
**Summary**				**-0.009 [-0.021; 0.003]**

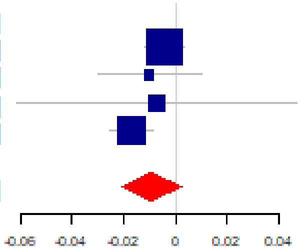

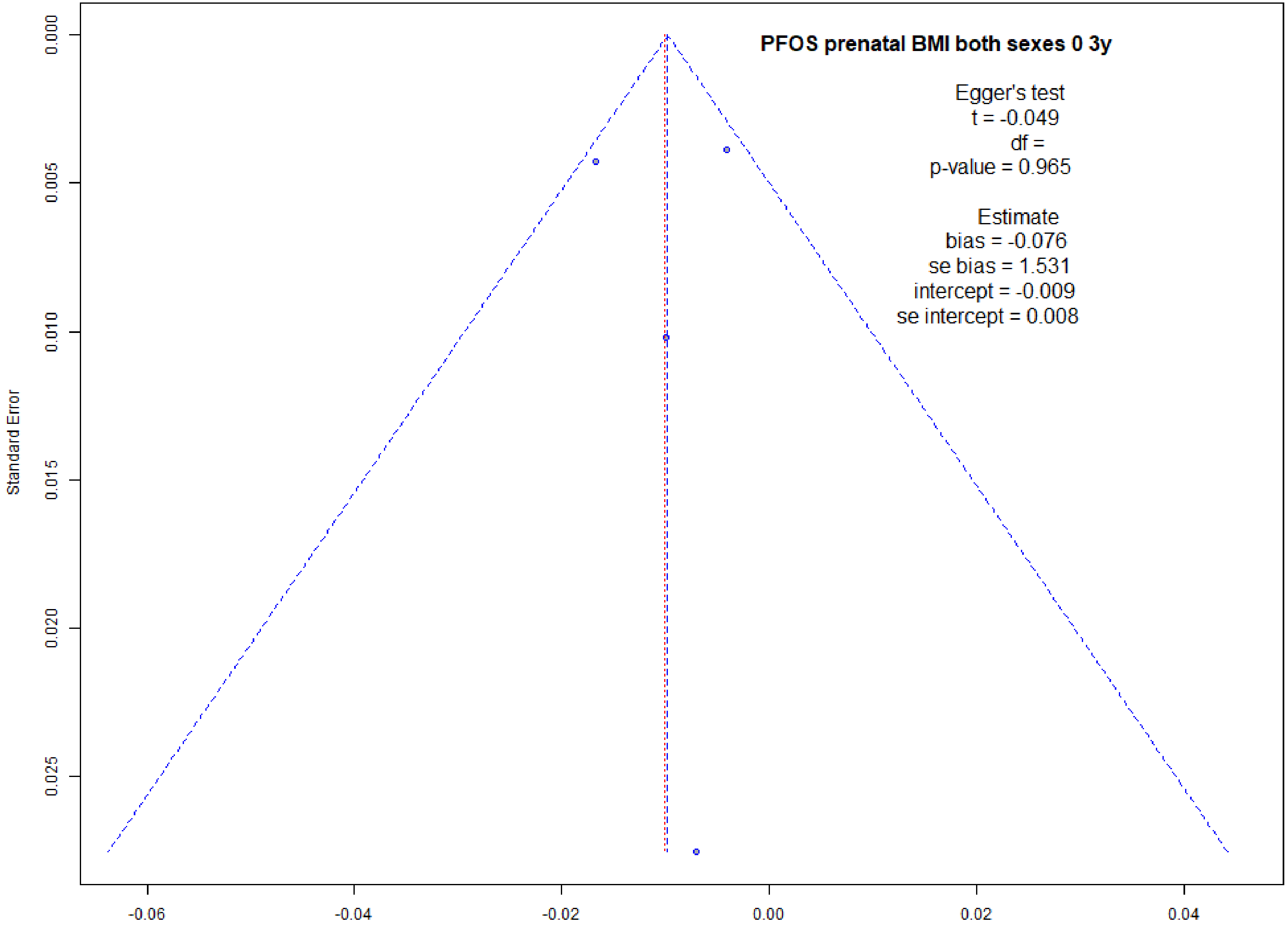
PFOS prenatal BMI both sexes 0 3y
Egger's test
t = -0.049
df =
p-value = 0.965
Estimate
bias = -0.076
se bias = 1.531
intercept = -0.009
se intercept = 0.008
Standard Error
0.000
0.005
0.010
0.015
0.020
0.025
-0.06
-0.04
-0.02
0.00
0.02
0.04

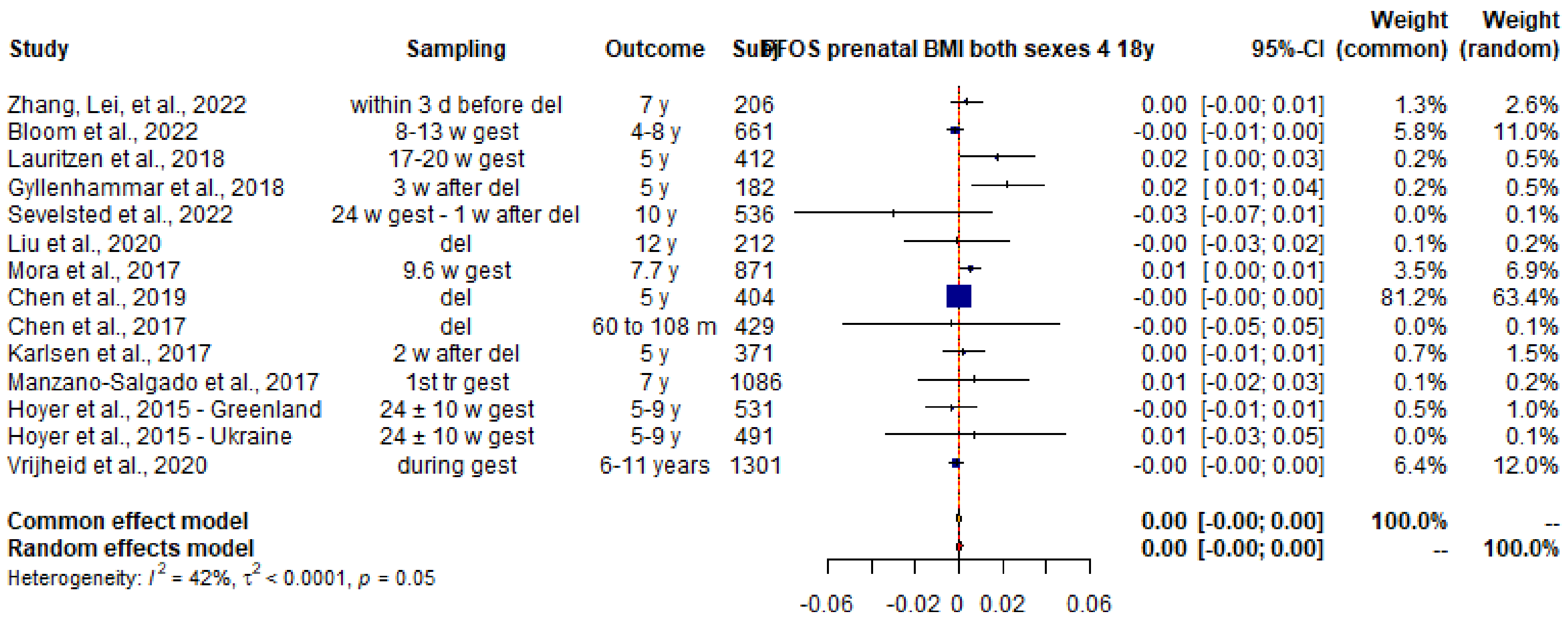
Study
Sampling
Outcome
Subj
PFOS prenatal BMI both sexes 4 18y
95%-CI
Weight (common)
Weight (random)
Zhang, Lei, et al., 2022 within 3 d before del 7 y 206 0.00 [-0.00; 0.01] 1.3% 2.6%
Bloom et al., 2022 8-13 w gest 4-8 y 661 -0.00 [-0.01; 0.00] 5.8% 11.0%
Lauritzen et al., 2018 17-20 w gest 5 y 412 0.02 [0.00; 0.03] 0.2% 0.5%
Gyllenhammar et al., 2018 3 w after del 5 y 182 0.02 [0.01; 0.04] 0.2% 0.5%
Sevelsted et al., 2022 24 w gest - 1 w after del 10 y 536 -0.03 [-0.07; 0.01] 0.0% 0.1%
Liu et al., 2020 del 12 y 212 -0.00 [-0.03; 0.02] 0.1% 0.2%
Mora et al., 2017 9.6 w gest 7.7 y 871 0.01 [0.00; 0.01] 3.5% 6.9%
Chen et al., 2019 del 5 y 404 -0.00 [-0.00; 0.00] 81.2% 63.4%
Chen et al., 2017 del 60 to 108 m 429 -0.00 [-0.05; 0.05] 0.0% 0.1%
Karlsen et al., 2017 2 w after del 5 y 371 0.00 [-0.01; 0.01] 0.7% 1.5%
Manzano-Salgado et al., 2017 1st tr gest 7 y 1086 0.01 [-0.02; 0.03] 0.1% 0.2%
Hoyer et al., 2015 - Greenland 24 ± 10 w gest 5-9 y 531 -0.00 [-0.01; 0.01] 0.5% 1.0%
Hoyer et al., 2015 - Ukraine 24 ± 10 w gest 5-9 y 491 0.01 [-0.03; 0.05] 0.0% 0.1%
Vrijheid et al., 2020 during gest 6-11 years 1301 -0.00 [-0.00; 0.00] 6.4% 12.0%
Common effect model 0.00 [-0.00; 0.00] 100.0% --
Random effects model 0.00 [-0.00; 0.00] -- 100.0%
Heterogeneity: I^2 = 42%, τ^2 < 0.0001, p = 0.05
-0.06 -0.02 0 0.02 0.06

Study	Sampling	Outcome	Subjects	Estimate [95% CI]
Zhang, Lei, et al., 2022	within 3 d before del	7 y	206	0.003 [-0.004; 0.010]
Bloom et al., 2022	8-13 w gest	4-8 y	661	-0.002 [-0.005; 0.001]
Lauritzen et al., 2018	17-20 w gest	5 y	412	0.018 [0.001; 0.035]
Gyllenhammar et al., 2018	3 w after del	5 y	182	0.022 [0.006; 0.039]
Sevelsted et al., 2022	24 w gest - 1 w after del	10 y	536	-0.030 [-0.075; 0.015]
Liu et al., 2020	del	12 y	212	-0.001 [-0.025; 0.023]
Mora et al., 2017	9.6 w gest	7.7 y	871	0.005 [0.001; 0.009]
Chen et al., 2019	del	5 y	404	0.000 [-0.001; 0.001]
Chen et al., 2017	del	60 to 108 m	429	-0.003 [-0.053; 0.046]
Karlsen et al., 2017	2 w after del	5 y	371	0.002 [-0.007; 0.011]
Manzano-Salgado et al., 2017	1st tr gest	7 y	1086	0.007 [-0.018; 0.032]
Hoyer et al., 2015 - Greenland	24 ± 10 w gest	5-9 y	531	-0.003 [-0.015; 0.008]
Hoyer et al., 2015 - Ukraine	24 ± 10 w gest	5-9 y	491	0.007 [-0.034; 0.048]
Vrijheid et al., 2020	during gest	6-11 years	1301	-0.001 [-0.004; 0.002]
**Summary**				**0.001 [-0.006; 0.007]**

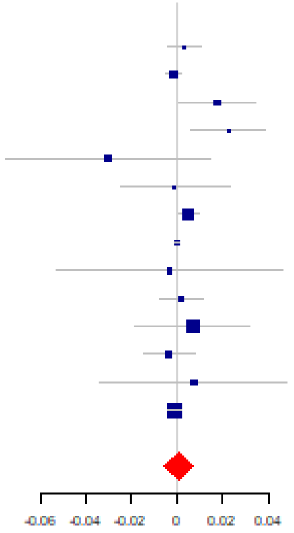

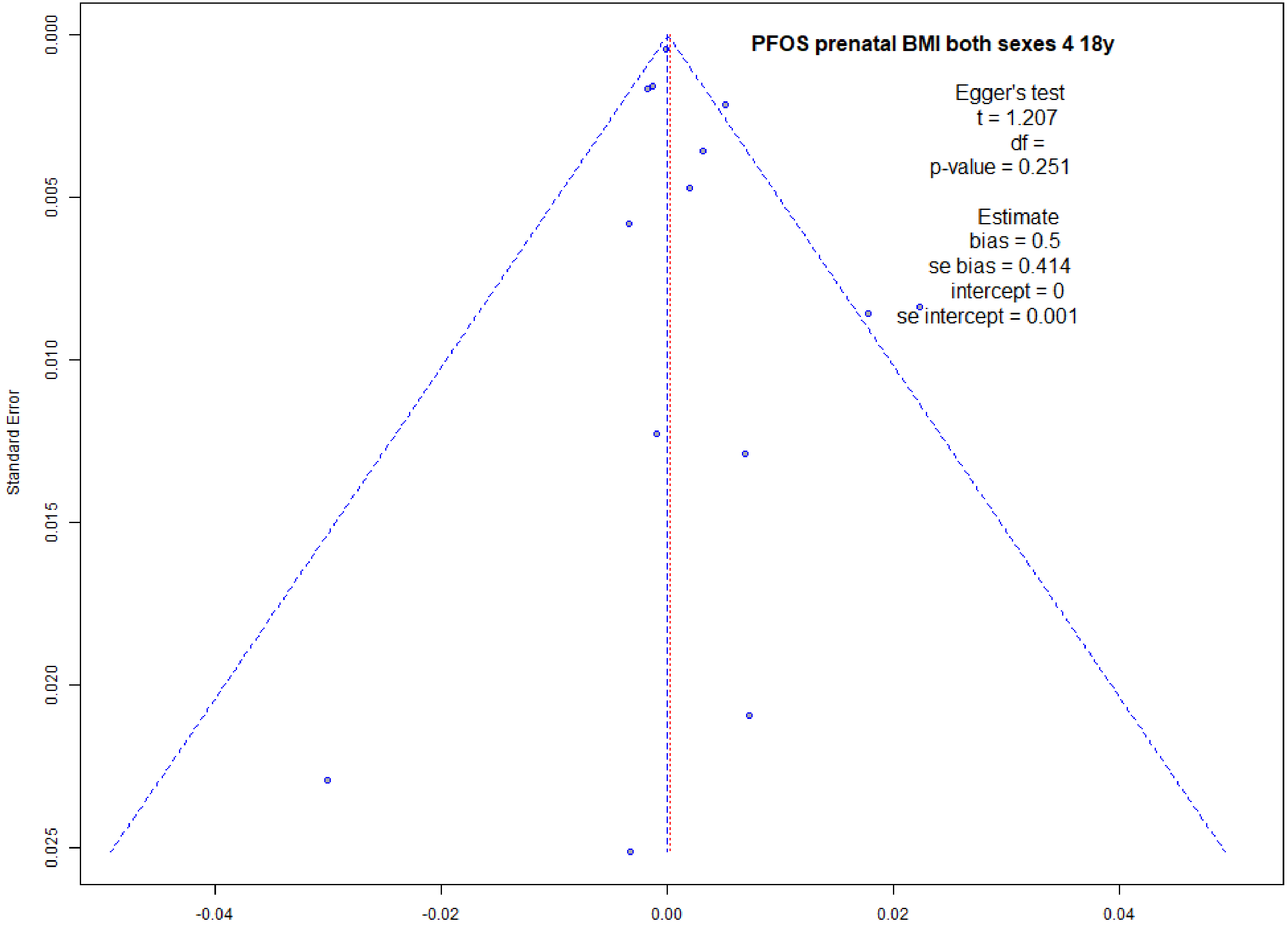
PFOS prenatal BMI both sexes 4 18y
Egger's test
t = 1.207
df =
p-value = 0.251
Estimate
bias = 0.5
se bias = 0.414
intercept = 0
se intercept = 0.001
Standard Error
0.000
0.005
0.010
0.015
0.020
0.025
-0.04
-0.02
0.00
0.02
0.04

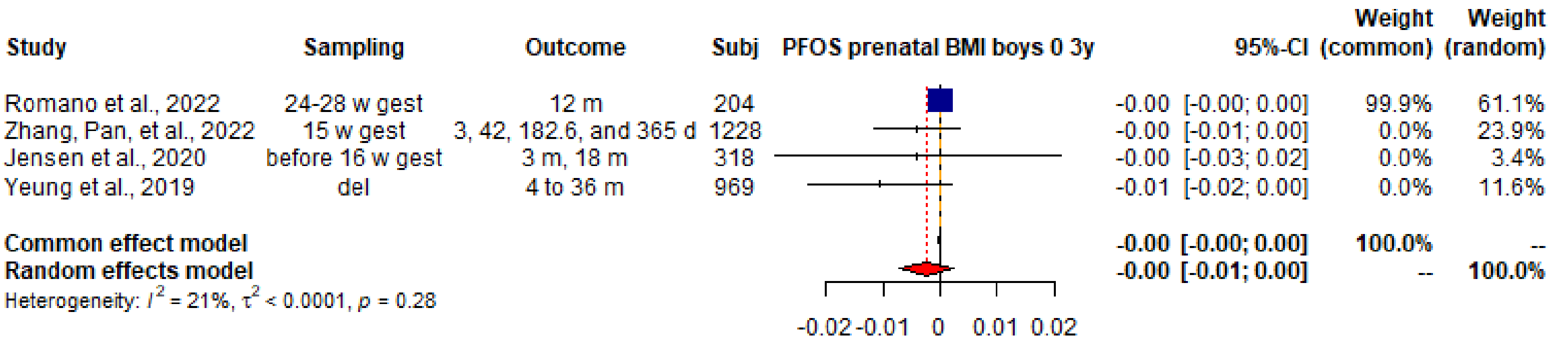
Study
Sampling
Outcome
Subj
PFOS prenatal BMI boys 0 3y
95%-CI
Weight (common)
Weight (random)
Romano et al., 2022 | 24-28 w gest | 12 m | 204 | -0.00 [-0.00; 0.00] | 99.9% | 61.1%
Zhang, Pan, et al., 2022 | 15 w gest | 3, 42, 182.6, and 365 d | 1228 | -0.00 [-0.01; 0.00] | 0.0% | 23.9%
Jensen et al., 2020 | before 16 w gest | 3 m, 18 m | 318 | -0.00 [-0.03; 0.02] | 0.0% | 3.4%
Yeung et al., 2019 | del | 4 to 36 m | 969 | -0.01 [-0.02; 0.00] | 0.0% | 11.6%
Common effect model | -0.00 [-0.00; 0.00] | 100.0% | --
Random effects model | -0.00 [-0.01; 0.00] | -- | 100.0%
Heterogeneity: I^2 = 21%, τ^2 < 0.0001, p = 0.28
-0.02 -0.01 0 0.01 0.02

Study	Sampling	Outcome	Subjects	Estimate [95% CI]
Romano et al., 2022	24-28 w gest	12 m	204	0.000 [0.000; 0.000]
Zhang, Pan, et al., 2022	15 w gest	3, 42, 182.6, and 365 d	1228	-0.004 [-0.012; 0.004]
Jensen et al., 2020	before 16 w gest	3 m, 18 m	318	-0.004 [-0.029; 0.021]
Yeung et al., 2019	del	4 to 36 m	969	-0.010 [-0.023; 0.002]
**Summary**				**-0.006 [-0.013; 0.001]**

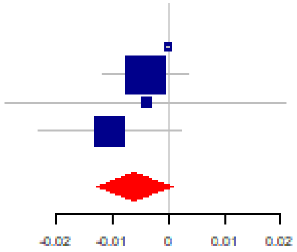

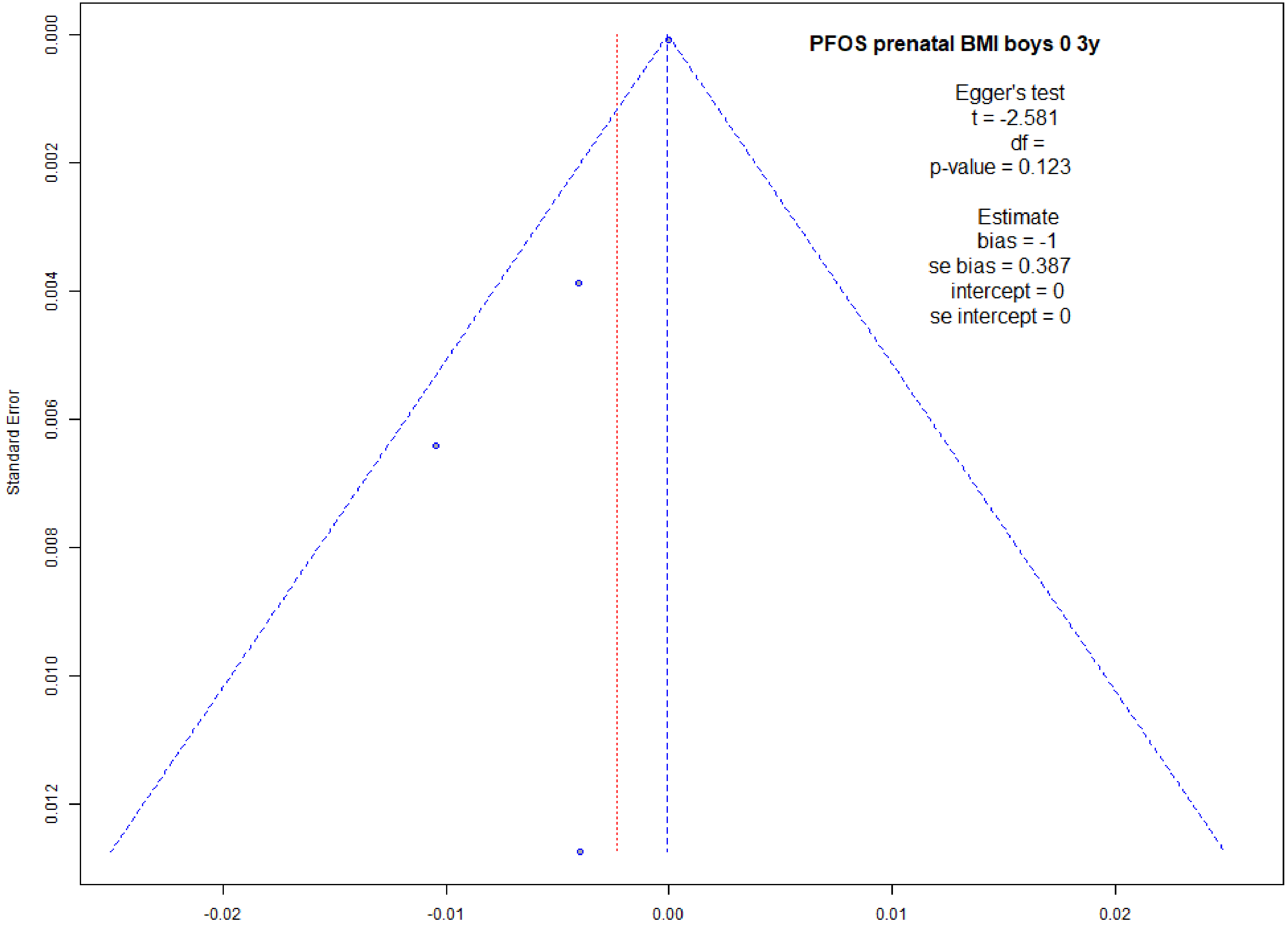
PFOS prenatal BMI boys 0 3y
Egger's test
t = -2.581
df =
p-value = 0.123
Estimate
bias = -1
se bias = 0.387
intercept = 0
se intercept = 0
Standard Error
0.000
0.002
0.004
0.006
0.008
0.010
0.012
-0.02
-0.01
0.00
0.01
0.02

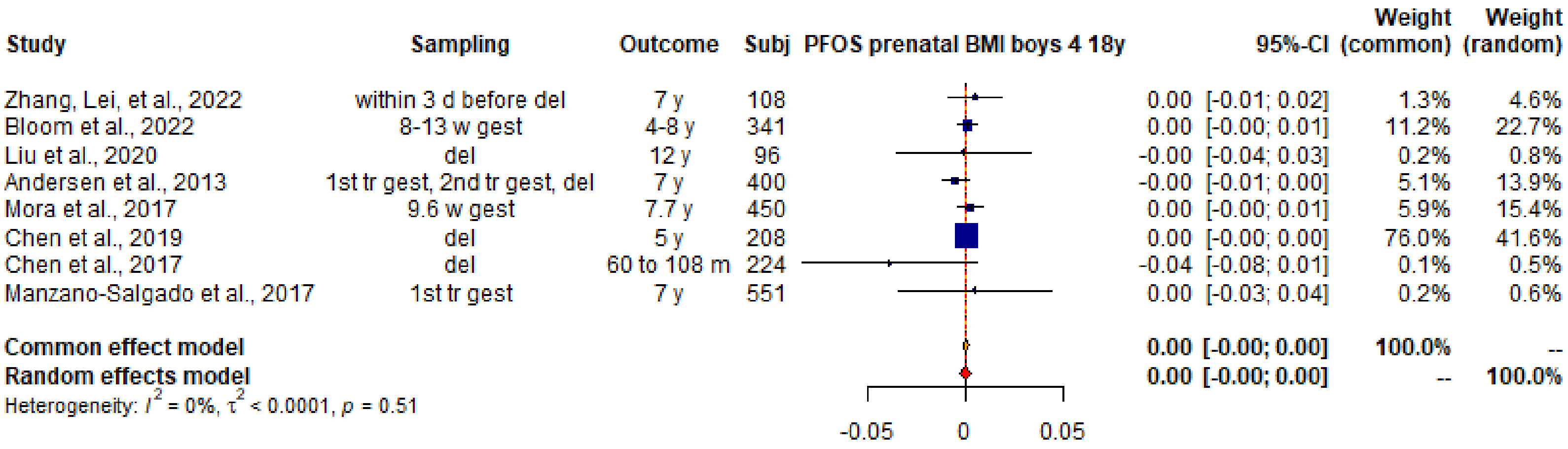
Study
Sampling
Outcome
Subj
PFOS prenatal BMI boys 4 18y
95%-CI
Weight (common)
Weight (random)
Zhang, Lei, et al., 2022 | within 3 d before del | 7 y | 108 | 0.00 [-0.01; 0.02] | 1.3% | 4.6%
Bloom et al., 2022 | 8-13 w gest | 4-8 y | 341 | 0.00 [-0.00; 0.01] | 11.2% | 22.7%
Liu et al., 2020 | del | 12 y | 96 | -0.00 [-0.04; 0.03] | 0.2% | 0.8%
Andersen et al., 2013 | 1st tr gest, 2nd tr gest, del | 7 y | 400 | -0.00 [-0.01; 0.00] | 5.1% | 13.9%
Mora et al., 2017 | 9.6 w gest | 7.7 y | 450 | 0.00 [-0.00; 0.01] | 5.9% | 15.4%
Chen et al., 2019 | del | 5 y | 208 | 0.00 [-0.00; 0.00] | 76.0% | 41.6%
Chen et al., 2017 | del | 60 to 108 m | 224 | -0.04 [-0.08; 0.01] | 0.1% | 0.5%
Manzano-Salgado et al., 2017 | 1st tr gest | 7 y | 551 | 0.00 [-0.03; 0.04] | 0.2% | 0.6%
Common effect model | 0.00 [-0.00; 0.00] | 100.0% | --
Random effects model | 0.00 [-0.00; 0.00] | -- | 100.0%
Heterogeneity: I^2 = 0%, τ^2 < 0.0001, p = 0.51
-0.05
0
0.05

Study	Sampling	Outcome	Subjects	Estimate [95% CI]
Zhang, Lei, et al., 2022	within 3 d before del	7 y	108	0.005 [-0.009; 0.019]
Bloom et al., 2022	8-13 w gest	4-8 y	341	0.001 [-0.004; 0.006]
Liu et al., 2020	del	12 y	96	-0.001 [-0.035; 0.033]
Andersen et al., 2013	1st tr gest, 2nd tr gest, del	7 y	400	-0.005 [-0.012; 0.002]
Mora et al., 2017	9.6 w gest	7.7 y	450	0.003 [-0.004; 0.009]
Chen et al., 2019	del	5 y	208	0.000 [-0.001; 0.002]
Chen et al., 2017	del	60 to 108 m	224	-0.039 [-0.083; 0.006]
Manzano-Salgado et al., 2017	1st tr gest	7 y	551	0.005 [-0.034; 0.044]
**Summary**				**-0.003 [-0.013; 0.008]**

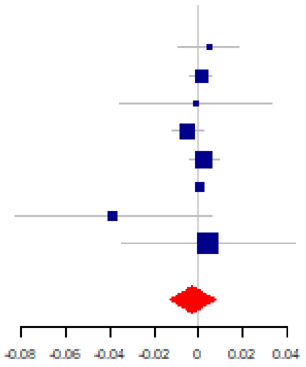

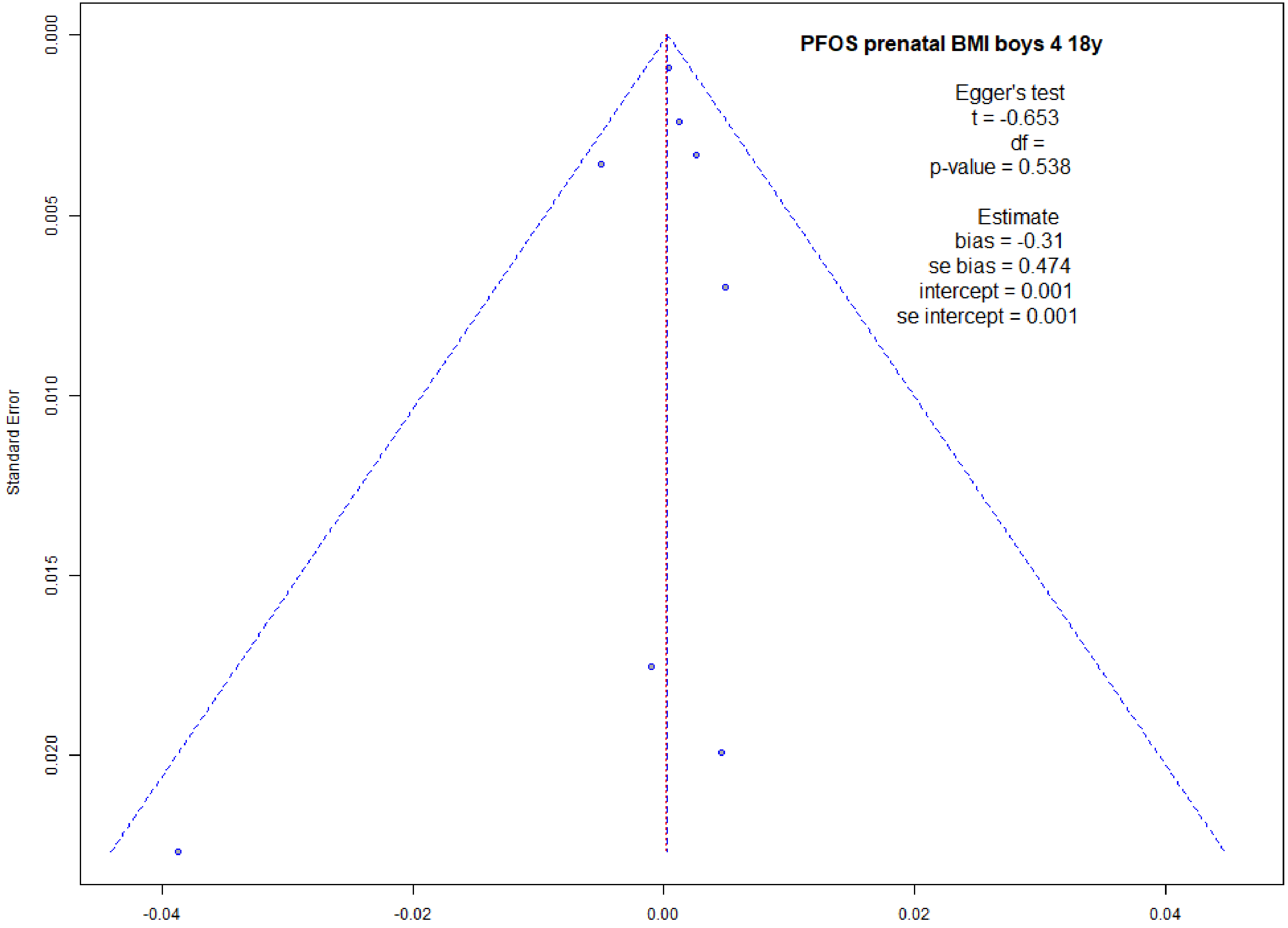
PFOS prenatal BMI boys 4 18y
Egger's test
t = -0.653
df =
p-value = 0.538
Estimate
bias = -0.31
se bias = 0.474
intercept = 0.001
se intercept = 0.001
Standard Error
0.000
0.005
0.010
0.015
0.020
-0.04
-0.02
0.00
0.02
0.04

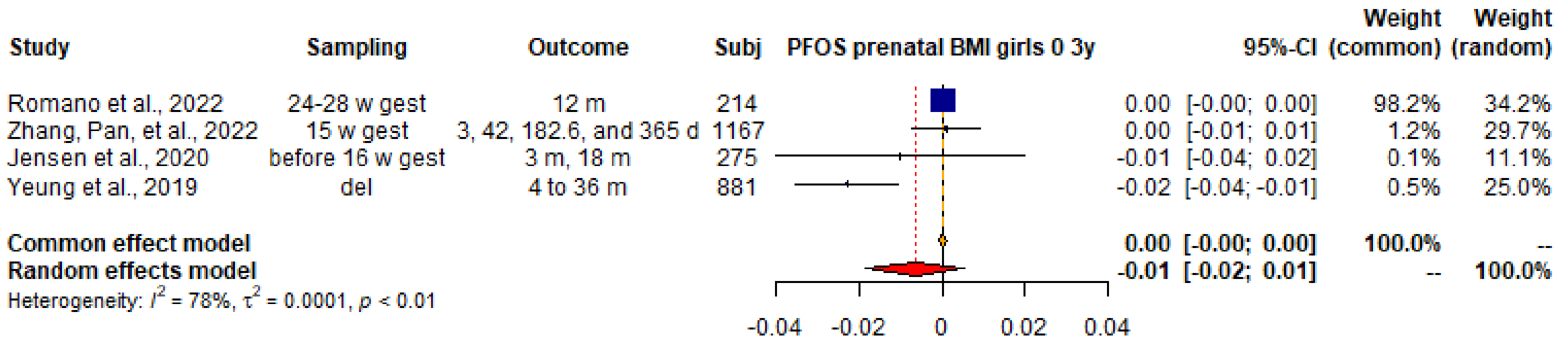
Study
Sampling
Outcome
Subj
PFOS prenatal BMI girls 0 3y
95%-CI
Weight (common)
Weight (random)
Romano et al., 2022 | 24-28 w gest | 12 m | 214 | 0.00 [-0.00; 0.00] | 98.2% | 34.2%
Zhang, Pan, et al., 2022 | 15 w gest | 3, 42, 182.6, and 365 d | 1167 | 0.00 [-0.01; 0.01] | 1.2% | 29.7%
Jensen et al., 2020 | before 16 w gest | 3 m, 18 m | 275 | -0.01 [-0.04; 0.02] | 0.1% | 11.1%
Yeung et al., 2019 | del | 4 to 36 m | 881 | -0.02 [-0.04; -0.01] | 0.5% | 25.0%
Common effect model | 0.00 [-0.00; 0.00] | 100.0% | --
Random effects model | -0.01 [-0.02; 0.01] | -- | 100.0%
Heterogeneity: I^2 = 78%, τ^2 = 0.0001, p < 0.01
-0.04
-0.02
0
0.02
0.04

Study	Sampling	Outcome	Subjects	Estimate [95% CI]
Romano et al., 2022	24-28 w gest	12 m	214	0.000 [-0.001; 0.001]
Zhang, Pan, et al., 2022	15 w gest	3, 42, 182.6, and 365 d	1167	0.001 [-0.007; 0.009]
Jensen et al., 2020	before 16 w gest	3 m, 18 m	275	-0.010 [-0.040; 0.020]
Yeung et al., 2019	del	4 to 36 m	881	-0.023 [-0.036; -0.010]
**Summary**				**-0.009 [-0.020; 0.003]**

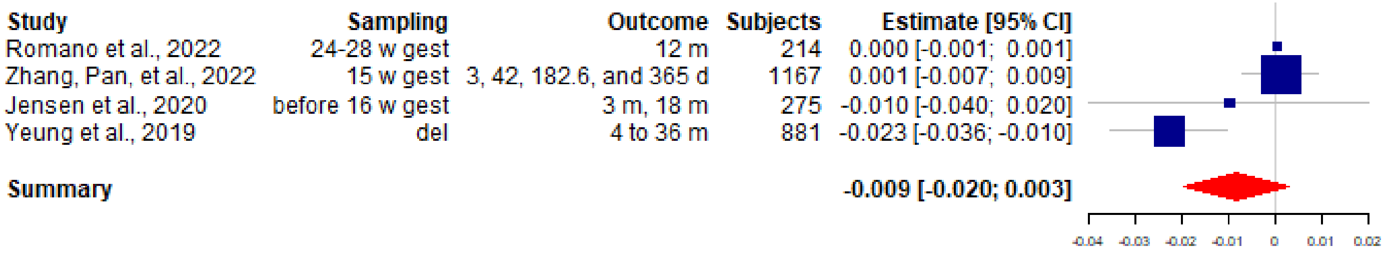

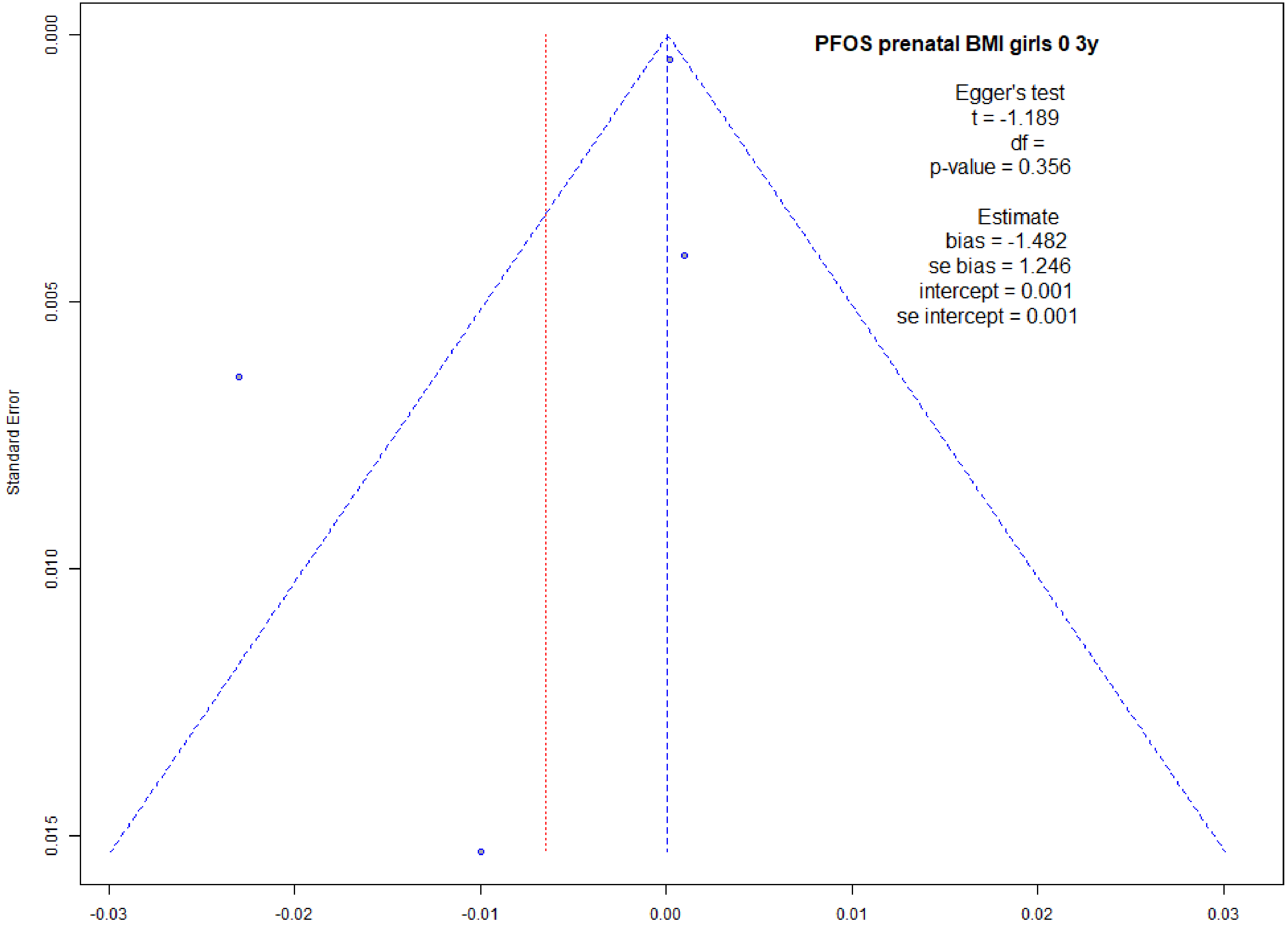
PFOS prenatal BMI girls 0 3y
Egger's test
t = -1.189
df =
p-value = 0.356
Estimate
bias = -1.482
se bias = 1.246
intercept = 0.001
se intercept = 0.001
Standard Error
0.000
0.005
0.010
0.015
-0.03
-0.02
-0.01
0.00
0.01
0.02
0.03

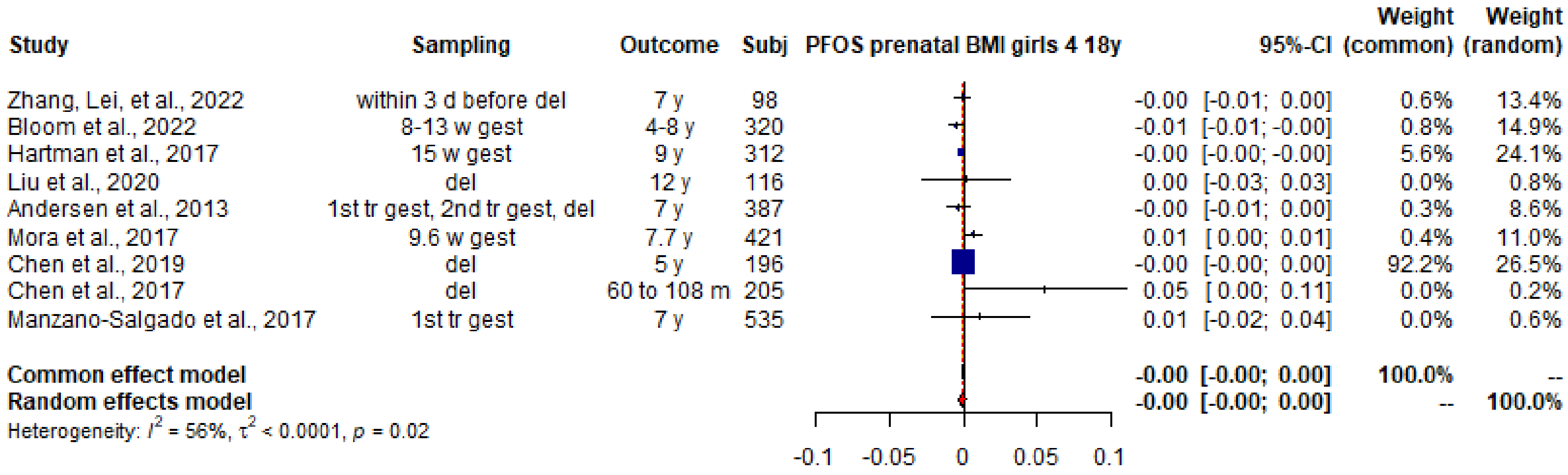
Study
Sampling
Outcome
Subj
PFOS prenatal BMI girls 4 18y
95%-CI
Weight (common)
Weight (random)
Zhang, Lei, et al., 2022 | within 3 d before del | 7 y | 98 | -0.00 [-0.01; 0.00] | 0.6% | 13.4%
Bloom et al., 2022 | 8-13 w gest | 4-8 y | 320 | -0.01 [-0.01; -0.00] | 0.8% | 14.9%
Hartman et al., 2017 | 15 w gest | 9 y | 312 | -0.00 [-0.00; -0.00] | 5.6% | 24.1%
Liu et al., 2020 | del | 12 y | 116 | 0.00 [-0.03; 0.03] | 0.0% | 0.8%
Andersen et al., 2013 | 1st tr gest, 2nd tr gest, del | 7 y | 387 | -0.00 [-0.01; 0.00] | 0.3% | 8.6%
Mora et al., 2017 | 9.6 w gest | 7.7 y | 421 | 0.01 [0.00; 0.01] | 0.4% | 11.0%
Chen et al., 2019 | del | 5 y | 196 | -0.00 [-0.00; 0.00] | 92.2% | 26.5%
Chen et al., 2017 | del | 60 to 108 m | 205 | 0.05 [0.00; 0.11] | 0.0% | 0.2%
Manzano-Salgado et al., 2017 | 1st tr gest | 7 y | 535 | 0.01 [-0.02; 0.04] | 0.0% | 0.6%
Common effect model | -0.00 [-0.00; 0.00] | 100.0% | --
Random effects model | -0.00 [-0.00; 0.00] | -- | 100.0%
Heterogeneity: I^2 = 56%, τ^2 < 0.0001, p = 0.02
-0.1 -0.05 0 0.05 0.1

Study	Sampling	Outcome	Subjects	Estimate [95% CI]
Zhang, Lei, et al., 2022	within 3 d before del	7 y	98	0.000 [-0.005; 0.005]
Bloom et al., 2022	8-13 w gest	4-8 y	320	-0.005 [-0.010; 0.000]
Hartman et al., 2017	15 w gest	9 y	312	-0.002 [-0.004; 0.000]
Liu et al., 2020	del	12 y	116	0.002 [-0.028; 0.032]
Andersen et al., 2013	1st tr gest, 2nd tr gest, del	7 y	387	-0.003 [-0.010; 0.004]
Mora et al., 2017	9.6 w gest	7.7 y	421	0.006 [0.000; 0.013]
Chen et al., 2019	del	5 y	196	0.000 [0.000; 0.000]
Chen et al., 2017	del	60 to 108 m	205	0.055 [0.001; 0.109]
Manzano-Salgado et al., 2017	1st tr gest	7 y	535	0.011 [-0.022; 0.045]
**Summary**				**0.007 [-0.002; 0.015]**

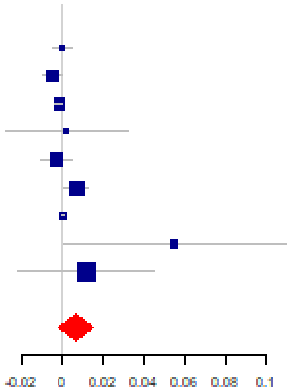

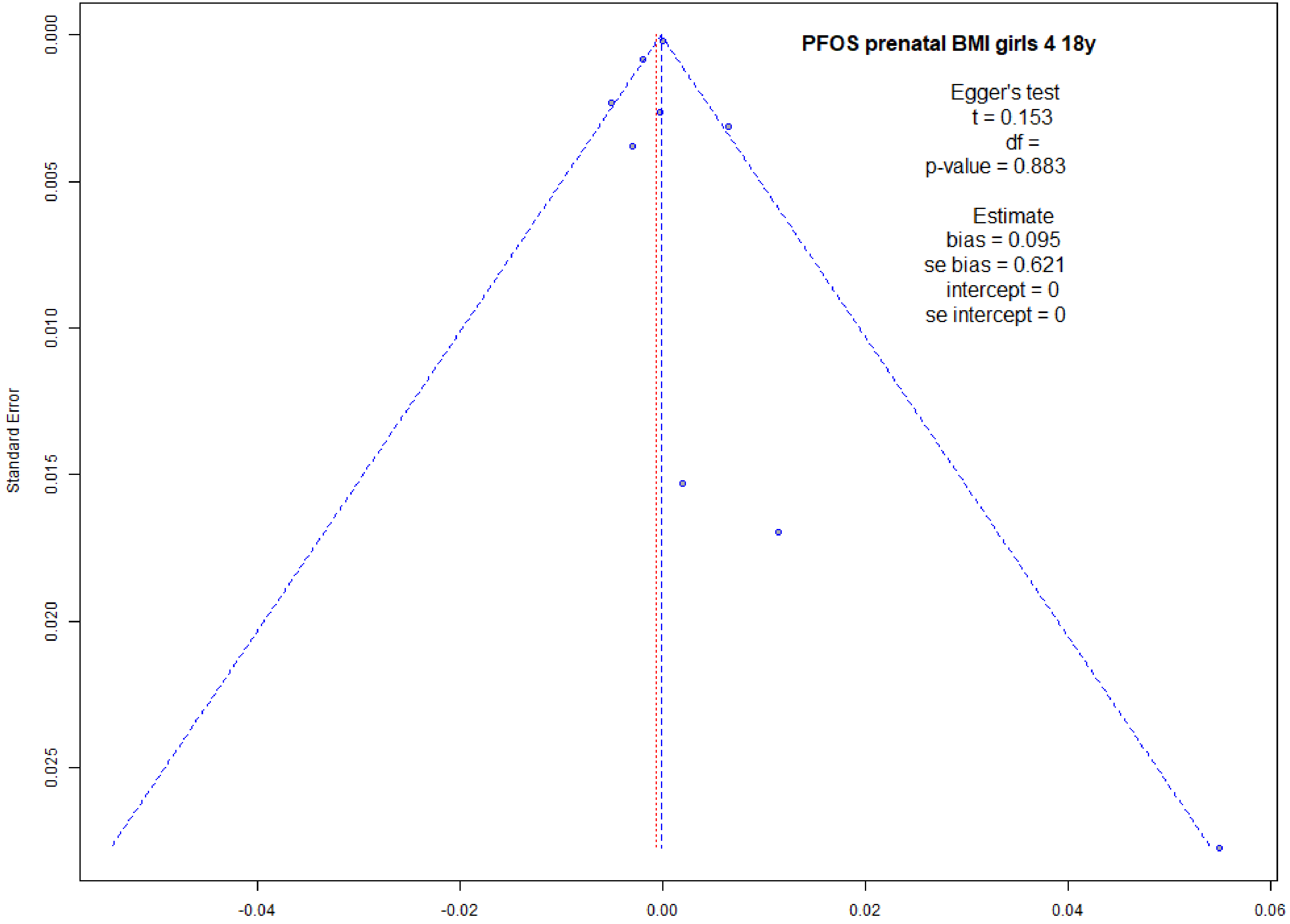
PFOS prenatal BMI girls 4 18y
Egger's test
t = 0.153
df =
p-value = 0.883
Estimate
bias = 0.095
se bias = 0.621
intercept = 0
se intercept = 0
Standard Error
0.000
0.005
0.010
0.015
0.020
0.025
-0.04
-0.02
0.00
0.02
0.04
0.06

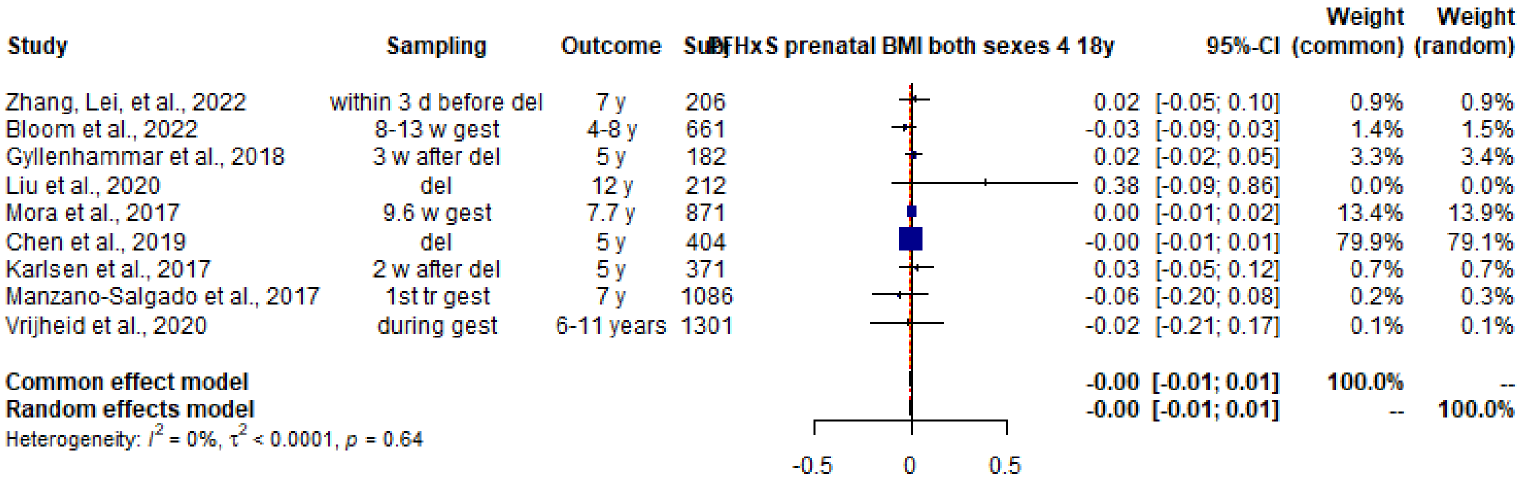
Study
Sampling
Outcome
PFHxS prenatal BMI both sexes 4 18y
95%-CI
Weight (common)
Weight (random)
Zhang, Lei, et al., 2022 within 3 d before del 7 y 206 0.02 [-0.05; 0.10] 0.9% 0.9%
Bloom et al., 2022 8-13 w gest 4-8 y 661 -0.03 [-0.09; 0.03] 1.4% 1.5%
Gyllenhammar et al., 2018 3 w after del 5 y 182 0.02 [-0.02; 0.05] 3.3% 3.4%
Liu et al., 2020 del 12 y 212 0.38 [-0.09; 0.86] 0.0% 0.0%
Mora et al., 2017 9.6 w gest 7.7 y 871 0.00 [-0.01; 0.02] 13.4% 13.9%
Chen et al., 2019 del 5 y 404 -0.00 [-0.01; 0.01] 79.9% 79.1%
Karlsen et al., 2017 2 w after del 5 y 371 0.03 [-0.05; 0.12] 0.7% 0.7%
Manzano-Salgado et al., 2017 1st tr gest 7 y 1086 -0.06 [-0.20; 0.08] 0.2% 0.3%
Vrijheid et al., 2020 during gest 6-11 years 1301 -0.02 [-0.21; 0.17] 0.1% 0.1%
Common effect model -0.00 [-0.01; 0.01] 100.0% --
Random effects model -0.00 [-0.01; 0.01] -- 100.0%
Heterogeneity: I^2 = 0%, τ^2 < 0.0001, p = 0.64
-0.5
0
0.5

Study	Sampling	Outcome	Subjects	Estimate [95% CI]
Zhang, Lei, et al., 2022	within 3 d before del	7 y	206	0.021 [-0.055; 0.096]
Bloom et al., 2022	8-13 w gest	4-8 y	661	-0.032 [-0.092; 0.027]
Gyllenhammar et al., 2018	3 w after del	5 y	182	0.015 [-0.023; 0.054]
Liu et al., 2020	del	12 y	212	0.384 [-0.091; 0.859]
Mora et al., 2017	9.6 w gest	7.7 y	871	0.005 [-0.014; 0.024]
Chen et al., 2019	del	5 y	404	-0.001 [-0.009; 0.006]
Karlsen et al., 2017	2 w after del	5 y	371	0.031 [-0.054; 0.117]
Manzano-Salgado et al., 2017	1st tr gest	7 y	1086	-0.056 [-0.196; 0.084]
Vrijheid et al., 2020	during gest	6-11 years	1301	-0.020 [-0.211; 0.170]
**Summary**				**-0.001 [-0.060; 0.058]**

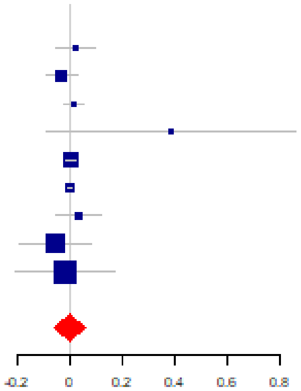

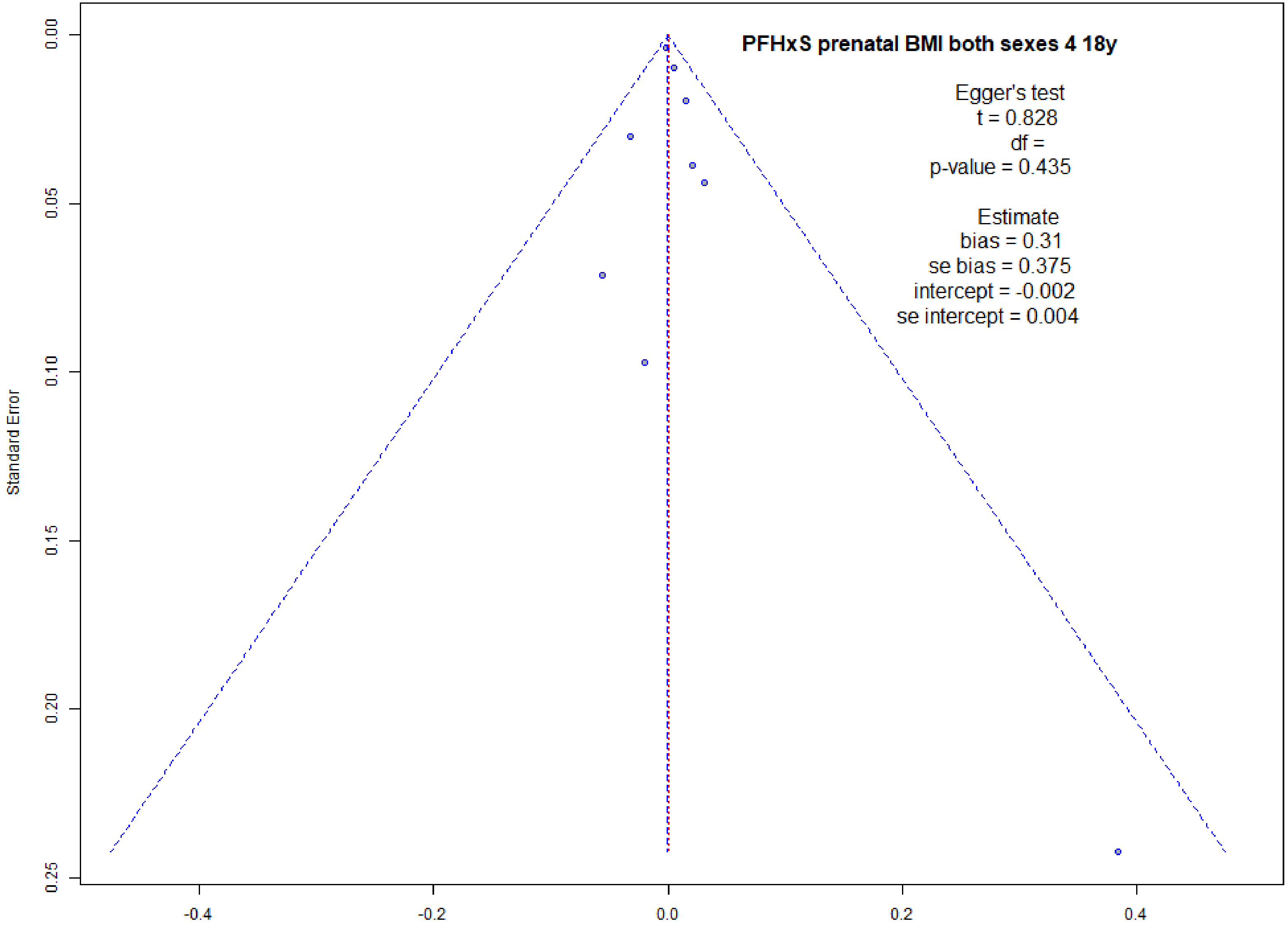
PFHxS prenatal BMI both sexes 4 18y
Egger's test
t = 0.828
df =
p-value = 0.435
Estimate
bias = 0.31
se bias = 0.375
intercept = -0.002
se intercept = 0.004
Standard Error
0.00
0.05
0.10
0.15
0.20
0.25
-0.4
-0.2
0.0
0.2
0.4

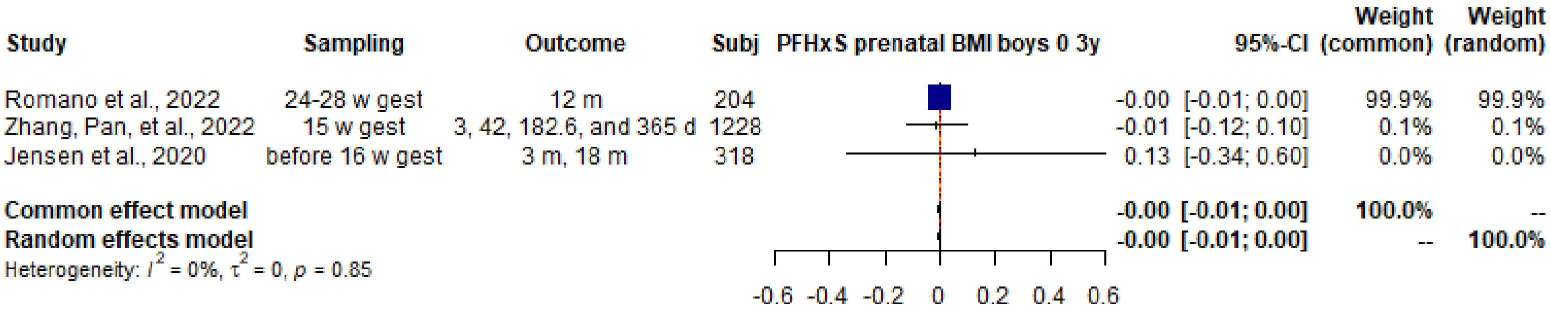
Study
Sampling
Outcome
Subj
PFHxS prenatal BMI boys 0 3y
95%-CI
Weight (common)
Weight (random)
Romano et al., 2022
24-28 w gest
12 m
204
-0.00 [-0.01; 0.00]
99.9%
99.9%
Zhang, Pan, et al., 2022
15 w gest
3, 42, 182.6, and 365 d
1228
-0.01 [-0.12; 0.10]
0.1%
0.1%
Jensen et al., 2020
before 16 w gest
3 m, 18 m
318
0.13 [-0.34; 0.60]
0.0%
0.0%
Common effect model
-0.00 [-0.01; 0.00]
100.0%
--
Random effects model
-0.00 [-0.01; 0.00]
--
100.0%
Heterogeneity: I^2 = 0%, τ^2 = 0, p = 0.85
-0.6 -0.4 -0.2 0 0.2 0.4 0.6

Study	Sampling	Outcome	Subjects	Estimate [95% CI]
Romano et al., 2022	24-28 w gest	12 m	204	-0.002 [-0.005; 0.001]
Zhang, Pan, et al., 2022	15 w gest	3, 42, 182.6, and 365 d	1228	-0.011 [-0.122; 0.101]
Jensen et al., 2020	before 16 w gest	3 m, 18 m	318	0.130 [-0.340; 0.600]
**Summary**				**0.016 [-0.100; 0.132]**

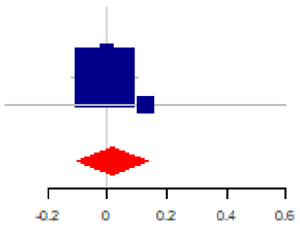

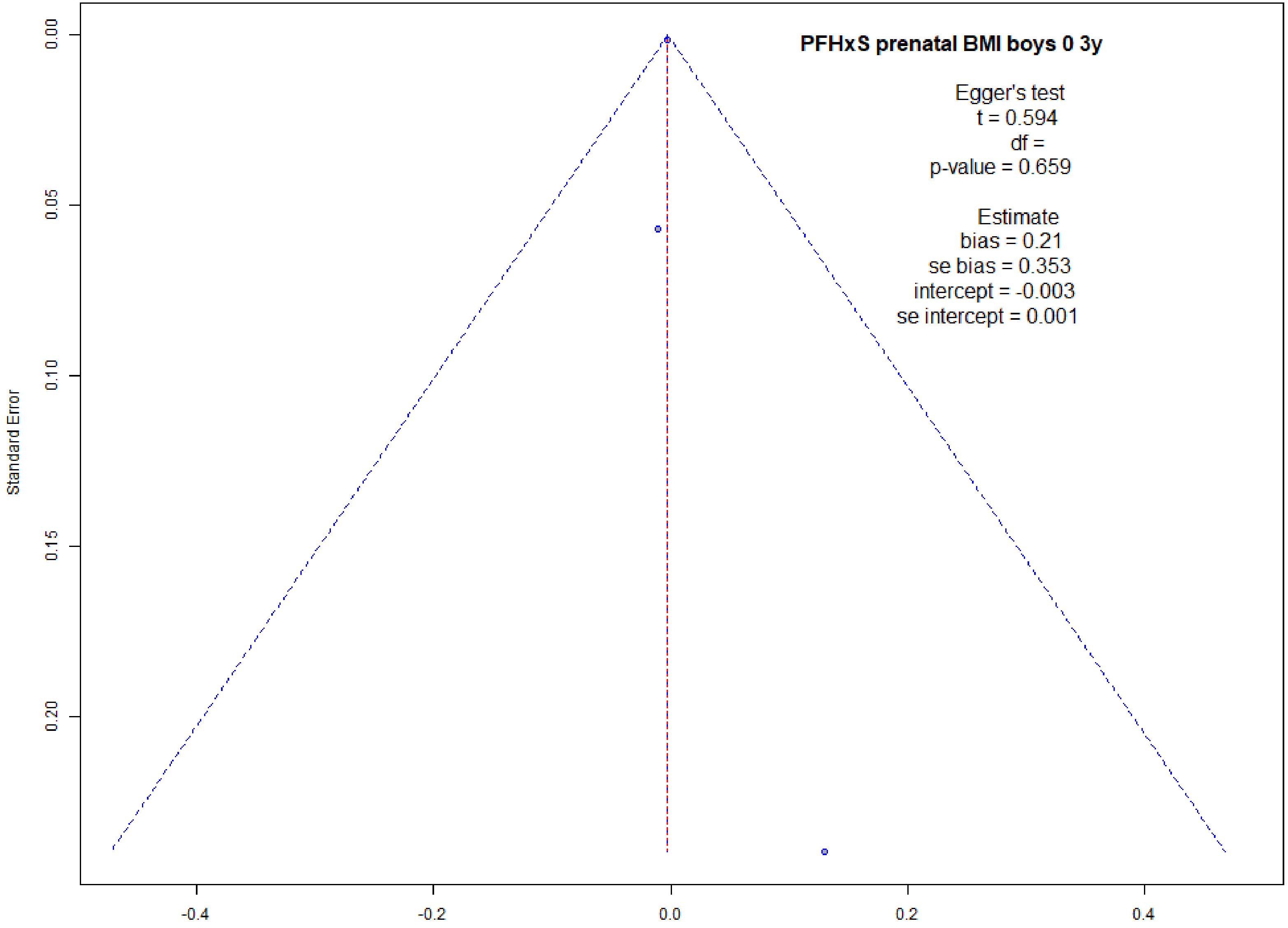
PFHxS prenatal BMI boys 0 3y
Egger's test
t = 0.594
df =
p-value = 0.659
Estimate
bias = 0.21
se bias = 0.353
intercept = -0.003
se intercept = 0.001
Standard Error
0.00
0.05
0.10
0.15
0.20
-0.4
-0.2
0.0
0.2
0.4

Study	Sampling	Outcome	Subj	95%-CI	Weight (common)	Weight (random)
Zhang, Lei, et al., 2022	within 3 d before del	7 y	108	0.01 [-0.14; 0.17]	0.9%	0.9%
Bloom et al., 2022	8-13 w gest	4-8 y	341	-0.02 [-0.11; 0.06]	3.1%	3.1%
Liu et al., 2020	del	12 y	96	-0.40 [-1.27; 0.47]	0.0%	0.0%
Mora et al., 2017	9.6 w gest	7.7 y	450	-0.00 [-0.04; 0.03]	22.8%	22.8%
Chen et al., 2019	del	5 y	208	0.01 [-0.01; 0.03]	72.6%	72.6%
Manzano-Salgado et al., 2017	1st tr gest	7 y	551	-0.14 [-0.35; 0.07]	0.5%	0.5%
**Common effect model**				**0.00 [-0.01; 0.02]**	**100.0%**	--
**Random effects model**				**0.00 [-0.01; 0.02]**	--	**100.0%**

Heterogeneity: $I^2 = 0\%$, $\tau^2 = 0$, $p = 0.61$

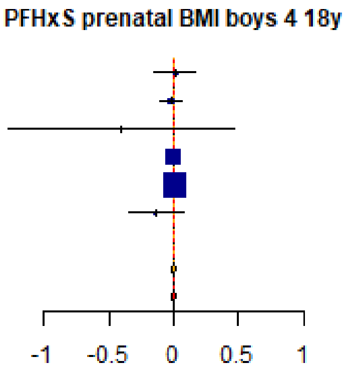

Gianfranco Frigerio, Chiara Matilde Ferrari, and Silvia Fustinoni

Study	Sampling	Outcome	Subjects	Estimate [95% CI]
Zhang, Lei, et al., 2022	within 3 d before del	7 y	108	0.014 [-0.143; 0.172]
Bloom et al., 2022	8-13 w gest	4-8 y	341	-0.022 [-0.105; 0.062]
Liu et al., 2020	del	12 y	96	-0.401 [-1.274; 0.472]
Mora et al., 2017	9.6 w gest	7.7 y	450	-0.005 [-0.036; 0.026]
Chen et al., 2019	del	5 y	208	0.009 [-0.008; 0.026]
Manzano-Salgado et al., 2017	1st tr gest	7 y	551	-0.140 [-0.350; 0.070]
**Summary**				**-0.069 [-0.153; 0.015]**

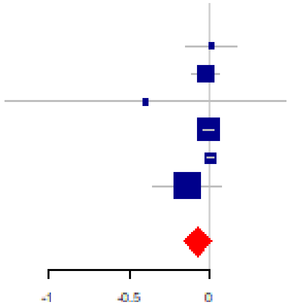

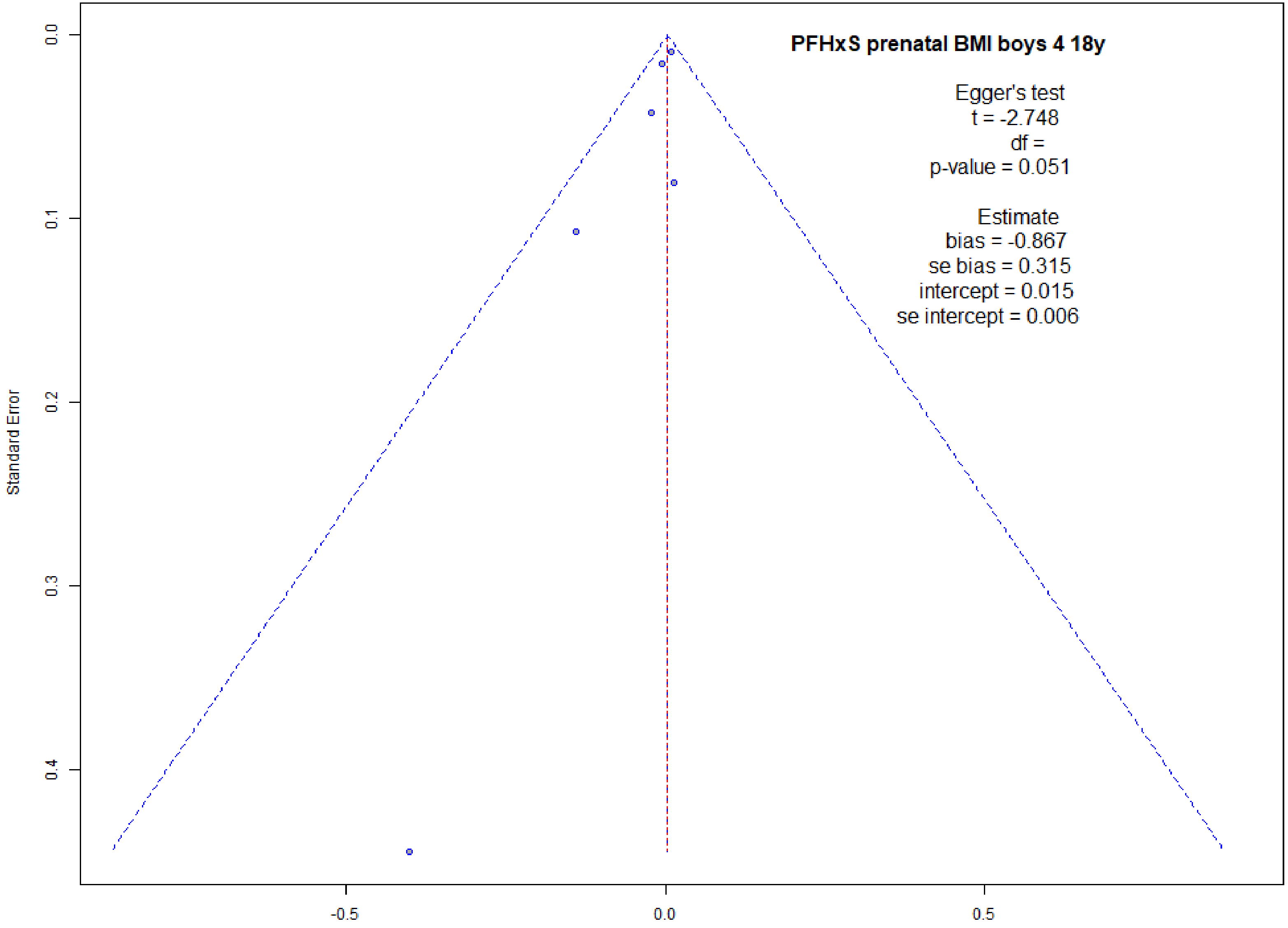
PFHxS prenatal BMI boys 4 18y
Egger's test
t = -2.748
df =
p-value = 0.051
Estimate
bias = -0.867
se bias = 0.315
intercept = 0.015
se intercept = 0.006
Standard Error
0.0
0.1
0.2
0.3
0.4
-0.5
0.0
0.5

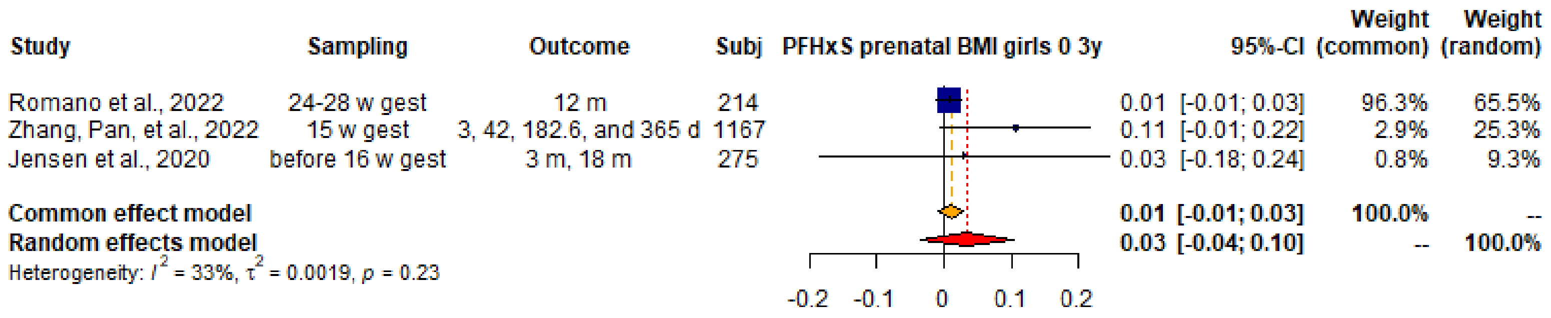
Study
Sampling
Outcome
Subj
PFHxS prenatal BMI girls 0 3y
95%-CI
Weight (common)
Weight (random)
Romano et al., 2022 | 24-28 w gest | 12 m | 214 | 0.01 [-0.01; 0.03] | 96.3% | 65.5%
Zhang, Pan, et al., 2022 | 15 w gest | 3, 42, 182.6, and 365 d | 1167 | 0.11 [-0.01; 0.22] | 2.9% | 25.3%
Jensen et al., 2020 | before 16 w gest | 3 m, 18 m | 275 | 0.03 [-0.18; 0.24] | 0.8% | 9.3%
Common effect model | 0.01 [-0.01; 0.03] | 100.0% | --
Random effects model | 0.03 [-0.04; 0.10] | -- | 100.0%
Heterogeneity: I^2 = 33%, τ^2 = 0.0019, p = 0.23
-0.2
-0.1
0
0.1
0.2

Study	Sampling	Outcome	Subjects	Estimate [95% CI]
Romano et al., 2022	24-28 w gest	12 m	214	0.007 [-0.012; 0.027]
Zhang, Pan, et al., 2022	15 w gest	3, 42, 182.6, and 365 d	1167	0.106 [-0.005; 0.217]
Jensen et al., 2020	before 16 w gest	3 m, 18 m	275	0.030 [-0.185; 0.245]
**Summary**				**0.081 [-0.020; 0.181]**

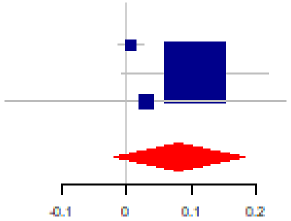

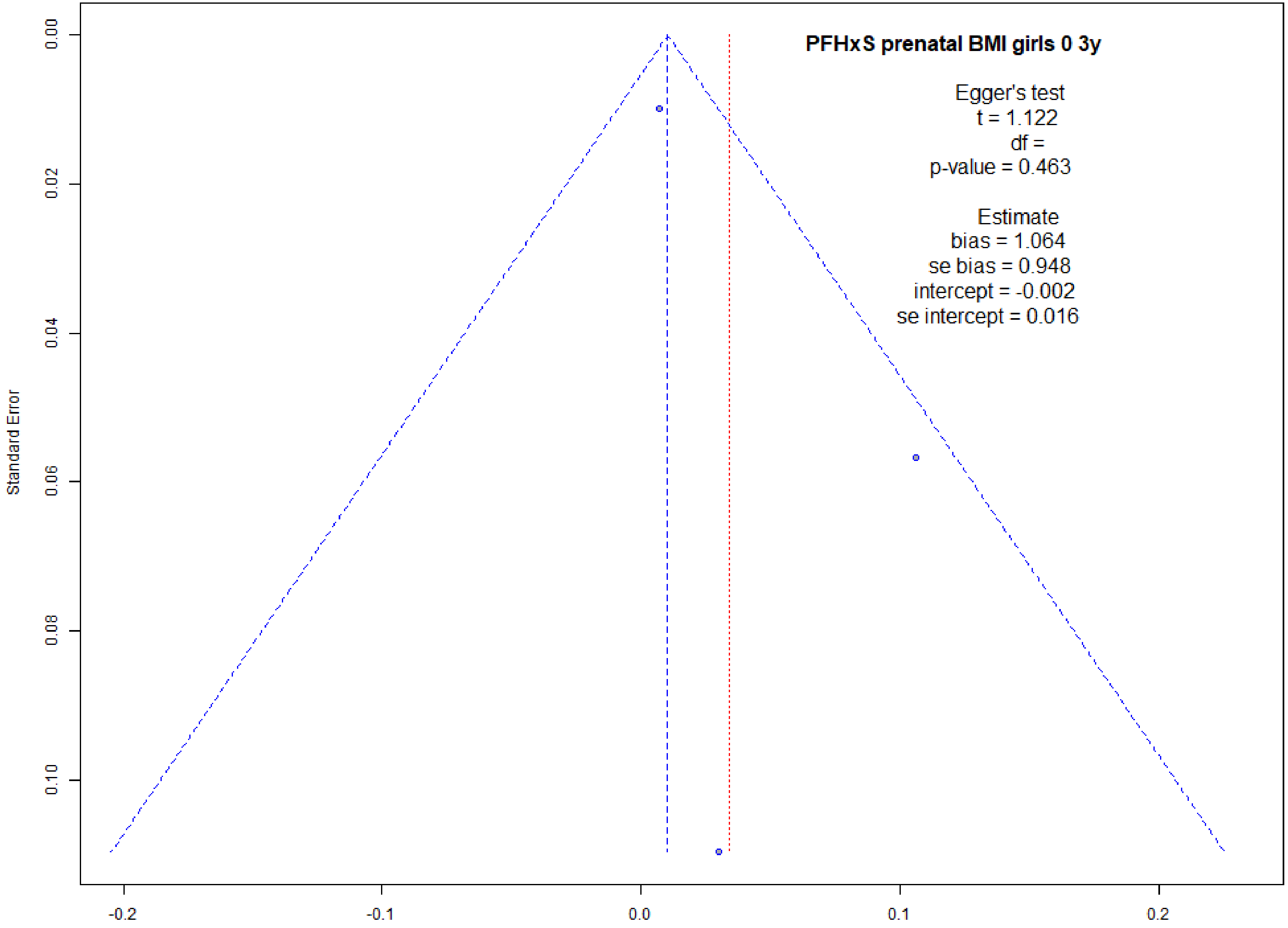
PFHxS prenatal BMI girls 0 3y
Egger's test
t = 1.122
df =
p-value = 0.463
Estimate
bias = 1.064
se bias = 0.948
intercept = -0.002
se intercept = 0.016
Standard Error
0.00
0.02
0.04
0.06
0.08
0.10
-0.2
-0.1
0.0
0.1
0.2

Study	Sampling	Outcome	Subj	PFHxS prenatal BMI girls 4 18y	95%-CI	Weight (common)	Weight (random)
Zhang, Lei, et al., 2022	within 3 d before del	7 y	98	0.02	[-0.03; 0.07]	0.2%	0.5%
Bloom et al., 2022	8-13 w gest	4-8 y	320	-0.04	[-0.12; 0.04]	0.1%	0.2%
Hartman et al., 2017	15 w gest	9 y	311	-0.00	[-0.00; 0.00]	65.2%	54.5%
Liu et al., 2020	del	12 y	116	0.33	[-0.22; 0.88]	0.0%	0.0%
Mora et al., 2017	9.6 w gest	7.7 y	421	0.01	[-0.02; 0.04]	0.7%	1.8%
Chen et al., 2019	del	5 y	196	-0.00	[-0.01; 0.00]	33.9%	42.9%
Manzano-Salgado et al., 2017	1st tr gest	7 y	535	0.03	[-0.14; 0.20]	0.0%	0.0%
**Common effect model**				**-0.00**	**[-0.00; 0.00]**	**100.0%**	--
**Random effects model**				**-0.00**	**[-0.01; 0.00]**	--	**100.0%**

Heterogeneity: $I^2 = 0\%$, $\tau^2 < 0.0001$, $p = 0.64$

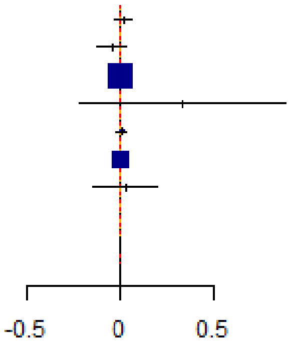

Study	Sampling	Outcome	Subjects	Estimate [95% CI]
Zhang, Lei, et al., 2022	within 3 d before del	7 y	98	0.016 [-0.034; 0.066]
Bloom et al., 2022	8-13 w gest	4-8 y	320	-0.043 [-0.121; 0.035]
Hartman et al., 2017	15 w gest	9 y	311	-0.001 [-0.004; 0.002]
Liu et al., 2020	del	12 y	116	0.329 [-0.219; 0.878]
Mora et al., 2017	9.6 w gest	7.7 y	421	0.010 [-0.017; 0.036]
Chen et al., 2019	del	5 y	196	-0.003 [-0.006; 0.001]
Manzano-Salgado et al., 2017	1st tr gest	7 y	535	0.028 [-0.140; 0.196]
**Summary**				**0.022 [-0.035; 0.079]**

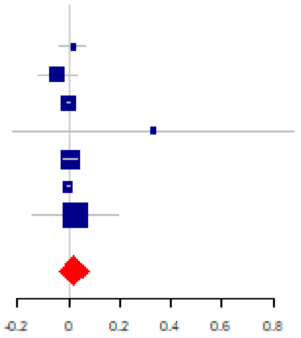

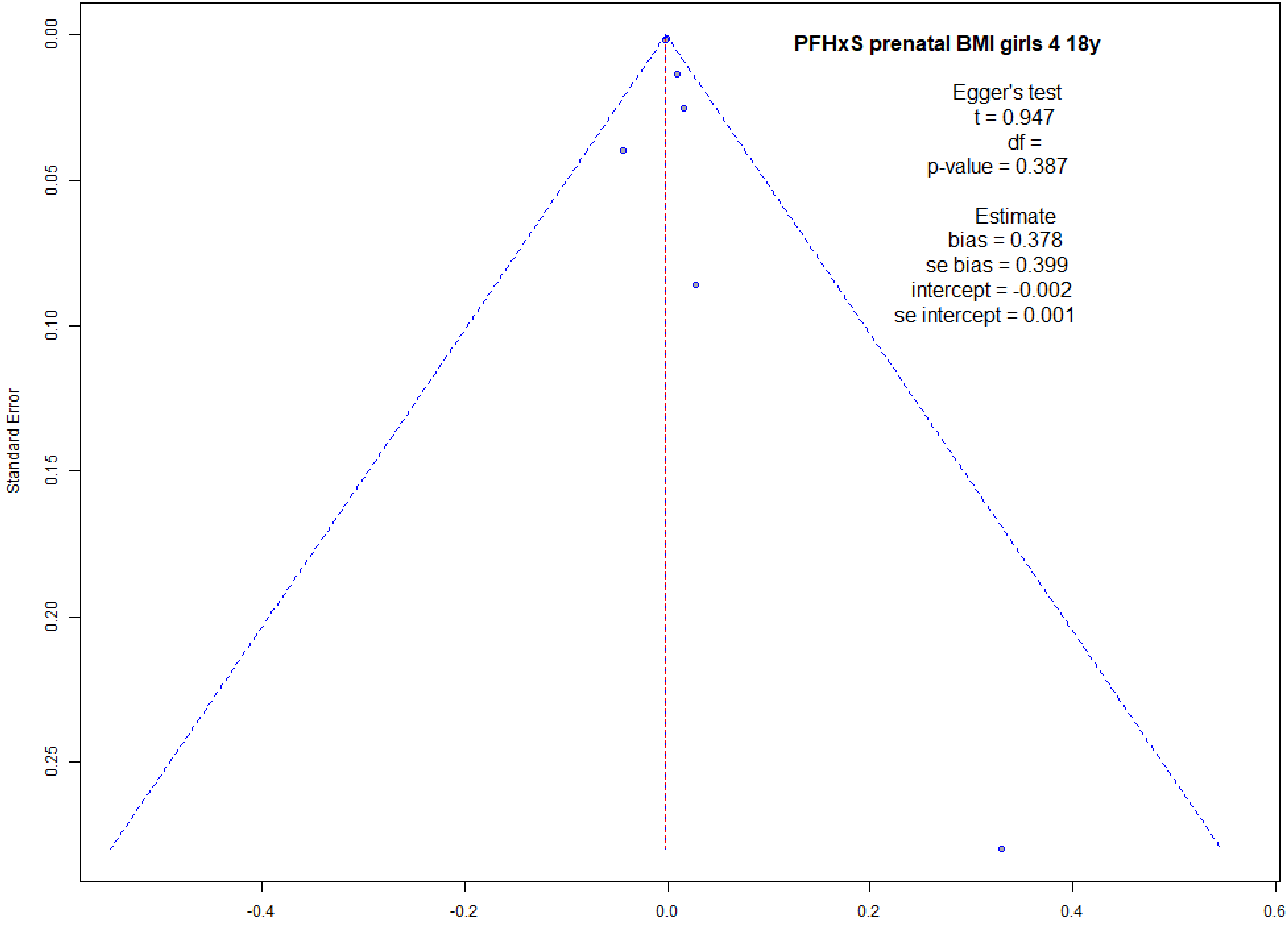
PFHxS prenatal BMI girls 4 18y
Egger's test
t = 0.947
df =
p-value = 0.387
Estimate
bias = 0.378
se bias = 0.399
intercept = -0.002
se intercept = 0.001
Standard Error
0.00
0.05
0.10
0.15
0.20
0.25
-0.4
-0.2
0.0
0.2
0.4
0.6

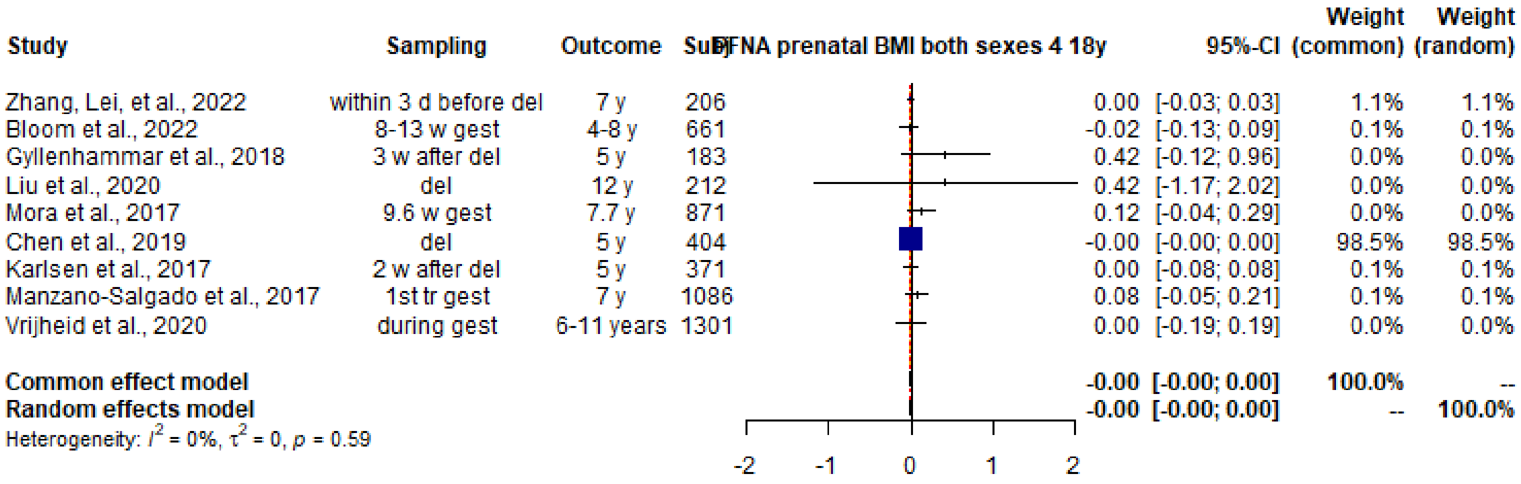
Study
Sampling
Outcome
Sub
PFNA prenatal BMI both sexes 4 18y
95%-CI
Weight (common)
Weight (random)
Zhang, Lei, et al., 2022 | within 3 d before del | 7 y | 206 | 0.00 [-0.03; 0.03] | 1.1% | 1.1%
Bloom et al., 2022 | 8-13 w gest | 4-8 y | 661 | -0.02 [-0.13; 0.09] | 0.1% | 0.1%
Gyllenhammar et al., 2018 | 3 w after del | 5 y | 183 | 0.42 [-0.12; 0.96] | 0.0% | 0.0%
Liu et al., 2020 | del | 12 y | 212 | 0.42 [-1.17; 2.02] | 0.0% | 0.0%
Mora et al., 2017 | 9.6 w gest | 7.7 y | 871 | 0.12 [-0.04; 0.29] | 0.0% | 0.0%
Chen et al., 2019 | del | 5 y | 404 | -0.00 [-0.00; 0.00] | 98.5% | 98.5%
Karlsen et al., 2017 | 2 w after del | 5 y | 371 | 0.00 [-0.08; 0.08] | 0.1% | 0.1%
Manzano-Salgado et al., 2017 | 1st tr gest | 7 y | 1086 | 0.08 [-0.05; 0.21] | 0.1% | 0.1%
Vrijheid et al., 2020 | during gest | 6-11 years | 1301 | 0.00 [-0.19; 0.19] | 0.0% | 0.0%
Common effect model | -0.00 [-0.00; 0.00] | 100.0% | --
Random effects model | -0.00 [-0.00; 0.00] | -- | 100.0%
Heterogeneity: I^2 = 0%, τ^2 = 0, p = 0.59
-2
-1
0
1
2

Study	Sampling	Outcome	Subjects	Estimate [95% CI]
Zhang, Lei, et al., 2022	within 3 d before del	7 y	206	0.001 [-0.029; 0.031]
Bloom et al., 2022	8-13 w gest	4-8 y	661	-0.023 [-0.133; 0.087]
Gyllenhammar et al., 2018	3 w after del	5 y	183	0.423 [-0.115; 0.962]
Liu et al., 2020	del	12 y	212	0.422 [-1.175; 2.018]
Mora et al., 2017	9.6 w gest	7.7 y	871	0.125 [-0.037; 0.288]
Chen et al., 2019	del	5 y	404	0.000 [-0.003; 0.003]
Karlsen et al., 2017	2 w after del	5 y	371	0.000 [-0.082; 0.082]
Manzano-Salgado et al., 2017	1st tr gest	7 y	1086	0.080 [-0.053; 0.213]
Vrijheid et al., 2020	during gest	6-11 years	1301	0.000 [-0.188; 0.188]
**Summary**				**0.066 [-0.025; 0.156]**

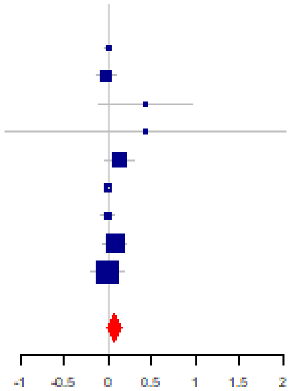

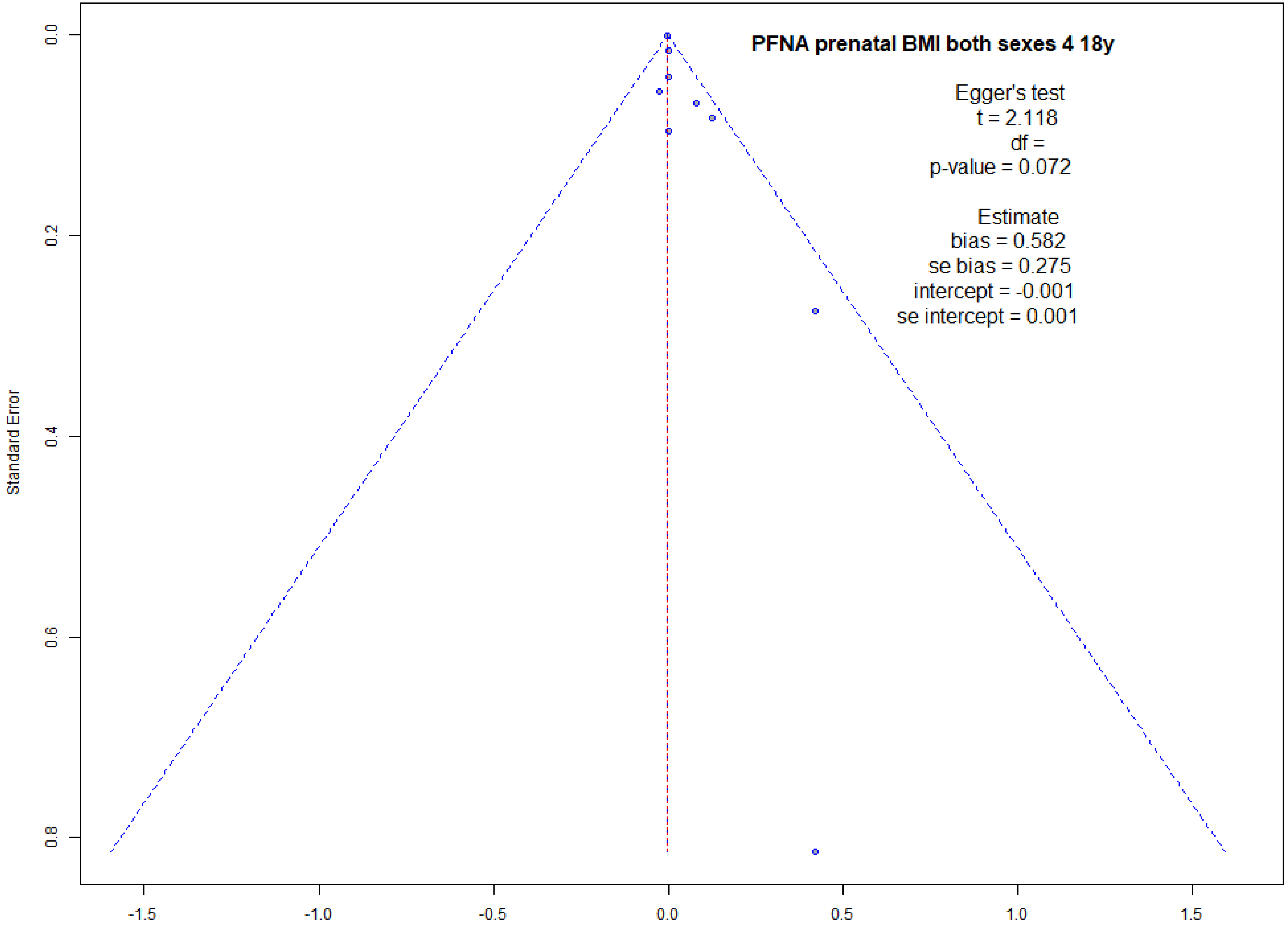
PFNA prenatal BMI both sexes 4 18y
Egger's test
t = 2.118
df =
p-value = 0.072
Estimate
bias = 0.582
se bias = 0.275
intercept = -0.001
se intercept = 0.001
Standard Error
0.0
0.2
0.4
0.6
0.8
-1.5
-1.0
-0.5
0.0
0.5
1.0
1.5

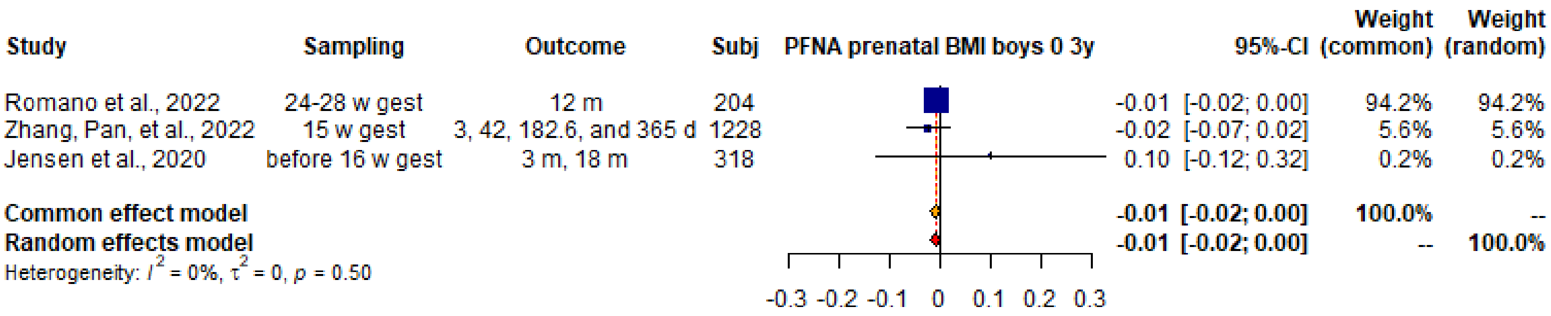
Study
Sampling
Outcome
Subj
PFNA prenatal BMI boys 0 3y
95%-CI
Weight (common)
Weight (random)
Romano et al., 2022 | 24-28 w gest | 12 m | 204 | -0.01 [-0.02; 0.00] | 94.2% | 94.2%
Zhang, Pan, et al., 2022 | 15 w gest | 3, 42, 182.6, and 365 d | 1228 | -0.02 [-0.07; 0.02] | 5.6% | 5.6%
Jensen et al., 2020 | before 16 w gest | 3 m, 18 m | 318 | 0.10 [-0.12; 0.32] | 0.2% | 0.2%
Common effect model | -0.01 [-0.02; 0.00] | 100.0% | --
Random effects model | -0.01 [-0.02; 0.00] | -- | 100.0%
Heterogeneity: I^2 = 0%, τ^2 = 0, p = 0.50
-0.3 -0.2 -0.1 0 0.1 0.2 0.3

Study	Sampling	Outcome	Subjects	Estimate [95% CI]
Romano et al., 2022	24-28 w gest	12 m	204	-0.008 [-0.018; 0.003]
Zhang, Pan, et al., 2022	15 w gest	3, 42, 182.6, and 365 d	1228	-0.023 [-0.065; 0.019]
Jensen et al., 2020	before 16 w gest	3 m, 18 m	318	0.100 [-0.125; 0.325]
**Summary**				**0.001 [-0.050; 0.051]**

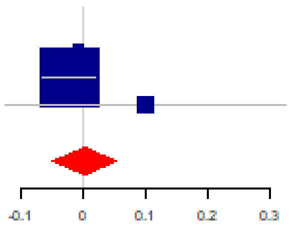

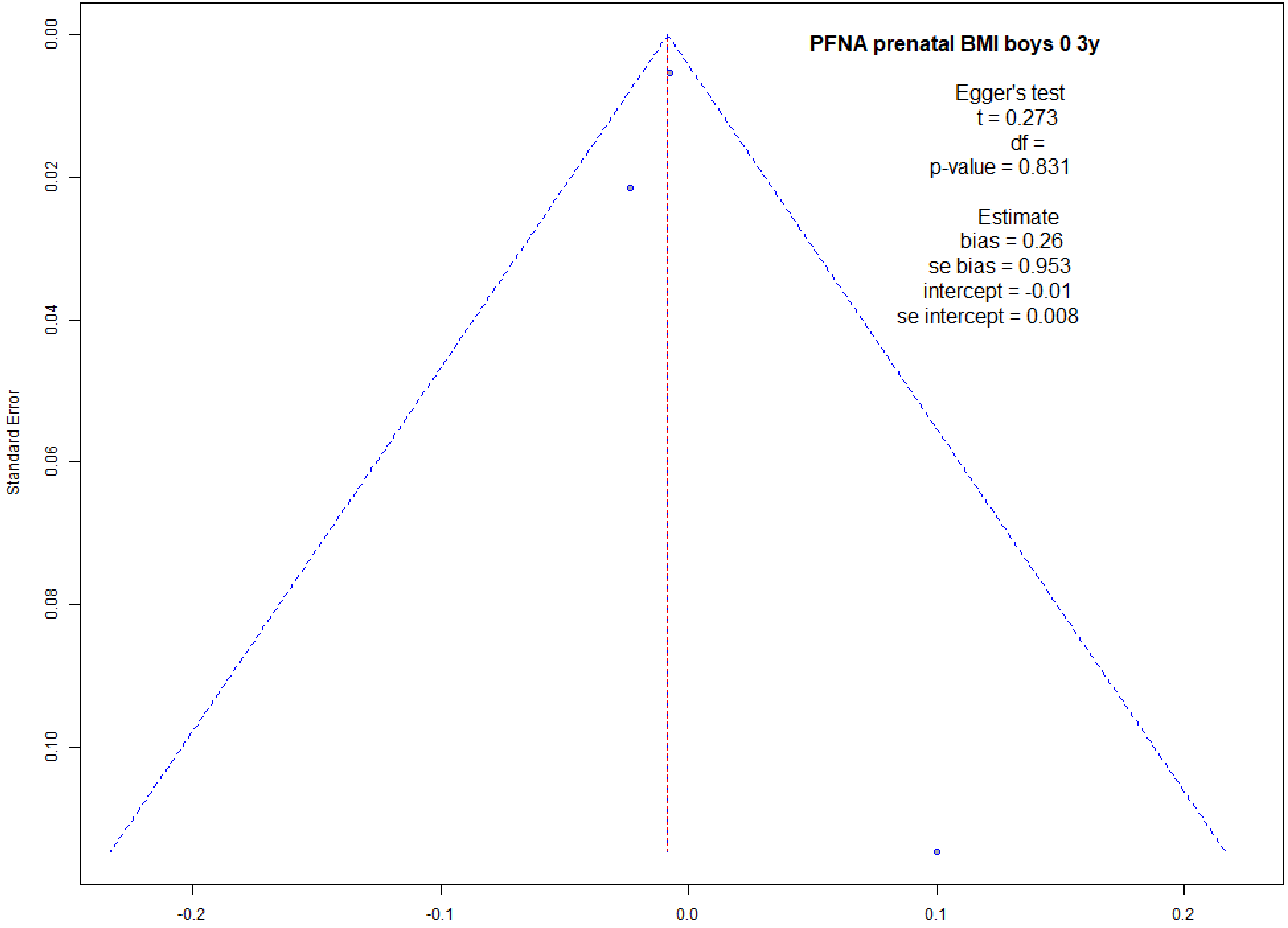
PFNA prenatal BMI boys 0 3y
Egger's test
t = 0.273
df =
p-value = 0.831
Estimate
bias = 0.26
se bias = 0.953
intercept = -0.01
se intercept = 0.008
Standard Error
0.00
0.02
0.04
0.06
0.08
0.10
-0.2
-0.1
0.0
0.1
0.2

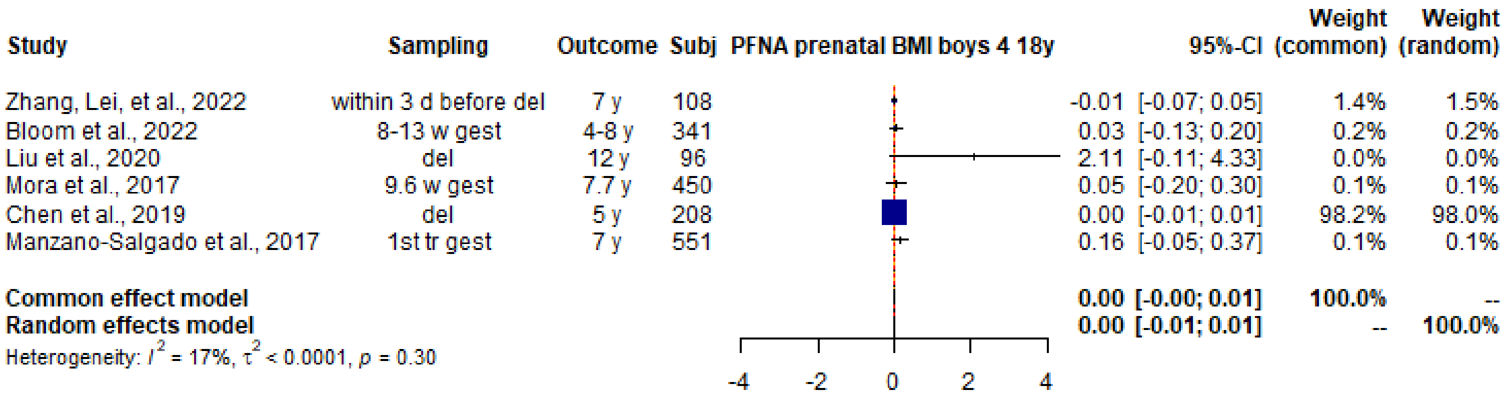
Study | Sampling | Outcome | Subj | PFNA prenatal BMI boys 4 18y | 95%-CI | Weight (common) | Weight (random)
Zhang, Lei, et al., 2022 | within 3 d before del | 7 y | 108 | -0.01 [-0.07; 0.05] | 1.4% | 1.5%
Bloom et al., 2022 | 8-13 w gest | 4-8 y | 341 | 0.03 [-0.13; 0.20] | 0.2% | 0.2%
Liu et al., 2020 | del | 12 y | 96 | 2.11 [-0.11; 4.33] | 0.0% | 0.0%
Mora et al., 2017 | 9.6 w gest | 7.7 y | 450 | 0.05 [-0.20; 0.30] | 0.1% | 0.1%
Chen et al., 2019 | del | 5 y | 208 | 0.00 [-0.01; 0.01] | 98.2% | 98.0%
Manzano-Salgado et al., 2017 | 1st tr gest | 7 y | 551 | 0.16 [-0.05; 0.37] | 0.1% | 0.1%
Common effect model | 0.00 [-0.00; 0.01] | 100.0% | --
Random effects model | 0.00 [-0.01; 0.01] | -- | 100.0%
Heterogeneity: I^2 = 17%, τ^2 < 0.0001, p = 0.30
-4
-2
0
2
4

Study	Sampling	Outcome	Subjects	Estimate [95% CI]
Zhang, Lei, et al., 2022	within 3 d before del	7 y	108	-0.010 [-0.070; 0.051]
Bloom et al., 2022	8-13 w gest	4-8 y	341	0.035 [-0.127; 0.196]
Liu et al., 2020	del	12 y	96	2.113 [-0.106; 4.332]
Mora et al., 2017	9.6 w gest	7.7 y	450	0.050 [-0.200; 0.300]
Chen et al., 2019	del	5 y	208	0.002 [-0.005; 0.009]
Manzano-Salgado et al., 2017	1st tr gest	7 y	551	0.160 [-0.053; 0.373]
**Summary**				**0.185 [0.028; 0.342]**

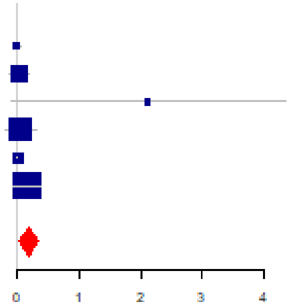

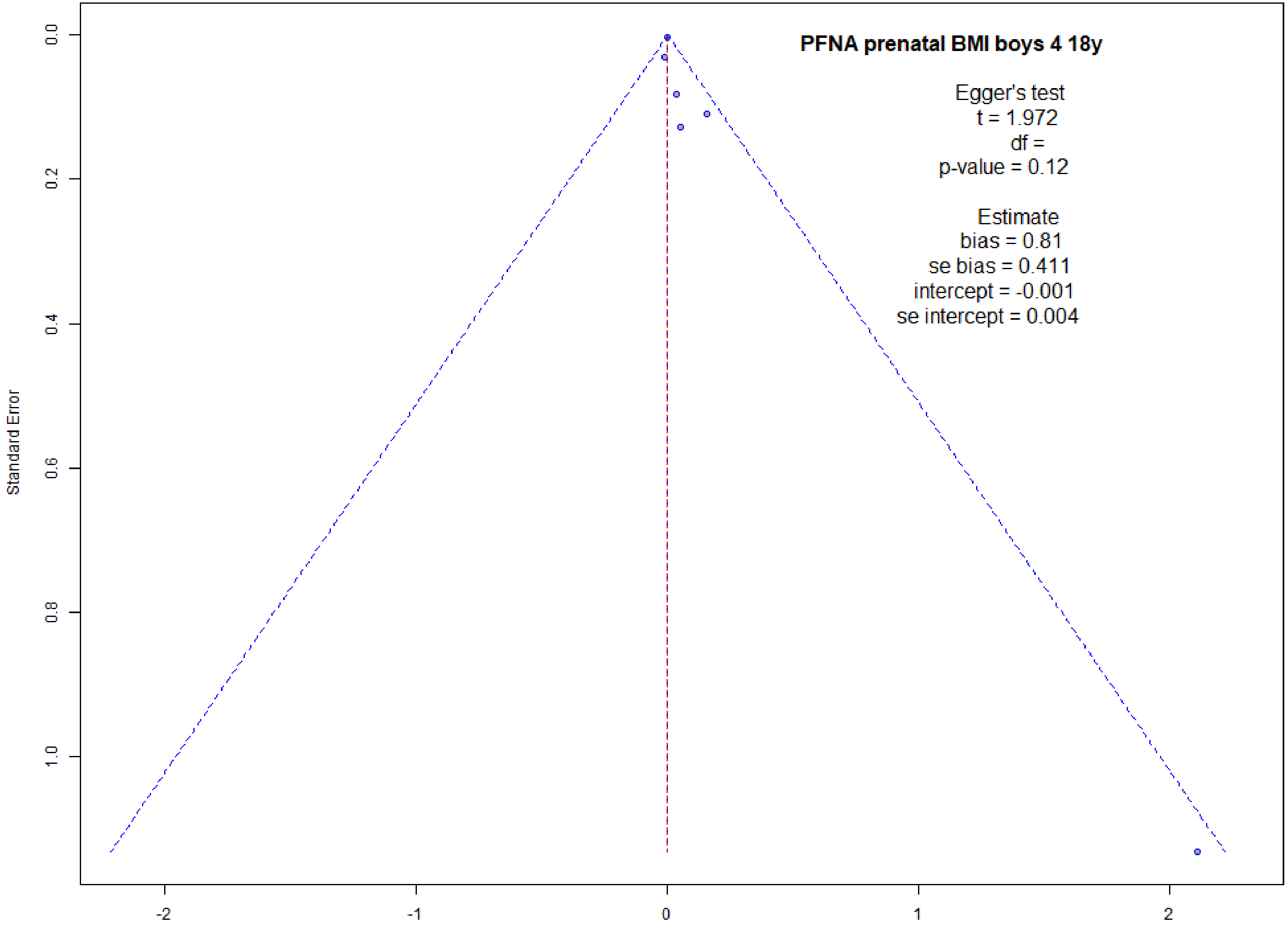
PFNA prenatal BMI boys 4 18y
Egger's test
t = 1.972
df =
p-value = 0.12
Estimate
bias = 0.81
se bias = 0.411
intercept = -0.001
se intercept = 0.004
Standard Error
0.0
0.2
0.4
0.6
0.8
1.0
-2
-1
0
1
2

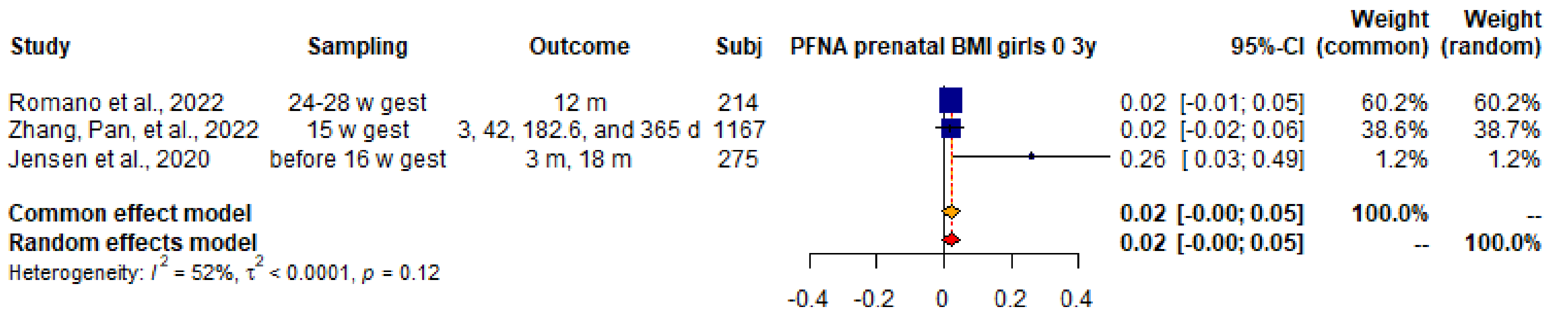
Study
Sampling
Outcome
Subj
PFNA prenatal BMI girls 0 3y
95%-CI
Weight (common)
Weight (random)
Romano et al., 2022
24-28 w gest
12 m
214
0.02 [-0.01; 0.05]
60.2%
60.2%
Zhang, Pan, et al., 2022
15 w gest
3, 42, 182.6, and 365 d
1167
0.02 [-0.02; 0.06]
38.6%
38.7%
Jensen et al., 2020
before 16 w gest
3 m, 18 m
275
0.26 [0.03; 0.49]
1.2%
1.2%
Common effect model
0.02 [-0.00; 0.05]
100.0%
--
Random effects model
0.02 [-0.00; 0.05]
--
100.0%
Heterogeneity: I^2 = 52%, τ^2 < 0.0001, p = 0.12
-0.4
-0.2
0
0.2
0.4

Study	Sampling	Outcome	Subjects	Estimate [95% CI]
Romano et al., 2022	24-28 w gest	12 m	214	0.019 [-0.013; 0.051]
Zhang, Pan, et al., 2022	15 w gest	3, 42, 182.6, and 365 d	1167	0.019 [-0.021; 0.058]
Jensen et al., 2020	before 16 w gest	3 m, 18 m	275	0.260 [0.030; 0.490]
**Summary**				**0.059 [-0.002; 0.120]**

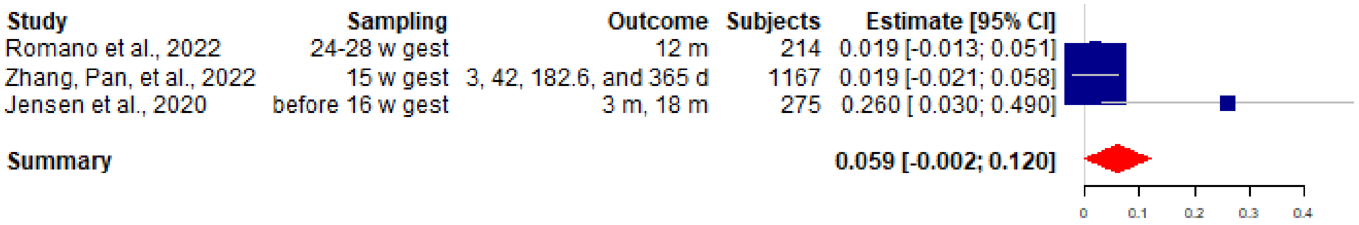

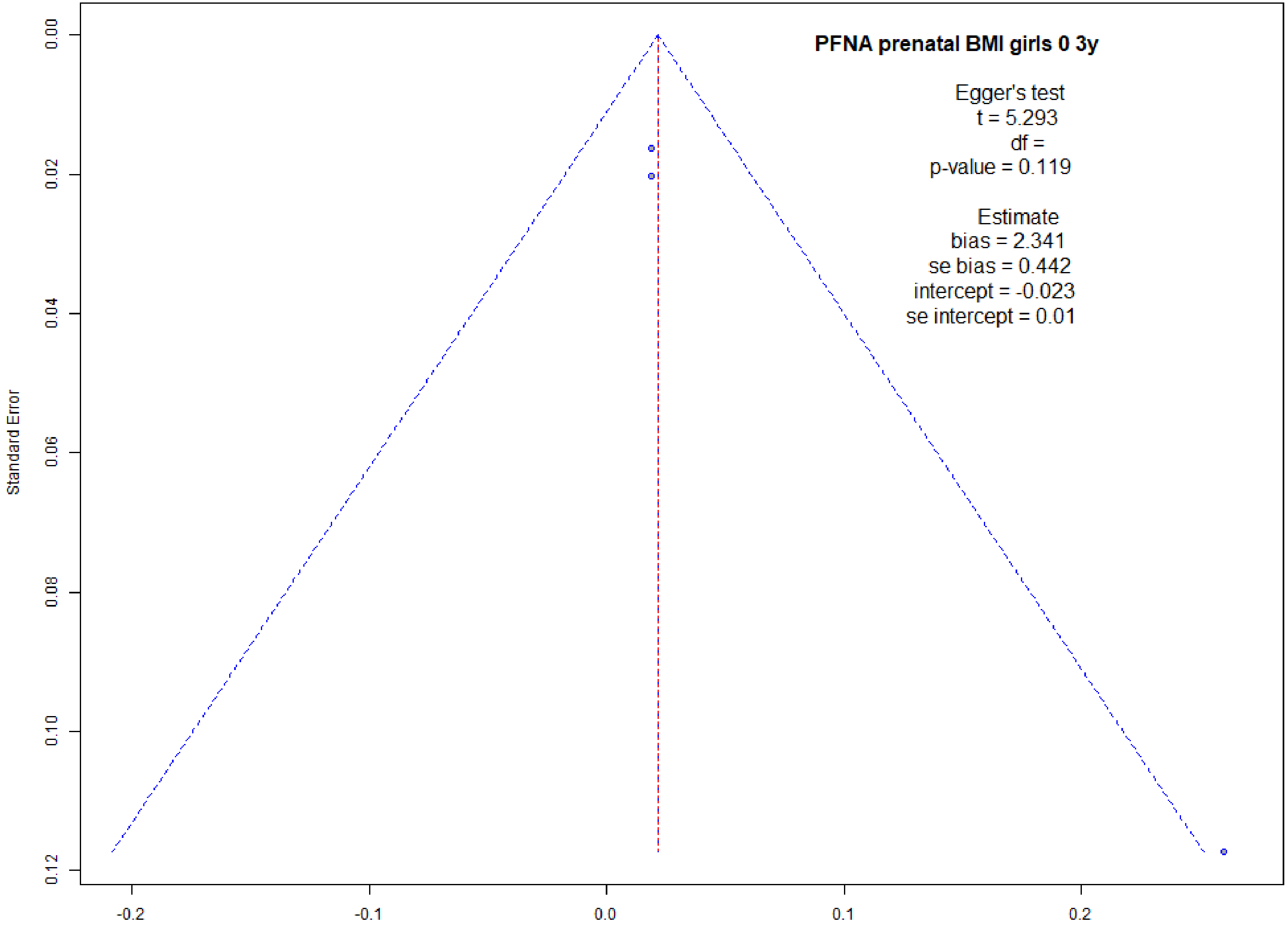
PFNA prenatal BMI girls 0 3y
Egger's test
t = 5.293
df =
p-value = 0.119
Estimate
bias = 2.341
se bias = 0.442
intercept = -0.023
se intercept = 0.01
Standard Error
0.00
0.02
0.04
0.06
0.08
0.10
0.12
-0.2
-0.1
0.0
0.1
0.2

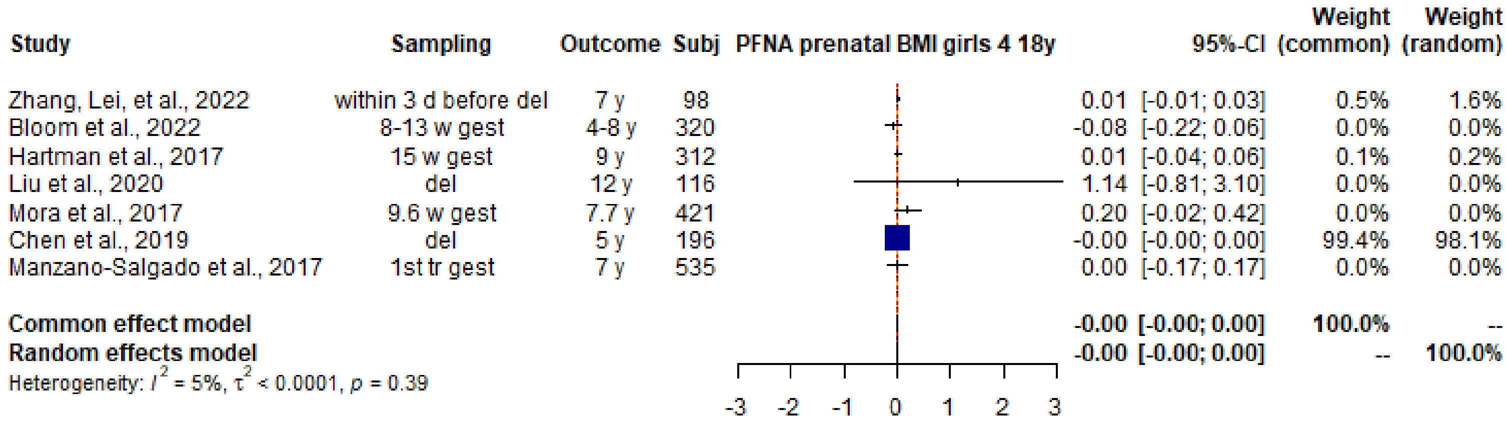
Study
Sampling
Outcome
Subj
PFNA prenatal BMI girls 4 18y
95%-CI
Weight (common)
Weight (random)
Zhang, Lei, et al., 2022 within 3 d before del 7 y 98 0.01 [-0.01; 0.03] 0.5% 1.6%
Bloom et al., 2022 8-13 w gest 4-8 y 320 -0.08 [-0.22; 0.06] 0.0% 0.0%
Hartman et al., 2017 15 w gest 9 y 312 0.01 [-0.04; 0.06] 0.1% 0.2%
Liu et al., 2020 del 12 y 116 1.14 [-0.81; 3.10] 0.0% 0.0%
Mora et al., 2017 9.6 w gest 7.7 y 421 0.20 [-0.02; 0.42] 0.0% 0.0%
Chen et al., 2019 del 5 y 196 -0.00 [-0.00; 0.00] 99.4% 98.1%
Manzano-Salgado et al., 2017 1st tr gest 7 y 535 0.00 [-0.17; 0.17] 0.0% 0.0%
Common effect model -0.00 [-0.00; 0.00] 100.0% --
Random effects model -0.00 [-0.00; 0.00] -- 100.0%
Heterogeneity: I^2 = 5%, τ^2 < 0.0001, p = 0.39
-3 -2 -1 0 1 2 3

Study	Sampling	Outcome	Subjects	Estimate [95% CI]
Zhang, Lei, et al., 2022	within 3 d before del	7 y	98	0.007 [-0.013; 0.027]
Bloom et al., 2022	8-13 w gest	4-8 y	320	-0.081 [-0.219; 0.058]
Hartman et al., 2017	15 w gest	9 y	312	0.011 [-0.040; 0.061]
Liu et al., 2020	del	12 y	116	1.145 [-0.813; 3.103]
Mora et al., 2017	9.6 w gest	7.7 y	421	0.200 [-0.025; 0.425]
Chen et al., 2019	del	5 y	196	0.000 [-0.002; 0.001]
Manzano-Salgado et al., 2017	1st tr gest	7 y	535	0.000 [-0.166; 0.166]
**Summary**				**0.098 [-0.036; 0.231]**

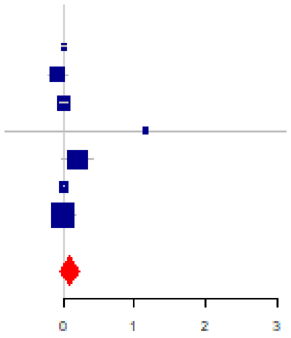

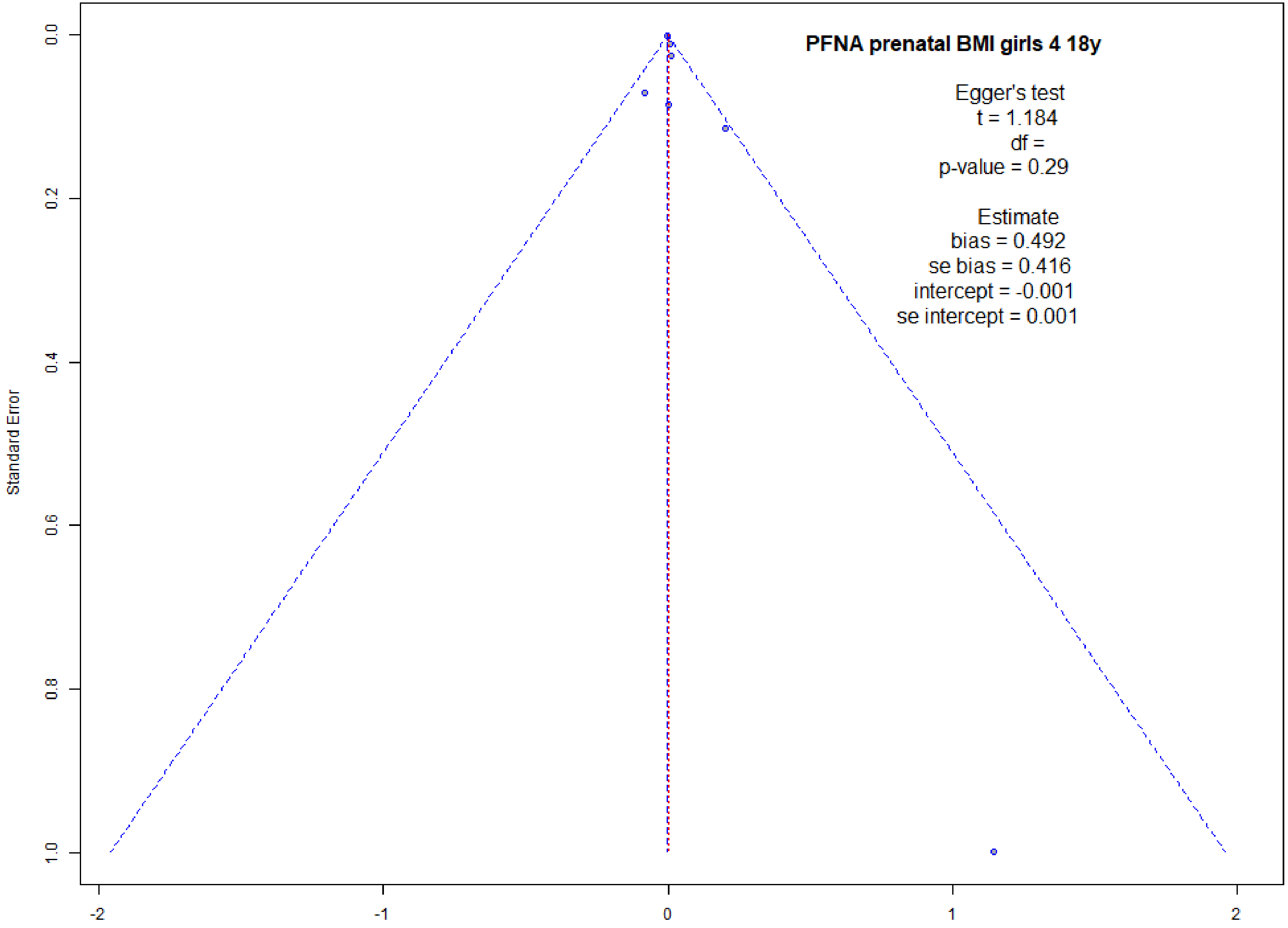
PFNA prenatal BMI girls 4 18y
Egger's test
t = 1.184
df =
p-value = 0.29
Estimate
bias = 0.492
se bias = 0.416
intercept = -0.001
se intercept = 0.001
Standard Error
0.0
0.2
0.4
0.6
0.8
1.0
-2
-1
0
1
2

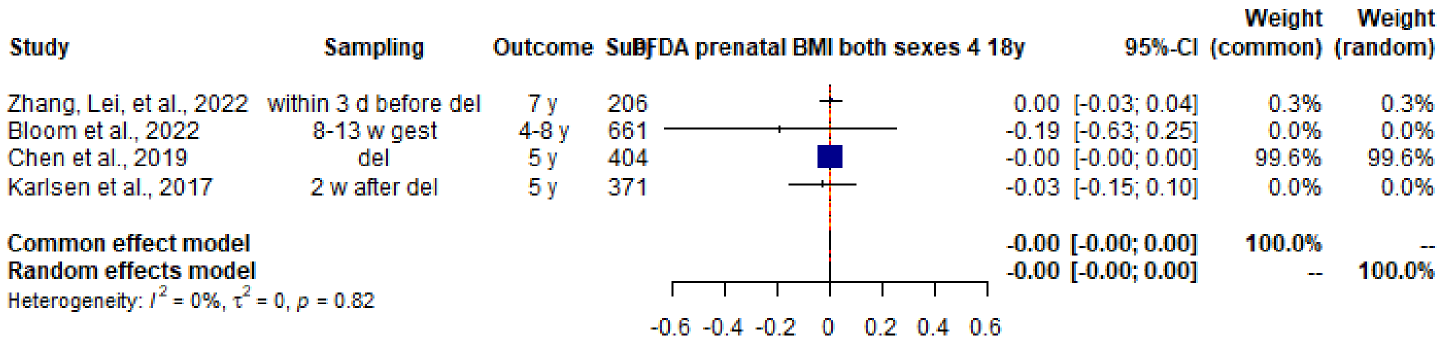
PFDA prenatal BMI both sexes 4 18y
Study
Sampling
Outcome
Subj
95%-CI
Weight (common)
Weight (random)
Zhang, Lei, et al., 2022 within 3 d before del 7 y 206 0.00 [-0.03; 0.04] 0.3% 0.3%
Bloom et al., 2022 8-13 w gest 4-8 y 661 -0.19 [-0.63; 0.25] 0.0% 0.0%
Chen et al., 2019 del 5 y 404 -0.00 [-0.00; 0.00] 99.6% 99.6%
Karlsen et al., 2017 2 w after del 5 y 371 -0.03 [-0.15; 0.10] 0.0% 0.0%
Common effect model -0.00 [-0.00; 0.00] 100.0% --
Random effects model -0.00 [-0.00; 0.00] -- 100.0%
Heterogeneity: I^2 = 0%, τ^2 = 0, p = 0.82
-0.6 -0.4 -0.2 0 0.2 0.4 0.6

Study	Sampling	Outcome	Subjects	Estimate [95% CI]
Zhang, Lei, et al., 2022	within 3 d before del	7 y	206	0.004 [-0.035; 0.042]
Bloom et al., 2022	8-13 w gest	4-8 y	661	-0.188 [-0.626; 0.251]
Chen et al., 2019	del	5 y	404	-0.001 [-0.003; 0.002]
Karlsen et al., 2017	2 w after del	5 y	371	-0.027 [-0.154; 0.099]
**Summary**				**-0.082 [-0.260; 0.097]**

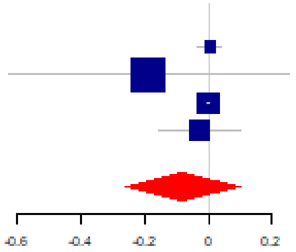

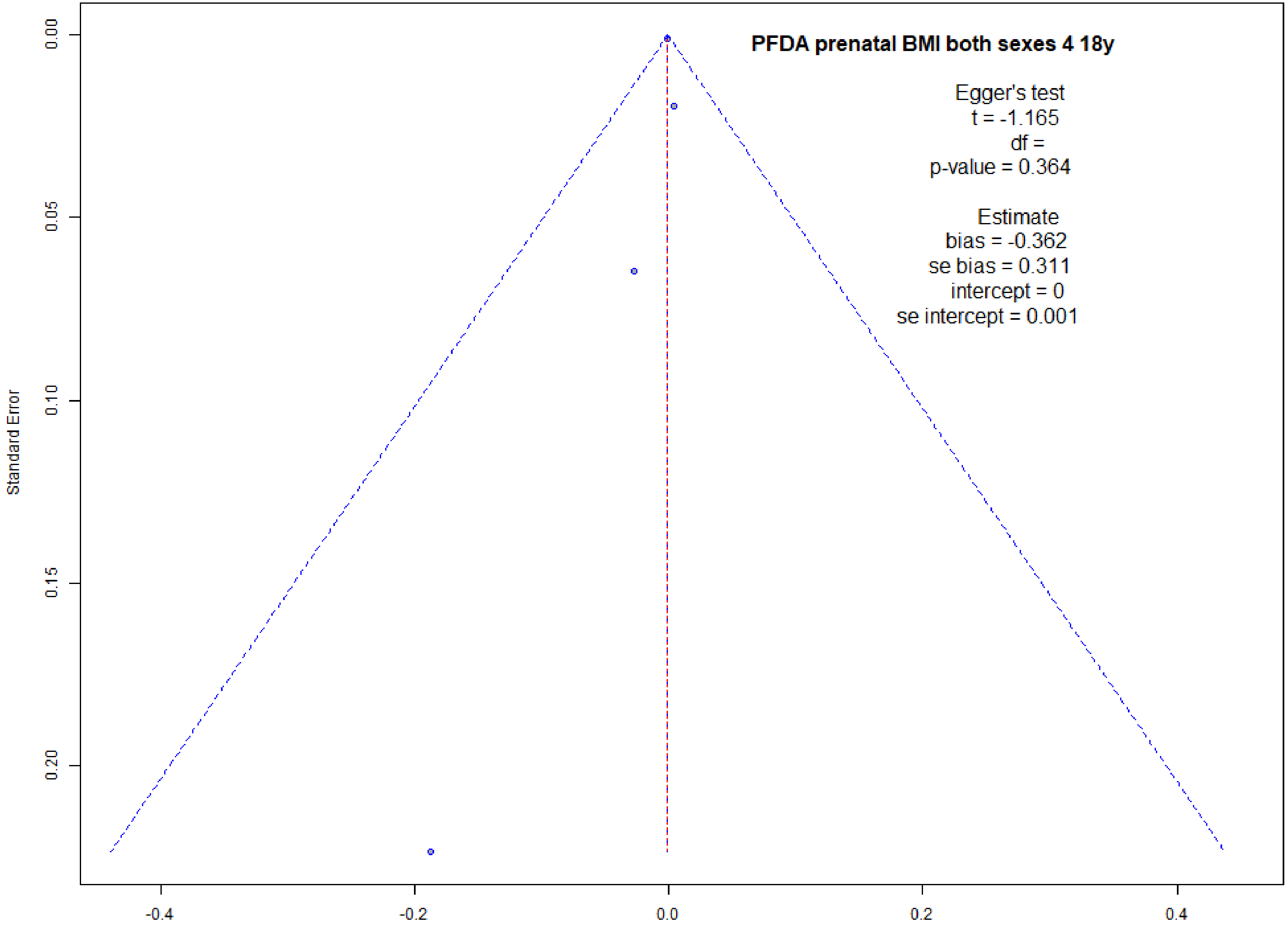
PFDA prenatal BMI both sexes 4 18y
Egger's test
t = -1.165
df =
p-value = 0.364
Estimate
bias = -0.362
se bias = 0.311
intercept = 0
se intercept = 0.001
Standard Error
0.00
0.05
0.10
0.15
0.20
-0.4
-0.2
0.0
0.2
0.4

Study	Sampling	Outcome	Subj	PFDA prenatal BMI boys 0 3y	95%-CI	Weight (common)	Weight (random)
Romano et al., 2022	24-28 w gest	12 m	204	-0.01	[-0.03; 0.02]	60.2%	60.2%
Zhang, Pan, et al., 2022	15 w gest	3, 42, 182.6, and 365 d	1228	-0.01	[-0.04; 0.03]	39.7%	39.7%
Jensen et al., 2020	before 16 w gest	3 m, 18 m	318	0.24	[-0.32; 0.80]	0.2%	0.2%
**Common effect model**				**-0.01**	**[-0.03; 0.01]**	**100.0%**	**--**
**Random effects model**				**-0.01**	**[-0.03; 0.01]**	**--**	**100.0%**

Heterogeneity: $I^2 = 0\%$, $\tau^2 = 0$, $p = 0.68$

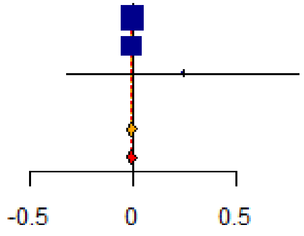

Study	Sampling	Outcome	Subjects	Estimate [95% CI]
Romano et al., 2022	24-28 w gest	12 m	204	-0.006 [-0.034; 0.022]
Zhang, Pan, et al., 2022	15 w gest	3, 42, 182.6, and 365 d	1228	-0.009 [-0.044; 0.025]
Jensen et al., 2020	before 16 w gest	3 m, 18 m	318	0.240 [-0.315; 0.795]
**Summary**				**0.036 [-0.067; 0.140]**

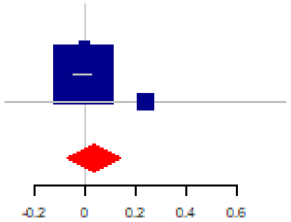

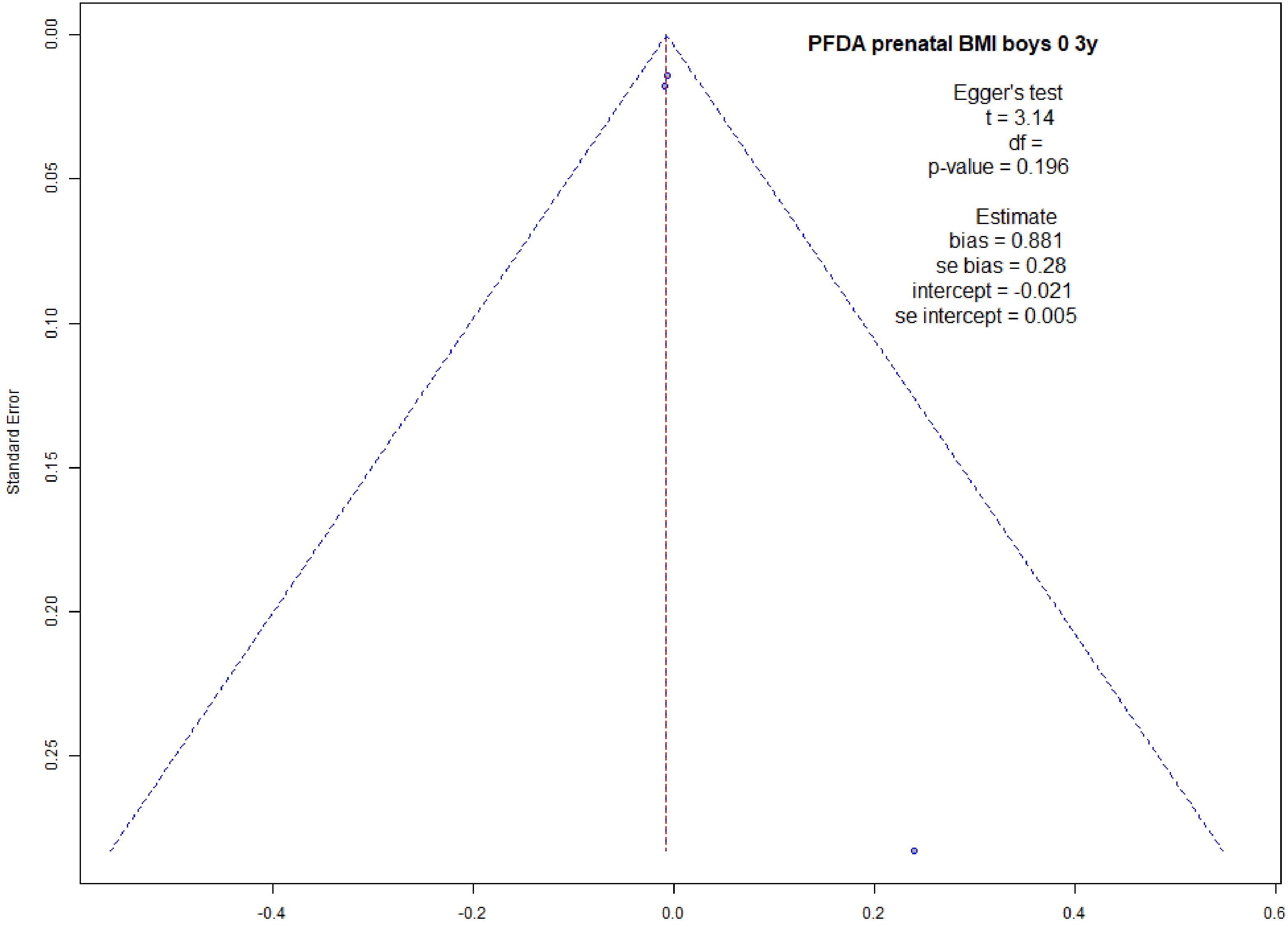
PFDA prenatal BMI boys 0 3y
Egger's test
t = 3.14
df =
p-value = 0.196
Estimate
bias = 0.881
se bias = 0.28
intercept = -0.021
se intercept = 0.005
Standard Error
0.00
0.05
0.10
0.15
0.20
0.25
-0.4
-0.2
0.0
0.2
0.4
0.6

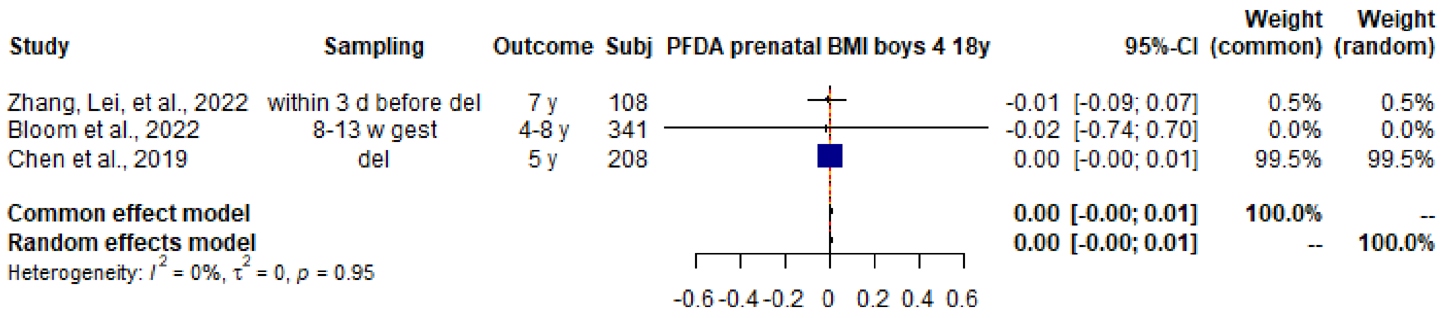
Study
Sampling
Outcome
Subj
PFDA prenatal BMI boys 4 18y
95%-CI
Weight (common)
Weight (random)
Zhang, Lei, et al., 2022 within 3 d before del 7 y 108 -0.01 [-0.09; 0.07] 0.5% 0.5%
Bloom et al., 2022 8-13 w gest 4-8 y 341 -0.02 [-0.74; 0.70] 0.0% 0.0%
Chen et al., 2019 del 5 y 208 0.00 [-0.00; 0.01] 99.5% 99.5%
Common effect model 0.00 [-0.00; 0.01] 100.0% --
Random effects model 0.00 [-0.00; 0.01] -- 100.0%
Heterogeneity: I^2 = 0%, τ^2 = 0, p = 0.95
-0.6 -0.4 -0.2 0 0.2 0.4 0.6

Study	Sampling	Outcome	Subjects	Estimate [95% CI]
Zhang, Lei, et al., 2022	within 3 d before del	7 y	108	-0.011 [-0.091; 0.069]
Bloom et al., 2022	8-13 w gest	4-8 y	341	-0.019 [-0.739; 0.702]
Chen et al., 2019	del	5 y	208	0.002 [-0.004; 0.007]
**Summary**				**-0.011 [-0.385; 0.363]**

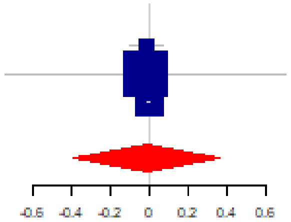

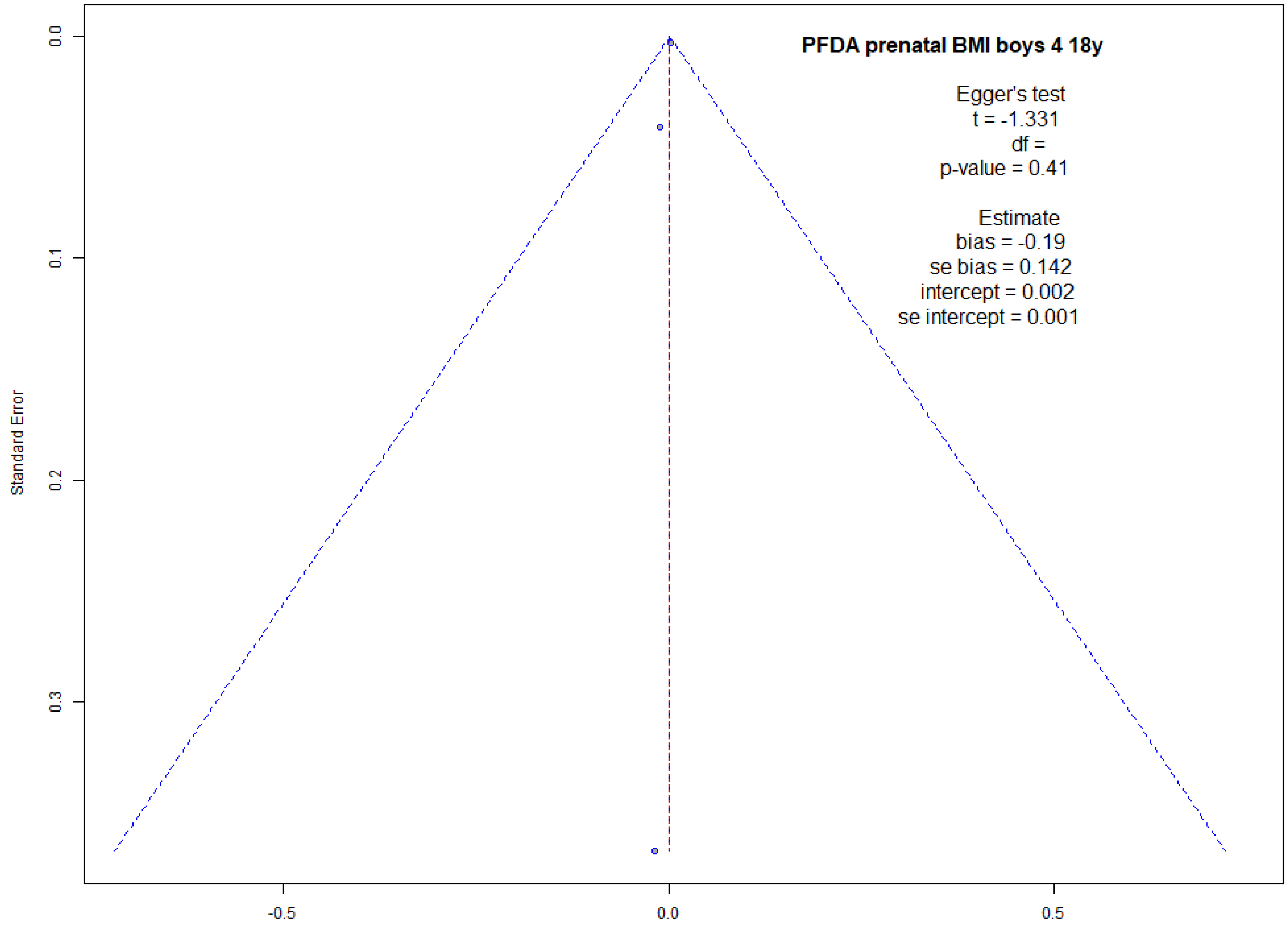
PFDA prenatal BMI boys 4 18y
Egger's test
t = -1.331
df =
p-value = 0.41
Estimate
bias = -0.19
se bias = 0.142
intercept = 0.002
se intercept = 0.001
Standard Error
0.0
0.1
0.2
0.3
-0.5
0.0
0.5

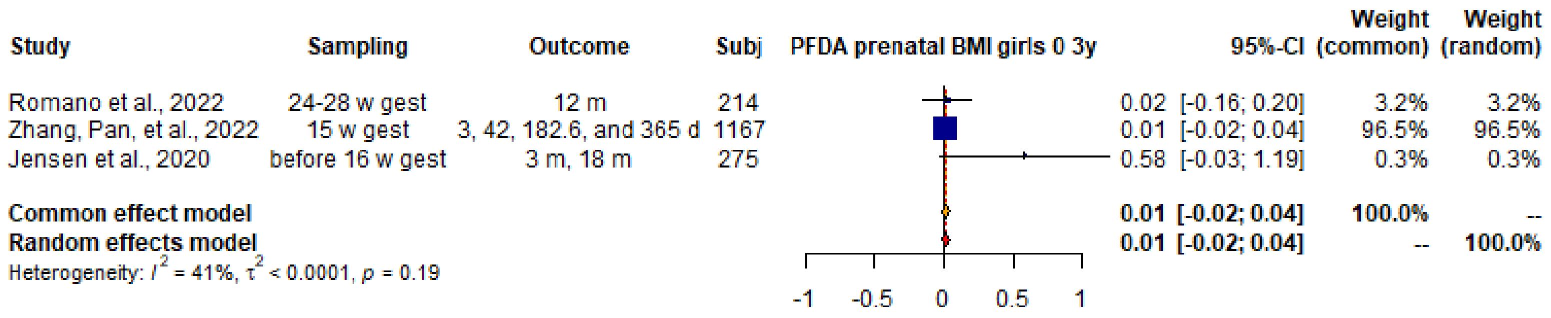
Study
Sampling
Outcome
Subj
PFDA prenatal BMI girls 0 3y
95%-CI
Weight (common)
Weight (random)
Romano et al., 2022 | 24-28 w gest | 12 m | 214 | 0.02 [-0.16; 0.20] | 3.2% | 3.2%
Zhang, Pan, et al., 2022 | 15 w gest | 3, 42, 182.6, and 365 d | 1167 | 0.01 [-0.02; 0.04] | 96.5% | 96.5%
Jensen et al., 2020 | before 16 w gest | 3 m, 18 m | 275 | 0.58 [-0.03; 1.19] | 0.3% | 0.3%
Common effect model | 0.01 [-0.02; 0.04] | 100.0% | --
Random effects model | 0.01 [-0.02; 0.04] | -- | 100.0%
Heterogeneity: I^2 = 41%, τ^2 < 0.0001, p = 0.19
-1
-0.5
0
0.5
1

Study	Sampling	Outcome	Subjects	Estimate [95% CI]
Romano et al., 2022	24-28 w gest	12 m	214	0.021 [-0.156; 0.197]
Zhang, Pan, et al., 2022	15 w gest	3, 42, 182.6, and 365 d	1167	0.009 [-0.023; 0.041]
Jensen et al., 2020	before 16 w gest	3 m, 18 m	275	0.580 [-0.030; 1.190]
**Summary**				**0.105 [-0.044; 0.255]**

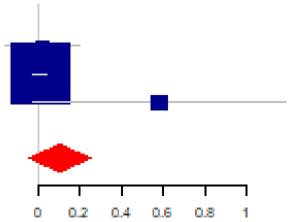

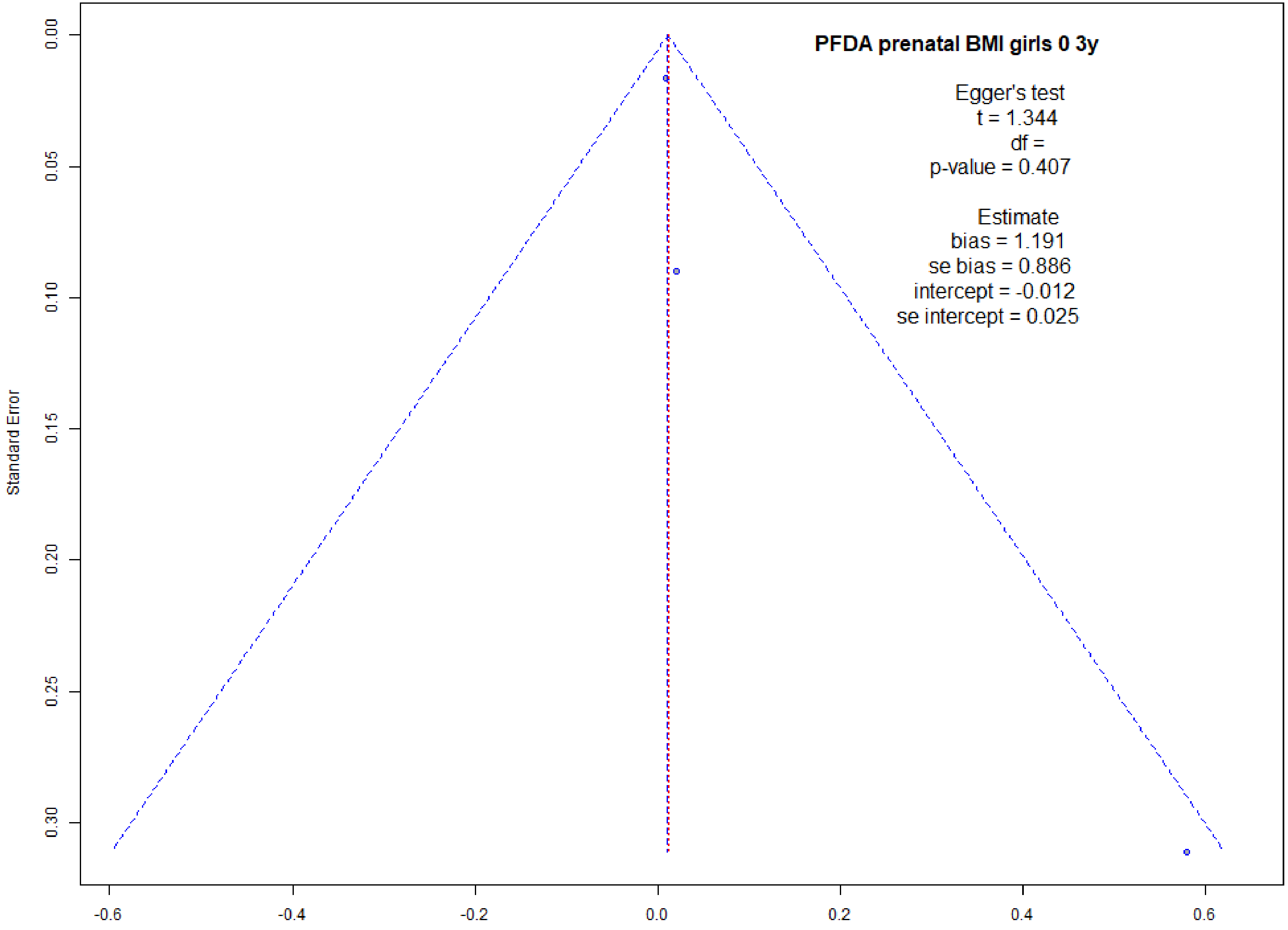
PFDA prenatal BMI girls 0 3y
Egger's test
t = 1.344
df =
p-value = 0.407
Estimate
bias = 1.191
se bias = 0.886
intercept = -0.012
se intercept = 0.025
Standard Error
0.00
0.05
0.10
0.15
0.20
0.25
0.30
-0.6
-0.4
-0.2
0.0
0.2
0.4
0.6

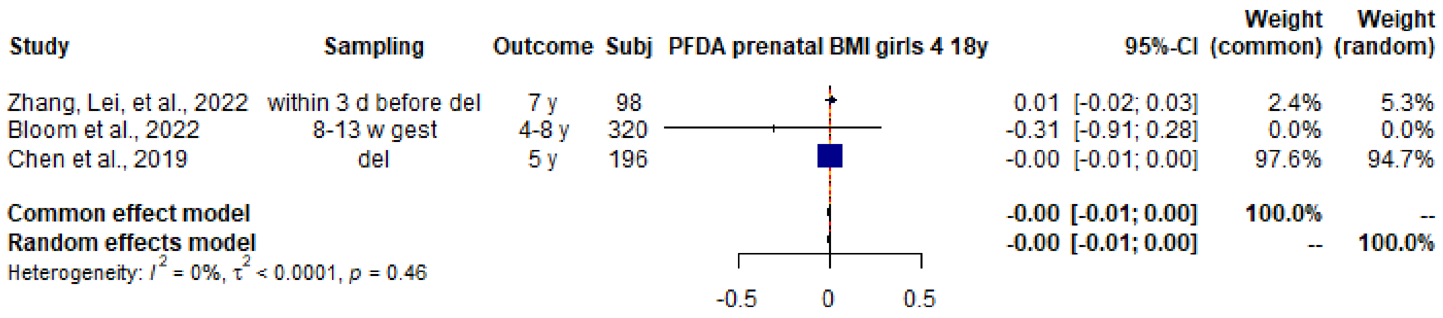
Study
Sampling
Outcome
Subj
PFDA prenatal BMI girls 4 18y
95%-CI
Weight (common)
Weight (random)
Zhang, Lei, et al., 2022 within 3 d before del 7 y 98 0.01 [-0.02; 0.03] 2.4% 5.3%
Bloom et al., 2022 8-13 w gest 4-8 y 320 -0.31 [-0.91; 0.28] 0.0% 0.0%
Chen et al., 2019 del 5 y 196 -0.00 [-0.01; 0.00] 97.6% 94.7%
Common effect model -0.00 [-0.01; 0.00] 100.0% --
Random effects model -0.00 [-0.01; 0.00] -- 100.0%
Heterogeneity: I^2 = 0%, τ^2 < 0.0001, p = 0.46
-0.5
0
0.5

Study	Sampling	Outcome	Subjects	Estimate [95% CI]
Zhang, Lei, et al., 2022	within 3 d before del	7 y	98	0.007 [-0.018; 0.033]
Bloom et al., 2022	8-13 w gest	4-8 y	320	-0.313 [-0.908; 0.282]
Chen et al., 2019	del	5 y	196	-0.002 [-0.006; 0.002]
**Summary**				**-0.163 [-0.473; 0.147]**

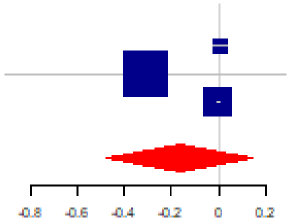

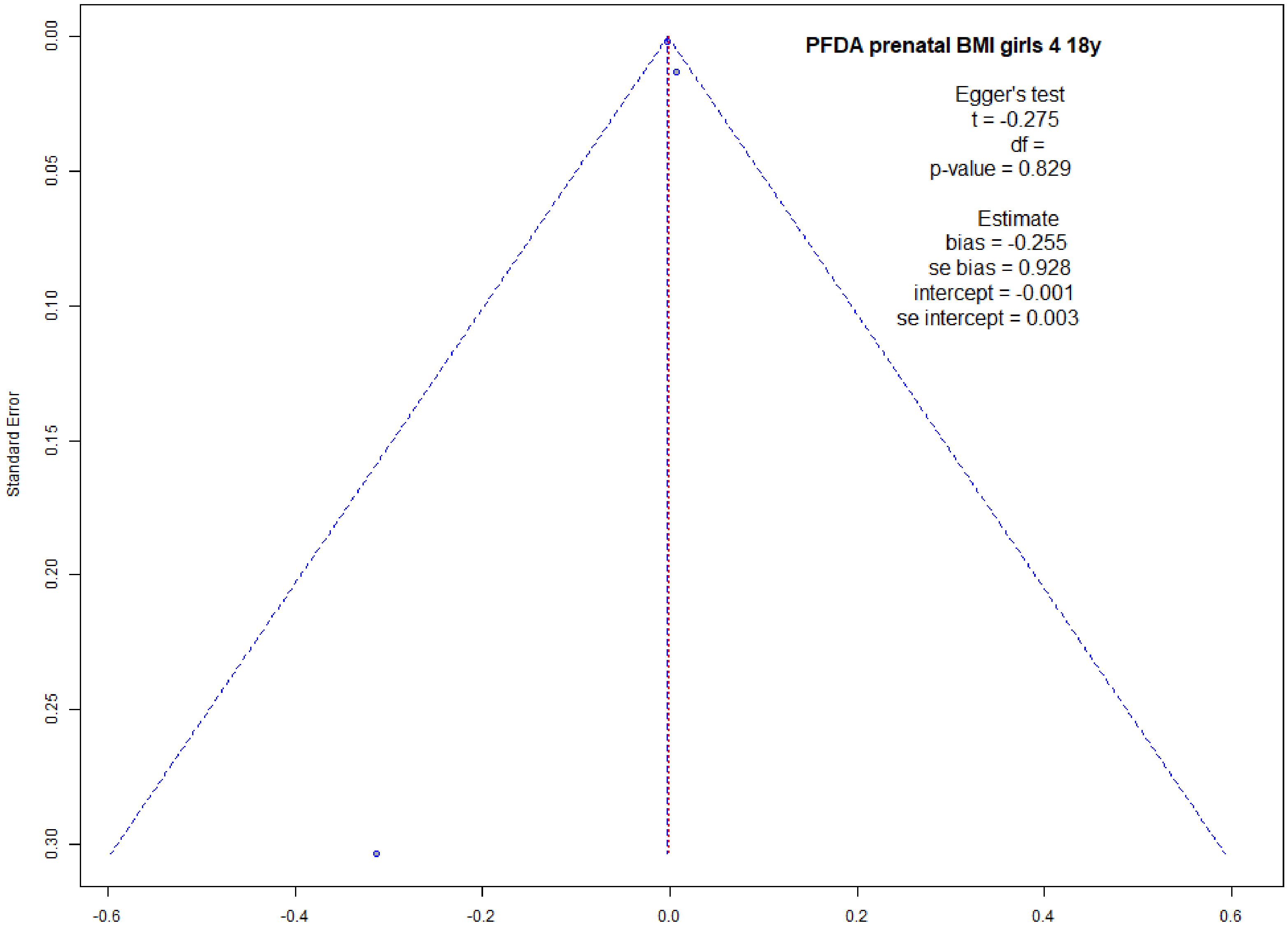
PFDA prenatal BMI girls 4 18y
Egger's test
t = -0.275
df =
p-value = 0.829
Estimate
bias = -0.255
se bias = 0.928
intercept = -0.001
se intercept = 0.003
Standard Error
0.00
0.05
0.10
0.15
0.20
0.25
0.30
-0.6
-0.4
-0.2
0.0
0.2
0.4
0.6

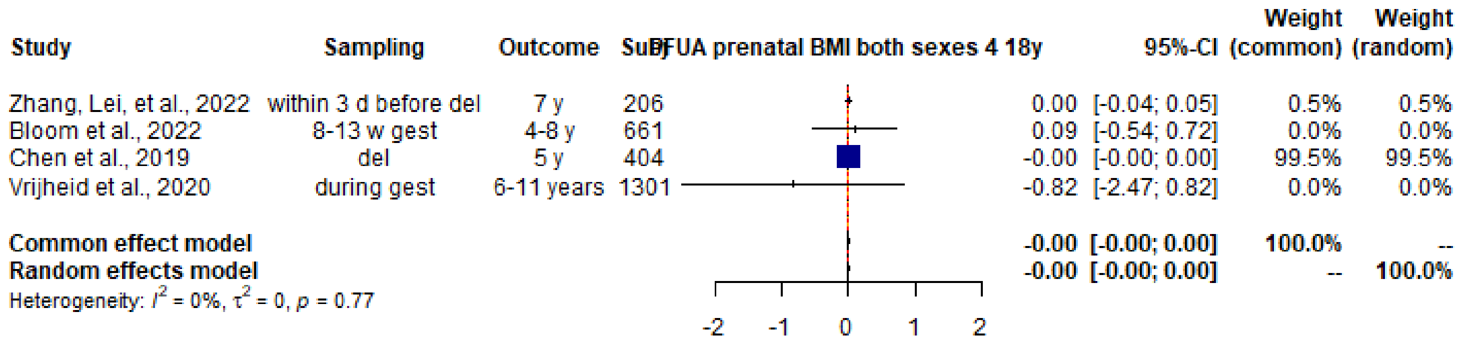
Study
Sampling
Outcome
Subj
PFUA prenatal BMI both sexes 4 18y
95%-CI
Weight (common)
Weight (random)
Zhang, Lei, et al., 2022 within 3 d before del 7 y 206 0.00 [-0.04; 0.05] 0.5% 0.5%
Bloom et al., 2022 8-13 w gest 4-8 y 661 0.09 [-0.54; 0.72] 0.0% 0.0%
Chen et al., 2019 del 5 y 404 -0.00 [-0.00; 0.00] 99.5% 99.5%
Vrijheid et al., 2020 during gest 6-11 years 1301 -0.82 [-2.47; 0.82] 0.0% 0.0%
Common effect model -0.00 [-0.00; 0.00] 100.0% --
Random effects model -0.00 [-0.00; 0.00] -- 100.0%
Heterogeneity: I^2 = 0%, τ^2 = 0, p = 0.77
-2
-1
0
1
2

Study	Sampling	Outcome	Subjects	Estimate [95% CI]
Zhang, Lei, et al., 2022	within 3 d before del	7 y	206	0.005 [-0.041; 0.051]
Bloom et al., 2022	8-13 w gest	4-8 y	661	0.090 [-0.538; 0.717]
Chen et al., 2019	del	5 y	404	-0.002 [-0.005; 0.002]
Vrijheid et al., 2020	during gest	6-11 years	1301	-0.823 [-2.469; 0.823]
**Summary**				**-0.393 [-1.241; 0.455]**

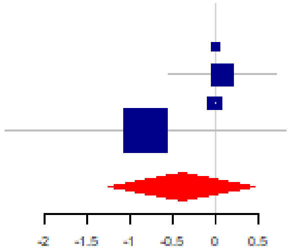

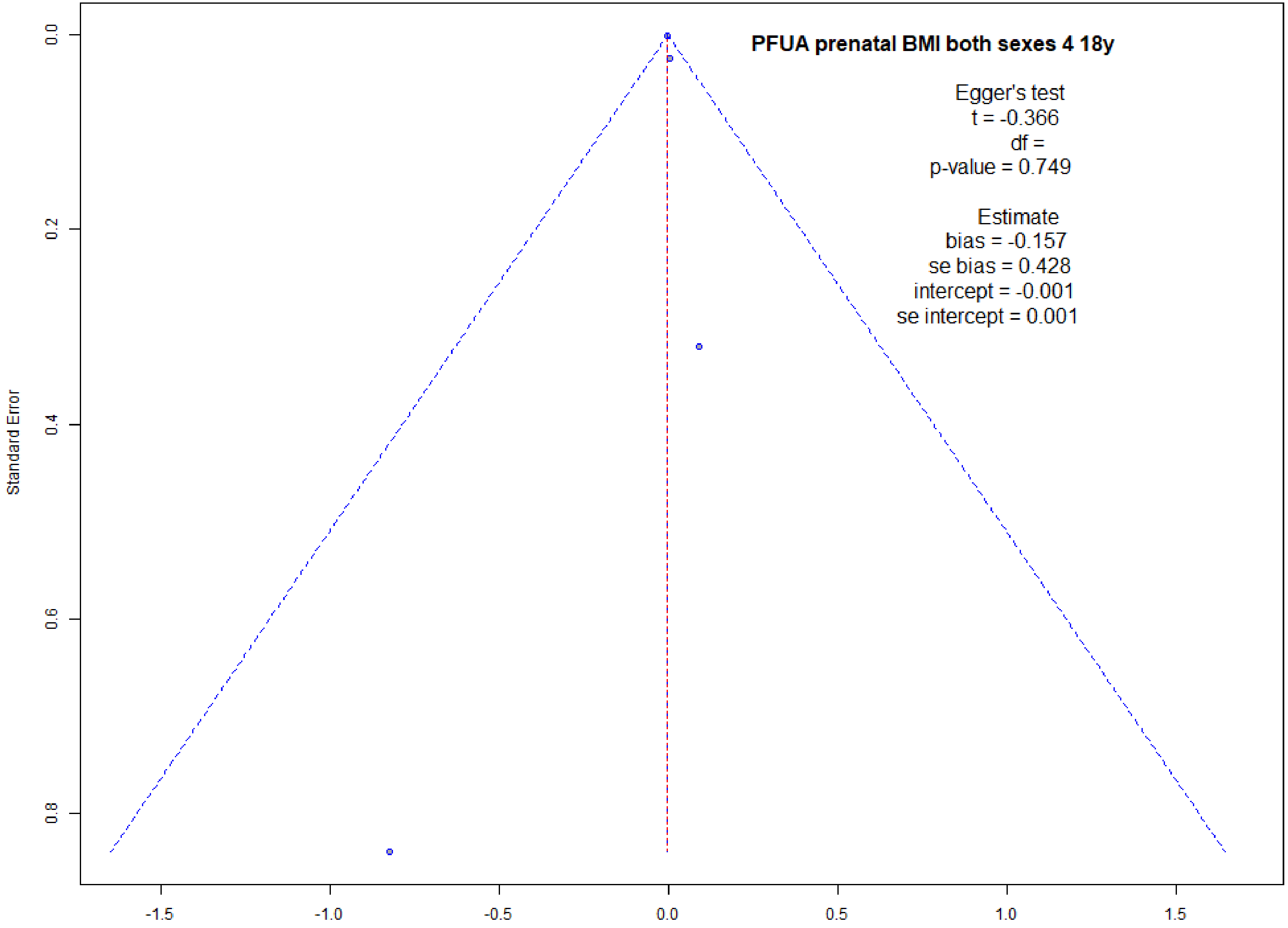
PFUA prenatal BMI both sexes 4 18y
Egger's test
t = -0.366
df =
p-value = 0.749
Estimate
bias = -0.157
se bias = 0.428
intercept = -0.001
se intercept = 0.001
Standard Error
0.0
0.2
0.4
0.6
0.8
-1.5
-1.0
-0.5
0.0
0.5
1.0
1.5

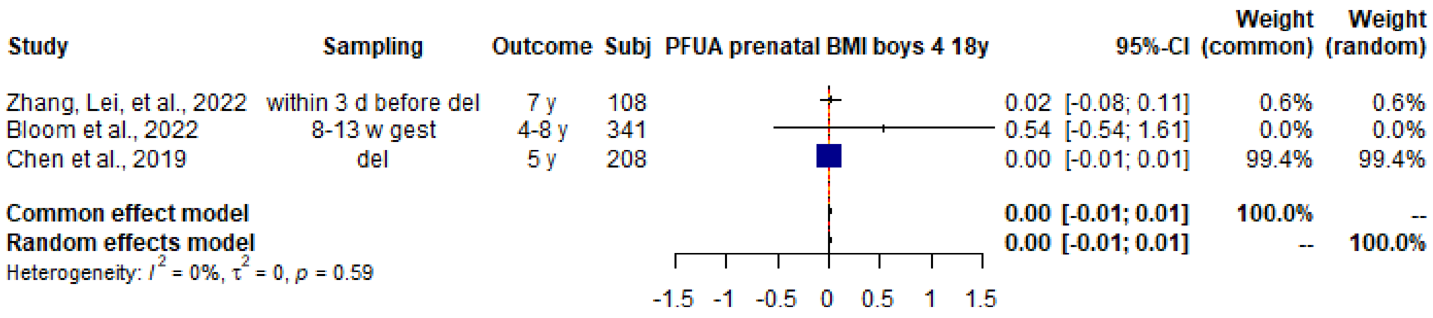
Study
Sampling
Outcome
Subj
PFUA prenatal BMI boys 4 18y
95%-CI
Weight (common)
Weight (random)
Zhang, Lei, et al., 2022 within 3 d before del 7 y 108 0.02 [-0.08; 0.11] 0.6% 0.6%
Bloom et al., 2022 8-13 w gest 4-8 y 341 0.54 [-0.54; 1.61] 0.0% 0.0%
Chen et al., 2019 del 5 y 208 0.00 [-0.01; 0.01] 99.4% 99.4%
Common effect model 0.00 [-0.01; 0.01] 100.0% --
Random effects model 0.00 [-0.01; 0.01] -- 100.0%
Heterogeneity: I^2 = 0%, τ^2 = 0, p = 0.59
-1.5 -1 -0.5 0 0.5 1 1.5

Study	Sampling	Outcome	Subjects	Estimate [95% CI]
Zhang, Lei, et al., 2022	within 3 d before del	7 y	108	0.019 [-0.077; 0.115]
Bloom et al., 2022	8-13 w gest	4-8 y	341	0.538 [-0.538; 1.613]
Chen et al., 2019	del	5 y	208	0.002 [-0.006; 0.010]
**Summary**				**0.283 [-0.276; 0.841]**

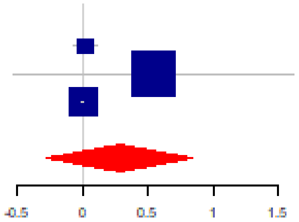

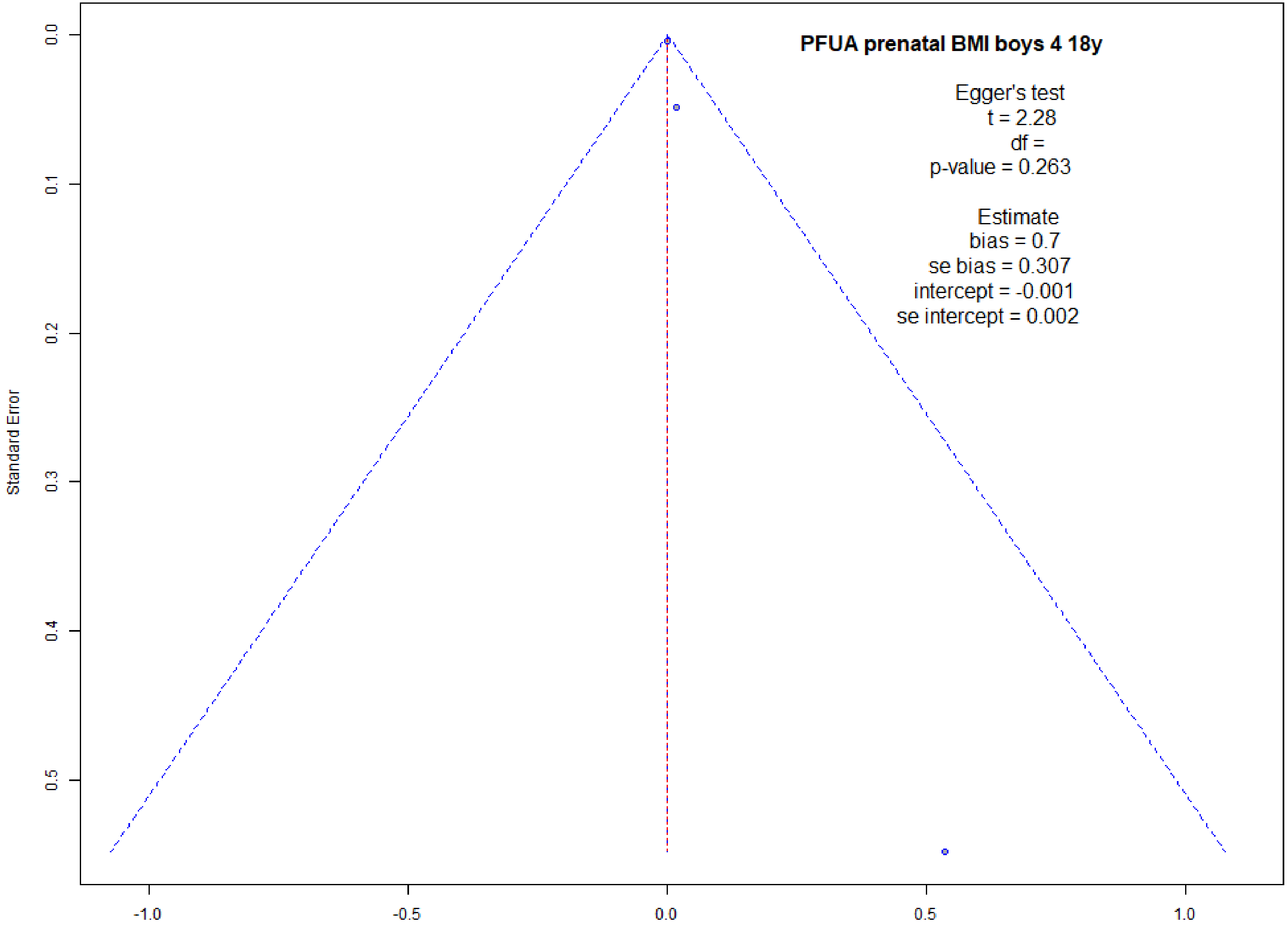
PFUA prenatal BMI boys 4 18y
Egger's test
t = 2.28
df =
p-value = 0.263
Estimate
bias = 0.7
se bias = 0.307
intercept = -0.001
se intercept = 0.002
Standard Error
0.0
0.1
0.2
0.3
0.4
0.5
-1.0
-0.5
0.0
0.5
1.0

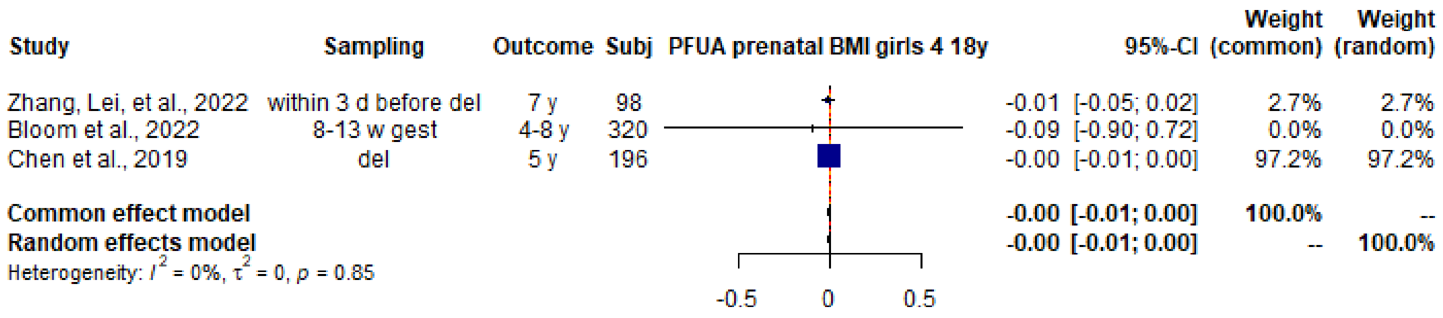
Study
Sampling
Outcome
Subj
PFUA prenatal BMI girls 4 18y
95%-CI
Weight (common)
Weight (random)
Zhang, Lei, et al., 2022 within 3 d before del 7 y 98 -0.01 [-0.05; 0.02] 2.7% 2.7%
Bloom et al., 2022 8-13 w gest 4-8 y 320 -0.09 [-0.90; 0.72] 0.0% 0.0%
Chen et al., 2019 del 5 y 196 -0.00 [-0.01; 0.00] 97.2% 97.2%
Common effect model -0.00 [-0.01; 0.00] 100.0% --
Random effects model -0.00 [-0.01; 0.00] -- 100.0%
Heterogeneity: I^2 = 0%, τ^2 = 0, p = 0.85
-0.5
0
0.5

Study	Sampling	Outcome	Subjects	Estimate [95% CI]
Zhang, Lei, et al., 2022	within 3 d before del	7 y	98	-0.013 [-0.045; 0.019]
Bloom et al., 2022	8-13 w gest	4-8 y	320	-0.090 [-0.896; 0.717]
Chen et al., 2019	del	5 y	196	-0.004 [-0.010; 0.001]
**Summary**				**-0.050 [-0.470; 0.370]**

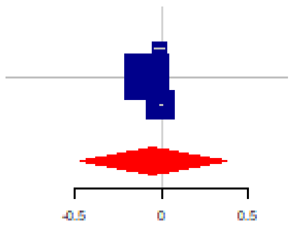

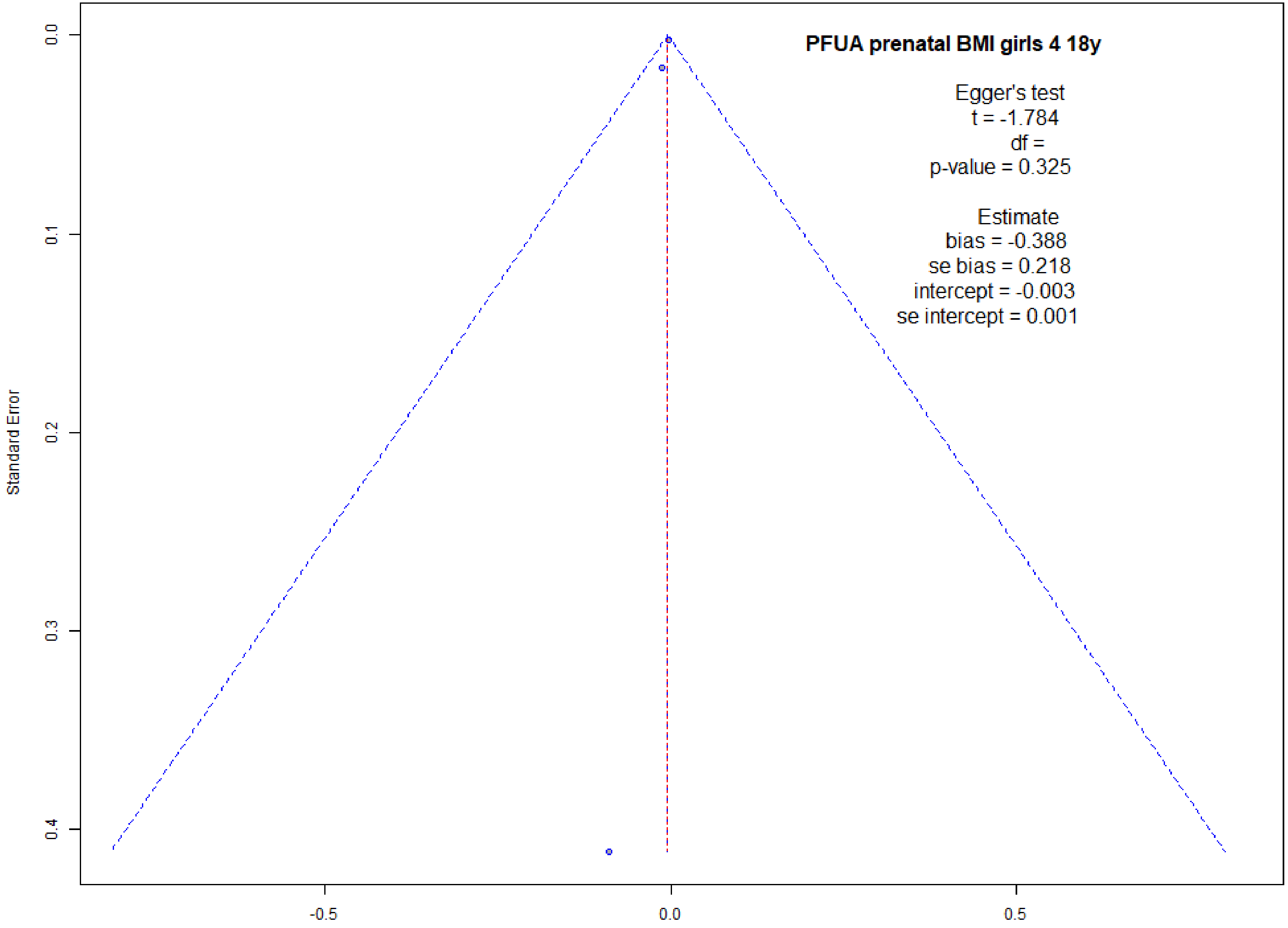
PFUA prenatal BMI girls 4 18y
Egger's test
t = -1.784
df =
p-value = 0.325
Estimate
bias = -0.388
se bias = 0.218
intercept = -0.003
se intercept = 0.001
Standard Error
0.0
0.1
0.2
0.3
0.4
-0.5
0.0
0.5

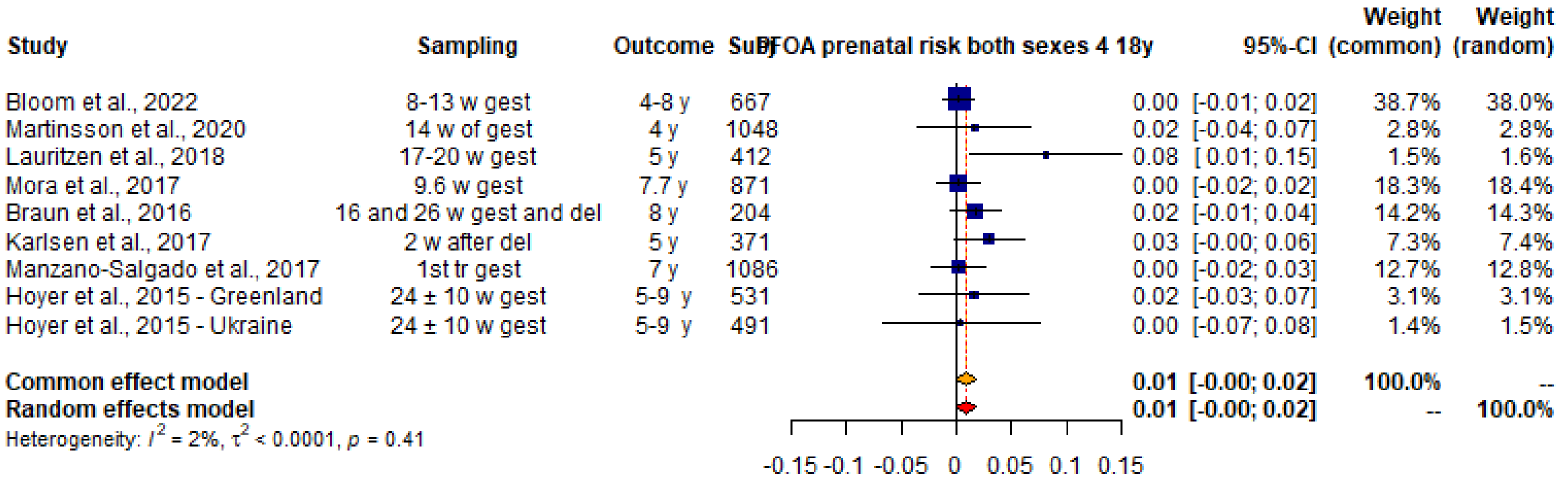
Study
Sampling
Outcome
Sub
PFOA prenatal risk both sexes 4 18y
95%-CI
Weight (common)
Weight (random)
Bloom et al., 2022 | 8-13 w gest | 4-8 y | 667 | 0.00 [-0.01; 0.02] | 38.7% | 38.0%
Martinsson et al., 2020 | 14 w of gest | 4 y | 1048 | 0.02 [-0.04; 0.07] | 2.8% | 2.8%
Lauritzen et al., 2018 | 17-20 w gest | 5 y | 412 | 0.08 [0.01; 0.15] | 1.5% | 1.6%
Mora et al., 2017 | 9.6 w gest | 7.7 y | 871 | 0.00 [-0.02; 0.02] | 18.3% | 18.4%
Braun et al., 2016 | 16 and 26 w gest and del | 8 y | 204 | 0.02 [-0.01; 0.04] | 14.2% | 14.3%
Karlsen et al., 2017 | 2 w after del | 5 y | 371 | 0.03 [-0.00; 0.06] | 7.3% | 7.4%
Manzano-Salgado et al., 2017 | 1st tr gest | 7 y | 1086 | 0.00 [-0.02; 0.03] | 12.7% | 12.8%
Hoyer et al., 2015 - Greenland | 24 ± 10 w gest | 5-9 y | 531 | 0.02 [-0.03; 0.07] | 3.1% | 3.1%
Hoyer et al., 2015 - Ukraine | 24 ± 10 w gest | 5-9 y | 491 | 0.00 [-0.07; 0.08] | 1.4% | 1.5%
Common effect model | 0.01 [-0.00; 0.02] | 100.0% | --
Random effects model | 0.01 [-0.00; 0.02] | -- | 100.0%
Heterogeneity: I^2 = 2%, τ^2 < 0.0001, p = 0.41
-0.15 -0.1 -0.05 0 0.05 0.1 0.15

Study	Sampling	Outcome	Subjects	Estimate [95% CI]
Bloom et al., 2022	8-13 w gest	4-8 y	667	0.002 [-0.012; 0.016]
Martinsson et al., 2020	14 w of gest	4 y	1048	0.016 [-0.035; 0.068]
Lauritzen et al., 2018	17-20 w gest	5 y	412	0.081 [0.012; 0.151]
Mora et al., 2017	9.6 w gest	7.7 y	871	0.002 [-0.018; 0.022]
Braun et al., 2016	16 and 26 w gest and del	8 y	204	0.017 [-0.006; 0.040]
Karlsen et al., 2017	2 w after del	5 y	371	0.030 [-0.002; 0.062]
Manzano-Salgado et al., 2017	1st tr gest	7 y	1086	0.002 [-0.022; 0.026]
Hoyer et al., 2015 - Greenland	24 ± 10 w gest	5-9 y	531	0.016 [-0.034; 0.065]
Hoyer et al., 2015 - Ukraine	24 ± 10 w gest	5-9 y	491	0.004 [-0.068; 0.076]
**Summary**				**0.014 [-0.001; 0.029]**

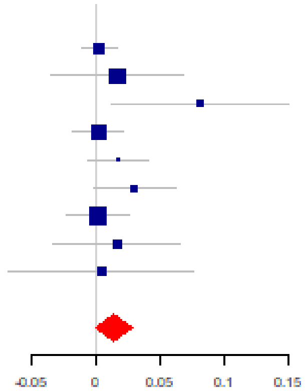

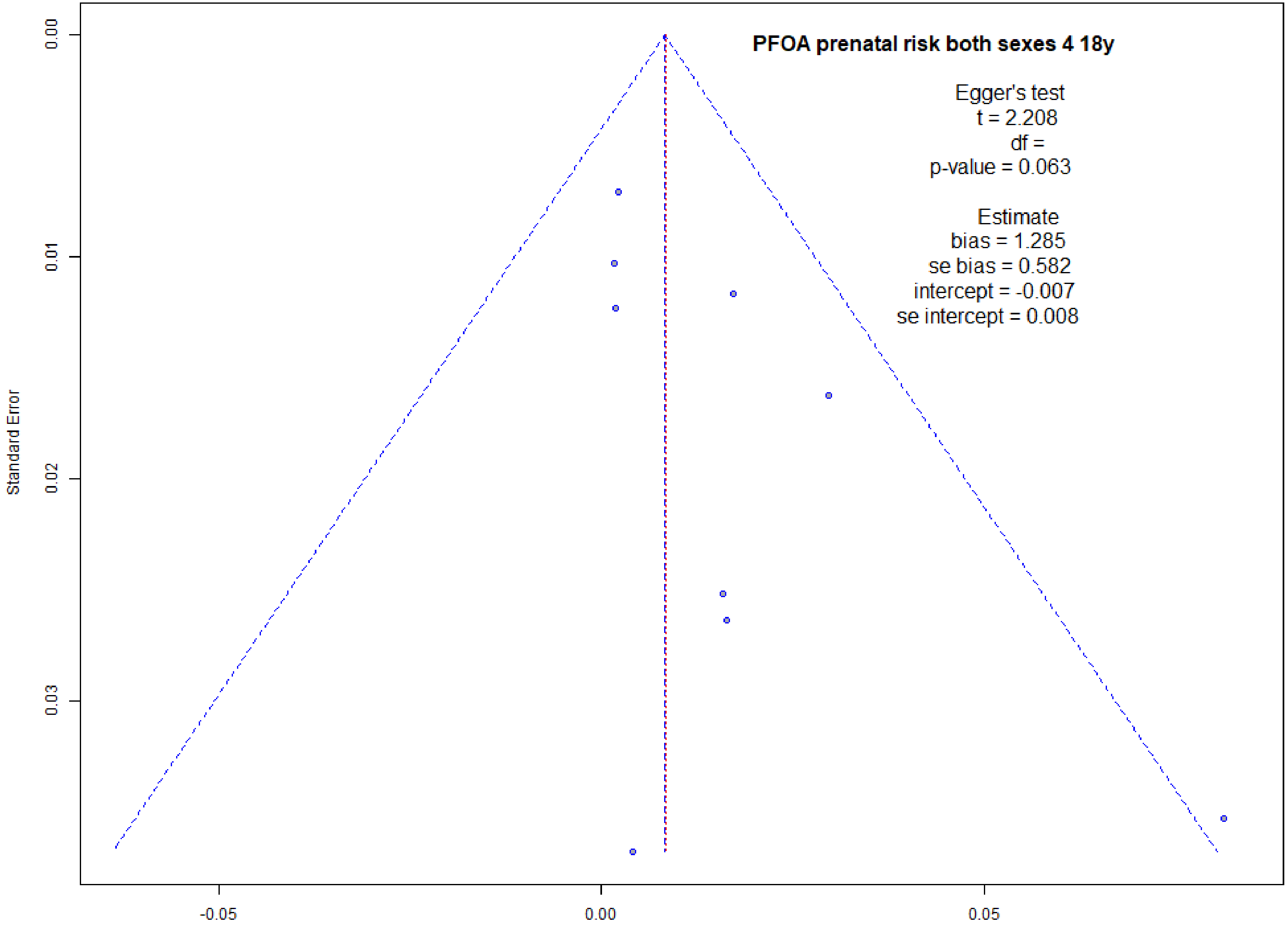
PFOA prenatal risk both sexes 4 18y
Egger's test
t = 2.208
df =
p-value = 0.063
Estimate
bias = 1.285
se bias = 0.582
intercept = -0.007
se intercept = 0.008
Standard Error
0.00
0.01
0.02
0.03
-0.05
0.00
0.05

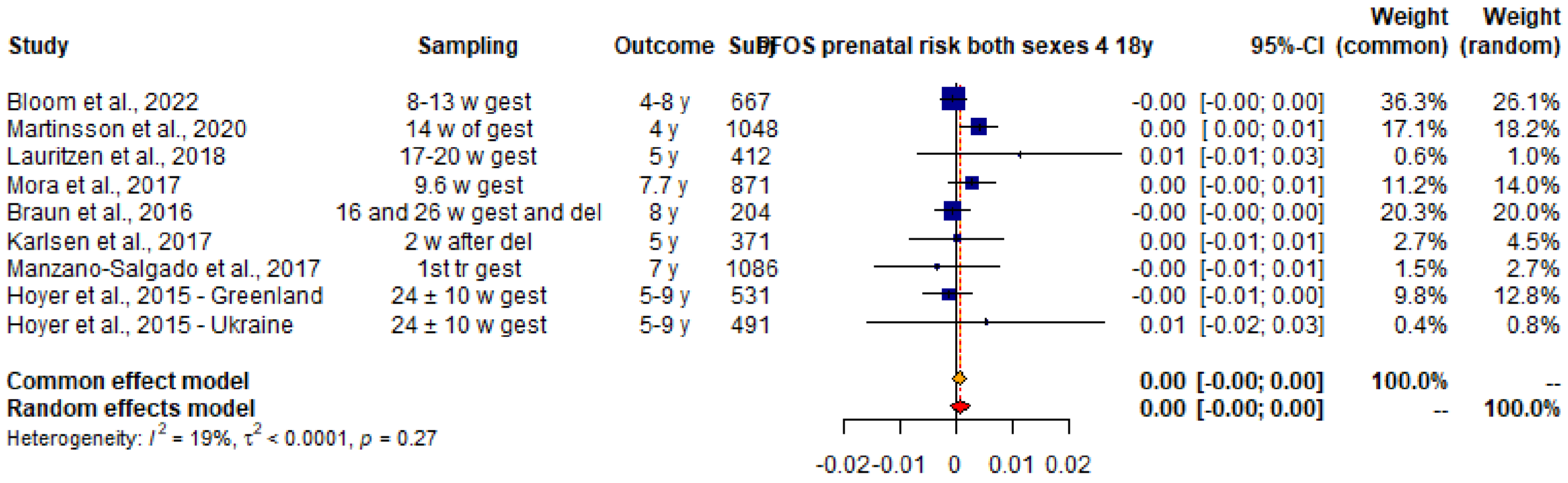
Study
Sampling
Outcome
Subj
PFOS prenatal risk both sexes 4 18y
95%-CI
Weight (common)
Weight (random)
Bloom et al., 2022 | 8-13 w gest | 4-8 y | 667 | -0.00 [-0.00; 0.00] | 36.3% | 26.1%
Martinsson et al., 2020 | 14 w of gest | 4 y | 1048 | 0.00 [0.00; 0.01] | 17.1% | 18.2%
Lauritzen et al., 2018 | 17-20 w gest | 5 y | 412 | 0.01 [-0.01; 0.03] | 0.6% | 1.0%
Mora et al., 2017 | 9.6 w gest | 7.7 y | 871 | 0.00 [-0.00; 0.01] | 11.2% | 14.0%
Braun et al., 2016 | 16 and 26 w gest and del | 8 y | 204 | -0.00 [-0.00; 0.00] | 20.3% | 20.0%
Karlsen et al., 2017 | 2 w after del | 5 y | 371 | 0.00 [-0.01; 0.01] | 2.7% | 4.5%
Manzano-Salgado et al., 2017 | 1st tr gest | 7 y | 1086 | -0.00 [-0.01; 0.01] | 1.5% | 2.7%
Hoyer et al., 2015 - Greenland | 24 ± 10 w gest | 5-9 y | 531 | -0.00 [-0.01; 0.00] | 9.8% | 12.8%
Hoyer et al., 2015 - Ukraine | 24 ± 10 w gest | 5-9 y | 491 | 0.01 [-0.02; 0.03] | 0.4% | 0.8%
Common effect model | 0.00 [-0.00; 0.00] | 100.0% | --
Random effects model | 0.00 [-0.00; 0.00] | -- | 100.0%
Heterogeneity: I^2 = 19%, τ^2 < 0.0001, p = 0.27
-0.02 -0.01 0 0.01 0.02

Study	Sampling	Outcome	Subjects	Estimate [95% CI]
Bloom et al., 2022	8-13 w gest	4-8 y	667	-0.001 [-0.003; 0.002]
Martinsson et al., 2020	14 w of gest	4 y	1048	0.004 [0.001; 0.007]
Lauritzen et al., 2018	17-20 w gest	5 y	412	0.011 [-0.007; 0.029]
Mora et al., 2017	9.6 w gest	7.7 y	871	0.003 [-0.001; 0.007]
Braun et al., 2016	16 and 26 w gest and del	8 y	204	-0.001 [-0.004; 0.002]
Karlsen et al., 2017	2 w after del	5 y	371	0.000 [-0.008; 0.008]
Manzano-Salgado et al., 2017	1st tr gest	7 y	1086	-0.003 [-0.014; 0.008]
Hoyer et al., 2015 - Greenland	24 ± 10 w gest	5-9 y	531	-0.002 [-0.006; 0.003]
Hoyer et al., 2015 - Ukraine	24 ± 10 w gest	5-9 y	491	0.005 [-0.016; 0.026]
**Summary**				**0.002 [-0.002; 0.005]**

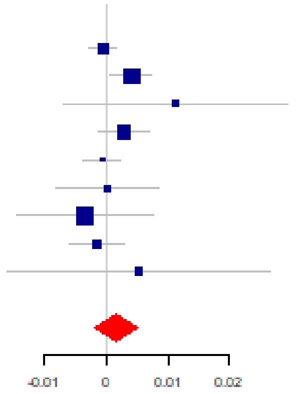

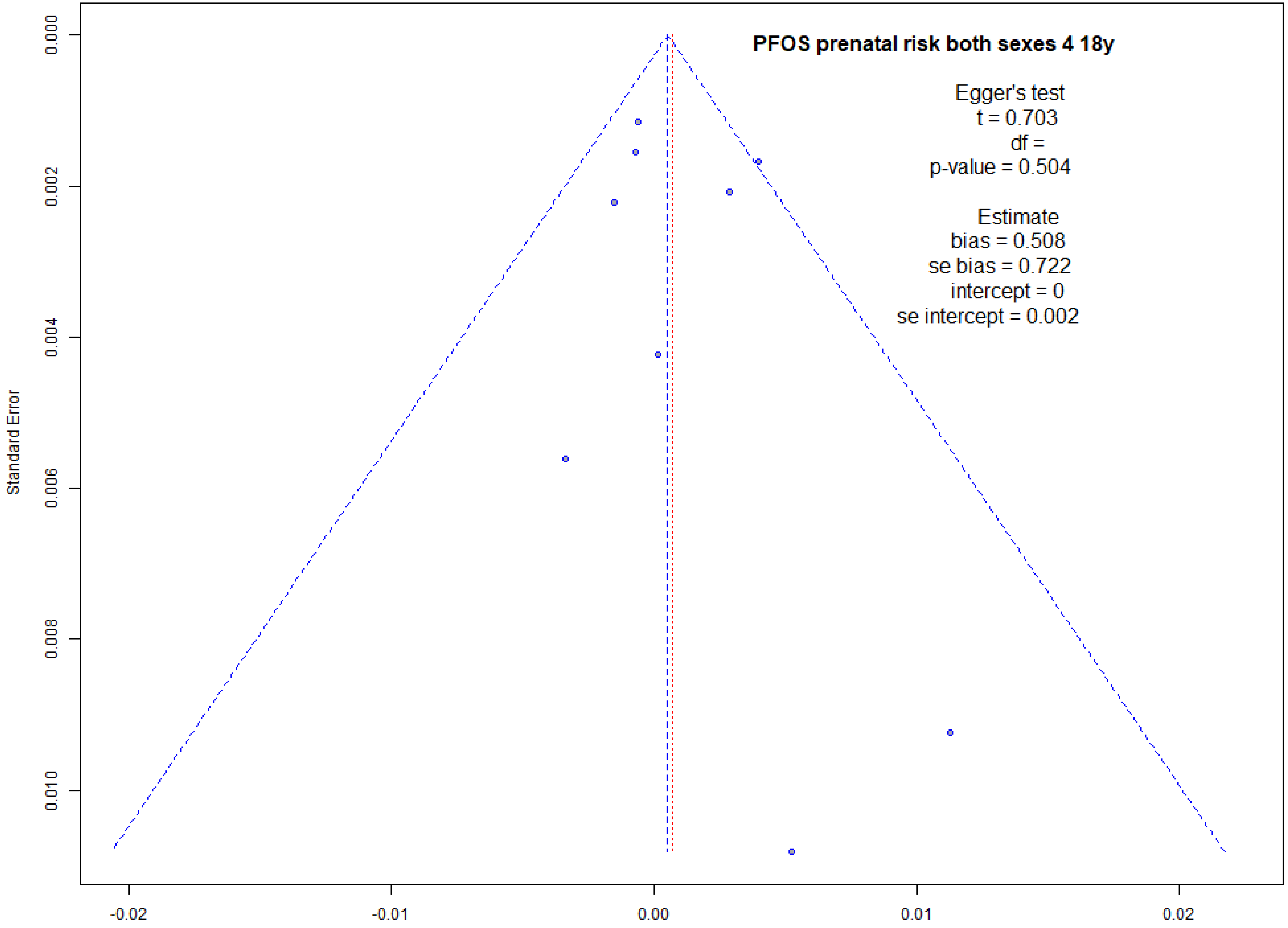
PFOS prenatal risk both sexes 4 18y
Egger's test
t = 0.703
df =
p-value = 0.504
Estimate
bias = 0.508
se bias = 0.722
intercept = 0
se intercept = 0.002
Standard Error
0.000
0.002
0.004
0.006
0.008
0.010
-0.02
-0.01
0.00
0.01
0.02

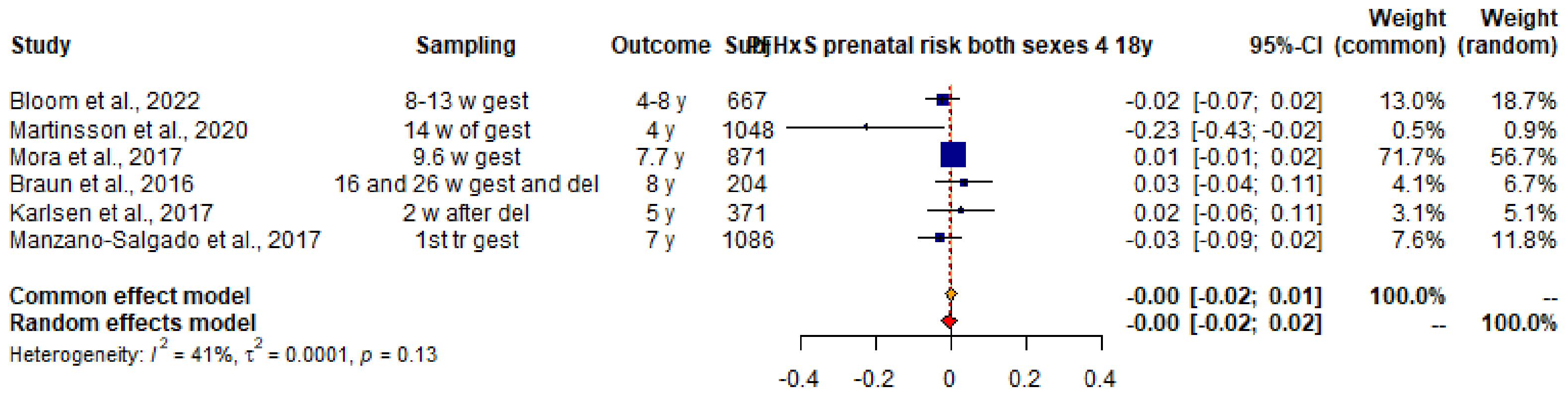
Study
Sampling
Outcome
Subj
PFHxS prenatal risk both sexes 4 18y
95%-CI
Weight (common)
Weight (random)
Bloom et al., 2022 | 8-13 w gest | 4-8 y | 667 | -0.02 [-0.07; 0.02] | 13.0% | 18.7%
Martinsson et al., 2020 | 14 w of gest | 4 y | 1048 | -0.23 [-0.43; -0.02] | 0.5% | 0.9%
Mora et al., 2017 | 9.6 w gest | 7.7 y | 871 | 0.01 [-0.01; 0.02] | 71.7% | 56.7%
Braun et al., 2016 | 16 and 26 w gest and del | 8 y | 204 | 0.03 [-0.04; 0.11] | 4.1% | 6.7%
Karlsen et al., 2017 | 2 w after del | 5 y | 371 | 0.02 [-0.06; 0.11] | 3.1% | 5.1%
Manzano-Salgado et al., 2017 | 1st tr gest | 7 y | 1086 | -0.03 [-0.09; 0.02] | 7.6% | 11.8%
Common effect model | -0.00 [-0.02; 0.01] | 100.0% | --
Random effects model | -0.00 [-0.02; 0.02] | -- | 100.0%
Heterogeneity: I^2 = 41%, τ^2 = 0.0001, p = 0.13
-0.4
-0.2
0
0.2
0.4

Study	Sampling	Outcome	Subjects	Estimate [95% CI]
Bloom et al., 2022	8-13 w gest	4-8 y	667	-0.024 [-0.066; 0.018]
Martinsson et al., 2020	14 w of gest	4 y	1048	-0.227 [-0.434; -0.019]
Mora et al., 2017	9.6 w gest	7.7 y	871	0.006 [-0.012; 0.024]
Braun et al., 2016	16 and 26 w gest and del	8 y	204	0.033 [-0.041; 0.108]
Karlsen et al., 2017	2 w after del	5 y	371	0.024 [-0.062; 0.111]
Manzano-Salgado et al., 2017	1st tr gest	7 y	1086	-0.031 [-0.086; 0.024]
**Summary**				**-0.063 [-0.120; -0.005]**

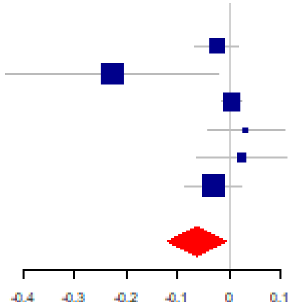

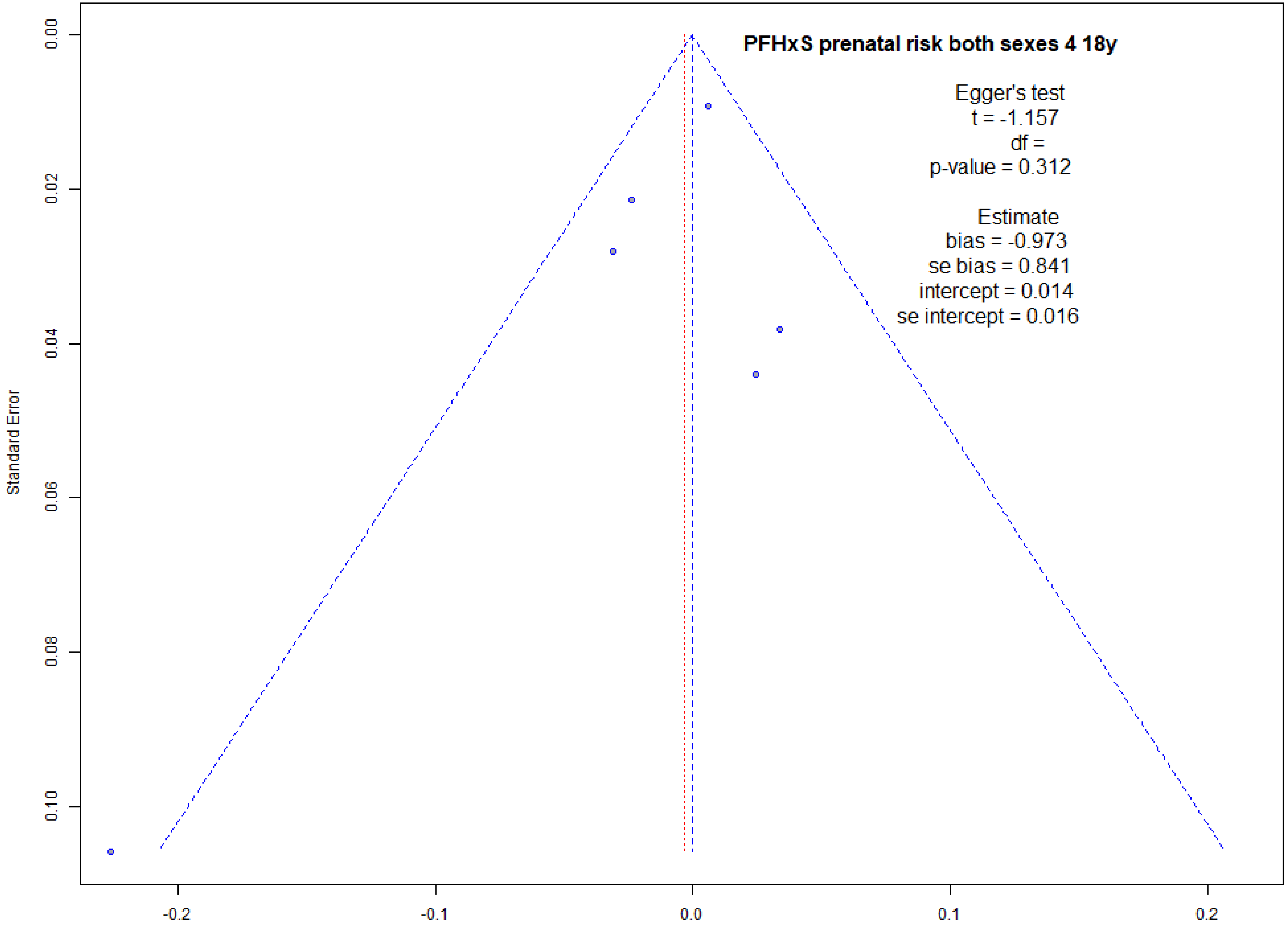
PFHxS prenatal risk both sexes 4 18y
Egger's test
t = -1.157
df =
p-value = 0.312
Estimate
bias = -0.973
se bias = 0.841
intercept = 0.014
se intercept = 0.016
Standard Error
0.00
0.02
0.04
0.06
0.08
0.10
-0.2
-0.1
0.0
0.1
0.2

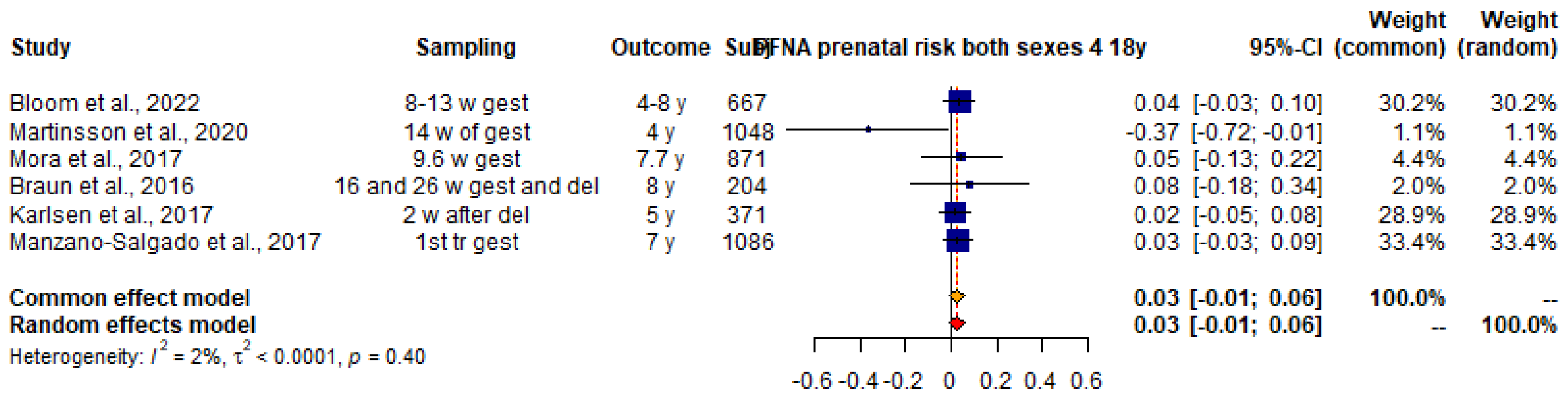
Study
Sampling
Outcome
Subj
PFNA prenatal risk both sexes 4 18y
95%-CI
Weight (common)
Weight (random)
Bloom et al., 2022 | 8-13 w gest | 4-8 y | 667 | 0.04 [-0.03; 0.10] | 30.2% | 30.2%
Martinsson et al., 2020 | 14 w of gest | 4 y | 1048 | -0.37 [-0.72; -0.01] | 1.1% | 1.1%
Mora et al., 2017 | 9.6 w gest | 7.7 y | 871 | 0.05 [-0.13; 0.22] | 4.4% | 4.4%
Braun et al., 2016 | 16 and 26 w gest and del | 8 y | 204 | 0.08 [-0.18; 0.34] | 2.0% | 2.0%
Karlsen et al., 2017 | 2 w after del | 5 y | 371 | 0.02 [-0.05; 0.08] | 28.9% | 28.9%
Manzano-Salgado et al., 2017 | 1st tr gest | 7 y | 1086 | 0.03 [-0.03; 0.09] | 33.4% | 33.4%
Common effect model | 0.03 [-0.01; 0.06] | 100.0% | --
Random effects model | 0.03 [-0.01; 0.06] | -- | 100.0%
Heterogeneity: I^2 = 2%, τ^2 < 0.0001, p = 0.40
-0.6 -0.4 -0.2 0 0.2 0.4 0.6

Study	Sampling	Outcome	Subjects	Estimate [95% CI]
Bloom et al., 2022	8-13 w gest	4-8 y	667	0.036 [-0.030; 0.103]
Martinsson et al., 2020	14 w of gest	4 y	1048	-0.368 [-0.724; -0.013]
Mora et al., 2017	9.6 w gest	7.7 y	871	0.045 [-0.128; 0.219]
Braun et al., 2016	16 and 26 w gest and del	8 y	204	0.080 [-0.181; 0.341]
Karlsen et al., 2017	2 w after del	5 y	371	0.017 [-0.051; 0.085]
Manzano-Salgado et al., 2017	1st tr gest	7 y	1086	0.028 [-0.035; 0.092]
**Summary**				**-0.063 [-0.161; 0.034]**

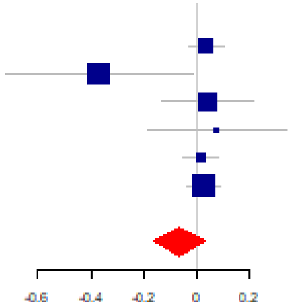

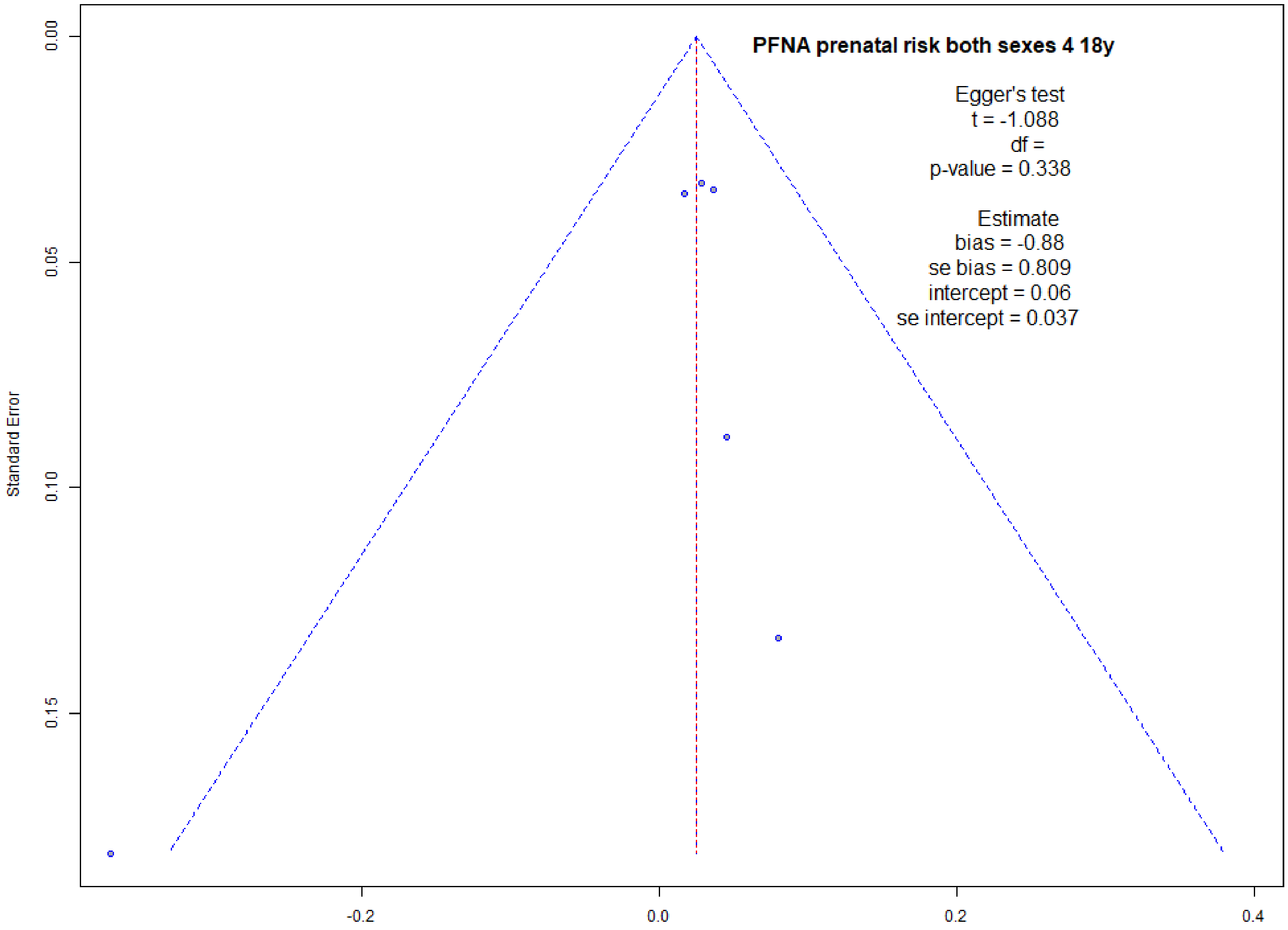
PFNA prenatal risk both sexes 4 18y
Egger's test
t = -1.088
df =
p-value = 0.338
Estimate
bias = -0.88
se bias = 0.809
intercept = 0.06
se intercept = 0.037
Standard Error
0.00
0.05
0.10
0.15
-0.2
0.0
0.2
0.4

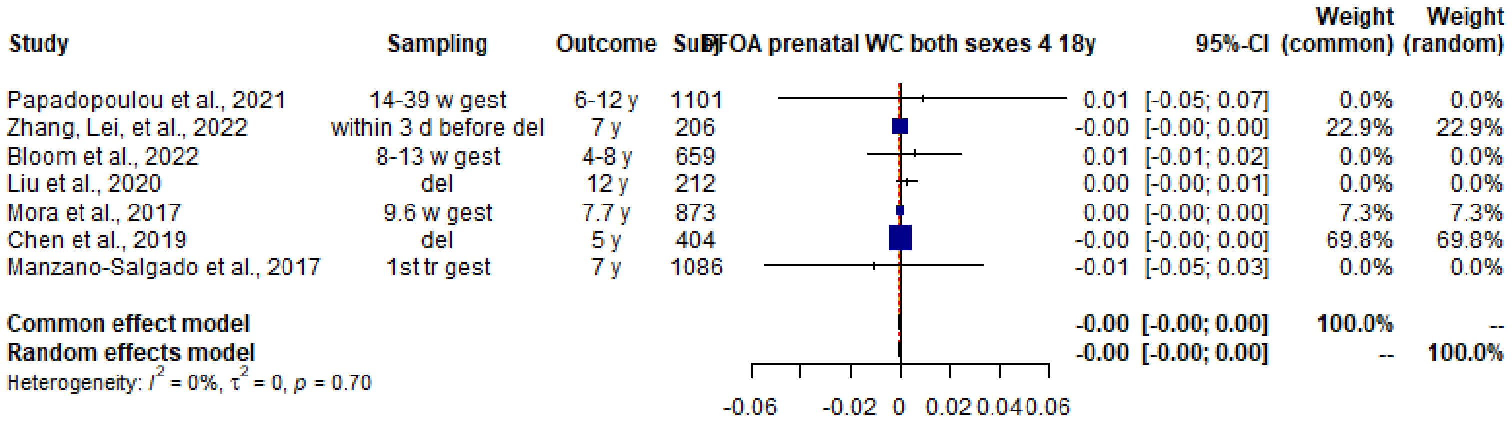
Study
Sampling
Outcome
Sub
PFOA prenatal WC both sexes 4 18y
95%-CI
Weight (common)
Weight (random)
Papadopoulou et al., 2021 | 14-39 w gest | 6-12 y | 1101 | 0.01 | [-0.05; 0.07] | 0.0% | 0.0%
Zhang, Lei, et al., 2022 | within 3 d before del | 7 y | 206 | -0.00 | [-0.00; 0.00] | 22.9% | 22.9%
Bloom et al., 2022 | 8-13 w gest | 4-8 y | 659 | 0.01 | [-0.01; 0.02] | 0.0% | 0.0%
Liu et al., 2020 | del | 12 y | 212 | 0.00 | [-0.00; 0.01] | 0.0% | 0.0%
Mora et al., 2017 | 9.6 w gest | 7.7 y | 873 | 0.00 | [-0.00; 0.00] | 7.3% | 7.3%
Chen et al., 2019 | del | 5 y | 404 | -0.00 | [-0.00; 0.00] | 69.8% | 69.8%
Manzano-Salgado et al., 2017 | 1st tr gest | 7 y | 1086 | -0.01 | [-0.05; 0.03] | 0.0% | 0.0%
Common effect model | -0.00 | [-0.00; 0.00] | 100.0% | --
Random effects model | -0.00 | [-0.00; 0.00] | -- | 100.0%
Heterogeneity: I^2 = 0%, τ^2 = 0, p = 0.70
-0.06
-0.02
0
0.02
0.04
0.06

Study	Sampling	Outcome	Subjects	Estimate [95% CI]
Papadopoulou et al., 2021	14-39 w gest	6-12 y	1101	0.009 [-0.049; 0.067]
Zhang, Lei, et al., 2022	within 3 d before del	7 y	206	0.000 [0.000; 0.000]
Bloom et al., 2022	8-13 w gest	4-8 y	659	0.006 [-0.013; 0.025]
Liu et al., 2020	del	12 y	212	0.003 [-0.001; 0.006]
Mora et al., 2017	9.6 w gest	7.7 y	873	0.000 [0.000; 0.000]
Chen et al., 2019	del	5 y	404	0.000 [0.000; 0.000]
Manzano-Salgado et al., 2017	1st tr gest	7 y	1086	-0.010 [-0.054; 0.034]
**Summary**				**0.001 [-0.017; 0.018]**

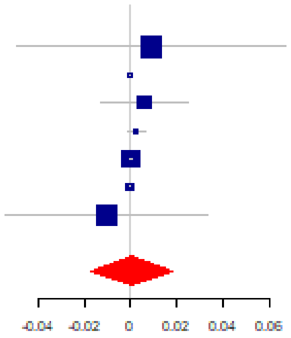

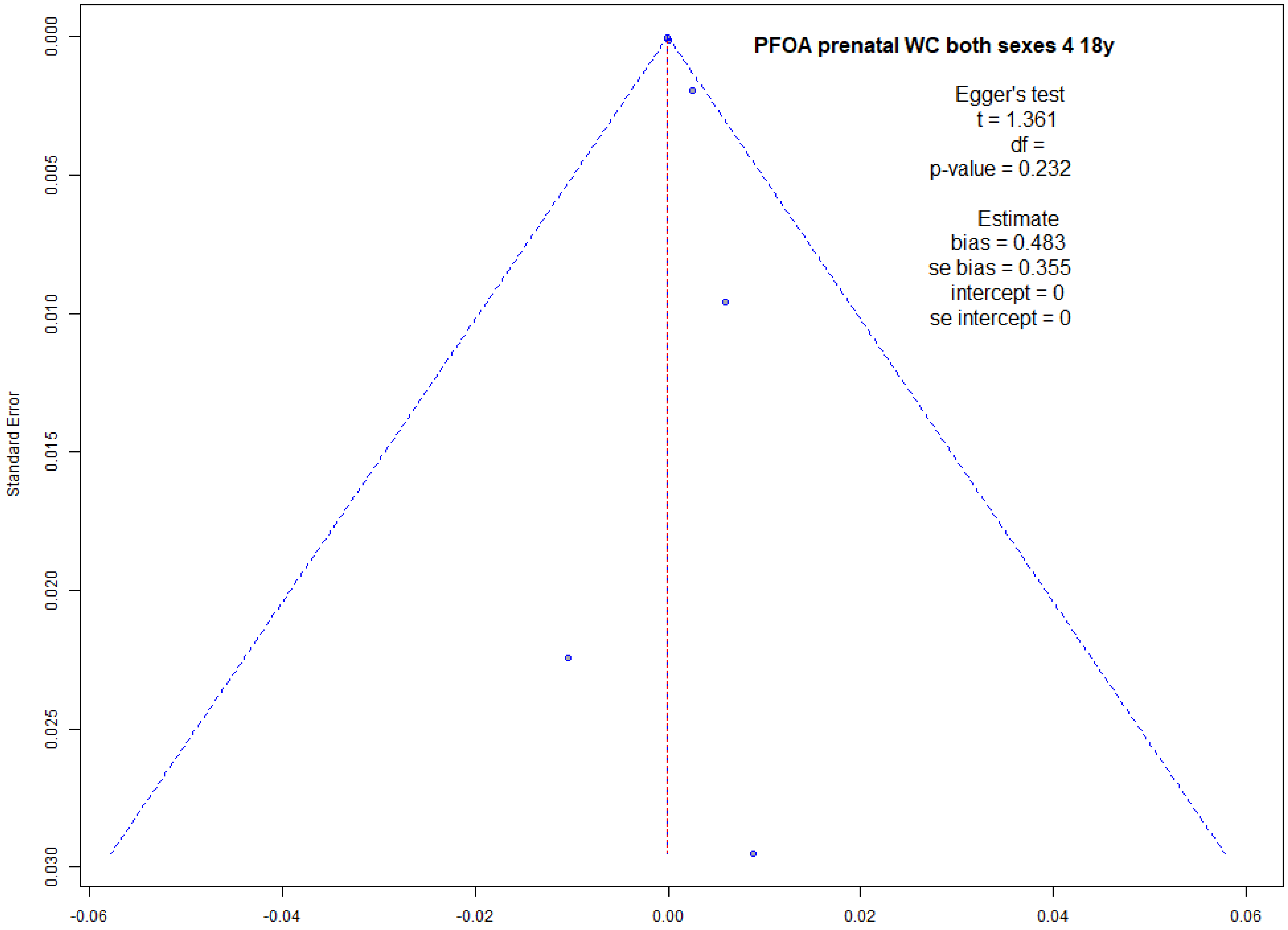
PFOA prenatal WC both sexes 4 18y
Egger's test
t = 1.361
df =
p-value = 0.232
Estimate
bias = 0.483
se bias = 0.355
intercept = 0
se intercept = 0
Standard Error
0.000
0.005
0.010
0.015
0.020
0.025
0.030
-0.06
-0.04
-0.02
0.00
0.02
0.04
0.06

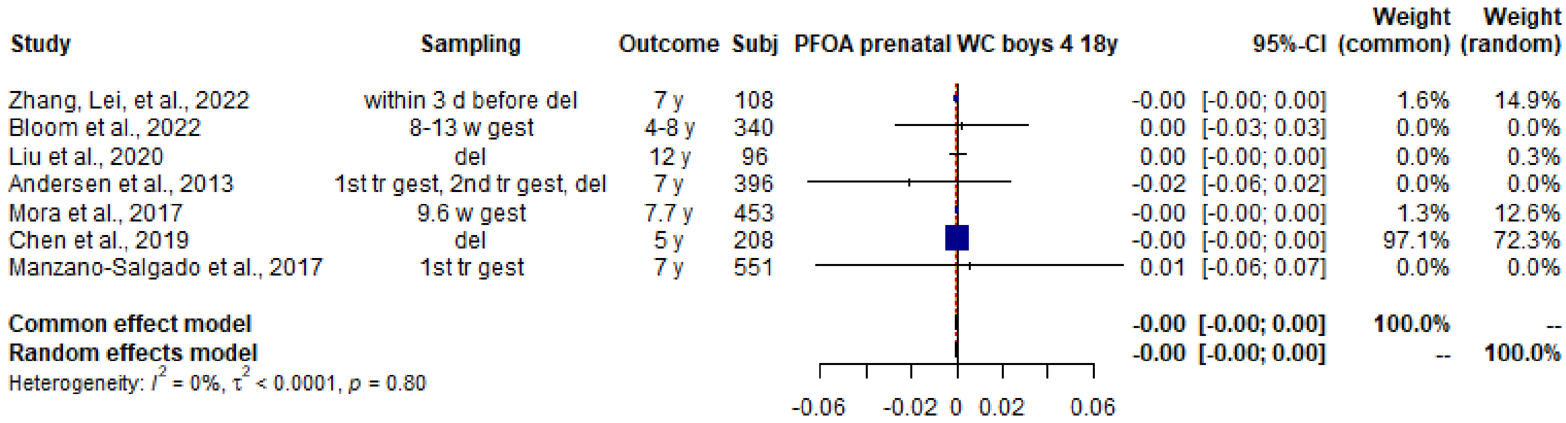
Study
Sampling
Outcome
Subj
PFOA prenatal WC boys 4 18y
95%-CI
Weight (common)
Weight (random)
Zhang, Lei, et al., 2022 within 3 d before del 7 y 108 -0.00 [-0.00; 0.00] 1.6% 14.9%
Bloom et al., 2022 8-13 w gest 4-8 y 340 0.00 [-0.03; 0.03] 0.0% 0.0%
Liu et al., 2020 del 12 y 96 0.00 [-0.00; 0.00] 0.0% 0.3%
Andersen et al., 2013 1st tr gest, 2nd tr gest, del 7 y 396 -0.02 [-0.06; 0.02] 0.0% 0.0%
Mora et al., 2017 9.6 w gest 7.7 y 453 -0.00 [-0.00; 0.00] 1.3% 12.6%
Chen et al., 2019 del 5 y 208 -0.00 [-0.00; 0.00] 97.1% 72.3%
Manzano-Salgado et al., 2017 1st tr gest 7 y 551 0.01 [-0.06; 0.07] 0.0% 0.0%
Common effect model -0.00 [-0.00; 0.00] 100.0% --
Random effects model -0.00 [-0.00; 0.00] -- 100.0%
Heterogeneity: I^2 = 0%, τ^2 < 0.0001, p = 0.80
-0.06
-0.02
0
0.02
0.06

Study	Sampling	Outcome	Subjects	Estimate [95% CI]
Zhang, Lei, et al., 2022	within 3 d before del	7 y	108	0.000 [-0.001; 0.000]
Bloom et al., 2022	8-13 w gest	4-8 y	340	0.002 [-0.027; 0.031]
Liu et al., 2020	del	12 y	96	0.000 [-0.003; 0.003]
Andersen et al., 2013	1st tr gest, 2nd tr gest, del	7 y	396	-0.021 [-0.065; 0.023]
Mora et al., 2017	9.6 w gest	7.7 y	453	0.000 [-0.001; 0.000]
Chen et al., 2019	del	5 y	208	0.000 [0.000; 0.000]
Manzano-Salgado et al., 2017	1st tr gest	7 y	551	0.005 [-0.062; 0.072]
**Summary**				**-0.002 [-0.022; 0.017]**

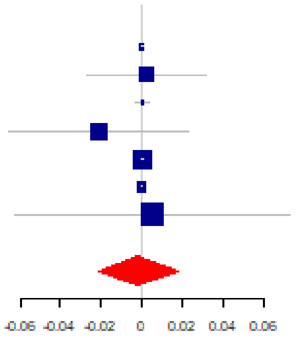

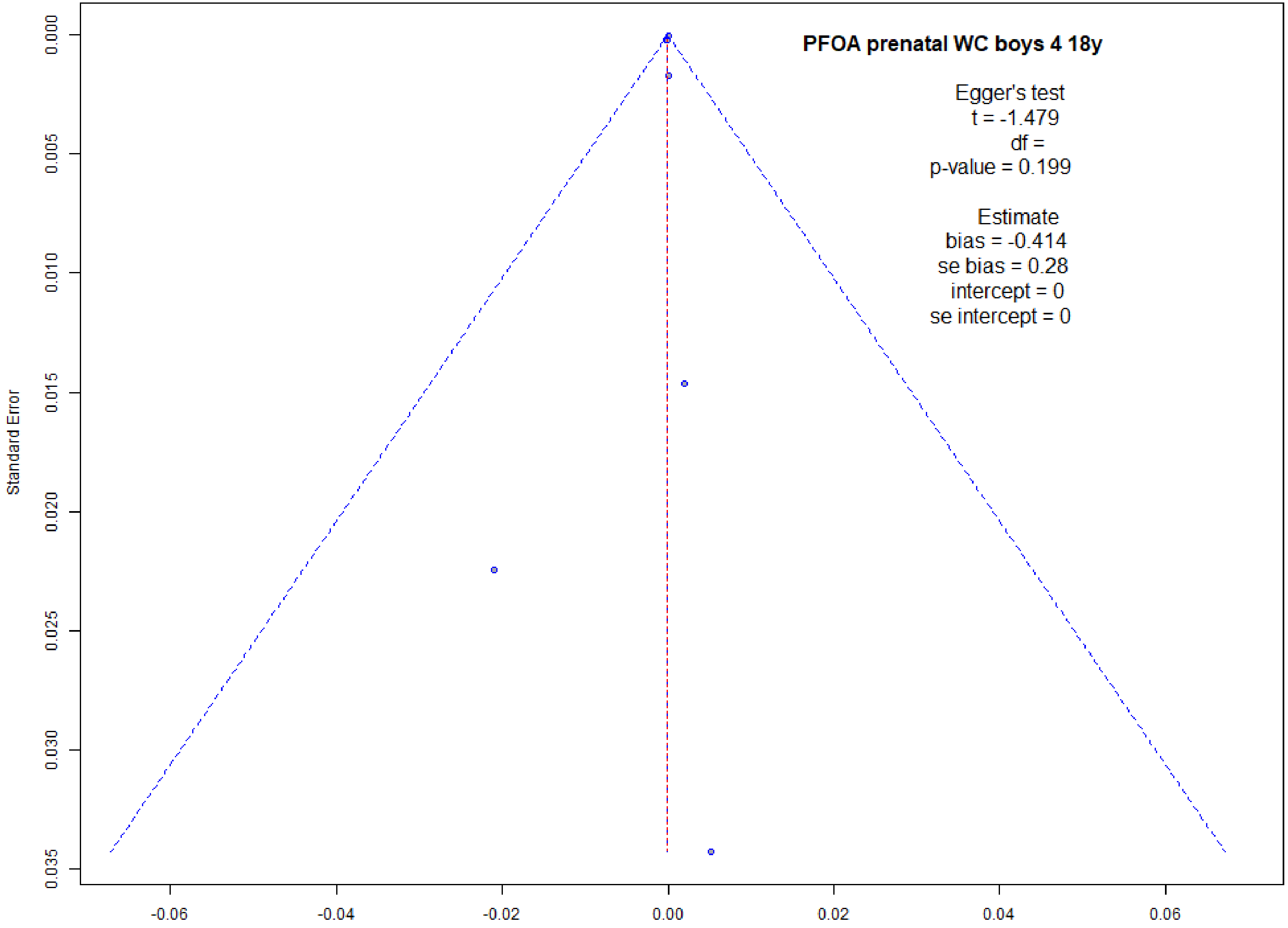
PFOA prenatal WC boys 4 18y
Egger's test
t = -1.479
df =
p-value = 0.199
Estimate
bias = -0.414
se bias = 0.28
intercept = 0
se intercept = 0
Standard Error
0.000
0.005
0.010
0.015
0.020
0.025
0.030
0.035
-0.06
-0.04
-0.02
0.00
0.02
0.04
0.06

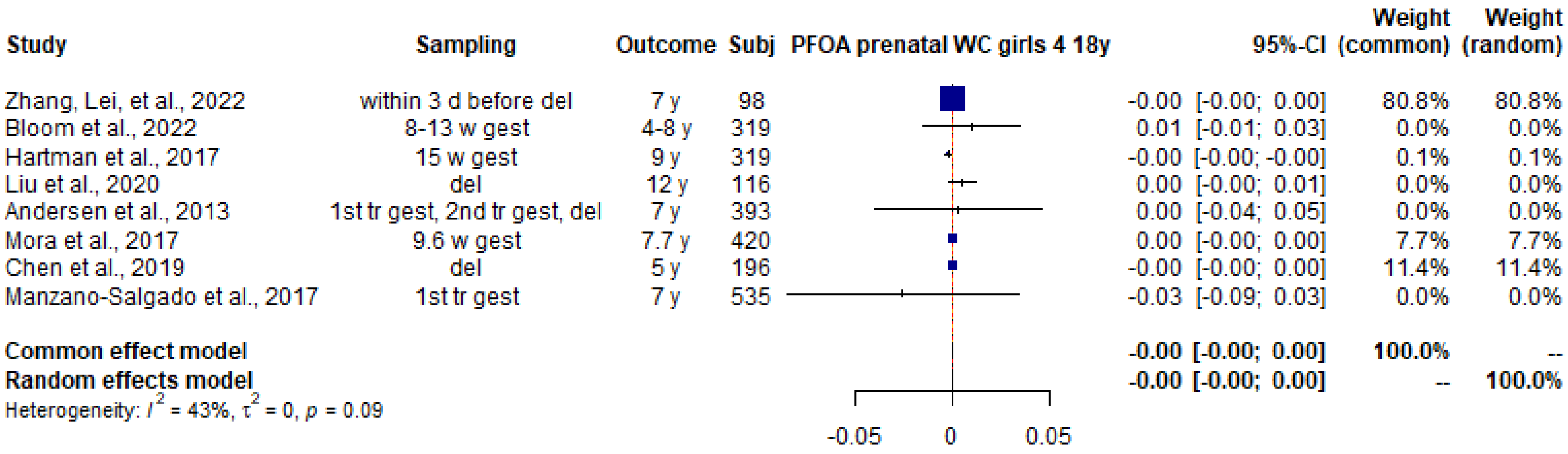
Study
Sampling
Outcome
Subj
PFOA prenatal WC girls 4 18y
95%-CI
Weight (common)
Weight (random)
Zhang, Lei, et al., 2022 | within 3 d before del | 7 y | 98 | -0.00 [-0.00; 0.00] | 80.8% | 80.8%
Bloom et al., 2022 | 8-13 w gest | 4-8 y | 319 | 0.01 [-0.01; 0.03] | 0.0% | 0.0%
Hartman et al., 2017 | 15 w gest | 9 y | 319 | -0.00 [-0.00; -0.00] | 0.1% | 0.1%
Liu et al., 2020 | del | 12 y | 116 | 0.00 [-0.00; 0.01] | 0.0% | 0.0%
Andersen et al., 2013 | 1st tr gest, 2nd tr gest, del | 7 y | 393 | 0.00 [-0.04; 0.05] | 0.0% | 0.0%
Mora et al., 2017 | 9.6 w gest | 7.7 y | 420 | 0.00 [-0.00; 0.00] | 7.7% | 7.7%
Chen et al., 2019 | del | 5 y | 196 | -0.00 [-0.00; 0.00] | 11.4% | 11.4%
Manzano-Salgado et al., 2017 | 1st tr gest | 7 y | 535 | -0.03 [-0.09; 0.03] | 0.0% | 0.0%
Common effect model | -0.00 [-0.00; 0.00] | 100.0% | --
Random effects model | -0.00 [-0.00; 0.00] | -- | 100.0%
Heterogeneity: I^2 = 43%, τ^2 = 0, p = 0.09
-0.05
0
0.05

Study	Sampling	Outcome	Subjects	Estimate [95% CI]
Zhang, Lei, et al., 2022	within 3 d before del	7 y	98	0.000 [0.000; 0.000]
Bloom et al., 2022	8-13 w gest	4-8 y	319	0.010 [-0.015; 0.035]
Hartman et al., 2017	15 w gest	9 y	319	-0.003 [-0.005; -0.001]
Liu et al., 2020	del	12 y	116	0.005 [-0.002; 0.012]
Andersen et al., 2013	1st tr gest, 2nd tr gest, del	7 y	393	0.003 [-0.040; 0.046]
Mora et al., 2017	9.6 w gest	7.7 y	420	0.000 [0.000; 0.000]
Chen et al., 2019	del	5 y	196	0.000 [0.000; 0.000]
Manzano-Salgado et al., 2017	1st tr gest	7 y	535	-0.026 [-0.085; 0.034]
**Summary**				**-0.004 [-0.019; 0.011]**

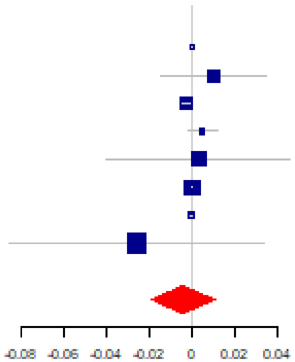

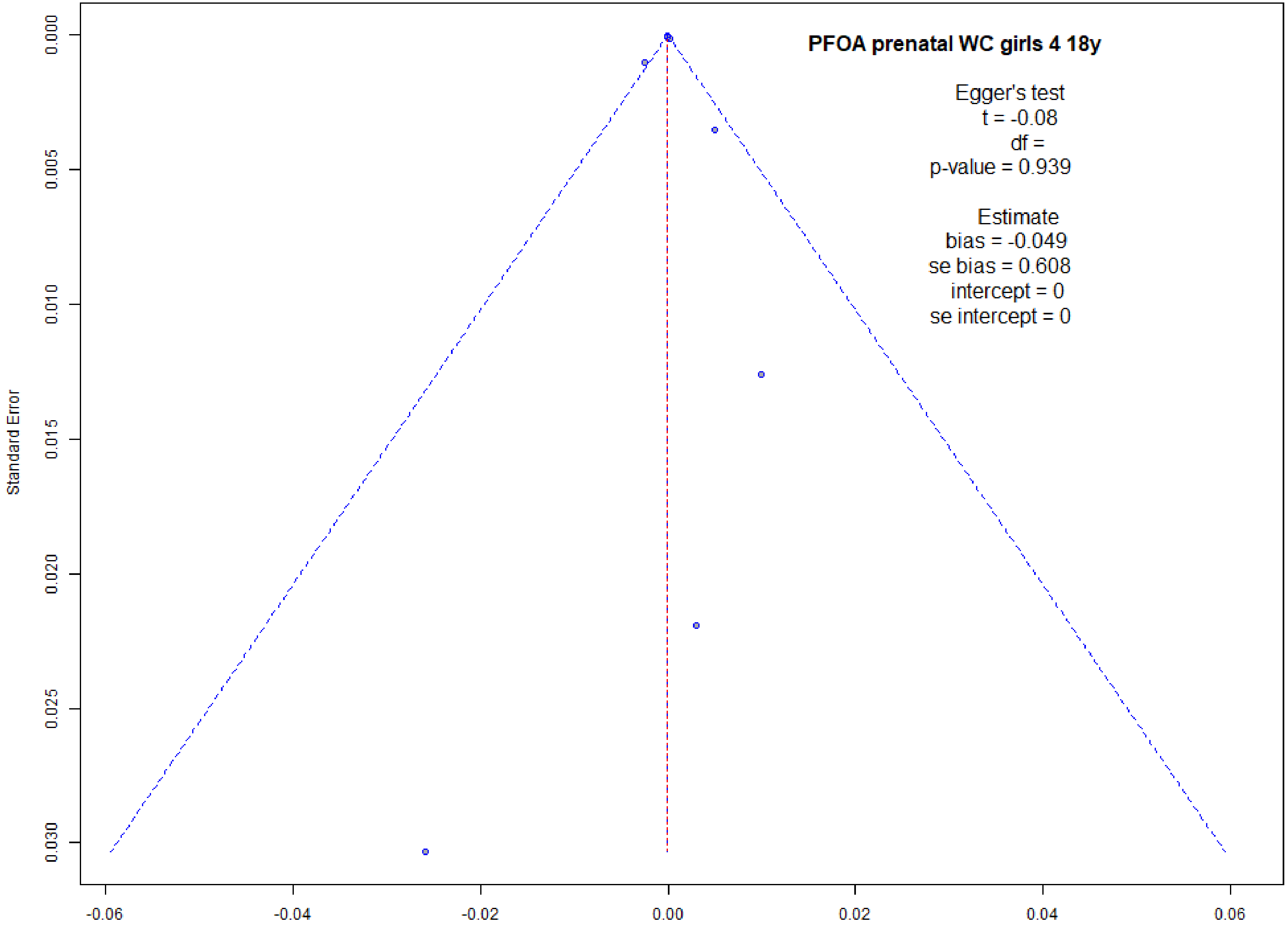
PFOA prenatal WC girls 4 18y
Egger's test
t = -0.08
df =
p-value = 0.939
Estimate
bias = -0.049
se bias = 0.608
intercept = 0
se intercept = 0
Standard Error
0.000
0.005
0.010
0.015
0.020
0.025
0.030
-0.06
-0.04
-0.02
0.00
0.02
0.04
0.06

PFOS prenatal WC both sexes 4 18y

Study	Sampling	Outcome	Subj	95%-CI		Weight (common)	Weight (random)
Papadopoulou et al., 2021	14-39 w gest	6-12 y	1101	-0.02	[-0.04; 0.01]	0.0%	0.0%
Zhang, Lei, et al., 2022	within 3 d before del	7 y	206	0.00	[-0.00; 0.00]	0.1%	0.1%
Bloom et al., 2022	8-13 w gest	4-8 y	659	-0.00	[-0.00; 0.00]	0.0%	0.0%
Liu et al., 2020	del	12 y	212	0.00	[-0.00; 0.00]	0.0%	0.0%
Mora et al., 2017	9.6 w gest	7.7 y	873	0.00	[-0.00; 0.00]	93.1%	93.1%
Chen et al., 2019	del	5 y	404	0.00	[-0.00; 0.00]	6.8%	6.8%
Manzano-Salgado et al., 2017	1st tr gest	7 y	1086	0.00	[-0.02; 0.02]	0.0%	0.0%
**Common effect model**				**0.00**	**[-0.00; 0.00]**	**100.0%**	**--**
**Random effects model**				**0.00**	**[-0.00; 0.00]**	**--**	**100.0%**

Heterogeneity: $I^2 = 0\%$, $\tau^2 = 0$, $p = 0.75$

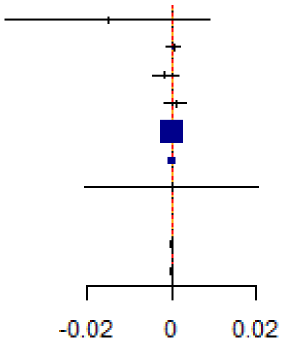

Study	Sampling	Outcome	Subjects	Estimate [95% CI]
Papadopoulou et al., 2021	14-39 w gest	6-12 y	1101	-0.015 [-0.039; 0.009]
Zhang, Lei, et al., 2022	within 3 d before del	7 y	206	0.001 [-0.001; 0.002]
Bloom et al., 2022	8-13 w gest	4-8 y	659	-0.001 [-0.004; 0.001]
Liu et al., 2020	del	12 y	212	0.001 [-0.001; 0.004]
Mora et al., 2017	9.6 w gest	7.7 y	873	0.000 [0.000; 0.000]
Chen et al., 2019	del	5 y	404	0.000 [0.000; 0.000]
Manzano-Salgado et al., 2017	1st tr gest	7 y	1086	0.000 [-0.021; 0.021]
**Summary**				**-0.004 [-0.011; 0.004]**

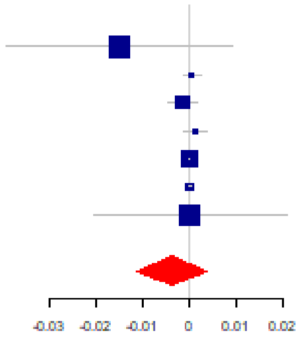

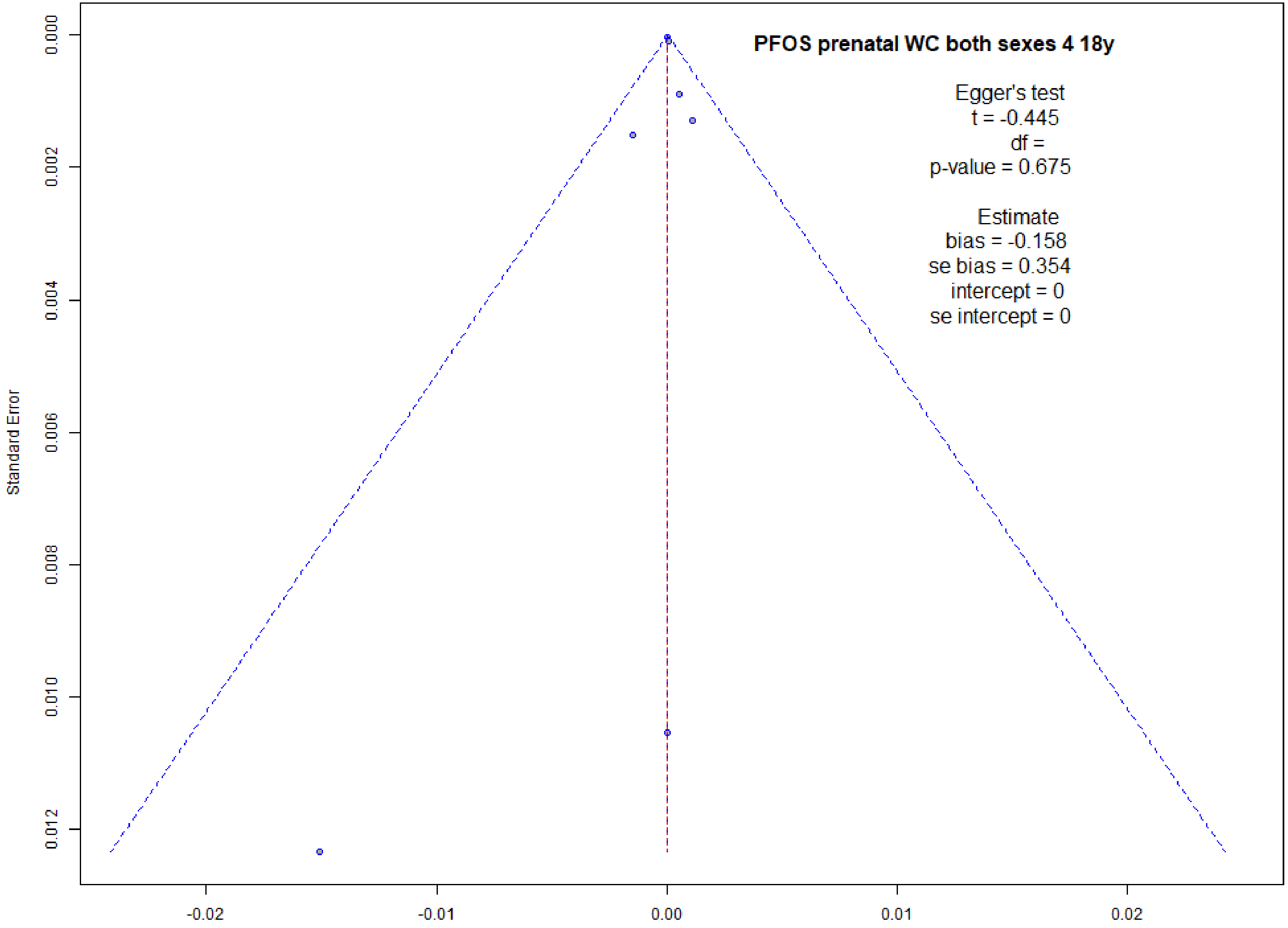
PFOS prenatal WC both sexes 4 18y
Egger's test
t = -0.445
df =
p-value = 0.675
Estimate
bias = -0.158
se bias = 0.354
intercept = 0
se intercept = 0
Standard Error
0.000
0.002
0.004
0.006
0.008
0.010
0.012
-0.02
-0.01
0.00
0.01
0.02

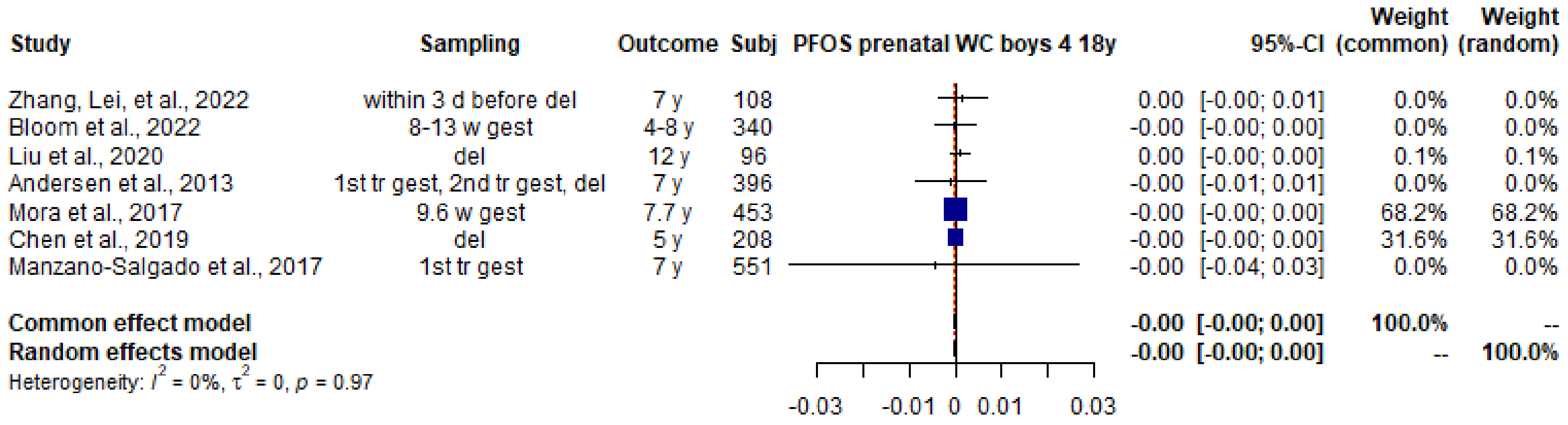
Study
Sampling
Outcome
Subj
PFOS prenatal WC boys 4 18y
95%-CI
Weight (common)
Weight (random)
Zhang, Lei, et al., 2022 within 3 d before del 7 y 108 0.00 [-0.00; 0.01] 0.0% 0.0%
Bloom et al., 2022 8-13 w gest 4-8 y 340 -0.00 [-0.00; 0.00] 0.0% 0.0%
Liu et al., 2020 del 12 y 96 0.00 [-0.00; 0.00] 0.1% 0.1%
Andersen et al., 2013 1st tr gest, 2nd tr gest, del 7 y 396 -0.00 [-0.01; 0.01] 0.0% 0.0%
Mora et al., 2017 9.6 w gest 7.7 y 453 -0.00 [-0.00; 0.00] 68.2% 68.2%
Chen et al., 2019 del 5 y 208 -0.00 [-0.00; 0.00] 31.6% 31.6%
Manzano-Salgado et al., 2017 1st tr gest 7 y 551 -0.00 [-0.04; 0.03] 0.0% 0.0%
Common effect model -0.00 [-0.00; 0.00] 100.0% --
Random effects model -0.00 [-0.00; 0.00] -- 100.0%
Heterogeneity: I^2 = 0%, τ^2 = 0, p = 0.97
-0.03
-0.01
0
0.01
0.03

Study	Sampling	Outcome	Subjects	Estimate [95% CI]
Zhang, Lei, et al., 2022	within 3 d before del	7 y	108	0.002 [-0.004; 0.007]
Bloom et al., 2022	8-13 w gest	4-8 y	340	0.000 [-0.004; 0.004]
Liu et al., 2020	del	12 y	96	0.001 [-0.001; 0.003]
Andersen et al., 2013	1st tr gest, 2nd tr gest, del	7 y	396	-0.001 [-0.009; 0.006]
Mora et al., 2017	9.6 w gest	7.7 y	453	0.000 [0.000; 0.000]
Chen et al., 2019	del	5 y	208	0.000 [0.000; 0.000]
Manzano-Salgado et al., 2017	1st tr gest	7 y	551	-0.005 [-0.036; 0.026]
**Summary**				**-0.001 [-0.009; 0.007]**

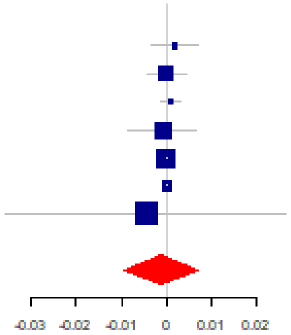

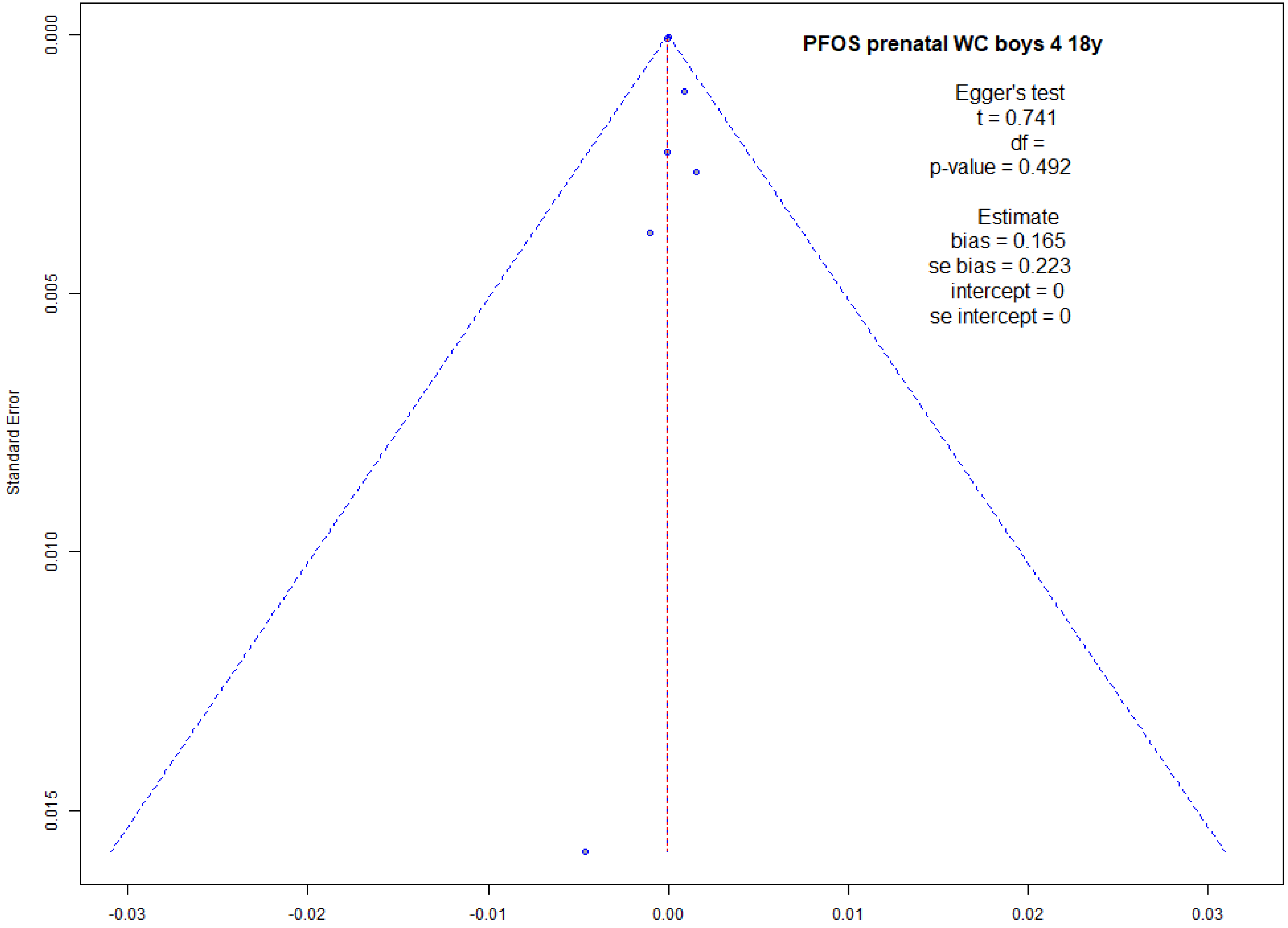
PFOS prenatal WC boys 4 18y
Egger's test
t = 0.741
df =
p-value = 0.492
Estimate
bias = 0.165
se bias = 0.223
intercept = 0
se intercept = 0
Standard Error
0.000
0.005
0.010
0.015
-0.03
-0.02
-0.01
0.00
0.01
0.02
0.03

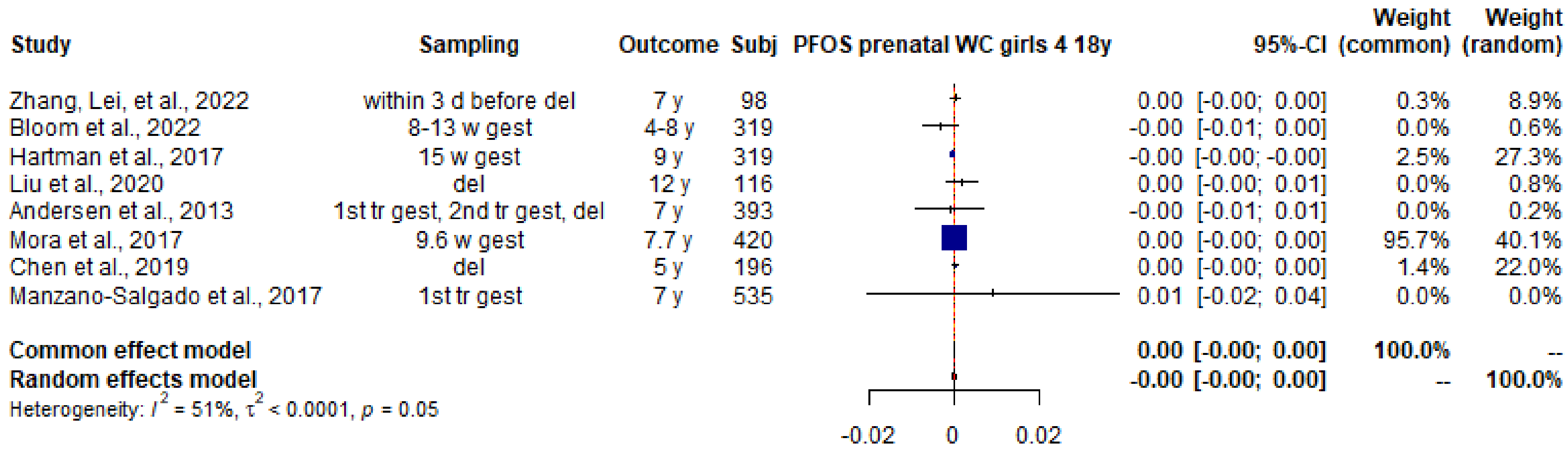
Study | Sampling | Outcome | Subj | PFOS prenatal WC girls 4 18y | 95%-CI | Weight (common) | Weight (random)
Zhang, Lei, et al., 2022 | within 3 d before del | 7 y | 98 | 0.00 [-0.00; 0.00] | 0.3% | 8.9%
Bloom et al., 2022 | 8-13 w gest | 4-8 y | 319 | -0.00 [-0.01; 0.00] | 0.0% | 0.6%
Hartman et al., 2017 | 15 w gest | 9 y | 319 | -0.00 [-0.00; -0.00] | 2.5% | 27.3%
Liu et al., 2020 | del | 12 y | 116 | 0.00 [-0.00; 0.01] | 0.0% | 0.8%
Andersen et al., 2013 | 1st tr gest, 2nd tr gest, del | 7 y | 393 | -0.00 [-0.01; 0.01] | 0.0% | 0.2%
Mora et al., 2017 | 9.6 w gest | 7.7 y | 420 | 0.00 [-0.00; 0.00] | 95.7% | 40.1%
Chen et al., 2019 | del | 5 y | 196 | 0.00 [-0.00; 0.00] | 1.4% | 22.0%
Manzano-Salgado et al., 2017 | 1st tr gest | 7 y | 535 | 0.01 [-0.02; 0.04] | 0.0% | 0.0%
Common effect model | 0.00 [-0.00; 0.00] | 100.0% | --
Random effects model | -0.00 [-0.00; 0.00] | -- | 100.0%
Heterogeneity: I^2 = 51%, τ^2 < 0.0001, p = 0.05
-0.02
0
0.02

Study	Sampling	Outcome	Subjects	Estimate [95% CI]
Zhang, Lei, et al., 2022	within 3 d before del	7 y	98	0.000 [-0.001; 0.001]
Bloom et al., 2022	8-13 w gest	4-8 y	319	-0.003 [-0.008; 0.001]
Hartman et al., 2017	15 w gest	9 y	319	-0.001 [-0.001; 0.000]
Liu et al., 2020	del	12 y	116	0.002 [-0.002; 0.005]
Andersen et al., 2013	1st tr gest, 2nd tr gest, del	7 y	393	-0.001 [-0.009; 0.007]
Mora et al., 2017	9.6 w gest	7.7 y	420	0.000 [0.000; 0.000]
Chen et al., 2019	del	5 y	196	0.000 [0.000; 0.001]
Manzano-Salgado et al., 2017	1st tr gest	7 y	535	0.009 [-0.021; 0.039]
**Summary**				**0.001 [-0.005; 0.008]**

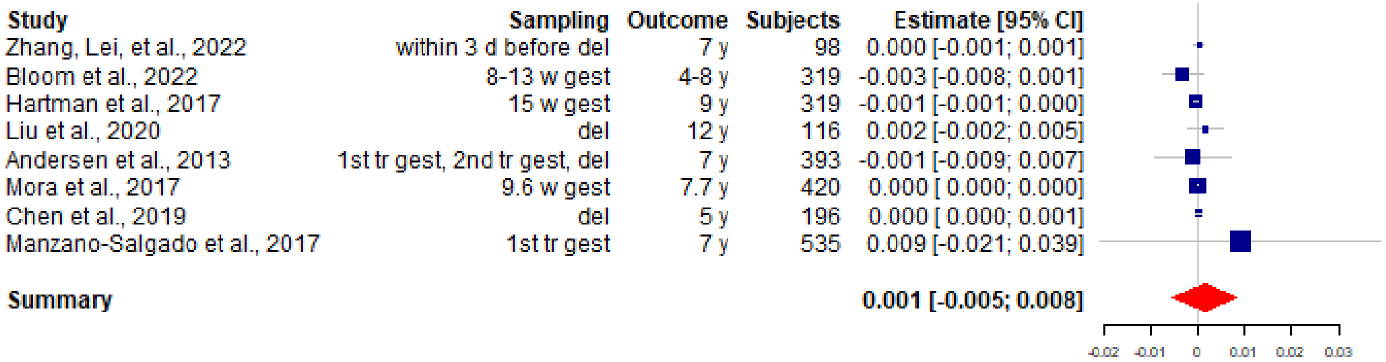

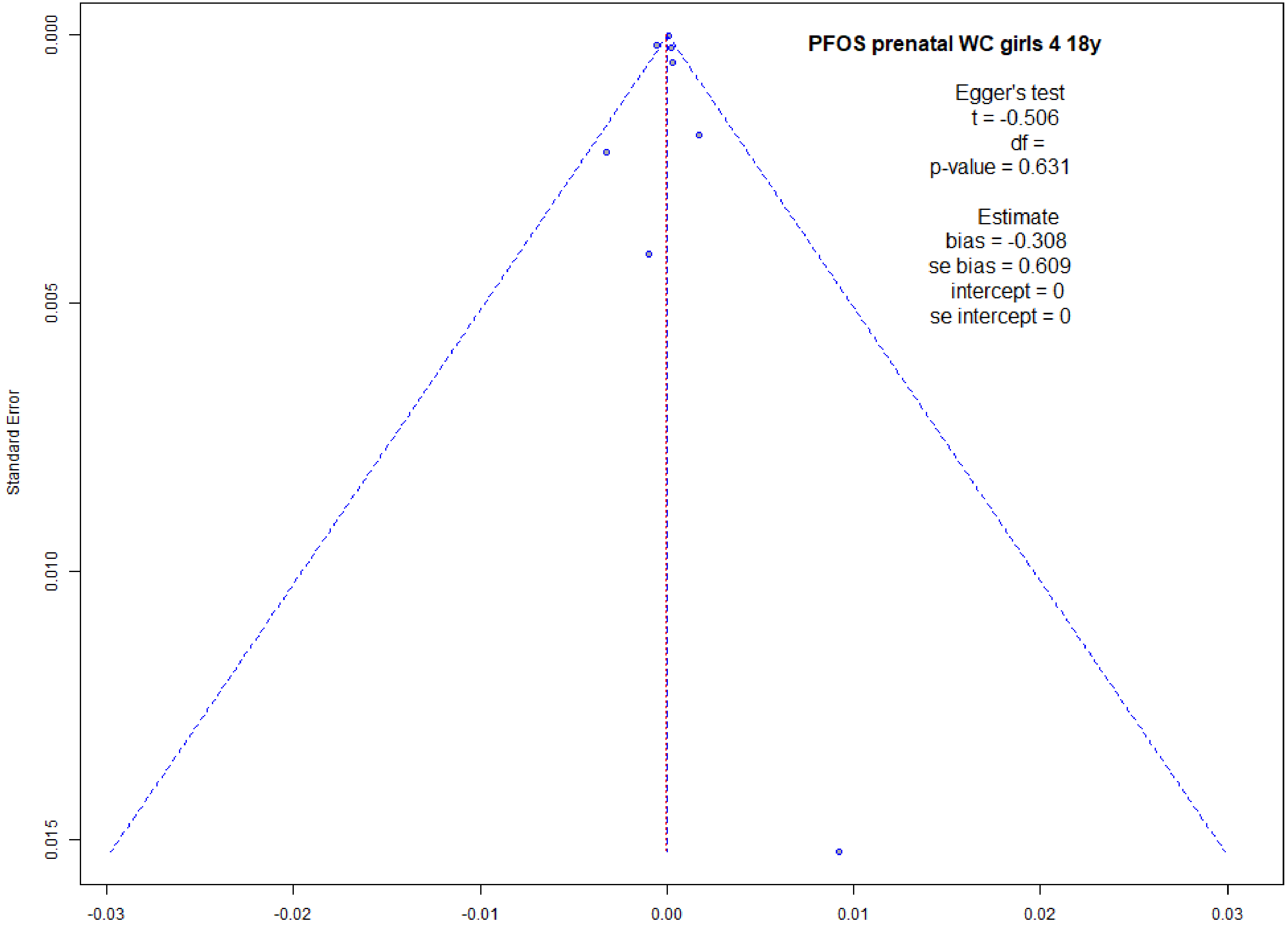
PFOS prenatal WC girls 4 18y
Egger's test
t = -0.506
df =
p-value = 0.631
Estimate
bias = -0.308
se bias = 0.609
intercept = 0
se intercept = 0
Standard Error
0.000
0.005
0.010
0.015
-0.03
-0.02
-0.01
0.00
0.01
0.02
0.03

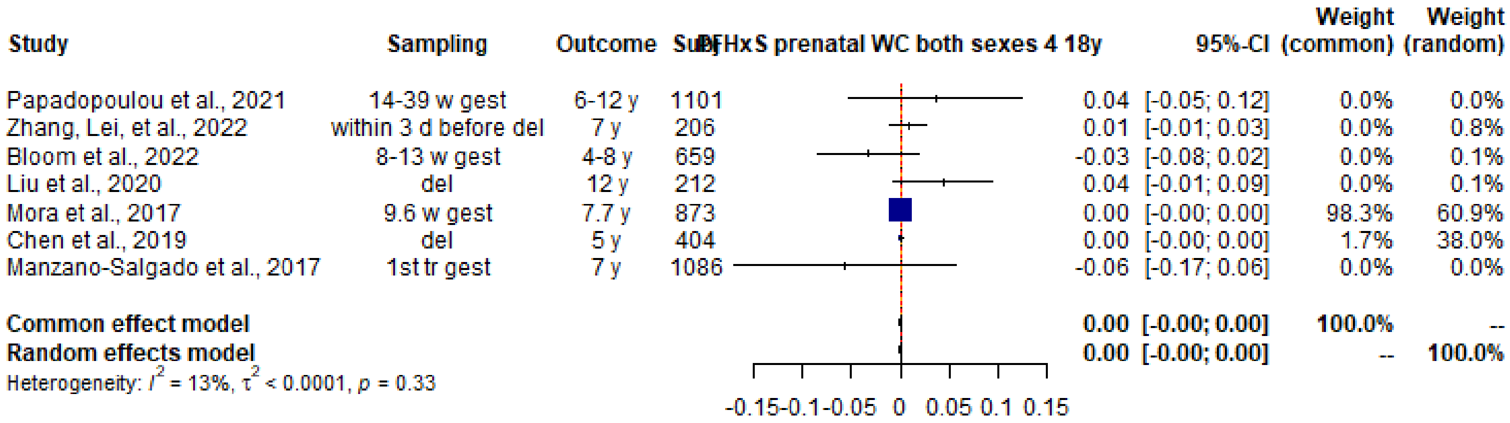
Study
Sampling
Outcome
PFHxS prenatal WC both sexes 4 18y
95%-CI
Weight (common)
Weight (random)
Papadopoulou et al., 2021 | 14-39 w gest | 6-12 y | 1101 | 0.04 | [-0.05; 0.12] | 0.0% | 0.0%
Zhang, Lei, et al., 2022 | within 3 d before del | 7 y | 206 | 0.01 | [-0.01; 0.03] | 0.0% | 0.8%
Bloom et al., 2022 | 8-13 w gest | 4-8 y | 659 | -0.03 | [-0.08; 0.02] | 0.0% | 0.1%
Liu et al., 2020 | del | 12 y | 212 | 0.04 | [-0.01; 0.09] | 0.0% | 0.1%
Mora et al., 2017 | 9.6 w gest | 7.7 y | 873 | 0.00 | [-0.00; 0.00] | 98.3% | 60.9%
Chen et al., 2019 | del | 5 y | 404 | 0.00 | [-0.00; 0.00] | 1.7% | 38.0%
Manzano-Salgado et al., 2017 | 1st tr gest | 7 y | 1086 | -0.06 | [-0.17; 0.06] | 0.0% | 0.0%
Common effect model | 0.00 | [-0.00; 0.00] | 100.0% | --
Random effects model | 0.00 | [-0.00; 0.00] | -- | 100.0%
Heterogeneity: I^2 = 13%, τ^2 < 0.0001, p = 0.33
-0.15 -0.1 -0.05 0 0.05 0.1 0.15

Study	Sampling	Outcome	Subjects	Estimate [95% CI]
Papadopoulou et al., 2021	14-39 w gest	6-12 y	1101	0.036 [-0.052; 0.123]
Zhang, Lei, et al., 2022	within 3 d before del	7 y	206	0.009 [-0.010; 0.028]
Bloom et al., 2022	8-13 w gest	4-8 y	659	-0.032 [-0.084; 0.019]
Liu et al., 2020	del	12 y	212	0.044 [-0.007; 0.094]
Mora et al., 2017	9.6 w gest	7.7 y	873	0.000 [0.000; 0.000]
Chen et al., 2019	del	5 y	404	0.000 [-0.001; 0.002]
Manzano-Salgado et al., 2017	1st tr gest	7 y	1086	-0.056 [-0.168; 0.056]
**Summary**				**-0.007 [-0.042; 0.028]**

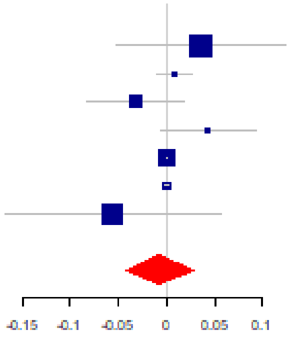

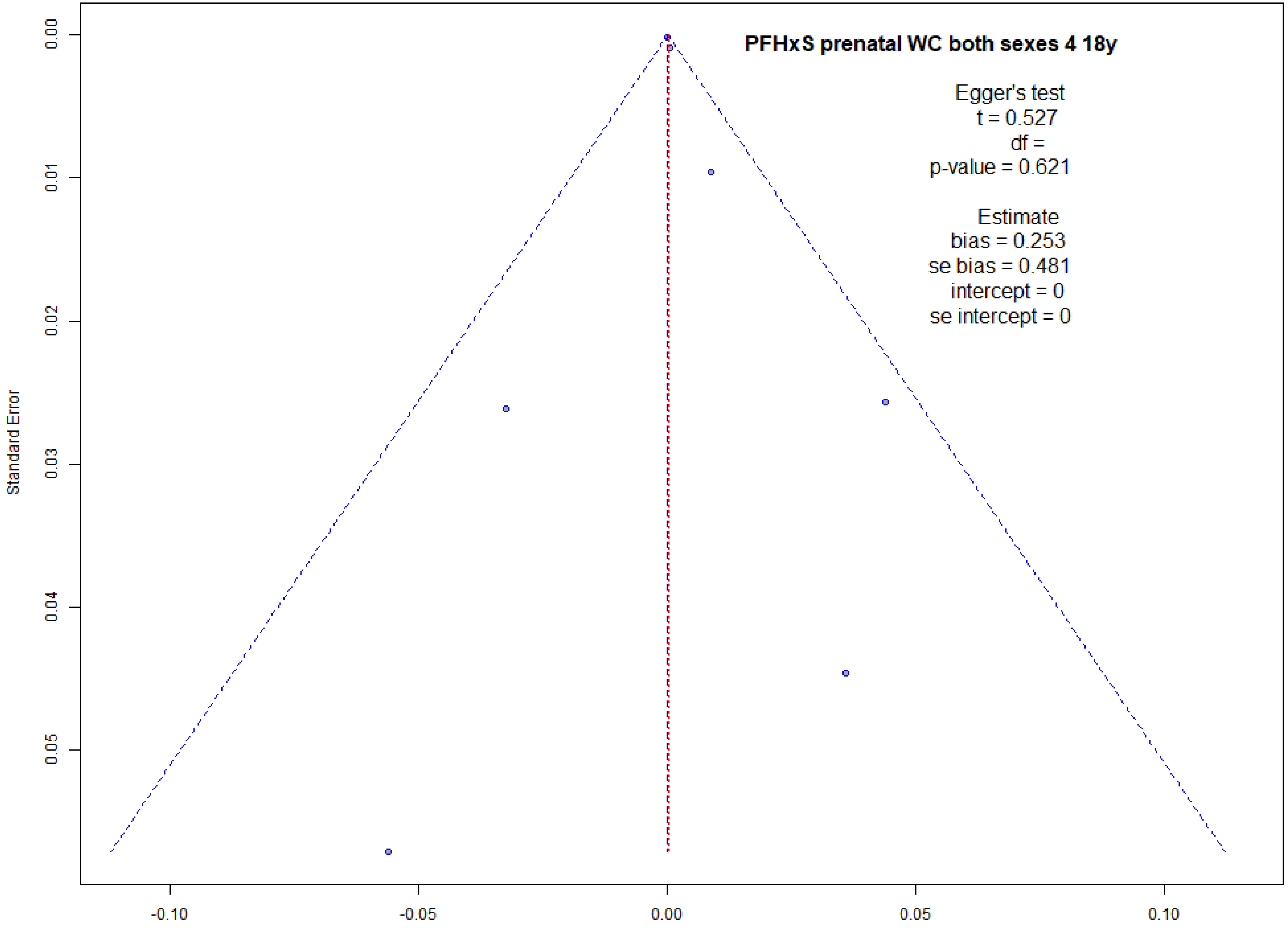
PFHxS prenatal WC both sexes 4 18y
Egger's test
t = 0.527
df =
p-value = 0.621
Estimate
bias = 0.253
se bias = 0.481
intercept = 0
se intercept = 0
Standard Error
0.00
0.01
0.02
0.03
0.04
0.05
-0.10
-0.05
0.00
0.05
0.10

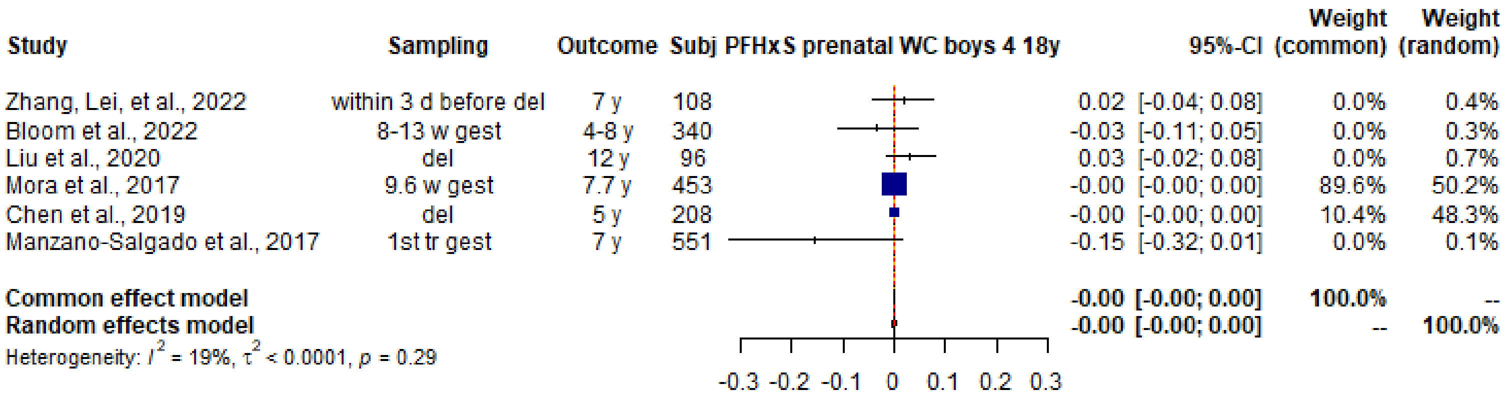
Study
Sampling
Outcome
Subj
PFHxS prenatal WC boys 4 18y
95%-CI
Weight (common)
Weight (random)
Zhang, Lei, et al., 2022 within 3 d before del 7 y 108 0.02 [-0.04; 0.08] 0.0% 0.4%
Bloom et al., 2022 8-13 w gest 4-8 y 340 -0.03 [-0.11; 0.05] 0.0% 0.3%
Liu et al., 2020 del 12 y 96 0.03 [-0.02; 0.08] 0.0% 0.7%
Mora et al., 2017 9.6 w gest 7.7 y 453 -0.00 [-0.00; 0.00] 89.6% 50.2%
Chen et al., 2019 del 5 y 208 -0.00 [-0.00; 0.00] 10.4% 48.3%
Manzano-Salgado et al., 2017 1st tr gest 7 y 551 -0.15 [-0.32; 0.01] 0.0% 0.1%
Common effect model -0.00 [-0.00; 0.00] 100.0% --
Random effects model -0.00 [-0.00; 0.00] -- 100.0%
Heterogeneity: I^2 = 19%, τ^2 < 0.0001, p = 0.29
-0.3 -0.2 -0.1 0 0.1 0.2 0.3

Study	Sampling	Outcome	Subjects	Estimate [95% CI]
Zhang, Lei, et al., 2022	within 3 d before del	7 y	108	0.018 [-0.041; 0.077]
Bloom et al., 2022	8-13 w gest	4-8 y	340	-0.032 [-0.110; 0.046]
Liu et al., 2020	del	12 y	96	0.031 [-0.015; 0.078]
Mora et al., 2017	9.6 w gest	7.7 y	453	0.000 [-0.001; 0.000]
Chen et al., 2019	del	5 y	208	0.000 [-0.002; 0.001]
Manzano-Salgado et al., 2017	1st tr gest	7 y	551	-0.154 [-0.322; 0.014]
**Summary**				**-0.052 [-0.107; 0.003]**

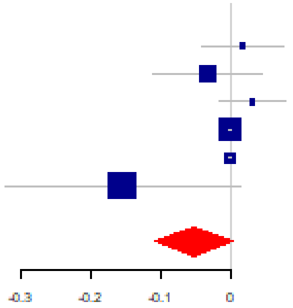

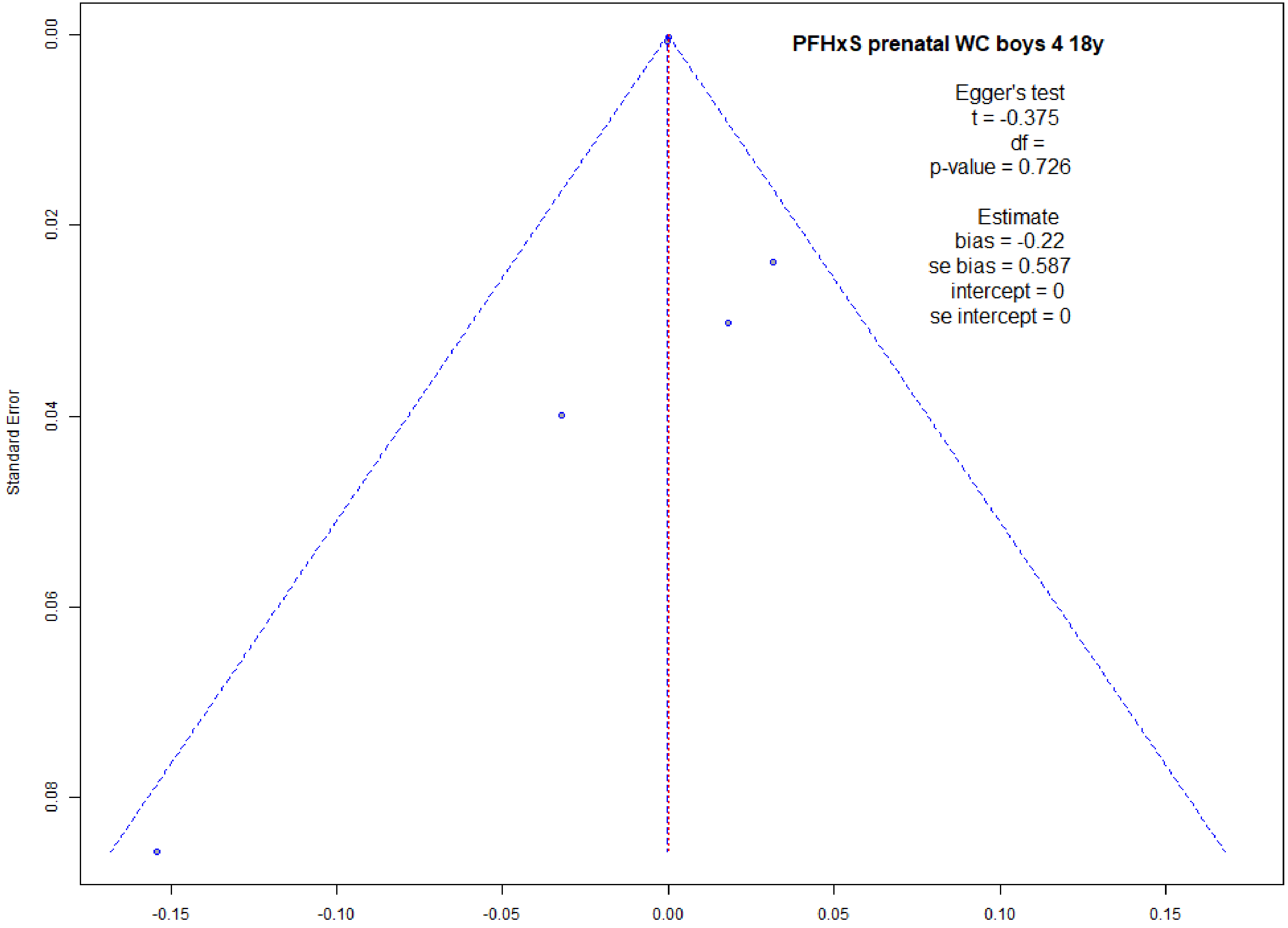
PFHxS prenatal WC boys 4 18y
Egger's test
t = -0.375
df =
p-value = 0.726
Estimate
bias = -0.22
se bias = 0.587
intercept = 0
se intercept = 0
Standard Error
0.00
0.02
0.04
0.06
0.08
-0.15
-0.10
-0.05
0.00
0.05
0.10
0.15

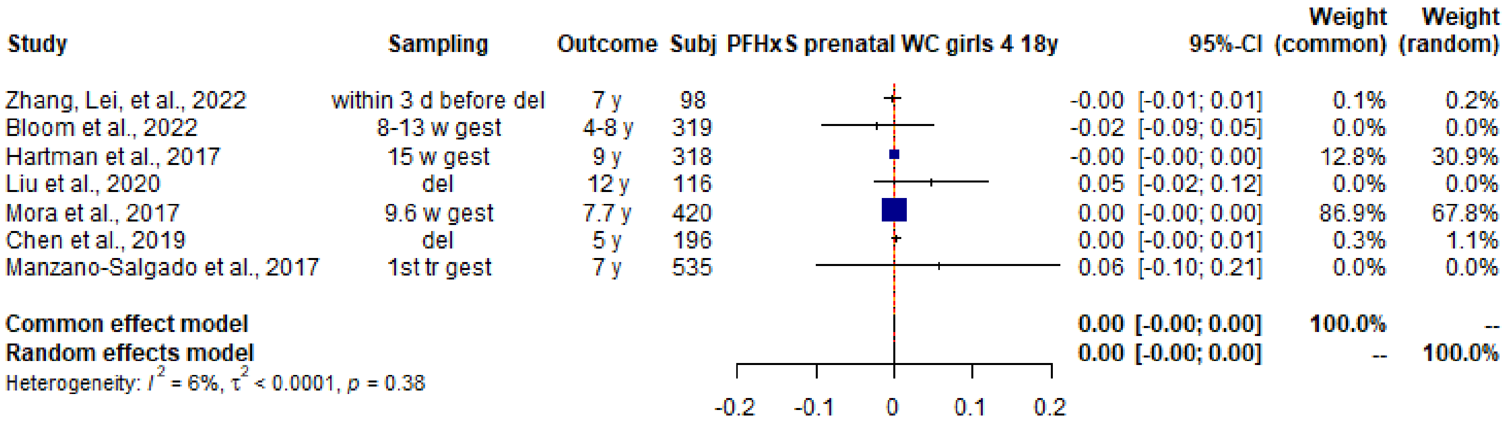
Study
Sampling
Outcome
Subj
PFHxS prenatal WC girls 4 18y
95%-CI
Weight (common)
Weight (random)
Zhang, Lei, et al., 2022 | within 3 d before del | 7 y | 98 | -0.00 [-0.01; 0.01] | 0.1% | 0.2%
Bloom et al., 2022 | 8-13 w gest | 4-8 y | 319 | -0.02 [-0.09; 0.05] | 0.0% | 0.0%
Hartman et al., 2017 | 15 w gest | 9 y | 318 | -0.00 [-0.00; 0.00] | 12.8% | 30.9%
Liu et al., 2020 | del | 12 y | 116 | 0.05 [-0.02; 0.12] | 0.0% | 0.0%
Mora et al., 2017 | 9.6 w gest | 7.7 y | 420 | 0.00 [-0.00; 0.00] | 86.9% | 67.8%
Chen et al., 2019 | del | 5 y | 196 | 0.00 [-0.00; 0.01] | 0.3% | 1.1%
Manzano-Salgado et al., 2017 | 1st tr gest | 7 y | 535 | 0.06 [-0.10; 0.21] | 0.0% | 0.0%
Common effect model | 0.00 [-0.00; 0.00] | 100.0% | --
Random effects model | 0.00 [-0.00; 0.00] | -- | 100.0%
Heterogeneity: I^2 = 6%, τ^2 < 0.0001, p = 0.38
-0.2
-0.1
0
0.1
0.2

Study	Sampling	Outcome	Subjects	Estimate [95% CI]
Zhang, Lei, et al., 2022	within 3 d before del	7 y	98	-0.003 [-0.012; 0.007]
Bloom et al., 2022	8-13 w gest	4-8 y	319	-0.022 [-0.092; 0.049]
Hartman et al., 2017	15 w gest	9 y	318	0.000 [-0.001; 0.000]
Liu et al., 2020	del	12 y	116	0.047 [-0.024; 0.118]
Mora et al., 2017	9.6 w gest	7.7 y	420	0.000 [0.000; 0.000]
Chen et al., 2019	del	5 y	196	0.003 [-0.002; 0.007]
Manzano-Salgado et al., 2017	1st tr gest	7 y	535	0.056 [-0.098; 0.210]
**Summary**				**0.014 [-0.028; 0.057]**

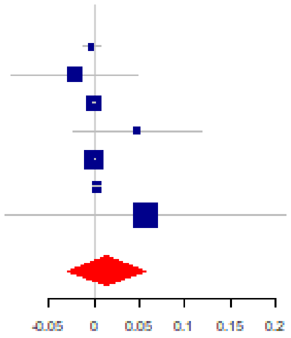

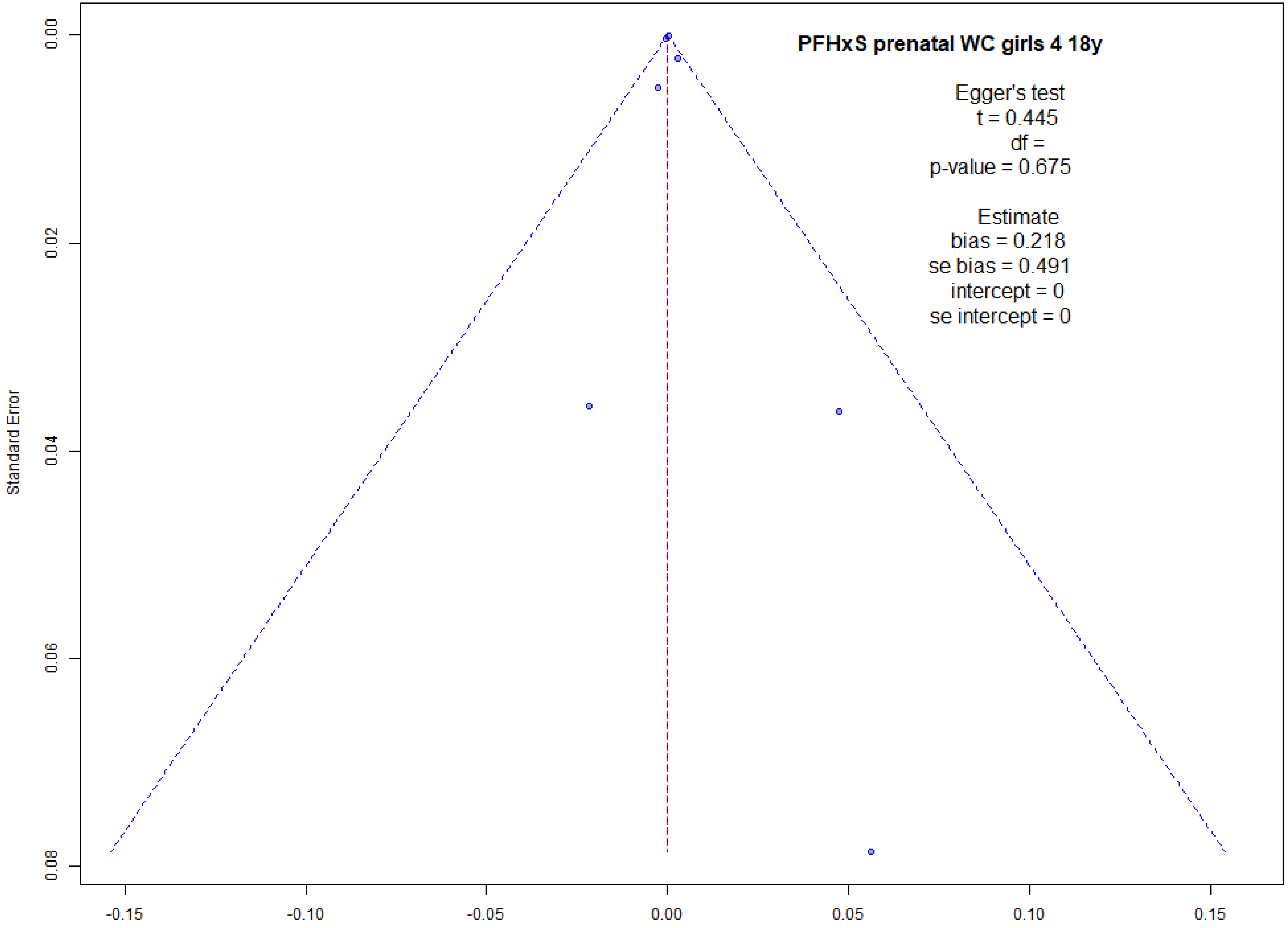
PFHxS prenatal WC girls 4 18y
Egger's test
t = 0.445
df =
p-value = 0.675
Estimate
bias = 0.218
se bias = 0.491
intercept = 0
se intercept = 0
Standard Error
0.00
0.02
0.04
0.06
0.08
-0.15
-0.10
-0.05
0.00
0.05
0.10
0.15

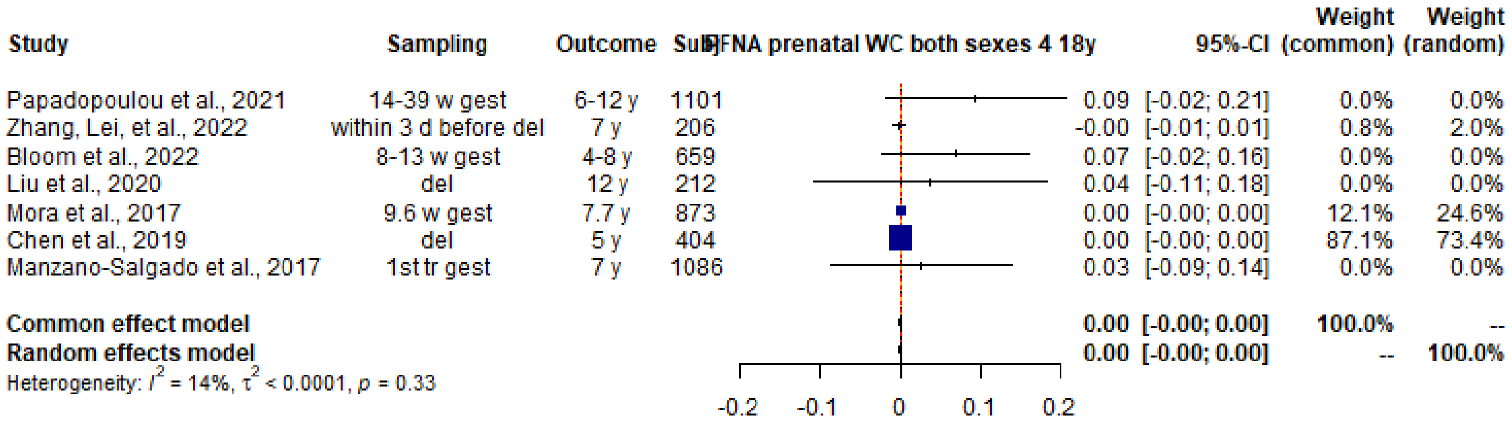
Study
Sampling
Outcome
Sub
PFNA prenatal WC both sexes 4 18y
95%-CI
Weight (common)
Weight (random)
Papadopoulou et al., 2021 14-39 w gest 6-12 y 1101 0.09 [-0.02; 0.21] 0.0% 0.0%
Zhang, Lei, et al., 2022 within 3 d before del 7 y 206 -0.00 [-0.01; 0.01] 0.8% 2.0%
Bloom et al., 2022 8-13 w gest 4-8 y 659 0.07 [-0.02; 0.16] 0.0% 0.0%
Liu et al., 2020 del 12 y 212 0.04 [-0.11; 0.18] 0.0% 0.0%
Mora et al., 2017 9.6 w gest 7.7 y 873 0.00 [-0.00; 0.00] 12.1% 24.6%
Chen et al., 2019 del 5 y 404 0.00 [-0.00; 0.00] 87.1% 73.4%
Manzano-Salgado et al., 2017 1st tr gest 7 y 1086 0.03 [-0.09; 0.14] 0.0% 0.0%
Common effect model 0.00 [-0.00; 0.00] 100.0% --
Random effects model 0.00 [-0.00; 0.00] -- 100.0%
Heterogeneity: I^2 = 14%, τ^2 < 0.0001, p = 0.33
-0.2
-0.1
0
0.1
0.2

Study	Sampling	Outcome	Subjects	Estimate [95% CI]
Papadopoulou et al., 2021	14-39 w gest	6-12 y	1101	0.094 [-0.019; 0.207]
Zhang, Lei, et al., 2022	within 3 d before del	7 y	206	-0.001 [-0.008; 0.006]
Bloom et al., 2022	8-13 w gest	4-8 y	659	0.069 [-0.023; 0.162]
Liu et al., 2020	del	12 y	212	0.037 [-0.109; 0.183]
Mora et al., 2017	9.6 w gest	7.7 y	873	0.001 [0.000; 0.003]
Chen et al., 2019	del	5 y	404	0.000 [-0.001; 0.001]
Manzano-Salgado et al., 2017	1st tr gest	7 y	1086	0.027 [-0.086; 0.140]
**Summary**				**0.041 [0.000; 0.082]**

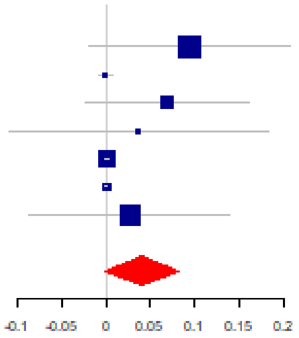

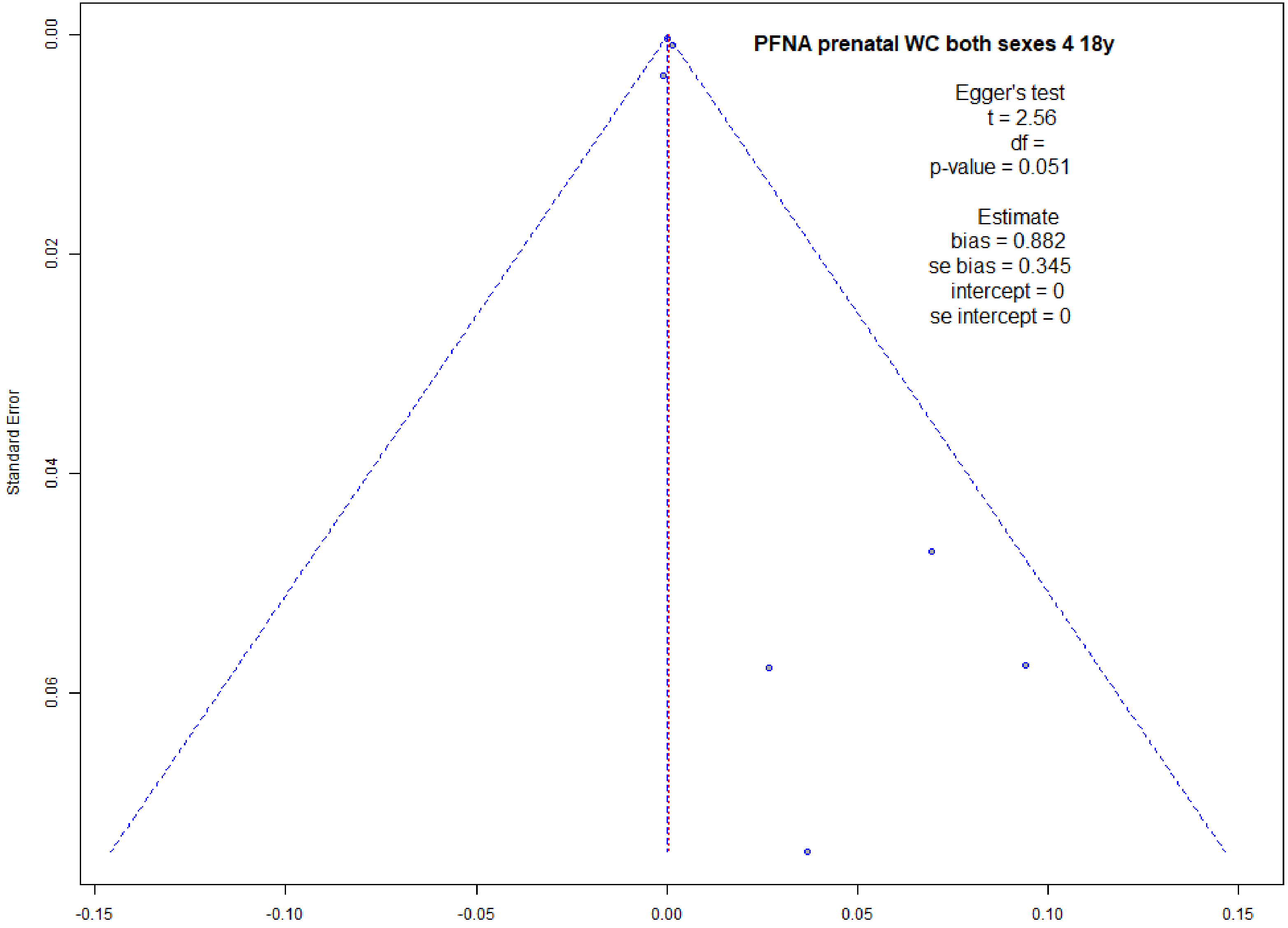
PFNA prenatal WC both sexes 4 18y
Egger's test
t = 2.56
df =
p-value = 0.051
Estimate
bias = 0.882
se bias = 0.345
intercept = 0
se intercept = 0
Standard Error
0.00
0.02
0.04
0.06
-0.15
-0.10
-0.05
0.00
0.05
0.10
0.15

Study	Sampling	Outcome	Subj	PFNA prenatal WC boys 4 18y	95%-CI	Weight (common)	Weight (random)
Zhang, Lei, et al., 2022	within 3 d before del	7 y	108		-0.00 [-0.03; 0.02]	0.0%	0.0%
Bloom et al., 2022	8-13 w gest	4-8 y	340		0.08 [-0.07; 0.23]	0.0%	0.0%
Liu et al., 2020	del	12 y	96		0.05 [-0.05; 0.16]	0.0%	0.0%
Mora et al., 2017	9.6 w gest	7.7 y	453		0.00 [-0.00; 0.00]	2.2%	2.2%
Chen et al., 2019	del	5 y	208		-0.00 [-0.00; 0.00]	97.7%	97.7%
Manzano-Salgado et al., 2017	1st tr gest	7 y	551		0.09 [-0.07; 0.25]	0.0%	0.0%
**Common effect model**					**-0.00 [-0.00; 0.00]**	**100.0%**	--
**Random effects model**					**-0.00 [-0.00; 0.00]**	--	**100.0%**

Heterogeneity: $I^2 = 0\%$, $\tau^2 = 0$, $p = 0.59$

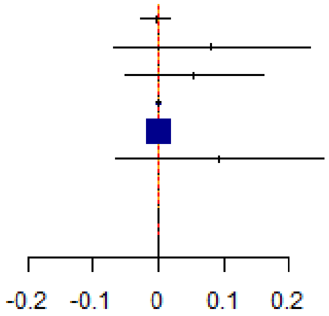

Study	Sampling	Outcome	Subjects	Estimate [95% CI]
Zhang, Lei, et al., 2022	within 3 d before del	7 y	108	-0.004 [-0.027; 0.019]
Bloom et al., 2022	8-13 w gest	4-8 y	340	0.081 [-0.069; 0.231]
Liu et al., 2020	del	12 y	96	0.054 [-0.052; 0.159]
Mora et al., 2017	9.6 w gest	7.7 y	453	0.001 [-0.003; 0.004]
Chen et al., 2019	del	5 y	208	0.000 [-0.001; 0.000]
Manzano-Salgado et al., 2017	1st tr gest	7 y	551	0.093 [-0.067; 0.253]
**Summary**				**0.048 [-0.011; 0.106]**

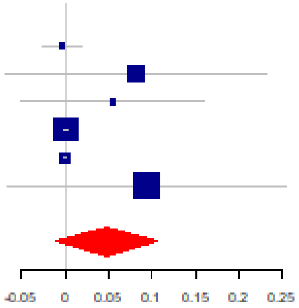

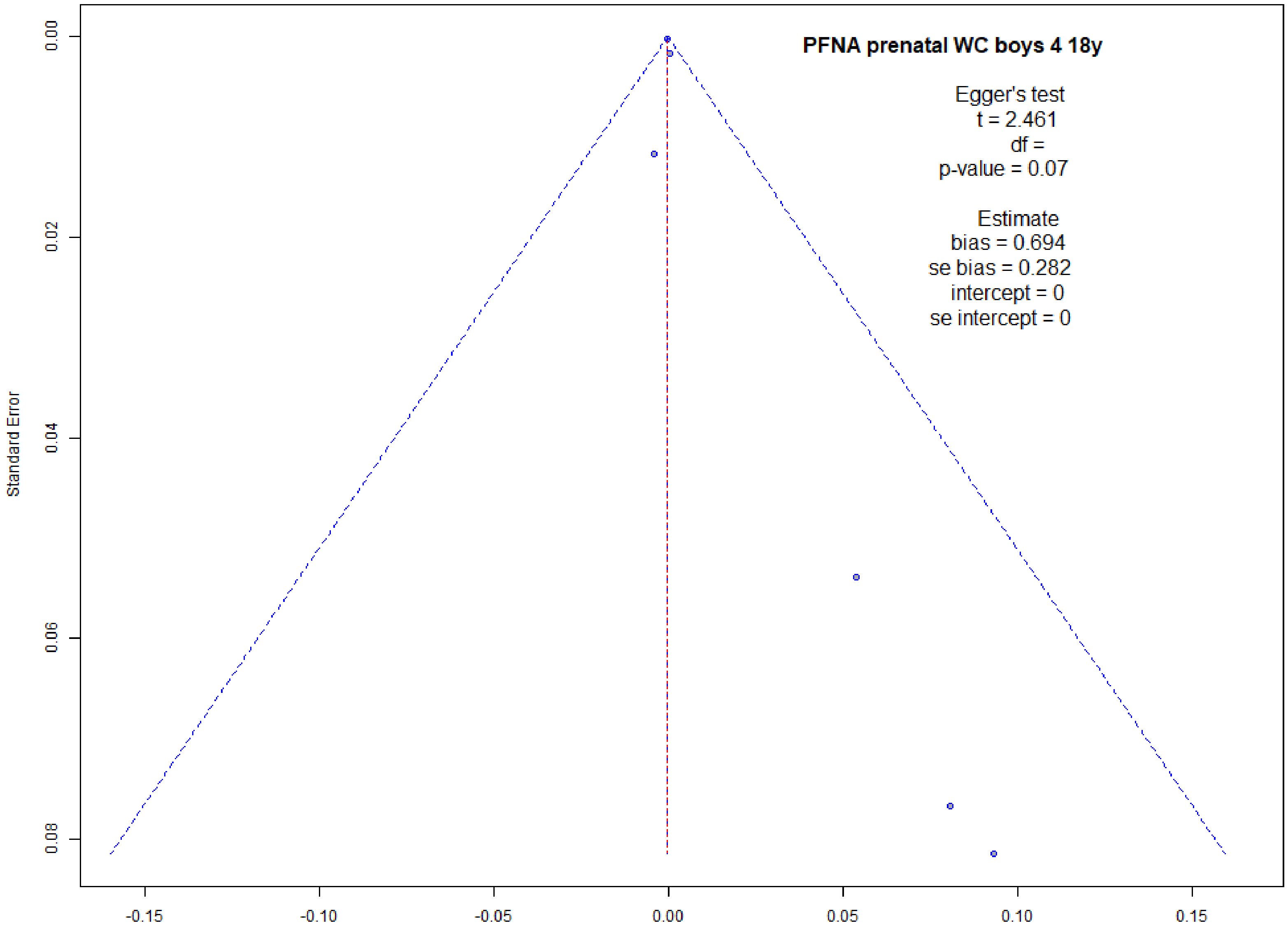
PFNA prenatal WC boys 4 18y
Egger's test
t = 2.461
df =
p-value = 0.07
Estimate
bias = 0.694
se bias = 0.282
intercept = 0
se intercept = 0
Standard Error
0.00
0.02
0.04
0.06
0.08
-0.15
-0.10
-0.05
0.00
0.05
0.10
0.15

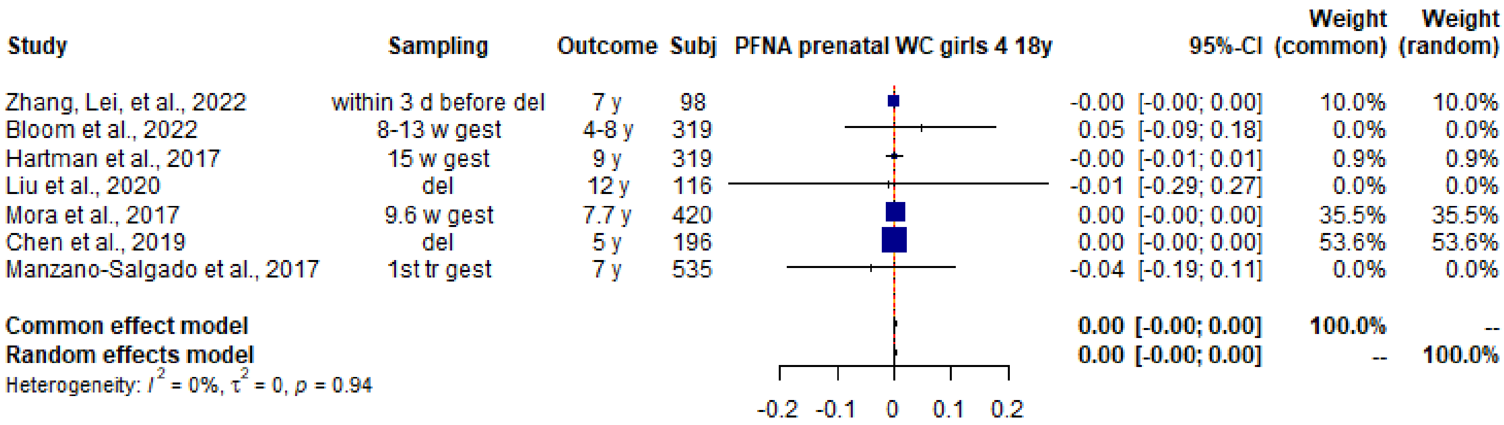
Study
Sampling
Outcome
Subj
PFNA prenatal WC girls 4 18y
95%-CI
Weight (common)
Weight (random)
Zhang, Lei, et al., 2022 within 3 d before del 7 y 98 -0.00 [-0.00; 0.00] 10.0% 10.0%
Bloom et al., 2022 8-13 w gest 4-8 y 319 0.05 [-0.09; 0.18] 0.0% 0.0%
Hartman et al., 2017 15 w gest 9 y 319 -0.00 [-0.01; 0.01] 0.9% 0.9%
Liu et al., 2020 del 12 y 116 -0.01 [-0.29; 0.27] 0.0% 0.0%
Mora et al., 2017 9.6 w gest 7.7 y 420 0.00 [-0.00; 0.00] 35.5% 35.5%
Chen et al., 2019 del 5 y 196 0.00 [-0.00; 0.00] 53.6% 53.6%
Manzano-Salgado et al., 2017 1st tr gest 7 y 535 -0.04 [-0.19; 0.11] 0.0% 0.0%
Common effect model 0.00 [-0.00; 0.00] 100.0% --
Random effects model 0.00 [-0.00; 0.00] -- 100.0%
Heterogeneity: I^2 = 0%, τ^2 = 0, p = 0.94
-0.2 -0.1 0 0.1 0.2

Study	Sampling	Outcome	Subjects	Estimate [95% CI]
Zhang, Lei, et al., 2022	within 3 d before del	7 y	98	0.000 [-0.004; 0.004]
Bloom et al., 2022	8-13 w gest	4-8 y	319	0.046 [-0.087; 0.179]
Hartman et al., 2017	15 w gest	9 y	319	-0.001 [-0.014; 0.013]
Liu et al., 2020	del	12 y	116	-0.010 [-0.290; 0.269]
Mora et al., 2017	9.6 w gest	7.7 y	420	0.002 [0.000; 0.004]
Chen et al., 2019	del	5 y	196	0.001 [-0.001; 0.002]
Manzano-Salgado et al., 2017	1st tr gest	7 y	535	-0.040 [-0.186; 0.106]
**Summary**				**-0.004 [-0.051; 0.044]**

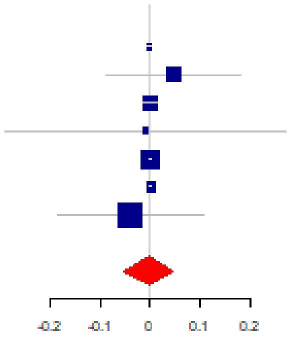

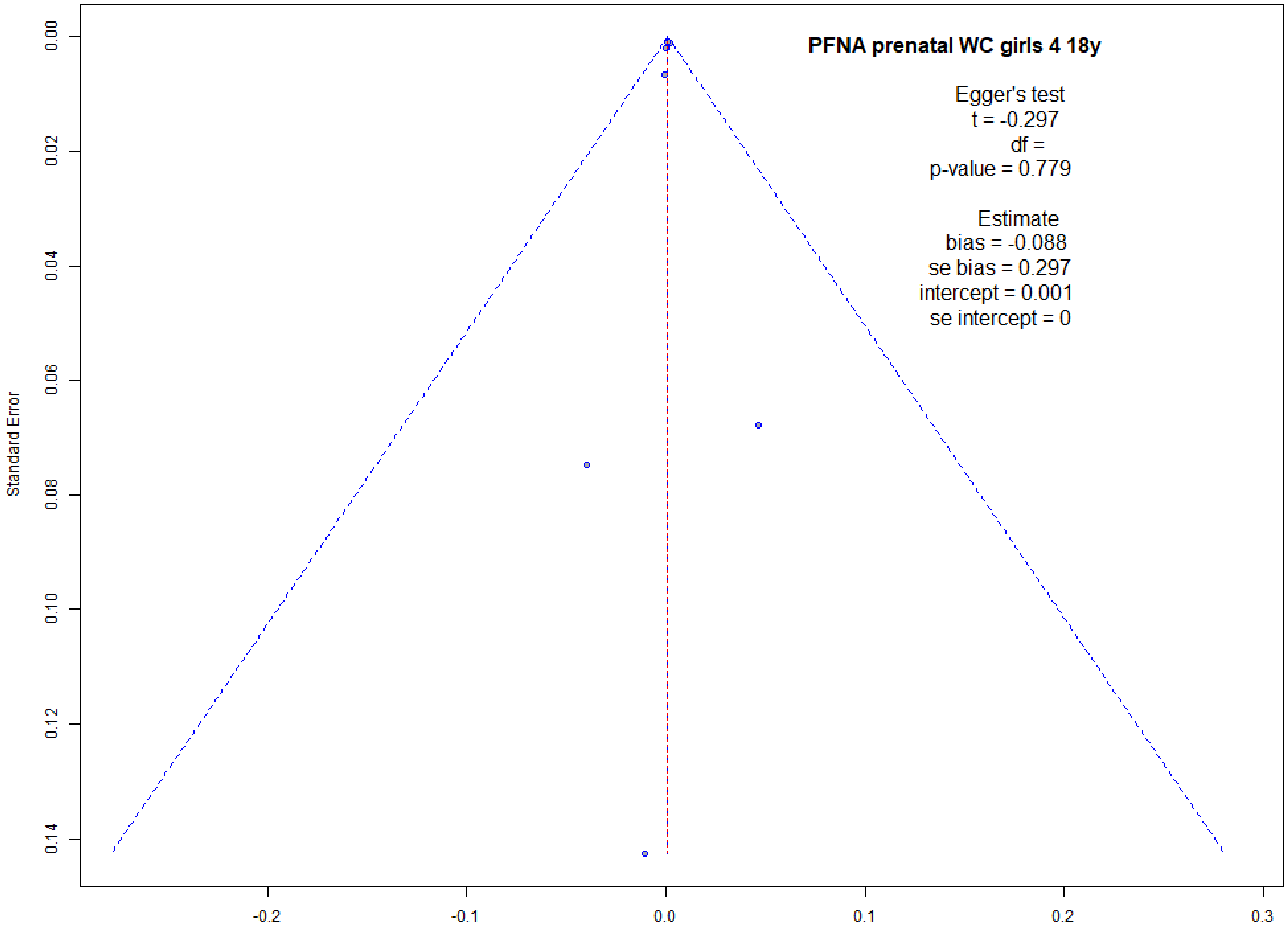
PFNA prenatal WC girls 4 18y
Egger's test
t = -0.297
df =
p-value = 0.779
Estimate
bias = -0.088
se bias = 0.297
intercept = 0.001
se intercept = 0
Standard Error
0.00
0.02
0.04
0.06
0.08
0.10
0.12
0.14
-0.2
-0.1
0.0
0.1
0.2
0.3

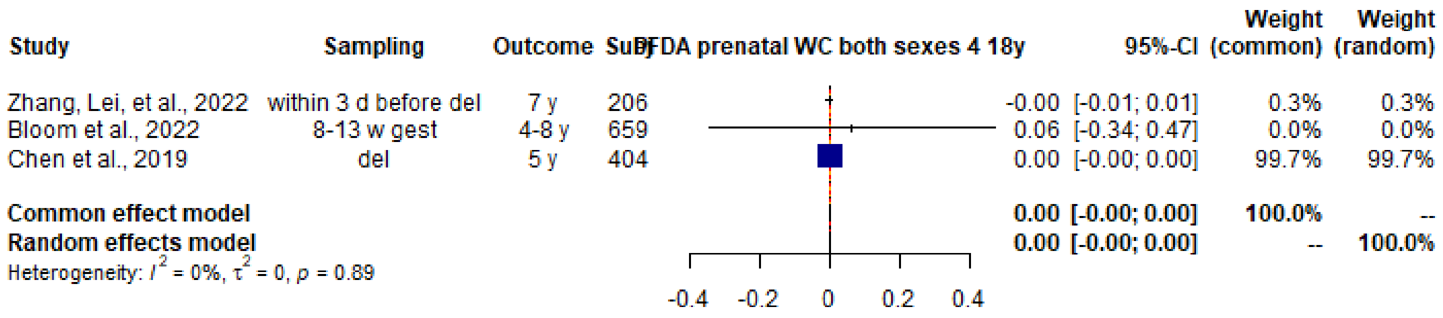
Study
Sampling
Outcome
Subj
PFDA prenatal WC both sexes 4 18y
95%-CI
Weight (common)
Weight (random)
Zhang, Lei, et al., 2022 within 3 d before del 7 y 206 -0.00 [-0.01; 0.01] 0.3% 0.3%
Bloom et al., 2022 8-13 w gest 4-8 y 659 0.06 [-0.34; 0.47] 0.0% 0.0%
Chen et al., 2019 del 5 y 404 0.00 [-0.00; 0.00] 99.7% 99.7%
Common effect model 0.00 [-0.00; 0.00] 100.0% --
Random effects model 0.00 [-0.00; 0.00] -- 100.0%
Heterogeneity: I^2 = 0%, τ^2 = 0, p = 0.89
-0.4
-0.2
0
0.2
0.4

Study	Sampling	Outcome	Subjects	Estimate [95% CI]
Zhang, Lei, et al., 2022	within 3 d before del	7 y	206	-0.001 [-0.011; 0.008]
Bloom et al., 2022	8-13 w gest	4-8 y	659	0.063 [-0.345; 0.470]
Chen et al., 2019	del	5 y	404	0.000 [0.000; 0.001]
**Summary**				**0.032 [-0.179; 0.244]**

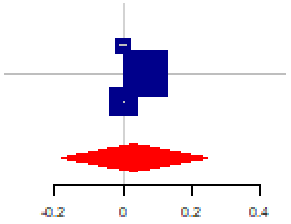

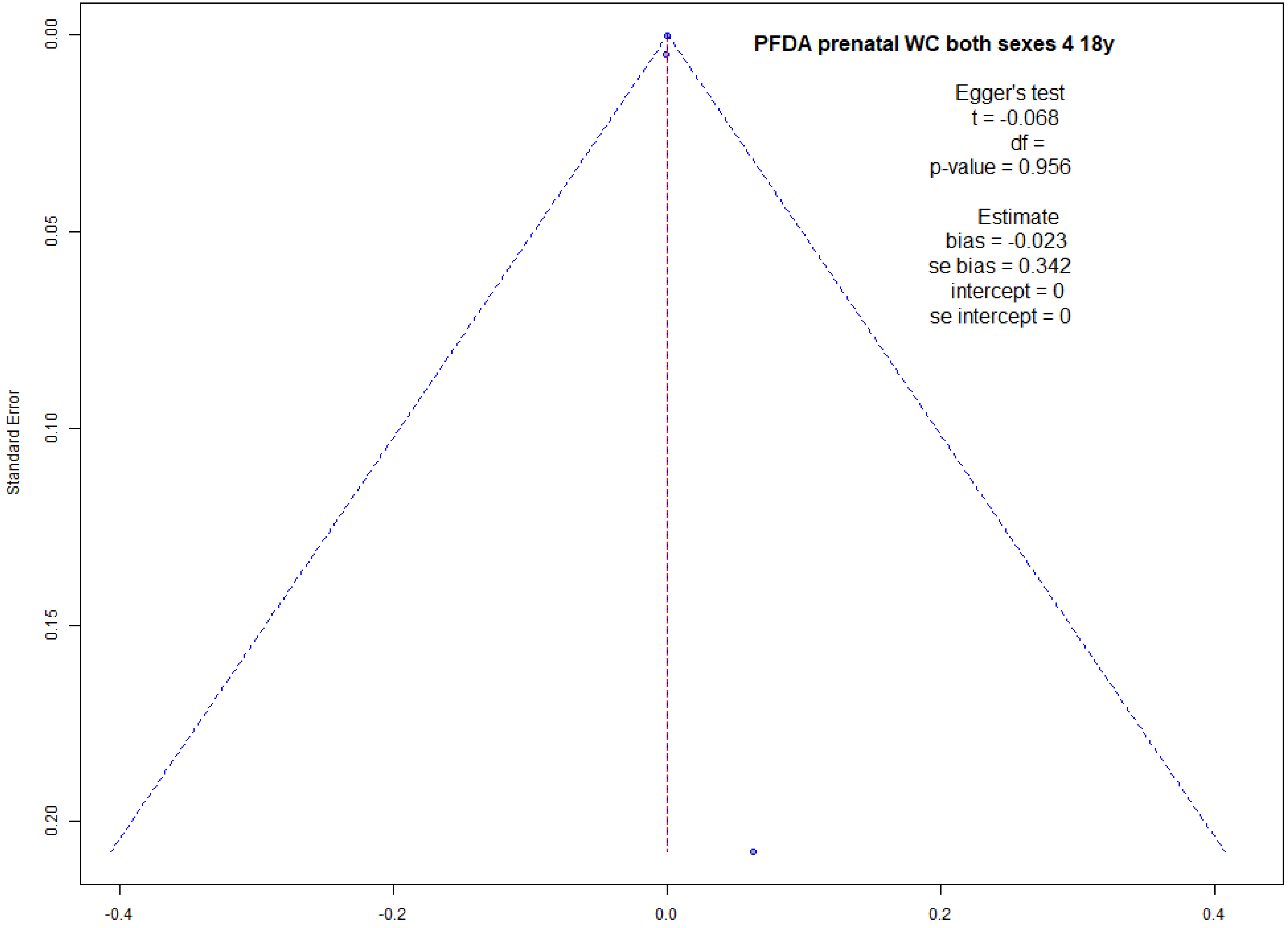
PFDA prenatal WC both sexes 4 18y
Egger's test
t = -0.068
df =
p-value = 0.956
Estimate
bias = -0.023
se bias = 0.342
intercept = 0
se intercept = 0
Standard Error
0.00
0.05
0.10
0.15
0.20
-0.4
-0.2
0.0
0.2
0.4

Study	Sampling	Outcome	Subj	PFDA prenatal WC boys 4 18y	95%-CI	Weight (common)	Weight (random)
Zhang, Lei, et al., 2022	within 3 d before del	7 y	108	-0.01	[-0.04; 0.02]	0.0%	0.0%
Bloom et al., 2022	8-13 w gest	4-8 y	340	0.06	[-0.60; 0.72]	0.0%	0.0%
Chen et al., 2019	del	5 y	208	-0.00	[-0.00; 0.00]	100.0%	100.0%
**Common effect model**				**-0.00**	**[-0.00; 0.00]**	**100.0%**	**--**
**Random effects model**				**-0.00**	**[-0.00; 0.00]**	**--**	**100.0%**

Heterogeneity: $I^2 = 0\%$, $\tau^2 = 0$, $p = 0.87$

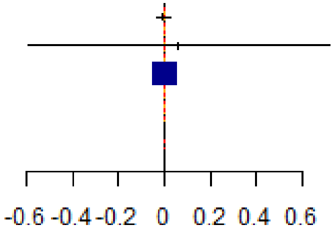

Study	Sampling	Outcome	Subjects	Estimate [95% CI]
Zhang, Lei, et al., 2022	within 3 d before del	7 y	108	-0.008 [-0.038; 0.023]
Bloom et al., 2022	8-13 w gest	4-8 y	340	0.063 [-0.595; 0.720]
Chen et al., 2019	del	5 y	208	0.000 [0.000; 0.000]
**Summary**				**0.031 [-0.310; 0.372]**

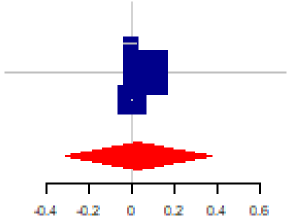

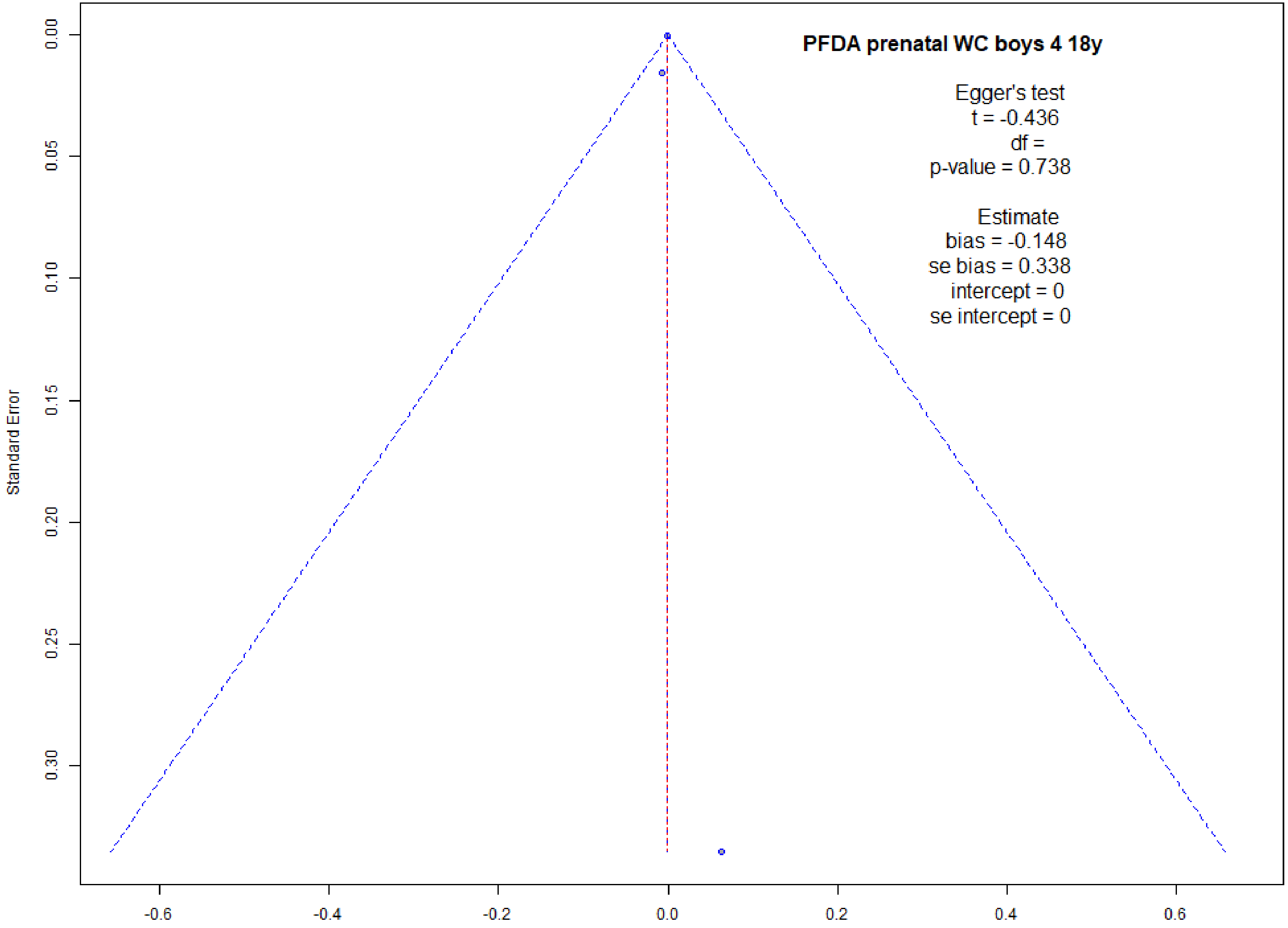
PFDA prenatal WC boys 4 18y
Egger's test
t = -0.436
df =
p-value = 0.738
Estimate
bias = -0.148
se bias = 0.338
intercept = 0
se intercept = 0
Standard Error
0.00
0.05
0.10
0.15
0.20
0.25
0.30
-0.6
-0.4
-0.2
0.0
0.2
0.4
0.6

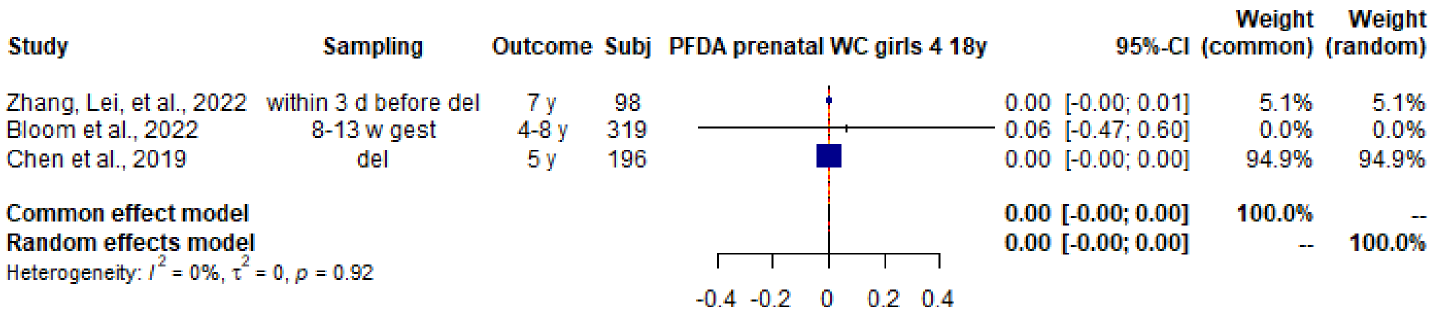
Study
Sampling
Outcome
Subj
PFDA prenatal WC girls 4 18y
95%-CI
Weight (common)
Weight (random)
Zhang, Lei, et al., 2022 within 3 d before del 7 y 98 0.00 [-0.00; 0.01] 5.1% 5.1%
Bloom et al., 2022 8-13 w gest 4-8 y 319 0.06 [-0.47; 0.60] 0.0% 0.0%
Chen et al., 2019 del 5 y 196 0.00 [-0.00; 0.00] 94.9% 94.9%
Common effect model 0.00 [-0.00; 0.00] 100.0% --
Random effects model 0.00 [-0.00; 0.00] -- 100.0%
Heterogeneity: I^2 = 0%, τ^2 = 0, p = 0.92
-0.4 -0.2 0 0.2 0.4

Study	Sampling	Outcome	Subjects	Estimate [95% CI]
Zhang, Lei, et al., 2022	within 3 d before del	7 y	98	0.000 [-0.005; 0.005]
Bloom et al., 2022	8-13 w gest	4-8 y	319	0.063 [-0.470; 0.595]
Chen et al., 2019	del	5 y	196	0.001 [0.000; 0.002]
**Summary**				**0.033 [-0.244; 0.310]**

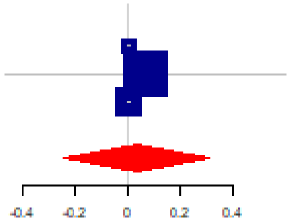

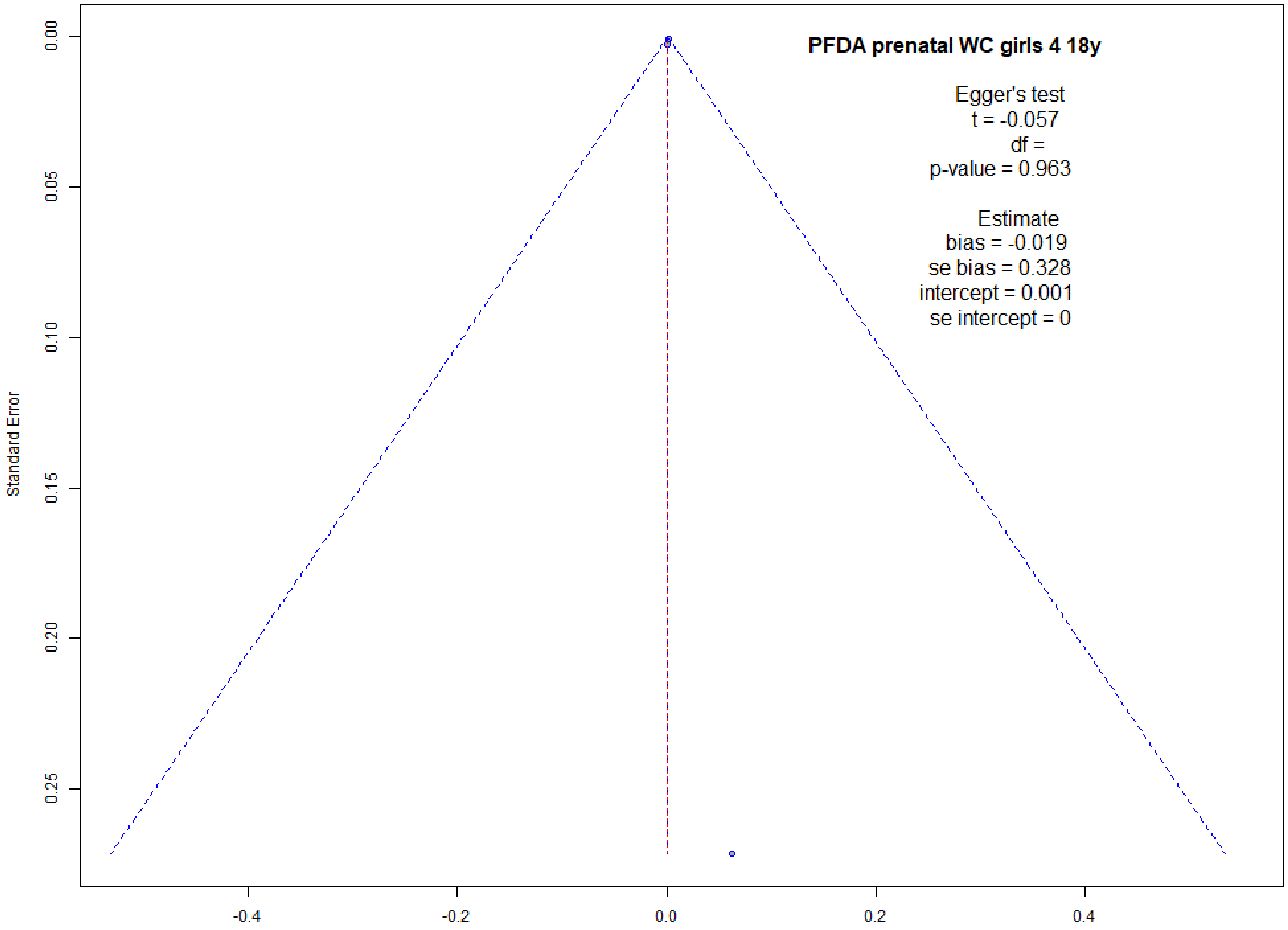
PFDA prenatal WC girls 4 18y
Egger's test
t = -0.057
df =
p-value = 0.963
Estimate
bias = -0.019
se bias = 0.328
intercept = 0.001
se intercept = 0
Standard Error
0.00
0.05
0.10
0.15
0.20
0.25
-0.4
-0.2
0.0
0.2
0.4

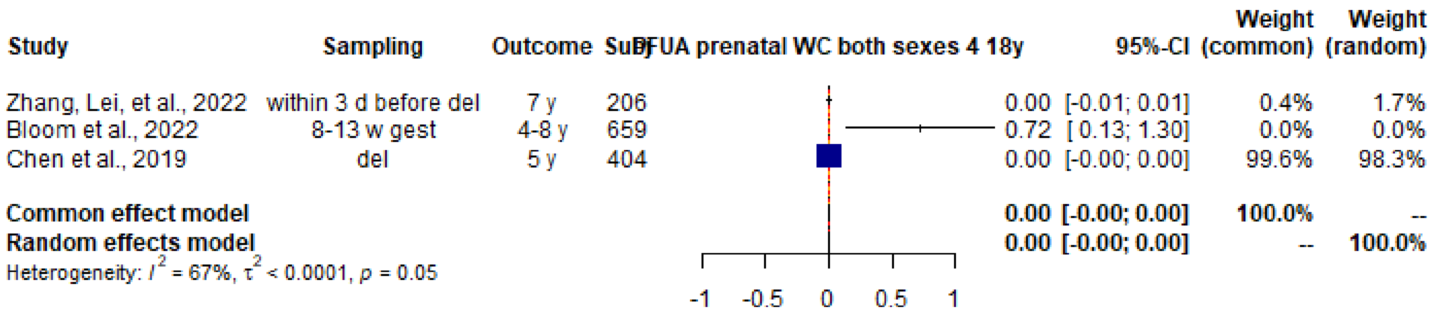
Study
Sampling
Outcome
Subj
PFUA prenatal WC both sexes 4 18y
95%-CI
Weight (common)
Weight (random)
Zhang, Lei, et al., 2022 within 3 d before del 7 y 206 0.00 [-0.01; 0.01] 0.4% 1.7%
Bloom et al., 2022 8-13 w gest 4-8 y 659 0.72 [0.13; 1.30] 0.0% 0.0%
Chen et al., 2019 del 5 y 404 0.00 [-0.00; 0.00] 99.6% 98.3%
Common effect model 0.00 [-0.00; 0.00] 100.0% --
Random effects model 0.00 [-0.00; 0.00] -- 100.0%
Heterogeneity: I^2 = 67%, τ^2 < 0.0001, p = 0.05
-1
-0.5
0
0.5
1

Study	Sampling	Outcome	Subjects	Estimate [95% CI]
Zhang, Lei, et al., 2022	within 3 d before del	7 y	206	0.003 [-0.008; 0.015]
Bloom et al., 2022	8-13 w gest	4-8 y	659	0.717 [0.134; 1.299]
Chen et al., 2019	del	5 y	404	0.000 [0.000; 0.001]
**Summary**				**0.373 [0.070; 0.675]**

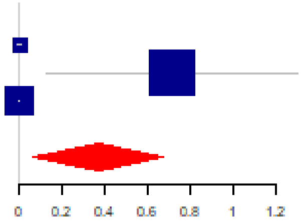

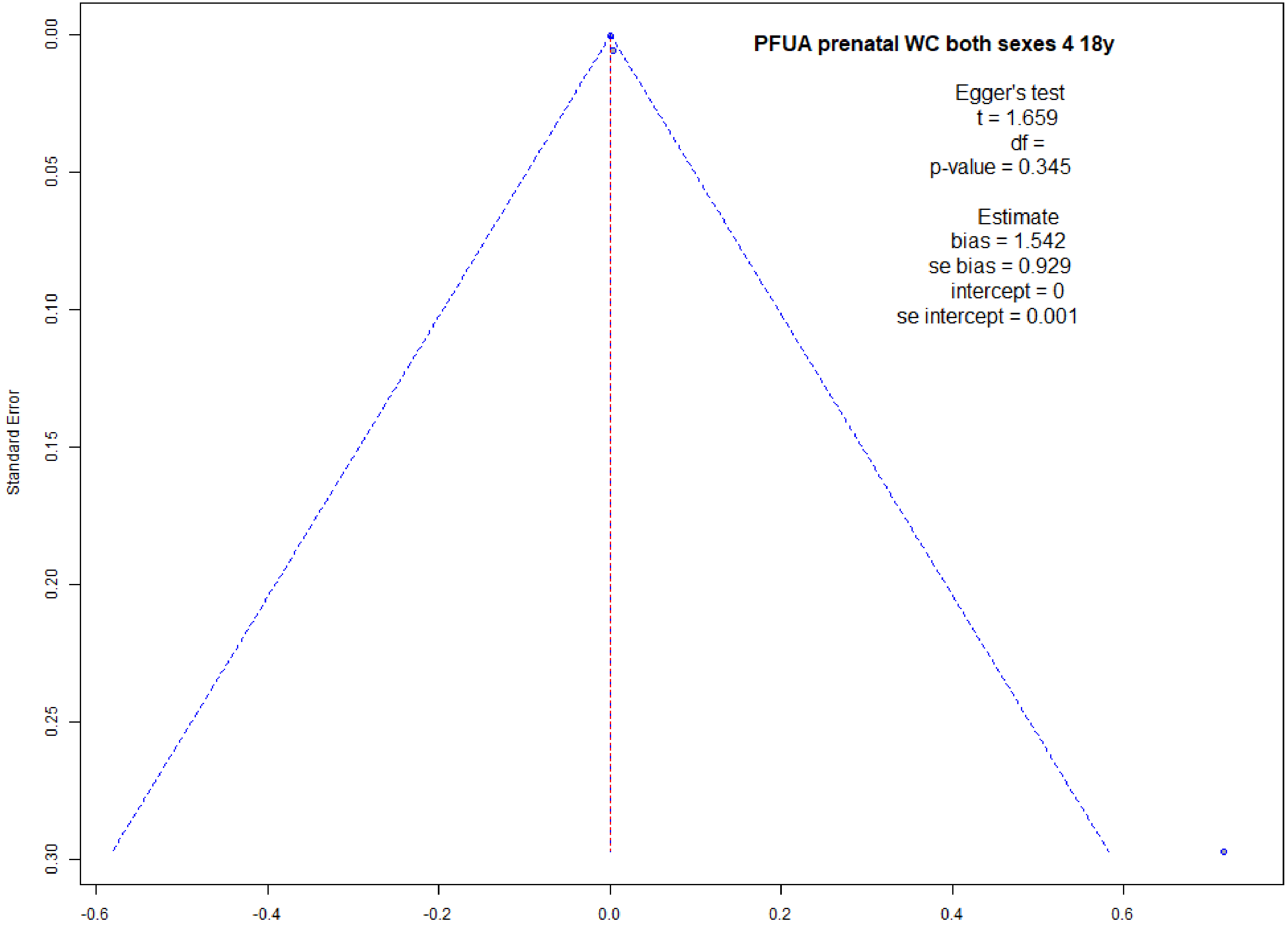
PFUA prenatal WC both sexes 4 18y
Egger's test
t = 1.659
df =
p-value = 0.345
Estimate
bias = 1.542
se bias = 0.929
intercept = 0
se intercept = 0.001
Standard Error
0.00
0.05
0.10
0.15
0.20
0.25
0.30
-0.6
-0.4
-0.2
0.0
0.2
0.4
0.6

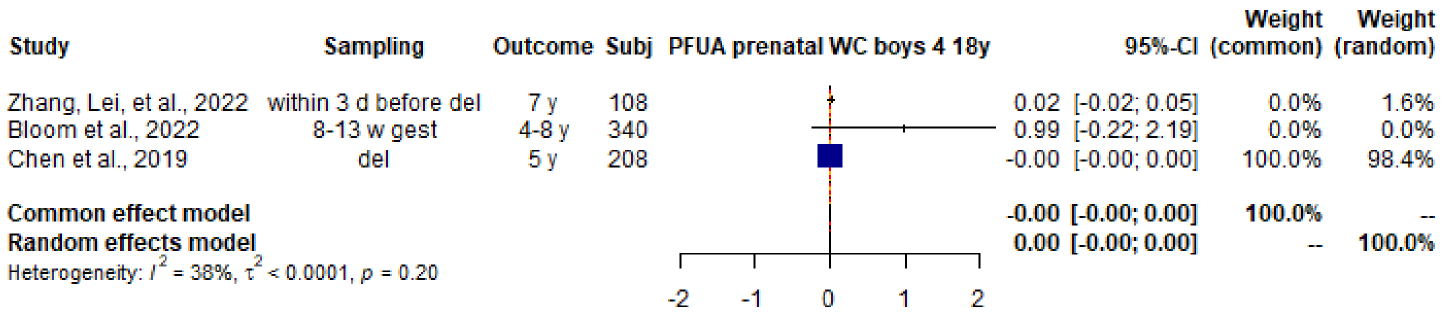
Study
Sampling
Outcome
Subj
PFUA prenatal WC boys 4 18y
95%-CI
Weight (common)
Weight (random)
Zhang, Lei, et al., 2022 within 3 d before del 7 y 108 0.02 [-0.02; 0.05] 0.0% 1.6%
Bloom et al., 2022 8-13 w gest 4-8 y 340 0.99 [-0.22; 2.19] 0.0% 0.0%
Chen et al., 2019 del 5 y 208 -0.00 [-0.00; 0.00] 100.0% 98.4%
Common effect model -0.00 [-0.00; 0.00] 100.0% --
Random effects model 0.00 [-0.00; 0.00] -- 100.0%
Heterogeneity: I^2 = 38%, τ^2 < 0.0001, p = 0.20
-2
-1
0
1
2

Study	Sampling	Outcome	Subjects	Estimate [95% CI]
Zhang, Lei, et al., 2022	within 3 d before del	7 y	108	0.015 [-0.021; 0.051]
Bloom et al., 2022	8-13 w gest	4-8 y	340	0.985 [-0.224; 2.195]
Chen et al., 2019	del	5 y	208	0.000 [-0.001; 0.000]
**Summary**				**0.513 [-0.114; 1.140]**

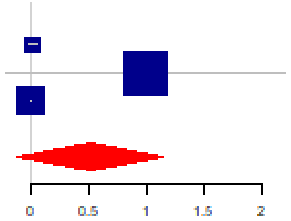

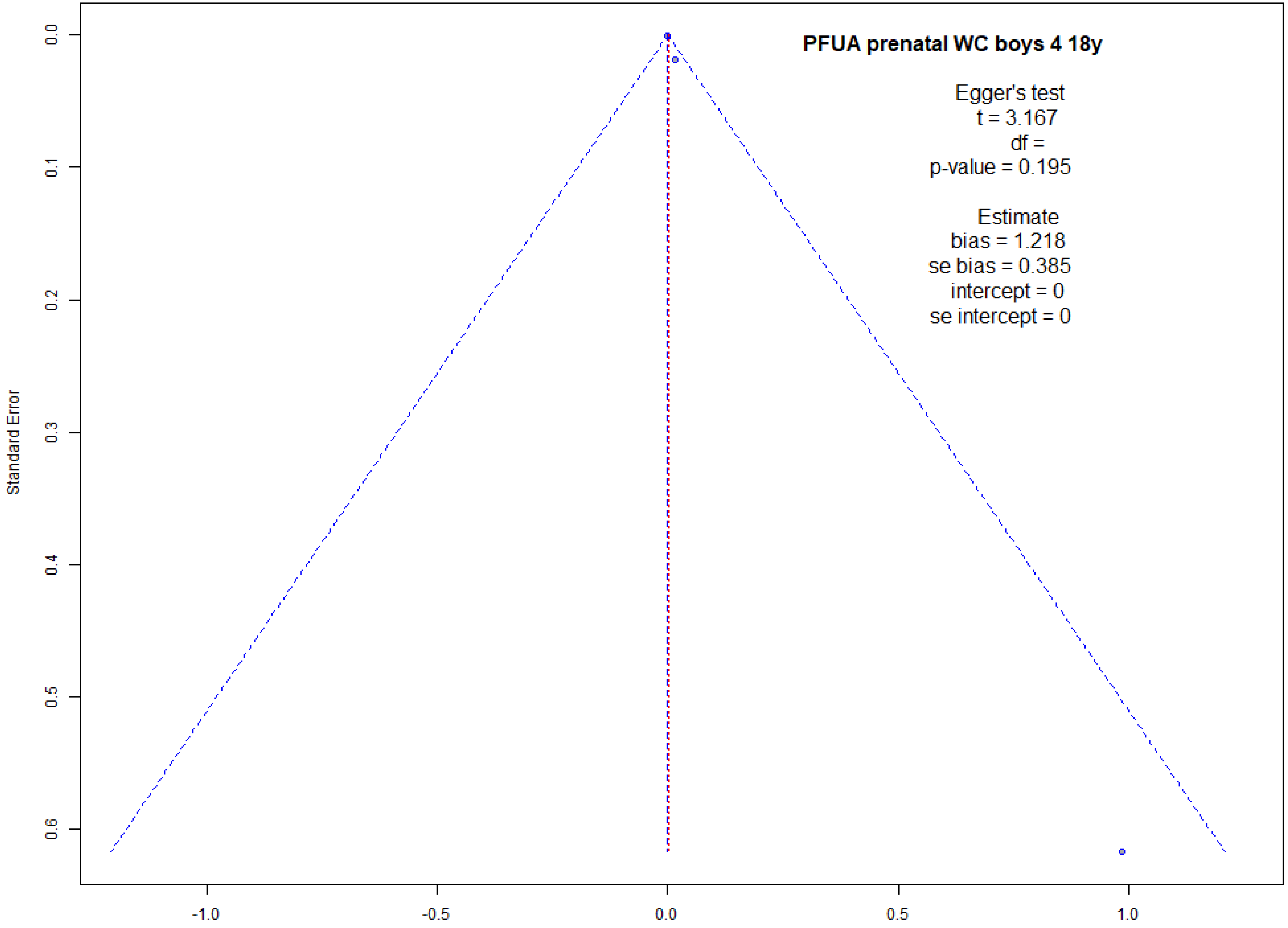
PFUA prenatal WC boys 4 18y
Egger's test
t = 3.167
df =
p-value = 0.195
Estimate
bias = 1.218
se bias = 0.385
intercept = 0
se intercept = 0
Standard Error
0.0
0.1
0.2
0.3
0.4
0.5
0.6
-1.0
-0.5
0.0
0.5
1.0

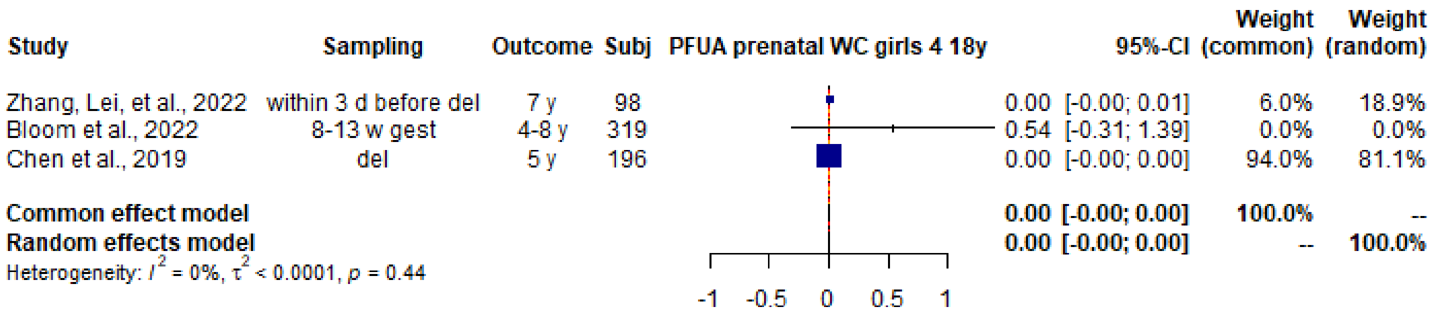
Study
Sampling
Outcome
Subj
PFUA prenatal WC girls 4 18y
95%-CI
Weight (common)
Weight (random)
Zhang, Lei, et al., 2022 within 3 d before del 7 y 98 0.00 [-0.00; 0.01] 6.0% 18.9%
Bloom et al., 2022 8-13 w gest 4-8 y 319 0.54 [-0.31; 1.39] 0.0% 0.0%
Chen et al., 2019 del 5 y 196 0.00 [-0.00; 0.00] 94.0% 81.1%
Common effect model 0.00 [-0.00; 0.00] 100.0% --
Random effects model 0.00 [-0.00; 0.00] -- 100.0%
Heterogeneity: I^2 = 0%, τ^2 < 0.0001, p = 0.44
-1
-0.5
0
0.5
1

Study	Sampling	Outcome	Subjects	Estimate [95% CI]
Zhang, Lei, et al., 2022	within 3 d before del	7 y	98	0.002 [-0.004; 0.009]
Bloom et al., 2022	8-13 w gest	4-8 y	319	0.538 [-0.314; 1.389]
Chen et al., 2019	del	5 y	196	0.001 [0.000; 0.003]
**Summary**				**0.281 [-0.162; 0.723]**

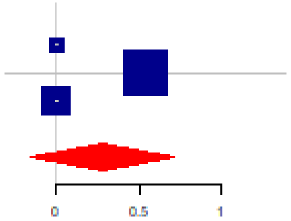

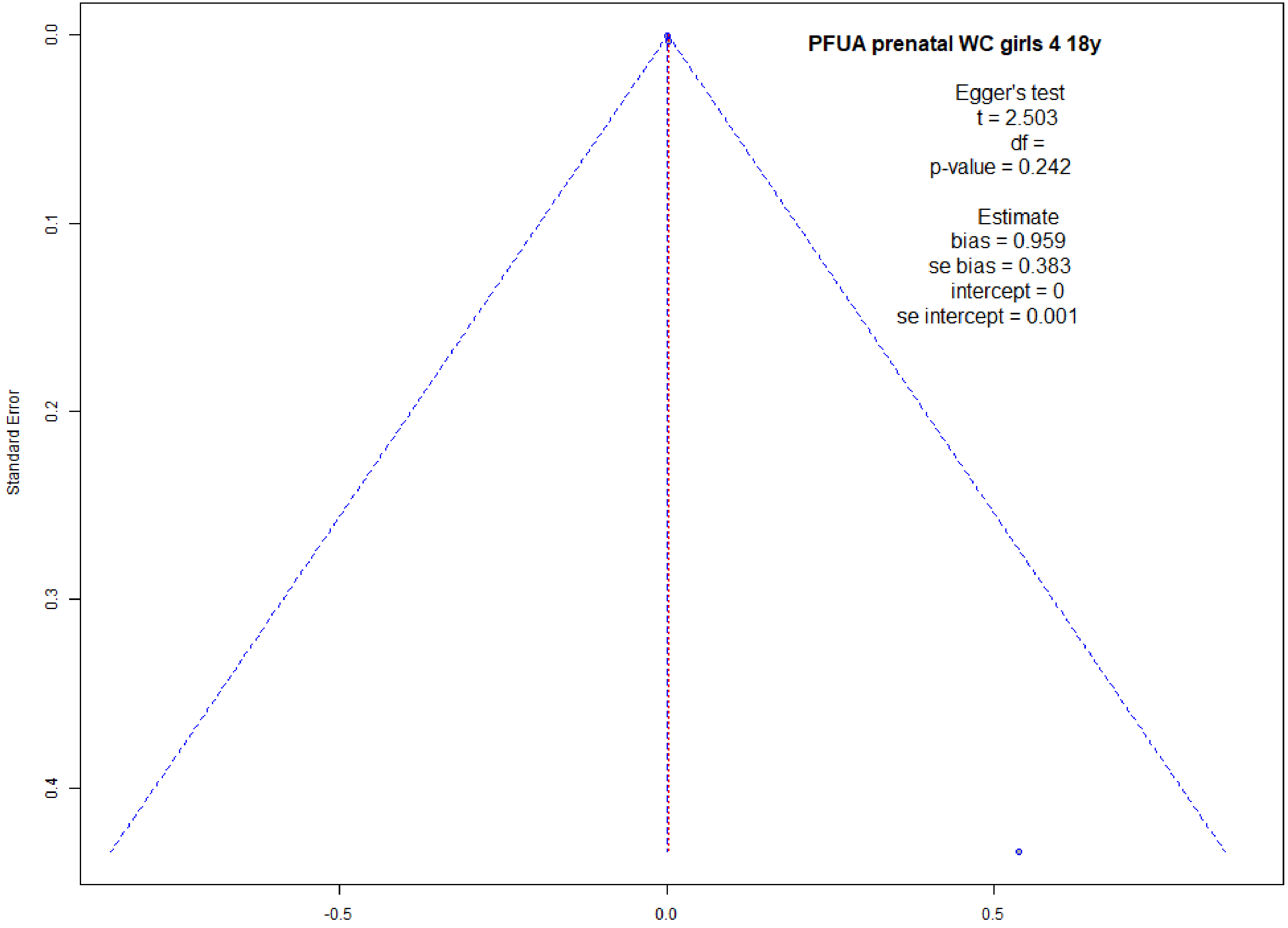
PFUA prenatal WC girls 4 18y
Egger's test
t = 2.503
df =
p-value = 0.242
Estimate
bias = 0.959
se bias = 0.383
intercept = 0
se intercept = 0.001
Standard Error
0.0
0.1
0.2
0.3
0.4
-0.5
0.0
0.5

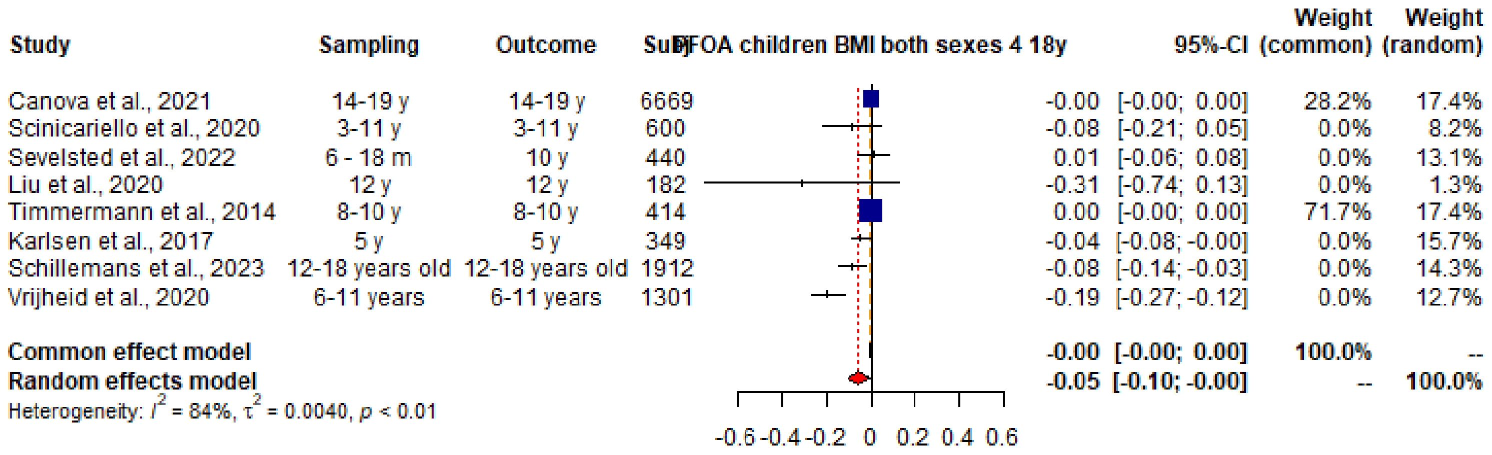
PFOA children BMI both sexes 4 18y
Study
Sampling
Outcome
95%-CI
Weight (common)
Weight (random)
Canova et al., 2021 | 14-19 y | 14-19 y | 6669 | -0.00 [-0.00; 0.00] | 28.2% | 17.4%
Scinicariello et al., 2020 | 3-11 y | 3-11 y | 600 | -0.08 [-0.21; 0.05] | 0.0% | 8.2%
Sevelsted et al., 2022 | 6 - 18 m | 10 y | 440 | 0.01 [-0.06; 0.08] | 0.0% | 13.1%
Liu et al., 2020 | 12 y | 12 y | 182 | -0.31 [-0.74; 0.13] | 0.0% | 1.3%
Timmermann et al., 2014 | 8-10 y | 8-10 y | 414 | 0.00 [-0.00; 0.00] | 71.7% | 17.4%
Karlsen et al., 2017 | 5 y | 5 y | 349 | -0.04 [-0.08; -0.00] | 0.0% | 15.7%
Schillemans et al., 2023 | 12-18 years old | 12-18 years old | 1912 | -0.08 [-0.14; -0.03] | 0.0% | 14.3%
Vrijheid et al., 2020 | 6-11 years | 6-11 years | 1301 | -0.19 [-0.27; -0.12] | 0.0% | 12.7%
Common effect model | -0.00 [-0.00; 0.00] | 100.0% | --
Random effects model | -0.05 [-0.10; -0.00] | -- | 100.0%
Heterogeneity: I^2 = 84%, τ^2 = 0.0040, p < 0.01
-0.6 -0.4 -0.2 0 0.2 0.4 0.6

Study	Sampling	Outcome	Subjects	Estimate [95% CI]
Canova et al., 2021	14-19 y	14-19 y	6669	-0.001 [-0.001; 0.000]
Scinicariello et al., 2020	3-11 y	3-11 y	600	-0.080 [-0.210; 0.050]
Sevelsted et al., 2022	6 - 18 m	10 y	440	0.010 [-0.060; 0.080]
Liu et al., 2020	12 y	12 y	182	-0.306 [-0.741; 0.129]
Timmermann et al., 2014	8-10 y	8-10 y	414	0.000 [0.000; 0.001]
Karlsen et al., 2017	5 y	5 y	349	-0.044 [-0.085; -0.003]
Schillemans et al., 2023	12-18 years old	12-18 years old	1912	-0.083 [-0.141; -0.026]
Vrijheid et al., 2020	6-11 years	6-11 years	1301	-0.193 [-0.267; -0.118]
**Summary**				**-0.045 [-0.060; -0.029]**

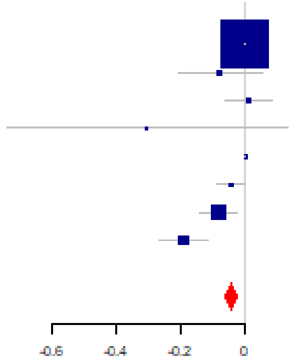

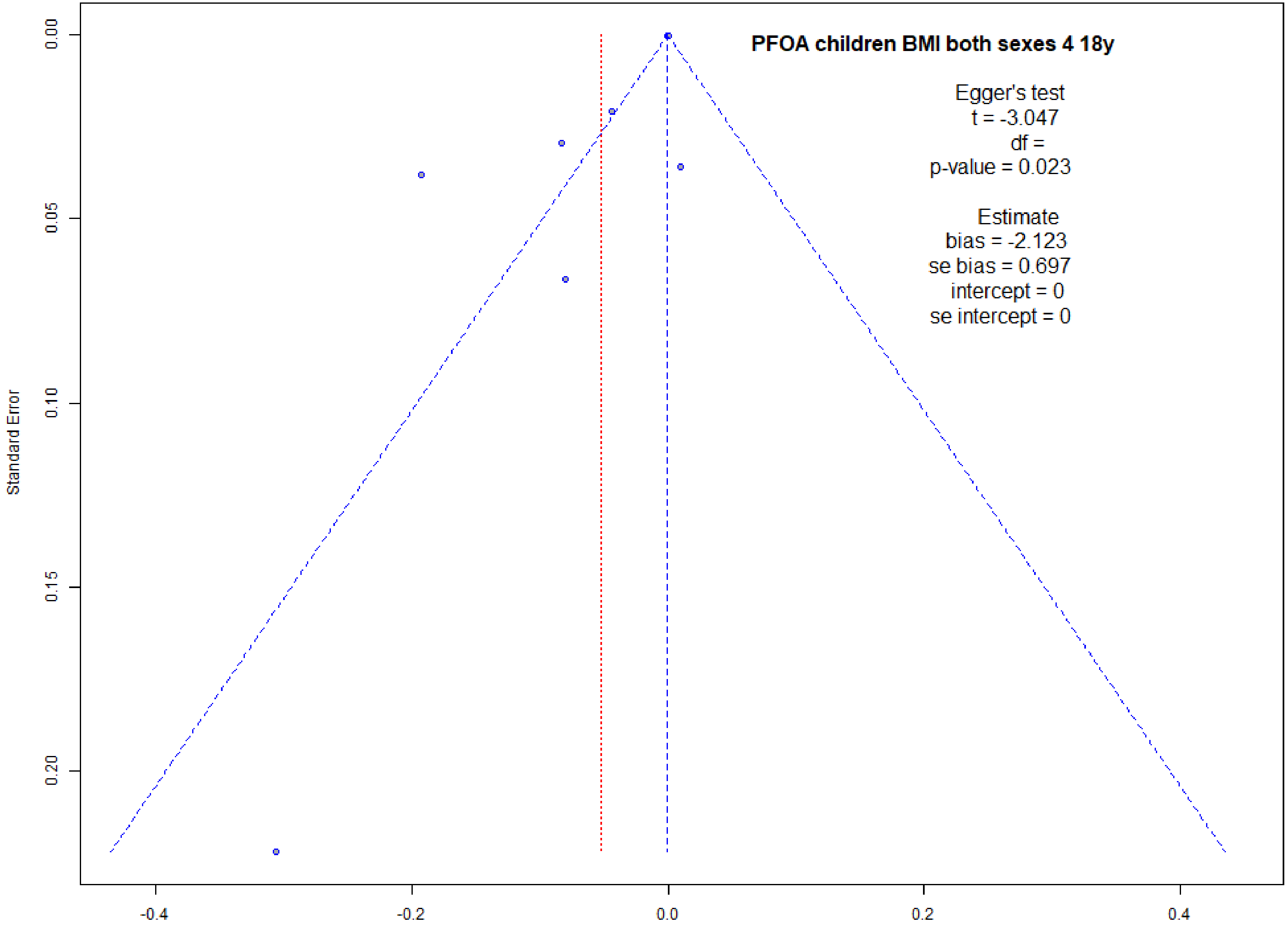
PFOA children BMI both sexes 4 18y
Egger's test
t = -3.047
df =
p-value = 0.023
Estimate
bias = -2.123
se bias = 0.697
intercept = 0
se intercept = 0
Standard Error
0.00
0.05
0.10
0.15
0.20
-0.4
-0.2
0.0
0.2
0.4

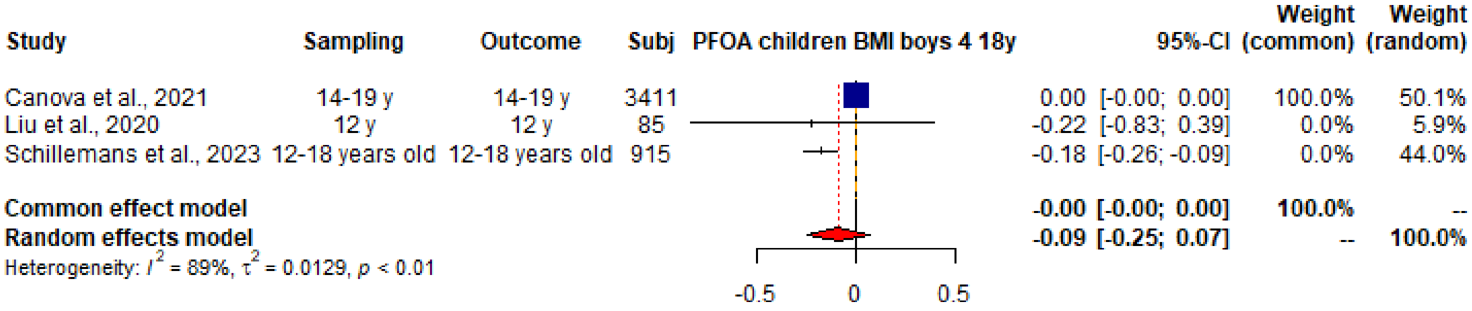
Study
Sampling
Outcome
Subj
PFOA children BMI boys 4 18y
95%-CI
Weight (common)
Weight (random)
Canova et al., 2021 14-19 y 14-19 y 3411 0.00 [-0.00; 0.00] 100.0% 50.1%
Liu et al., 2020 12 y 12 y 85 -0.22 [-0.83; 0.39] 0.0% 5.9%
Schillemans et al., 2023 12-18 years old 12-18 years old 915 -0.18 [-0.26; -0.09] 0.0% 44.0%
Common effect model -0.00 [-0.00; 0.00] 100.0% --
Random effects model -0.09 [-0.25; 0.07] -- 100.0%
Heterogeneity: I^2 = 89%, τ^2 = 0.0129, p < 0.01
-0.5
0
0.5

Study	Sampling	Outcome	Subjects	Estimate [95% CI]
Canova et al., 2021	14-19 y	14-19 y	3411	0.000 [-0.001; 0.001]
Liu et al., 2020	12 y	12 y	85	-0.222 [-0.833; 0.389]
Schillemans et al., 2023	12-18 years old	12-18 years old	915	-0.177 [-0.261; -0.094]
**Summary**				**-0.041 [-0.228; 0.146]**

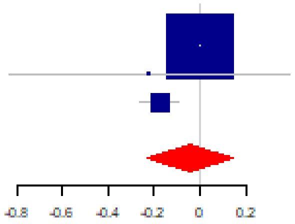

Gianfranco Frigerio, Chiara Matilde Ferrari, and Silvia Fustinoni

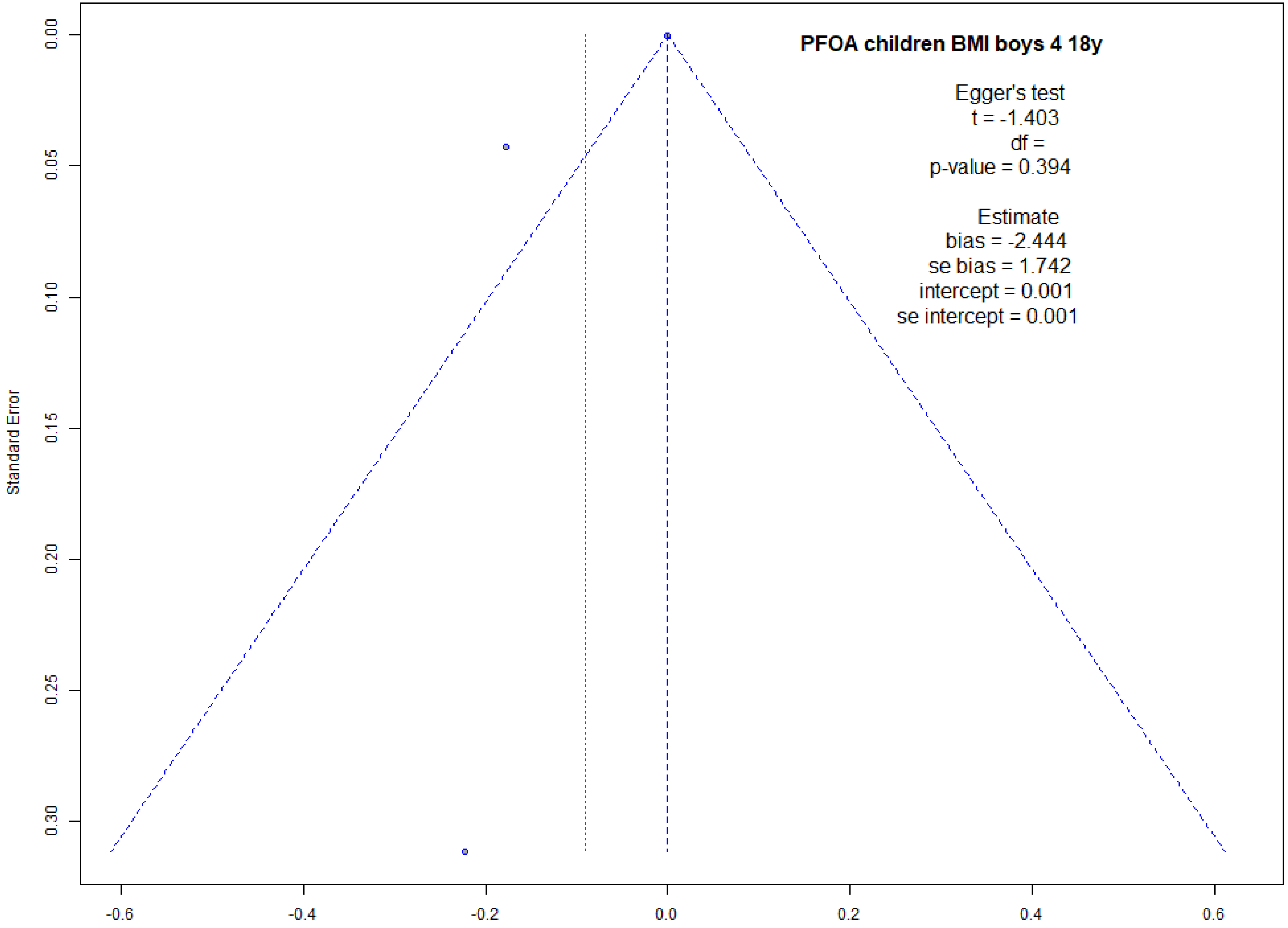

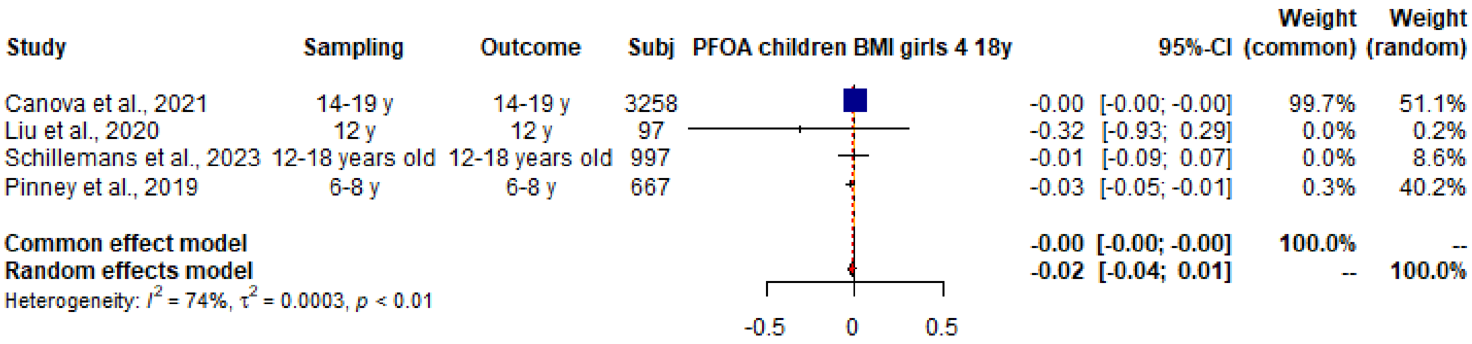
Study
Sampling
Outcome
Subj
PFOA children BMI girls 4 18y
95%-CI
Weight (common)
Weight (random)
Canova et al., 2021 14-19 y 14-19 y 3258 -0.00 [-0.00; -0.00] 99.7% 51.1%
Liu et al., 2020 12 y 12 y 97 -0.32 [-0.93; 0.29] 0.0% 0.2%
Schillemans et al., 2023 12-18 years old 12-18 years old 997 -0.01 [-0.09; 0.07] 0.0% 8.6%
Pinney et al., 2019 6-8 y 6-8 y 667 -0.03 [-0.05; -0.01] 0.3% 40.2%
Common effect model -0.00 [-0.00; -0.00] 100.0% --
Random effects model -0.02 [-0.04; 0.01] -- 100.0%
Heterogeneity: I^2 = 74%, τ^2 = 0.0003, p < 0.01
-0.5
0
0.5

Study	Sampling	Outcome	Subjects	Estimate [95% CI]
Canova et al., 2021	14-19 y	14-19 y	3258	-0.002 [-0.003; -0.001]
Liu et al., 2020	12 y	12 y	97	-0.316 [-0.926; 0.295]
Schillemans et al., 2023	12-18 years old	12-18 years old	997	-0.010 [-0.089; 0.068]
Pinney et al., 2019	6-8 y	6-8 y	667	-0.032 [-0.050; -0.013]
**Summary**				**-0.013 [-0.046; 0.019]**

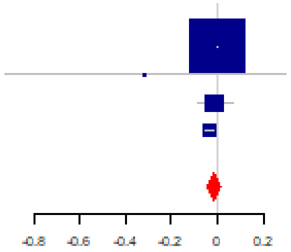

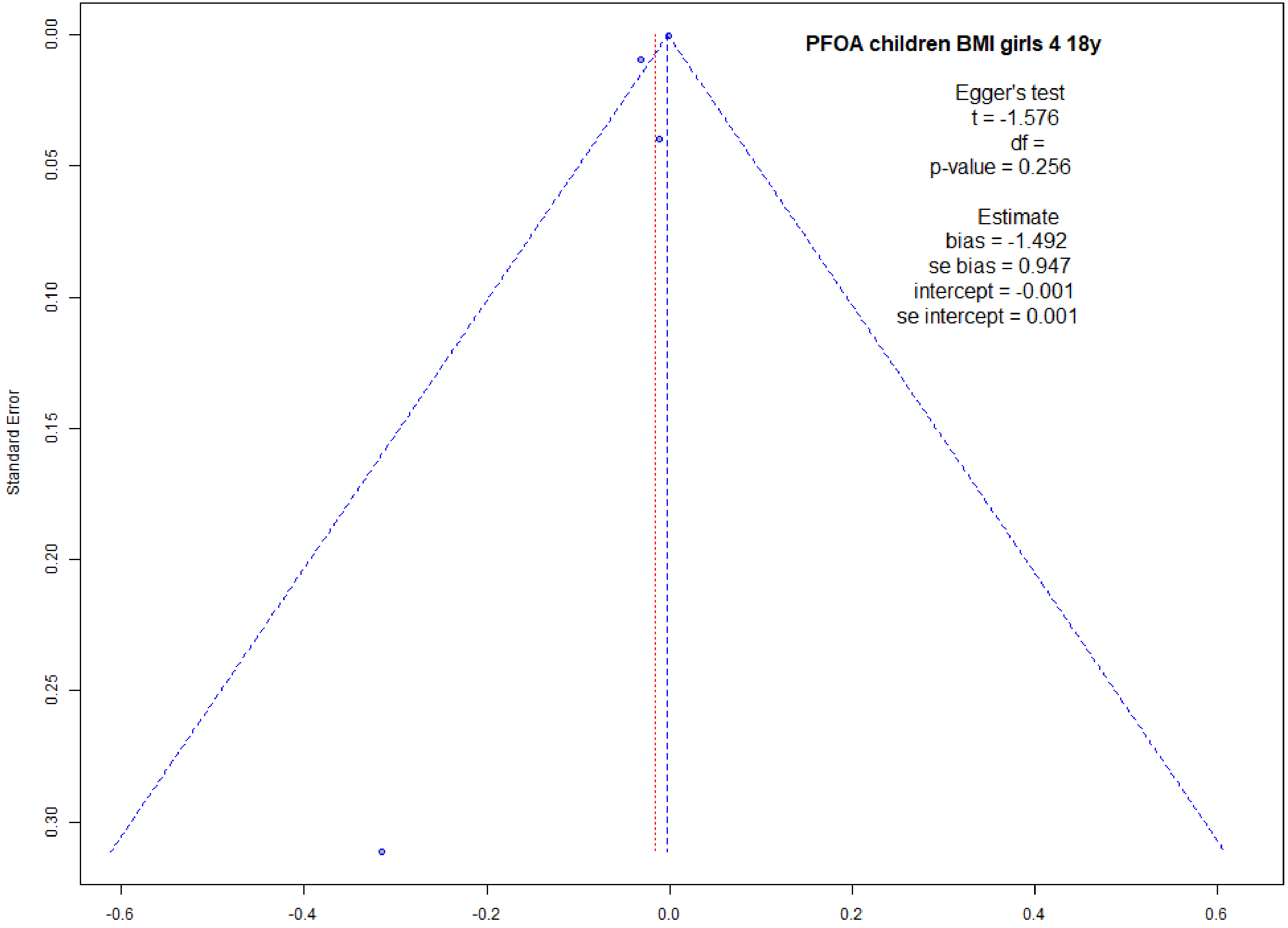
PFOA children BMI girls 4 18y
Egger's test
t = -1.576
df =
p-value = 0.256
Estimate
bias = -1.492
se bias = 0.947
intercept = -0.001
se intercept = 0.001
Standard Error
0.00
0.05
0.10
0.15
0.20
0.25
0.30
-0.6
-0.4
-0.2
0.0
0.2
0.4
0.6

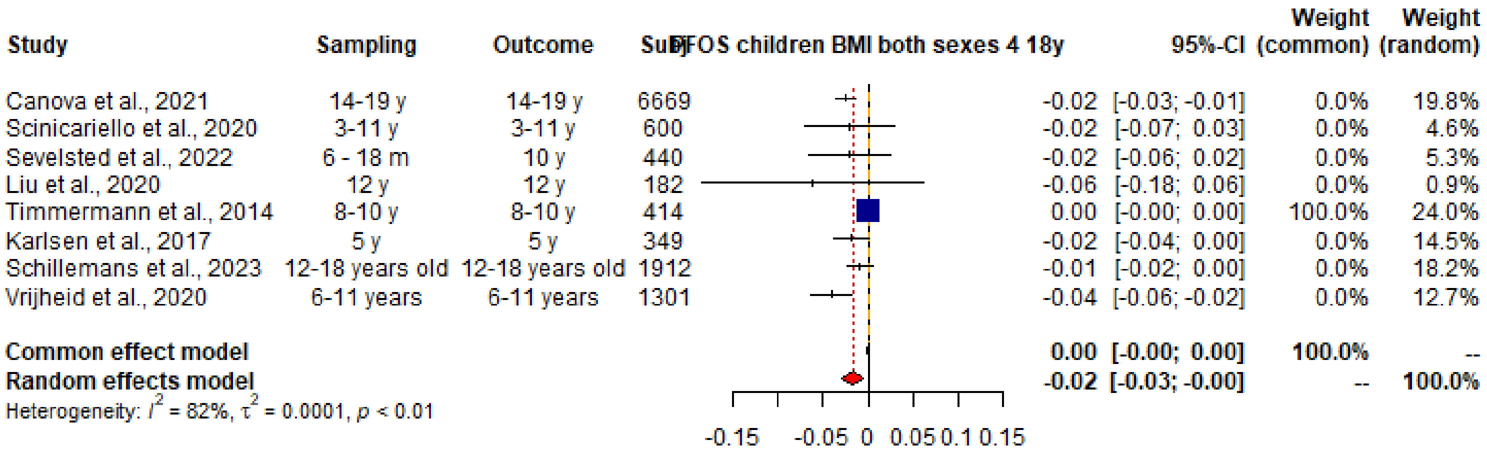

Study
Sampling
Outcome
SuPFOS children BMI both sexes 4 18y
95%-CI
Weight (common)
Weight (random)
Canova et al., 2021 14-19 y 14-19 y 6669 -0.02 [-0.03; -0.01] 0.0% 19.8%
Scinicariello et al., 2020 3-11 y 3-11 y 600 -0.02 [-0.07; 0.03] 0.0% 4.6%
Sevelsted et al., 2022 6 - 18 m 10 y 440 -0.02 [-0.06; 0.02] 0.0% 5.3%
Liu et al., 2020 12 y 12 y 182 -0.06 [-0.18; 0.06] 0.0% 0.9%
Timmermann et al., 2014 8-10 y 8-10 y 414 0.00 [-0.00; 0.00] 100.0% 24.0%
Karlsen et al., 2017 5 y 5 y 349 -0.02 [-0.04; 0.00] 0.0% 14.5%
Schillemans et al., 2023 12-18 years old 12-18 years old 1912 -0.01 [-0.02; 0.00] 0.0% 18.2%
Vrijheid et al., 2020 6-11 years 6-11 years 1301 -0.04 [-0.06; -0.02] 0.0% 12.7%
Common effect model 0.00 [-0.00; 0.00] 100.0% --
Random effects model -0.02 [-0.03; -0.00] -- 100.0%
Heterogeneity: I^2 = 82%, τ^2 = 0.0001, p < 0.01
-0.15 -0.05 0 0.05 0.1 0.15

Study	Sampling	Outcome	Subjects	Estimate [95% CI]
Canova et al., 2021	14-19 y	14-19 y	6669	-0.024 [-0.035; -0.013]
Scinicariello et al., 2020	3-11 y	3-11 y	600	-0.021 [-0.070; 0.029]
Sevelsted et al., 2022	6 - 18 m	10 y	440	-0.020 [-0.065; 0.025]
Liu et al., 2020	12 y	12 y	182	-0.061 [-0.183; 0.061]
Timmermann et al., 2014	8-10 y	8-10 y	414	0.000 [0.000; 0.000]
Karlsen et al., 2017	5 y	5 y	349	-0.018 [-0.037; 0.002]
Schillemans et al., 2023	12-18 years old	12-18 years old	1912	-0.009 [-0.022; 0.004]
Vrijheid et al., 2020	6-11 years	6-11 years	1301	-0.040 [-0.063; -0.018]
**Summary**				**-0.023 [-0.041; -0.004]**

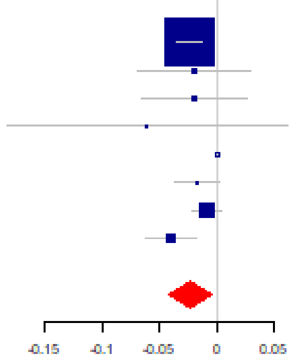

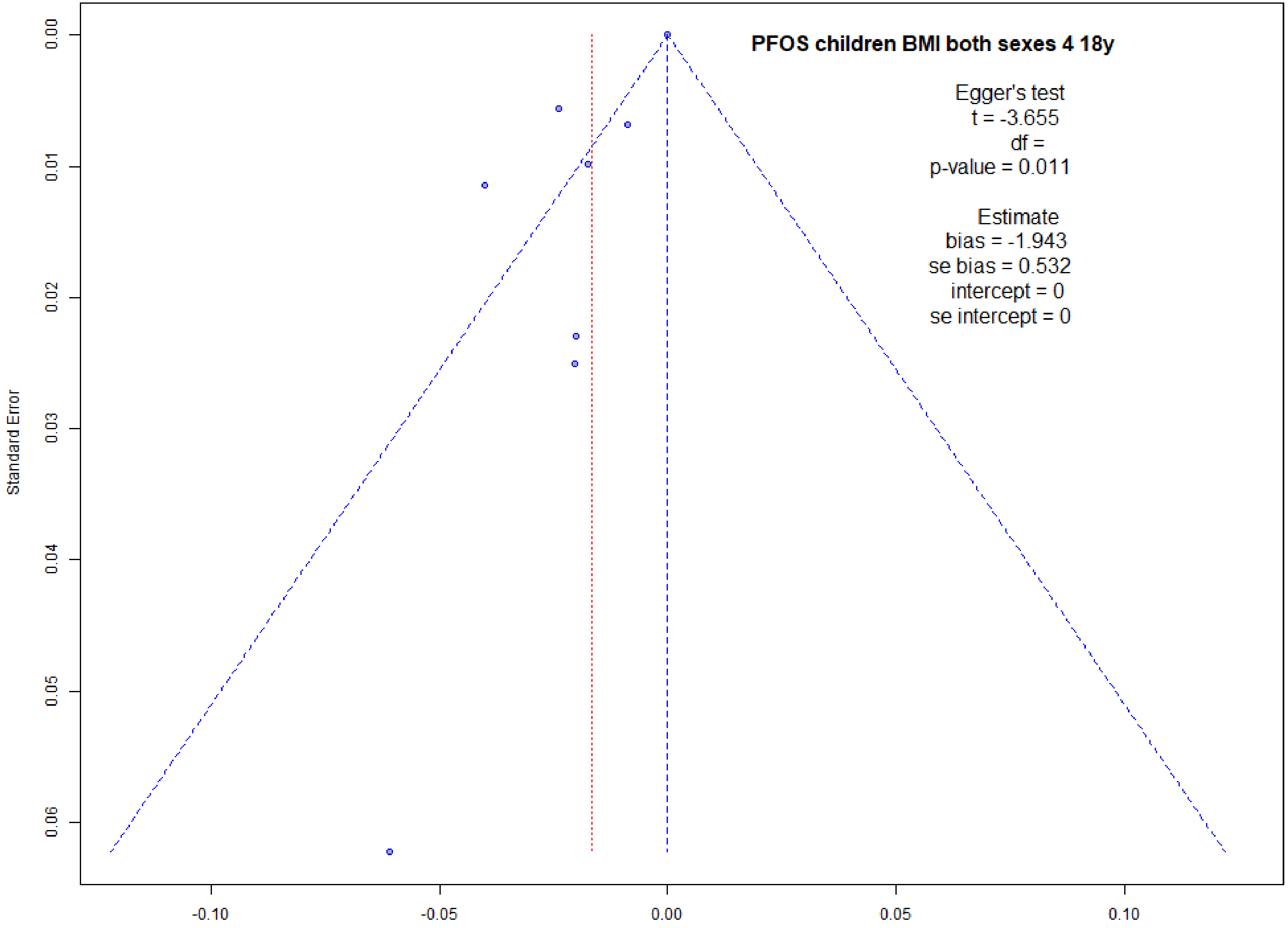
PFOS children BMI both sexes 4 18y
Egger's test
t = -3.655
df =
p-value = 0.011
Estimate
bias = -1.943
se bias = 0.532
intercept = 0
se intercept = 0
Standard Error
0.00
0.01
0.02
0.03
0.04
0.05
0.06
-0.10
-0.05
0.00
0.05
0.10

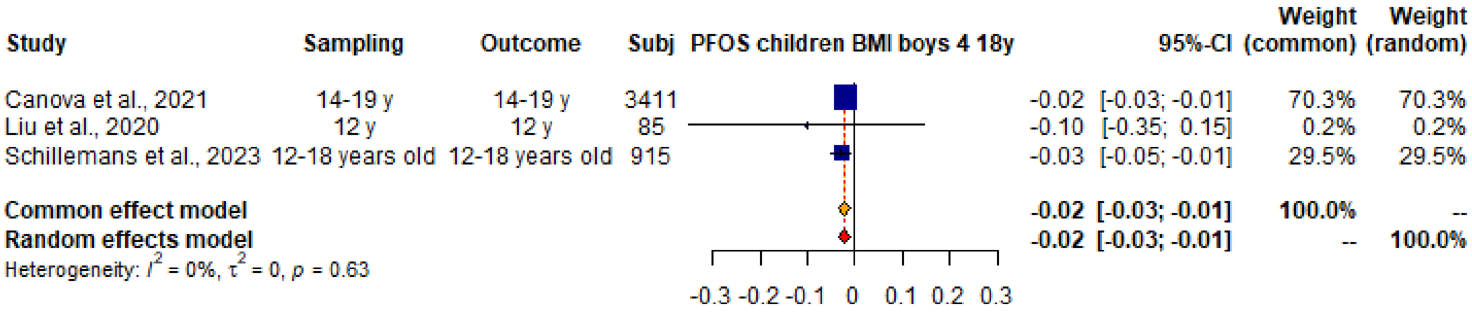
Study
Sampling
Outcome
Subj
PFOS children BMI boys 4 18y
95%-CI
Weight (common)
Weight (random)
Canova et al., 2021 | 14-19 y | 14-19 y | 3411 | -0.02 [-0.03; -0.01] | 70.3% | 70.3%
Liu et al., 2020 | 12 y | 12 y | 85 | -0.10 [-0.35; 0.15] | 0.2% | 0.2%
Schillemans et al., 2023 | 12-18 years old | 12-18 years old | 915 | -0.03 [-0.05; -0.01] | 29.5% | 29.5%
Common effect model | -0.02 [-0.03; -0.01] | 100.0% | --
Random effects model | -0.02 [-0.03; -0.01] | -- | 100.0%
Heterogeneity: I^2 = 0%, τ^2 = 0, p = 0.63
-0.3 -0.2 -0.1 0 0.1 0.2 0.3

Study	Sampling	Outcome	Subjects	Estimate [95% CI]
Canova et al., 2021	14-19 y	14-19 y	3411	-0.020 [-0.033; -0.007]
Liu et al., 2020	12 y	12 y	85	-0.101 [-0.348; 0.146]
Schillemans et al., 2023	12-18 years old	12-18 years old	915	-0.029 [-0.049; -0.009]
**Summary**				**-0.024 [-0.035; -0.012]**

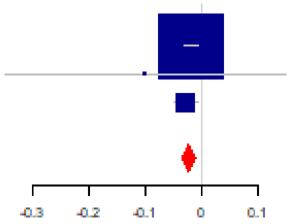

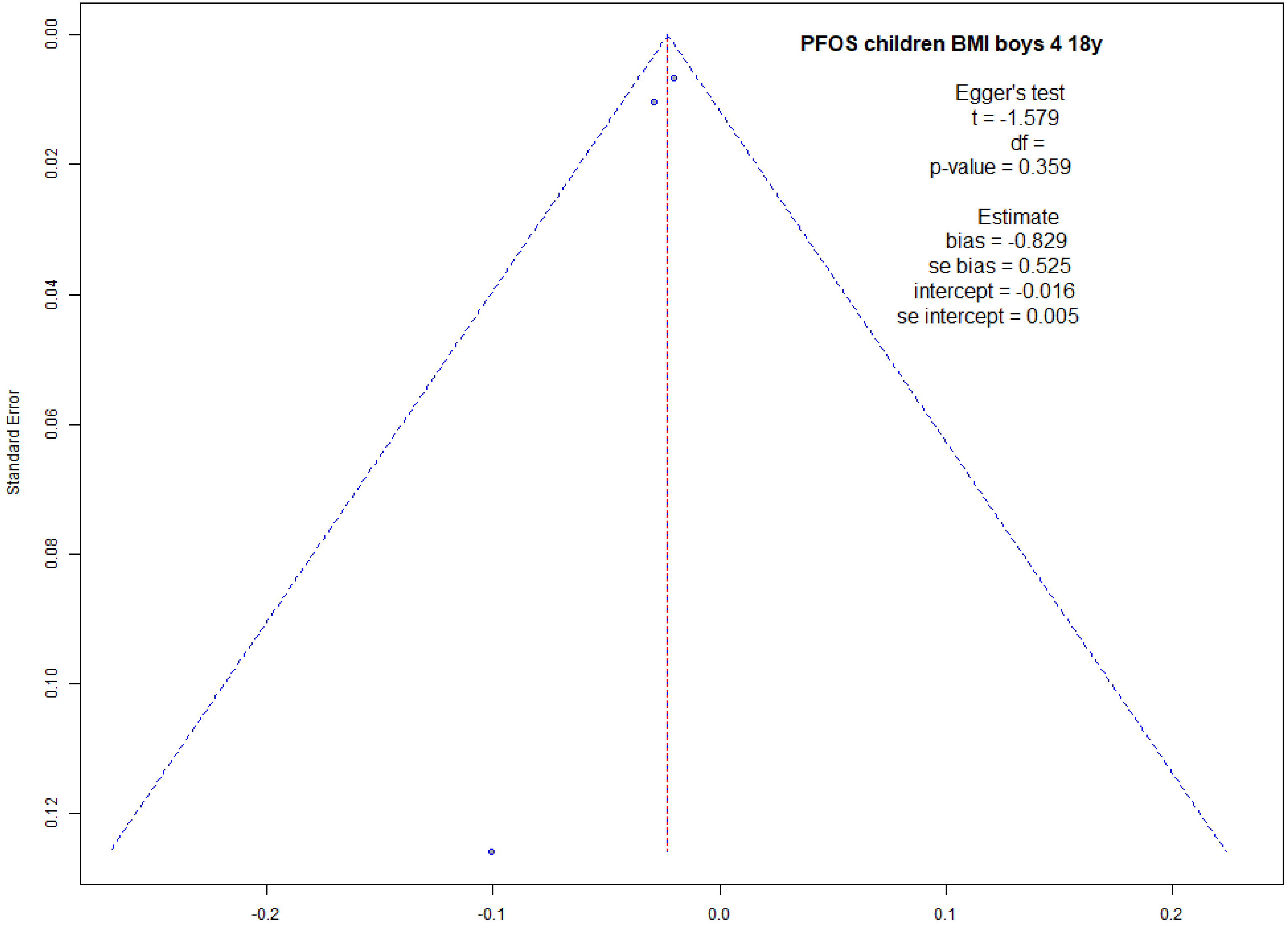
PFOS children BMI boys 4 18y
Egger's test
t = -1.579
df =
p-value = 0.359
Estimate
bias = -0.829
se bias = 0.525
intercept = -0.016
se intercept = 0.005
Standard Error
0.00
0.02
0.04
0.06
0.08
0.10
0.12
-0.2
-0.1
0.0
0.1
0.2

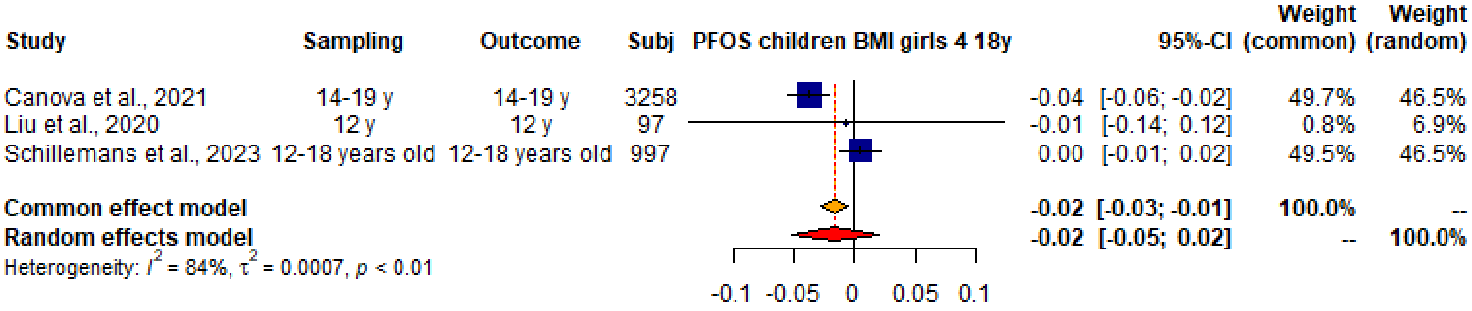
Study
Sampling
Outcome
Subj
PFOS children BMI girls 4 18y
95%-CI
Weight (common)
Weight (random)
Canova et al., 2021 14-19 y 14-19 y 3258 -0.04 [-0.06; -0.02] 49.7% 46.5%
Liu et al., 2020 12 y 12 y 97 -0.01 [-0.14; 0.12] 0.8% 6.9%
Schillemans et al., 2023 12-18 years old 12-18 years old 997 0.00 [-0.01; 0.02] 49.5% 46.5%
Common effect model -0.02 [-0.03; -0.01] 100.0% --
Random effects model -0.02 [-0.05; 0.02] -- 100.0%
Heterogeneity: I^2 = 84%, τ^2 = 0.0007, p < 0.01
-0.1 -0.05 0 0.05 0.1

Study	Sampling	Outcome	Subjects	Estimate [95% CI]
Canova et al., 2021	14-19 y	14-19 y	3258	-0.038 [-0.055; -0.022]
Liu et al., 2020	12 y	12 y	97	-0.007 [-0.137; 0.123]
Schillemans et al., 2023	12-18 years old	12-18 years old	997	0.004 [-0.012; 0.021]
**Summary**				**-0.028 [-0.072; 0.016]**

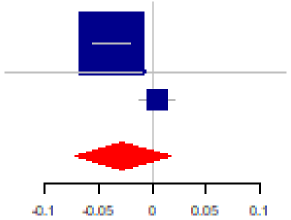

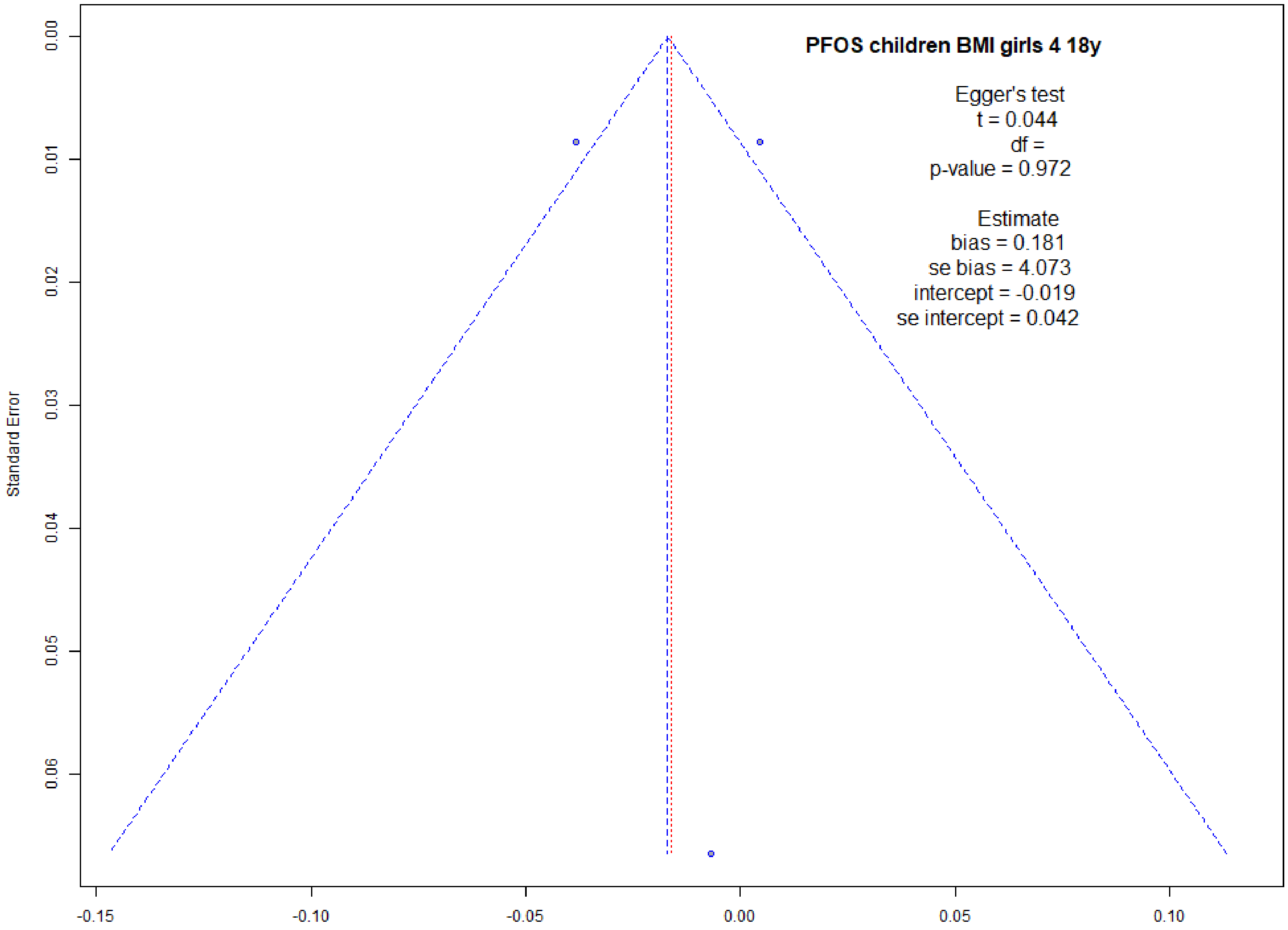
PFOS children BMI girls 4 18y
Egger's test
t = 0.044
df =
p-value = 0.972
Estimate
bias = 0.181
se bias = 4.073
intercept = -0.019
se intercept = 0.042
Standard Error
0.00
0.01
0.02
0.03
0.04
0.05
0.06
-0.15
-0.10
-0.05
0.00
0.05
0.10

PFHxS children BMI both sexes 4 18y

Study	Sampling	Outcome	Su[illegible]	95%-CI		Weight (common)	Weight (random)
Canova et al., 2021	14-19 y	14-19 y	6669	0.01	[-0.00; 0.02]	98.1%	54.1%
Scinicariello et al., 2020	3-11 y	3-11 y	600	-0.06	[-0.16; 0.04]	0.9%	20.0%
Liu et al., 2020	12 y	12 y	182	0.23	[-0.35; 0.81]	0.0%	1.0%
Karlsen et al., 2017	5 y	5 y	349	-0.09	[-0.21; 0.02]	0.7%	16.8%
Schillemans et al., 2023	12-18 years old	12-18 years old	1626	0.04	[-0.17; 0.25]	0.2%	6.5%
Vrijheid et al., 2020	6-11 years	6-11 years	1301	-0.34	[-0.77; 0.09]	0.1%	1.7%
**Common effect model**				**0.01**	**[-0.00; 0.02]**	**100.0%**	**--**
**Random effects model**				**-0.02**	**[-0.08; 0.03]**	**--**	**100.0%**

Heterogeneity: $I^2 = 35\%$, $\tau^2 = 0.0015$, $p = 0.18$

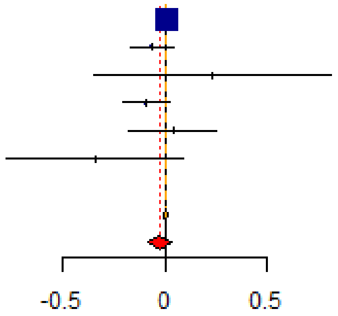

Study	Sampling	Outcome	Subjects	Estimate [95% CI]
Canova et al., 2021	14-19 y	14-19 y	6669	0.007 [-0.002; 0.017]
Scinicariello et al., 2020	3-11 y	3-11 y	600	-0.062 [-0.163; 0.039]
Liu et al., 2020	12 y	12 y	182	0.230 [-0.346; 0.806]
Karlsen et al., 2017	5 y	5 y	349	-0.091 [-0.206; 0.024]
Schillemans et al., 2023	12-18 years old	12-18 years old	1626	0.038 [-0.171; 0.247]
Vrijheid et al., 2020	6-11 years	6-11 years	1301	-0.340 [-0.774; 0.094]
**Summary**				**-0.033 [-0.116; 0.049]**

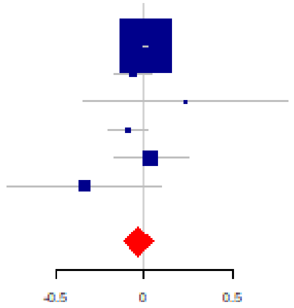

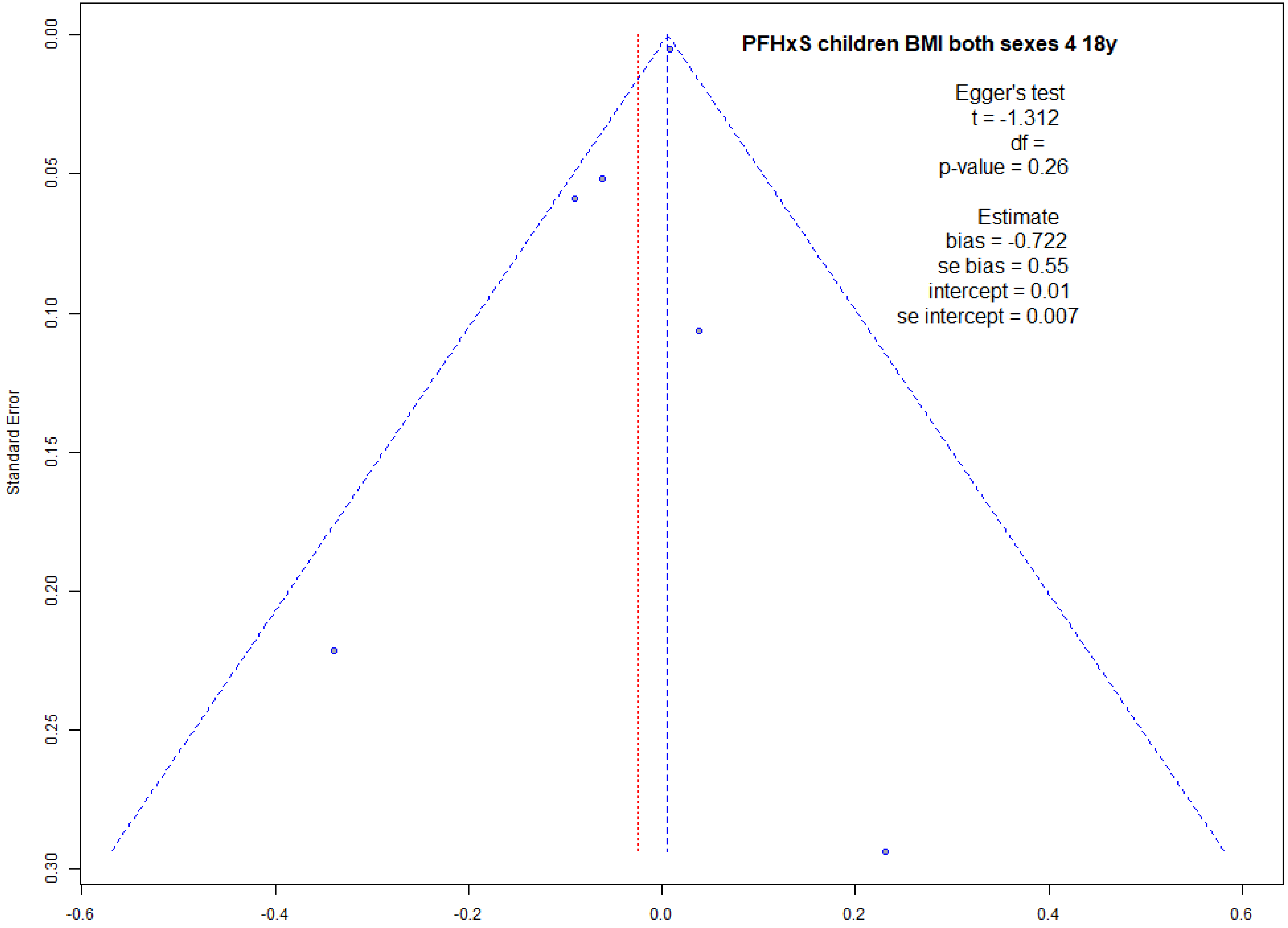
PFHxS children BMI both sexes 4 18y
Egger's test
t = -1.312
df =
p-value = 0.26
Estimate
bias = -0.722
se bias = 0.55
intercept = 0.01
se intercept = 0.007
Standard Error
0.00
0.05
0.10
0.15
0.20
0.25
0.30
-0.6
-0.4
-0.2
0.0
0.2
0.4
0.6

Gianfranco Frigerio, Chiara Matilde Ferrari, and Silvia Fustinoni

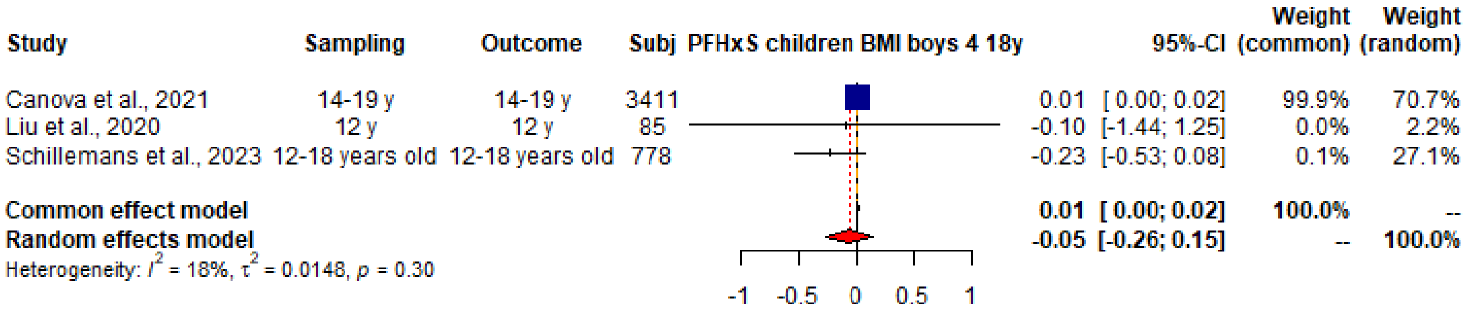

Study	Sampling	Outcome	Subjects	Estimate [95% CI]
Canova et al., 2021	14-19 y	14-19 y	3411	0.013 [0.002; 0.023]
Liu et al., 2020	12 y	12 y	85	-0.096 [-1.440; 1.248]
Schillemans et al., 2023	12-18 years old	12-18 years old	778	-0.228 [-0.531; 0.076]
**Summary**				**-0.033 [-0.162; 0.096]**

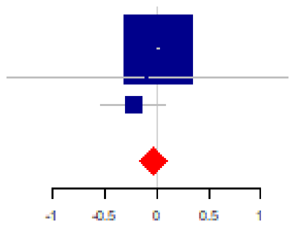

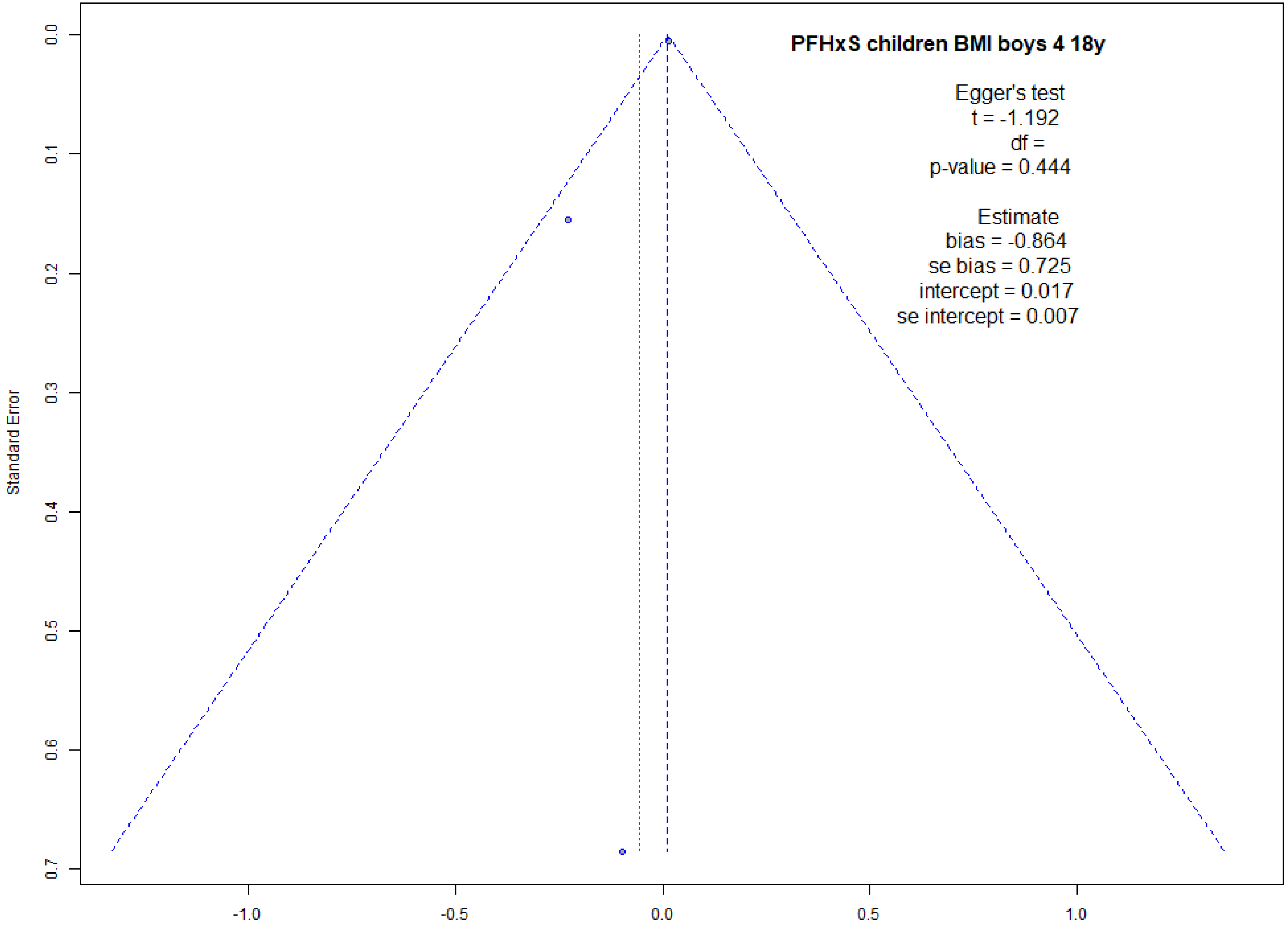
PFHxS children BMI boys 4 18y
Egger's test
t = -1.192
df =
p-value = 0.444
Estimate
bias = -0.864
se bias = 0.725
intercept = 0.017
se intercept = 0.007
Standard Error
0.0
0.1
0.2
0.3
0.4
0.5
0.6
0.7
-1.0
-0.5
0.0
0.5
1.0

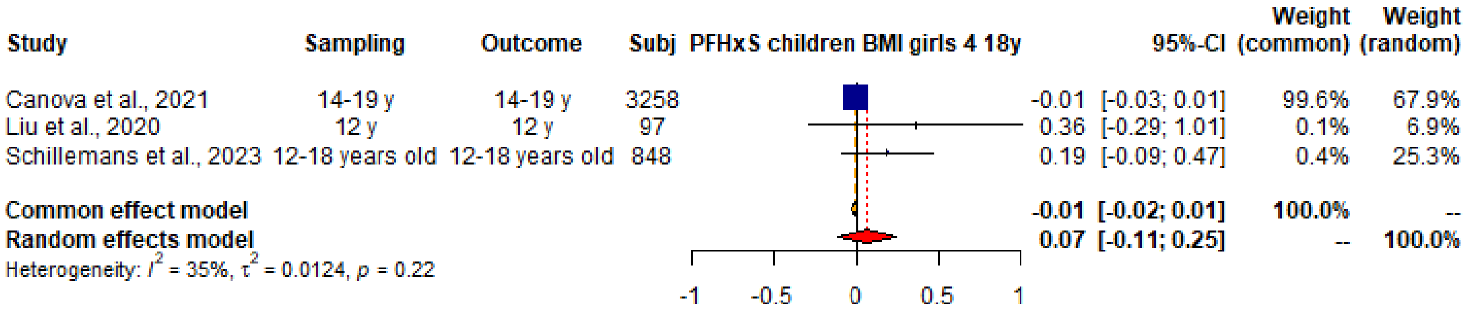
Study
Sampling
Outcome
Subj
PFHxS children BMI girls 4 18y
95%-CI
Weight (common)
Weight (random)
Canova et al., 2021 14-19 y 14-19 y 3258 -0.01 [-0.03; 0.01] 99.6% 67.9%
Liu et al., 2020 12 y 12 y 97 0.36 [-0.29; 1.01] 0.1% 6.9%
Schillemans et al., 2023 12-18 years old 12-18 years old 848 0.19 [-0.09; 0.47] 0.4% 25.3%
Common effect model -0.01 [-0.02; 0.01] 100.0% --
Random effects model 0.07 [-0.11; 0.25] -- 100.0%
Heterogeneity: I² = 35%, τ² = 0.0124, p = 0.22
-1
-0.5
0
0.5
1

Study	Sampling	Outcome	Subjects	Estimate [95% CI]
Canova et al., 2021	14-19 y	14-19 y	3258	-0.009 [-0.026; 0.009]
Liu et al., 2020	12 y	12 y	97	0.358 [-0.294; 1.011]
Schillemans et al., 2023	12-18 years old	12-18 years old	848	0.190 [-0.095; 0.475]
**Summary**				**0.040 [-0.125; 0.205]**

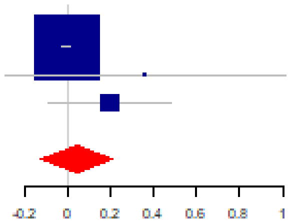

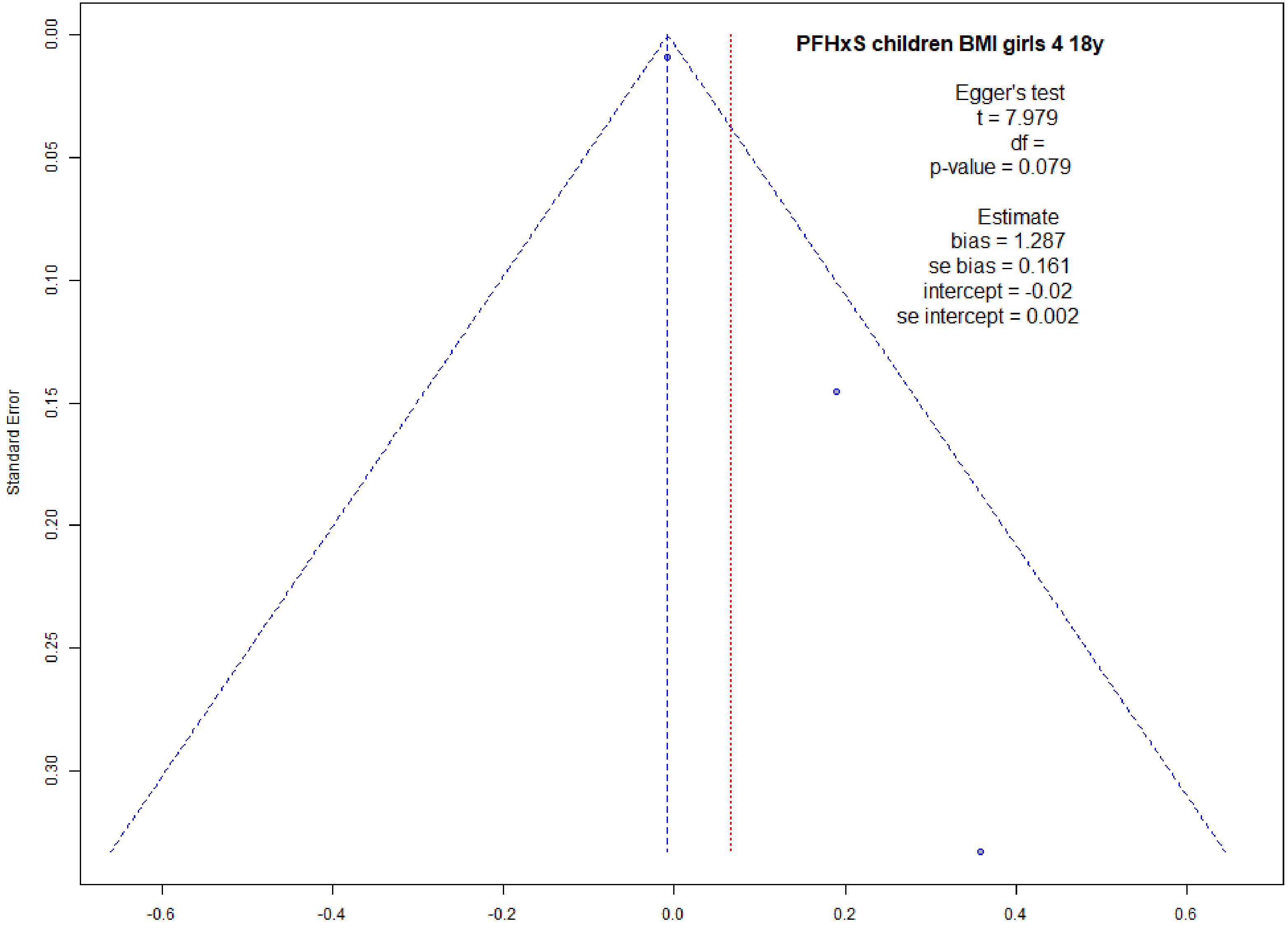
PFHxS children BMI girls 4 18y
Egger's test
t = 7.979
df =
p-value = 0.079
Estimate
bias = 1.287
se bias = 0.161
intercept = -0.02
se intercept = 0.002
Standard Error
0.00
0.05
0.10
0.15
0.20
0.25
0.30
-0.6
-0.4
-0.2
0.0
0.2
0.4
0.6

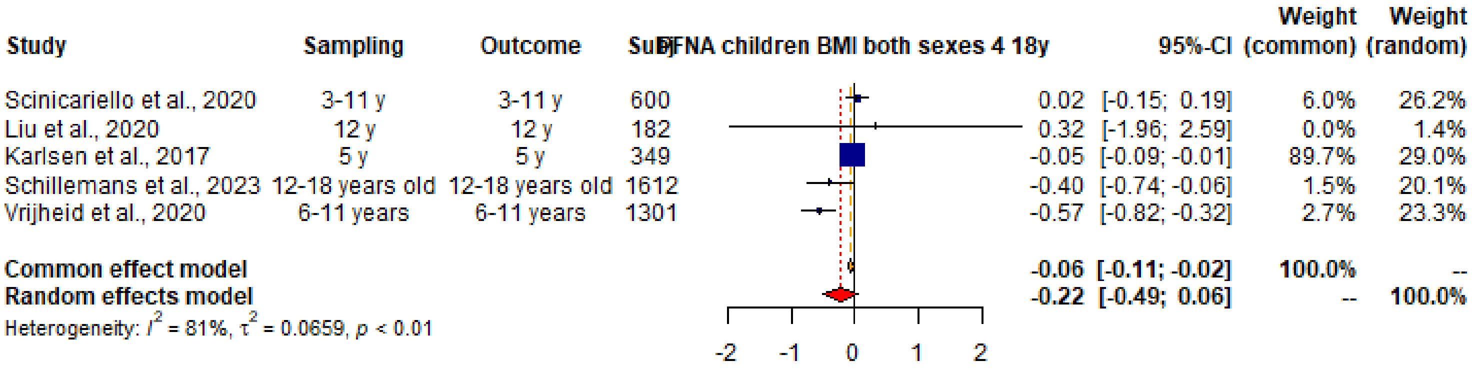
Study | Sampling | Outcome | Sub | PFNA children BMI both sexes 4 18y | 95%-CI | Weight (common) | Weight (random)
Scinicariello et al., 2020 | 3-11 y | 3-11 y | 600 | 0.02 | [-0.15; 0.19] | 6.0% | 26.2%
Liu et al., 2020 | 12 y | 12 y | 182 | 0.32 | [-1.96; 2.59] | 0.0% | 1.4%
Karlsen et al., 2017 | 5 y | 5 y | 349 | -0.05 | [-0.09; -0.01] | 89.7% | 29.0%
Schillemans et al., 2023 | 12-18 years old | 12-18 years old | 1612 | -0.40 | [-0.74; -0.06] | 1.5% | 20.1%
Vrijheid et al., 2020 | 6-11 years | 6-11 years | 1301 | -0.57 | [-0.82; -0.32] | 2.7% | 23.3%
Common effect model | -0.06 | [-0.11; -0.02] | 100.0% | --
Random effects model | -0.22 | [-0.49; 0.06] | -- | 100.0%
Heterogeneity: I^2 = 81%, τ^2 = 0.0659, p < 0.01
-2 -1 0 1 2

Study	Sampling	Outcome	Subjects	Estimate [95% CI]
Scinicariello et al., 2020	3-11 y	3-11 y	600	0.024 [-0.146; 0.195]
Liu et al., 2020	12 y	12 y	182	0.317 [-1.957; 2.591]
Karlsen et al., 2017	5 y	5 y	349	-0.050 [-0.094; -0.006]
Schillemans et al., 2023	12-18 years old	12-18 years old	1612	-0.397 [-0.737; -0.057]
Vrijheid et al., 2020	6-11 years	6-11 years	1301	-0.570 [-0.823; -0.317]
**Summary**				**-0.328 [-0.612; -0.044]**

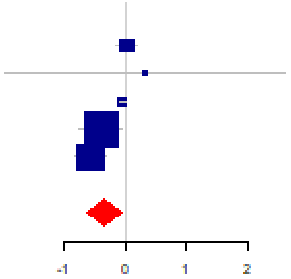

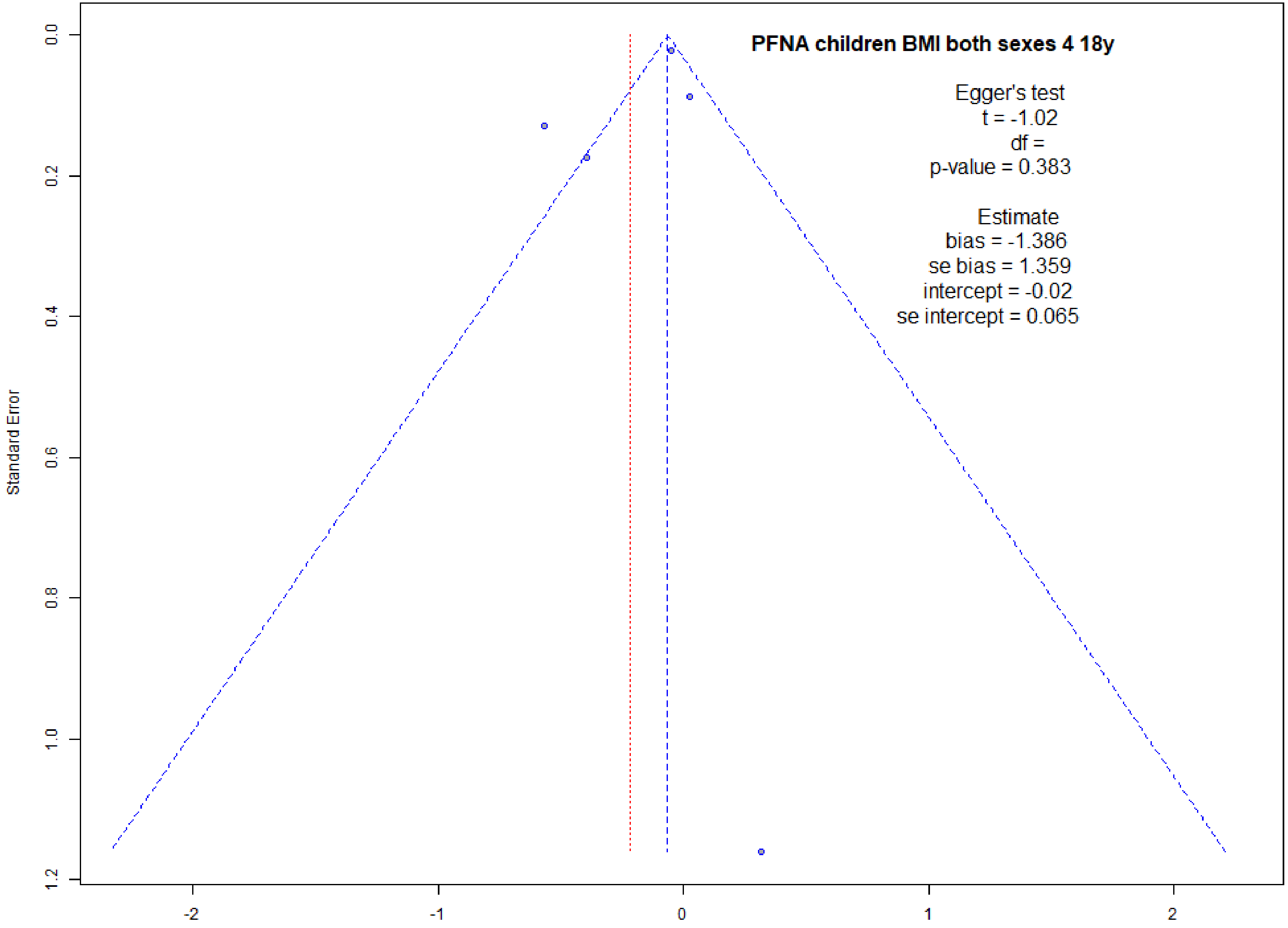
PFNA children BMI both sexes 4 18y
Egger's test
t = -1.02
df =
p-value = 0.383
Estimate
bias = -1.386
se bias = 1.359
intercept = -0.02
se intercept = 0.065
Standard Error
0.0
0.2
0.4
0.6
0.8
1.0
1.2
-2
-1
0
1
2

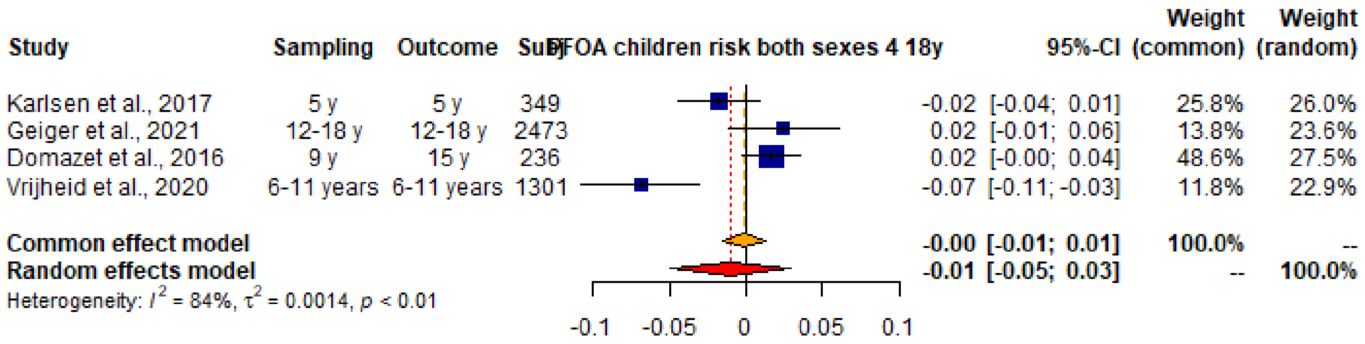
Study
Sampling
Outcome
Sub
PFOA children risk both sexes 4 18y
95%-CI
Weight (common)
Weight (random)
Karlsen et al., 2017 5 y 5 y 349 -0.02 [-0.04; 0.01] 25.8% 26.0%
Geiger et al., 2021 12-18 y 12-18 y 2473 0.02 [-0.01; 0.06] 13.8% 23.6%
Domazet et al., 2016 9 y 15 y 236 0.02 [-0.00; 0.04] 48.6% 27.5%
Vrijheid et al., 2020 6-11 years 6-11 years 1301 -0.07 [-0.11; -0.03] 11.8% 22.9%
Common effect model -0.00 [-0.01; 0.01] 100.0% --
Random effects model -0.01 [-0.05; 0.03] -- 100.0%
Heterogeneity: I² = 84%, τ² = 0.0014, p < 0.01
-0.1
-0.05
0
0.05
0.1

Study	Sampling	Outcome	Subjects	Estimate [95% CI]
Karlsen et al., 2017	5 y	5 y	349	-0.018 [-0.044; 0.009]
Geiger et al., 2021	12-18 y	12-18 y	2473	0.024 [-0.012; 0.060]
Domazet et al., 2016	9 y	15 y	236	0.017 [-0.003; 0.036]
Vrijheid et al., 2020	6-11 years	6-11 years	1301	-0.069 [-0.108; -0.030]
**Summary**				**-0.007 [-0.055; 0.041]**

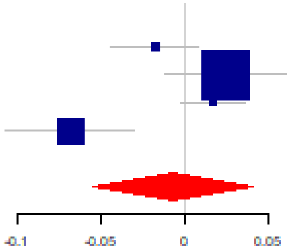

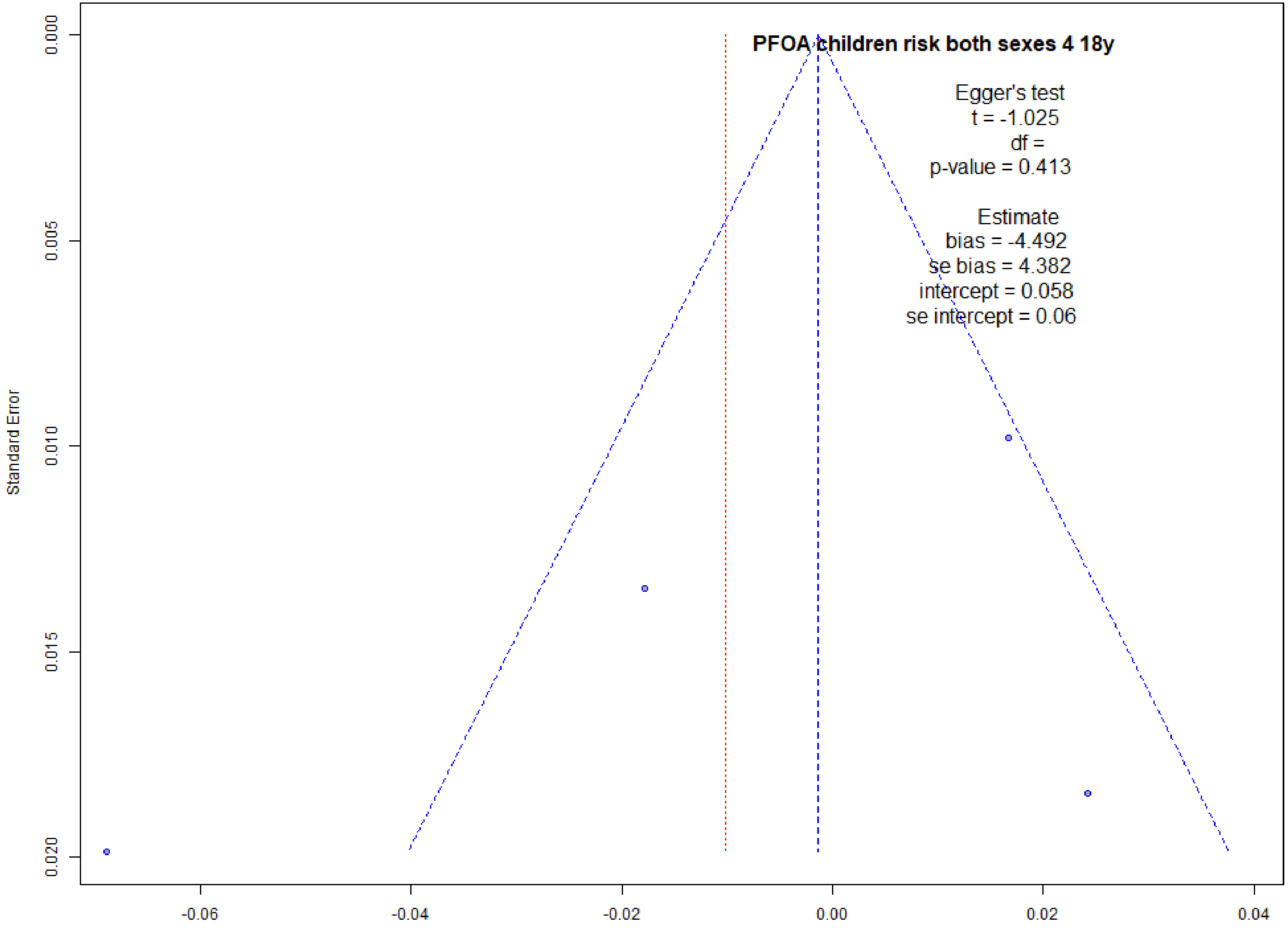
PFOA children risk both sexes 4 18y
Egger's test
t = -1.025
df =
p-value = 0.413
Estimate
bias = -4.492
se bias = 4.382
intercept = 0.058
se intercept = 0.06
Standard Error
0.000
0.005
0.010
0.015
0.020
-0.06
-0.04
-0.02
0.00
0.02
0.04

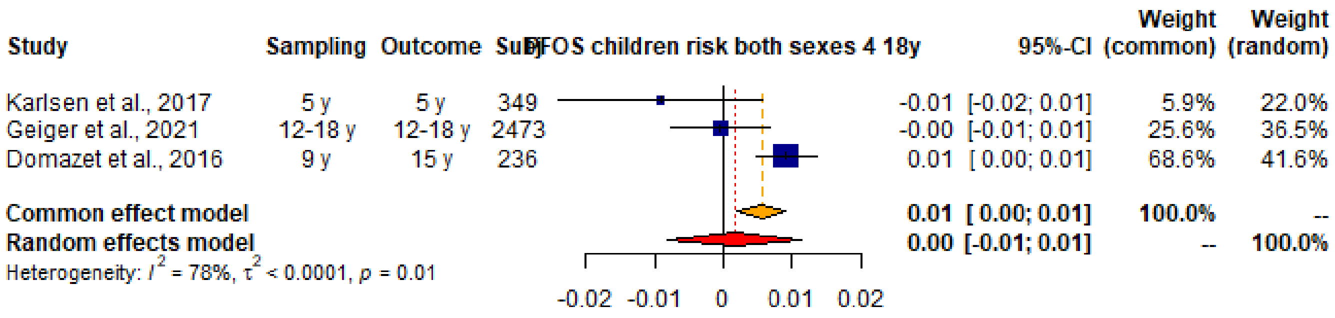
PFOS children risk both sexes 4 18y
Study
Sampling
Outcome
Sub
95%-CI
Weight (common)
Weight (random)
Karlsen et al., 2017 5 y 5 y 349 -0.01 [-0.02; 0.01] 5.9% 22.0%
Geiger et al., 2021 12-18 y 12-18 y 2473 -0.00 [-0.01; 0.01] 25.6% 36.5%
Domazet et al., 2016 9 y 15 y 236 0.01 [0.00; 0.01] 68.6% 41.6%
Common effect model 0.01 [0.00; 0.01] 100.0% --
Random effects model 0.00 [-0.01; 0.01] -- 100.0%
Heterogeneity: I^2 = 78%, τ^2 < 0.0001, p = 0.01
-0.02 -0.01 0 0.01 0.02

Study	Sampling	Outcome	Subjects	Estimate [95% CI]
Karlsen et al., 2017	5 y	5 y	349	-0.009 [-0.024; 0.006]
Geiger et al., 2021	12-18 y	12-18 y	2473	0.000 [-0.008; 0.007]
Domazet et al., 2016	9 y	15 y	236	0.009 [0.005; 0.013]
**Summary**				**-0.001 [-0.014; 0.012]**

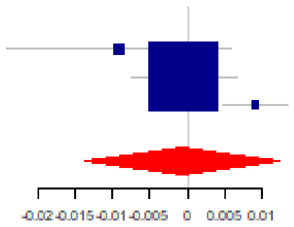

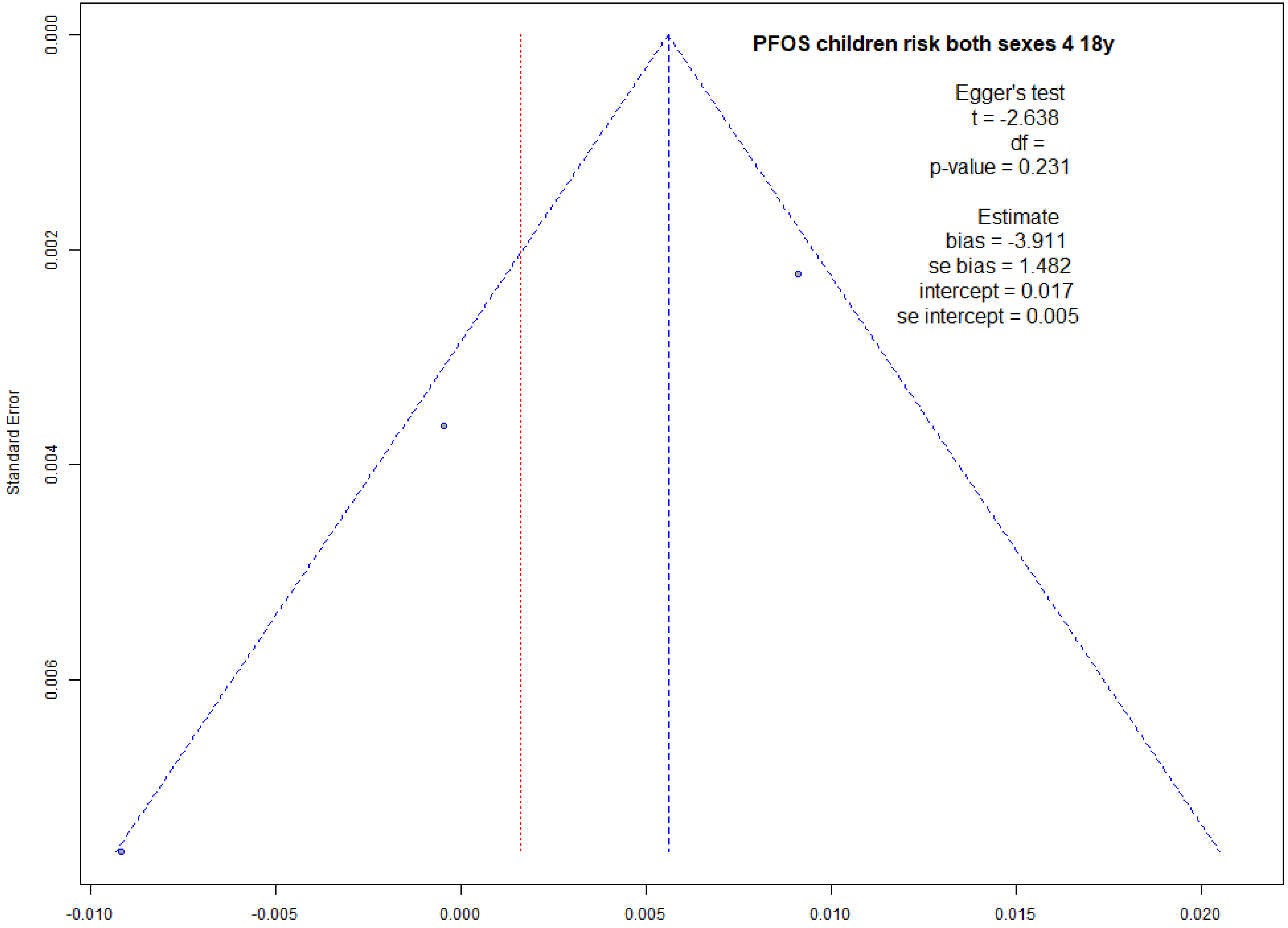
PFOS children risk both sexes 4 18y
Egger's test
t = -2.638
df =
p-value = 0.231
Estimate
bias = -3.911
se bias = 1.482
intercept = 0.017
se intercept = 0.005
Standard Error
0.000
0.002
0.004
0.006
-0.010
-0.005
0.000
0.005
0.010
0.015
0.020
